# Cobalt(0)-Catalyzed Isomerization of Allylamines Promoted by Monodentate Benzofuran Phosphines

**DOI:** 10.1021/jacs.5c09017

**Published:** 2025-08-19

**Authors:** Sebastian Ahrens, Rafał Kusy, Anke Spannenberg, Thanh H. Vuong, Jabor Rabeah, Bernhard M. E. Russbueldt, Johannes Panten, Haijun Jiao, Kathrin Junge, Matthias Beller

**Affiliations:** † 28392 Leibniz-Institut für Katalyse e.V., Albert-Einstein-Straße 29a, 18059 Rostock, Germany; ‡ State Key Laboratory of Low Carbon Catalysis and Carbon Dioxide Utilization, Lanzhou Institute of Chemical Physics (LICP), Chinese Academy of Sciences, Lanzhou 730000, P. R. China; § 10784 Symrise AG, Mühlenfeldstraße 1, 37603 Holzminden, Germany

## Abstract

Isomerization of C–C double bonds is an efficient tool for converting bulk olefins into high-value compounds. Herein we describe a newly developed homogeneous cobalt-based system which enables the isomerization of allylamines to enamines with similar activity and selectivity to noble metal catalysts. The catalyst activity was bound to the presence of furan-2-yl substituents of the phosphine ligand, while the selectivity was increased with the number of benzofuran-2-yl groups. The resulting cobalt(0) catalysts allowed the isomerization of various aliphatic and aromatic allylamines, including the synthesis of industry-relevant products with high catalytic activity. Potential industrial application of our system was substantiated by the scale-up experiment, which provided 311 g of citronellal in 96% yield. We proposed a plausible mechanism based on EPR and NMR analysis combined with deuterium labeling, radical trapping experiments, and kinetic studies.

## Graphical abstract

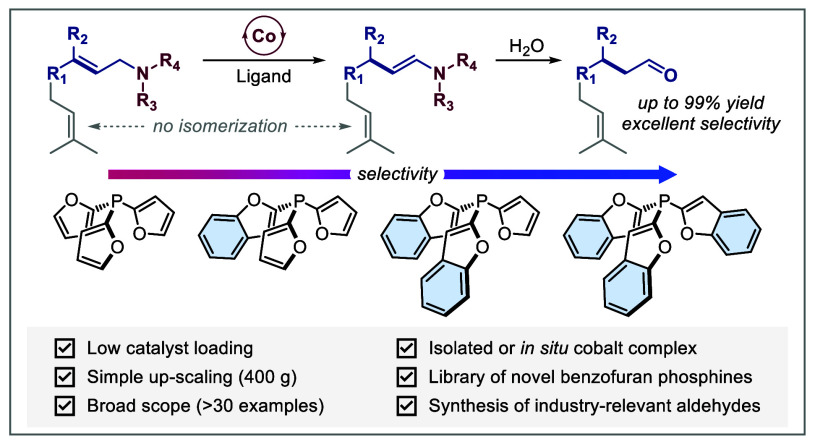

## Introduction

Transition-metal-catalyzed isomerization of olefins is an atom- and energy-efficient transformation which enables conversion of accessible low-cost alkenes into higher-value products, which are in demand in pharmaceutical, food, flavor, and fragrance industries.^1^ Although a wide range of systems for olefin isomerization has been reported in the last decades, the vast majority of efficient and industry-relevant catalysts are based on noble metals such as Pd,^2^ Ir,^3^ Pt,^4^ and Ru.^5^ However, more recently, the attention has shifted to base metals such as Fe,^6^ Co,^7,8^ and Ni,^9,10^ due to their higher abundance, lower cost, and lower toxicity.^11^

In particular, the application of Co complexes as catalysts in olefin isomerization reactions is of growing interest (Scheme 1A). Liu (Scheme 1A1) and Findlater (Scheme 1A2) employed Co pincer complexes in the isomerization of methylenecyclohexane derivatives, allowing the stereoselective synthesis of trisubstituted alkenes.^12^ In 2020, Diver reported a radical-triggered double isomerization of disubstituted-1,3-dienes catalyzed by cobaloxime complexes (Scheme 1A3).^13^ In 2020, (*Z*)-selective isomerization of terminal olefins was disclosed by Holland (Scheme 1A4).^14^ More recently, Lu presented a Co-catalyzed multipositional isomerization of conjugated dienes via a metal-hydride mechanism. While significant progress has been made in olefin isomerization, the application of Co-based catalysts remains limited to the transformation of relatively simple substrates, such as terminal or unfunctionalized olefins. In particular, the isomerization of internal olefins containing heteroatoms (O, N, or S), (e.g., the transformation of allylamines to enamines) remains a challenge for any base-metal catalyst.^15^ Otsuka et al. reported isomerization of (*E*)-allylamines to the corresponding (*E*)-allylenamines catalyzed by Co­(I)-hydride, but the presented scope was limited to two examples.^16^

**Scheme 1:**
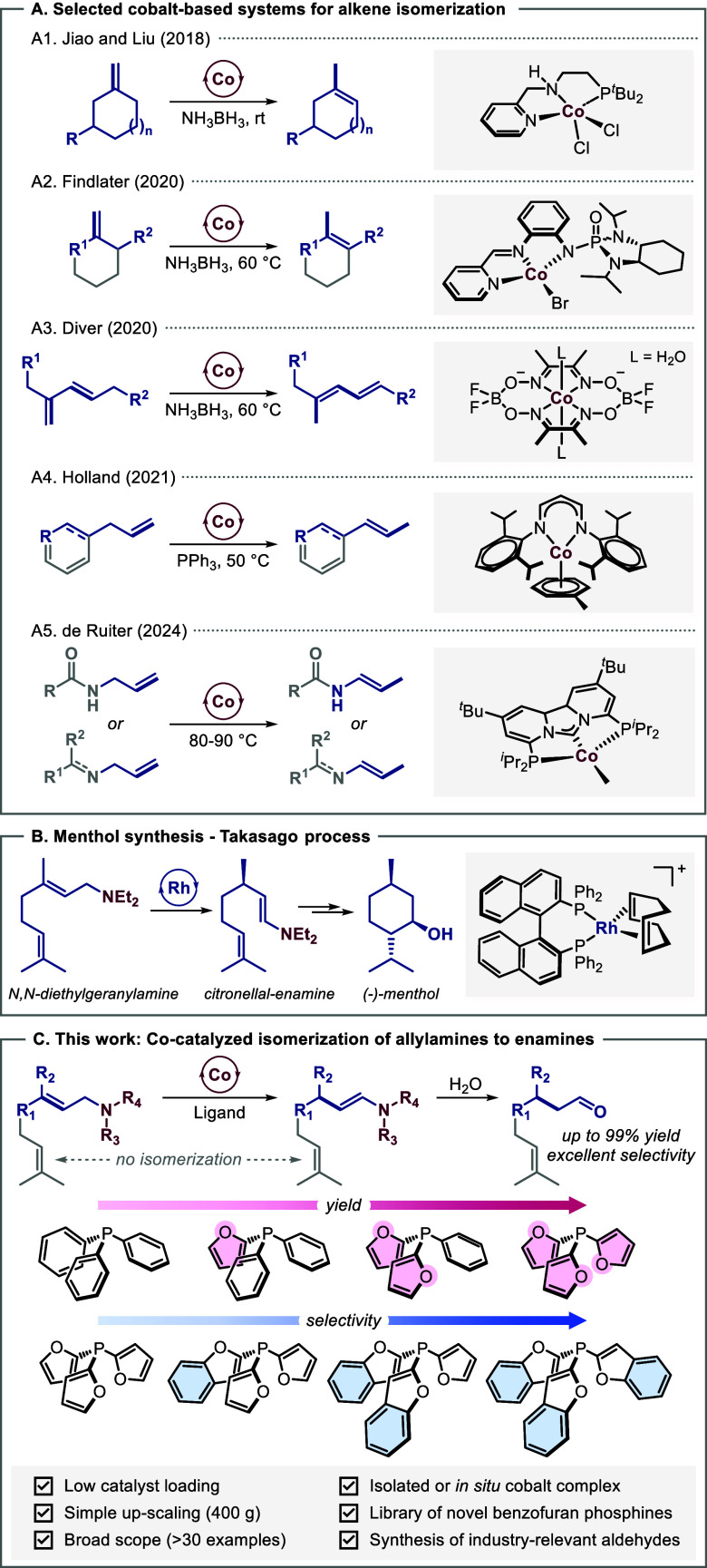
Transition-Metal-Catalyzed Isomerization of Olefins

In 2024, the group of de Ruiter developed a neutral PC_NHC_P Co­(I)-pincer complex for the isomerization of terminal (*E*)-allylamines and (*E*)-allylamides (Scheme 1A5).^17^ The importance of allylamine isomerization is reflected in the Takasago process, which allows menthol synthesis and constitutes one of the largest industrial applications of homogeneous asymmetric catalysis (Scheme 1B).^18^ It encompasses the Rh-catalyzed isomerization of *N*,*N*-diethylgeranylamine (**1a**) to citronellal enamine (**1b**), which we chose as our model reaction. Herein, we report the development of a novel *in situ* generated Co(0) catalyst from tri­(benzofuran-2-yl)­phosphine as a ligand, which is capable of converting structurally diverse and industry-relevant aliphatic and aromatic allylamines into the corresponding enamines in up to quantitative yields (Scheme 1C).

## Results and Discussion

### Catalyst Development and Optimization of the Reaction System

We initiated our investigations on the Co-catalyzed isomerization of allylamines by screening Co pincer complexes bearing different (PNP, NNN, and NNP) pincer ligands (see SI section 2 for details, Table S1). Recently, these Co complexes have been described as catalysts for the isomerization of various olefins.^19^ Unfortunately, only negligible reactivity was observed for the isomerization of allylamines in the presence of these Co pincer complexes. Hence, we focused on *in situ*-generated Co catalysts using commercially available and newly developed bi- and monodentate ligands. After testing more than 40 different phosphines (see SI section 3), we discovered a surprisingly high activity for tri­(furan-2-yl)­phosphine (TFP, **L1**).

The combination of Co­(OAc)_2_, DIBAL-H, and **L1** performed well for the isomerization of *N*,*N*-diethylgeranylamine **1a** and yielded 81% of **1b** with good selectivity (Scheme 2). Other aromatic phosphines bearing heterocycles, such as thiophene **L2** or pyridine **L3**, led to the deactivation of the catalyst, while the use of triphenylphosphine **L4** gave a much lower yield. To evaluate the effect of the furan moiety in more detail, we prepared a library of furan-based phosphines **L4**–**L12**. The yield increased with the replacement of the benzene ring with furyl one **L4**–**L6**, confirming the crucial importance of this moiety.

**Scheme 2:**
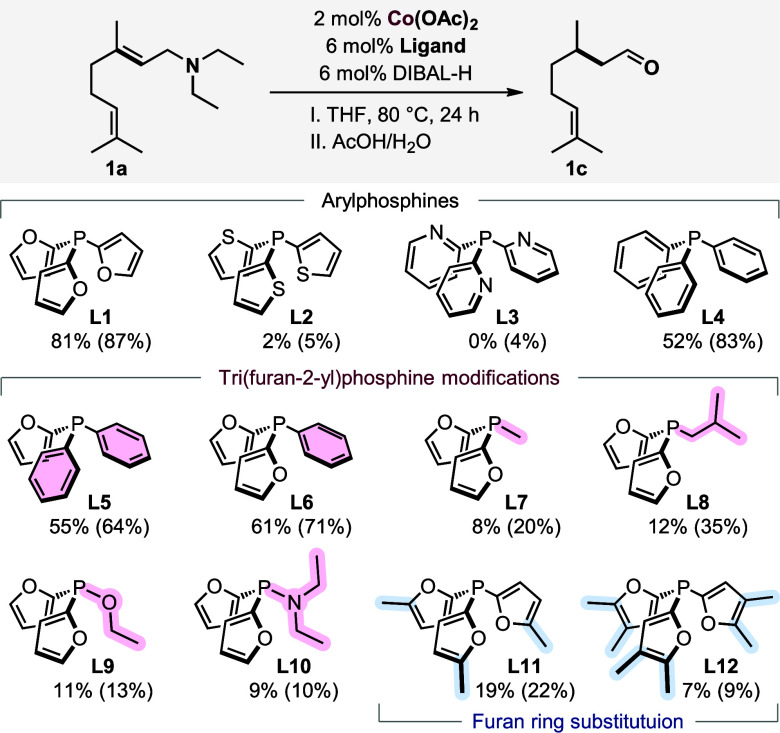
Co-Catalyzed Isomerization of Allylamine **1a**: Testing Different Phosphines^a^

a Reaction conditions: 2.5 mmol of **1a**, 1.5 mL of THF, Co­(OAc)_2_, ligand, DIBAL-H, 80 °C, 24 h. After catalysis, **1b** was hydrolyzed by the addition of 1.5 mL of a 5% acetic acid solution, yielding **1c**.
b Conversion of **1a** and yield of **1c** were determined by GC analysis using *n*-hexadecane as an internal standard. The numbers in parentheses represent the conversion.

In the case of difurylphosphines bearing alkyl substituents (−Me, **L7**; −*i*Bu, **L8**), a significantly lower reactivity of the catalyst was detected compared to the aromatic phosphines. A similar activity was observed while using **L9** and **L10**, bearing a diethylamine or an ethoxy unit, respectively. Finally, the presence of a monosubstituted (**L11**) or disubstituted furan ring (**L12**) with a methyl group suppressed the catalytic activity, probably due to steric reasons.

Interestingly, the performance of the Co catalyst was considerably improved by the exchange of one furan ring with a benzofuran moiety (**L13**), leading to the complete conversion of **1a** to the desired product **1b** in 94% yield (Table S3, entry 13). To understand the superior performance of **L13** compared to **L1**, we altered the electronic and steric properties of TFP (Scheme 3). To our delight, we observed that further replacement of furyl substituents with benzofuryl ones (**L14**, **L15**) led to an increase in selectivity. Moreover, the use of 0.1 mol % Co­(acac)_2_ and tri­(benzofuran-2-yl)­phosphine **L15** as a ligand at 100 °C enabled the full conversion of **1a** and production of **1b** in almost quantitative yield (SI section 6).

**Scheme 3:**
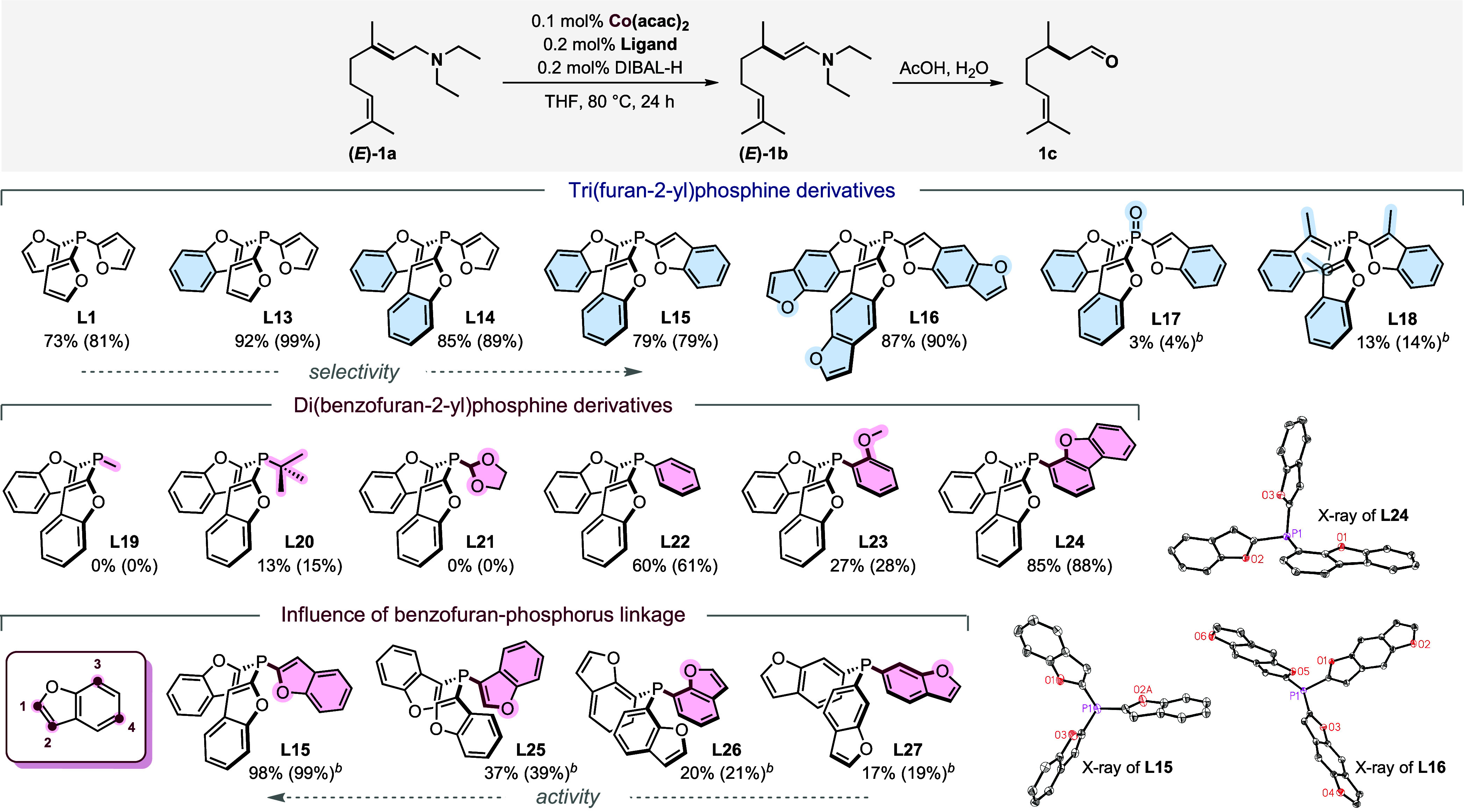
Overview of Synthesized and Tested Benzofuran Phosphines^a^

a Reaction conditions: 2.5 mmol of **1a**, 1.5 mL of THF, Co­(acac)_2_, selected phosphine, DIBAL-H, 80 °C, 24 h. Conversion of **1a** and yield of **1c** were determined by GC analysis using *n*-hexadecane as an internal standard. The numbers in parentheses represent the conversion of **1a**. In the molecular structures of **L15**, **L16**, and **L24**, displacement ellipsoids correspond to 30% probability, and hydrogen atoms have been omitted for clarity.
b Catalyst loading: 0.5 mol % Co­(acac)_2_, 1 mol % benzofuran phosphine and DIBAL-H.

Of note, the application of benzofuran phosphines such as **L15** in homogeneous catalysis has been rarely described.^20,21^ The ligand **L15**, previously reported by the Santelli^20^ group, was characterized by X-ray diffraction (see SI section 8). The employment of tris­(benzo­[1,2-*b*:4,5-*b*′]­difuran-2-yl)­phosphine **L16** gave slightly lower selectivity. Only traces of **1b** were detected for phosphine oxide **L17**,^22^ which clearly demonstrates that oxidation of phosphine **L15** leads to the deactivation of the corresponding Co catalyst. Moreover, the presence of the methyl group at the C3 position of the benzofuran ring in phosphine **L18** hampered catalytic activity significantly, probably due to steric hindrance. A similar trend was observed for **L11** and **L12** (Scheme 2).

Subsequently, we investigated the influence of the replacement of one benzofuran-2-yl moiety with different substituents. In the case of alkyl groups like methyl (**L19**) or dioxolane (**L21**), no catalytic activity was observed, which was consistent with the previous observations for **L7** and **L8** (Scheme 2). Better results were observed after the introduction of aryl substituents (**L22**–**L24**). The presence of an sp^2^ oxygen atom seems to be crucial (**L15**, **L24**), since the ligands containing sp^3^ oxygen atoms (**L21**, **L23**) were inefficient. Interestingly, **L24** exhibited higher activity compared to **L15**, albeit with inferior selectivity.

Once we had proved that benzofuran phosphines were the most suitable ligands, we examined the influence of the benzofuran-phosphorus linkage. Much lower activity was observed for phosphine with a phosphorus atom linked to the benzofuran rings at the C3 position (**L25**). The performance of phosphines with a phosphorus atom bonded to a benzene ring of benzofuran (**L26**, **L27**) was comparable to the performance of triphenylphosphine **L4**. The above examples indicate that the vicinity of an sp^2^ oxygen atom two bonds away from the phosphorus atom is essential for catalytic activity.

Having identified **L15** as the most efficient ligand, the influence of crucial reaction parameters such as catalyst loading, precursors, ligand ratio, solvents, and reductants was investigated (Table 1). Co­(acac)_2_ and Co­(acac)_3_ showed enhanced reactivity compared to other Co­(II) precursors such as CoCl_2_, CoBr_2_, Co­(OTf)_2_, or Co­(BF_4_)_2_ (see SI section 6 for details). In some cases, decomposition of the resulting Co complexes was indicated by a brown precipitate after catalysis.

**Table 1:**
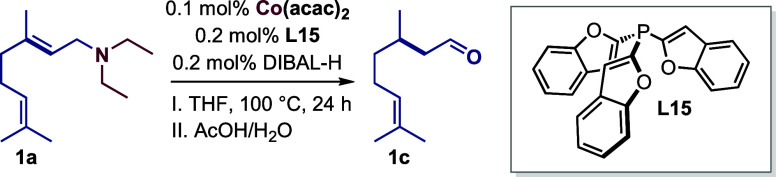
Co-Catalyzed Isomerization of Allylamine **1a**: Optimization of the Reaction Conditions

entry	changes from standard conditions	yield [%]^a^
1^b^	none	98 (99)
2^c^	Co(acac)_3_ as Co precursor	97 (99)
3	without Co(acac)_2_	traces
4	without **L15**	0 (5)
5	without DIBAL-H	traces
6	only using **L15** as catalyst	–
7^d^	H_2_ was used as the H source	traces
8	toluene was used as a solvent	21 (23)
9^e^	0.075 mol % [Co], 2-MeTHF, 120 °C	96 (98)

a Conversion of **1a** and yield of **1b** were determined by GC analysis using *n*-hexadecane as an internal standard.

b Reaction conditions: 2.5 mmol of **1a**, 100 °C, 24 h.

c Catalyst: 0.1 mol % Co­(acac)_3_, 0.2 mol % **L15**, 0.3 mol % DIBAL-H.

d 20 bar H_2_

e Optimized reaction conditions: 1.5 mL of 2-MeTHF, 0.075 mol % Co­(acac)_2_, 0.15 mol % DIBAL-H, 0.15 mol % **L15**, 120 °C, 24 h.

Due to the slightly better catalytic performance and the lower usage of reductant equivalents for Co­(acac)_2_ compared to Co­(acac)_3_, we continued our investigations with Co­(acac)_2_ (Table 1, entry 1). Furthermore, the addition of different reductants such as DIBAL-H, AlEt_3_, LiAlH_4_, or LiAlH­[O^*t*^Bu]_3_ led to an active Co catalyst (SI section 6). The best reactivity of the *in situ* Co catalyst was achieved with two equivalents of DIBAL-H. Notably, the active Co(0) catalyst was not generated under a molecular hydrogen atmosphere (Table 1, entry 7).

Any further alterations of the optimized catalyst system did not provide the desired product (Table 1, entries 3–6), indicating that all components are crucial to form the active catalytic species. Of note, by increasing the temperature, we were able to reduce the catalyst amount to 0.075 mol % (Table 1, entry 9). To the best of our knowledge, this is one of the lowest base-metal catalyst loadings reported for olefin isomerization reactions.^23^

Having optimal reaction conditions for the isomerization of **1a**, we identified and analyzed side-products to better understand the reaction course. **L1** was used as a less selective ligand in larger-scale reactions to allow isomer isolation. The byproducts were analyzed by 2D-NMR spectroscopy, and their signals were assigned by GC analysis (see SI section 7 for details). In total, five internal olefin isomers (**1ba**–**1bd**) and four enamine isomers (**1be**–**1bi**) could be characterized (Scheme 4A).

**Scheme 4:**
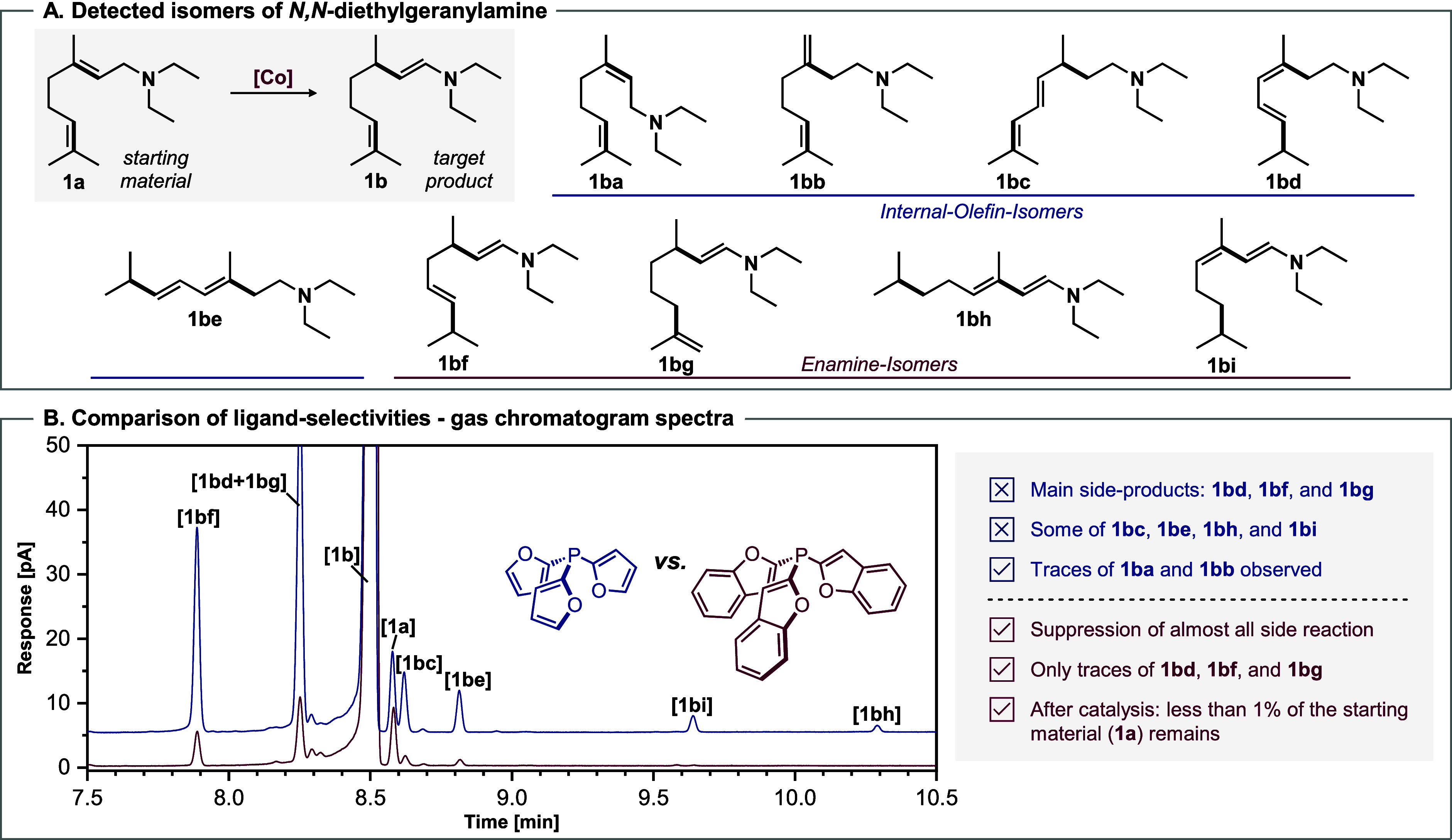
Suppressed or Promoted Side Reactions of **1a**: Comparison of **L1** and **L15** ^a^

a (A) Identified isomers of **1a** after catalysis. (B) Comparison of selectivities of ligands **L1** and **L15**: GC spectra of product mixtures after the Co-catalyzed isomerization. Reaction conditions: 2.5 mmol of **1a**, 1.5 mL of THF, 1 mol % Co­(acac)_2_, 2 mol % DIBAL-H/ligand, 100 °C.

It is noteworthy that the formation of isomers **1be**, **1bh**, and **1bi** is completely suppressed using **L15** in comparison to **L1**. Moreover, the production of **1bd**, **1bf**, and **1bg** is significantly lower for **L15**, compared to **L1**. In general, tri­(benzofuran-2-yl)­phosphine **L15** effectively prevents (*Z*)–(*E*) isomerization and chain-walking of double bonds of **1a**, which makes this ligand particularly interesting for industrial applications.

### Substrate Scope and Limitations

We explored the scope and limitations of the optimized system, starting with an investigation of the effects of different N-substituents on the isomerization of allylamines. Thus, several geranylamine derivatives bearing alkyl or aryl residues were prepared (**2a**–**10a**, Scheme 5). Compared to the industrial benchmark substrate diethyl-geranylamine (**1a**), we observed full conversion for other linear alkyl geranylamine derivatives, e.g., ethyl (**2a**), ethyl methyl (**3a**), dimethyl (**4a**), and isopropyl (**5a**) geranylamine. However, the regioselectivity decreased slightly for the methyl-substituted derivatives **3a**, **4a**, and **5a**. In the case of cyclic aliphatic amines like pyrrolidine (**6a**) and morpholine (**7a**) derivatives, we observed lower conversions. No conversion of the *N*,*N*-diaryl derivatives **8a** and **9a** was achieved, even though a significantly higher catalyst loading and harsher reaction conditions (120 °C) were applied. Nevertheless, even well-established Ru and Rh catalysts failed to convert the aryl derivative **8a** to the corresponding enamine.^24^ Overall, the screening of different N-substituted geranylamine derivatives revealed the importance of a suitable amine moiety.

**Scheme 5:**
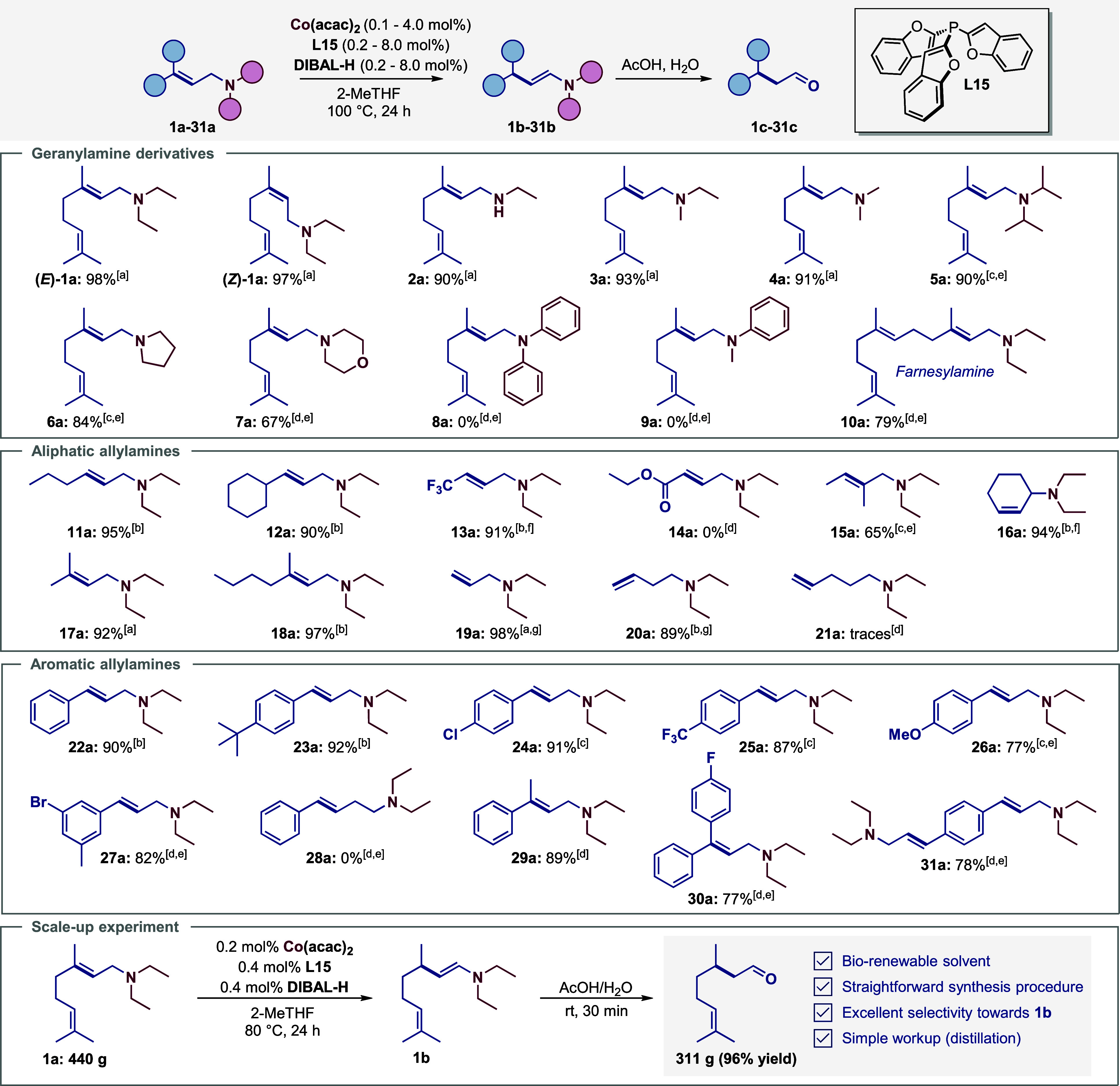
Substrate Scope: Co-Catalyzed Isomerization of Various Allylamine Derivatives^a^

a Values below the structures represent the isolated yield(s) of the corresponding aldehydes. Reaction conditions: substrate (0.5–2.5 mmol, see SI section 10 for details), 2-MeTHF or THF, 100 °C, 24 h. Note: Product **10c** represents the sum of the isomers. [a] Catalyst: 0.1 mol % Co­(acac)_2_, 0.2 mol % **L15**, and DIBAL-H. [b] Catalyst: 1 mol % Co­(acac)_2_, 2 mol % **L15**, and DIBAL-H. [c] Catalyst: 2 mol % Co­(acac)_2_, 4 mol % **L15**, and DIBAL-H. [d] Catalyst: 4 mol % Co­(acac)_2_, 8 mol % **L15**, and DIBAL-H. [e] Reaction time: 48 h. [f] Isolated as an enamine. [g] GC yield.

Subsequently, we prepared several aliphatic (*E*)-allylamines containing diethylamine residues **10a**–**21a** and systematically studied their isomerization (Scheme 5). Notably, we selectively isomerized the highly challenging and industry-relevant farnesal-derivative **10a**, obtaining **10c** in 79% yield. Furthermore, internal (*E*)-allylamine derivatives with sterically demanding substituents (**11a**–**13a**) were converted to their corresponding enamines with low catalyst loading and high selectivity. The isomerization of the hex-2-enamine derivative **11a** and the cyclohexane derivative **12a** was realized with only 0.2 mol % catalyst amount. However, our system was incompatible with allylamine bearing ester group **14a**, as were most base metal catalysts described in the literature. We were also able to achieve isomerization of the challenging substrates **15a**, **16a**, or **18a** by increasing the catalyst loading to 1–2 mol %.

Next, we investigated the Co-catalyzed isomerization of terminal allylamine derivatives over several bonds.^24^ Generally, olefin migration becomes significantly more challenging with the number of bonds between the double bond and the directing group.^25,26^ First, the isomerization of the terminal allylamine **19a** over one C–C bond was performed with 0.1 mol % catalyst loading at 80 °C and gave **19c** in a quantitative yield. To our delight, we achieved the isomerization of **20a** over two C–C bonds with a slightly higher catalyst loading of 1 mol %. However, our system was unable to achieve isomerization of **21a** over three C–C-bonds, as only traces of the desired product were obtained, although full conversion was achieved. The production of **21c** was not observed even at higher temperatures and with increased catalyst loadings.

To further demonstrate the potential applications of our developed system, we synthesized and tested several aromatic allylamine derivatives (**22a**–**31a**, Scheme 5). Amines **22a** and **23a** were successfully converted to their corresponding enamines in nearly quantitative yields. Notably, various functional groups on the phenyl ring, such as chloro (**24a**), trifluoromethyl (**25a**), or methoxy (**26a**) were tolerated. The isomerization of a more challenging substrate bearing a bromo substituent **27a** was achieved with 4 mol % catalyst loading. In comparison to the aliphatic allylamine **20a**, no isomerization occurred over two C–C bonds for the aromatic allylamine **28a**. Under the same conditions none of the initially tested noble metal catalysts were able to catalyze the isomerization of **28a** (SI section 11.4). Noteworthy, C3-substituted allylamines bearing a methyl (**29a**) or fluorobenzene (**30a**) residue were transformed into the corresponding products in yields over 70%. Furthermore, to demonstrate the synthetic utility of this transformation, our system was successfully applied to the difunctionalized allylamine **31a**. To highlight the industrial significance of our methodology, the isomerization of **1a** was successfully applied on a multigram scale, producing 311 g of citronellal **1c** (Scheme 5). Achieving a yield of 96%, we successfully demonstrated that the developed system also operates efficiently on the molar scale.

### Mechanistic Investigations

Determination of the hetero- or homogeneous character of the actual catalyst is often not evident.^27^ Thus, we performed the model reaction with the addition of a mercury drop, as a nanoparticle scavenger, which did not affect the catalytic activity. Moreover, transmission electron microscopy (TEM) analysis of the model reaction mixture with or without substrate **1a** did not reveal the formation of metal nanoparticles or clusters (see SI section 9.4 for details). The latter were also not observed by MS (SI section 9.6). The above observations indicated homogeneous catalysis.

To obtain more information, we attempted to isolate the catalytically active Co complex (see SI section 8, **Co-1**). The treatment of Co­(acac)_2_ and **L15** (ratio 1:4) in THF with two equivalents DIBAL-H resulted in the release of H_2_ and the formation of a dark brown Co solution at −80 °C (Scheme 6). The reaction mixture was filtered, layered with *n*-pentane, and the crystallization at −30 °C gave dark red platelet crystals. The molecular structure of **Co-2** was determined by SC-XRD, disclosing a homoleptic tetrahedral Co d^9^ complex with four coordinating phosphines.

**Scheme 6:**
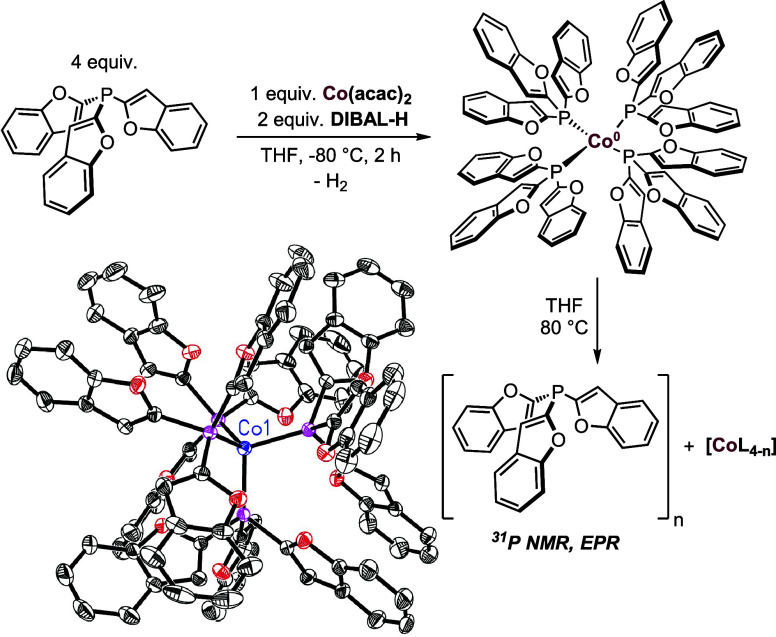
Synthesis and Transformation of **Co-2** ^a^

a Solid-state structure of **Co-2** at 30% probability ellipsoids. Hydrogen atoms have been omitted for clarity.

Interestingly, we observed the formation of **Co-2** even in the presence of only one equivalent DIBAL-H or **L15.** In subsequent crystallization experiments with substrate **1a** or 1,5-cyclooctadiene (COD), we also observed the formation of **Co-2**, however, the isolation of a Co(0) complex with coordinated **1a** or COD could not be achieved (see SI section 8 for details).

To distinguish between the activities of the isolated complex **Co-2** and the catalyst generated *in situ*, a series of control experiments were carried out (SI section 9). When 0.1 mol % catalyst loading was used, the *in situ* generated Co catalyst with 1:2 metal-to-ligand ratio gave nearly quantitative yield (98%), while the defined **Co-2** catalyst showed significantly lower activity (18% yield). However, increasing the catalyst loading to 0.3 mol % led to a significant enhancement in catalytic performance, affording **1b** in 98% yield. These combined results strongly suggest that the catalytically active Co species differs from the isolated **Co-2** complex, which may represent an off-cycle resting state.

Furthermore, based on the **Co-2** complex with either **L15** or **L1** as ligands, we carried out DFT computations employing *N*,*N*-dimethyl-3-methylbut-2-en-1-amine as a simplified model substrate (see SI section 12 for details). However, the calculated activation barriers for the [Co­(L)_4_] species were inconclusive and considerably too high to permit the isomerization to proceed under the applied reaction conditions. These observations further support the conclusion that **Co-2** does not represent the catalytically active Co species.

Moreover, EPR measurements of the molecular defined 17 valence-electron Co complex (**Co-2**) were performed to provide more insights into the reaction mechanism. *In situ* EPR analysis of **Co-2** in toluene showed no resonances at room temperature. However, at 95 K, it exhibited an EPR signal of axial symmetry at *g*_∥_ = 2.190 and *g*_⊥_ = 1.989, *A*_∥_ = 103, *A*_⊥_ = 148.5 MHz (estimated from EPR simulation, SI section 9.5). The coupling of the unpaired electrons (*S* = 1/2) with ^59^Co nuclear spins (*I* = 7/2, 100%) leads to the well-resolved eight-line hyperfine structure of the parallel component of the EPR signal with *A*_∥_ = 148.5 MHz (SI section 9.5). The poorly resolved middle hyperfine lines (second derivative EPR signal) show an additional splitting due to the hyperfine interaction with ^31^P nucleus (*I* = 1/2). Compared to low-spin Co­(II) compounds with *g*_⊥_ > *g*_∥_, **Co-2** with *g*_∥_ > *g*_⊥_ can be assigned to Co(0) in a tetragonally distorted octahedral field of the ligands.^28,29^

To further investigate the activity of **Co-2**, titration experiments were conducted in toluene, with or without substrate **1a** or THF. The addition of **1a** to **Co-2** in toluene resulted in slight shifts and decreased signal intensity in the EPR spectra (Scheme 7A), indicating a direct interaction between **1a** and **Co-2** occurs, and consequently another Co(0) complex (*g*_∥_ = *g*_∥_ = 2.188 and *g*_⊥_ = 1.989, *A*_∥_ = 105.5, *A*_⊥_ = 150.0 MHz) is present.

**Scheme 7:**
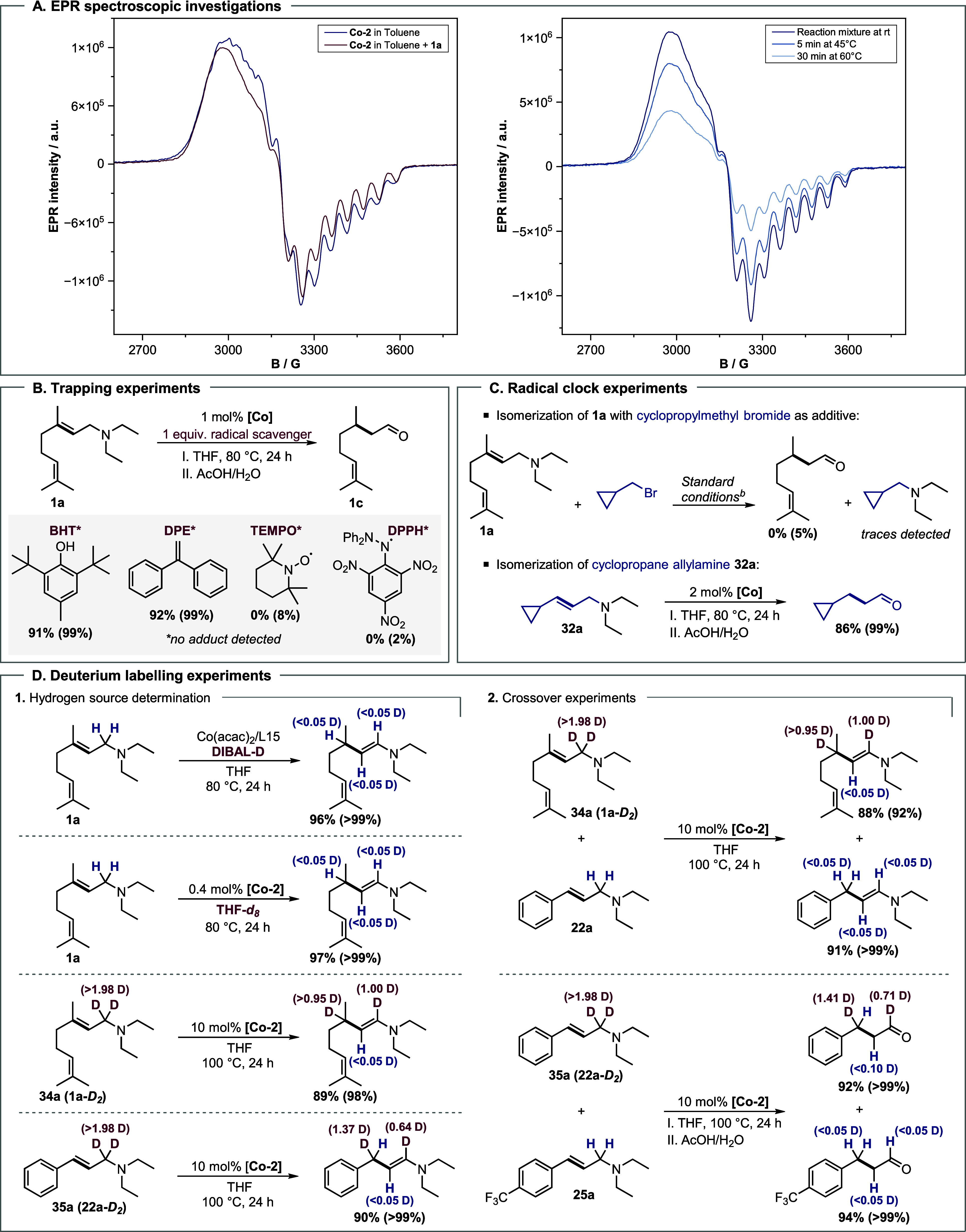
Mechanistic Investigations^a^

a (A) Recorded EPR spectra of **Co-2** in toluene, measured at 95 K. (B) Trapping experiments with radical scavengers, reaction-scale: 2.5 mmol of **1a**. (C) Radical clock experiments.
b Standard conditions: 2.5 mmol of **1a**, 1.5 mL of THF, 1 mol % Co­(acac)_2_, 2 mol % **L15**, and DIBAL-H, 80 °C, 24 h. (D) Deuterium labeling experiments.

Similarly, the addition of THF to **Co-2** resulted in a signal shift (*g*_∥_ = 2.187 and *g*_⊥_ = 1.989, *A*_∥_ = 106.2, *A*_⊥_ = 151.5 MHz), exhibiting similar features to those observed after the addition of **1a** (SI section 9.5). ^31^P NMR experiments further suggested the presence of free phosphine ligand (**L15**) when **Co-2** was dissolved in THF-*d*_8_ compared to toluene-*d*_8_, showing a peak at −67.93 ppm in the ^31^P NMR. Based on these combined results, we conclude that the phosphine undergoes dissociation in the presence of THF or **1a**, leading to the formation of the active species.

Next, to rule out a radical pathway, a series of EPR measurements were performed. The reaction mixture, consisting of **Co-2**, **1a**, and THF, was first heated to 45 °C for 5 min, followed by heating to 60 °C for 30 min (Scheme 7A). No radical intermediate was detected during the *in situ* EPR measurements. To further confirm these results, different radical scavengers were added to the model reaction with substrate **1a**. The application of TEMPO, Galvinoxyl, or DPPH led to complete suppression of the isomerization (Scheme 7B); however, no adduct was identified by GC-MS and LC-MS. We assume that the addition of these radicals led to the deactivation of the catalyst by coordination to the metal center. On the other hand, the addition of DHT or BPE did not significantly affect the model reaction. Finally, radical clock experiments (Scheme 7C) with cyclopropane-containing substrates were carried out. However, no cyclopropane ring opening could be detected for either (bromomethyl)­cyclopropane or allylamine **32a** and **33a**, which rules out the existence of a free radical intermediate during the isomerization.

Generally, the metal-catalyzed isomerization of C–C double bonds can occur via an inter- or intramolecular pathway.^7,10^ The former category encompasses radical (Scheme 8A) and metal hydride mechanisms (Scheme 8B); however, both pathways could already be excluded. In the group of intramolecular processes, one may distinguish between inner- or outer-sphere allyl mechanism (Scheme 8C), oxidative cyclization (Scheme 8D), and a cationic (Scheme 8E) or metalloradical allyl mechanism (Scheme 8F). To identify a plausible mechanistic pathway, we planned and performed a series of further experiments.

**Scheme 8:**
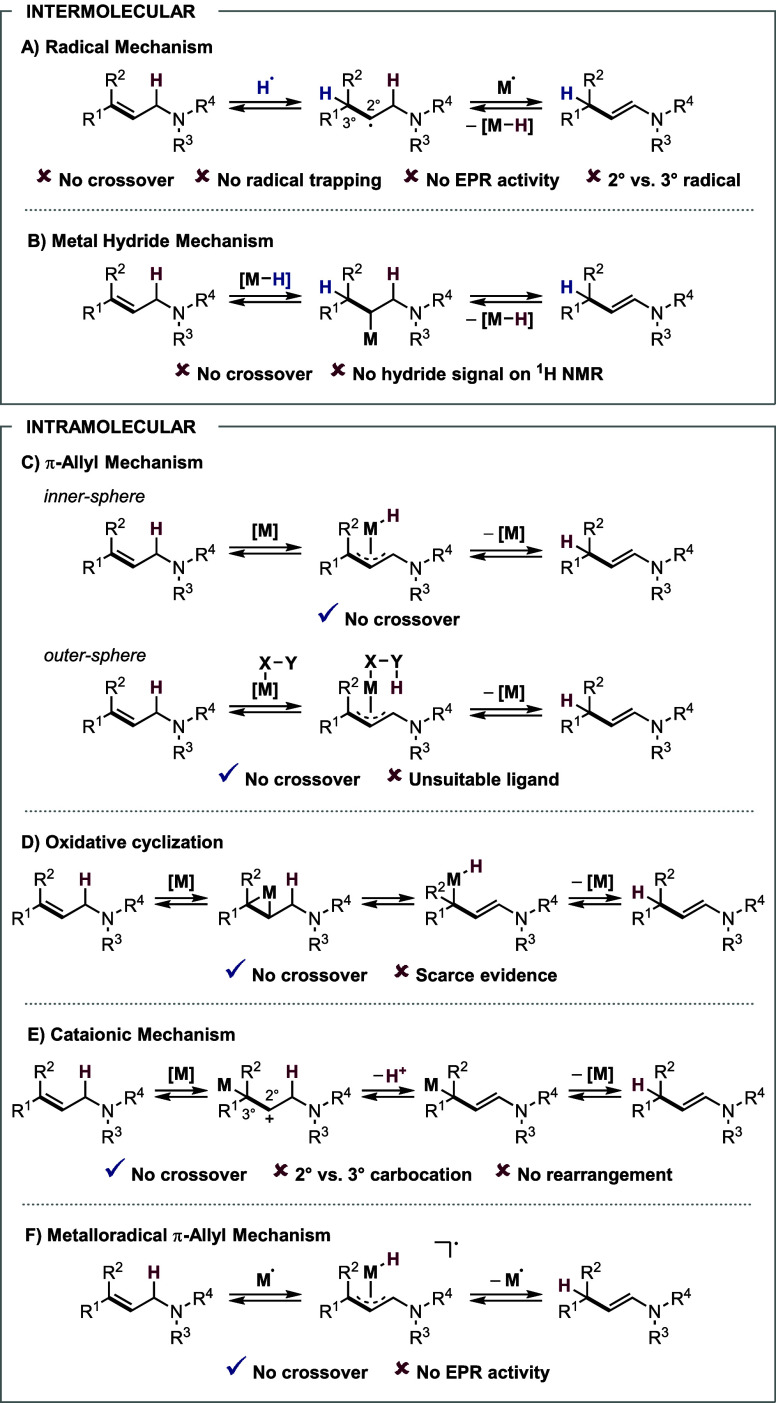
Main Pathways: Transition-Metal Catalyzed Isomerization of Allylamines

First, we performed isotope-labeling experiments (Scheme 7D). No deuterium incorporation was observed when the model reaction of **1a** was performed in THF-*d*_8_ or with DIBAL-D, excluding both compounds as hydrogen sources. On the other hand, the isomerization of the γ-deuterated substrates **1a**-***D*_2_** (**34a**) and **22a**-***D*_2_** (**35a**) led to the respective enamines **34b** and **35b**, with deuterium incorporation in the α-position, indicating the 1,3-hydrogen shift.

Finally, to distinguish between the inter- and intramolecular mechanisms, we performed H/D crossover experiments using γ-deuterated substrates **1a**-***D*_2_** or **22a**-***D*_2_** with **22a** or **25a**, respectively (Scheme 7D). No deuterium incorporation was detected in **22b** or **25b**, excluding a radical (Scheme 7A) or metal hydride mechanism (Scheme 7B). Moreover, no metal hydride was observed by ^1^H NMR.

Among the intramolecular processes mentioned above, the inner-sphere π-allyl mechanism (Scheme 8C) seems to be the most plausible pathway. First, no EPR activity should exclude the metalloradical allyl pathway (Scheme 8F).^10^ Moreover, the cationic mechanism (Scheme 8E) would involve the formation of 2° instead of 3° carbocation, and no rearrangement of 3,3-dimethylbut-1-ene, indicating carbocation formation, was observed (see SI section 9.9).^30^ Finally, oxidative cyclization (Scheme 8D) is scarcely described and substantiated in the literature.^31^

Next, we examined the model reaction mixture in time using tri­(benzofuran-2-yl) **L15** or tri­(furan-2-yl) phosphine **L1** as a ligand (Scheme 9A). In the case of **L15**, over 95% of **1a** was converted into **1b** in 4 h, and the production of unwanted isomers was negligible. In contrast, the maximal conversion of **1a** was about 80% for **L1**, and a substantial amount of byproducts was produced.

**Scheme 9:**
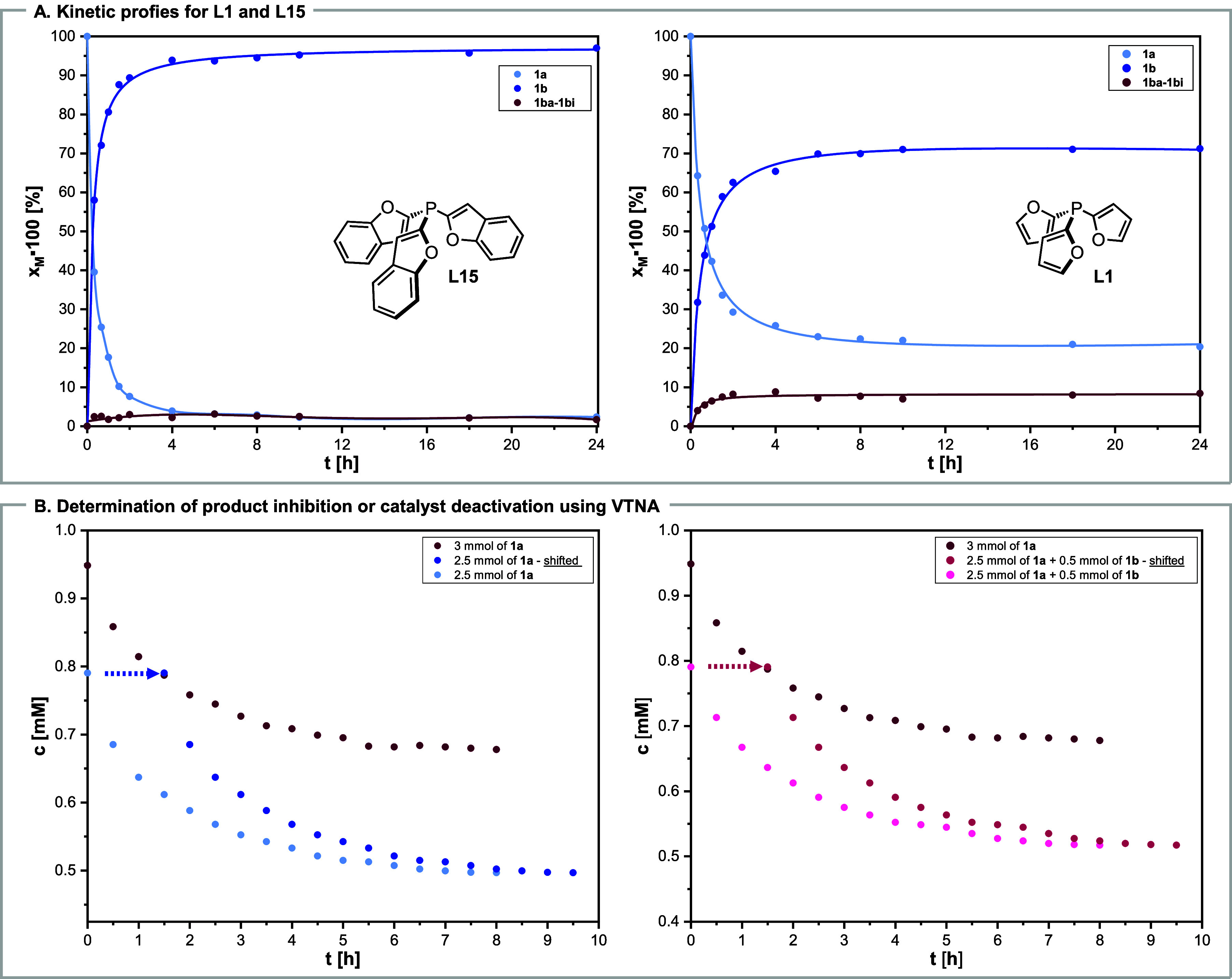
Kinetic Studies^a^

a (A) Kinetic profile experiments, reaction conditions: 2.5 mmol of **1a**, 0.2 mol % Co­(acac)_2_, 0.4 mol % **L15**, and DIBAL-H, 2-MeTHF, 100 °C. (B) VTNA plots, modified reaction conditions: 2.5 mmol of **1a**, 0.5 mol % Co­(acac)_2_, 1 mol % **L15**, and DIBAL-H, 2-MeTHF, 60 °C.

Subsequently, we employed variable-time normalization analysis (VTNA) to derive more information about the mechanism.^32^ Our initial attempts to determine substrate or catalyst order using VTNA for three different substrate or catalyst concentration profiles were unsuccessful (see SI section 9.10). Hence, we checked the possibility of the catalyst deactivation or product inhibition, which proved to occur due to the lack of curve overlay after the time shift (Scheme 9B). Distinguishing between these two required an additional experiment with the product addition, which indicated catalyst deactivation (Scheme 9B). For substantial variation in catalyst concentration, determining the substrate or catalyst order requires monitoring the catalyst concentration over time, which is impossible without knowing the actual active species.^33^

To elucidate the reason for the difference in activity and selectivity of ligands **L1** and **L15**, we hypothesized that the presence of a benzene ring in the tri­(benzofuran-2-yl)­phosphine contributes to the stability of the furyl moiety.^34^ However, ^31^P NMR analysis of the reaction mixtures did not reveal the presence of any additional phosphorus species except free ligands in both cases (SI section 9.6). Moreover, no change in the reaction with **L1** was observed after the addition of a mercury drop, excluding catalyst deactivation by nanoparticle formation.

Based on our mechanistic experiments and kinetic studies, we proposed the inner-sphere allyl mechanism (Scheme 10). The first step is the formation of the active Co species from Co­(acac)_2_ (a) or the defined **Co-2** precatalyst (b). Then, the addition of an allylamine to the Co catalyst leads to a metal substrate intermediate (c) as evidenced by EPR measurements. In the next step, C–H activation of the allylic C–H bond occurs as a rate-determining step, which generates Co–H species (d). Subsequently, the Co–H complex undergoes reinsertion of the hydride into the allylic system, generating product intermediate (e), followed by the release of the desired enamine (f). During the reaction, the catalyst is transformed into an inactive species (g).

**Scheme 10:**
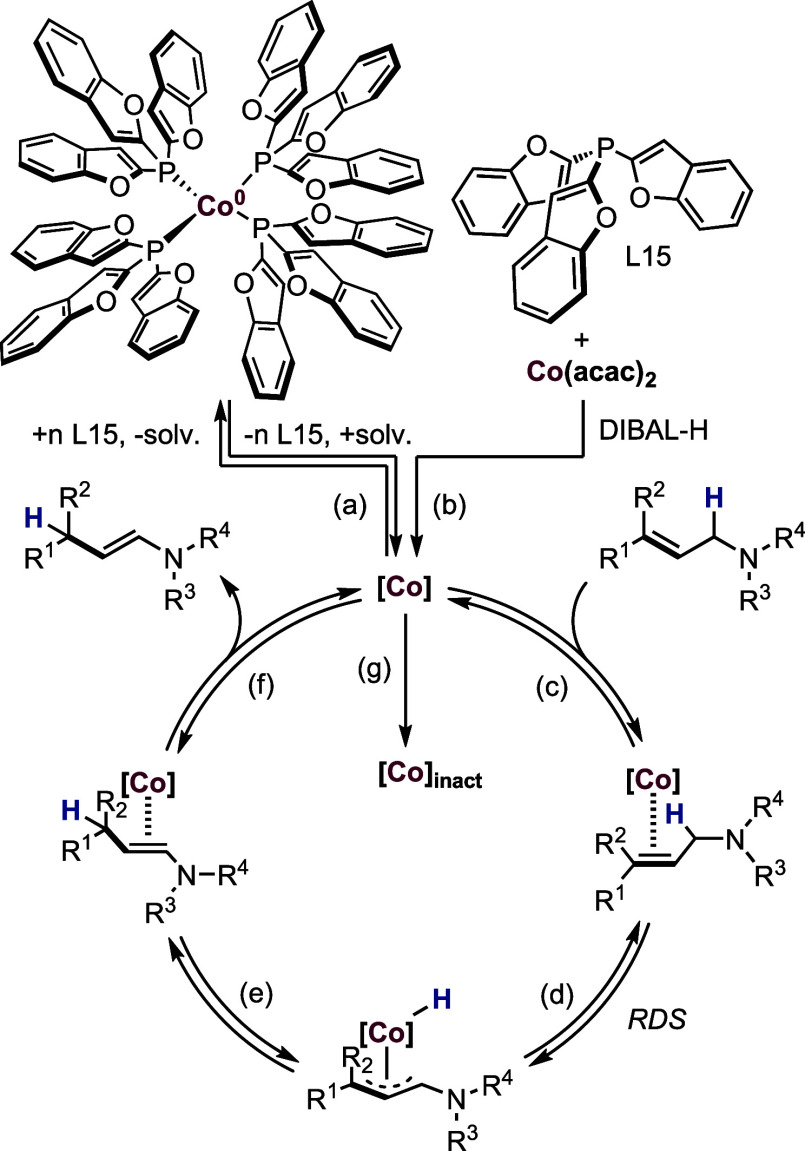
Proposed Mechanism for the Co-Catalyzed Isomerization of Allylamines

## Conclusions

In summary, a series of novel monodentate phosphine ligands bearing furan- and benzofuran-substituents were synthesized and screened in the industry-relevant Co-based isomerization of allylamines. As a result, we discovered a highly active and selective system that provides similar activity compared to currently applied Rh catalysts. Moreover, the presented method allows the isomerization of structurally diverse substrates with up to quantitative yields, including terminal and internal (aliphatic and aromatic) allylamines, bearing various functionalities (e.g., Cl, F, CF_3_). Potential industrial application of our system was substantiated by the scale-up experiment, which provided 311 g of citronellal in 96% yield. Furthermore, we observed that the catalyst activity was bound to the presence of furan-2-yl substituents of the phosphine ligand, while the selectivity increased with the number of benzofuran-2-yl groups. These trends may be helpful in the context of designing new, efficient catalysts. Moreover, we managed to isolate and characterize the Co complex, serving as a precatalyst. Finally, we employed various analytical techniques such as TEM, EPR, and NMR to understand the nature of the isomerization process. Based on the above, in combination with deuterium labeling, radical trapping experiments, and kinetic studies, we postulated a plausible mechanism encompassing a 1,3-hydrogen shift.

## Supplementary Material



## Notes

Experimental procedures, mechanistic studies, computational studies and characterization of new compounds (PDF)

## References

[1] Massad I., Marek I. (2020). Alkene Isomerization through Allylmetals
as a Strategic Tool in Stereoselective Synthesis. ACS Catal..

[2] Gauthier D., Lindhardt A. T., Olsen E. P. K., Overgaard J., Skrydstrup T. (2010). In Situ Generated
Bulky Palladium Hydride Complexes
as Catalysts for the Efficient Isomerization of Olefins. Selective
Transformation of Terminal Alkenes to 2-Alkenes. J. Am. Chem. Soc..

[3] Mantilli L., Gérard D., Torche S., Besnard C., Mazet C. (2009). Iridium-Catalyzed Asymmetric Isomerization of Primary Allylic Alcohols. Angew. Chem., Int. Ed..

[4] Scarso A., Colladon M., Sgarbossa P., Santo C., Michelin R. A., Strukul G. (2010). Highly Active and Selective
Platinum­(II)-Catalyzed
Isomerization of Allylbenzenes: Efficient Access to (*E*)-Anethole and Other Fragrances via Unusual Agostic Intermediates. Organometallics.

[5] Larsen C. R., Grotjahn D. B. (2012). Stereoselective
Alkene Isomerization
over One Position. J. Am. Chem. Soc..

[6] Yu X., Zhao H., Li P., Koh M. J. (2020). Iron-Catalyzed
Tunable and Site-Selective Olefin Transposition. J. Am. Chem. Soc..

[7] Liu X., Li B., Liu Q. (2019). Base-Metal-Catalyzed
Olefin Isomerization Reactions. Synthesis.

[8] Raje S., Garhwal S., Młodzikowska-Pieńko K., Sheikh Mohammad T., Raphaeli R., Fridman N., Shimon L. J. W., Gershoni-Poranne R., de Ruiter G. (2024). N_2_ Dissociation vs Reversible 1,2-Methyl
Migration in PC_NHC_P Cobalt­(I) Complexes in the Stereoselective
Isomerization (*E*/*Z*) of Allyl Ethers. JACS Au.

[9] Weber F., Schmidt A., Röse P., Fischer M., Burghaus O., Hilt G. (2015). Double-Bond Isomerization:
Highly Reactive Nickel Catalyst Applied in the Synthesis of the Pheromone
(9*Z*,12*Z*)-Tetradeca-9,12-dienyl Acetate. Org. Lett..

[10] Kapat A., Sperger T., Guven S., Schoenebeck F. (2019). *E*-Olefins through intramolecular radical relocation. Science.

[11] Ludwig J. R., Schindler C. S. (2017). Catalyst:
Sustainable Catalysis. Chem.

[12] Liu X., Zhang W., Wang Y., Zhang Z.-X., Jiao L., Liu Q. (2018). Cobalt-Catalyzed Regioselective
Olefin Isomerization Under Kinetic Control. J. Am. Chem. Soc..

[13] Delgado K. R., Youmans D. D., Diver S. T. (2020). Mild Isomerization
of Conjugated
Dienes Using Co-Mediated Hydrogen Atom Transfer. Org. Lett..

[14] Kim D., Pillon G., DiPrimio D. J., Holland P. L. (2021). Highly *Z*-Selective Double Bond Transposition in Simple Alkenes and Allylarenes
through a Spin-Accelerated Allyl Mechanism. J. Am. Chem. Soc..

[15] Zhao P., Huang J., Li J., Zhang K., Yang W., Zhao W. (2021). Ligand-controlled cobalt-catalyzed
remote hydroboration and alkene
isomerization of allylic siloxanes. Chem. Commun..

[16] Yamamoto A., Kitazume S., Pu L. S., Ikeda S. (1967). Study of the fixation
of nitrogen. Isolation of tris­(triphenylphosphine)­cobalt
complex co-ordinated with molecular nitrogen. Chem. Commun..

[17] Raje S., Sheikh Mohammad T., de Ruiter G. (2024). A Neutral PC_NHC_P Co­(I)-Me
Pincer Complex as a Catalyst for *N*-Allylic Isomerization
with a Broad Substrate Scope. J. Org. Chem..

[18] Nicolaou, K. C. ; Sorensen, E. J. Classics in Total Synthesis: Targets, Strategies, Methods; Wiley, 1996.

[19] Liu X., Zhang W., Wang Y., Zhang Z. X., Jiao L., Liu Q. (2018). Cobalt-Catalyzed Regioselective Olefin Isomerization Under Kinetic
Control. J. Am. Chem. Soc..

[20] Santelli-Rouvier C., Coin C., Toupet L., Santelli M. (1995). Synthesis of the tertiaryphosphine
derivatives of iron carbonyl phosphines: preparation of tetracarbonyl­[tris­(2-furyl)­phosphine]
iron(0), pentacarbonylbis­[μ-bis­(2-furyl)­phosphido]-[tris­(2-furyl)
phosphine] diiron(0), tetracarbonyl­[tris­(2-benzofuryl) phosphine]
iron(0) and pentacarbonylbis­[μ-bis­(2-benzofuryl) phosphido]-[tris­(2-benzofuryl)
phosphine]­diiron-(0). J. Organomet. Chem..

[21] Clarke M. L., Roff G. J. (2007). A highly efficient
procedure for hydroformylation and hydroamino-vinylation of methyl
acrylate. Green Chem..

[22] Chmielewska E., Miodowska N., Dziuk B., Psurski M., Kafarski P. (2023). One-Pot Phosphonylation
of Heteroaromatic Lithium Reagents: The Scope and Limitations of Its
Use for the Synthesis of Heteroaromatic Phosphonates. Molecules.

[23] Liu H., Cai C., Ding Y., Chen J., Liu B., Xia Y. (2020). Cobalt-Catalyzed *E*-Selective Isomerization of Alkenes with a Phosphine-Amido-Oxazoline
Ligand. ACS Omega.

[24] Tani K., Yamagata T., Akutagawa S., Kumobayashi H., Taketomi T., Takaya H., Miyashita A., Noyori R., Otsuka S. (1984). Metal-assisted terpenoid synthesis.
7. Highly enantioselective isomerization of prochiral allylamines
catalyzed by chiral diphosphine rhodium­(I) complexes. Preparation
of optically active enamines. J. Am. Chem. Soc..

[25] Chen X., Cheng Z., Guo J., Lu Z. (2018). Asymmetric
remote C-H borylation of internal alkenes via alkene isomerization. Nat. Commun..

[26] Lin L., Romano C., Mazet C. (2016). Palladium-Catalyzed Long-Range Deconjugative
Isomerization of Highly Substituted α,β-Unsaturated Carbonyl
Compounds. J. Am. Chem. Soc..

[27] Prima D. O., Kulikovskaya N. S., Galushko A. S., Mironenko R. M., Ananikov V. P. (2021). Transition metal
‘cocktail’-type catalysis. Curr.
Opin. Green Sustainable Chem..

[28] Saraev V. V., Shmidt F. K., Larin G. M., Lipovich V. G. (1974). Study of cobalt(0)
complexes in catalytic systems by EPR method. Bull. Acad. Sci. USSR, Div. Chem. Sci..

[29] Klein H.-F. (1980). Trimethylphosphane Complexes of Nickel,
Cobalt, and Iron—Model Compounds for Homogeneous Catalysis. Angew. Chem., Int. Ed..

[30] Sen A., Lai T. W. (1981). Catalytic
isomerization of alkenes by palladium­(II)
compounds. An alternative mechanistic view. Inorg. Chem..

[31] Kobayashi T., Yorimitsu H., Oshima K. (2009). Cobalt-Catalyzed Isomerization of 1-Alkenes to (*E*)-2-Alkenes with Dimethylphenylsilylmethylmagnesium Chloride
and Its Application to the Stereoselective Synthesis of (*E*)-Alkenylsilanes. Chem.—Asian J..

[32] Nielsen C. D. T., Burés J. (2019). Visual kinetic
analysis. Chem.
Sci..

[33] Martínez-Carrión A., Howlett M. G., Alamillo-Ferrer C., Clayton A. D., Bourne R. A., Codina A., Vidal-Ferran A., Adams R. W., Burés J. (2019). Kinetic Treatments
for Catalyst Activation and Deactivation Processes based on Variable
Time Normalization Analysis. Angew. Chem., Int.
Ed..

[34] Cao H., Rupar P. A. (2017). Recent Advances in Conjugated Furans. Chem.—Eur. J..

