# Supplementary material for: Cobalt(0)-Catalyzed Isomerization of Allylamines Promoted by Monodentate Benzofuran Phosphines
Source: J Am Chem Soc. 2025 Aug 19;147(35):31859–70. doi: 10.1021/jacs.5c09017 (PMC12412175; doi:10.1021/jacs.5c09017)
Supplement: Supplementary file 1 [file ja5c09017_si_001.pdf]

Supporting Information

Cobalt(0)-Catalyzed Isomerization of Allylamines Promoted by Monodentate Benzofuran Phosphines

Sebastian Ahrens,† Rafał Kusy,† Anke Spannenberg,† Thanh H. Vuong,† Jabor Rabeah,‡ Bernhard M. E. Russbueldt,$ Johannes Panten,$ Haijun Jiao,† Kathrin Junge,† and Matthias Beller,†

†Leibniz-Institut für Katalyse e.V.
Albert-Einstein-Straße 29a, 18059 Rostock, Germany
‡State Key Laboratory of Low Carbon Catalysis and Carbon Dioxide Utilization:
Lanzhou Institute of Chemical Physics (LICP), Chinese Academy of Sciences
Lanzhou 730000, P. R. China
$Symrise AG, Mühlenfeldstraße 1, 37603 Holzminden, Germany

Matthias Beller – matthias.beller@catalysis.de
Kathrin Junge – kathrin.junge@catalysis.de
Haijun Jiao - haijun.jiao@catalysis.de
Johannes Panten – johannes.panten@symrise.com

Contents

1 General Information 4

2 Literature Known Complexes – Isomerization of 1a 7

3 Initial Screening of Phosphine Ligands 9

3.1 Experimental procedures 9

4 Synthesis of Furan- and Benzofuran-Phosphines 12

4.1 Bis(2-furyl)-phosphine chloride 12

4.2 Furan-2-yldiphenylphosphine (**L5**) 13

4.3 Bis(furan-2-yl)(phenyl)phosphine (**L6**) 14

4.4 Bis(furan-2-yl)(methyl)phosphine (**L7**) 15

4.5 Bis(furan-2-yl)(isobutyl)phosphine (**L8**) 16

4.6 Ethoxydi(furan-2-yl)phosphine (**L9**) 17

4.7 *N,N*-diethyl-1,1-di(furan-2-yl)phosphanamine (**L10**) 18

4.8 Tris(5-methylfuran-2-yl)phosphine (**L11**) 19

4.9 Tris(4,5-dimethylfuran-2-yl)phosphine (**L12**) 20

4.10 Benzofuran-2-yldi(furan-2-yl)phosphine (**L13**) 22

4.11 Di(benzofuran-2-yl)(furan-2-yl)phosphine (**L14**) 24

4.12 Tri(benzofuran-2-yl)phosphine (**L15**) 26

4.13 Tris(benzo[1,2-b:4,5-b']difuran-2-yl)phosphine (**L16**) 27

4.14 Tri(benzofuran-2-yl)phosphine oxide (**L17**) 28

4.15 Tris(3-methylbenzofuran-2-yl)phosphine (**L18**) 29

4.16 Di(benzofuran-2-yl)chlorophosphine 31

4.17 Di(benzofuran-2-yl)(methyl)phosphine (**L19**) 32

4.18 Di(benzofuran-2-yl)(*tert*-butyl)phosphine (**L20**) 33

4.19 Di(benzofuran-2-yl)(1,3-dioxolan-2-yl)phosphine (**L21**) 34

4.20 Di(benzofuran-2-yl)(methyl)phosphine (**L22**) 36

4.21 Di(benzofuran-2-yl)(2-methoxyphenyl)phosphine (**L23**) 37

4.22 Di(benzofuran-2-yl)(dibenzo[*b,d*]furan-4-yl)phosphine (**L24**) 39

4.23 Tri(benzofuran-3-yl)phosphine (**L25**) 41

4.24 Tri(benzofuran-7-yl)phosphine (**L26**) 42

4.25 Tri(benzofuran-5-yl)phosphine (**L27**) 43

5 Ligand Screening: Furan- and Benzofuran-Phosphines 44

6 Optimization of Reaction Conditions 49

7 Identification of Side-Products - Isomerization of 1a 53

8 Single Crystal X-ray Diffraction 83

9 Mechanistic Investigations 90
9.1 Comparison: isolated Co complex and *in situ* Co catalyst 90
9.2 Control Experiments 91
9.3 Mercury Drop Test 93
9.4 TEM Measurements 94
9.5 EPR Measurements 95
9.6 NMR Measurements 100
9.7 Radical Trap Experiments 106
9.7.1 Radical Scavenger 106
9.7.2 Radical clock experiments 112
9.8 Deuterium Labelling Experiments 122
9.8.1 Synthesis of compound 1a-D_2 (34a) 122
9.8.2 Isomerization of 1a using DIBAL-D as reductant 129
9.8.3 Isomerization of 1a in THF-d_8 130
9.8.4 Isomerization of 1a-D_2 (34a) 131
9.8.5 Synthesis of compound 22a-D_2 (35a) 134
9.8.6 Isomerization of 22a-D_2 (35a) 139
9.8.7 Crossover-Experiment 142
9.9 Isomerization of 3,3-dimethylbutene 150
9.10 Kinetic Studies 151
10 Substrate Synthesis - Allylamine Derivatives 160
10.1 Geranylamine Derivatives 160
10.2 Aliphatic and Aromatic Substrates 169
11 Isomerization of Allylamine Derivatives 189
11.1 General Procedure: Isomerization of Allylamine Derivatives 189
11.2 Isolated Products 190
11.3 Up-scale Experiment 204
11.4 Failed Substrates 206
12 Computational Methods and Models 208
13 NMR Spectra 301
13.1 Furan- and Benzofuran-Phosphines 301
13.2 Allylamines Derivatives 351
13.3 Isolated Enamines and Aldehydes 386
14 IR Spectra 410
15 HRMS 418
16 References 426

1 General Information

Procedure and chemicals

All commercial reagents were obtained from the following chemical companies: Sigma-Aldrich, Fisher Scientific, BLDpharm, TCI, ABCR and Strem. Unless otherwise noted, the commercial reagents were used without purification. The reactions with air- and moisture-sensitive reagents were carried out under argon atmosphere using standard Schlenk technique or in a M. Braun glovebox. Anhydrous and oxygen-free solvents (THF, DCM, diethyl ether, toluene, benzene, *n*-pentane, *n*-hexane, and *n*-heptane) were received from an Innovative Technology PS-MD-6 solvent purification system or they were prepared by freeze-pump-thaw technique. All anhydrous solvent were stored over 3 Å molecular sieves under argon atmosphere.

Thin-Layer Chromatography

Analytical thin-layer chromatography (TLC) was performed on Machery-Nagel pre-coated ALUGRAM Xtra SIL G/UV254 TLC sheets. Visualization was achieved by irradiation with UV-light, or by staining with potassium permanganate, *p*-anisaldehyde or phosphomolybdic acid. Flash Column chromatography was performed with a Combi Flash Rf + from Teledyne ISCO using HPLC grade solvents.

Nuclear Magnetic Resonance Spectroscopy

NMR spectra were recorded on Bruker Avance 300 (300 MHz) or 400 (400 MHz) NMR spectrometer. The chemical shifts (δ) are reported in parts per million (ppm) and coupling constants (J) in hertz (Hz). All chemical shifts (δ) are given relative to solvent: references for THF-d_8 were 1.72 and 3.58 ppm (^{1}H), 67.21, and 25.31 ppm (^{13}C), for $CDCl_3$ 7.26 ppm (^{1}H) and 77.16 ppm (^{13}C) and for CD_2Cl_2 5.32 ppm (^{1}H) and 53.84 ppm (^{13}C). Multiplets of NMR were assigned as s (singlet), br s (broad singlet), d (doublet), t (triplet), q (quartet), ps-qui (pseudo-quintet), ps-h (pseudo-hextet), hept (heptet), ps-n (pseudo-nonet), dd (doublet of doublet), dt (doublet of triplet), dq (doublet of quartet), dh (doublet of heptet), ddd (doublet of doublet of doublet), td (triplet of doublet), and m (multiplet).

Electron Paramagnetic Resonance Spectroscopy

EPR measurements were performed on a Bruker EMX CW-micro X-band spectrometer with a microwave power $\approx$ 6.9 mW, a modulation frequency of 100 kHz and modulation amplitude of 1 G. The EPR spectrometer is equipped with a variable temperature control unit including a liquid N_2 cryostat and a temperature controller for recording the EPR spectra at low temperature down to 95K. g values were calculated using the equation

$h\nu = g\beta B_0$ with β, B_0 and ν being the Bohr magneton, resonance field and frequency, respectively. The DPPH standard (g = 2.0036 ± 0.0004) was used as reference substance for calibration of the g values.

X-Ray Crystallographic Analysis

Diffraction data were collected on a Bruker Kappa APEX II Duo and an IPDS II diffractometer, respectively. The structures were solved by intrinsic phasing (SHELXT: Sheldrick, G. M. Acta Cryst. 2015, A71, 3.) and refined by full-matrix least-squares procedures on F2 (SHELXL-2019: Sheldrick, G. M. Acta Cryst. 2015, C71, 3.). XP (Bruker AXS) was used for graphical representations. Contributions of disordered solvent in complex AX2638 were removed from the diffraction data using the SQUEEZE procedure in PLATON (Spek, A. L. Acta Cryst. 2015, C71, 9). CCDC 2379866 - 2379870 contain the supplementary crystallographic data for this paper. These data are provided free of charge by the joint Cambridge Crystallographic Data Centre and Fachinformationszentrum Karlsruhe Access Structures service www.ccdc.cam.ac.uk/structures.

Gas Chromatography

GC measurements were performed on an Agilent HP 6890 with a HP5 column. GC conversion and yields were determined using *n*-hexadecane as internal standard. GC-MS spectra were recorded on a GC-MS Agilent 5973 Network.

High-Resolution Mass Spectrometry

HRMS measurements were performed using a Waters Xevo G2XS TOF MS.

IR Spectroscopy

ATR-IR spectra were recorded on a Nicolet iS5 FT-IR equipped with a PIKE Technologies GladiATR (Thermo Fisher).

Important Notes:

Given the high sensitivity of the catalytic reaction to even trace amounts of water, all tested allylamine derivatives were dried over 3 Å molecular sieves for at least 3 days. Furthermore, to achieve consistent and optimal results, the water content of the used anhydrous solvents was regularly determined by Karl Fischer titration. Initial test reactions, especially with low catalyst loading have demonstrated that trace amounts of water can lead to reproducibility issues or even catalyst deactivation. However, prolonged storage of allylamines over molecular sieves resulted in partial decomposition of the substrates, indicated by a color change from light yellow/colorless to orange.

2 Literature Known Complexes – Isomerization of 1a

The initially tested cobalt complexes **C4-C6**, which are shown in **Figure S1**, have been synthesized according to the literature.[1, 2] Additionally, the Rh-BINAP-catalyst **C1** as well as the commercially available rhodium catalyst **C2** and Shvo catalyst **C3** have been tested for the isomerization of allylamine **1a**. The resulting data from these catalysts for the isomerization of **1a** are summarized in **Table S1**.

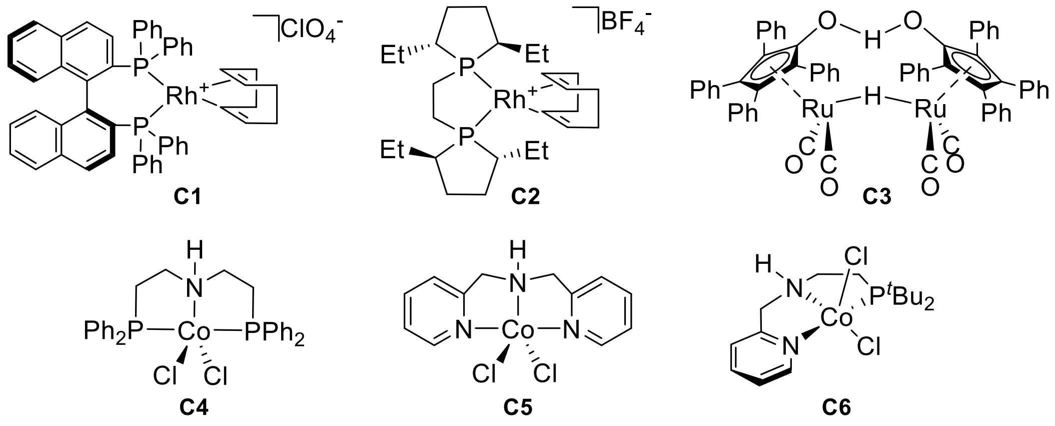

Figure S1: Tested noble- and non-noble metal complexes for the isomerization of **1a**.

General procedure:

Under argon atmosphere, an oven-dried 25 mL Schlenk tube was charged with the respective catalyst (**Figure S1**) and a stirring bar. Then, 1.5 mL of anhydrous THF and **1a** (0.523 g, 2.5 mmol) were added. The sealed Schlenk tube was heated for 24 h at 80 °C. Then, the reaction mixture was cooled down to room temperature. The conversion of the starting material was determined with GC analysis using *n*-hexadecane as internal standard. After, the solvent was removed *in vacuo*, a vacuum distillation was performed from the crude mixture. The distilled enamine was then hydrolyzed with 3 mL of a 5% acetic acid solution. After 30 min of stirring, citronellal (**1c**) was extracted with *n*-pentane followed by one washing step with a sodium carbonate solution and distilled water. The organic solution was dried with sodium sulfate and the solvent was removed *in vacuo*, yielding a colorless oil as the product. The yield was determined with GC analysis using *n*-hexadecane as internal standard.

Table S1: Comparison of noble metal- and Co-catalysts for the isomerization of **1a**.

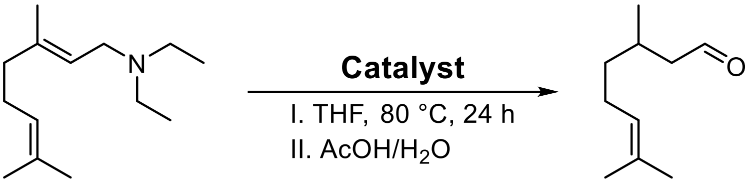

1a **1c**

Entry	T [°C]	t [h]	Solvent	**Catalyst**	Catalyst [mol%]	**Conversion[a] [mol%]**	**Yield[a] [mol%]**
1	80	24	THF	**C1**	0.02	**97**	**97**
2	80	24	THF	**C2**	0.1	**80**	**75**
3	80	24	THF	**C3**	0.1	**89**	**80**
4[b]	80	24	THF	**C4**	2	**-**	**-**
5[b]	80	24	THF	**C5**	2	**2**	**-**
6[b]	80	24	THF	**C6**	2	**13**	**-**

[a] General conditions: Substrate (2.5 mmol), THF (1.5 mL), catalyst, 80 °C, 24 h. The isolated yields and the substrate conversions were determined by GC using *n*-hexadecane as internal standard. [b] 4 mol% $NaBHEt_3$ was added.

3 Initial Screening of Phosphine Ligands

3.1 Experimental procedures

Under argon atmosphere an oven-dried 25 mL Schlenk tube was charged with cobalt(II) acetate (8.9 mg, 0.05 mmol, 1 equiv.), a selected phosphine (0.15 mmol, 3 equiv.) and a stirring bar.[1] Then, the Schlenk tube was sealed with a rubber septum and 2 mL of anhydrous THF were added. At room temperature, a DIBAL-THF- solution (0.5 M, 0.3 mL, 0.15 mmol, 3 equiv.) was dropwise injected to the solution under stirring. Then, **1a** (0.523 g, 2.5 mmol) was added and the Schlenk tube was sealed. The reaction mixture was heated for 24 h at 80 °C. Then, the reaction mixture was cooled down to room temperature. The conversion of the starting material was determined with GC analysis using *n*-hexadecane as internal standard. After, the solvent was removed *in vacuo*, a vacuum distillation of the obtained brown oil was performed. The distilled enamine was then hydrolyzed with 3 mL of a 5% acetic acid solution. After 30 min of stirring, the citronellal was extracted with *n*-pentane followed by two washing steps with a sodium carbonate solution and distilled water. The organic solution was dried with sodium sulfate and the solvent was removed *in vacuo*, yielding citronellal (**1c**) as colorless oil. The yield was determined with GC analysis using *n*-hexadecane as internal standard.

[1]Bidentate phosphine ligands were used with 0.05 mmol (1 equiv.).

Table S2: Screening of monodentate phosphines - isomerization of **1a**.

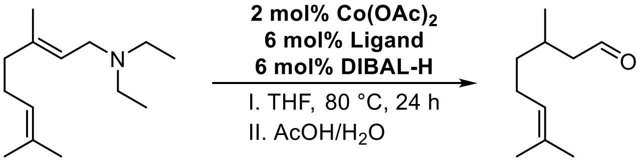

1a → **1c**

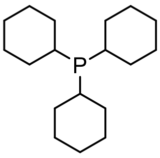

Yield = 2%

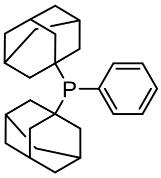

Yield = 14%

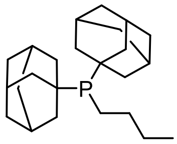

Yield = 15%

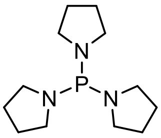

Yield = 9%

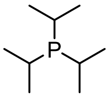

Yield = 3%

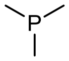

Yield = 0%

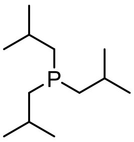

Yield = 2%

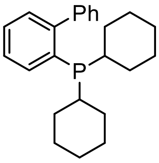

Yield = 7%

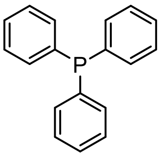

Yield = 52%

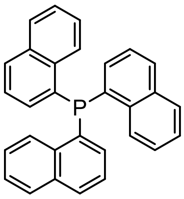

Yield = 21%

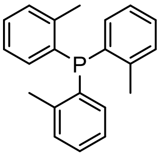

Yield = 31%

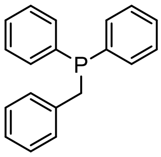

Yield = 20%

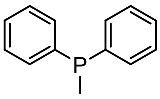

Yield = 45%

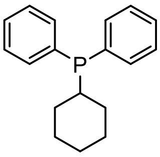

Yield = 42%

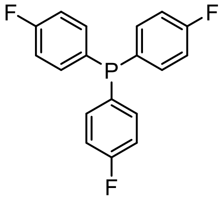

Yield = 35%

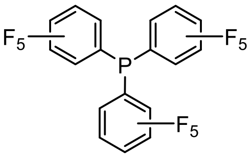

Yield = 19%

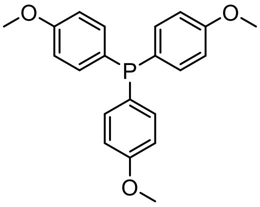

Yield = 9%

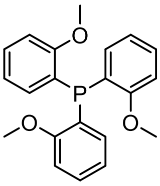

Yield = 8%

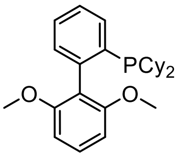

Yield = 15%

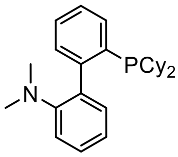

Yield = 0%

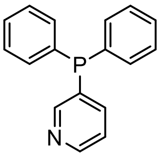

Yield = 2%

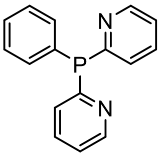

Yield = 0%

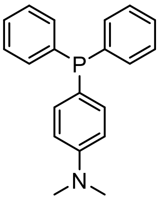

Yield = 0%

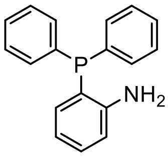

Yield = 2%

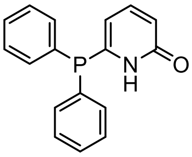

Yield = 2%

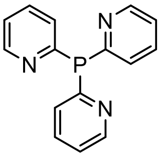

Yield = 0%

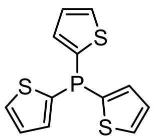

Yield = 2%

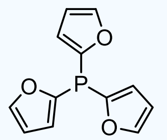

Yield = 81%

Table S3: Screening of bidentate phosphines - isomerization of **1a**.

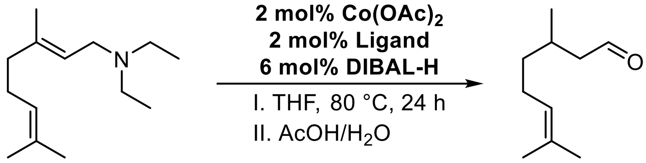

1a **1c**

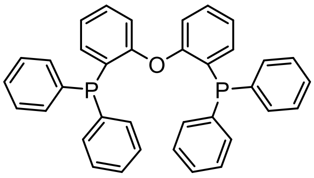

Yield = 27%

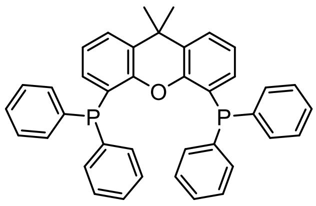

Yield = 30%

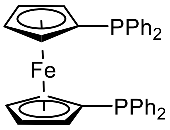

Yield = 21%

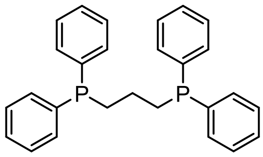

Yield = 17%

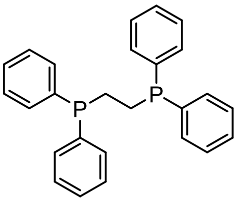

Yield = 15%

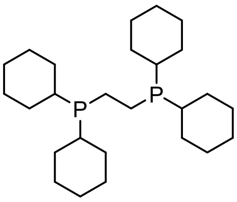

Yield = 8%

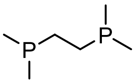

Yield = 0%

4 Synthesis of Furan- and Benzofuran-Phosphines

4.1 Bis(2-furyl)-phosphine chloride

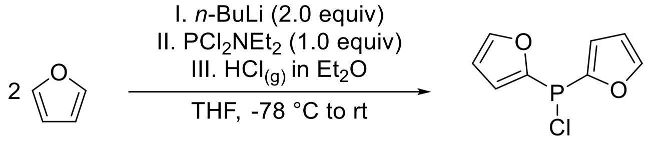

Under argon atmosphere, a 100 mL Schlenk flask was charged with degassed furan (6.81 g, 100 mmol, 2 equiv.) and 60 mL of anhydrous diethyl ether. Then, the solution was cooled down to -80 °C and 40 mL *n*-BuLi (2.5 M, 100 mmol, 2 equiv.) were dropwise added, which resulted in a color change from colorless to yellow. The solution was allowed to warm up to room temperature and stir for 2 h. Afterwards, the yellow solution was cooled again to -20 °C and dichloro(diethylamino)phosphine (8.70 g, 50 mmol, 1 equiv.) was added slowly via syringe. The resulting solution stirred overnight. Next, ethereal HCl (2.0 M, 63 mL, 126 mmol, 2.1 equiv.) was transferred into a Schlenk dropping funnel and was slowly added to the reaction mixture. Subsequently, the formed suspension was stirred for 2 h and the mixture was filtered with a Schlenk frit packed with Celite®, which yielded a light-yellow solution. After, removing the solvent *in vacuo* a yellow viscous oil was received. Finally, a vacuum distillation of the crude oil was performed and the desired product was obtained as a colorless oil.[4]

Chemical Formula: $C_8H_6ClO_2P$

Molecular Weight [g/mol]: 200.5578

Appearance: colorless oil

Isolated Yield: 75% (7.54 g, 37.6 mmol)

^{1}H NMR (400 MHz, THF-*d₈*): δ = 7.89 (dd, *J* = 1.7, 0.8, 2H), 7.08 (ddd, *J* = 3.4, 1.5, 0.7, 2H), 6.54 (dt, *J* = 3.5, 1.8, 2H).

^{13}C NMR (101 MHz, THF-d_8): δ = 151.32 (d, *J*=31.3), 150.03 (d, *J*=4.0), 123.59 (d, *J*=29.5), 111.78 (d, *J*=5.9).

^{31}P NMR (162 MHz, THF-d_8): δ = -70.02.

4.2 Furan-2-yldiphenylphosphine (L5)

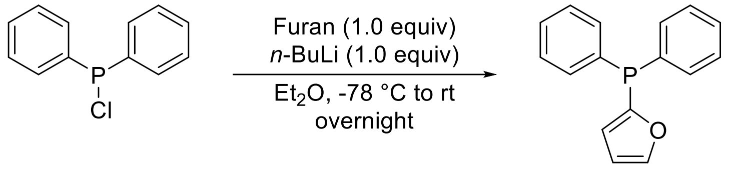

Under argon atmosphere, chlorodiphenylphosphine (1.50 g, 6.80 mmol, 1 equiv.) was charged in a flame dried Schlenk flask. Then, 15 mL of anhydrous diethyl ether were added. In another Schlenk flask, degassed furan (0.46 g, 6.80 mmol, 1 equiv.) was dissolved in 10 mL anhydrous diethyl ether. The solution was cooled to -78 °C and *n*-BuLi (2.5 M, 2.72 mL, 6.80 mmol, 1 equiv.) was added dropwise to the solution with a syringe. Next, the reaction mixture was allowed to warm up to room temperature and stir for 2 h. The reaction mixture was transferred into a Schlenk dropping funnel and the solution was slowly added to the chlorodiphenylphosphine solution at -78 °C. The solution was allowed to warm up to room temperature and stir over night. The resulting mixture was filtrated through a syringe filter to remove the lithium chloride. Then, the solvent was removed *in vacuo* from the collected filtrate, which yielded a yellow oil. Finally, a colorless oil was obtained by high vacuum distillation, which crystallized shortly after the distillation.[6]

Chemical Formula: $C_{16}H_{13}OP$

Molecular Weight [g/mol]: 252.2528

Appearance: white solid

Isolated Yield: 82% (1.41 g, 5.58 mmol)

^{1}H NMR (400 MHz, THF-*d$_8$*): δ = 7.74 (dd, *J* = 1.9, 0.8, 1H), 7.38 - 7.32 (m, 4H), 7.32 - 7.27 (m, 6H), 6.69 - 6.63 (m, 1H), 6.44 (dt, *J* = 3.2, 1.6, 1H).

^{13}C NMR (101 MHz, THF-*d$_8$*): δ = 153.25 (d, *J*=18.1), 148.51 (d, *J*=1.7), 137.43 (d, *J*=6.3), 133.90 (d, *J*=19.8), 129.32, 128.97 (d, *J*=7.0), 122.39 (d, *J*=24.7), 111.16 (d, *J*=5.5).

^{31}P NMR (122 MHz, THF-*d$_8$*): δ = -27.12.

GC-MS: *m/z* (%): 253.08 ([M+H]$^+$, 18), 252.08 (M$^+$, 100), 251.08 ([M-H]$^+$, 19), 205.10 (11), 204.10 (9), 203.09 (9), 183.02 (17), 175.02 (41), 147.02 (9), 146.02 (10), 145.01 (16), 144.03 (10), 128.04 (10), 115.04 (16), 107.99 (12), 106.99 (13), 105.01 (7).

4.3 Bis(furan-2-yl)(phenyl)phosphine (L6)

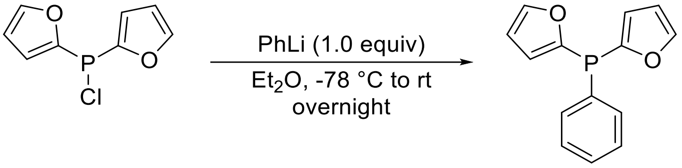

Under argon atmosphere, bis(2-furyl)-phosphine chloride (1.02 g, 5.08 mmol, 1 equiv.) was weighed in a flame dried Schlenk flask. Then, 15 mL of anhydrous diethyl ether were added, and the solution was cooled to -78 °C. Phenyllithium (1.9 M, 2.67 mL, 5.08 mmol, 1 equiv.) was slowly transferred to the stirring solution and the temperature was kept at -78 °C for 1h. The reaction mixture was allowed to warm up to room temperature and stir over night. After, the lithium chloride was removed from the reaction mixture by anaerobic filtration with a syringe filter, the solvent was removed *in vacuo*. Finally, the received yellow oil was purified by vacuum distillation, which yielded the target product as a colorless oil.[5]

Chemical Formula: $C_{14}H_{11}O_2P$

Molecular Weight [g/mol]: 242.2138

Appearance: colorless oil

Isolated Yield: 85% (1.05 g, 4.34 mmol)

^{1}H NMR (400 MHz, THF-*d$_8$*): δ = 7.73 (dt, *J*=1.8, 0.6, 2H), 7.42 - 7.34 (m, 2H), 7.31 - 7.25 (m, 3H), 6.72 (ddd, *J*=3.3, 1.7, 0.8, 2H), 6.44 (dt, *J*=3.3, 1.6, 2H).

^{13}C NMR (101 MHz, THF-*d$_8$*): δ = 151.59 (d, *J*=9.0), 148.37 (d, *J*=2.7), 136.04 (d, *J*=2.2), 133.15 (d, *J*=20.1), 129.35, 128.91 (d, *J*=7.2), 121.82 (d, *J*=23.3), 111.22 (d, *J*=5.8).

^{31}P NMR (162 MHz, THF-*d$_8$*): δ = -51.18.

GC-MS: *m/z* (%): 243.08 ($[M+H]^+$, 16), 242.08 (M^+, 100), 241.08 ($[M-H]^+$, 7), 195.08 (5), 183.05 (7), 179.09 (7), 178.09 (11), 177.08 (6), 167.09 (14), 166.08 (11), 162.03 (17), 152.06 (14), 145.03 (11), 144.05 (14), 134.03 (7), 128.06 (9), 115.06 (19), 109.02 (12), 108.01 (8), 107.00 (14), 105.02 (8), 97.99 (9), 77.05 (11), 70.00 (9), 51.04 (8).

4.4 Bis(furan-2-yl)(methyl)phosphine (L7)

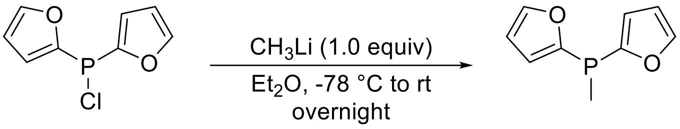

Bis(furan-2-yl)(methyl)phosphine was synthesized by the same method as described for bis(furan-2-yl)(phenyl)phosphine (**L6**). The desired product was received by the reaction of methyllithium (1.6 M, 1.71 mL, 2.74 mmol, 1 equiv.) and bis(2-furyl)-phosphine chloride (0.55 g, 2.74 mmol, 1 equiv.).[7]

Chemical Formula: $C_9H_9O_2P$

Molecular Weight [g/mol]: 180.1428

Appearance: colorless oil

Isolated Yield: 69% (0.34 g, 1.89 mmol)

^{1}H NMR (400 MHz, THF-*d₈*): δ = 7.65 (dd, *J* = 1.7, 0.7, 2H), 6.73 - 6.58 (m, 2H), 6.45 - 6.27 (m, 2H), 1.59 (d, *J* = 4.2, 3H).

^{13}C NMR (101 MHz, THF-d_8): δ = 153.66 (d, *J*=16.2), 147.51 (d, *J*=1.5), 119.22 (d, *J*=23.9), 111.00 (d, *J*=5.4), 9.35 (d, *J*=3.7).

^{31}P NMR (162 MHz, THF-d_8): δ = -70.02.

GC-MS: *m/z* (%): 181.05 ($[M+H]^+$, 7), 180.04 (M^+, 70), 166.02 (9), 165.02 (100), 137.02 (14), 136.01 (6), 118.04 (7), 109.02 (45), 107.01 (5), 83.01 (13), 81.02 (5), 70.01 (6), 69 (9), 68 (4), 65.04 (5), 57.01 (9).

4.5 Bis(furan-2-yl)(isobutyl)phosphine (L8)

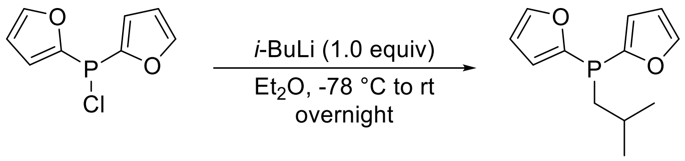

Bis(furan-2-yl)(isobutyl)phosphine was synthesized by the same method as described for bis(furan-2-yl)(phenyl)phosphine (**L6**). The desired product was received by the reaction of isobutyllithium (1.7 M, 1.61 mL, 2.74 mmol, 1 equiv.) and bis(2-furyl)-phosphine chloride (0.55 g, 2.74 mmol, 1 equiv.).

Chemical Formula: $C_{12}H_{15}O_2P$

Molecular Weight [g/mol]: 222.2238

Appearance: colorless oil

Isolated Yield: 81% (0.492 g, 2.21 mmol)

^{1}H NMR (400 MHz, THF-*d8*): δ = 7.66 (dd, *J* = 1.9, 0.8, 2H), 6.85 - 6.54 (m, 2H), 6.53 - 6.13 (m, 2H), 2.09 (d, *J* = 7.0, 2H), 1.61 (ddp, *J* = 13.5, 9.5, 6.7, 1H), 0.97 (d, *J* = 6.7, 6H).

^{13}C NMR (101 MHz, THF-*d8*): δ = 153.01 (d, *J*=17.0), 147.52 (d, *J*=1.8), 120.13 (d, *J*=24.2), 111.00 (d, *J*=5.5), 35.89 (d, *J*=2.6), 26.84 (d, *J*=14.4), 23.93 (d, *J*=9.9).

^{31}P NMR (162 MHz, THF-*d8*): δ = -64.88.

GC-MS: *m/z* (%): 223.12 ([M+H]$^+$, 9), 222.12 (M$^+$, 64), 180.05 (8), 166.03 (16), 165.04 (100), 137.03 (16), 119.05 (5), 118.05 (5), 109.03 (27), 107.02 (5), 99.01 (9), 98 (8), 91.06 (5), 83.02 (9), 81.04 (23), 71.02 (6), 70.01 (7), 69.01 (5), 65.05 (5), 57.05 (7).

HRMS (ESI): *m/z* calcd. for $C_{12}H_{15}O_2P$: 223.0888 [M+H]$^+$, found: 223.0884.

IR (ATF): **ν** [cm^{-1}] = 1461 (w), 1367 (w), 1264 (s), 1212 (w), 1150 (w), 1118 (w), 1005 (s), 901 (m), 732 (s), 703 (s), 595 (m).

4.6 Ethoxydi(furan-2-yl)phosphine (L9)

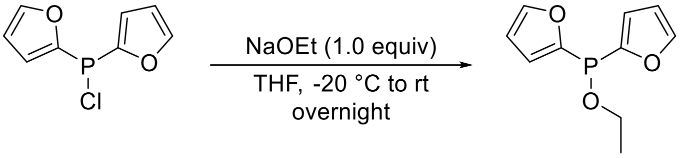

Under argon atmosphere, a flame dried Schlenk flask was charged with sodium ethoxide (0.39 g, 5.76 mmol, 1.05 equiv.) and 10 mL of anhydrous THF. In another Schlenk flask, bis(2-furyl)-phosphine chloride (1.10 g, 5.48 mmol, 1 equiv.) was dissolved in 10 mL anhydrous THF. At -20 °C, the sodium ethoxide solution was added dropwise to the bis(2-furyl)-phosphine chloride solution and the resulting reaction mixture was then allowed to stir at same temperature for 1 h. After the solution was warmed up to room temperature, the solvent was removed *in vacuo*. Finally, the obtained oil was purified by vacuum distillation, which yielded the target product (**L9**).

Chemical Formula: $C_{10}H_{11}O_3P$

Molecular Weight [g/mol]: 210.1688

Appearance: colorless oil

Isolated Yield: 65% (0.75 g, 3.56 mmol)

^{1}H NMR (400 MHz, THF-*d_8*): δ = 7.78 (dd, J=1.7, 0.7, 2H), 6.92 - 6.78 (m, 2H), 6.47 (dt, J=3.2, 1.6, 2H), 3.79 - 3.72 (m, 2H), 1.08 - 1.04 (m, 3H).

^{13}C NMR (101 MHz, THF-*d_8*): δ = 155.44 (d, J=23.4), 148.20 (d, J=3.4), 121.75 (d, J=23.3), 111.01 (d, J=4.9), 65.31, 16.84 (d, J=5.0).

^{31}P NMR (162 MHz, THF-*d_8*): δ = 55.63.

GC-MS: *m/z* (%): 211.07 ([M+H]$^+$, 9), 210.07 (M$^+$, 83), 182.03 (46), 181.02 (100), 166.02 (75), 165.02 (29), 134.04 (51), 119.05 (12), 118.04 (48), 115.00 (20), 109.02 (16), 107.04 (17), 106.04 (18), 105.04 (13), 99.00 (32), 95.02 (16), 94.04 (11), 92.03 (10), 89.04 (14), 79.06 (10), 78.05 (19), 70.01 (12), 69.01 (11), 68.02 (10), 51.04 (11).

HRMS (ESI): *m/z* calcd. for $C_{10}H_{11}O_3P$: 211.0519 [M+H]$^+$, found: 211.0522.

IR (ATF): **ν** [cm^{-1}] = 1548 (w), 1453 (m), 1369 (m), 1214 (m), 1153 (m), 1122 (m), 1029 (s), 1005 (s), 923 (m), 899 (m), 744 (br), 593 (m).

4.7 *N,N*-diethyl-1,1-di(furan-2-yl)phosphanamine (L10)

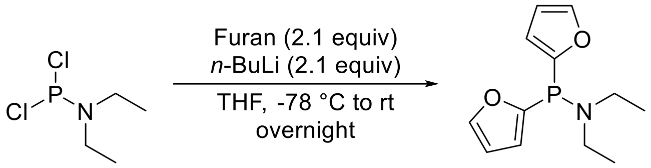

First, a flame dried Schlenk flask was charged with degassed furan (1.47 g, 21.60 mmol, 2.1 equiv.) and then 15 mL of anhydrous diethyl ether were added. The solution was cooled to -78 °C, followed by the slowly addition of *n*-BuLi (2.5 M, 8.66 mL, 21.65 mmol, 2.1 equiv.) with a syringe. The reaction mixture was allowed to warm up to room temperature and stir for 2 h. Dichloro(diethylamino)phosphine (1.80 g, 10.31 mmol, 1 equiv.) was weighed in a separate Schlenk flask and was dissolved in 15 mL anhydrous diethyl ether. Next, the lithiated furan solution was transferred into a Schlenk dropping funnel and the solution was dropwise added to the dichloro(diethylamino)phosphine solution at -78 °C. The reaction mixture was allowed to warm up to room temperature overnight. The resulting brown suspension was filtrated with a syringe filter. Then, the solvent was removed *in vacuo*, which yielded a brown oil. Finally, the product (**L10**) was obtained as a colorless oil by vacuum distillation.[5]

Chemical Formula: $C_{12}H_{16}NO_2P$

Molecular Weight [g/mol]: 237.2388

Appearance: colorless oil

Isolated Yield: 68% (1.66 g, 7.00 mmol)

^{1}H NMR (400 MHz, THF-*d_8*): δ = 7.69 (dd, *J*=1.7, 0.8, 1H), 6.59 (dt, *J*=3.3, 0.6, 1H), 6.41 (dt, *J*=3.2, 1.5, 1H), 3.11 (dq, *J*=10.3, 7.0, 4H), 0.94 (t, *J*=7.1, 6H).

^{13}C NMR (101 MHz, THF-*d_8*): δ = 155.91 (d, J=9.8), 146.95 (d, J=3.6), 119.06 (d, J=21.0), 110.81 (d, J=4.0), 45.07 (d, J=16.2), 14.57 (d, J=3.8).

^{31}P NMR (162 MHz, THF-*d_8*): δ = 13.68.

GC-MS: *m/z* (%): 238.13 ($[M+H]^+$, 6), 237.13 (M^+, 41), 222.10 (13), 194.06 (5), 166.04 (11), 165.06 (100), 156.07 (14), 139.09 (4), 137.03 (7), 109.03 (17), 99.01 (9), 83.01 (5), 74.03 (4), 71.03 (4), 70.03 (4).

4.8 Tris(5-methylfuran-2-yl)phosphine (L11)

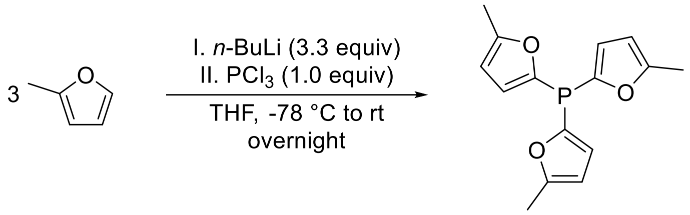

A flame dried Schlenk flask was charged with degassed 2-methylfuran (2.28 g, 27.71 mmol, 3.3 equiv.) and 20 mL of anhydrous THF. Then, *n*-BuLi (2.5 M, 11.1 mL, 27.75 mmol, 3.3 equiv.) was added dropwise to the solution at -78 °C and the temperature was kept for 1h. The reaction mixture was allowed to warm up to room temperature slowly and stir for 2 h. Next, the reaction mixture was cooled again to -78 °C and phosphorus trichloride (1.15 g, 8.39 mmol, 1 equiv.) was slowly injected. After, the reaction mixture was allowed to warm up to room temperature and stir overnight, the solvent was removed *in vacuo*. To remove the lithium chloride, the brownish product was dissolved in anhydrous diethyl ether, and the solution was filtrated with a syringe filter. The collected filtrate was then concentrated, and crystallization was achieved at -20 °C. After, two recrystallisation's colorless crystals of **L11** were obtained.[3]

Chemical Formula: $C_{15}H_{15}O_3P$

Molecular Weight [g/mol]: 274.2558

Appearance: colorless solid

Isolated Yield: 72% (1.65 g, 6.02 mmol)

^{1}H NMR (400 MHz, THF-*d₈*): δ = 6.60 (ddd, *J*=3.2, 1.6, 0.5, 3H), 5.99 (dt, *J*=3.2, 1.2, 3H), 2.27 (d, *J*=1.1, 9H).

^{13}C NMR (101 MHz, THF-d_8): δ = 157.66 (d, J=3.4), 148.70 (d, J=2.0), 122.22 (d, J=21.1), 107.59 (d, J=5.4), 13.50.

^{31}P NMR (162 MHz, THF-d_8): δ = -76.41.

GC-MS: *m/z* (%): 275.11 ($[M+H]^+$, 17), 274.11 (M^+, 100), 273.11 ($[M-H]^+$, 3), 231.08 (10), 213.08 (5), 212.11 (6), 211.13 (5), 210.13 (5), 195.09 (8), 193.06 (6), 185.10 (7), 184.10 (6), 180.05 (5), 174.08 (12), 167.09 (8), 166.04 (19), 163.08 (8), 162.08 (60), 161.08 (17) 159.06 (9), 151.03 (9), 145.08 (8), 119.05 (14), 112.01 (20), 96.99 (13).

4.9 Tris(4,5-dimethylfuran-2-yl)phosphine (L12)

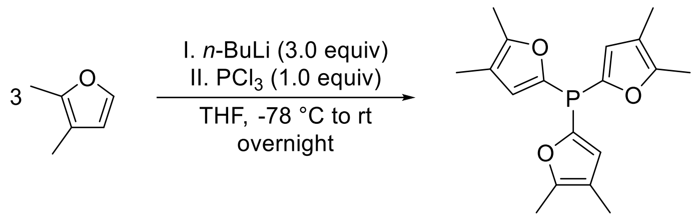

First, 2,3-dimethylfuran (787 mg, 8.19 mmol, 3 equiv.) was weighted in a flame dried Schlenk flask. Then, 20 mL of anhydrous THF were added, and the solution was cooled to -78°C. Under stirring *n*-BuLi (2.5 M, 3.3 mL, 8.19 mmol, 3 equiv.) was slowly added with a syringe and the temperature was kept for 1 h at -78 °C. Afterwards, the reaction mixture was allowed to warm up to room temperature and stir for 2 h. Then, the lithiated dimethylfuran solution was cooled again to -78 °C and phosphorus trichloride (375 mg, 2.73 mmol, 1 equiv.) was slowly added. The solution was warmed up to room temperature and stirred overnight. The solvent was then removed *in vacuo*, yielding a dark orange residue. To remove the lithium chloride, the residue was dissolved in anhydrous diethyl ether and the solution was filtered using a syringe filter. Afterwards, the solvent was removed under vacuum, resulting in an orange oil. Finally, high vacuum distillation yielded **L12** as colorless oil. The ligand crystallized overnight, forming colorless platelet-shaped crystals.

Chemical Formula: $C_{18}H_{21}O_3P$

Molecular Weight [g/mol]: 316.3368

Appearance: colorless oil

Isolated Yield: 42% (362.6 mg, 1.15 mmol)

^{1}H NMR (400 MHz, THF-*d$_8$*): δ = 6.44 (dt, *J*=0.9, 0.4, 3H), 2.17 (s, 9H), 1.88 (s, 9H).

^{13}C NMR (101 MHz, THF-*d$_8$*): δ = 152.83 (d, *J*=3.4), 147.49 (d, *J*=3.5), 124.15 (d, *J*=19.3), 115.93 (d, *J*=5.0), 11.39, 9.52.

^{31}P NMR (162 MHz, THF-*d$_8$*): δ = -76.01.

GC-MS: *m/z* (%): 317.17 ($[M+H]^+$, 21), 316.17 (M^+, 100), 315.17 ($[M-H]^+$, 2), 301.13 (7), 268.18 (6), 254.16 (18), 253.18 (12), 238.16 (12), 237.16 (17), 226.16 (7), 211.14 (9), 202.12 (9), 195.1 (7), 194.08 (37), 191.12 (12), 190.13 (81), 189.12 (26), 173.11 (6), 159.09 (9), 147.09 (14), 126.04 (13), 119.07 (6), 111.01 (9), 91.06 (7).

HRMS (ESI): *m/z* calcd. for $C_{18}H_{21}O_3P$: 317.1306 $[M+H]^+$, found: 317.1306.

IR (ATF): v [cm^{-1}] = 1620 (w), 1456 (m), 1437 (w), 1220 (br), 1193 (w), 1149 (m), 1097 (w), 930 (m), 827 (m), 735 (m), 621 (m).

4.10 Benzofuran-2-yldi(furan-2-yl)phosphine (L13)

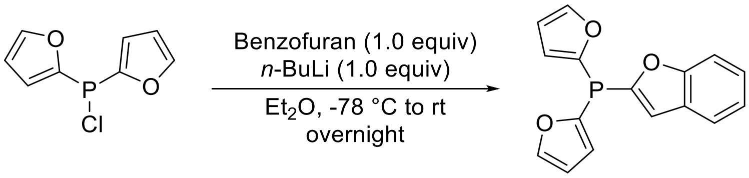

A flame dried Schlenk flask was charged with anhydrous benzofuran (0.99 g, 8.42 mmol, 1 equiv.) and 10 mL of anhydrous diethyl ether. At -20 °C, *n*-BuLi (2.5 M, 3.37 mL, 8.42 mmol, 1 equiv.) was dropwise added to the solution and the temperature was kept for 1h. Then, the reaction mixture was allowed to warm up to room temperature and stir for 2 h. Bis(2-furyl)-phosphine chloride (1.69 g, 8.42 mmol, 1 equiv.) was weighed in a separate Schlenk flask and dissolved in 15 mL of anhydrous diethyl ether. The lithiated benzofuran solution was transferred into a Schlenk dropping funnel and the orange solution was slowly added to the phosphine chloride at -78 °C. After, the completely addition of the benzofuran solution, the reaction mixture was warmed up to room temperature. The lithium chloride was removed from the reaction mixture by anaerobic filtration and the solvent was removed *in vacuo*. Finally, vacuum distillation of the crude oil was performed, yielding a colorless oil as the desired product. Shortly after distillation, the oil completely crystallized.

Chemical Formula: $C_{16}H_{11}O_3P$

Molecular Weight [g/mol]: 282.2348

Appearance: white solid

Isolated Yield: 78% (1.86 g, 6.59 mmol)

^{1}H NMR (400 MHz, THF-*d$_8$*): δ = 7.76 (dt, *J* = 1.9, 0.9, 2H), 7.57 - 7.51 (m, 1H), 7.45 (dd, *J* = 8.3, 1.0, 1H), 7.26 (ddd, *J* = 8.3, 7.2, 1.4, 1H), 7.17 (td, *J* = 7.5, 1.1, 1H), 7.00 (t, *J* = 1.1, 1H), 6.93 - 6.88 (m, 2H), 6.47 (dt, *J* = 3.4, 1.7, 2H).

^{13}C NMR (101 MHz, THF-*d$_8$*): δ = 158.70 (d, *J*=3.9), 154.15, 148.84 (d, *J*=2.5), 148.73 (d, *J*=2.8), 128.87 (d, *J*=4.6), 125.67, 123.42, 122.46 (d, J=24.2), 121.78, 116.79 (d, *J*=18.0), 111.85, 111.48 (d, *J*=6.2).

^{31}P NMR (122 MHz, THF-*d$_8$*): δ = -73.92.

GC-MS: *m/z* (%): 283.09 ([M+H]$^+$, 19), 282.10 (M$^+$, 100), 281.09 ([M-H]$^+$, 7), 235.11 (9), 219.11 (7), 218.11 (8), 207.11 (14), 202.05 (21), 186.05 (8), 185.09 (9), 184.09 (65),

179.11 (7), 178.11 (9), 157.05 (8), 155.07 (13), 148.03 (10), 139.07 (9), 133.04 (10), 128.08 (13), 120.03 (15), 98.01 (11), 70.02 (10).

HRMS (ESI): *m/z* calcd. for $C_{16}H_{11}O_3P$: 305.0338 $[M+Na]^+$, found: 305.0335.

IR (ATF): **ν** [cm^{-1}] = 1442 (m), 1252 (m), 1163 (m), 1147 (m), 1115 (m), 1005 (s), 901 (m), 816 (m), 791 (w), 746 (s), 733 (s), 636 (w), 629 (m), 592 (w), 507 (m).

4.11 Di(benzofuran-2-yl)(furan-2-yl)phosphine (L14)

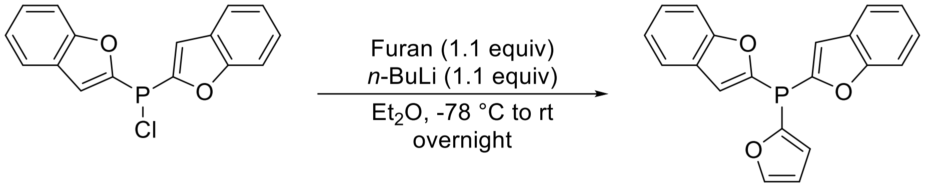

Degassed furan (142 mg, 2.1 mmol, 1.05 equiv.) was charged together with 20 mL of anhydrous diethyl ether in a 100 mL Schlenk flask. At -78 °C, *n*-BuLi (2.5 M, 0.85 mL, 2.1 mmol, 1.05 equiv.) was added dropwise to the solution under stirring. The reaction mixture was allowed to warm up to room temperature slowly and stir for 2 h. A separate Schlenk flask was charged with bis(benzofuran-2-yl)-phosphine chloride (601 g, 2 mmol, 1 equiv.) and 15 mL of anhydrous diethyl ether. The lithiated furan solution was transferred into a Schlenk dropping funnel and the solution was dropwise added to the phosphine chloride solution at - 78 °C. Afterwards, the reaction mixture was slowly warmed to room temperature and stirred for 2 hours. The resulting suspension was filtered using a syringe filter. Next, the solvent was removed *in vacuo*, which yielded a slightly yellow oil. Finally, the target phosphine was purified by vacuum distillation and a colorless oil was obtained. Shortly after distillation, the oil started crystallization.

Chemical Formula: $C_{20}H_{13}O_3P$

Molecular Weight [g/mol]: 332.2948

Appearance: white solid

Isolated Yield: 58% (385 mg, 1.16 mmol)

^{1}H NMR (400 MHz, THF-*d*$_8$): δ = 7.83 (dt, *J*=1.6, 0.7, 1H), 7.57 (ddd, *J*=7.7, 1.3, 0.7, 2H), 7.48 (dq, *J*=8.3, 0.9, 2H), 7.28 (ddd, *J*=8.4, 7.2, 1.4, 2H), 7.23 - 7.14 (m, 4H), 7.05 (ddd, *J*=3.3, 2.0, 0.7, 1H), 6.52 (dt, *J*=3.4, 1.7, 1H).

^{13}C NMR (101 MHz, THF-*d*$_8$): δ = 159.12 (d, *J*=3.5), 158.82, 156.96, 153.44, 153.34, 132.37 (d, *J*=2.2), 128.89 (d, *J*=5.4), 128.22, 125.98, 125.00 (d, *J*=3.5), 124.51 (d, *J*=2.3), 124.05 (d, *J*=1.8), 123.71, 123.56, 123.06, 121.98, 121.45, 118.91, 118.71, 116.86, 116.78, 112.36, 112.02.

^{31}P NMR (161.98 MHz, THF-*d*$_8$): δ = -70.87.

GC-MS: *m/z* (%): 333.04 ([M+H]$^+$, 23), 332.05 (M$^+$, 100), 331.05 ([M-H]$^+$, 10), 285.06 (8), 268.07 (7), 257.07 (11), 235.06 (15), 234.06 (81), 205.04 (10), 189.04 (8), 184.02 (17), 168.03 (7), 155.02 (7), 147.99 (25), 139.03 (7), 132.99 (7), 119.99 (21).

HRMS (ESI): *m/z* calcd. for $C_{20}H_{13}O_3P$: 355.0494 [M+Na]$^+$, found: 355.0500.

IR (ATF): v [cm^{-1}] = 1441 (m), 1264 (m), 1252 (m), 1163 (m), 1111 (m), 1065 (w), 1007 (m), 921 (m), 792 (m), 732 (s).

4.12 Tri(benzofuran-2-yl)phosphine (L15)

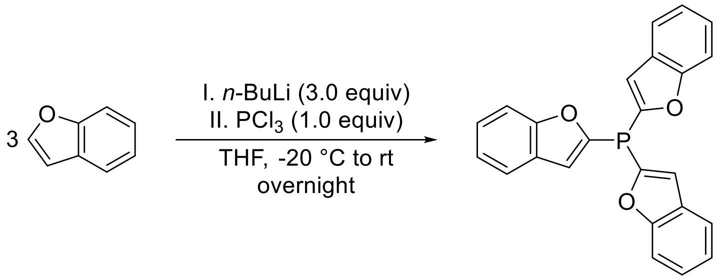

Tri(benzofuran-2-yl)phosphine was synthesized according to the literature with some modifications.[8] Under argon atmosphere, anhydrous benzofuran (2.52 g, 21.35 mmol, 3 equiv.) was dissolved in 30 mL anhydrous THF. The solution was cooled to -20 °C and then *n*-BuLi (2.5 M, 8.54 mL, 21.35 mmol, 3 equiv.) was slowly added. The yellow solution was allowed to warm up to room temperature and stir for 2 h. Next, the reaction mixture was cooled again to -20 °C and phosphorus trichloride (978 mg, 7.12 mmol, 1 equiv.) was slowly added. The reaction temperature was kept at -20 °C for 1 h, before the solution was warmed up to room temperature. After overnight stirring, the solvent was removed *in vacuo* and a yellow solid was obtained. To remove the lithium chloride, the yellow solid was dissolved in diethyl ether and the resulting suspension was filtrated. Finally, the desired product was obtained by column chromatography using *n*-hexane and 5% ethyl acetate.

Chemical Formula: $C_{24}H_{15}O_3P$

Molecular Weight [g/mol]: 382.3548

Appearance: white solid

Isolated Yield: 73% (1.99 g, 5.20 mmol)

^{1}H NMR (400 MHz, THF-*d$_8$*): δ = 7.60 (dt, *J* = 7.8, 1.2, 3H), 7.52 (dt, *J* = 8.3, 1.0, 3H), 7.36 - 7.26 (m, 6H), 7.21 (td, *J* = 7.6, 1.1, 3H).

^{13}C NMR (101 MHz, THF-*d$_8$*): δ = 158.99 (d, *J*=3.6), 151.86 (d, *J*=3.8), 128.77 (d, *J*=6.2), 126.23, 123.67, 122.10, 118.86 (d, *J*=21.4), 112.09.

^{31}P NMR (122 MHz, THF-*d$_8$*): δ = -67.32.

GC-MS: *m/z* (%): 383.05 ([M+H]$^+$, 22), 382.05 (M$^+$, 85), 381.05 ([M-H]$^+$, 13), 318.06 (4), 291.07 (5), 289.01 (9), 276.01 (6), 263.99 (16), 246.03 (9), 236.02 (7), 235.04 (18), 234.04 (100), 218.04 (9), 205.03 (16), 189.03 (18), 147.97 (58), 119.97 (28).

HRMS (ESI): *m/z* calcd. for $C_{24}H_{15}O_3P$: 383.0837 $[M+H]^+$, found: 383.0841.

IR (ATF): **ν** [cm^{-1}] = 1439 (m), 1252 (m), 1159 (m), 1109 (m), 1063 (m), 920 (m), 793 (s), 743 (s), 625 (m).

4.13 Tris(benzo[1,2-b:4,5-b']difuran-2-yl)phosphine (L16)

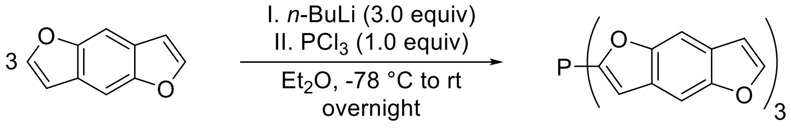

First, 5*H*-indeno[5,6-*b*]furan (433 mg, 2.74 mmol, 3 equiv.) was dissolved in 15 mL of anhydrous diethyl ether in a Schlenk flask. The solution was then cooled to -78 °C, and *n*-BuLi (2.5 M, 1.1 mL, 2.75 mmol, 3 equiv.) was added dropwise using a syringe. After the complete addition of *n*-BuLi, the reaction mixture was slowly warmed to room temperature and stirred for 2 h. The yellow solution was cooled again to -78 °C and phosphorus trichloride (125 mg, 0.91 mmol, 1 equiv.) was dropwise added to the reaction mixture. Then, the solution was allowed to warm to room temperature. As the next step, the light-yellow suspension was filtrated with a syringe filter to remove the lithium chloride. The solvent of the resulting yellow solution was removed *in vacuo*. Finally, the desired product was obtained after column chromatography using *n*-hexane and 5% ethyl acetate.

Chemical Formula: $C_{30}H_{15}O_6P$

Molecular Weight [g/mol]: 502.4178

Appearance: white solid

Isolated Yield: 46% (211 mg, 0.42 mmol)

^{1}H NMR (300 MHz, THF-*d₈*): δ = 7.82 - 7.73 (m, 3H), 7.69 (dt, *J* = 6.0, 0.9 Hz, 6H), 7.39 (dd, *J* = 1.7, 1.0 Hz, 3H), 6.88 (dd, *J* = 2.3, 1.0 Hz, 3H).

^{13}C NMR (101 MHz, THF-d_8): δ = 155.99 (d, *J*=4.4), 153.02, 152.57 (d, *J*=3.6), 147.57, 127.85, 126.77 (d, *J*=5.4), 119.16, 118.96, 107.40, 102.75 (d, *J*=1.8).

^{31}P NMR (122 MHz, THF-d_8): δ = -65.79.

HRMS (ESI): *m/z* calcd. for $C_{30}H_{15}O_6P$: 503.0685 $[M+H]^+$, found: 503.0684

IR (ATF): **ν** [cm^{-1}] = 1545 (m), 1425 (m), 1375 (m), 1304 (w), 1169 (m), 1161 (m), 1115 (m), 1090 (m), 1024 (s), 914 (m), 843 (s), 769 (m), 702 (m), 636 (m).

4.14 Tri(benzofuran-2-yl)phosphine oxide (L17)

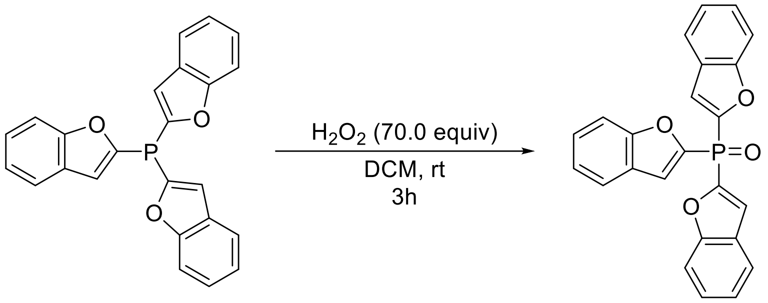

A 100 mL round bottom flask was charged with tri(benzofuran-2-yl)phosphine (268 mg, 0.7 mmol, 1 equiv.) and a stirring bar. After, the addition of 30 mL DCM, hydrogen peroxide (30 wt%, 49 mmol, 70 equiv.) was slowly injected while stirring. The reaction mixture was allowed to stir for 3 h at room temperature. Then, the mixture was transferred into a separating funnel and the organic layer was washed twice with water. After, the organic phase was dried with sodium sulphate, the solvent was evaporated.[11] Finally, a white solid was obtained after column chromatography.[12]

Chemical Formula: $C_{24}H_{15}O_4P$

Molecular Weight [g/mol]: 398.3538

Appearance: white solid

Isolated Yield: 58% (163 mg, 0.41 mmol)

^{1}H NMR (400 MHz, THF) δ = 7.78 - 7.68 (m, 6H), 7.59 (dq, *J*=8.4, 0.9, 3H), 7.48 - 7.39 (m, 3H), 7.31 (ddd, *J*=8.0, 7.2, 0.9, 3H).

^{13}C NMR (101 MHz, THF) δ = 158.85, 158.75, 150.06, 148.57, 127.93, 127.62, 127.52, 124.39, 123.33, 120.75, 120.54, 112.75.

^{31}P NMR (162 MHz, THF) δ = -11.96.

GC-MS: *m/z* (%): 399.10 ($[M+H]^+$, 8), 398.10 (M^+, 30), 382.10 (9), 266.06 (18), 265.06 (100), 236.06 (9), 235.08 (7), 234.08 (38), 218.09 (11), 207.05 (16), 205.07 (13), 189.08 (12), 176.07 (6), 148.00 (9), 120.01 (8), 89.03 (11), 63.04 (8).

4.15 Tris(3-methylbenzofuran-2-yl)phosphine (L18)

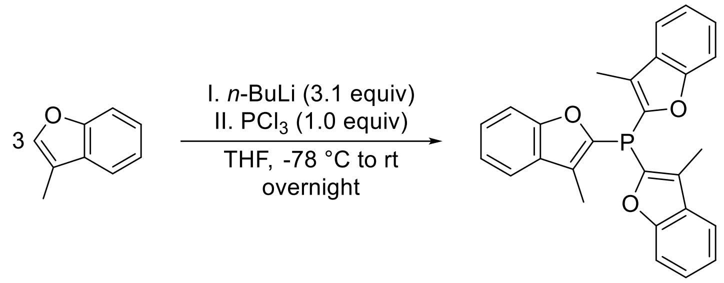

Under argon atmosphere, 3-methylbenzofuran (1.05 g, 7.91 mmol, 3.1 equiv.) was dissolved in 20 mL of anhydrous THF. Then, *n*-BuLi (2.5 mM, 3.16 mL, 7.91 mmol, 3.1 equiv.) was slowly added to the solution at -78 °C. The reaction mixture was kept at -78 °C for 1 h, before the solution was warmed to room temperature. The solution stirred for 2 h at room temperature. At -78 °C, phosphorus trichloride (0.35 g, 2.55 mmol, 1 equiv.) was dropwise injected to the lithiated benzofuran compound. The reaction mixture was allowed to slowly warm to room temperature and stirred overnight. Next, the solvent was removed *in vacuo*, and the resulting white solid was dissolved in anhydrous DCM. The obtained white suspension was filtered under anaerobic conditions through a syringe filter to remove lithium chloride. Afterwards, the solvent was evaporated, and a white solid was received. The target phosphine **L18** was purified by column chromatography using *n*-hexane and 0.5% ethyl acetate.

Chemical Formula: $C_{27}H_{21}O_3P$

Molecular Weight [g/mol]: 424.4358

Appearance: white solid

Isolated Yield: 86% (0.93 g, 2.19 mmol)

^{1}H NMR (400 MHz, THF-*d_8*): δ = 7.57 - 7.50 (m, 3H), 7.45 (dt, *J*=8.2, 0.9, 3H), 7.28 (ddd, *J*=8.3, 7.1, 1.4, 3H), 7.19 (td, *J*=7.4, 1.0, 3H), 2.38 (s, 9H).

^{13}C NMR (101 MHz, THF-*d_8*): δ = 158.08 (d, *J*=2.6), 146.54 (d, *J*=9.9), 130.09 (d, *J*=6.2), 127.58 (d, *J*=28.6), 126.16, 122.96, 120.29 (d, *J*=1.8), 112.00, 8.95 (d, *J*=9.9).

^{31}P NMR (122 MHz, THF-*d_8*): δ = -88.93.

GC-MS: *m/z* (%): 425.14 ($[M+H]^+$, 12), 424.13 (M^+, 43), 409.10 ($[M-H]^+$, 9), 292.05 (11), 274.08 (8), 263.10 (20), 262.09 (100), 261.09 (29), 260.07 (5), 259.06 (16), 247.07 (9),

218.07 (6), 215.09 (5), 202.07 (6), 162.00 (35), 133 (12), 131.03 (9), 115.03 (45), 102.03 (6), 77.02 (10).

HRMS (ESI): *m/z* calcd. for $C_{27}H_{21}O_3P$: 447.1120 $[M+Na]^+$, found: 447.1119.

IR (ATF): **ν** [cm^{-1}] = 1444 (m), 1382 (w), 1338 (br), 1230 (m), 1134 (m), 1097 (m), 1082 (m), 930 (w), 798 (m), 742 (s), 715 (m), 646 (m).

4.16 Di(benzofuran-2-yl)chlorophosphine

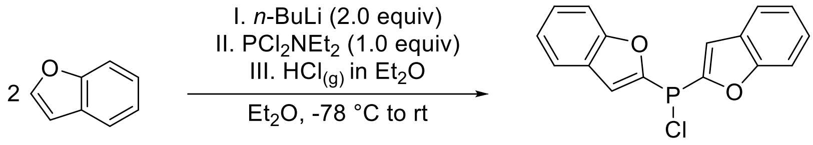

A flame dried 250 mL Schlenk flask was charged with degassed benzofuran (9.40 g, 79.6 mmol, 2 equiv.) and 50 mL of anhydrous diethyl ether. At -20 °C, *n*-BuLi (2.5 M, 31.8 mL, 79.6 mmol, 2 equiv.) was added dropwise to the solution, and the reaction mixture was allowed to warm to room temperature. After stirring for 2 h, the orange solution was cooled again to -20 °C and dichloro(diethylamino)phosphine (7.00 g, 40 mmol, 1 equiv.) was added slowly via syringe. The reaction mixture stirred overnight. Then, ethereal HCl (2.0 M, 44 mL, 88 mmol, 2.1 equiv.) was transferred into a Schlenk dropping funnel and was dropwise added. The resulting suspension was filtered with a Schlenk frit packed with Celite®. Finally, the solvent was removed *in vacuo* from the received yellowish solution, yielding a slightly yellow solid.[9]

Chemical Formula: $C_{30}H_{15}O_6P$

Molecular Weight [g/mol]: 300.6778

Appearance: yellow to white solid

Isolated Yield: 64% (7.69 g, 25.6 mmol)

^{1}H NMR (400 MHz, THF-*d_8*): δ = 7.67 (ddd, *J*=7.8, 1.2, 0.7, 2H), 7.63 - 7.51 (m, 4H), 7.40 (ddd, *J*=8.4, 7.2, 1.3, 2H), 7.32 - 7.22 (m, 2H).

^{13}C NMR (101 MHz, THF-*d_8*): δ = 159.20 (d, *J*=3.1), 153.43 (d, *J*=34.6), 128.18 (d, *J*=6.7), 127.47, 124.10, 122.83, 119.98 (d, *J*=29.2), 112.39.

^{31}P NMR (162 MHz, THF-*d_8*): δ = 24.08.

4.17 Di(benzofuran-2-yl)(methyl)phosphine (L19)

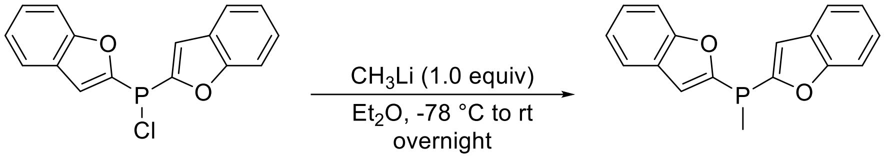

Under argon atmosphere, di(benzofuran-2-yl)chlorophosphine (601 mg, 2 mmol, 1 equiv.) was charged in a 100 mL Schlenk flask. Then, 25 mL of anhydrous diethyl ether was added, and the solution was cooled to -78 °C. Next, a methyllithium solution (1.6 M, 1.25 mL, 2 mmol, 1 equiv.) was dropwise injected and the solution was allowed to stir for 1 h at -78 °C. The reaction mixture was slowly warmed to room temperature and stirred overnight. After, the suspension was anaerobically filtrated a light-yellow solution was received. Finally, the solvent was removed *in vacuo* and the received yellow solid was recrystallized in DCM, yielding colorless crystals.

Chemical Formula: $C_{17}H_{13}O_2P$

Molecular Weight [g/mol]: 280.2628

Appearance: white solid

Isolated Yield: 71% (398 mg, 1.42 mmol)

^{1}H NMR (400 MHz, THF-*d_8*): δ = 7.55 (ddd, *J*=7.7, 1.4, 0.8, 2H), 7.45 (dq, *J*=8.3, 1.0, 2H), 7.28 - 7.21 (m, 2H), 7.20 - 7.16 (m, 2H), 7.15 (dd, *J*=1.7, 1.0, 2H), 1.82 (d, *J*=4.1, 3H).

^{13}C NMR (101 MHz, THF-*d_8*): δ = 158.63 (d, *J*=2.1), 156.59 (d, *J*=19.3), 128.97 (d, *J*=5.5), 125.62, 123.40, 121.74, 116.04 (d, *J*=21.9), 111.79, 8.31 (d, *J*=5.5).

^{31}P NMR (162 MHz, THF-*d_8*): δ = -63.12.

GC-MS: *m/z* (%): 281.06 (M^+, 18), 280.08 ($[M-H]^+$, 79), 266.06 (18), 265.05 (100), 237.05 (6), 236.05 (13), 235.07 (8), 234.08 (43), 218.08 (18), 209.04 (5), 208.05 (6), 207.04 (26), 205.04 (5), 190.08 (5), 189.07 (22), 132.97 (5), 131.01 (5), 119.99 (8), 118.03 (5), 115.05 (5), 89.03 (7), 63.00 (8).

HRMS (ESI): *m/z* calcd. for $C_{17}H_{13}O_2P$: 281.0731 $[M+H]^+$, found 281.0726.

IR (ATF): **ν** [cm^{-1}] = 1441 (m), 1252 (m), 1225 (w), 1161 (m), 1109 (w), 1055 (w), 920 (m), 874 (m), 791 (m), 750 (s).

4.18 Di(benzofuran-2-yl)(*tert*-butyl)phosphine (L20)

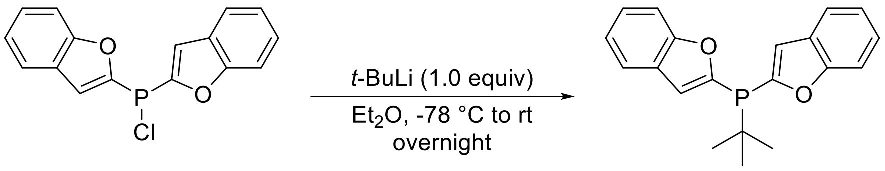

The synthesis was carried out following the same procedure as described for di(benzofuran-2-yl)(methyl)phosphine (**L19**) with *t*-BuLi (1.7 M, 1.18 mL, 2 mmol, 1 equiv.) instead of methyllithium. The desired product was obtained after performing column chromatography under inert conditions, using *n*-hexane with 3% ethyl acetate as the eluent.

Chemical Formula: $C_{20}H_{19}O_2P$

Molecular Weight [g/mol]: 322.3438

Appearance: white solid

Isolated Yield: 53% (342 mg, 1.06 mmol)

^{1}H NMR (400 MHz, THF-*d₈*): δ = 7.64 - 7.51 (m, 4H), 7.37 - 7.16 (m, 6H), 1.26 (d, *J* = 13.8 Hz, 9H).

^{13}C NMR (101 MHz, THF-d_8): δ = 157.93 (d), 154.00, 127.90 (d), 124.99, 122.69, 121.04, 118.65 (d), 111.03, 32.17 (d), 27.95 (d).

^{31}P NMR (162 MHz, THF-d_8): δ = -23.16.

GC-MS: *m/z* (%): 323.08 ($[M+H]^+$, 12), 322.08 (M^+, 49), 267.03 (22), 266.05 (100), 265.05 (77), 237.01 (8), 236.01 (12), 235.06 (3), 234.03 (13), 219.03 (7), 218.03 (18), 206.99 (11), 203.02 (4), 190.02 (5), 189.02 (23), 182.98 (4), 148.96 (13), 147.98 (97), 119.96 (17), 57.06 (11).

IR (ATF): v [cm^{-1}] = 1441 (m), 1250 (m), 918 (m), 918(m), 791 (m), 748 (s), 741 (br), 636 (m).

4.19 Di(benzofuran-2-yl)(1,3-dioxolan-2-yl)phosphine (L21)

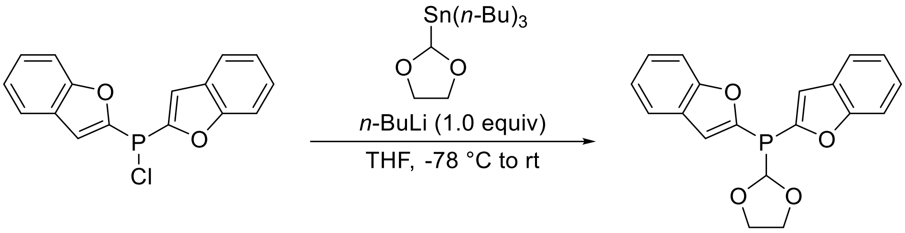

According to the procedure of Shiner *et al.*, 2-Lithio-1,3-dioxolan was prepared from (1,3-dioxolan-2-yl)tri-*n*-butylstannane (381 mg, 1.05 mmol, 1 equiv.) by the addition of *n*-BuLi (2.5 M, 0.42 mL, 1.05 mmol, 1 equiv.) at -78 °C.[10] Next, a separate 100 mL Schlenk flask was charged with bis(benzofuran-2-yl)-phosphine chloride (301 g, 1 mmol, 1 equiv.) and 10 mL of anhydrous THF. At -78 °C, the 2-Lithio-1,3-dioxolan THF solution was slowly added to phosphine chloride solution and the temperature was kept for 1 h at -78 °C. Then, the reaction mixture was allowed to warm up slowly to room temperature and stir overnight. After, the solvent was removed *in vacuo*, the received yellow solid was dissolved in anhydrous DCM and the yellow solution was anaerobic filtrated with a syringe filter. As the next step, **L21** was purified by an anaerobic column chromatography using anhydrous *n*-hexane and DCM. After, the solvent was evaporated a colorless oil was received, which slowly became solid. Finally, colorless crystals of **L21** were received from a concentrated DCM solution layered with pentane.

Chemical Formula: $C_{19}H_{15}O_4P$

Molecular Weight [g/mol]: 338.2988

Appearance: white solid

Isolated Yield: 34% (115 mg, 0.34 mmol)

^{1}H NMR (400 MHz, THF-*d$_8$*): δ = 7.58 (ddd, *J*=7.7, 1.4, 0.7, 2H), 7.49 (dq, *J*=8.3, 0.9, 2H), 7.34 - 7.23 (m, 4H), 7.23 - 7.15 (m, 2H), 6.41 (d, *J*=8.6, 1H), 4.14 - 3.86 (m, 4H).

^{13}C NMR (101 MHz, THF-*d$_8$*): δ = 158.68 (d, *J*=2.4), 153.06 (d, *J*=16.5), 128.78 (d, *J*=5.8), 125.87, 123.45, 121.86, 118.36 (d, *J*=19.5), 111.90, 106.94 (d, *J*=16.5), 66.02 (d, *J*=3.0).

^{31}P NMR (161.98 MHz, THF-*d$_8$*): δ = -62.86.

GC-MS: *m/z* (%): 338.07 (M^+, 5), 281.04 (2), 266.05 (4), 265.04 (13), 253.02 (2), 237.06 (2), 236.05 (4), 234.06 (2), 218.07 (5), 209.03 (4), 208.04 (5), 207.03 (24), 191.01 (2),

190.06 (2), 189.06 (9), 183.02 (2), 165.05 (2), 147.98 (5), 132.96 (2), 119.99 (5), 74.03 (4), 73.02 (100), 63.02 (1).

HRMS (ESI): *m/z* calcd. for $C_{19}H_{15}O_4P$: 366.0600 $[M+Na]^+$, found: 366.0608

4.20 Di(benzofuran-2-yl)(methyl)phosphine (L22)

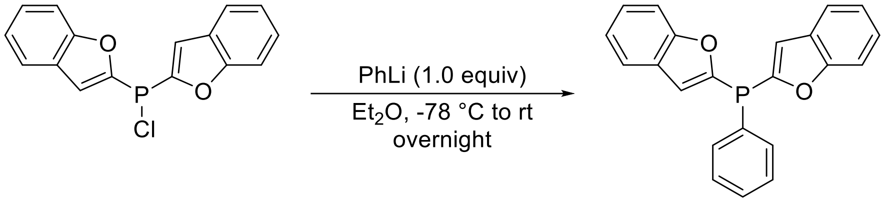

The synthesis was carried out following the same procedure as described for di(benzofuran-2-yl)(methyl)phosphine (**L19**) with phenyllithium (1.9 M, 1.05 mL, 2 mmol, 1 equiv.) instead of methyllithium. The desired product was obtained after column chromatography was performed using *n*-hexane and 5% ethyl acetate.

Chemical Formula: $C_{22}H_{15}O_2P$

Molecular Weight [g/mol]: 342.3338

Appearance: white solid

Isolated Yield: 47% (322 mg, 0.94 mmol)

^{1}H NMR (400 MHz, THF-*d$_8$*): δ = 7.55 (ddd, J=7.7, 1.4, 0.8, 2H), 7.45 (dq, J=8.3, 1.0, 2H), 7.28 - 7.21 (m, 2H), 7.20 - 7.16 (m, 2H), 7.15 (dd, J=1.7, 1.0, 2H), 1.82 (d, J=4.1, 3H).

^{13}C NMR (75.49 MHz, THF-*d$_8$*): δ = 159.02 (d, J=3.0), 154.79 (d, J=11.1), 134.21 (d, J=21.0), 133.63 (d, J=2.9), 130.36, 129.35 (d, J=7.7), 128.83 (d, J=5.3), 125.91, 123.54, 121.91, 118.30 (d, J=20.2), 111.96.

^{31}P NMR (162 MHz, THF-*d$_8$*): δ = -44.83.

GC-MS: *m/z* (%): 343.10 ($[M+H]^+$, 24) 342.10 (M^+, 100) 341.11 ($[M-H]^+$, 14), 265.06 (17), 264.05 (7), 249.06 (6), 236.07 (9), 235.09 (15), 234.09 (85), 224.05 (8), 218.08 (11), 207.06 (9), 205.08 (10), 194.07 (17), 189.07 (12), 178.08 (9), 165.07 (15), 148.00 (43), 120.01 (18), 108.01 (9), 107.00 (7).

HRMS (ESI): *m/z* calcd. for $C_{22}H_{15}O_2P$: 343.0888 $[M+H]^+$, found: 343.0888.

IR (ATF): v [cm^{-1}] = 1530 (w), 1470 (w), 1441 (m), 1436 (m), 1295 (w), 1250 (m), 1159 (m), 1108 (m), 1062 (m), 918 (m), 751 (m), 742 (m).

4.21 Di(benzofuran-2-yl)(2-methoxyphenyl)phosphine (L23)

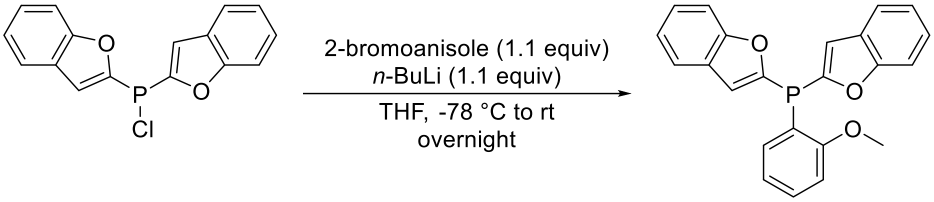

Under argon atmosphere, di(benzofuran-2-yl)chlorophosphine (301 mg, 1 mmol, 1 equiv.) was charged together with 15 mL of anhydrous THF in a 100 mL Schlenk flask and the solution was cooled to -78 °C. Then, another Schlenk flask was charged with degassed 2-bromoanisole (206 mg, 1.1 mmol, 1.1 equiv.) and 10 mL anhydrous THF was added. At -78 °C, *n*-BuLi (2.5 M, 0.44 mL, 1.1 mmol, 1 equiv.) was added dropwise to the 2-bromoanisole solution and the temperature was kept for 1h. Then, the lithiated anisole compound was dropwise transferred to the phosphine chloride solution at -78 °C. The reaction mixture was stirred for 1h at -78 °C. Afterwards, the mixture was slowly warmed to room temperature and stirred overnight. Next, the solvent was removed *in vacuo,* which yielded a yellow solid. The desired product was obtained by column chromatography using *n*-hexane and 15% ethyl acetate. Finally, the solvent was evaporated, and a white crystalline solid was obtained, **L23** was stored under argon atmosphere.

Chemical Formula: $C_{23}H_{17}O_3P$

Molecular Weight [g/mol]: 372.3598

Appearance: white solid

Isolated Yield: 42% (156 mg, 0.42 mmol)

^{1}H NMR (400 MHz, THF-*d$_8$*): δ = 7.55 (ddd, J=7.7, 1.4, 0.7, 2H), 7.46 (dq, J=8.3, 0.9, 2H), 7.41 - 7.32 (m, 1H), 7.30 - 7.22 (m, 2H), 7.21 - 7.15 (m, 2H), 7.14 - 7.08 (m, 1H), 7.01 (q, J=1.3, 2H), 7.00 - 6.96 (m, 1H), 6.89 (tt, J=7.5, 0.9, 1H), 3.76 (s, 3H).

^{13}C NMR (75.49 MHz, THF-*d$_8$*): δ = 162.16 (d, J=16.6), 158.93 (d, J=3.3), 155.13 (d, J=11.3), 134.07, 131.83, 129.05 (d, J=5.1), 125.57, 123.37, 122.01 (d, J=4.0), 121.75, 121.71, 117.83 (d, J=18.5), 111.91, 111.33 (d, J=2.1), 55.83.

^{31}P NMR (162 MHz, THF-*d$_8$*): δ = -44.83.

GC-MS: *m/z* (%): 373.08 ([M+H]$^+$, 25), 372.07 (M$^+$, 100), 371.13 ([M-H]$^+$, 6), 357.04 (6), 341.04 (7), 265.02 (8), 264.01 (10), 254.03 (6), 241.02 (20), 239.02 (11), 236.04 (7), 235.07 (7), 234.06 (32), 224.07 (11), 223.05 (9), 207.04 (10), 189.06 (8), 165.06 (7), 148.00 (10), 137.02 (11), 131.05 (29), 120.01 (7), 91.06 (9).

HRMS (ESI): *m/z* calcd. for $C_{23}H_{17}O_3P$: 373,0993 [M+H]$^+$, found: 373.0992.

IR (ATF): **ν** [cm^{-1}] = 1471 (w), 1442 (w), 1264 (m), 1252 (m), 1162 (w), 1111 (w), 1023 (w), 921 (w), 792 (w), 731 (s), 702 (w).

4.22 Di(benzofuran-2-yl)(dibenzo[*b,d*]furan-4-yl)phosphine (L24)

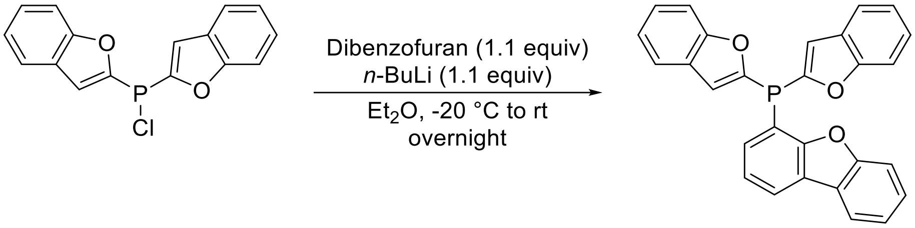

A flame dried Schlenk flask was charged with dibenzofuran (353 mg, 2.1 mmol, 1.05 equiv.) and 10 mL of anhydrous diethyl ether. At -20 °C, *n*-BuLi (2.5 M, 0.85 mL, 2.1 mmol, 1.05 equiv.) was added dropwise to the solution and the temperature was kept for 1h. Then, the reaction mixture was allowed to slowly warm to room temperature and stirred for 2 hours. Bis(2-furyl)-phosphine chloride (601 mg, 2 mmol, 1 equiv.) was weighed in a separate Schlenk flask and was dissolved in 15 mL of anhydrous diethyl ether. The lithiated benzofuran solution was transferred into a Schlenk dropping funnel and the orange solution was slowly added to the phosphine chloride at -78 °C. After the completely addition of the benzofuran solution, the reaction mixture was warmed to room temperature. Next, the lithium chloride was removed from the reaction mixture by anaerobic filtration with a syringe filter and the solvent was removed *in vacuo*. Then, column chromatography was performed using *n*-hexane and 0.5% ethyl acetate, yielding a white solid as the desired product.

Chemical Formula: $C_{28}H_{17}O_3P$

Molecular Weight [g/mol]: 432.4148

Appearance: white solid

Isolated Yield: 63% (545 mg, 1.26 mmol)

^{1}H NMR (400 MHz, THF-*d$_8$*): δ = 8.10 (dd, *J*=7.7, 1.3, 1H), 8.03 (ddd, *J*=7.7, 1.4, 0.7, 1H), 7.57 (ddd, *J*=7.8, 1.4, 0.7, 2H), 7.55 - 7.46 (m, 4H), 7.46 - 7.40 (m, 1H), 7.40 - 7.25 (m, 4H), 7.24 - 7.14 (m, 4H).

^{13}C NMR (101 MHz, THF-*d$_8$*): δ = 159.12 (d, J=3.5), 158.74 (d, J=17.9), 156.96, 153.39 (d, J=9.4), 132.37 (d, J=2.2), 128.89 (d, J=5.4), 128.22, 125.98, 125.00 (d, J=3.5), 124.51 (d, J=2.3), 124.05 (d, J=1.8), 123.71, 123.56, 123.06, 121.98, 121.45, 118.81 (d, J=20.6), 116.82 (d, J=8.4), 112.36, 112.02.

^{31}P NMR (162 MHz, THF-*d$_8$*): δ = -58.49.

GC-MS: *m/z* (%): 433.14 ([M+H]$^+$, 30), 432.13 (M$^+$, 100), 431.14 ([M-H]$^+$, 17), 339.07 (9), 314.06 (13), 296.09 (6), 285.09 (8), 284.1 (36), 281.06 (19), 265.06 (9), 264.06 (13), 255.09 (11), 239.11 (6), 235.07 (7), 234.09 (39), 218.08 (8), 209.06 (6), 208.05 (9), 207.06 (42), 198.04 (20), 197.03 (8), 170.03 (6), 148.03 (21), 120.01 (7).

HRMS (ESI): *m/z* calcd. for $C_{28}H_{17}O_3P$: 455.0807 [M+Na]$^+$, found: 455.0808.

IR (ATF): v [cm^{-1}] = 1441 (w), 1400 (w), 1252 (w), 1182 (m), 1163 (w), 1057 (w), 918 (w), 833 (m), 791 (m), 746 (s), 634 (m).

4.23 Tri(benzofuran-3-yl)phosphine (L25)

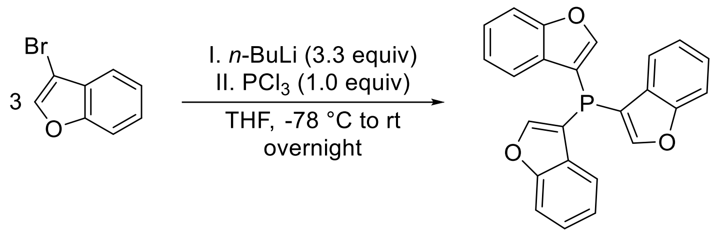

First, 3-bromobenzofuran (450 mg, 2.28 mmol, 3 equiv.) was weighed in a flame dried Schlenk flask. Then, 12 mL of anhydrous THF were added, and the solution was cooled to -78 °C. Next, *n*-BuLi (2.5 M, 1 mL, 2.50 mmol, 3.3 equiv.) was dropwise added to the solution, which resulted in a fast color change to yellow. After, the solution stirred for 1 h at -78 °C, phosphorus trichloride (104 mg, 0.76 mmol, 1 equiv.) was slowly added. The reaction mixture was allowed to warm to room temperature and stirred overnight. A light-yellow solution was received after the suspension was filtrated. The solvent was removed *in vacuo*, whereby a yellow solid was obtained. The desired product was isolated by column chromatography using *n*-hexane and 5% ethyl acetate.

Chemical Formula: $C_{24}H_{15}O_3P$

Molecular Weight [g/mol]: 382.3548

Appearance: white solid

Isolated Yield: 78% (226 mg, 0.59 mmol)

^{1}H NMR (400 MHz, THF-*d₈*): δ = 7.70 (d, *J*=1.5, 1H), 7.59 - 7.54 (m, 1H), 7.52 (dq, *J*=8.3, 1.1, 1H), 7.29 (ddd, *J*=8.3, 7.2, 1.3, 1H), 7.19 - 7.13 (m, 1H).

^{13}C NMR (101 MHz, THF-d_8): δ = 156.30 (d, *J*=5.4), 149.51 (d, *J*=19.0), 128.88 (d, *J*=15.3), 124.67, 122.88, 120.84 (d, *J*=2.6), 111.36, 111.28.

^{31}P NMR (122 MHz, THF-d_8): δ = -86.33.

GC-MS: *m/z* (%): 383.11 ($[M+H]^+$, 26), 382.12 (M^+, 100), 381.12 ($[M-H]^+$, 20), 289.08 (7), 264.06 (6), 263.06 (17), 236.07 (8), 234.09 (10), 218.1 (9), 207.07 (15), 205.08 (7), 189.08 (11), 176.08 (4), 148.01 (30), 120.02 (18), 89.05 (5), 63.04 (5).

HRMS (ESI): m/z calcd. for $C_{24}H_{15}O_3P$: 383.0837 $[M+H]^+$, found: 383.0828.

IR (ATF): v [cm^{-1}] = 1522 (m), 1446 (s), 1304 (w), 1254 (m), 1165 (w), 1107 (m), 1086 (br), 1007 (w), 856 (m), 737 (s).

4.24 Tri(benzofuran-7-yl)phosphine (L26)

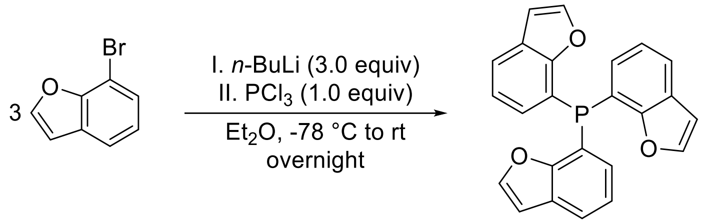

First, 7-bromobenzofuran (1.00 g, 5.07 mmol, 3 equiv.) was weighed into a 100 mL Schlenk flask under argon. Then, 10 mL of anhydrous diethyl ether were added, and the solution was cooled to -78 °C, followed by the slowly addition of *n*-BuLi (5.07 mmol, 3 equiv.). After the reaction mixture stirred for 2 h at -78 °C, phosphorus trichloride (0.23 g, 1.69 mmol, 1 equiv.) was added dropwise to the lithiated benzofuran solution. Then, the reaction mixture was allowed to warm to room temperature. The suspension was filtrated with a syringe filter and a colorless solution was received. Then, the solvent was removed under vacuum, yielding a white solid. Finally, crystals of **L26** were obtained from a concentrated DCM solution at -30 °C.

Chemical Formula: $C_{24}H_{15}O_3P$

Molecular Weight [g/mol]: 382.3548

Appearance: white solid

Isolated Yield: 62% (401 mg, 1.05 mmol)

^{1}H NMR (400 MHz, THF-*d$_8$*): δ = 7.67 (d, J=2.2, 3H), 7.61 (dd, J=7.7, 1.2, 3H), 7.08 (ddd, J=7.8, 7.4, 0.5, 3H), 6.83 (t, J=2.2, 3H), 6.80 - 6.74 (m, 3H).

^{13}C NMR (101 MHz, THF-*d$_8$*): δ = 157.98 (d, J=17.6), 146.17, 129.64 (d, J=3.6), 127.93 (d, J=2.8), 123.70 (d, J=1.5), 122.83, 118.52 (d, J=16.9), 106.96 (d, J=1.8).

^{31}P NMR (162 MHz, THF-*d$_8$*): δ = -44.82.

GC-MS: *m/z* (%): 383.11 ([M+H]$^+$, 26), 382.11 (M$^+$, 100), 381.12 ([M-H]$^+$, 12), 265.06 (4), 264.06 (9), 263.05 (19), 237.07 (6), 236.07 (16), 234.09 (12), 208.07 (5), 207.06 (18), 205.08 (7), 148.01 (30), 147.02 (12), 120.01 (5), 118.05 (5), 89.05 (6), 63.03 (6).

HRMS (ESI): *m/z* calcd. for $C_{24}H_{15}O_3P$: 405.0651 [M+Na]$^+$, found: 405.0649.

IR (ATF): v [cm^{-1}] = 1533 (w), 1406 (m), 1319 (w), 1255 (w), 1219 (m), 1167 (m), 1126 (m), 1028 (m), 949 (w), 793 (m), 727 (s), 621 (m).

4.25 Tri(benzofuran-5-yl)phosphine (L27)

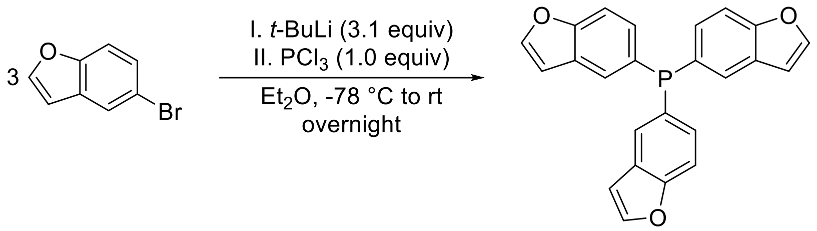

Under argon atmosphere, 5-bromobenzofuran (520 mg, 2.64 mmol, 3 equiv.) was dissolved in 15 mL of anhydrous diethyl ether in a 100 mL Schlenk flask. At -78 °C, *t*-BuLi (2.73 mmol, 3.1 equiv.) was slowly added to the solution, and the reaction mixture was kept for 1 h at this temperature. Then, phosphorus trichloride (121 mg, 0.88 mmol, 1 equiv.) was added dropwise to the lithiated benzofuran compound at -78 °C. After warming to room temperature, the white suspension was filtered through a syringe filter to remove lithium chloride, yielding a colorless solution. Next, the solvent was removed under reduced pressure and a white solid was received. Crystallization of the target phosphine was achieved by a concentrated DCM solution at -30 °C.

Chemical Formula: $C_{24}H_{15}O_3P$

Molecular Weight [g/mol]: 382.3548

Appearance: white solid

Isolated Yield: 53% (178 mg, 0.47 mmol)

^{1}H NMR (400 MHz, THF-*d*$_8$): δ = 7.74 (d, *J* = 2.2, 1H), 7.53 (dd, *J* = 7.3, 1.7, 1H), 7.51 - 7.45 (m, 1H), 7.33 - 7.24 (m, 1H), 6.76 (dd, *J* = 2.3, 1.0, 1H).

^{13}C NMR (101 MHz, THF-*d*$_8$): δ = 156.21, 146.51, 133.10 (d, *J*=11.7), 130.55 (d, *J*=24.1), 128.96 (d, *J*=8.1), 127.51 (d, *J*=20.6), 111.96 (d, *J*=8.4), 107.19.

^{31}P NMR (122 MHz, THF-*d*$_8$): δ = -3.79.

GC-MS: *m/z* (%): 383.09 ([M+H]$^+$, 26), 382.07 (M$^+$, 100), 381.08 ([M-H]$^+$, 11), 265.03 (10), 264.03 (6), 263.02 (27), 236.03 (8), 234.06 (8), 208.03 (6), 207.03 (23), 205.05 (7), 147.98 (36), 146.98 (15), 120.00 (5), 118.02 (4), 89.03 (8), 63.01 (8).

HRMS (ESI): m/z calcd. for $C_{24}H_{15}O_3P$: 383.0837 [M+H]$^+$, found: 383.0836.

IR (ATF): ν [cm^{-1}] = 1531 (w), 1446 (m), 1419 (w), 1306 (w), 1259 (m), 1242 (m), 1173 (w), 1109 (m), 1026 (s), 806 (m), 768 (s), 735 (m), 613 (m).

5 Ligand Screening: Furan- and Benzofuran-Phosphines

The synthesized furan- and benzofuran-phosphine ligands **L1-L27** have been tested for the isomerization of substrate **1a** with the same procedure as described for the initial screening of phosphine ligands (section 3). The obtained results from the ligand screening and optimizations are summarized in **Table S3**, **Table S4** and **Table S5**.

Table S3: Isomerization of **1a** using the furan-phosphine ligands **L1-L13**.

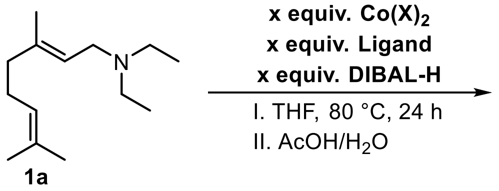

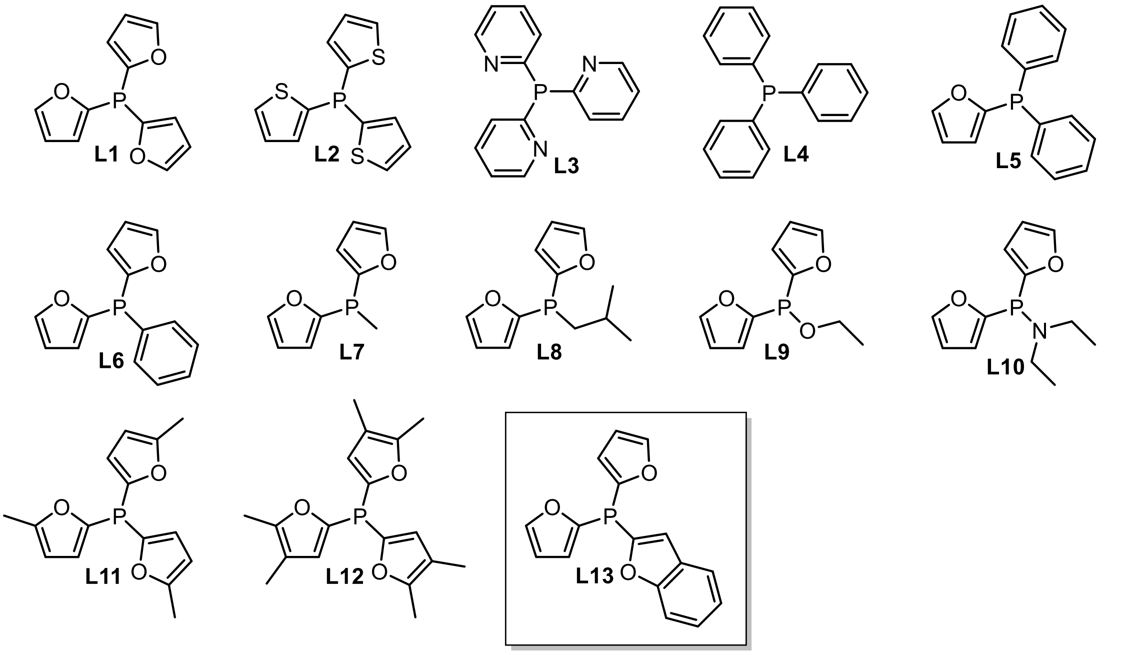

Entry	T [°C]	t [h]	Solvent	$Co(OAc)_2$ [mol%]	DIBAL-H [mol%]	Ligand [mol%]	Yield[a] [%]	Conversion [a] [%]
1	80	24	THF	2	6	L1 (6)	81	87
2	80	24	THF	2	6	L2 (6)	2	5
3	80	24	THF	2	6	L3 (6)	0	4
4	80	24	THF	2	6	L4 (6)	52	83
5	80	24	THF	2	6	L5 (6)	55	64

6	80	24	THF	2	6	L6 (6)	61	71
7	80	24	THF	2	6	L7 (6)	8	20
8	80	24	THF	2	6	L8 (6)	12	35
9	80	24	THF	2	6	L9 (6)	11	13
10	80	24	THF	2	6	L10 (6)	9	10
11	80	24	THF	2	6	L11 (6)	19	22
12	80	24	THF	2	6	L12 (6)	7	9
13	80	24	THF	2	6	L13 (6)	94	99
14	80	24	THF	1	3	L13 (3)	94	99
15	80	24	THF	0.5	1.5	L13 (1.5)	94	99
16	80	24	THF	0.5	1	L13 (1)	93	99
17	80	24	THF	0.2	0.4	L13 (0.4)	52	54

[a]General reaction conditions: Substrate **1a** (2.5 mmol), THF (1.5 mL), $Co(OAc)_2$ (8.9 mg, 0.05 mmol), Ligand (0.15 mmol), DIBAL-H (0.15 mmol), 80 °C, 24 h. The isolated yields and the substrate conversions were determined by GC using *n*-hexadecane as internal standard.

Table S4: Screening of benzofuran phosphines **L13-L16 & L19-L24.**

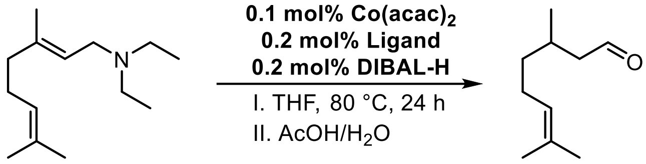

1a **1c**

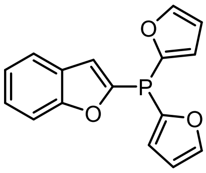

L13

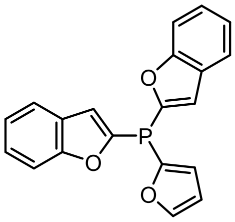

L14

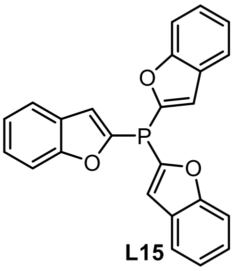

L15

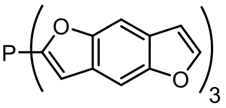

L16

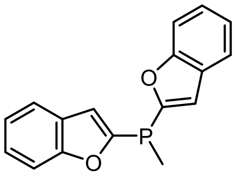

L19

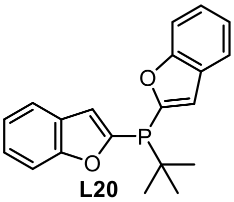

L20

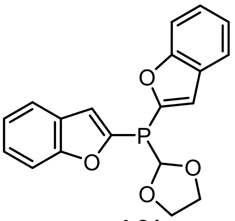

L21

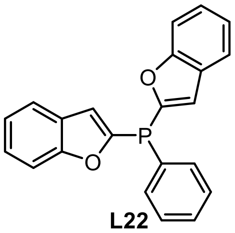

L22

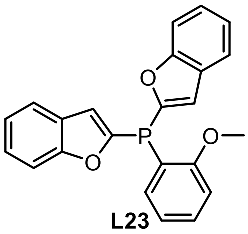

L23

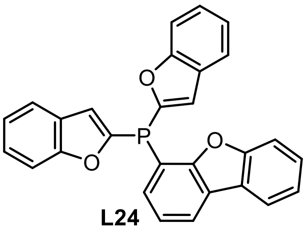

L24

Entry	T [°C]	t [h]	Solvent	$Co(acac)_2$ [mol%]	DIBAL-H [mol%]	Ligand [mol%]	Yield[a] [mol%]	Conversion[a] [mol%]
1	80	24	THF	0.1	0.2	L13 (0.2)	92	99
2	80	24	THF	0.08	0.16	L13 (0.16)	90	97
3	80	24	THF	0.1	0.2	L14 (0.2)	85	89
4	80	24	THF	0.1	0.2	L15 (0.2)	79	79
5	100	24	THF	0.1	0.2	L15 (0.2)	98	99
6	80	24	THF	0.1	0.2	L16 (0.2)	87	90
7	80	24	THF	0.1	0.2	L19 (0.2)	0	0
8	80	24	THF	0.1	0.2	L20 (0.2)	13	15
9	80	24	THF	0.1	0.2	L21 (0.2)	0	0

10	80	24	THF	0.1	0.2	L22 (0.2)	60	61
11	80	24	THF	0.1	0.2	L23 (0.2)	27	28
12	80	24	THF	0.1	0.2	L24 (0.2)	85	88

[a]General reaction conditions: Substrate **1a** (2.5 mmol), THF (1.5 mL), Co(acac)$_2$, Ligand, DIBAL-H, 80 °C, 24 h. The isolated yields and the substrate conversions were determined by GC using *n*-hexadecane as internal standard.

Table S5: Screening of benzofuran phosphines **L15/L17/L18 & L25-27**.

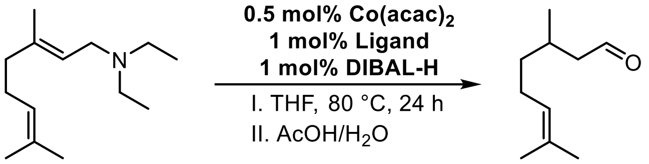

1a **1c**

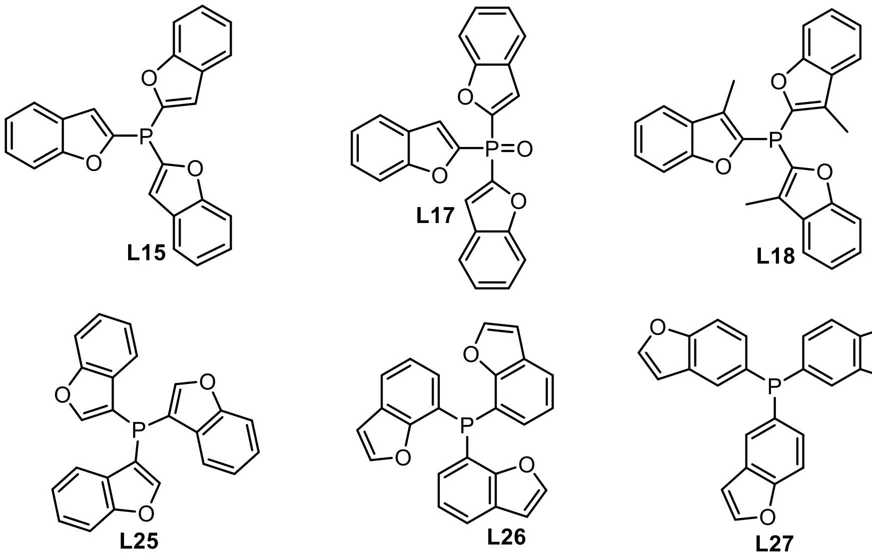

Entry	T [°C]	t [h]	Solvent	$Co(acac)_2$ [mol%]	DIBAL-H [mol%]	Ligand [mol%]	Yield[a] [mol%]	Conversion[a] [mol%]
1	80	24	THF	0.5	1	L15 (1)	98	99
2	80	24	THF	0.2	0.4	L15 (0.4)	98	99
3	80	24	THF	0.5	1	L17 (1)	3	4
4	80	24	THF	0.5	1	L18 (1)	13	14
5	80	24	THF	0.5	1	L25 (1)	37	39
6	80	24	THF	0.5	1	L26 (1)	20	21
7	80	24	THF	0.5	1	L27 (1)	17	19

[a]General reaction conditions: Substrate **1a** (2.5 mmol), THF (1.5 mL), $Co(acac)_2$, Ligand, DIBAL-H, 80 °C, 24 h. The isolated yields and the substrate conversions were determined by GC using *n*-hexadecane as internal standard.

6 Optimization of Reaction Conditions

The following chapter shows the optimization of the Co catalyst for the isomerization of substrate **1a** using the benzofuran phosphines **L13** and **L15**. The obtained results from the precursor, solvent and reducing agent screening as well as further optimizations are summarized in **Table S6 - Table S9**.

Table S6: Optimization of the reaction conditions using **L13**.

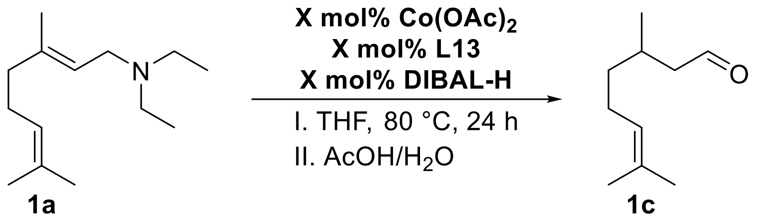

Entry	T [°C]	t [h]	Solvent	$Co(OAc)_2$ [mol%]	DIBAL-H [mol%]	L13 [mol%]	Yield[a] [mol%]	Conversion[a] [mol%]
1	80	24	THF	2	6	6	94	99
2	80	24	THF	1	3	3	94	99
3	80	24	THF	0.5	1.5	1.5	94	99
4	80	24	THF	0.5	1	1	93	99
5	80	24	THF	0.2	0.4	0.4	52	54

[a]General reaction conditions: Substrate **1a** (2.5 mmol), THF (1.5 mL), $Co(OAc)_2$, **L13**, DIBAL-H, 80 °C, 24 h. The isolated yields and the substrate conversions were determined by GC using *n*-hexadecane as internal standard.

Table S7: Optimization ligand/metal/reductant ratio using **L15**.

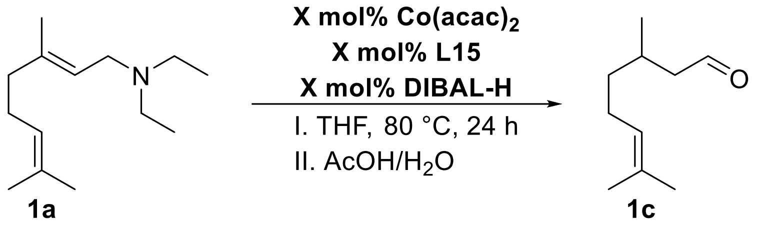

Entry	T [°C]	t [h]	Solvent	**Co(acac)$_2$ [mol%]**	**DIBAL-H [mol%]**	**L15 [mol%]**	Yield[a] [mol%]	Conversion[a] [mol%]
1	80	24	THF	**0.5**	**1.0**	**1.0**	98	99
2	80	24	THF	**0.5**	**0.5**	**1.0**	99	99
3	80	24	THF	**0.5**	**0.5**	**0.5**	98	98
4	80	24	THF	**0.4**	**0.4**	**0.4**	97	98
5	80	24	THF	**0.3**	**0.3**	**0.3**	90	90
6	80	24	THF	**0.2**	**0.2**	**0.2**	67	69
7	80	24	THF	**0.2**	**0.4**	**0.4**	98	99
8	80	24	THF	**0.1**	**0.2**	**0.2**	79	79
9	100	24	THF	**0.1**	**0.2**	**0.2**	98	99

[a]General reaction conditions: Substrate **1a** (2.5 mmol), THF (1.5 mL), Co(acac)$_2$, **L15**, DIBAL-H, 80 °C, 24 h. The isolated yields and the substrate conversions were determined by GC using *n*-hexadecane as internal standard.

Table S8: Varying the Co-precursor for the isomerization of **1a**.

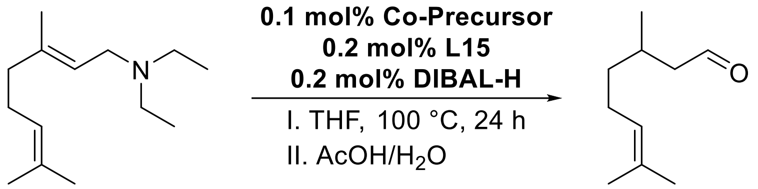

1a → **1c**

Entry	T [°C]	t [h]	Solvent	**Precursor [mol%]**	Yield[a] [mol%]	Conversion[a] [mol%]
1	100	24	THF	**$Co(OAc)_2$**	30	32
2	100	24	THF	**$CoCl_2$**	0	0
3	100	24	THF	**$CoBr_2$**	0	3
4	100	24	THF	**$CoOTf_2$**	0	2
5	100	24	THF	**$Co(BF_4)_2$**	0	0
6	100	24	THF	**$Co(C_6H_5COO)_2$**	0	2
7	100	24	THF	**$Co(acac)_2$**	98	99
8[b]	100	24	THF	**$Co(acac)_3$**	97	99

[a]General reaction conditions: Substrate **1a** (2.5 mmol), THF (1.5 mL), 0.1 mol% Co-precursor, 0.2 mol% DIBAL-H, 0.2 mol% **L15**, 100 °C, 24 h. [b]0.3 mol% DIBAL-H. The isolated yields and the substrate conversions were determined by GC using *n*-hexadecane as internal standard.

Table S9: Screening of reductants for the Co-catalyzed isomerization of **1a**.

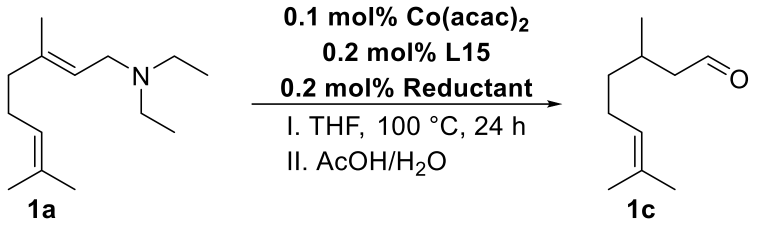

Entry	T [°C]	t [h]	Solvent	**Reductant [mol%]**	Yield[a] [mol%]	Conversion[a] [mol%]
1	100	24	THF	**NaH**	0	2
2	100	24	THF	**KH**	0	4
3	100	24	THF	**$NaHBEt_3$**	0	3
4[b]	100	24	THF	**Zn**	0	2
5[b]	100	24	THF	**NH_3BH_3**	0	0
6	100	24	THF	**MeMgBr**	0	3
7	100	24	THF	**$LiAlH_4$**	16	21
8	100	24	THF	**$LiAlH[OC(CH_3)_3]_3$**	38	42
9	100	24	THF	**$AlEt_3$**	96	99
10	100	24	THF	**DIBAL-H**	98	99

[a]General reaction conditions: Substrate **1a** (2.5 mmol), THF (1.5 mL), 0.1 mol% $Co(acac)_2$, 0.2 mol% reductant, 0.2 mol% **L15**, 100 °C, 24 h. [b]0.5 mol% reductant. The isolated yields and the substrate conversions were determined by GC using *n*-hexadecane as internal standard.

7 Identification of Side-Products - Isomerization of 1a

To isolate the side-products of the Co-catalyzed isomerization of **1a**, scale-up experiments were performed using the initial developed **Co/L1/DIBAL-H** catalyst. These experiments were conducted on a molar scale to obtain higher concentrations of the side reaction products for isolation. The majority of the side-products were isolated by distillation, with additional purification via column chromatography when required.

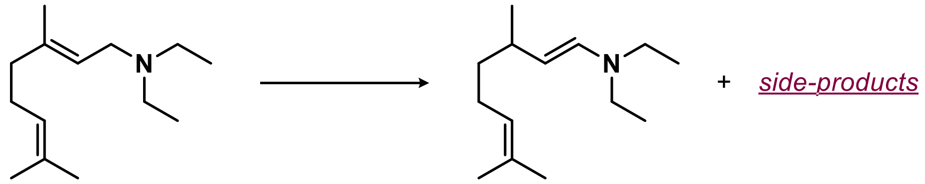

1a Starting material → **1b** Target product

The isolated isomers of **1a** were analyzed and characterized by NMR spectroscopy. In total, five internal-olefine isomers (**1ba-1be**) and four enamine isomers (**1bf-1bi**) were isolated, as demonstrated in **Figure S2**.

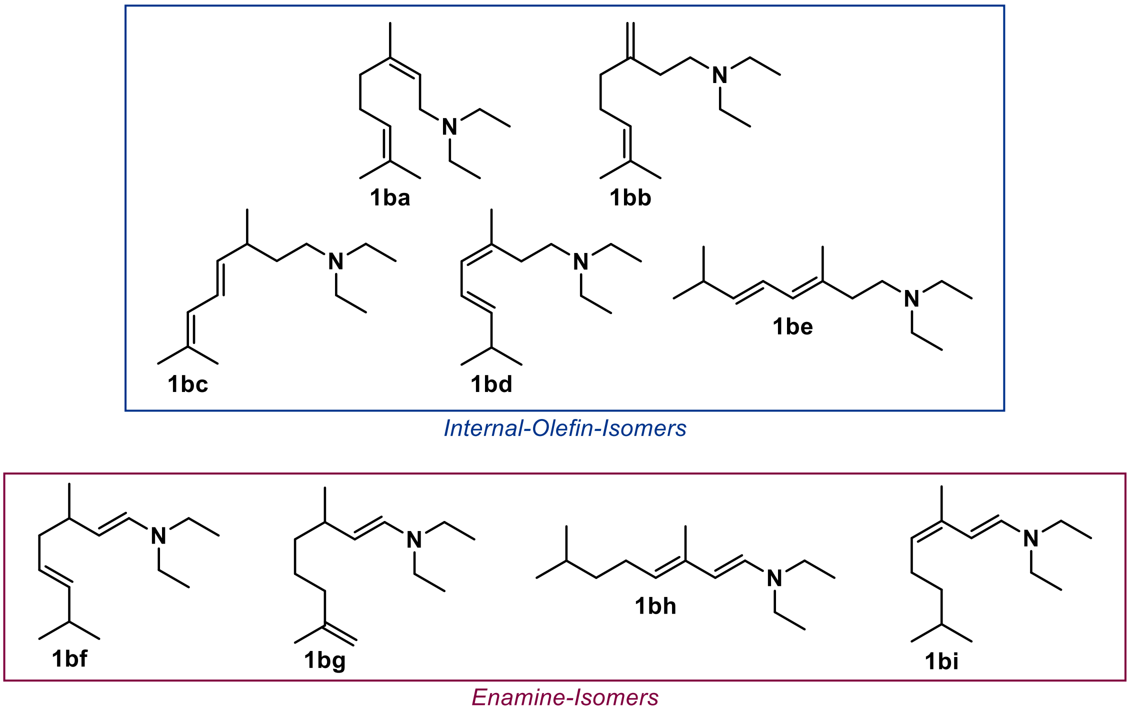

Figure S2: Isolated side-products from the Co-catalyzed isomerization of **1a**.

Isomer: (*E*)-*N,N*-diethyl-3,7-dimethylocta-2,6-dien-1-amine (1a)

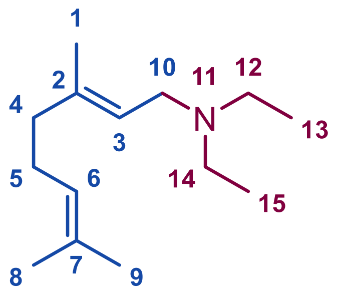

^{1}H NMR (600 MHz, C_6D_6): δ = 5.48 (tq, *J* = 6.7, 1.3 Hz, 1H, **3**), 5.20 (tdt, *J* = 7.0, 2.9, 1.4 Hz, 1H, **6**), 3.09 (dq, *J* = 6.7, 0.9 Hz, 2H, **10**), 2.48 (q, *J* = 7.1 Hz, 4H, **12/14**), 2.20 - 2.12 (m, 2H, **5**), 2.08 (ddt, *J* = 8.0, 7.1, 1.1 Hz, 2H, **4**), 1.66 (q, *J* = 1.3 Hz, 3H, **9**), 1.61 (q, *J* = 1.0 Hz, 3H, **1**), 1.54 (d, *J* = 1.3 Hz, 3H, **8**), 1.02 (t, *J* = 7.1 Hz, 6H, **13/15**).

^{13}C NMR (151 MHz, C_6D_6): δ = 137.03 (-$C_{quart.}$, **2**), 131.24 (-$C_{quart.}$, **7**), 124.85 (-CH, **6**), 123.58 (-CH_2, **3**), 51.22 (-CH_2, **10**), 47.19 (-CH_2, **12/14**), 40.25 (-CH_2, **4**), 26.95 (-CH_2, **5**), 25.88 (-CH_3, **9**), 17.74 (-CH_3, **8**), 16.39 (-CH_3, **1**), 12.58 (-CH_3, **13/15**).

HH-COSY NMR (600 MHz, C_6D_6): δ = 5.48 (1H, **3**) and 3.08 (2H, **10**); 5.21 (1H, **6**) and 2.15 (2H, **5**); 3.07 (2H, **10**) and 1.61 (3H, **1**); 2.47 (4H, **12/14**) and 1.01 (6H, **13/15**).

HSQC NMR (600 and 151 MHz, C_6D_6): δ = 5.47 (1H, **3**) and 123.27 (-CH, **3**); 5.18 (1H, **6**) and 124.43 (-CH, **6**); 3.08 (2H, **10**) and 50.63 (-CH_2, **10**); 2.47 (4H, **12/14**) and 46.68 (-CH_2, **12/14**); 2.16 (2H, **5**) and 26.42 (-CH_2, **5**); 2.08 (2H, **4**) and 39.69 (-CH_2, **4**); 1.65 (3H, **9**) and 25.26 (-CH_3, **9**); 1.60 (3H, **1**) and 15.71 (-CH_3, **1**); 1.53 (3H, **8**) and 17.11 (-CH_3, **8**); 1.01 (6H, **13/15**) and 11.99 (-CH_3, **13/15**).

Isomer **1a**: ^{1}H NMR and ^{13}C NMR.[2]

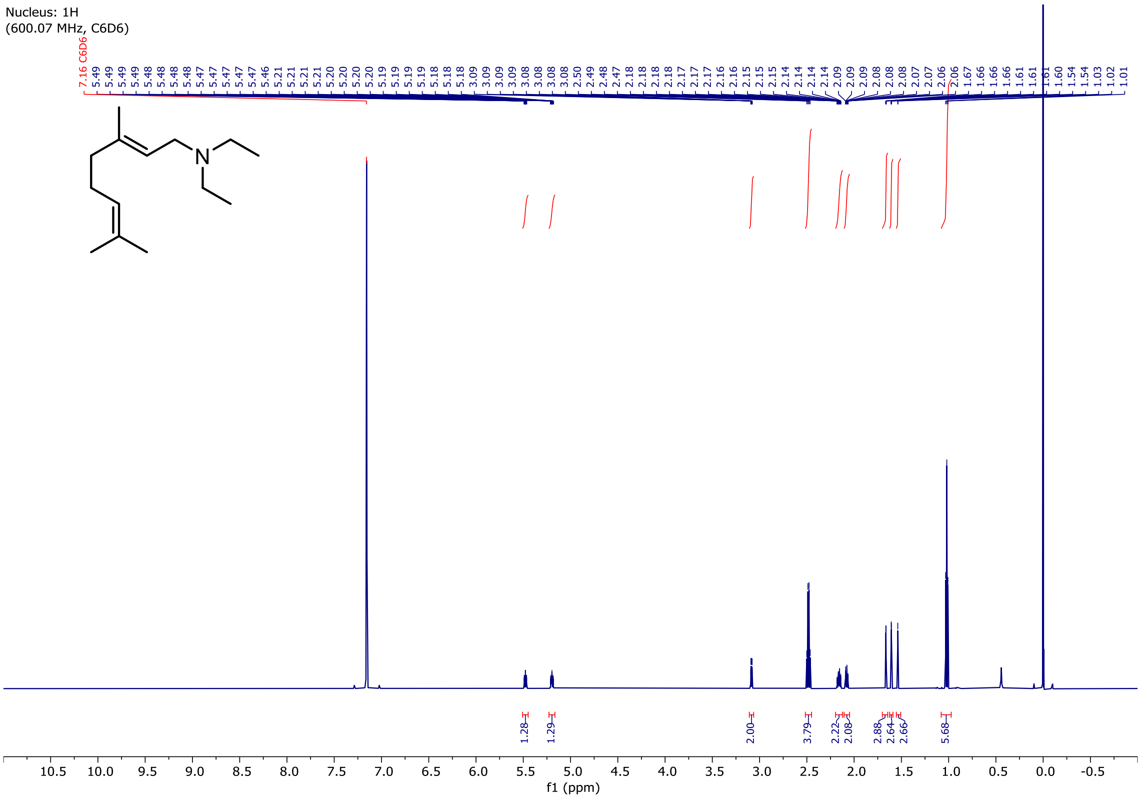

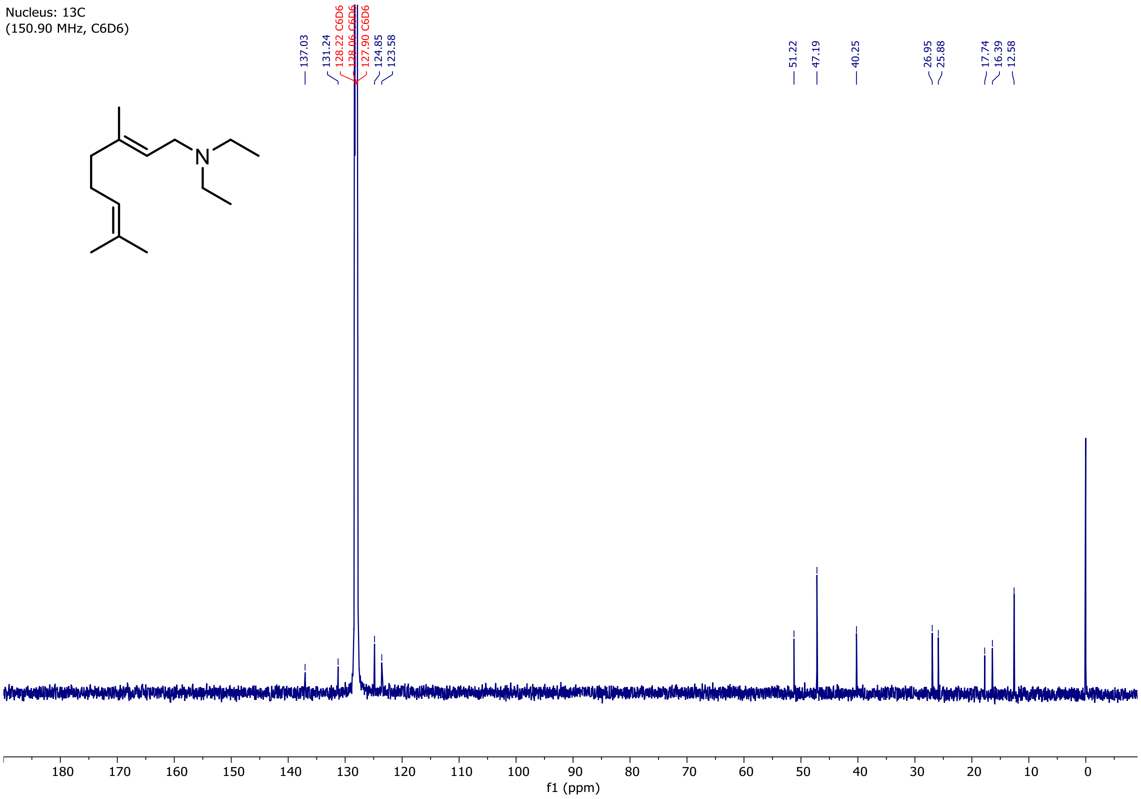

[2]Tetramethylsilane (TMS) was used as a reference.

Isomer **1a**: HH-COSY and HSQC NMR.

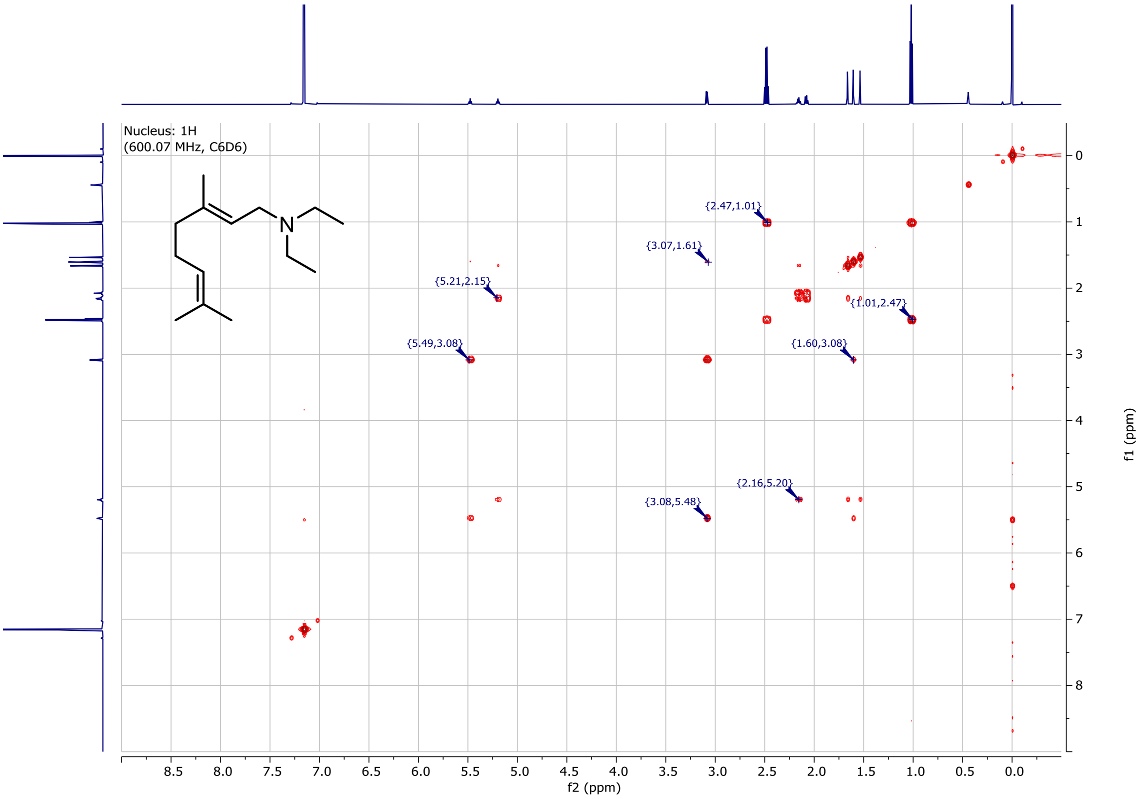

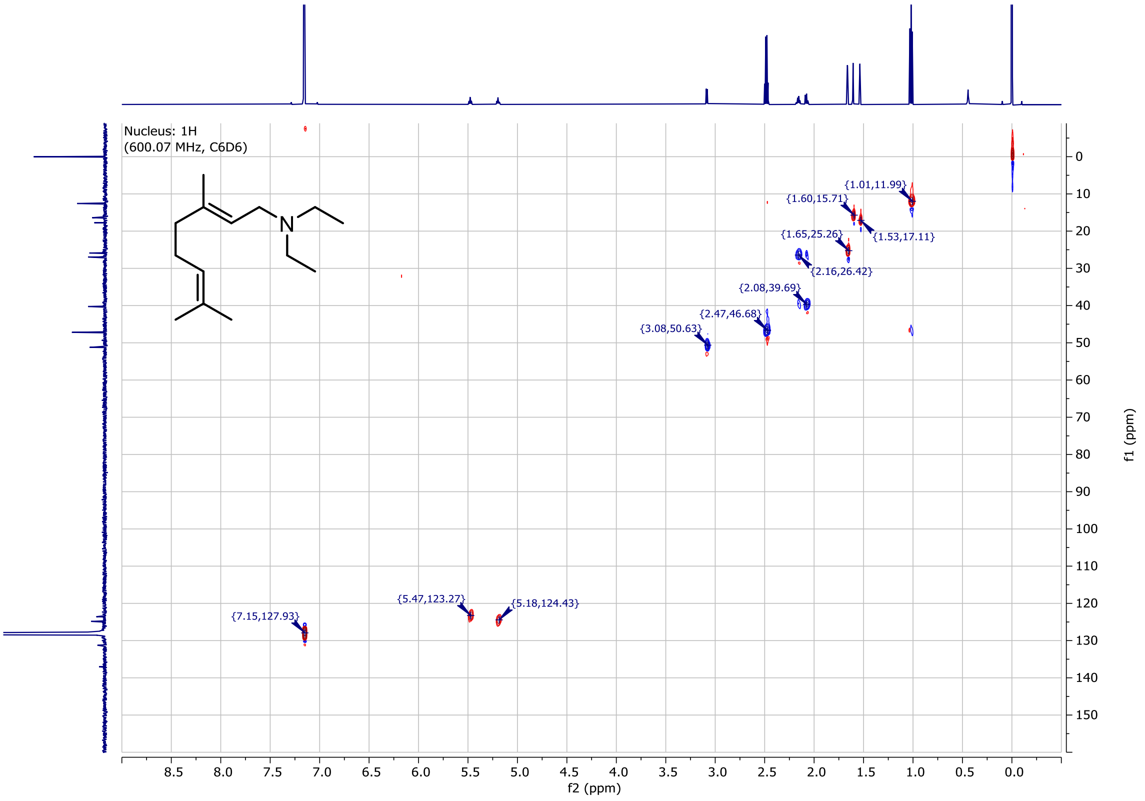

Isomer: (*E*)-*N,N*-diethyl-3,7-dimethylocta-1,6-dien-1-amine (1b)

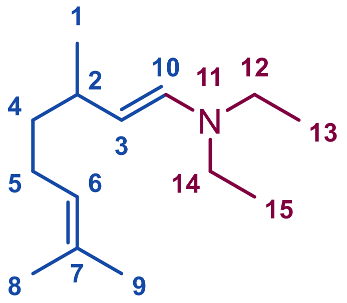

^{1}H NMR (300 MHz, C_6D_6): δ = 5.84 (dd, *J* = 13.8, 0.8 Hz, 1H, **10**), 5.31 (ddp, *J* = 7.2, 5.7, 1.4 Hz, 1H, **6**), 4.11 (dd, *J* = 13.8, 8.3 Hz, 1H, **3**), 2.75 (q, *J* = 7.1 Hz, 4H, **12/14**), 2.29 - 2.05 (m, 3H, **2/5**), 1.74 - 1.67 (m, 3H, **9**), 1.67 - 1.58 (m, 3H, **8**), 1.57 - 1.36 (m, 2H, **4**), 1.16 (d, *J* = 6.8 Hz, 3H, **1**), 0.90 (t, *J* = 7.1 Hz, 6H, **13/15**).

^{13}C NMR (75 MHz, C_6D_6): δ = 136.45 (-CH, **10**), 130.62 ($C_{quart.}$, **7**), 125.94 (-CH, **6**), 104.11 (-CH, **3**), 44.78 (-CH_2, **12/14**), 39.59 (-CH, **2**), 35.82 (-CH_2, **4**), 26.78 (-CH_2, **5**), 25.97 (-CH_3, **9**), 23.56 (-CH3, **1**), 17.85 (-CH_3, **8**), 12.57 (-CH_3, **13/15**).

^{135}DEPT NMR (75 MHz, C_6D_6): δ = 136.44 (neg., -CH, **10**), 125.94 (neg., -CH, **6**), 104.11 (neg., -CH, **3**), 44.78 (pos., -CH_2, **12/14**), 39.59 (pos., -CH, **2**), 35.82 (neg., -CH_2, **4**), 26.77 (pos., -CH_2, **5**), 25.96 (neg., -CH_3, **9**), 23.56 (neg., -CH_3, **1**), 17.85 (neg., -CH_3, **8**), 12.57 (neg., -CH_3, **13/15**).

Isomer **1b**: ^{1}H NMR and ^{13}C NMR.

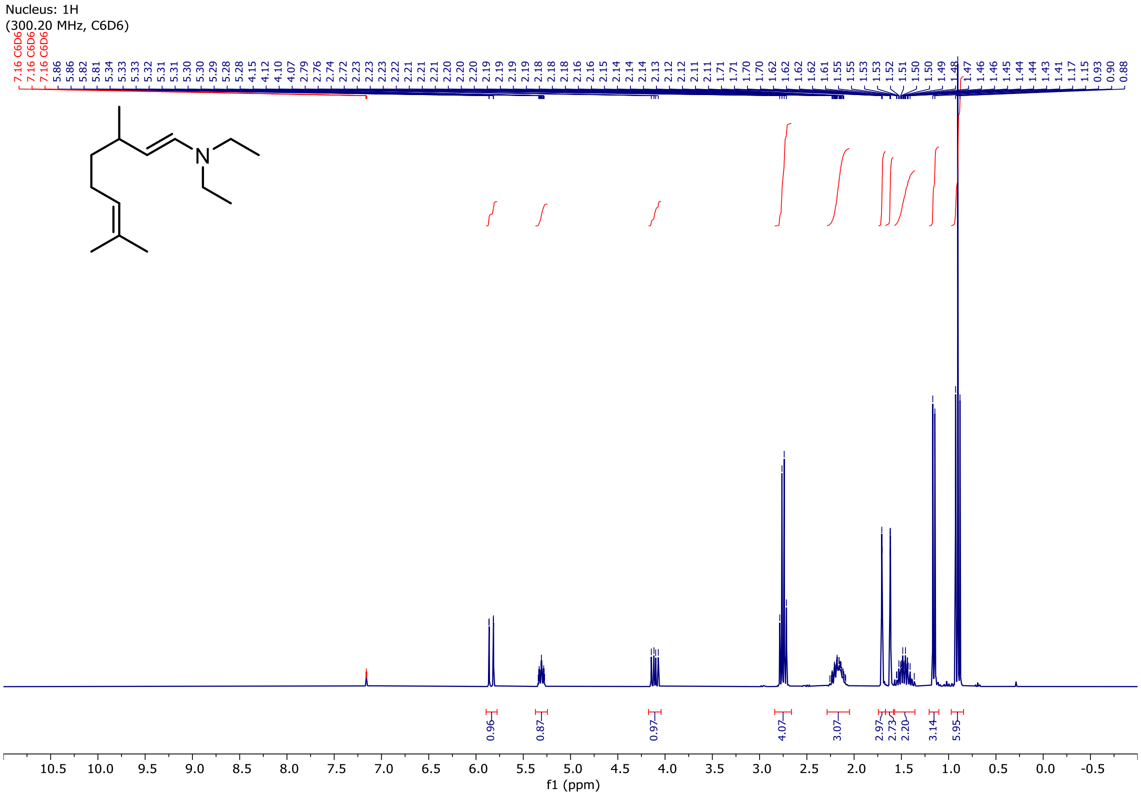

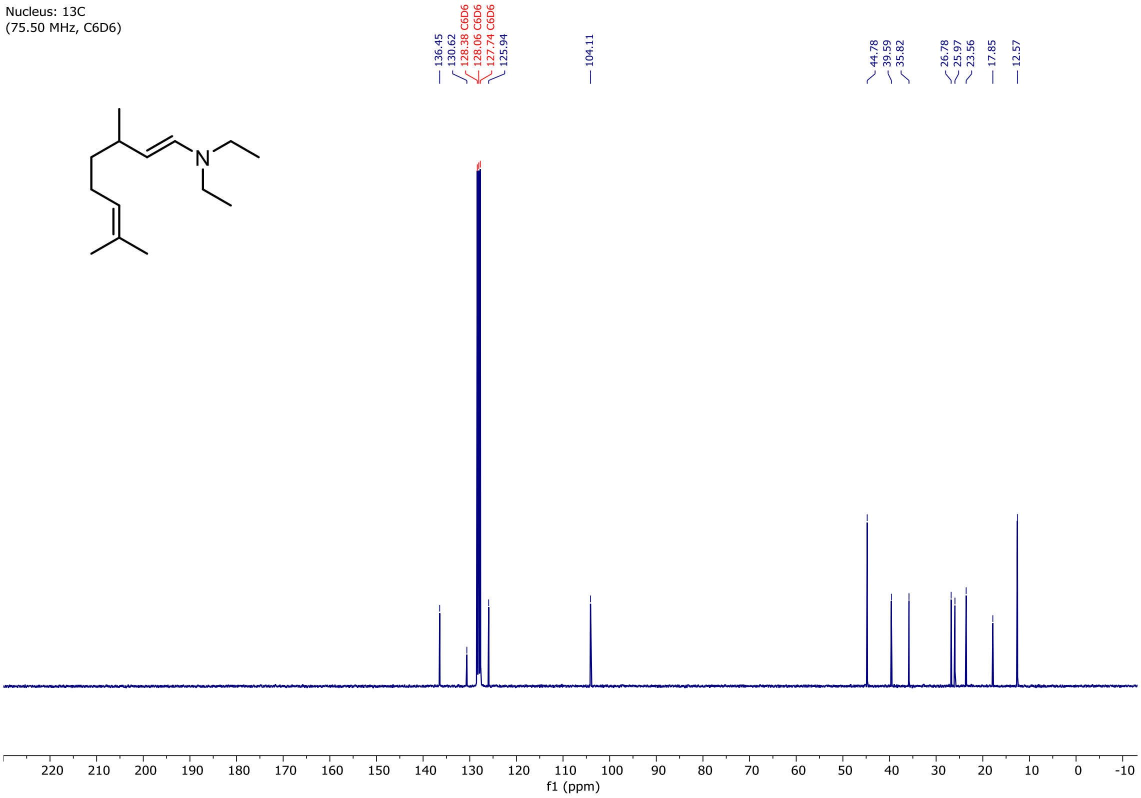

Isomer **1b**: ^{135}DEPT NMR.

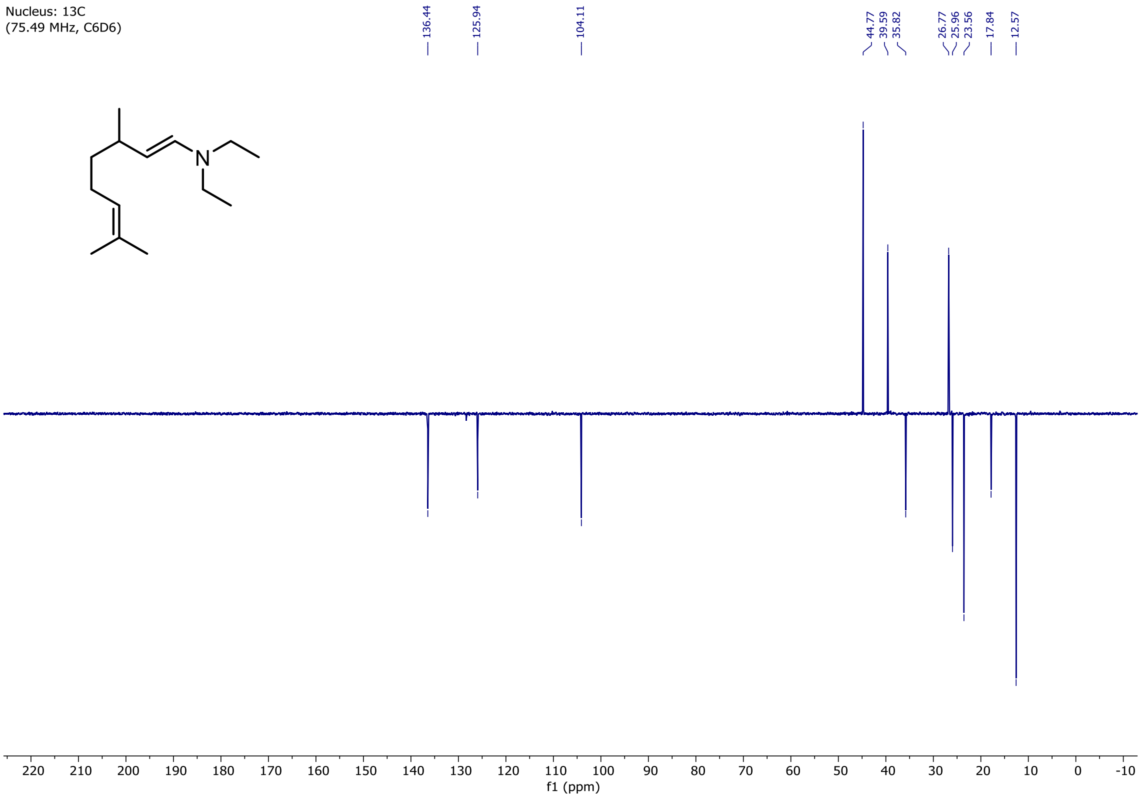

Isomer: (*Z*)-*N,N*-diethyl-3,7-dimethylocta-2,6-dien-1-amine (1ba)

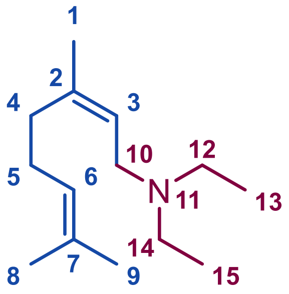

^{1}H NMR (600 MHz, C_6D_6): δ = 5.47 (td, *J* = 6.6, 1.5 Hz, 1H, **3**), 5.25 - 5.21 (m, 1H, **6**), 3.12 (dt, *J* = 6.7, 1.3 Hz, 2H, **10**), 2.48 (q, *J* = 7.1 Hz, 4H, **12/14**), 2.16 - 2.10 (m, 4H, **4/5**), 1.70 (q, *J* = 1.3 Hz, 3H, **1**), 1.68 - 1.66 (m, 3H, **9**), 1.58 - 1.56 (m, 3H, **8**), 1.02 (t, *J* = 7.1 Hz, 6H, **13/15**).

^{13}C NMR (151 MHz, C_6D_6): δ = 137.32 (-$C_{quart.}$, **2**), 131.45 (-$C_{quart.}$, **7**), 124.84 (**6**), 124.50 (**3**), 51.12 (**10**), 47.22 (**12/14**), 32.62 (**4**), 27.11 (**5**), 25.88 (**9**), 23.70 (**1**), 17.71 (**8**), 12.59 (**13/15**).

HSQC NMR (600 and 151 MHz, C_6D_6): δ = 5.46 (1H, **3**) and 124.55 (-CH, **3**); 5.22 (1H, **6**) and 124.78 (-CH, **6**); 3.12 (2H, **10**) and 50.98 (-CH_2, **10**); 2.48 (4H, **12/14**) and 47.02 (-CH_2, **12/14**); 2.13 (2H, **4**) and 32.26 (-CH_2, **4**); 2.13 (2H, **5**) and 27.00 (-CH_2, **5**); 1.70 (3H, **1**) and 23.51 (-CH_3, **1**), 1.67 (3H, **9**) and 25.61 (-CH_3, **9**), 1.56 (3H, **8**) and 17.46 (-CH_3, **8**), 1.01 (6H, **13/15**) and 12.34 (-CH_3, **13/15**).

Isomer **1ba**: ^{1}H NMR and ^{13}C NMR.[3]

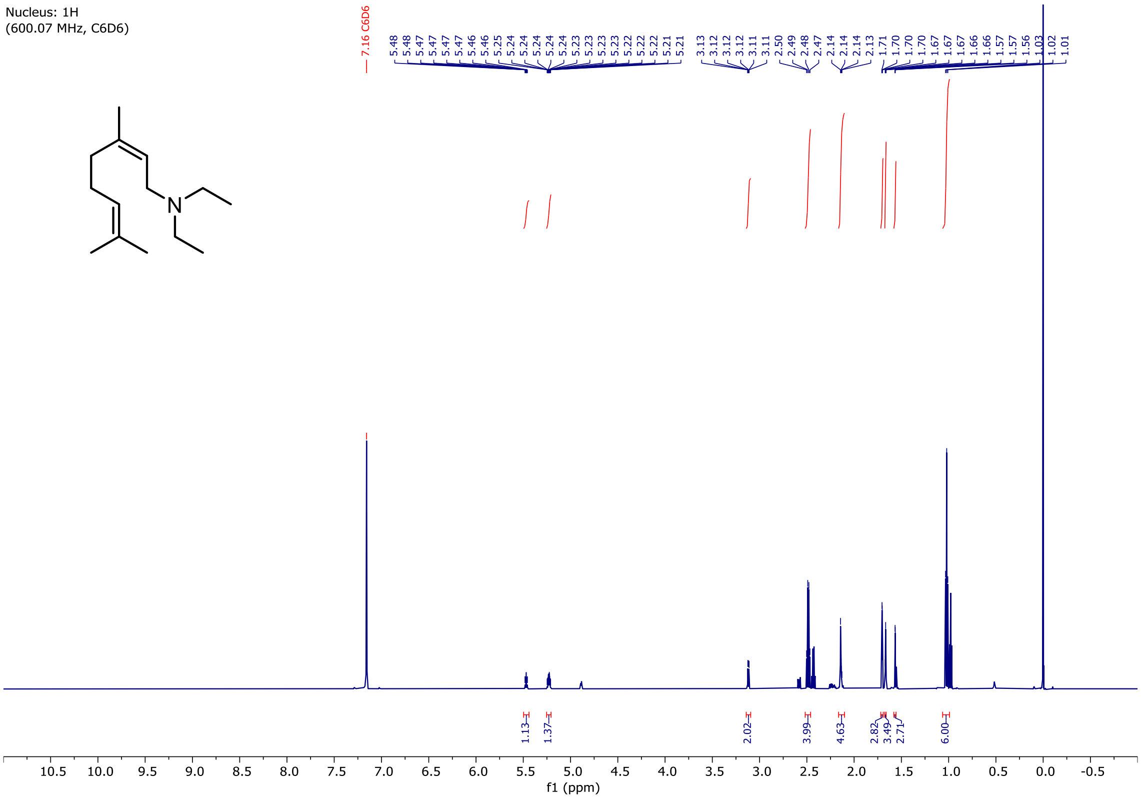

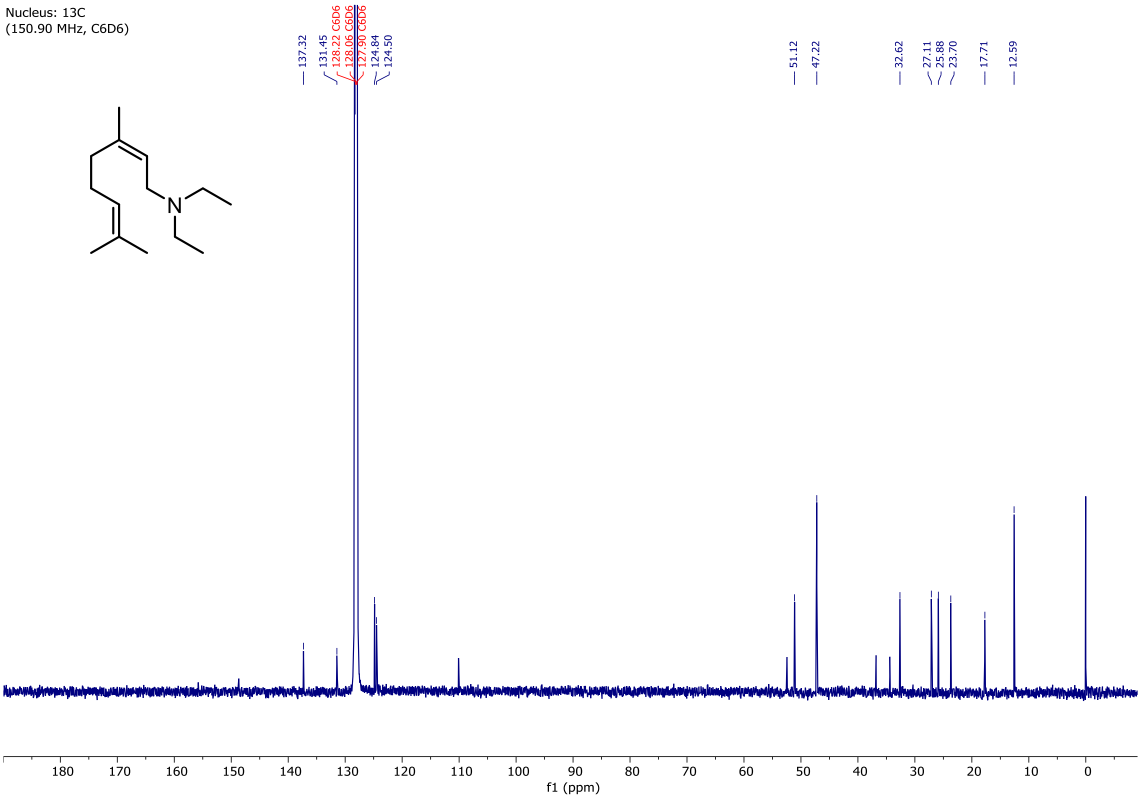

[3]Tetramethylsilane (TMS) was used as a reference.

Isomer **1ba**: HSQC NMR.

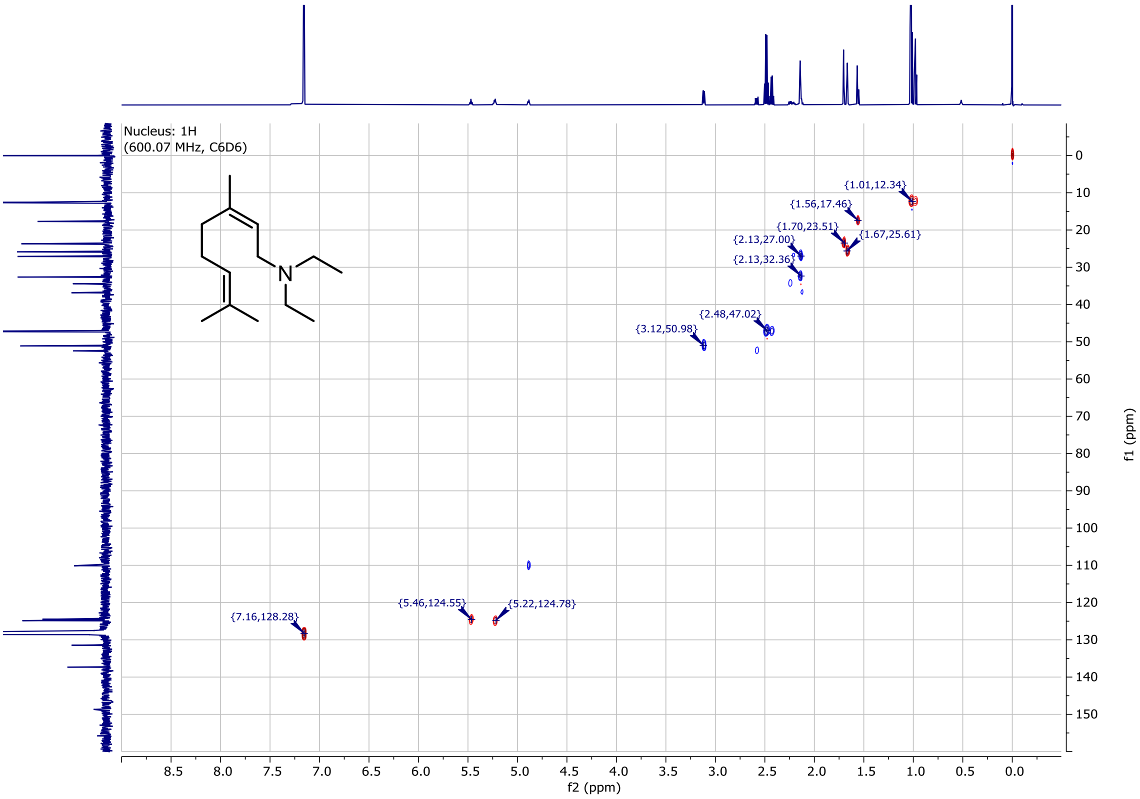

Isomer: (*E*)-*N,N*-diethyl-3,7-dimethylocta-4,6-dien-1-amine (1bb)

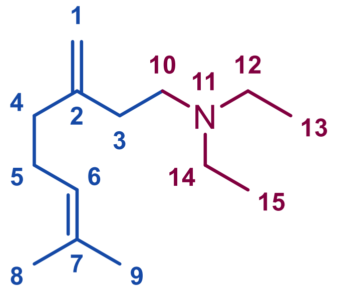

^{1}H NMR (600 MHz, C_6D_6): δ = 5.24 (ddt, *J* = 8.4, 5.6, 1.4 Hz, 1H, **6**), 4.94 - 4.83 (m, 2H, **1**), 2.62 - 2.55 (m, 2H, **10**), 2.43 (q, *J* = 7.1 Hz, 4H, **12/14**), 2.29 - 2.18 (m, 4H, **3/5**), 2.17 - 2.10 (m, 2H, **4**), 1.67 (d, *J* = 1.4 Hz, 3H, **9**), 1.58 - 1.52 (m, 3H, **8**), 0.98 (t, *J* = 7.1 Hz, 5H, **13/15**).

^{13}C NMR (151 MHz, C_6D_6): δ = 148.67 (**2**), 131.37 (**7**), 124.83 (**6**), 110.11 (**1**), 52.45 (**10**), 47.32 (**12/14**), 36.81 (**4**), 34.39 (**3**), 26.99 (**5**), 25.85 (**9**), 17.75 (**8**), 12.52 (**13/15**).

HSQC NMR (600 and 151 MHz, C_6D_6): δ = 5.23 (1H, **6**) and 124.78 (-CH, **6**); 4.89 (2H, **1**) and 110.12 (-CH_2,**1**); 2.59 (2H, **10**) and 52.38 (-CH_2, **10**); 2.42 (4H, **12/14**) and 47.02 (-CH_2, **12/14**); 2.25 (4H, **3/5**) and 36.78 (-CH_2, **3/5**); 2.22 (-CH_2, **3**) and 34.22 (-CH_2, **3**); 2.14 (2H, **5**) and 26.77 (-CH_2, **5**); 1.67 (3H, **9**) and 25.61 (-CH_3, **9**); 1.55 (3H, **8**) and 17.46 (-CH_3, **8**); 0.97 (3H, **13/15**) and 12.34 (-CH_3, **13/15**).

Isomer **1bb**: ^{1}H NMR and ^{13}C NMR.[4]

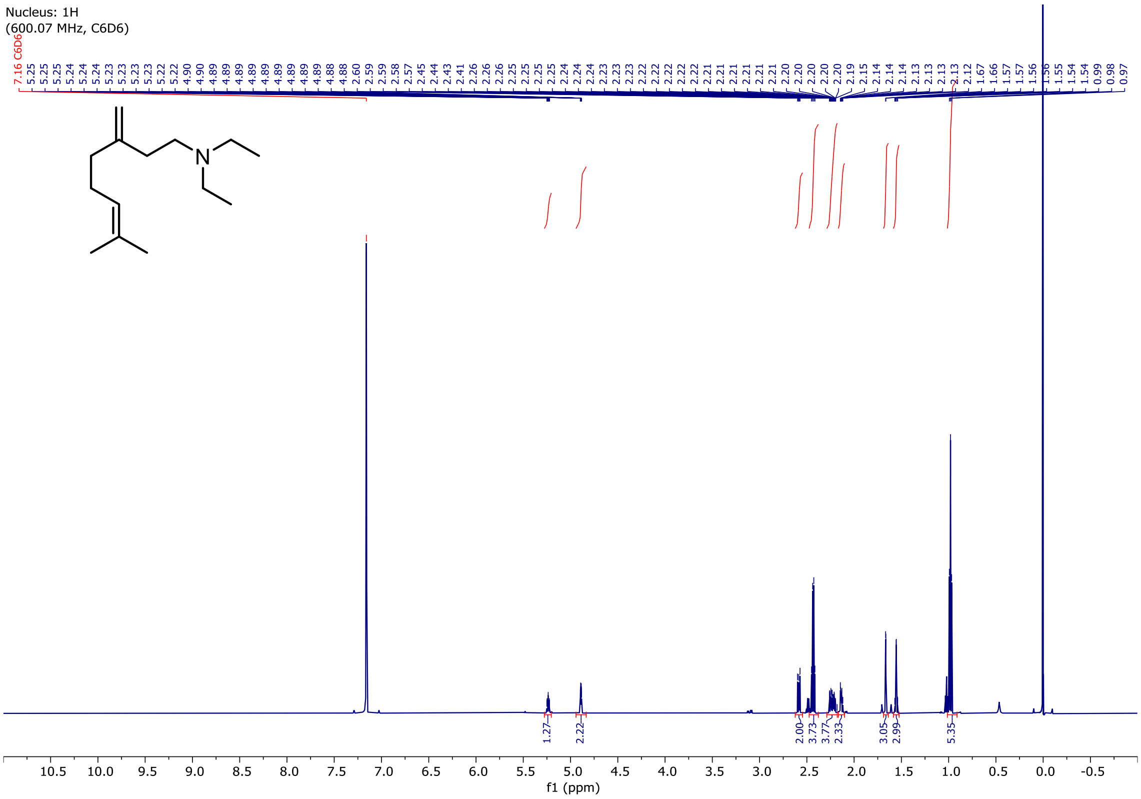

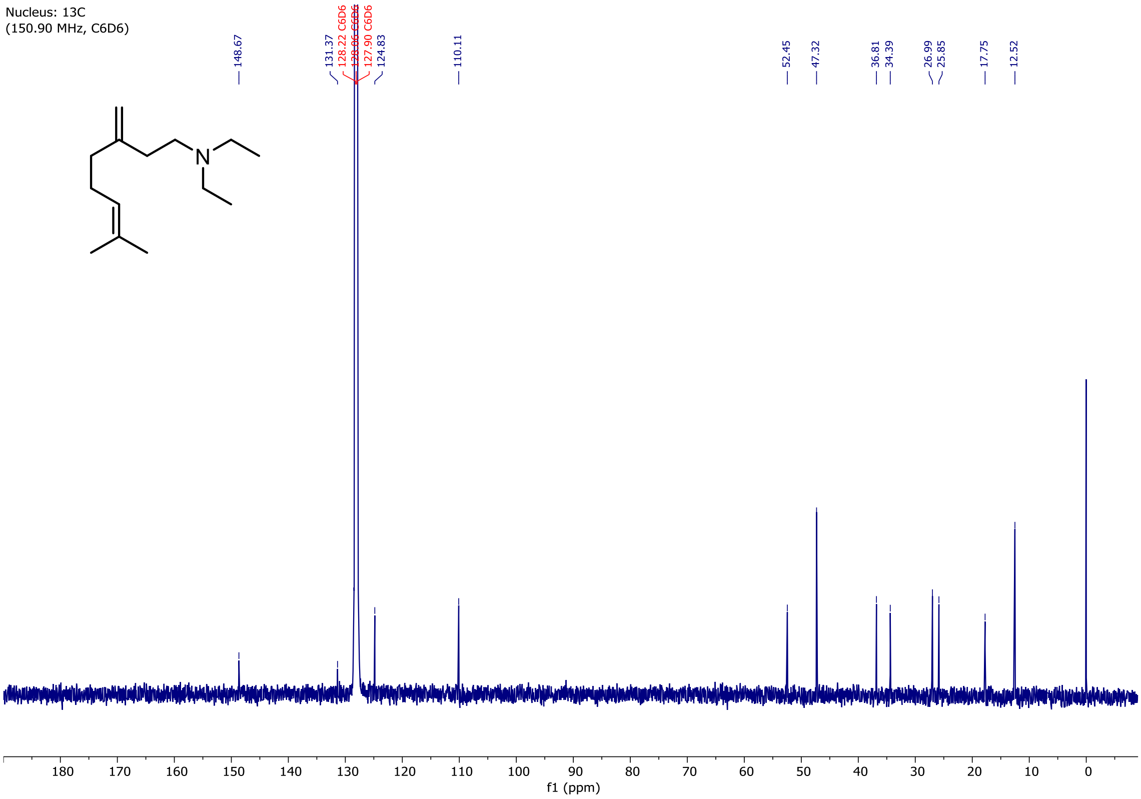

[4]Tetramethylsilane (TMS) was used as a reference.

Isomer **1bb**: HSQC NMR.

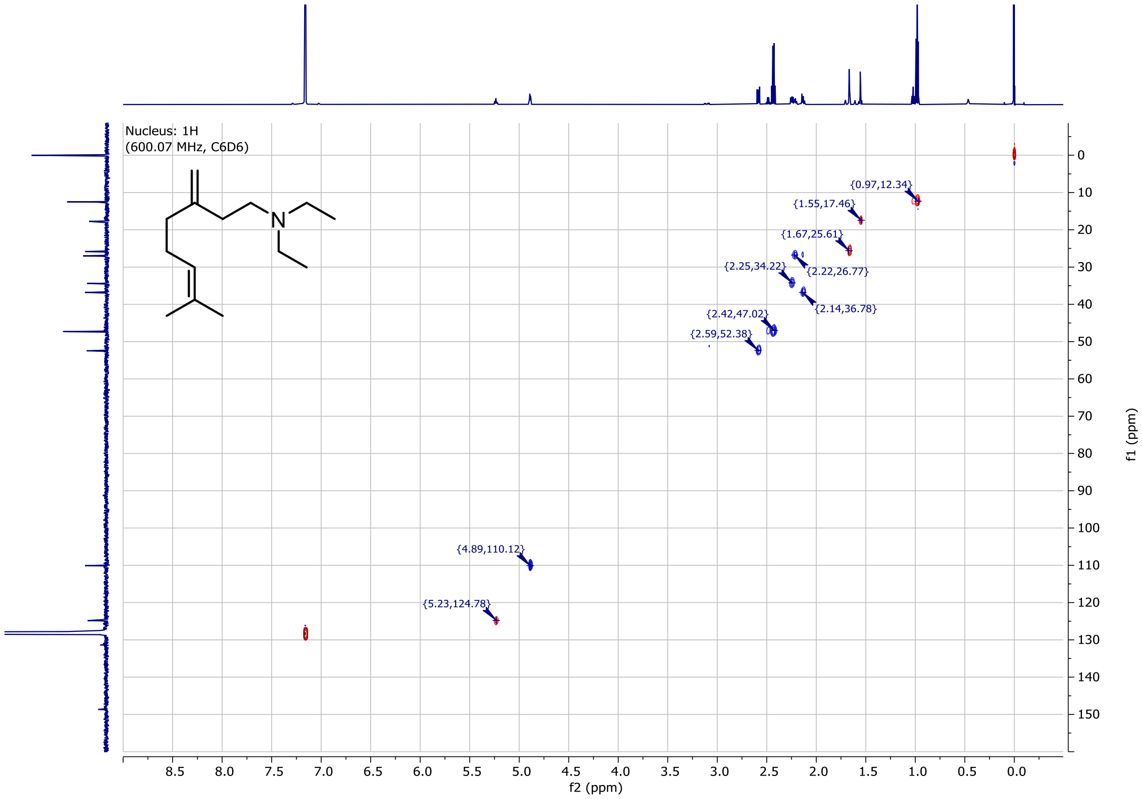

Isomer: (*E*)-*N,N*-diethyl-3,7-dimethylocta-4,6-dien-1-amine (1bc)

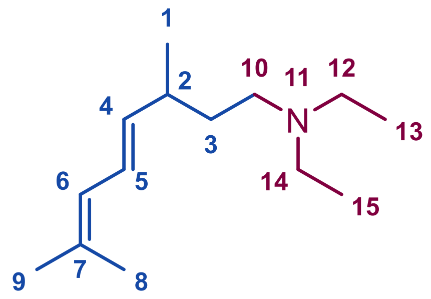

^{1}H NMR (600 MHz, C_6D_6): δ = 6.38 (ddd, *J* = 15.0, 10.8, 1.0 Hz, 1H, **5**), 5.93 (ddd, *J* = 10.8, 2.4, 1.3 Hz, 1H, **6**), 5.49 (dd, *J* = 15.1, 8.1 Hz, 1H, **4**), 2.51 - 2.36 (m, 6H, **10/12/14**), 2.33 (dt, *J* = 14.2, 7.1 Hz, 1H, **2**), 1.69 - 1.65 (m, 3H, **8**), 1.63 (d, *J* = 1.3 Hz, 3H, **9**), 1.54 - 1.45 (m, 2H, **3**), 1.05 (d, *J* = 6.7 Hz, 3H, **1**), 0.98 (t, *J* = 7.1 Hz, 6H, **13/15**).

^{13}C NMR (151 MHz, C_6D_6): δ = 138.07 (-CH, **4**), 132.15 (-$C_{quart.}$, **7**), 126.21 (-CH, **6**), 125.84 (-CH, **5**), 51.40 (-CH_2, **10**), 47.40 (-CH_2, **12/14**), 35.57 (-CH, **2**), 35.25 (-CH_2, **3**), 25.97 (-CH_3, **8**), 21.41 (-CH_3, **1**), 18.20 (-CH_3, **9**), 12.50 (-CH_3, **13/15**).

HH-COSY NMR (600 MHz, C_6D_6): δ = 6.38 (1H, **5**) and 5.94 (1H, **4**); 6.38 (1H, **5**) and 5.49 (1H, **6**); 5.93 (1H, **6**) and 1.66 (3H, **8**); 5.50 (1H, **4**) and 2.33 (1H, **2**); 2.42 (6H, **10/12/14**) and 1.50 (2H, **3**); 2.41 (6H, **10/12/14**) and 0.99 (6H, **13/15**); 2.32 (1H, **2**) and 1.05 (3H, **1**).

HSQC NMR (600 and 151 MHz, C_6D_6): δ = 6.38 (1H, **5**) and 125.73 (-CH, **5**); 5.94 (1H, **4**) and 125.96 (-CH, **5**); 5.49 (1H, **4**) and 138.08 (-CH, **4**); 2.43 (6H, **10/12/14**) and 50.96 (-CH_2, **10**); 2.43 (6H, **10/12/14**) and 47.00 (-CH_2, **12/14**); 2.32 (1H, **2**) and 35.12 (-CH_2, **3**); 1.66 (m, 3H, **8**) and 25.57 (-CH_3, **8**), 1.62 (m, 3H, **9**) and 17.65 (-CH_3, **9**), 1.49 (2H, **3**) and 34.88 (-CH_2, **3**); 1.05 (3H, **1**) and 20.91 (-CH_3, **1**); 1.00 (6H, **13/15**) and 47.10 (-CH_2, **12/14**); 0.98 (6H, **13/15**) and 12.06 (-CH_3, **13/15**).

Isomer **1bc**: ^{1}H NMR and ^{13}C NMR.[5]

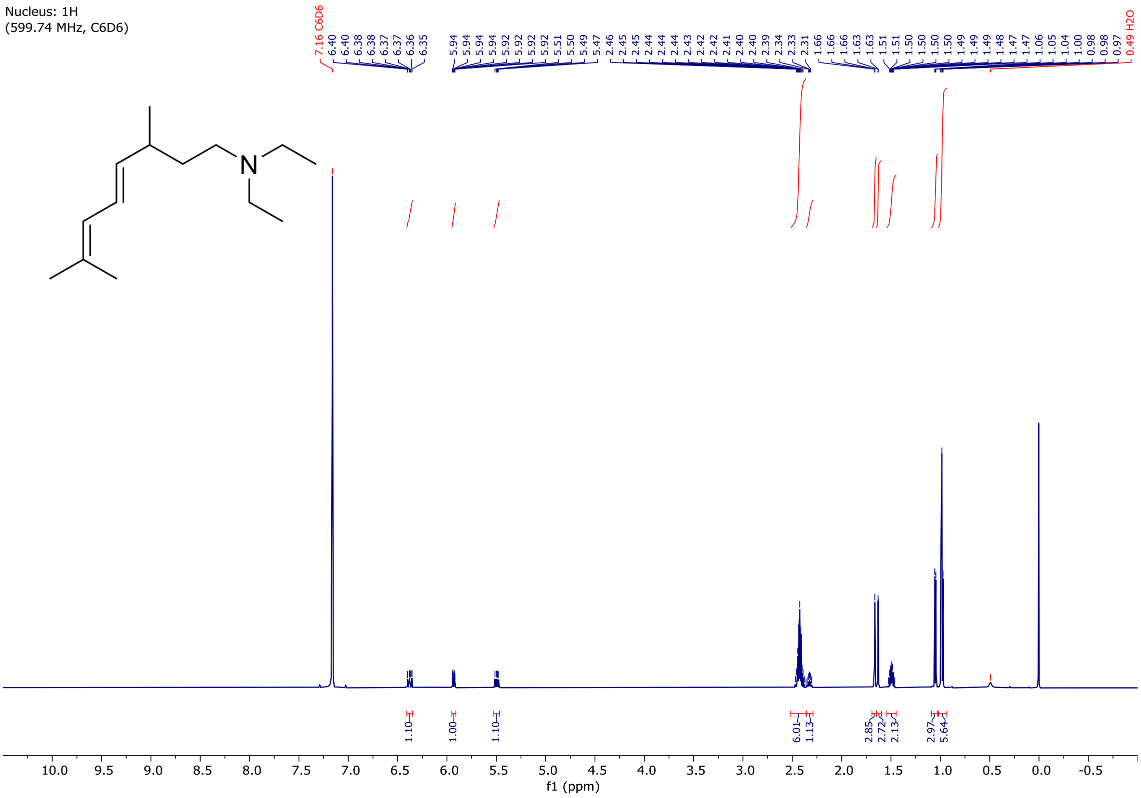

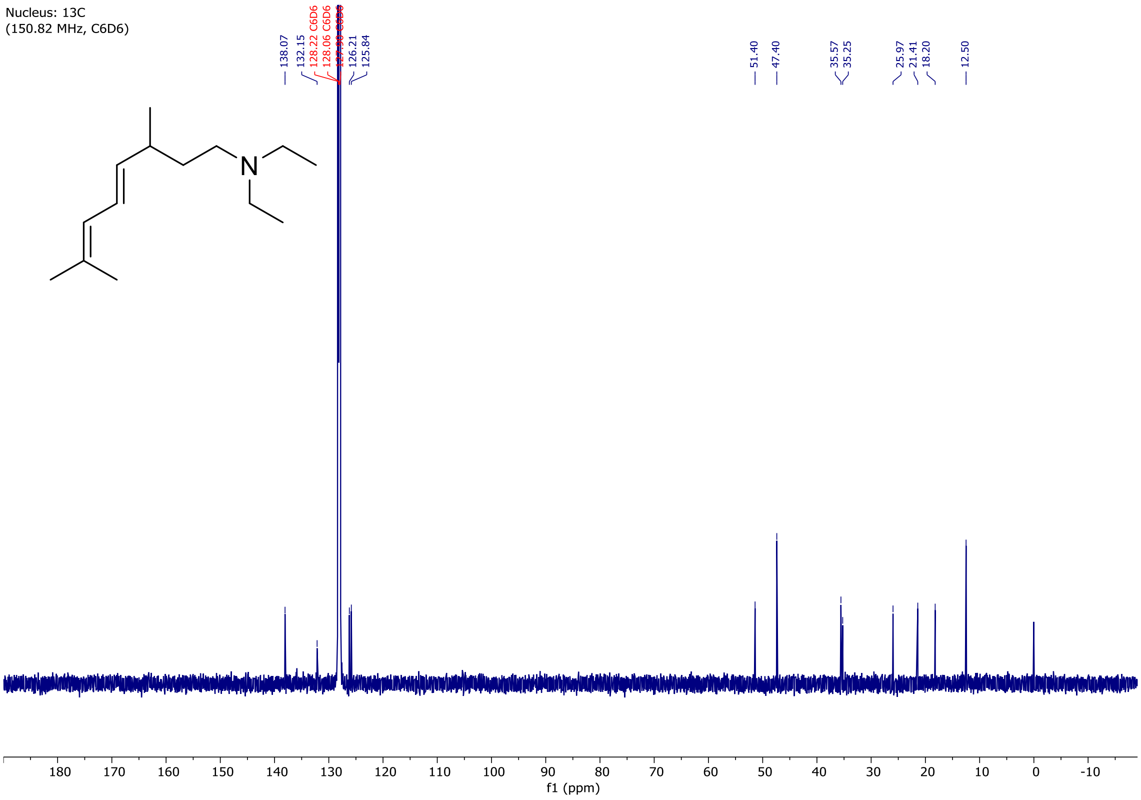

[5]Tetramethylsilane (TMS) was used as a reference.

Isomer **1bc**: HH-COSY and HSQC NMR.

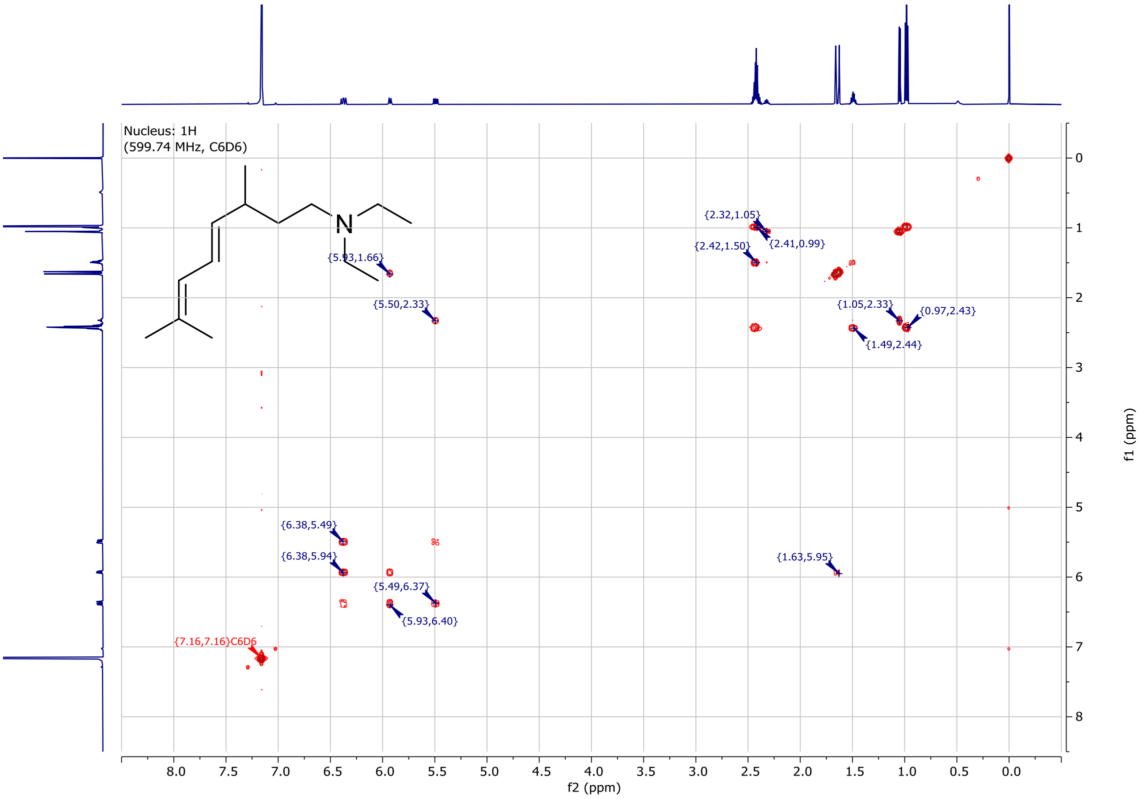

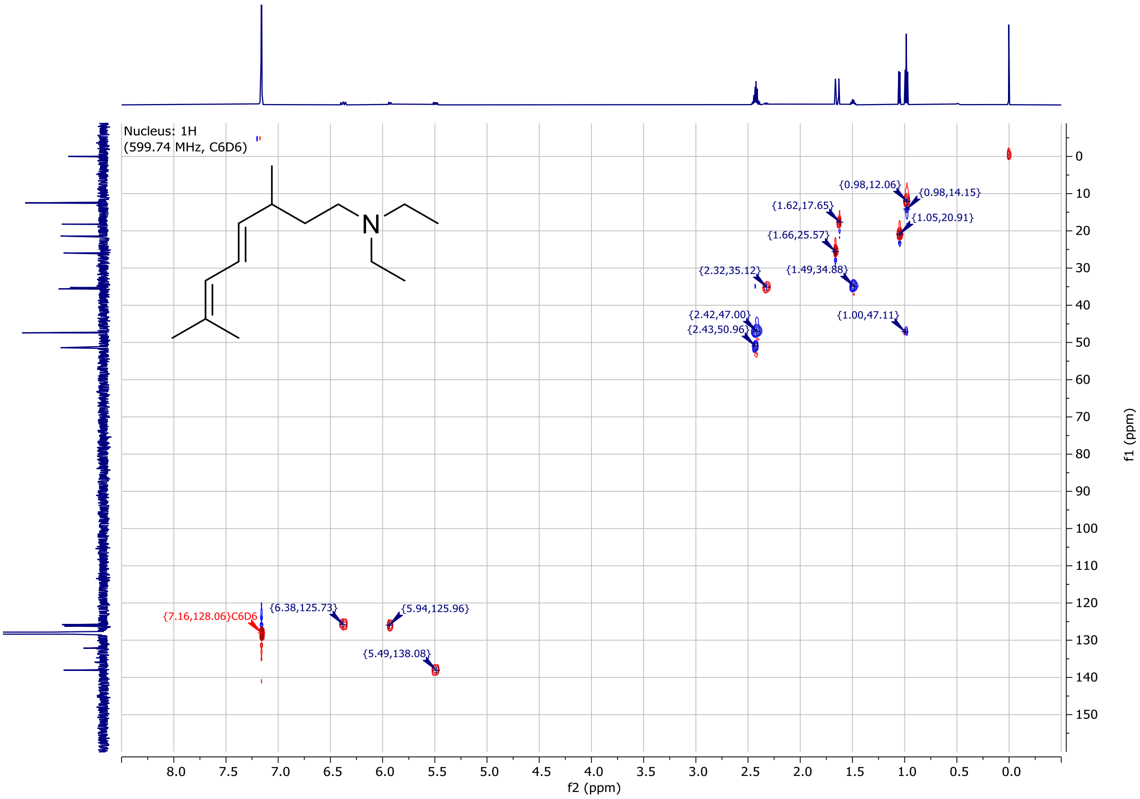

Isomer: (3*E*,5*E*)-*N*,*N*-diethyl-3,7-dimethylocta-3,5-dien-1-amine (1be)

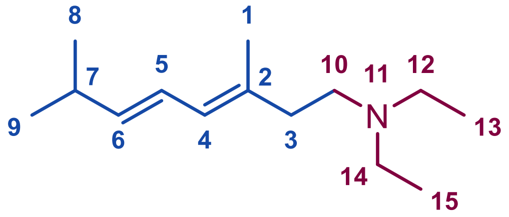

^{1}H NMR (600 MHz, C_6D_6): δ = 6.35 (ddd, *J* = 15.1, 10.7, 1.3 Hz, 1H, **5**), 5.99 (d, *J* = 10.7 Hz, 1H, **1**), 5.58 (dd, *J* = 15.1, 7.0 Hz, 1H, **4**), 2.56 - 2.52 (m, 2H, **10**), 2.45 - 2.41 (m, 4H, **13/15**), 2.32 - 2.25 (m, 1H, **7**), 2.22 (t, *J* = 8.2 Hz, 2H, **3**), 1.72 (d, *J* = 1.3 Hz, 3H, **6**), 1.00 - 0.96 (m, 12H, **8/9/14/15**).

^{13}C NMR (151 MHz, C_6D_6): δ = 139.51 (-CH, **6**), 135.08 (-$C_{quart.}$, **2**), 126.45 (-CH, **4**), 124.43 (-CH, **5**), 52.27 (-CH_2, **10**), 47.27 (-CH_2, **12/14**), 38.07 (-CH_2, **3**), 31.81 (-CH, **7**), 22.81 (-CH_3, **8/9**), 16.88 (-CH_3, **1**), 12.47 (-CH_3, **13/15**).

HH-COSY NMR (600 MHz, C_6D_6): δ = 6.36 (1H, **5**) and 6.00 (1H, **1**); 6.36 (1H, **5**) and 5.59 (1H, **4**); 5.99 (1H, **1**) and 1.72 (3H, **6**); 5.58 (1H, **4**) and 2.30 (1H, **7**); 2.53 (2H, **10**) and 2.23 (2H, **3**); 2.43 (4H, **13/15**) and 0.98 (12H, **8/9/14/15**); 2.33 (1H, **7**) and 0.99 (12H, **8/9/14/15**).

HMBC NMR (600 and 151 MHz, C_6D_6): δ = 6.38 (1H, **5**) and 31.81 (-CH, **7**); 5.98 (1H, **1**) and 139.70 (-CH, **6**); 5.99 (1H, **1**) and 124.52 (-CH, **5**); 5.99 (1H, **1**) and 38.07 (-CH_2, **3**); 5.99 (1H, **1**) and 17.01 (-CH_3, **1**); 5.58 (1H, **4**) and 126.54 (-CH, **4**); 5.57 (1H, **4**) and 31.94 (-CH, **7**), 5.58 (1H, **4**) and 22.83 (-CH_3, **8/9**); 2.54 (2H, **10**) and 135.14 (-$C_{quart.}$, **2**); 2.54 (2H, **10**) and 47.37 (-CH_2, **12/14**), 2.54 (2H, **10**) and 38.01 (-CH_2, **3**); 2.43 (4H, **13/15**) and 52.17 (-CH_2, **10**); 2.43 (4H, **13/15**) and 47.37 (-CH_2, **12/14**); 2.43 (4H, **13/15**) and 12.46 (-CH_3, **13/15**); 2.28 (1H, **7**) and 139.44 (-CH, **6**), 2.28 (1H, **7**) and 124.52 (-CH, **5**); 2.29 (1H, **7**) and 22.83 (-CH_3, **8/9**); 2.23 (1H, **7**) and 135.14 (-$C_{quart.}$, **2**); 2.23 (1H, **7**) and 126.54 (-CH, **4**); 2.23 (1H, **7**) and 52.17 (-CH_2, **10**); 2.23 (1H, **7**) and 17.01 (-CH_3, **1**); 1.72 (3H, **6**) and 135.14 (-$C_{quart.}$, **2**); 1.72 (3H, **6**) and 126.54 (-CH, **4**); 1.72 (3H, **6**) and 38.01 (-CH_2, **3**); 0.99 (12H, **8/9/14/15**) and 139.44 (-CH, **6**); 0.99 (12H, **8/9/14/15**)

and 47.37 (-CH_2, **12/14**); 0.99 (12H, **8/9/14/15**) and 31.94 (-CH, **7**); 0.99 (12H, **8/9/14/15**) and 22.83 (-CH_3, **8/9**).

Isomer **1be**: ^{1}H NMR.[6]

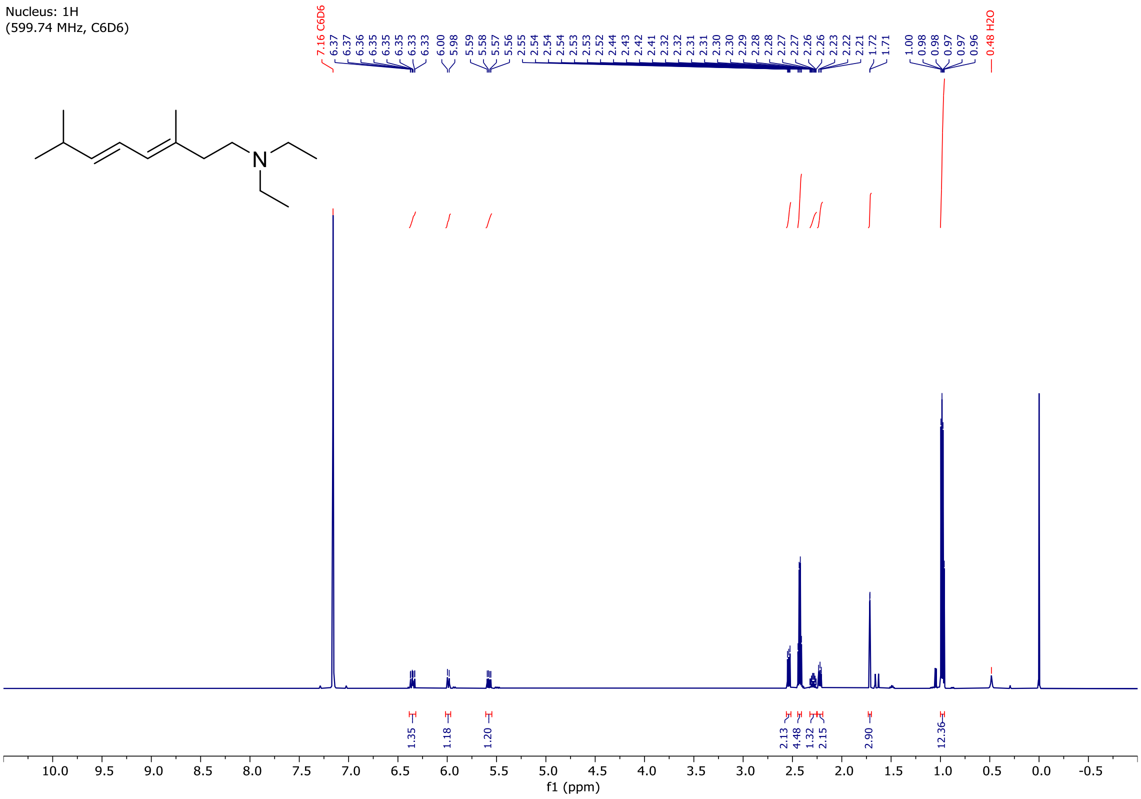

[6]Tetramethylsilane (TMS) was used as a reference.

Isomer **1be**: ^{13}C NMR and HH-COSY.

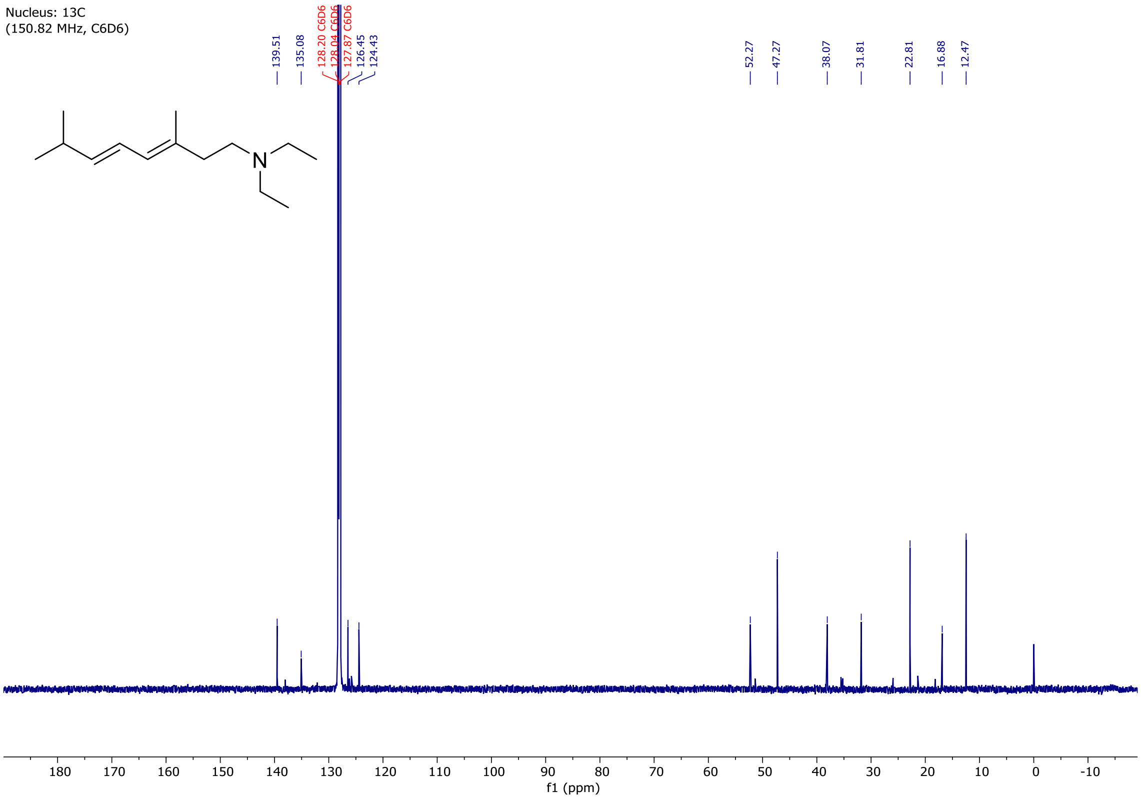

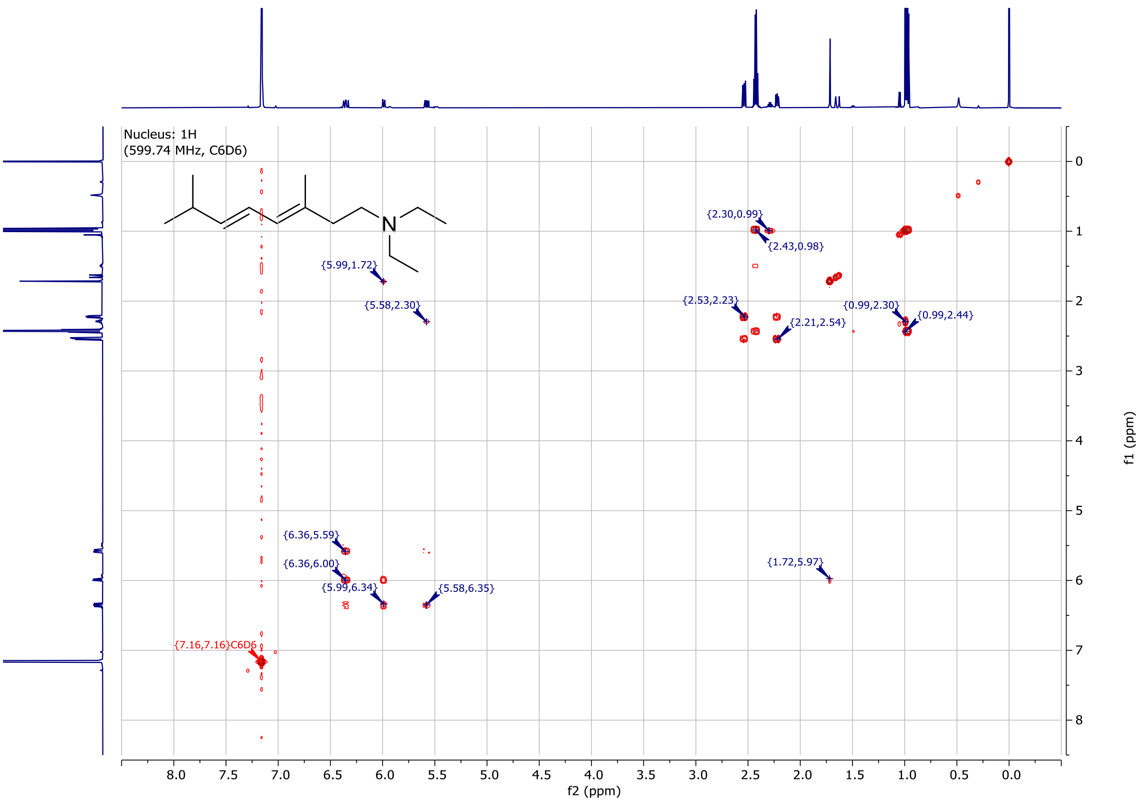

Isomer **1be**: HMBC NMR.

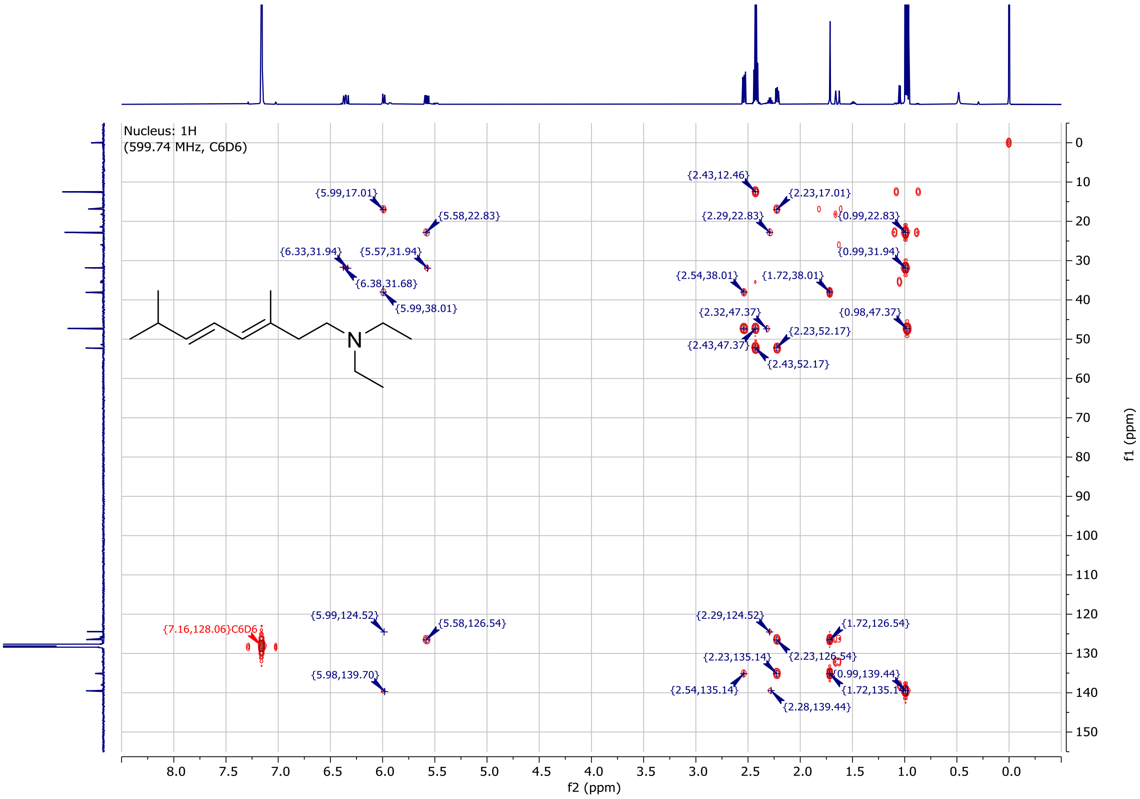

Isomer: (1*E*,5*E*)-*N,N*-diethyl-3,7-dimethylocta-1,5-dien-1-amine (1bf)

Because of its reactivity, enamine **1bf** was identified indirectly by isolation of the corresponding aldehyde (*E*-3,7-dimethyloct-5-enal). However, partial degradation of the aldehyde was observed.

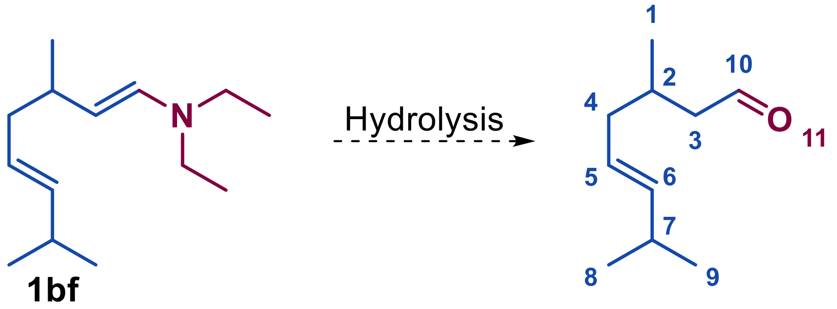

NMR analysis of **<u>(*E*)-3,7-dimethyloct-5-enal</u>:**

^{1}H NMR (600 MHz, C_6D_6): δ = 9.36 (t, *J* = 2.1 Hz, 1H, **10**), 5.30 (ddt, *J* = 15.3, 6.7, 1.3 Hz, 1H, **3**), 5.18 (dtd, *J* = 15.3, 7.0, 1.2 Hz, 1H, **2**), 2.15 - 2.19 (m, 1H, **7**), 1.94 (ddd, *J* = 16.2, 5.5, 1.8 Hz, 2H, **4a**), 1.82 - 1.69 (m, 3H, **3b/4**), 0.94 (d, *J* = 6.8 Hz, 6H, **8/9**), 0.74 (d, *J* = 6.6 Hz, 3H, **6**).

^{13}C NMR (151 MHz, C_6D_6): δ = 200.49 (**10**), 139.91 (**6**), 124.62 (**5**), 50.10 (**3**), 39.76 (**4**), 31.18 (**7**), 28.17 (**2**), 22.49 (**8/9**), 19.52 (**1**).

Isomer **1bf**: ^{1}H NMR and ^{13}C NMR.

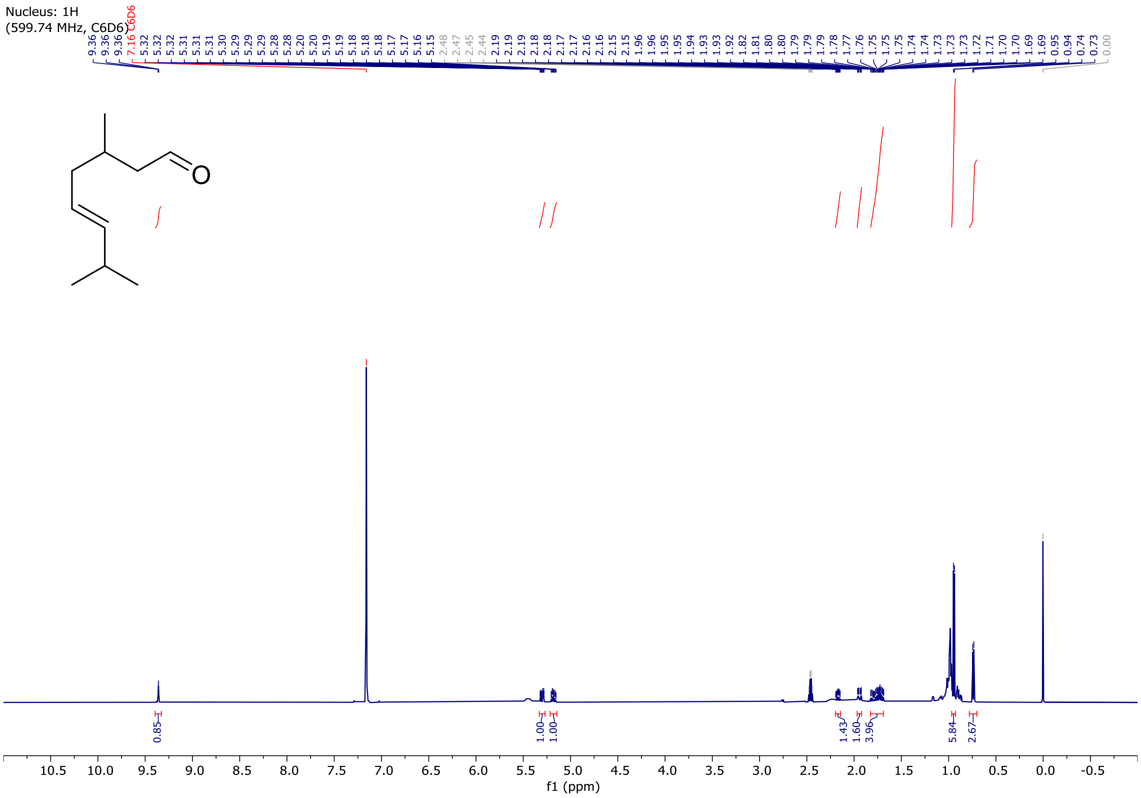

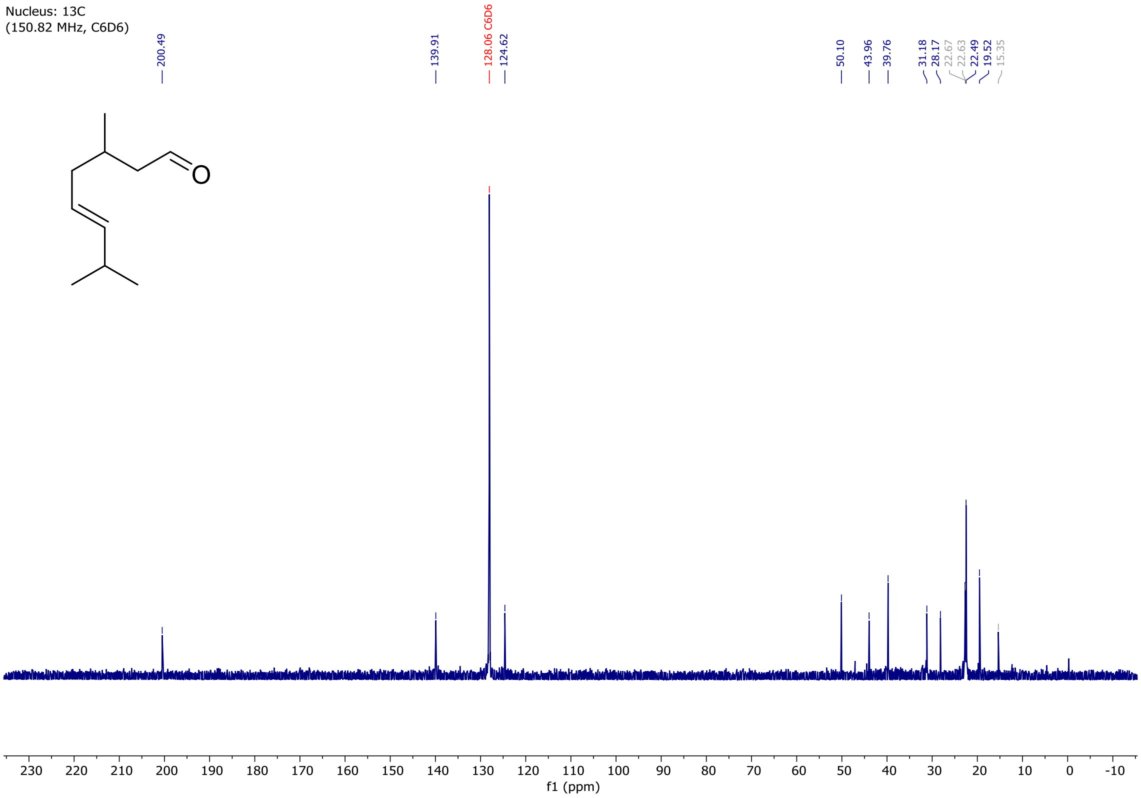

Isomer **1bf**: HMBC and HSQC NMR.

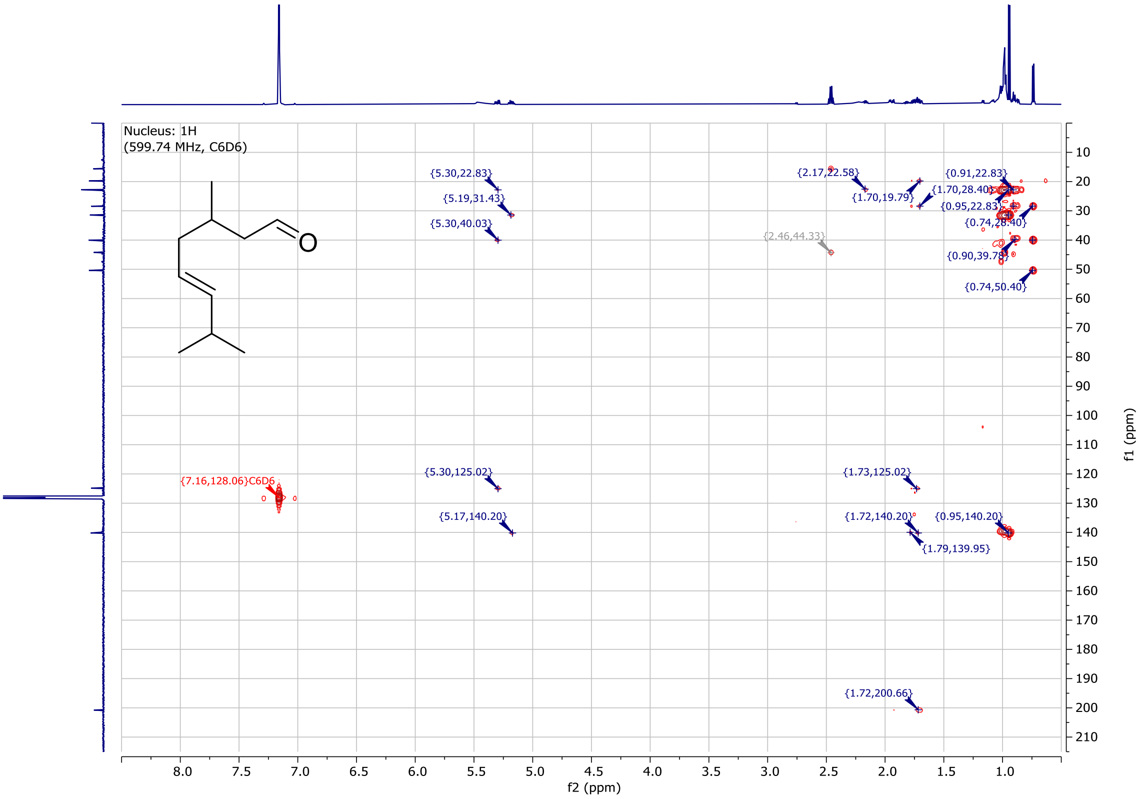

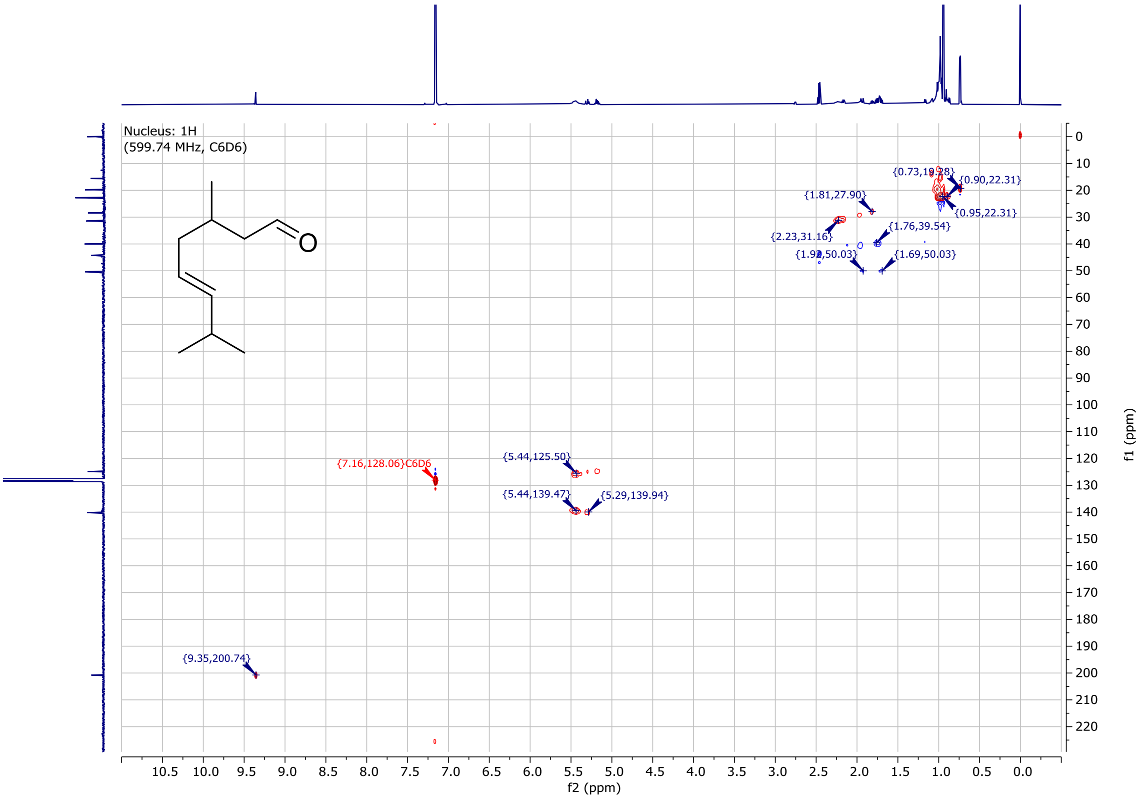

Isomer: (1*E*)-*N,N*-diethyl-3,7-dimethylocta-1,3-dien-1-amine (1bh/1bi)

Effective separation of enamine **1bi** (cis-isomer) and **1bh** (trans-isomer) could not be achieved by vacuum distillation. However, the chemical shifts of both compounds could be assigned from the cis/trans mixture by 2D-NMR analysis.

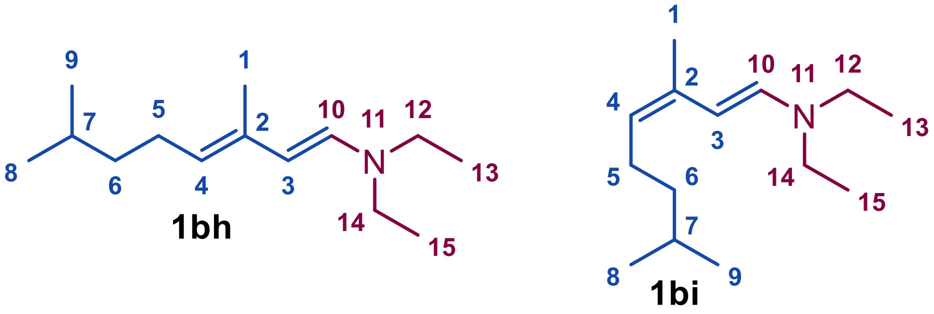

NMR analysis of **<u>Isomer 1bh</u>**:

^{1}H NMR (600 MHz, C_6D_6): δ = 6.13 (d, *J* = 14.1 Hz, 1H, **10**), 5.37 (t, *J* = 7.0 Hz, 1H, **4**), 5.34 (d, *J* = 14.0 Hz, 1H, **3**), 2.77 (d, 4H,**12/14**)[2], 2.26 (q, *J* = 7.5 Hz, 2H, **5**), 1.86 (q, *J* = 1.1 Hz, 3H, **1**), 1.63 - 1.58 (m, 2H, **7**), 1.40 - 1.34 (m, 3H, **6**), 0.92 (d, *J* = 6.6 Hz, 6H, **8/9**), 0.87 (t, 6H, **13/15**)[2].

^{13}C NMR (151 MHz, C_6D_6): δ = 135.20 **(10)**, 133.55 **(2)**, 122.06 **(4),** 104.53 **(3)**, 97.01, 45.13 **(12/14)**, 40.16 **(6)**, 27.99 or 27.97 **(7)**[2], 26.53 **(5)**, 22.85 **(8/9)**, 13.20 **(1)**, 13.09 or 13.08 **(13/15)**[2].

NMR analysis of **<u>Isomer 1bi</u>**:

^{1}H NMR (600 MHz, C_6D_6): δ = 6.21 (d, *J* = 13.9 Hz, 1H, **10**), 5.56 (d, *J* = 14.0 Hz, 1H, **3**), 5.11 (t, *J* = 7.1 Hz, 1H, **4**), 2.77 (d, 4H,**12/14**)[7], 2.37 (qd, *J* = 7.4, 7.4 Hz, 2H, **5**), 1.98 (q, *J* = 1.2 Hz, 3H, **1**), 1.68 - 1.65 (m, 2H, **7**), 1.46 - 1.41 (m, 2H, **6**), 0.94 (d, *J* = 6.6 Hz, 6H, **8/9**), 0.87 (t, 6H, **13/15**)[2].

^{13}C NMR (151 MHz, C_6D_6): δ = 137.22 **(10)**, 132.33 **(2)**, 120.68 **(4)**, 97.01 **(3)**, 45.25 **(12/14)**, 39.97 **(6)**, 27.99 or 27.97 **(7)**[2], 25.88 **(5)**, 22.90 **(8/9)**, 21.54 **(1)**, 13.09 or 13.08 **(13/15)**[2].

---

[7]Overlap of signals. Exact assignment not possible.

Isomer **1bh/1bi**: ^{1}H NMR and ^{13}C NMR.

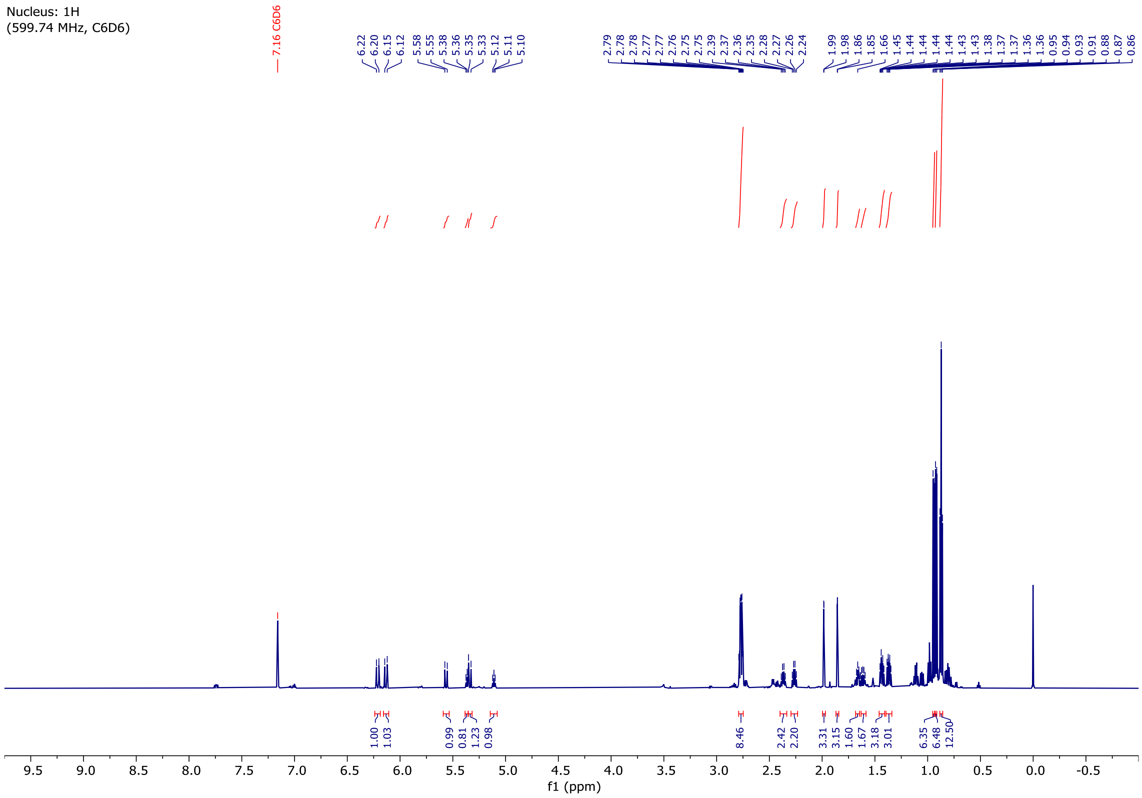

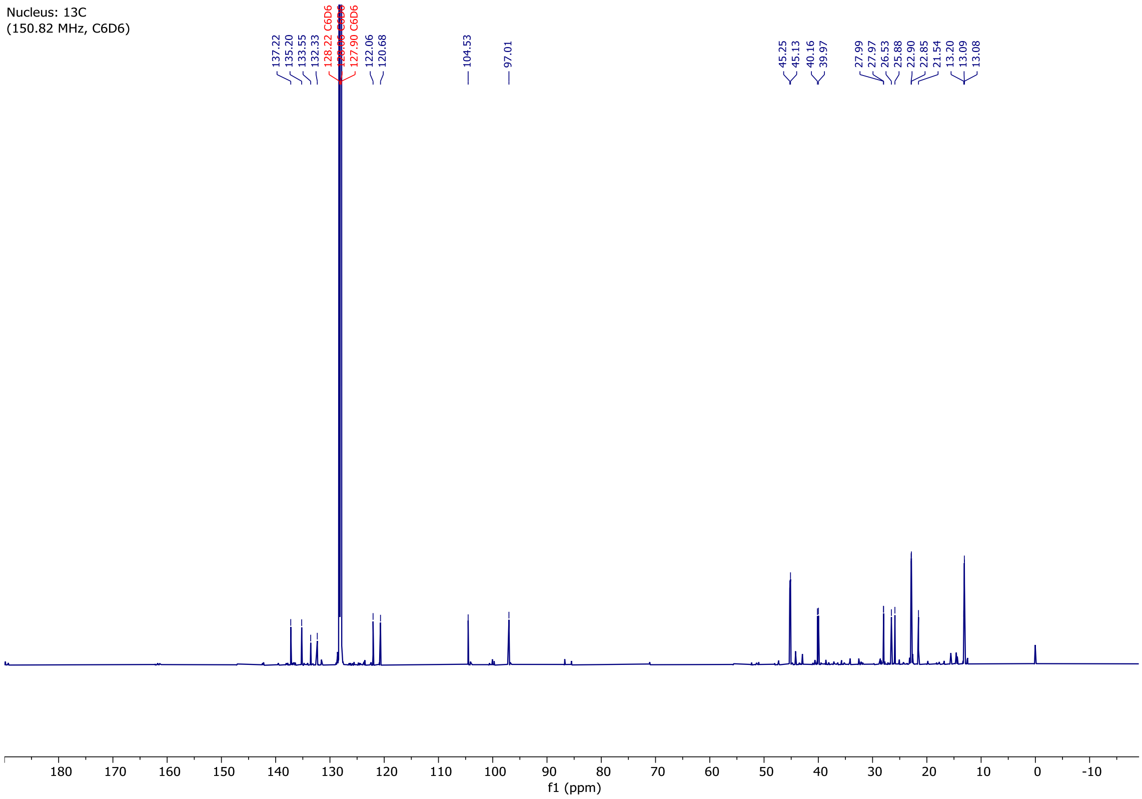

Isomer **1bh/1bi**: HH-COSY and HSQC NMR.

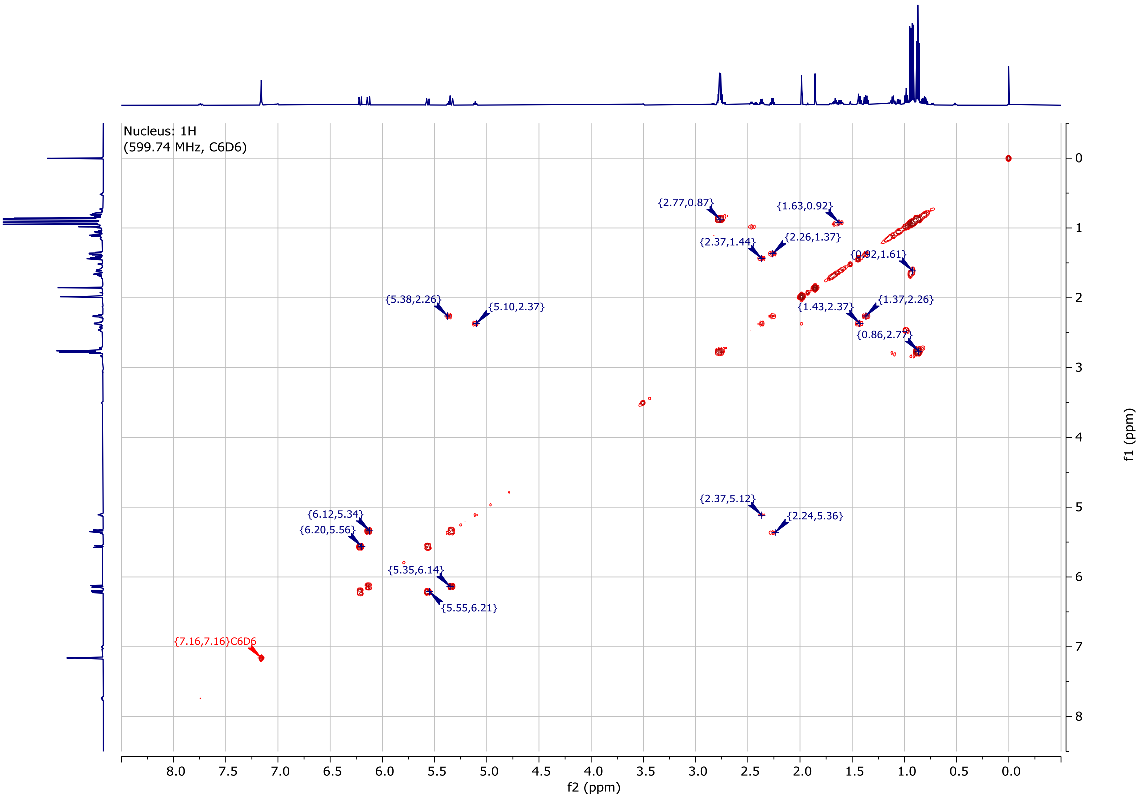

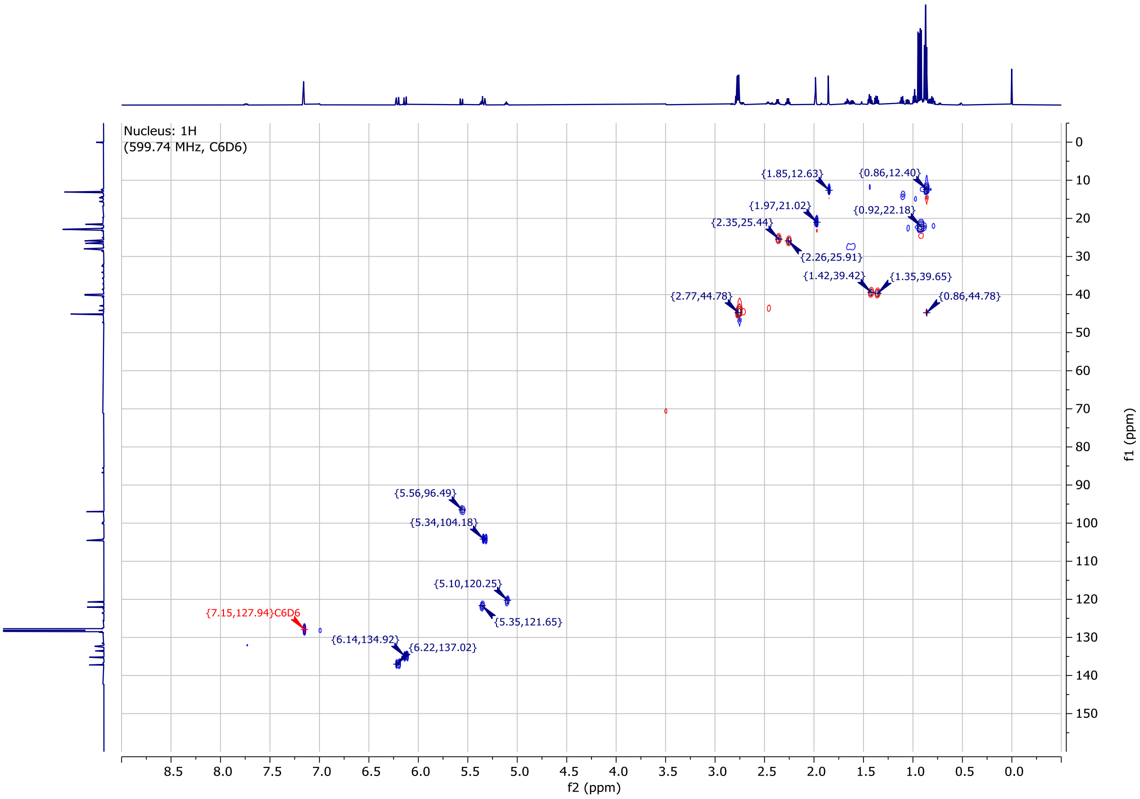

Isomer **1bh/1bi**: HMBC and NOESY NMR.

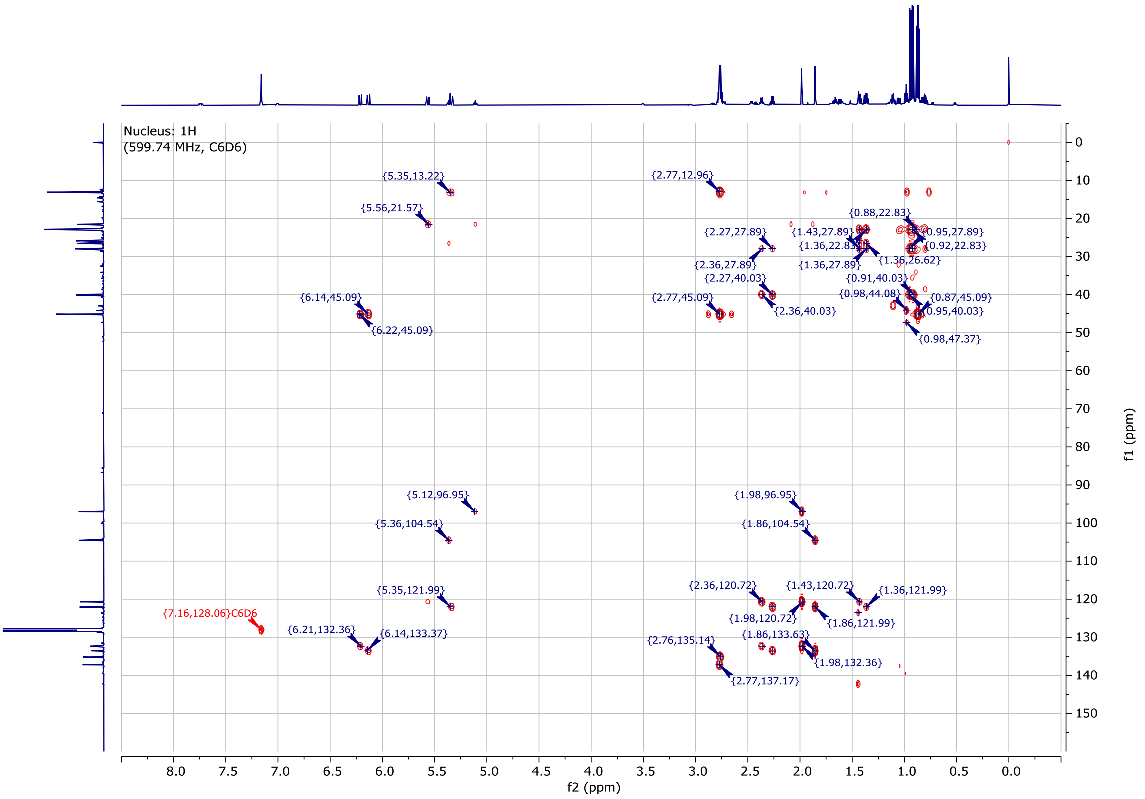

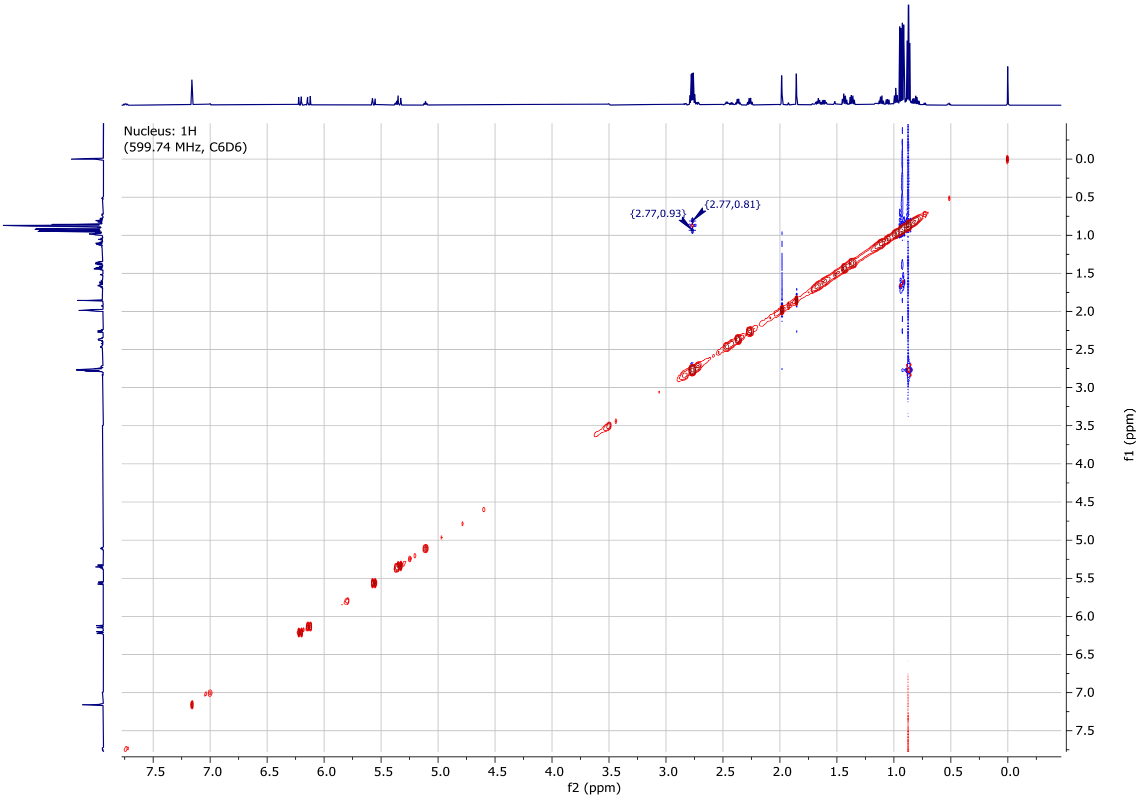

Isomer: (3*Z*,5*E*)-*N,N*-diethyl-3,7-dimethylocta-3,5-dien-1-amine (1bd) and (*E*)-*N,N*-diethyl-3,7-dimethylocta-1,7-dien-1-amine (1bg)

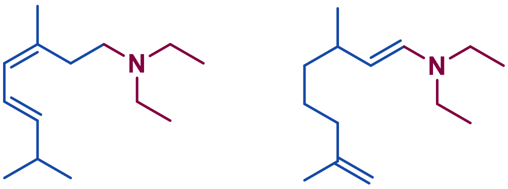

1bd **1bg**

In the case of isomer **1bd** and **1bg**, product mixtures were obtained, and it was therefore not possible to obtain spectra of the pure components. Nevertheless, the identity could be confirmed by NMR spectroscopy.

GC-Spectra: Isomerization of 1a using Co/L1/DIBAL-H

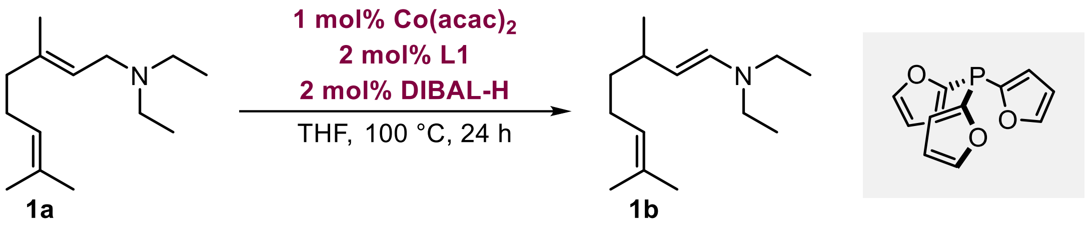

The isomerization was carried out with the same procedure that was used for the initial ligand screening (section 3). However, the reaction mixture was not quenched to the respective aldehydes after catalysis. The resulting product mixture was directly analyzed by GC after vacuum distillation.

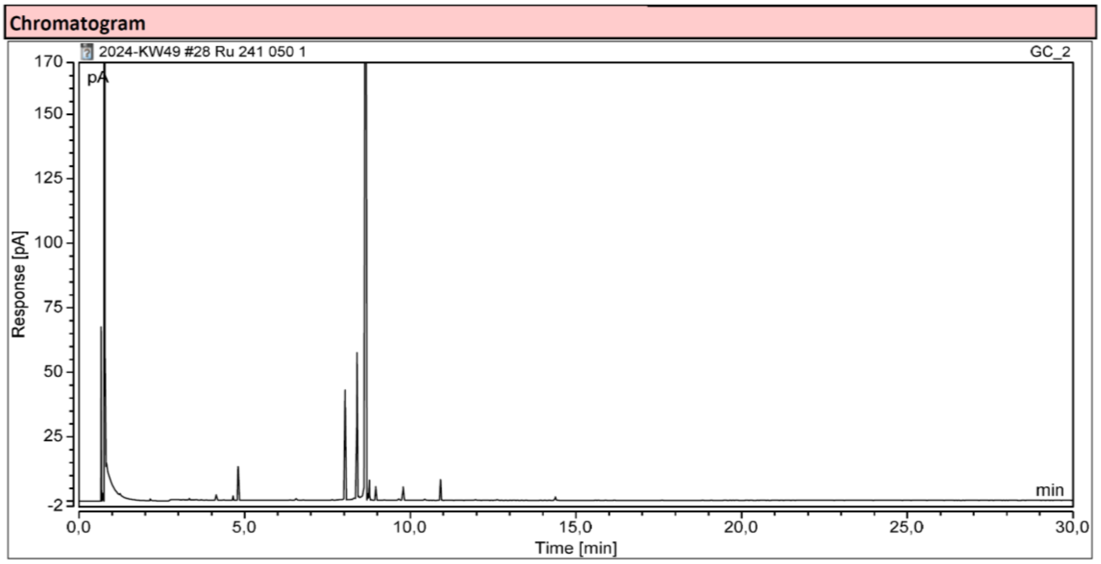

Entry	Retention time [min]	Isomer
1	4.802	**1c**
2	8.030	**1bf**
3	8.394	**1bd + 1bg**
4	8.652	**1b**
5	8.763	**1a**
6	8.956	**1be**
7	9.786	**1bi**

GC-Spectra: Isomerization of 1a using Co/L15/DIBAL-H

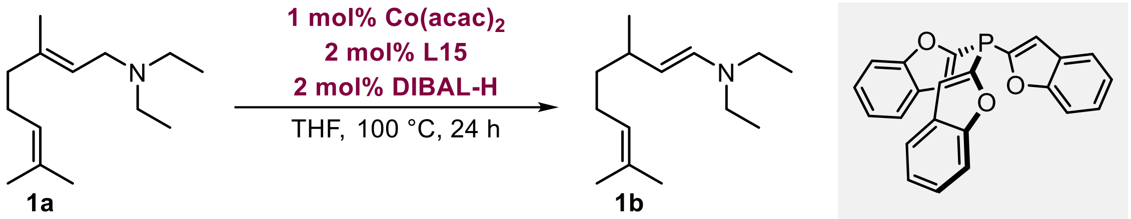

The isomerization was carried out with the same procedure that was used for the initial ligand screening (section 3). However, the reaction mixture was not quenched to the respective aldehydes after catalysis. The resulting product mixture was directly analyzed by GC after vacuum distillation.

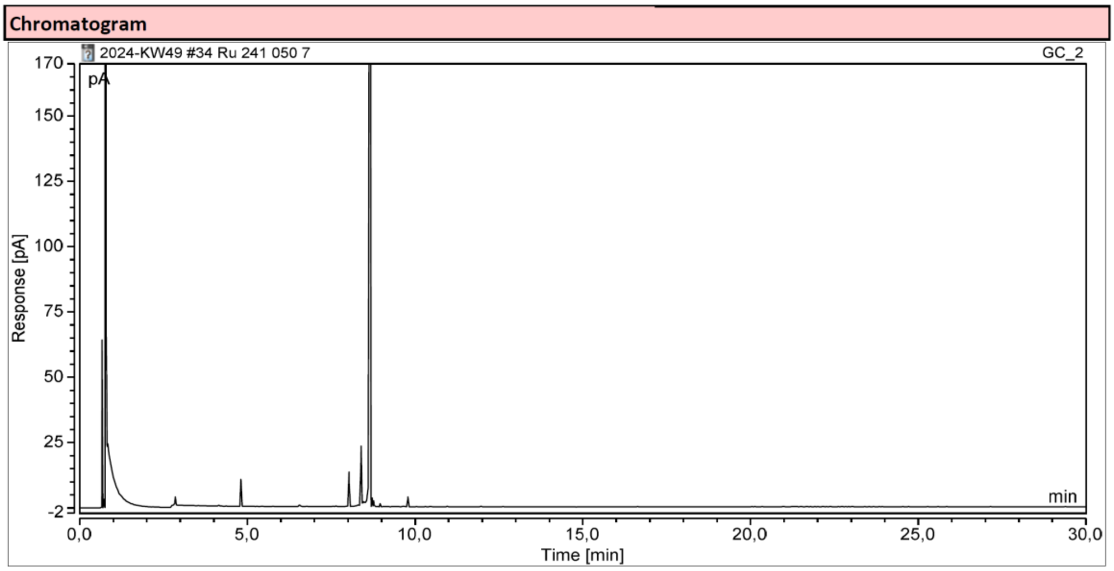

Entry	Retention time [min]	Isomer
1	4.803	**1c**
2	8.028	**1bf**
3	8.308	**1bd + 1bg**
4	8.658	**1b**
5	8.721	**1a**
6	9.786	**1bi**

8 Single Crystal X-ray Diffraction

Crystal data of CCDC 2379867 (**L15**): $C_{24}H_{15}O_3P$, *M* = 382.33, monoclinic, space group $P2_1$, $\bar{1}a$ = 5.9038(7), *b* = 16.1922(18), *c* = 9.7412(11) Å, *b* = 97.5899(19)°, *V* = 923.06(18) Å^3, *T* = 150(2) K, *Z* = 2, 13574 reflections measured, 4465 independent reflections (R_{int} = 0.0193), final *R* values ($I > 2\sigma(I)$): R_1 = 0.0511, wR_2 = 0.1332, final *R* values (all data): R_1 = 0.0546, wR_2 = 0.1370, 242 parameters.

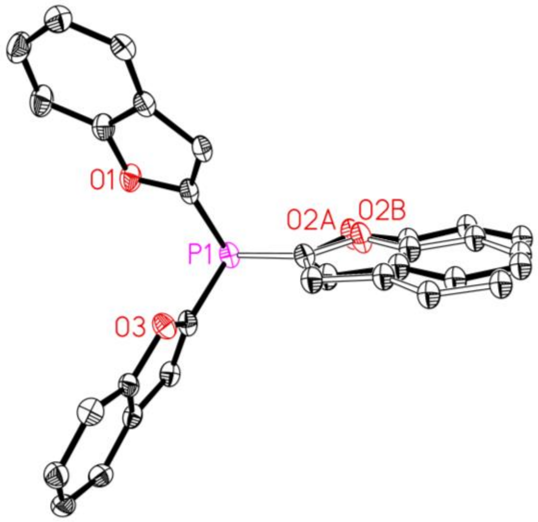

Figure S3: Molecular structure of **L15**. Displacement ellipsoids correspond to 30% probability. Hydrogen atoms are omitted for clarity. Lower occupied parts of disorder are shown with unfilled lines.

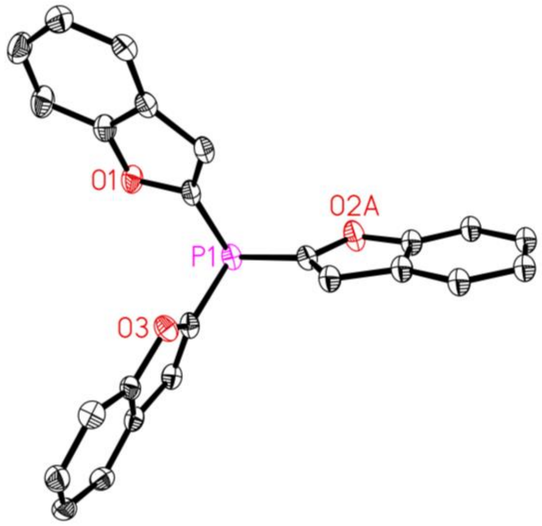

Figure S4: Molecular structure of **L15**. Displacement ellipsoids correspond to 30% probability. Hydrogen atoms and the lower occupied part of one disordered benzofuran unit are omitted for clarity.

Crystal data of CCDC 2379869 (**L16**): $C_{30}H_{15}O_6P$, M = 502.39, monoclinic, space group $P2_1/c$, $\bar{1}a$ = 22.3733(7), b = 6.3225(1), c = 16.7540(5) Å, b = 110.705(2)°, V = 2216.87(11) Å^3, T = 150(2) K, Z = 4, 35378 reflections measured, 6000 independent reflections (R_{int} = 0.0226), final R values ($I > 2\sigma(I)$): R_1 = 0.0383, wR_2 = 0.1059, final R values (all data): R_1 = 0.0422, wR_2 = 0.1090, 334 parameters.

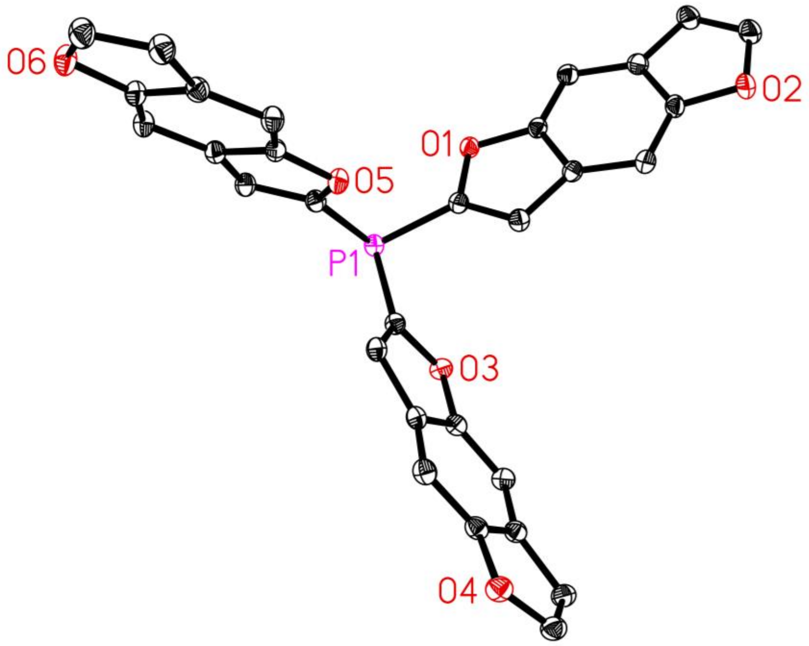

Figure S5: Molecular structure of **L16**. Displacement ellipsoids correspond to 30% probability. Hydrogen atoms are omitted for clarity.

Crystal data of CCDC 2379870 (**L24**): $C_{28}H_{17}O_3P$, *M* = 432.38, monoclinic, space group $P2_1$, $\bar{1}a$ = 6.4528(3), *b* = 14.4005(5), *c* = 11.2976(5) Å, *b* = 103.265(3)°, *V* = 1021.80(8) $Å^3$, *T* = 150(2) K, *Z* = 2, 15678 reflections measured, 5523 independent reflections (R_{int} = 0.0156), final *R* values ($I > 2\sigma(I)$): R_1 = 0.0301, wR_2 = 0.0788, final *R* values (all data): R_1 = 0.0314, wR_2 = 0.0795, 289 parameters.

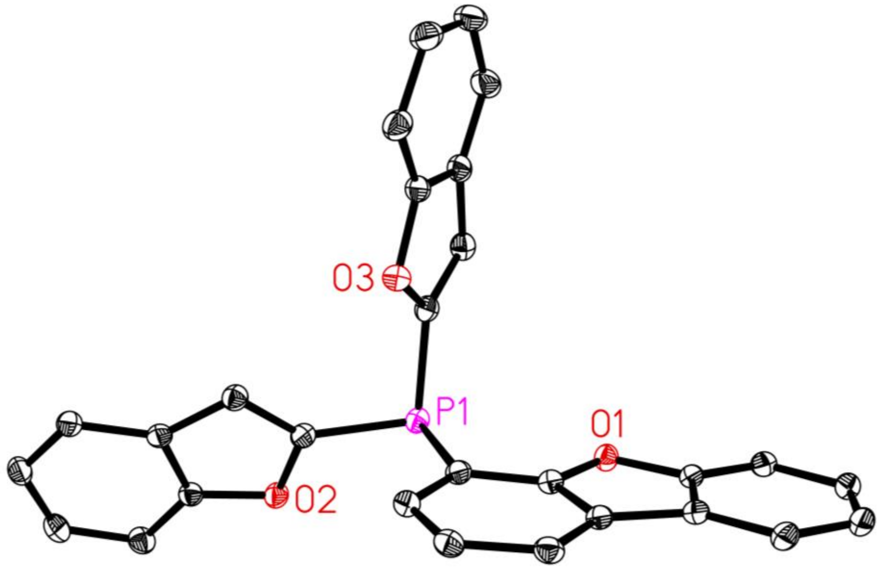

Figure S6: Molecular structure of **L24**. Displacement ellipsoids correspond to 30% probability. Hydrogen atoms are omitted for clarity.

Crystal data of CCDC 2379868 (**Co-1**): $C_{66}H_{60}CoO_{12}P_2$, M = 1166.01, triclinic, space group $P\bar{1}$, $\bar{1}a$ = 9.6953(9), b = 10.0691(10), c = 16.4128(15) Å, a = 87.055(3), b = 75.572(2), g = 67.290(2)°, V = 1429.6(2) Å^3, T = 150(2) K, Z = 1, 72188 reflections measured, 6913 independent reflections (R_{int} = 0.0438), final R values ($I > 2\sigma(I)$): R_1 = 0.0391, wR_2 = 0.1066, final R values (all data): R_1 = 0.0454, wR_2 = 0.1129, 369 parameters.

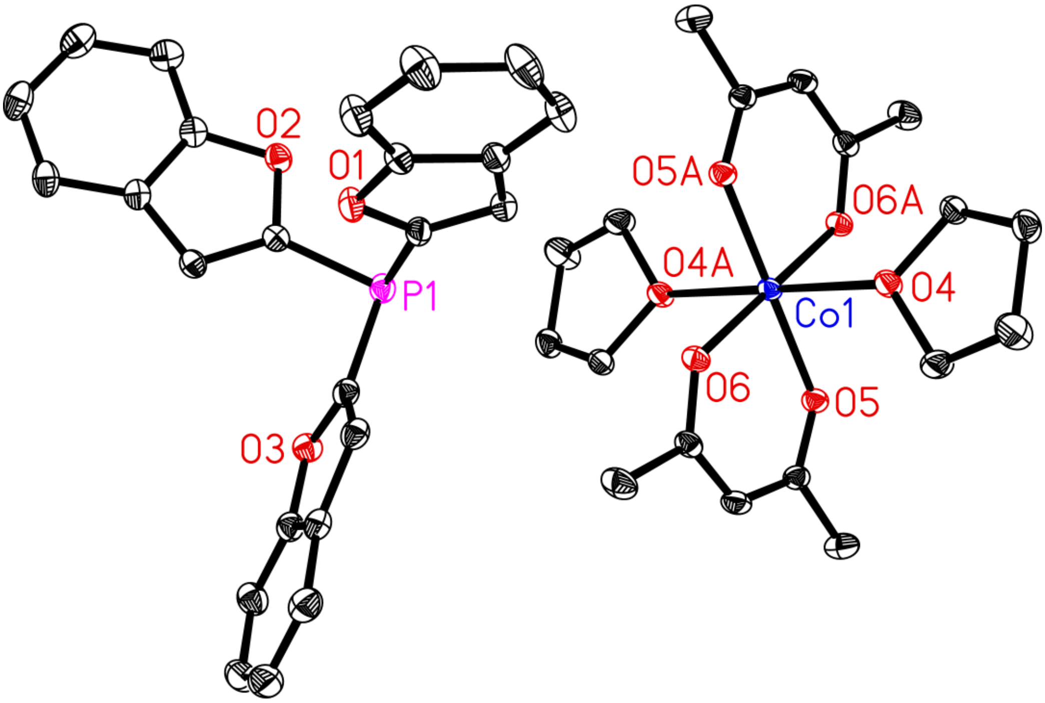

Figure S7: Molecular structure of **Co-1**. Displacement ellipsoids correspond to 30% probability. Hydrogen atoms are omitted for clarity.

Crystal data of CCDC 2379866 (**Co-2**): $C_{96}H_{60}CoO_{12}P_4$, M = 1588.25, trigonal, space group $R3$, $\bar{1}a$ = 22.9793(6), c = 17.0108(5) Å, V = 7779.1(5) Å^3, T = 150(2) K, Z = 3, 45475 reflections measured, 6095 independent reflections (R_{int} = 0.046), final R values ($I > 2\sigma(I)$): R_1 = 0.0355, wR_2 = 0.0886, final R values (all data): R_1 = 0.0371, wR_2 = 0.0897, 340 parameters.

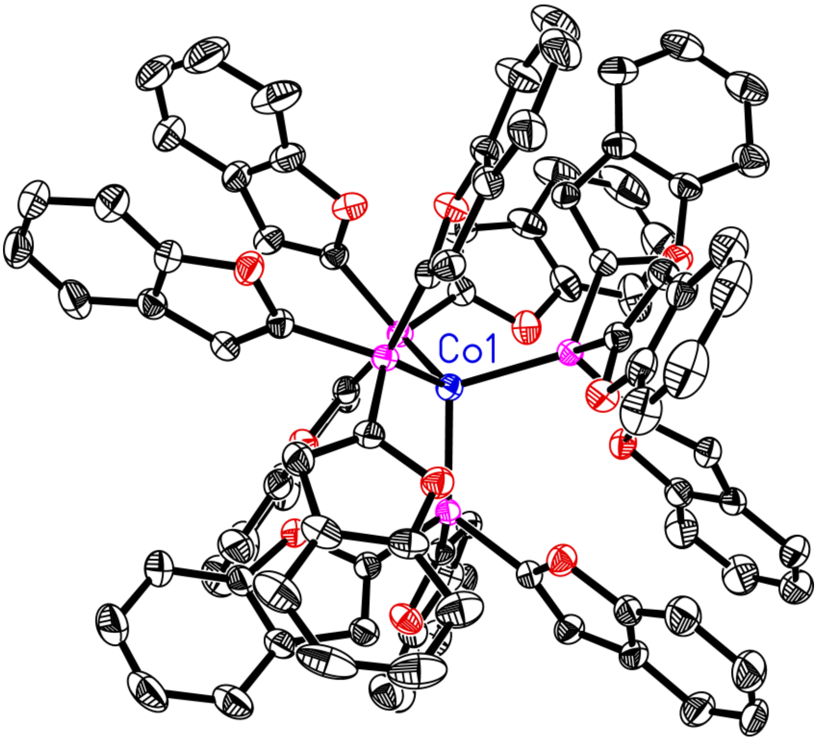

Figure S8: Molecular structure of **Co-2** (O red, P magenta, Co blue). Displacement ellipsoids correspond to 30% probability. Hydrogen atoms are omitted for clarity. Due to unresolved disorder SAME instruction for some benzofuran units are used to optimize their geometry.

Complex synthesis of Co-2:

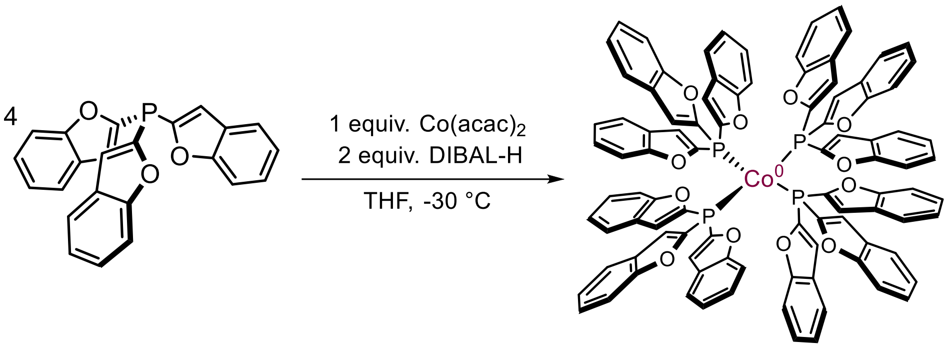

Under argon atmosphere, a flame dried 25 mL Schlenk flask was charged with $Co(acac)_2$ (16.1 mg, 0.0625 mmol, 1 equiv.) and **L15** (95.6 mg, 0.25 mmol, 4 equiv.). Then, 4 ml anhydrous THF were added, and the mixture was stirred for approximately 5 min and cooled to -78 °C. Afterwards, DIBAL-H was added dropwise (0.5 mL, 0.25 mM, 0.125 mmol, 2 equiv.), and the reaction mixture was stirred for 1 h at -30 °C. The brown solution was then anaerobically filtered and layered with anhydrous *n*-pentane. After two weeks of crystallization at -30 °C, dark red platelet crystals were obtained, which were dried under vacuum (38.7 mg, 0.024 mmol, 39% yield).

HMRS of Co-2

In an argon filled glovebox, **Co-2** (2.5 mg, 1.6 µmol) was charged with 1.5 mL anhydrous toluene in a GC-vial. The freshly prepared complex solution was measured directly after preparation to avoid decomposition.

Formula	Expected Mass	Observed Mass	Error PPM	Error mDa
$C_{96}H_{60}CoO_{12}P_4$	1588.2440	1588.2448	0.5	0.8

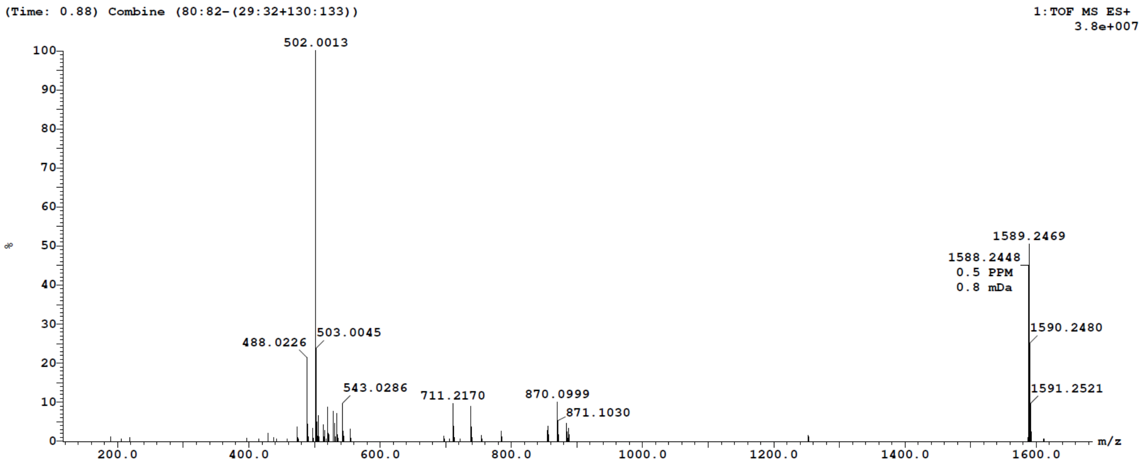

Figure S9: HRMS spectra of **Co-2** in toluene.

Crystallization experiment with COD:

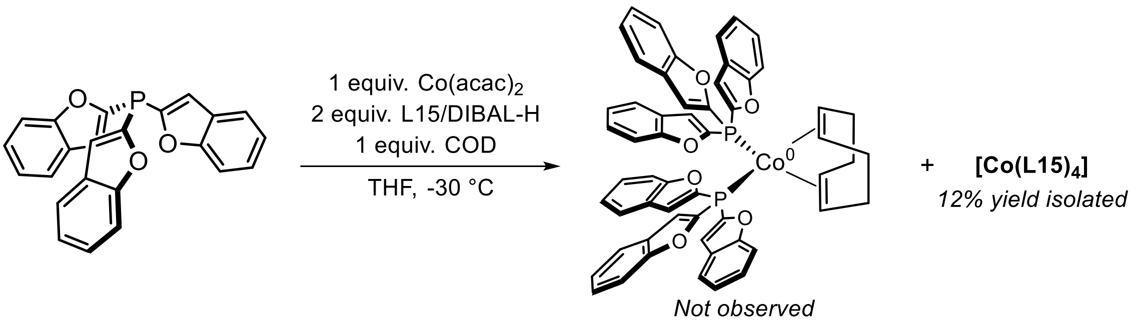

In an argon filled glovebox, a 25 mL Schlenk flask was charged with $Co(acac)_2$ (32.2 mg, 0.125 mmol, 1 equiv.) and **L15** (95.6 mg, 0.25 mmol, 2 equiv.). Next, 10 ml anhydrous THF were added, and the mixture was stirred for approximately 5 min. Then, an anhydrous COD-THF solution (2 mL, 0.0625 M, 0.125 mmol, 1 equiv.) was added dropwise and the solution stirred 30 min. At -78 °C, DIBAL-H was added dropwise (0.125 mmol, 2 equiv.) and the mixture was stirred for 2 h at -30 °C. The obtained brown solution was anaerobically filtered and layered with anhydrous *n*-pentane. After two weeks of crystallization at -30 °C, red crystals of **Co-2** were obtained (23,8 mg, 0.015 mmol, 12% yield). However, the isolation of a Co(0)/COD/**L15** complex could not be accomplished, and subsequent attempts were also unsuccessful.

9 Mechanistic Investigations

9.1 Comparison: isolated Co complex and *in situ* Co catalyst

The reactions have been carried out in 10 mL Schlenk pressure tubes with the same procedure as described for the initial screening of phosphine ligands (see section 3 for details). Due to the limited solubility of **Co-2** in THF, the catalysis of **1a** was performed in a THF/toluene mixture.

Table S10: Comparison of **Co-2** and *in situ* Co catalyst for the isomerization of **1a**.

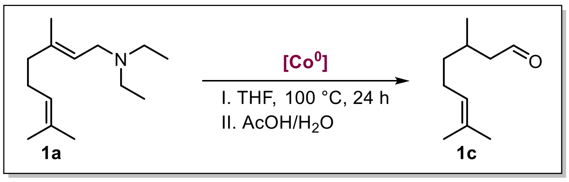

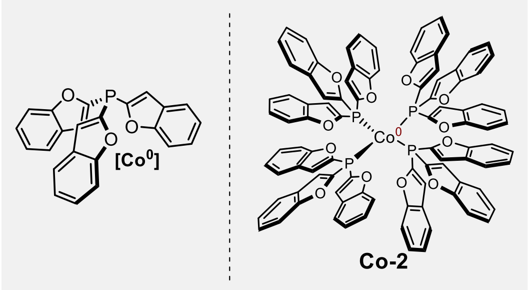

Entry	Reaction conditions Catalyst-loading	Yield 1c[a] [mol%]	Conversion 1a[a] [mol%]
1	0.1 mol% $Co(acac)_2$, 0.2 mol% DIBAL-H, 0.2 mol% **L15**	98	99
2	0.1 mol% **Co-2**[b]	18	19
3	0.2 mol% **Co-2**[b]	67	68
4	0.3 mol% **Co-2**[b]	98	99

[a]General reaction conditions: Substrate **1a** (2.5 mmol), THF (1.5 mL), $Co(acac)_2$, **L15**, DIBAL-H, 100 °C, 24 h. [b]Solvent: 1.5 mL THF/toluene (1:1 ratio). The isolated yields and the substrate conversions were determined by GC using *n*-hexadecane as internal standard.

9.2 Control Experiments

The different control experiments, which have been performed for the Co-catalyzed isomerization of **1a** are summarized in **Table S11** and **Table S12**. The reactions have been carried out with the same procedure as described for the initial screening of phosphine ligands (section 3)

Table S11: Control experiments for the Co-catalyzed isomerization of **1a**.

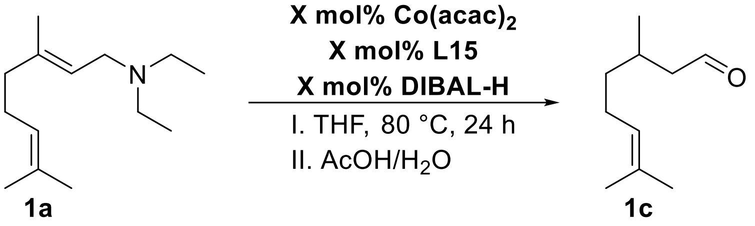

Entry	Co(acac)$_2$ [mol%]	DIBAL-H [mol%]	L15 [mol%]	Yield 1c[a] [mol%]	Conversion 1a[a] [mol%]
1	0.5	-	-	-	traces
2	0.5	-	1	-	traces
3	0.5	1	-	0	5
4	-	1	-	-	traces

[a]General reaction conditions: Substrate **1a** (2.5 mmol), THF (1.5 mL), Co(acac)$_2$, **L15**, DIBAL-H, 80 °C, 24 h. The isolated yields and the substrate conversions were determined by GC using *n*-hexadecane as internal standard.

Co-catalyzed isomerization of 1a under H_2-atmosphere:

In an argon filled glovebox, $Co(acac)_2$ and **L15** were weighed into a glass vial equipped with a stir bar and 2 mL of THF were injected. Then, DIBAL-H was added dropwise, and the mixture was stirred for approximately 2 min. Next, the substrate **1a** (523 mg, 2.5 mmol) was added and the vial was closed with a screw cap containing a septum. Before, the vial was transferred into an autoclave, the septum was punctured with a needle to allow gas exchange. Then, the autoclave was flushed ten times with 5 to 10 bar hydrogen. Finally, the autoclave was charged with the desired hydrogen pressure and was placed in a preheated aluminum block. After, the reaction time was finished, the autoclave was cooled with an ice bath to room temperature and the gas pressure was released. The further work-up was performed with the same procedure as described for the initial screening of phosphine ligands (section 3).

Table S12: Co-catalyzed isomerization of **1a** under H_2-atmosphere.

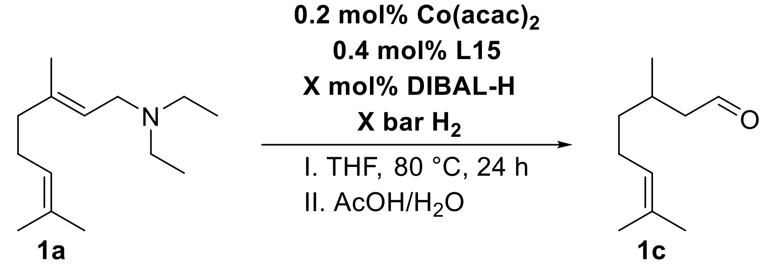

Entry	H_2 [bar]	DIBAL-H [mol%]	$Co(acac)_2$ [mol%]	L15 [mol%]	Yield 1c[a] [mol%]	Conversion 1a[a] [mol%]
1	20	-	0.2	0.4	-	traces
2	50	-	0.2	0.4	-	traces
3	50	0.4	0.2	0.4	0	4

[a]General reaction conditions: Substrate **1a** (2.5 mmol), THF (2 mL), $Co(acac)_2$, **L15**, DIBAL-H, 80 °C, 24 h. The isolated yields and the substrate conversions were determined by GC using *n*-hexadecane as internal standard.

9.3 Mercury Drop Test

Under argon atmosphere, a 5 mL Schlenk pressure tube was charged with Co(acac)$_2$ (3.2 mg, 12.5 µmol), **Ligand** (25 µmol) and a stirring bar. Then, 1.5 mL of anhydrous THF was injected, followed by the addition of DIBAL-H (25 µmol). After 2 min of stirring, first **1a** (523.5 mg, 2.5 mmol) and then **Hg** (approx. 40 mg) were added to the reaction mixture. Afterwards, the Schlenk pressure tube was sealed and heated for 24 h. Finally, the substrate conversion of **1a** and the yield of **1b** were determined by GC analysis, using *n*-hexadecane as the internal standard.

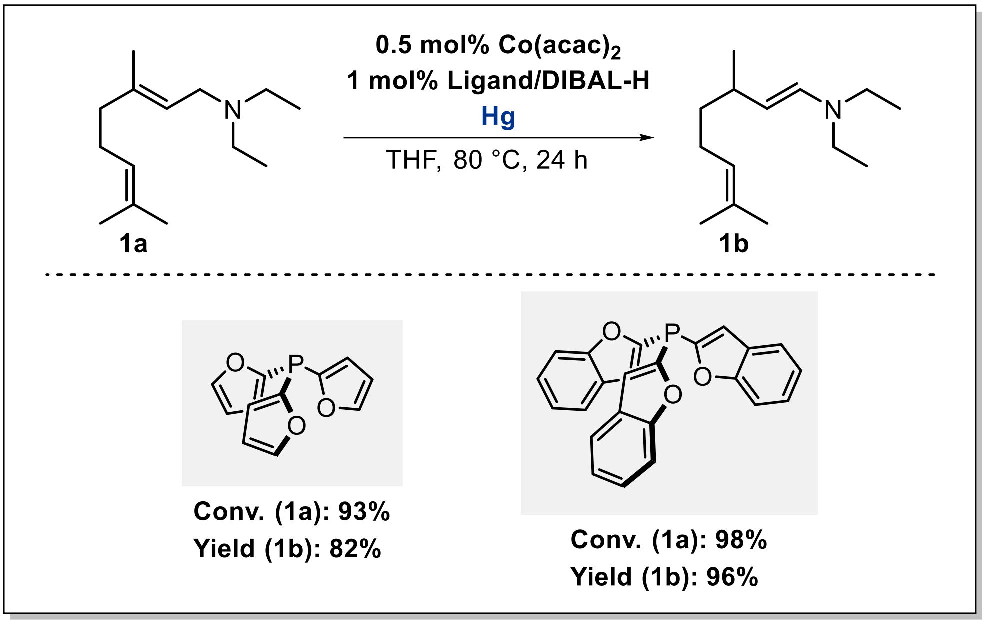

The mercury drop test was conducted in the presence of ligands **L1** and **L15** under the same reaction conditions as described above.

9.4 TEM Measurements

Transmission Electron Microscopy (TEM) was performed on the *in situ* Co catalyst ($Co(acac)_2$, **L15**, and DIBAL-H in THF) as well as on the catalyst after the isomerization reaction using substrate **1a**. The results clearly demonstrated the absence of Co nanoparticles or Co clusters in the solution, as no particles were observed during the measurements under the applied reaction conditions. Furthermore, no significant changes in particle size, morphology, or dispersion were detected between the *in situ* and post-reaction samples of the Co catalyst.

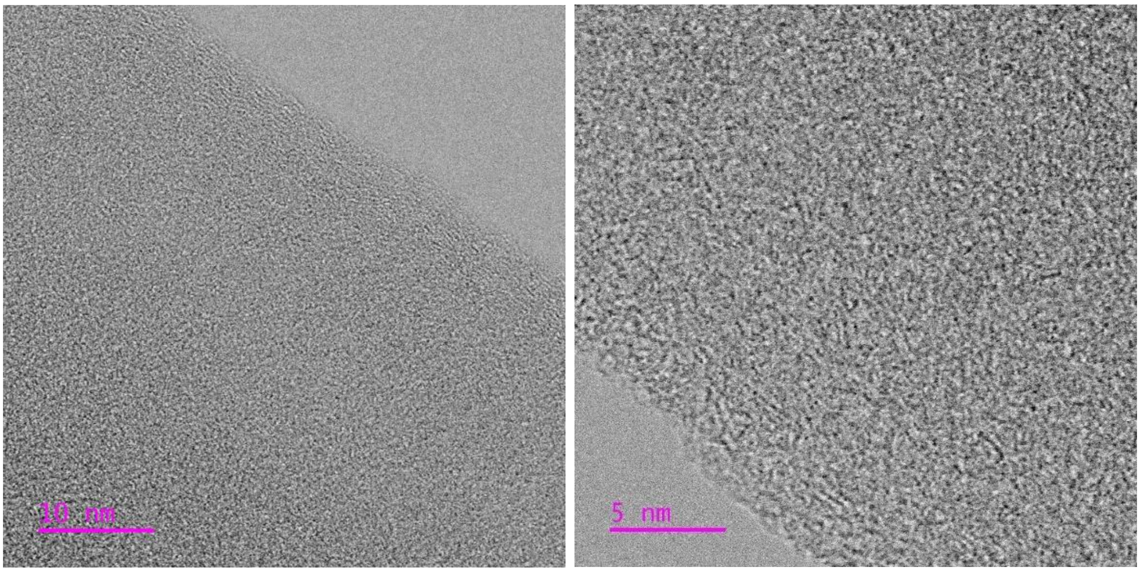

9.5 EPR Measurements

All EPR samples were prepared and measured under inert conditions. First, a defined quantity of **Co-2** was weighed into a 4 mL vial. Then, anhydrous toluene was added, and the complex solution was stirred for approximately 5 min. Afterwards, the complex solution was transferred into an EPR tube, which was sealed with a rubber septum. The subsequent addition of **THF** and **1a** was carried out under anaerobic conditions to ensure that no water was injected.

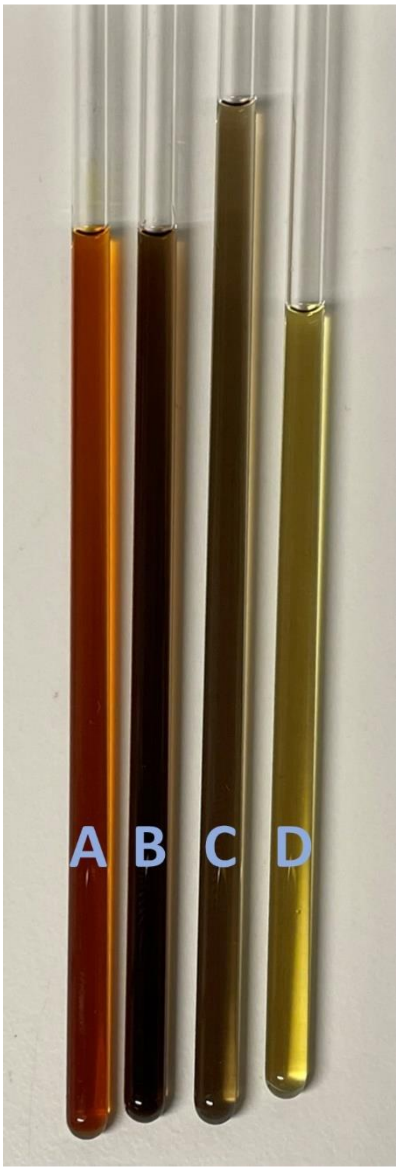

<u>EPR samples after measurement:</u>

A: Co-2 in toluene

B: Co-2 in toluene + THF + 1a (60 °C heating)

C: Co-2 in toluene + THF

D: Co-2 in toluene + 1a

EPR spectra of Co-2: addition of THF

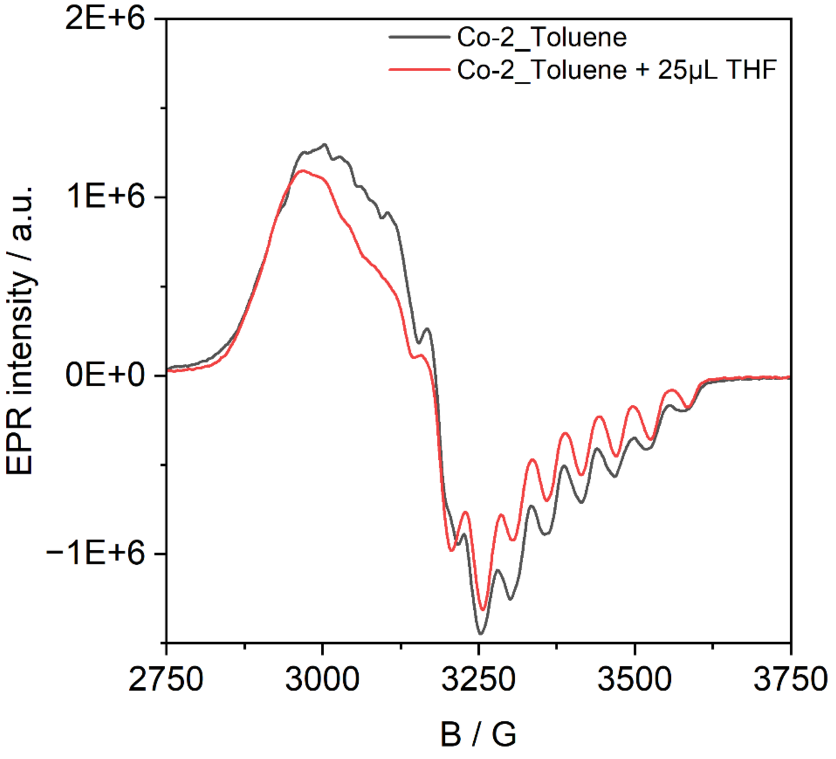

Figure S10: EPR spectra of **Co-2** ($M_{complex}$ = 1588.35 g/mol) in toluene before and after adding 25 μL THF measured at 95 K.

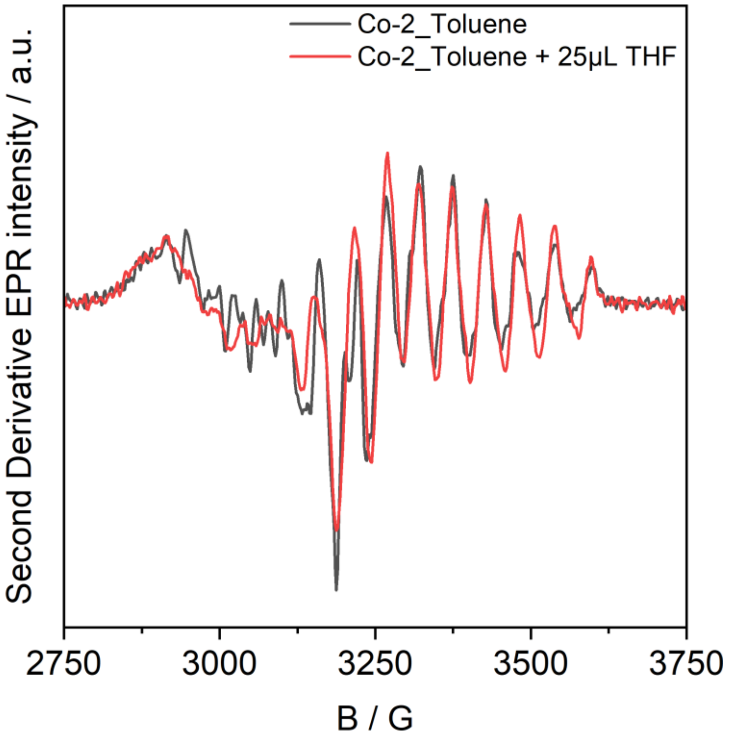

Figure S11: Second derivative EPR spectra of **Co-2** ($M_{complex}$ = 1588.35 g/mol) in toluene before and after adding 25 μL THF measured at 95 K.

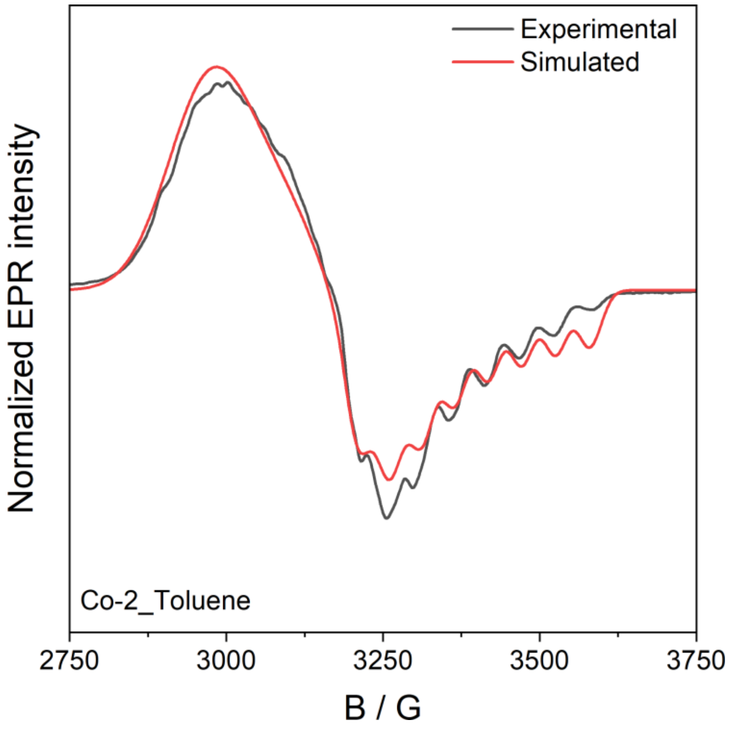

Figure S12: Experimental and simulated EPR spectra of **Co-2** ($M_{complex}$ = 1588.35 g/mol) in toluene.

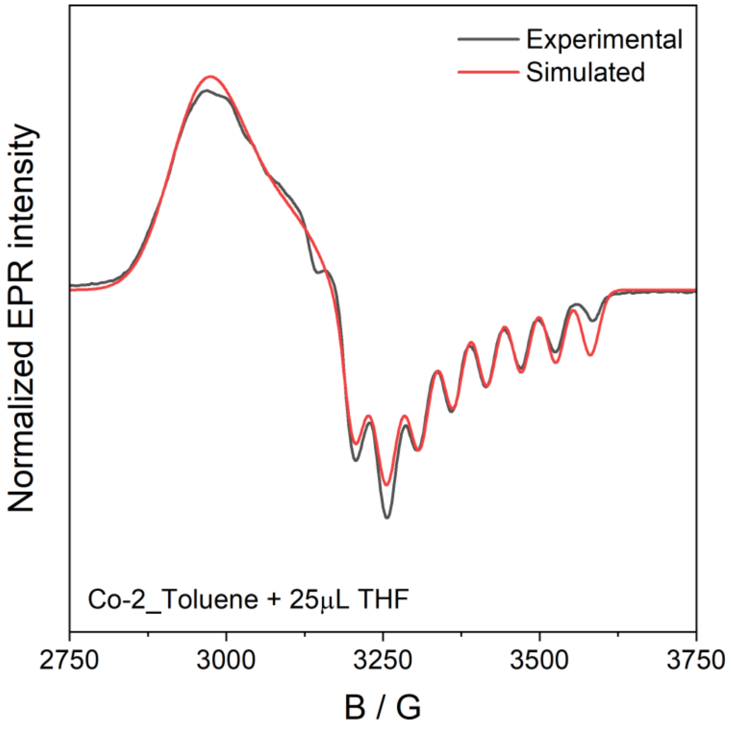

Figure S13: Experimental and simulated EPR spectra of **Co-2** ($M_{complex}$ = 1588.35 g/mol) with THF as additive in toluene.

EPR spectra of Co-2: addition of THF + 1a

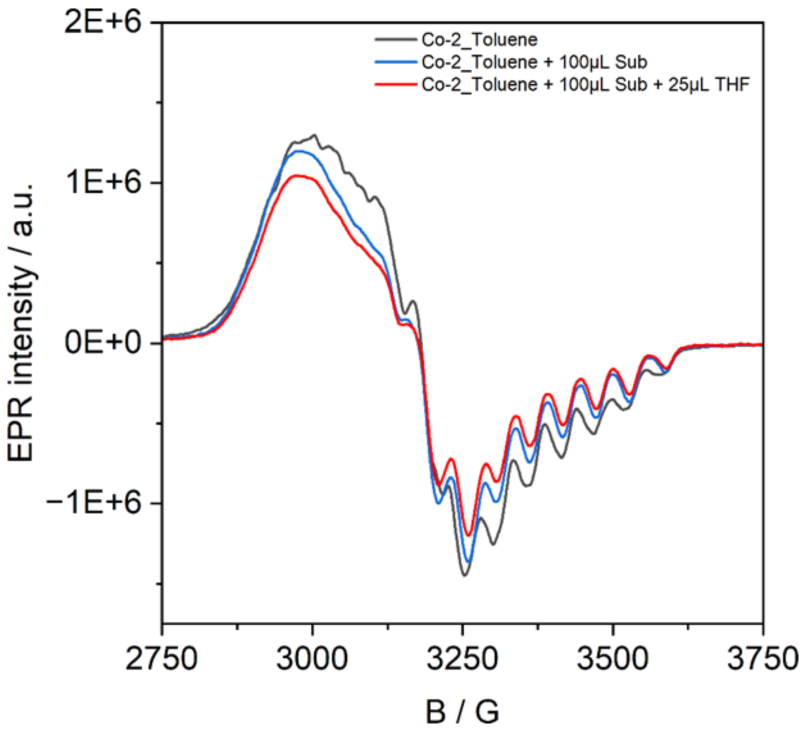

Figure S14: EPR spectra of **Co-2** ($M_{complex}$ = 1588.35 g/mol) in toluene before and after adding 100 µL substrate, a mixture of 100 µL **1a** and 25 µL THF measured at 95K.

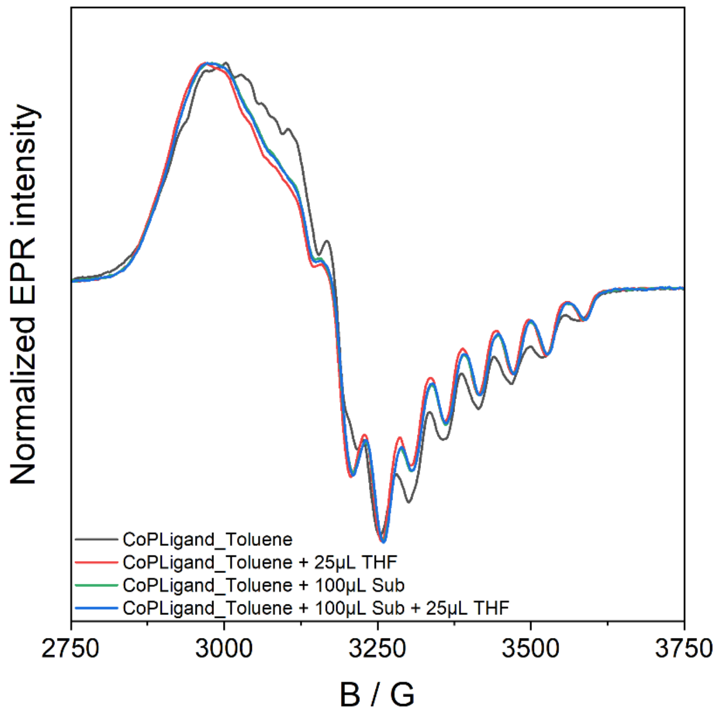

Figure S15: Normalized EPR spectra of **Co-2** ($M_{complex}$ = 1588.35 g/mol) in toluene before and after adding 25µL THF, 100µL **1a**, mixture of 100µL **1a** and 25µL THF measured at 95K.

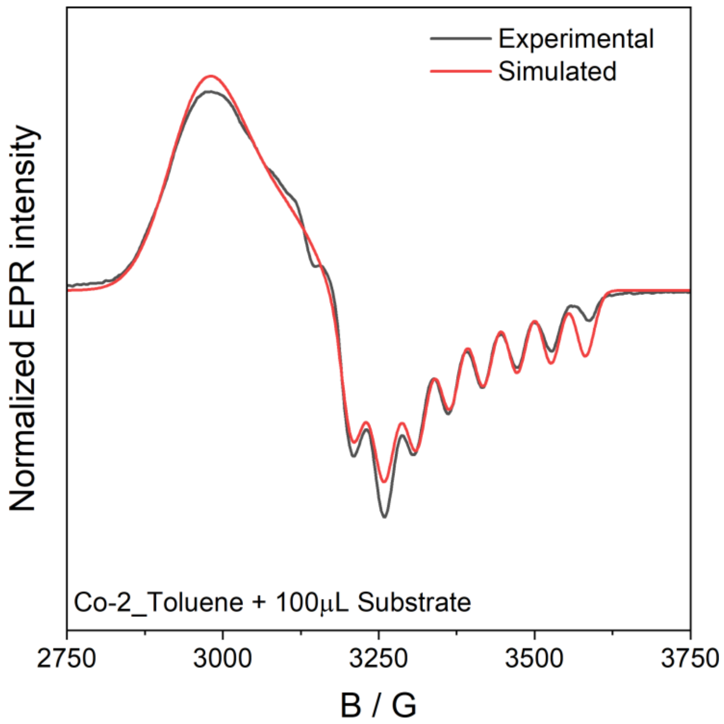

Figure S16: Experimental and simulated EPR spectra of **Co-2** ($M_{complex}$ = 1588.35 g/mol) with **1a** in toluene.

9.6 NMR Measurements

Isomerization of **1a** using **Co-2** - NMR *in situ* experiment:

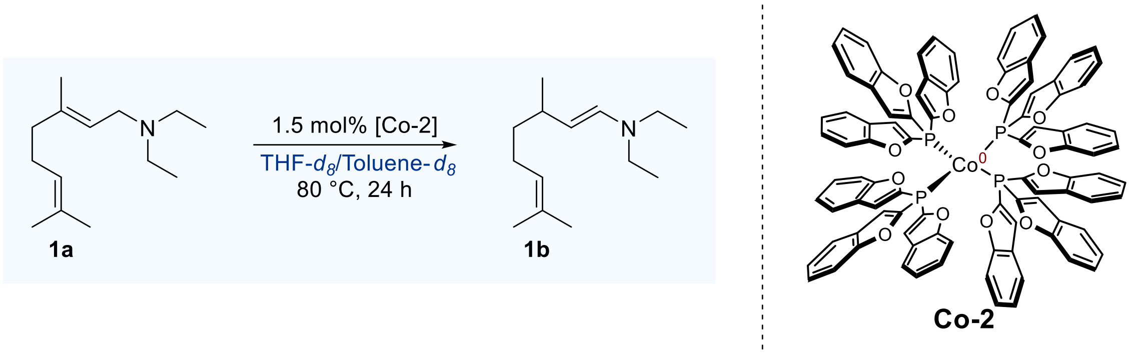

In an argon-filled glovebox, a J-Young tube was charged with **Co-2** (7.1 mg, 4.47 µmol) and a solvent mixture consisting of 0.3 mL THF-d_8 and 0.3 mL toluene-d_8. After a short mixing time, substrate **1a** (62.8 mg, 0.3 mmol) was added and the NMR tube was sealed. The following ^{1}H NMRs were recorded at 60 °C.

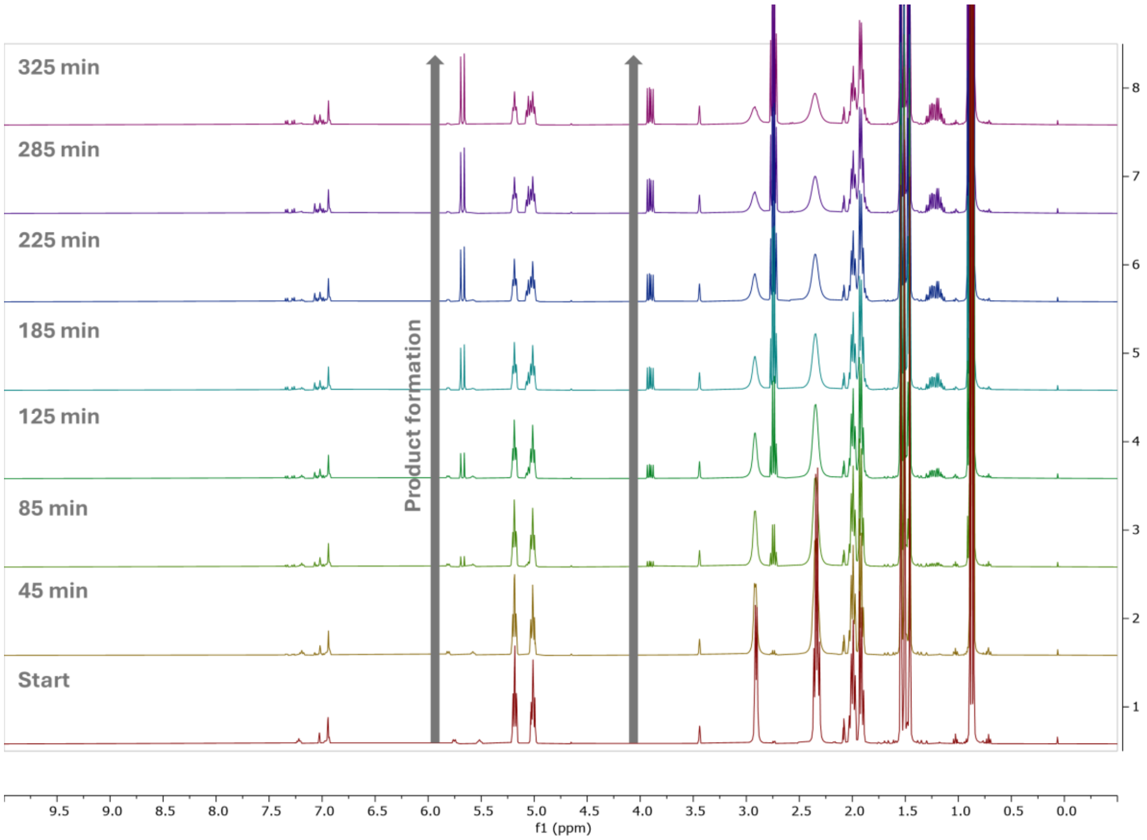

Figure S17: ^{1}H NMR (300 MHz, THF-d_8, 333 K) spectra of the catalysis at different reaction times.

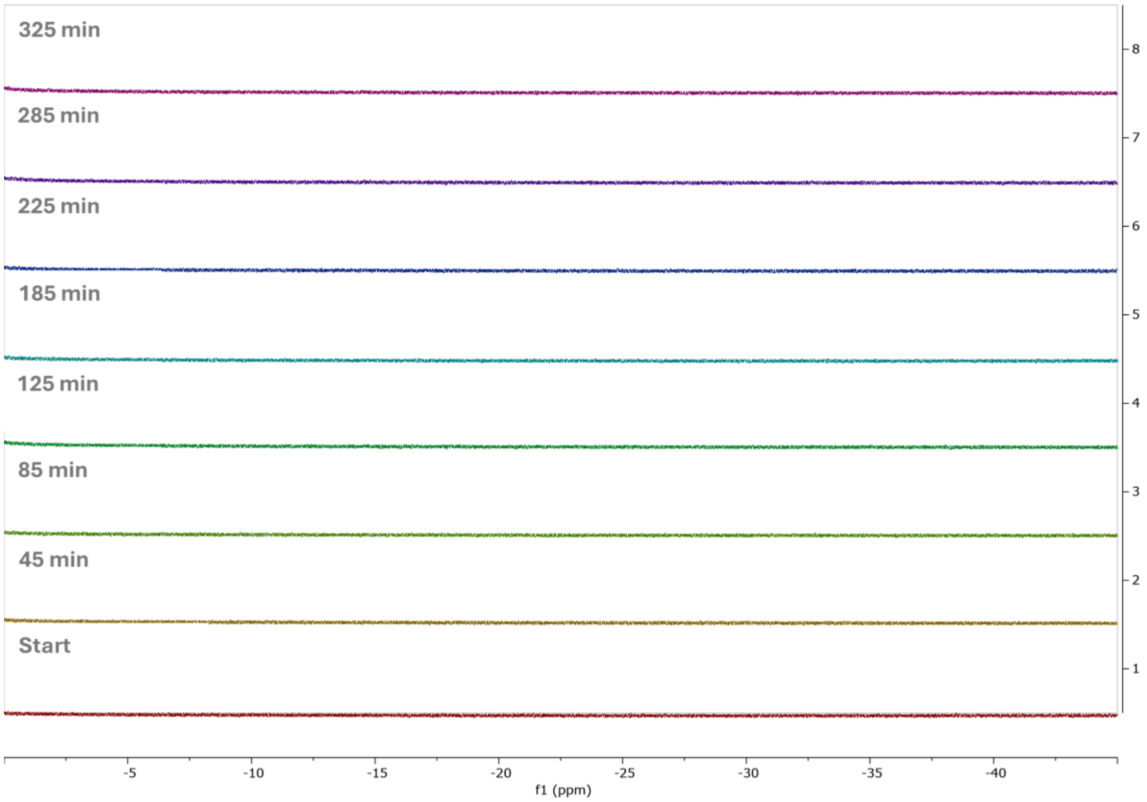

Figure S18: ^{1}H NMR (300 MHz, THF-d_8, 333 K) spectra (0 to -50 ppm) of the catalysis at different reaction times.

No Co-H signal was recorded during the measurement time. Further control experiments, such as using lower reaction temperatures or a broader measurement spectrum (-100 to 100 ppm), did not reveal any Co-H signal either.

^{31}P NMR of Co-2 in THF-d_8:

A J-Young tube was charged with **Co-2** (approx. 5 mg) and 0.6 mL anhydrous THF-d_8 was added. The sample was then gently mixed until the complex dissolved. The following ^{31}P NMR was recorded at room temperature.

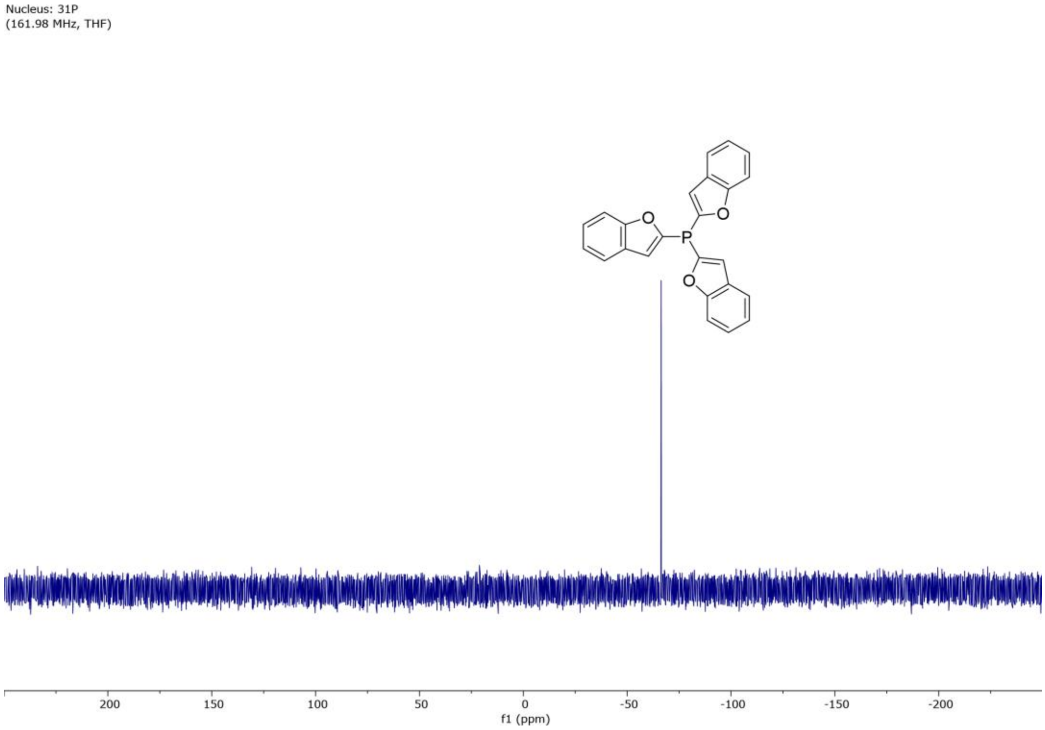

Figure S19: ^{31}P NMR (162.0 MHz THF-d_8, 293 K) spectra of **Co-2**.

HRMS-spectra, Co-2 in THF:

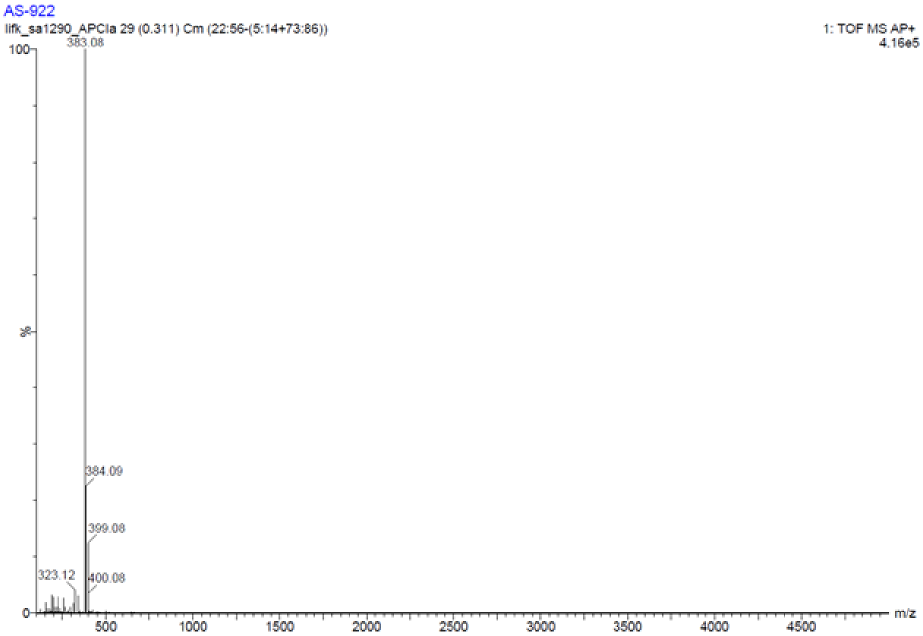

^{31}P NMR, after the catalysis of 1a (with L15):

The isomerization of **1a** was carried out under conditions similar to the optimized reaction parameters (2.5 mmol of **1a**, 0.2 mol% Co(acac)$_2$, 0.4 mol% **L15**, 0.4 mol% DIBAL-H, 1.5 mL THF, 80 °C, 24 h). After catalysis, 0.1 mL of the reaction mixture was transferred into a J-Young tube and 0.5 mL anhydrous toluene-d_8 was added. The following ^{31}P NMR was recorded at room temperature.

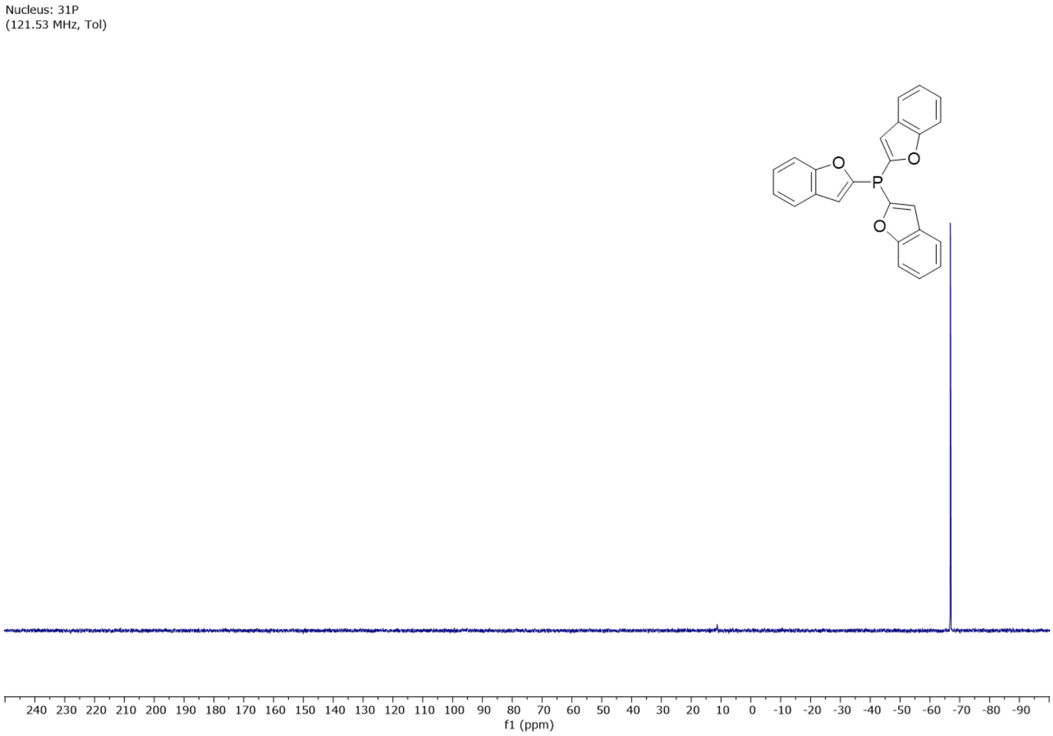

Figure S20: ^{31}P NMR (121.5 MHz, Toluene/THF-d_8, 333 K) spectra of the reaction mixture (**L15**) after the Co-catalyzed isomerization of **1a**.

^{31}P NMR, after the catalysis of 1a (with L1):

The reaction was performed and analyzed according to the described method (with $Co(acac)_2$, **L1** and DIBAL-H).

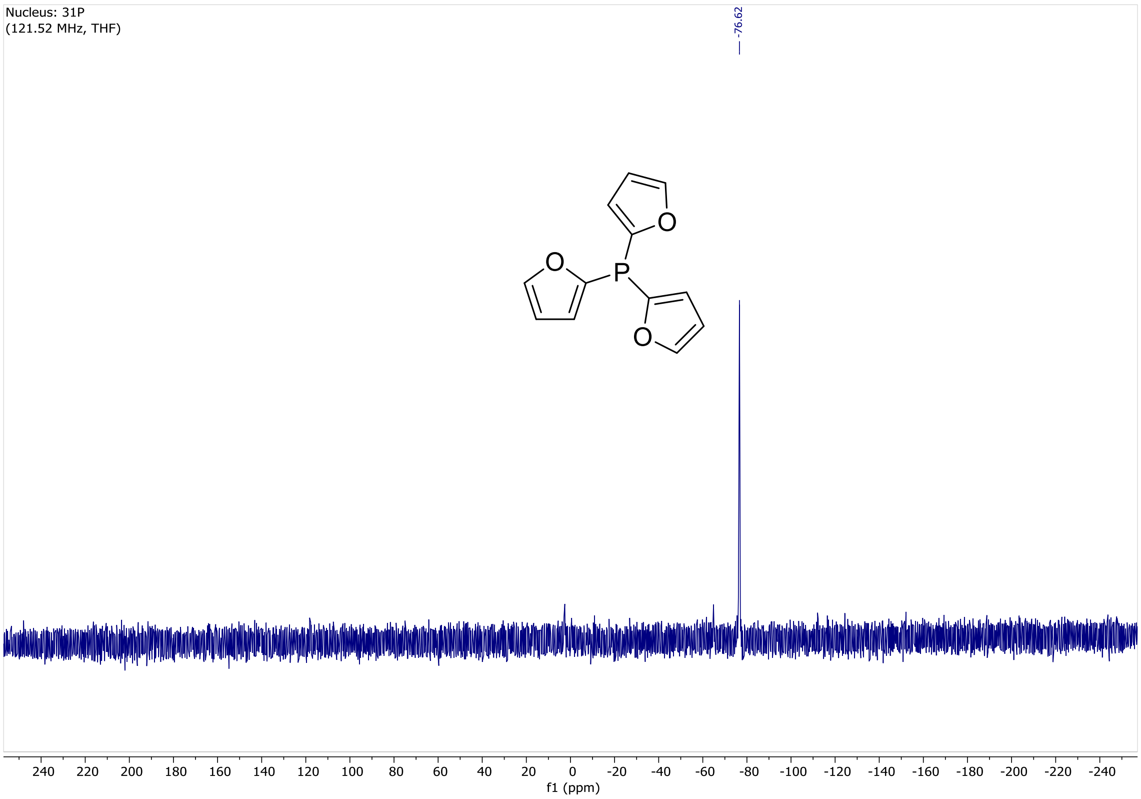

Figure S21: ^{31}P NMR (121.5 MHz, THF-d_8, 333 K) spectra of the reaction mixture (**L1**) after the Co-catalyzed isomerization of **1a**.

HRMS-spectra, after the catalysis of 1a (with Co-2):

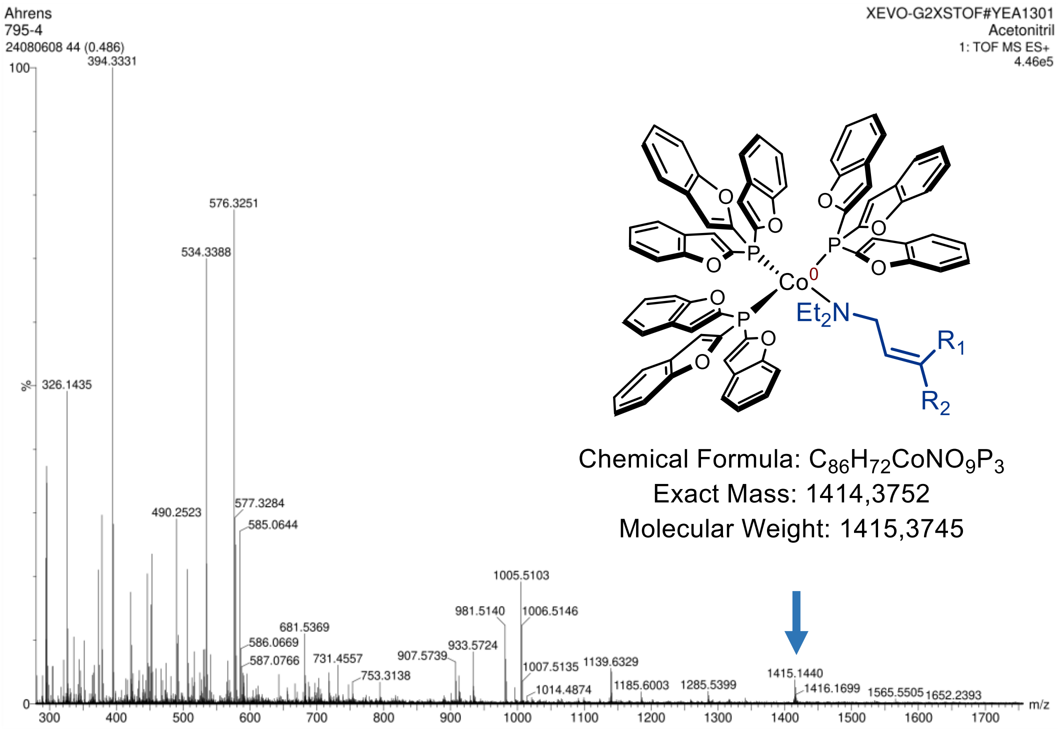

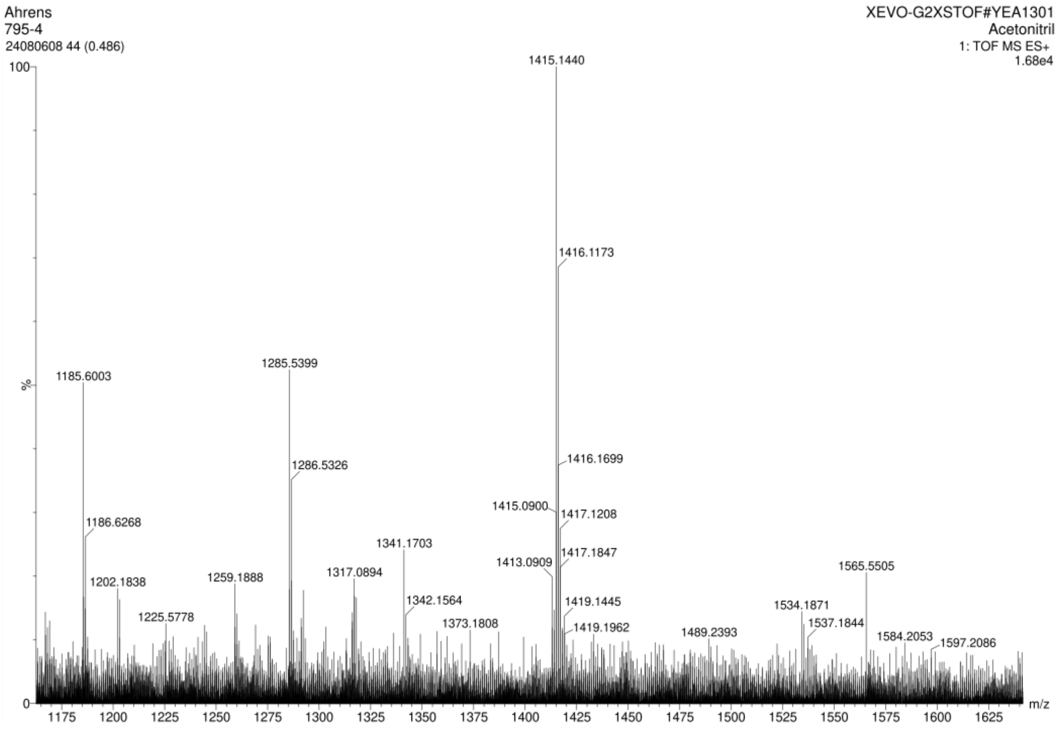

9.7 Radical Trap Experiments

9.7.1 Radical Scavenger

The radical trap experiments with 1,1-diphenylethylene (DPE), 2,6-di-tert-butyl-4-methylphenol (BHT), 2,2,6,6-tetramethylpiperidinyloxyl (TEMPO), galvinoxyl and 2,2-di-phenyl-1-(2,4,6-trinitrophenyl)hydrazin-1-yl (DPPH) were carried out with the same procedure that was used for the initial ligand screening (section 3).

Isomerization of 1a - DPE:

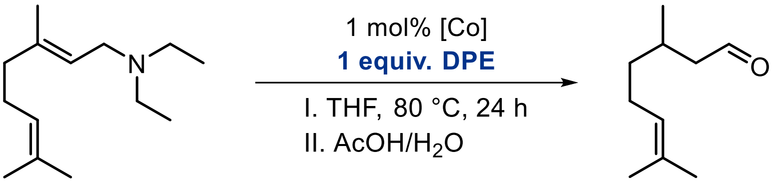

1a **1c**

Conver. (**1a**): 99%
Yield (**1c**): 92%

Under argon atmosphere, a 5 mL Schlenk pressure tube was charged with **Co(acac)₂** (2.6 mg, 10 µmol), **L15** (7.6 mg, 20 µmol) and a stirring bar. Then, 1 mL anhydrous THF was injected followed by the addition of **DIBAL-H** (20 µmol). After 2 min of stirring, first **1a** (209.4 mg, 1 mmol) and then **DPE** (180.3 mg, 1 mmol, 1 equiv.) was added to the catalyst solution. The Schlenk pressure tube was sealed and heated for 24 h. Afterwards, the solvent was removed *in vacuo*, followed by a vacuum distillation of the crude oil. The conversion of **1a** was determined by GC analysis using *n*-hexadecane as an ISTD.

The enamine mixture was then hydrolyzed with 3 mL of a 5% acetic acid solution. After 30 min of stirring, the aldehyde was extracted with diethyl ether followed by washing steps with a sodium carbonate solution and distilled water. The organic solution was dried with sodium sulfate and the solvent was removed *in vacuo*. Finally, the product (**1c**) was purified by column chromatography (142 mg, 0.92 mmol, 92% yield).

Isomerization of 1a - BHT:

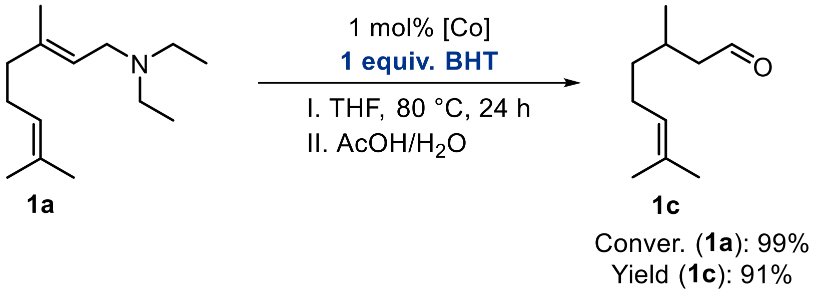

The BHT radical trap experiment followed the same procedure as the DPE experiment. The catalysis was performed with **Co(acac)$_2$** (2.6 mg, 10 µmol), **L15** (7.6 mg, 20 µmol), **DIBAL-H** (20 µmol), **1a** (209.4 mg, 1 mmol) and **BHT** (220.4 mg, 1 mmol, 1 equiv.) in 1 mL anhydrous THF. The conversion of **1a** was determined by GC analysis using *n*-hexadecane as an internal standard.

^{1}H NMR, after distillation of the crude mixture:

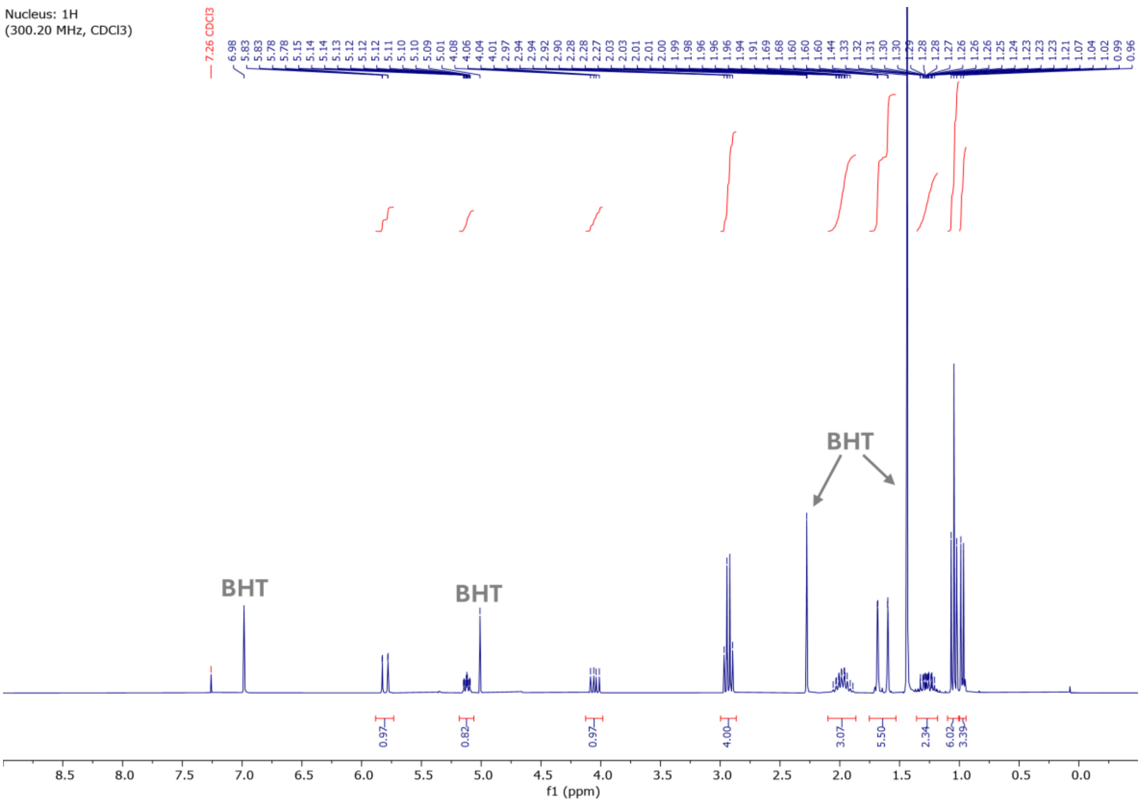

Figure S22: ^{1}H NMR (300 MHz, $CDCl_3$, 293 K) spectrum of the reaction mixture after distillation (separation of the catalyst) - BHT.

Subsequently, the enamine mixture was hydrolyzed following the same procedure as described for DPE.

<u>^{1}H NMR, after hydrolysis and column chromatography:</u>

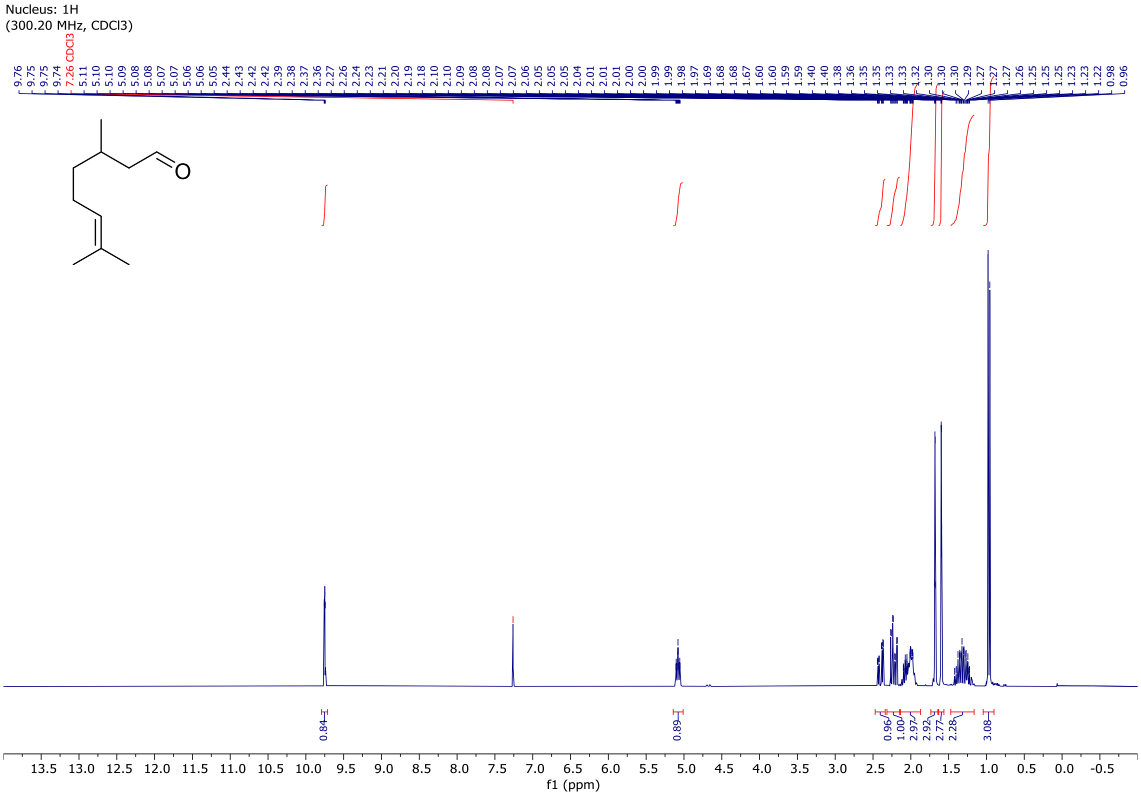

Figure S23: ^{1}H NMR (300 MHz, $CDCl_3$, 293 K) spectrum of the isolated citronellal (**1c**).

Isomerization of 1a - TEMPO:

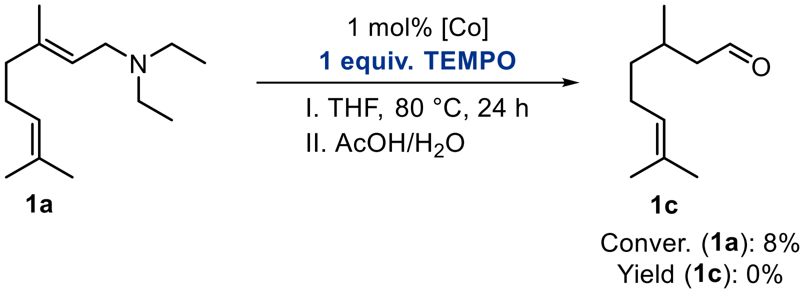

The TEMPO radical trap experiment followed the same procedure as the DPE experiment. The catalysis was performed with **Co(acac)₂** (2.6 mg, 10 µmol), **L15** (7.6 mg, 20 µmol), **DIBAL-H** (20 µmol), **1a** (209.4 mg, 1 mmol) and **TEMPO** (156.3 mg, 1 mmol, 1 equiv.) in 1 mL anhydrous THF. The conversion of **1a** was determined by GC analysis using *n*-hexadecane as an internal standard.

^{1}H NMR, after distillation of the crude mixture:

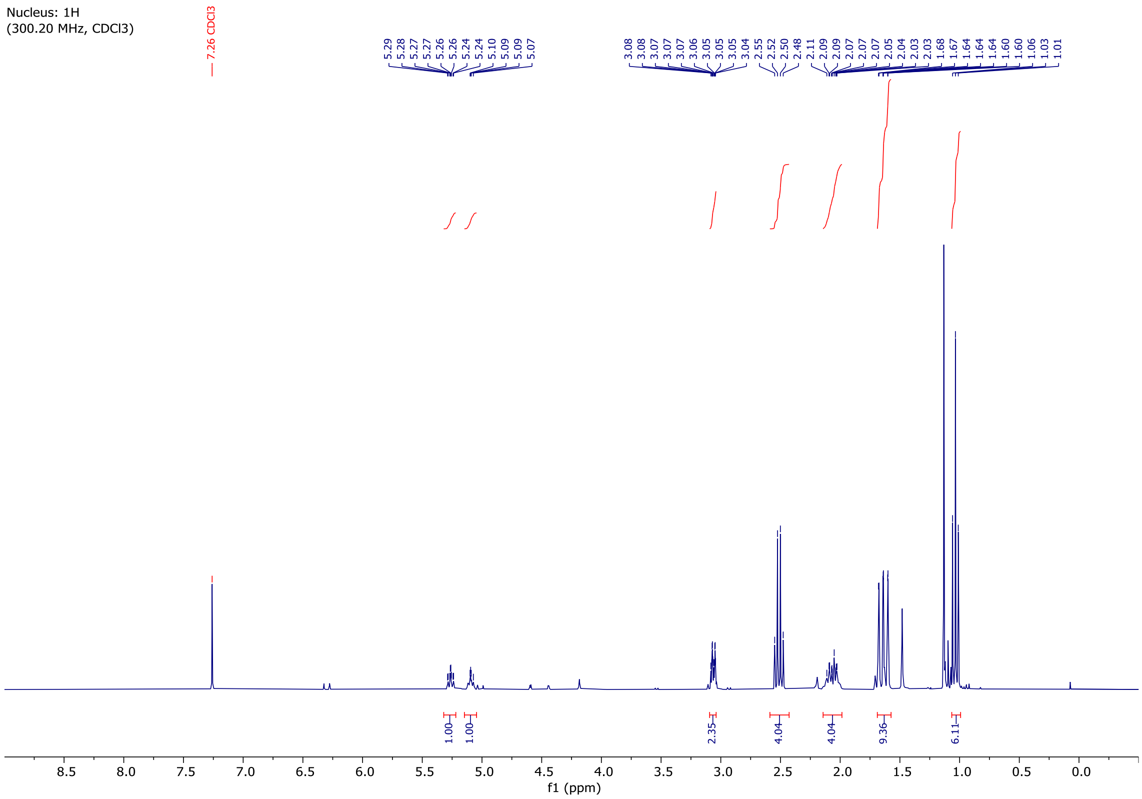

Figure S24: ^{1}H NMR (300 MHz, $CDCl_3$, 293 K) spectrum of the reaction mixture after distillation (separation of the catalyst) - TEMPO.

Isomerization of 1a - galvinoxyl:

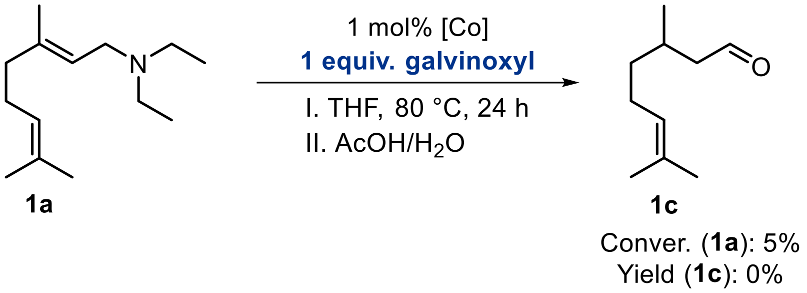

The galvinoxyl radical trap experiment followed the same procedure as the DPE experiment. The catalysis was performed with **Co(acac)$_2$** (2.6 mg, 10 µmol), **L15** (7.6 mg, 20 µmol), **DIBAL-H** (20 µmol), **1a** (209.4 mg, 1 mmol) and **galvinoxy** (421.6 mg, 1 mmol, 1 equiv.) in 2 mL anhydrous THF. The conversion of **1a** was determined by GC analysis using *n*-hexadecane as an internal standard.

^{1}H NMR, after distillation of the crude mixture:

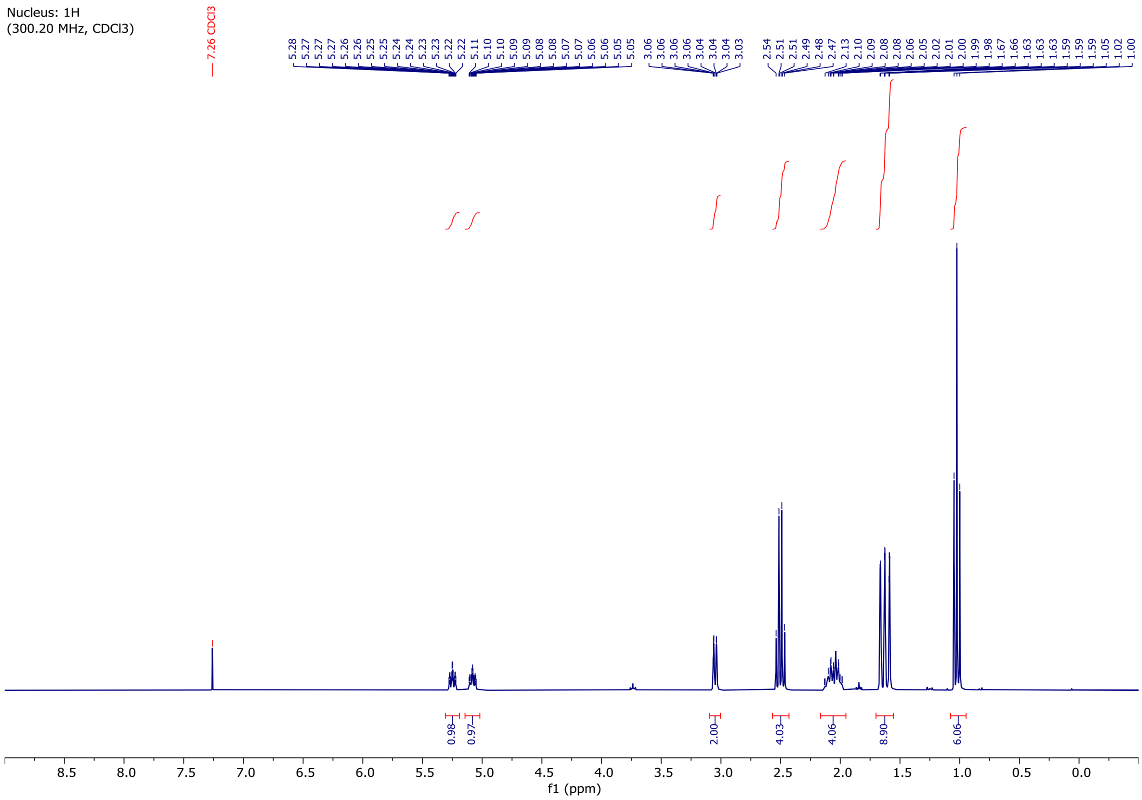

Figure S25: ^{1}H NMR (300 MHz, $CDCl_3$, 293 K) spectrum of the reaction mixture after distillation (separation of the catalyst) - galvinoxyl.

Isomerization of 1a - DPPH:

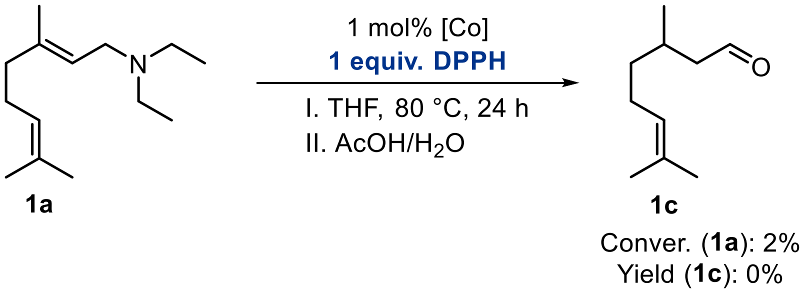

The DPPH radical trap experiment followed the same procedure as the DPE experiment. The catalysis was performed with **Co(acac)₂** (2.6 mg, 10 µmol), **L15** (7.6 mg, 20 µmol), **DIBAL-H** (20 µmol), **1a** (209.4 mg, 1 mmol) and **DPPH** (394.3 mg, 1 mmol, 1 equiv.) in 2 mL anhydrous THF. The conversion of **1a** was determined by GC analysis using *n*-hexadecane as an internal standard.

¹H NMR, after column chromatography:

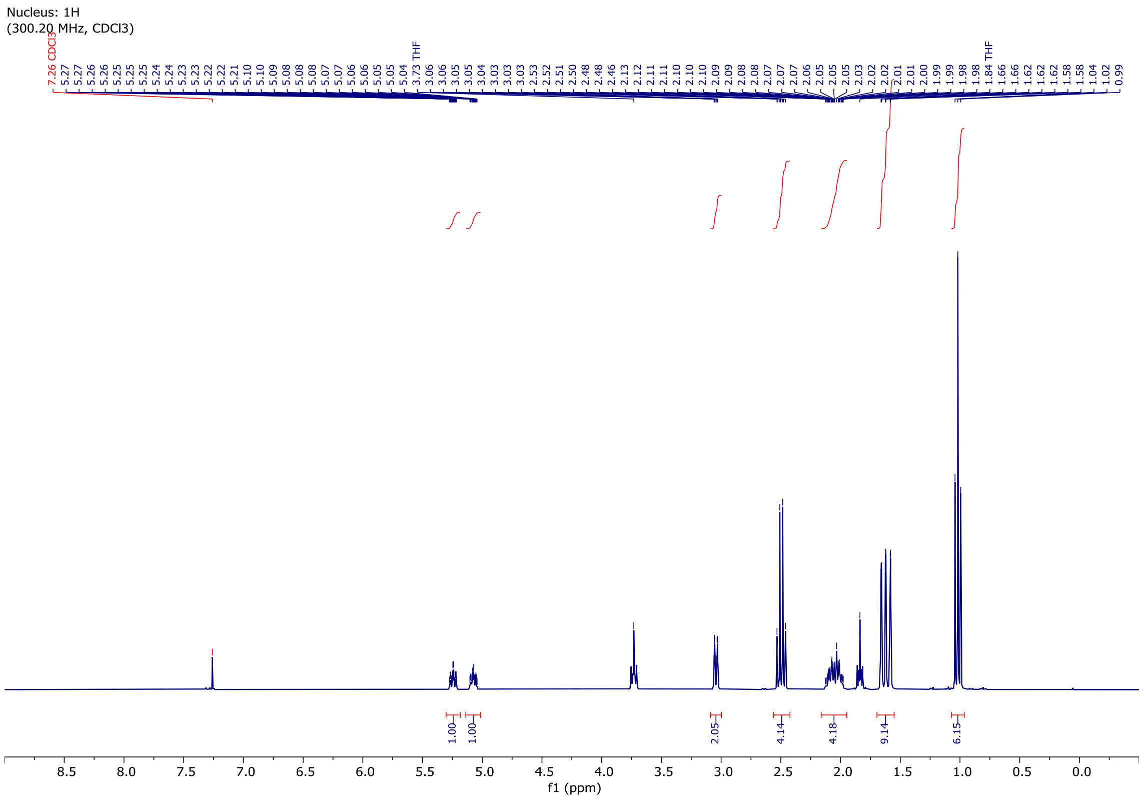

Figure S26: ¹H NMR (300 MHz, $CDCl_3$, 293 K) spectrum of the reaction mixture after column chromatography - DPPH.

9.7.2 Radical clock experiments

Isomerization of 1a with cyclopropylmethyl bromide as additive:

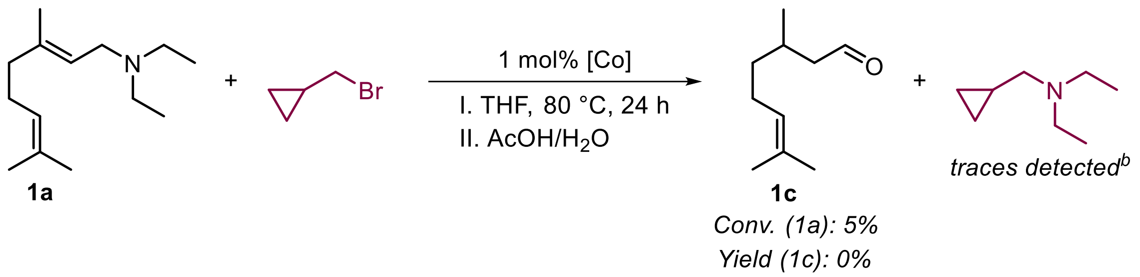

Under argon atmosphere, a 5 mL Schlenk pressure tube was charged with **Co(acac)₂** (2.6 mg, 10 µmol), **L15** (7.6 mg, 20 µmol) and a stirring bar. Then, 1 mL anhydrous THF was injected followed by the addition of **DIBAL-H** (20 µmol). After 2 min of stirring, first **1a** (209.4 mg, 1 mmol) and then **cyclopropylmethyl bromide** (135.0 mg, 1 mmol, 1 equiv.) was added to the catalyst solution. The Schlenk pressure tube was sealed and heated for 24 h.[8] Afterwards, the crude reaction mixture was analyzed by GC-MS as shown in **Figure S27**. No intermediate was identified by GC-MS or NMR.

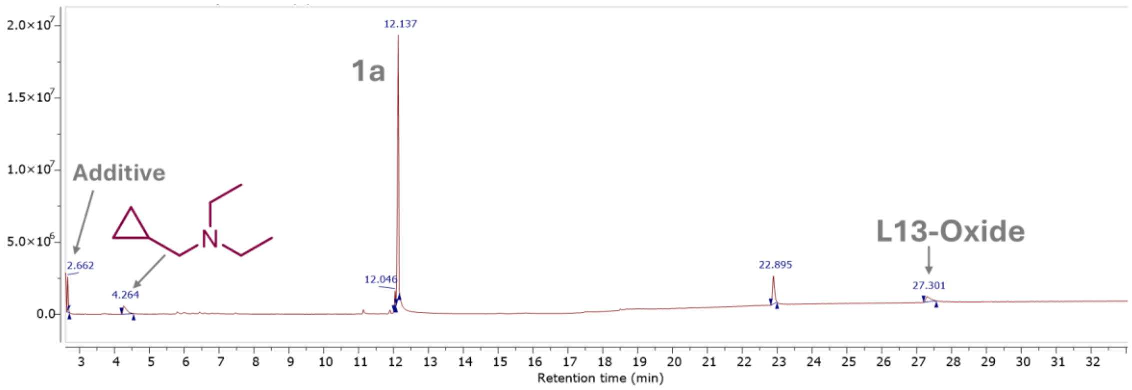

Figure S27: GC-spectrum of the reaction mixture after catalysis.

The ^{1}H NMR of the reaction mixture with cyclopropylmethyl bromide after column chromatography is shown in **Figure S28**.

[8]The conversions were determined by GC using *n*-hexadecane as internal standard.

^{1}H NMR, after column chromatography:

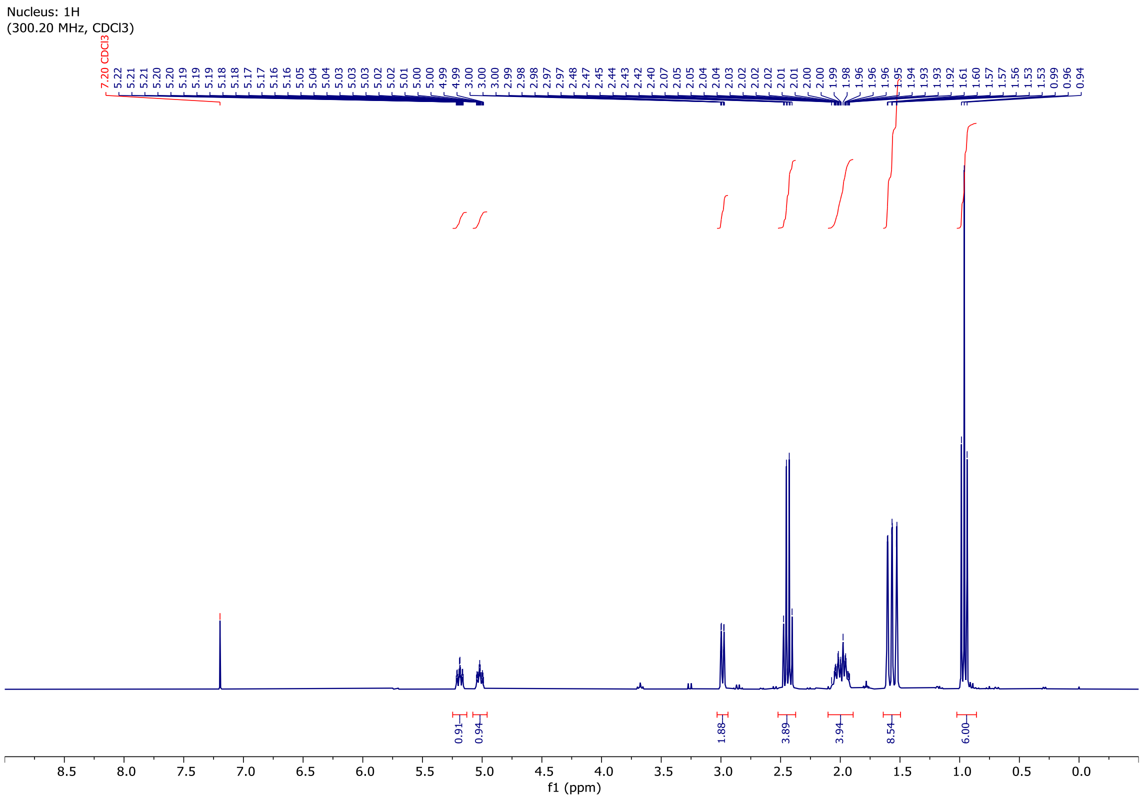

Figure S28: ^{1}H NMR (300 MHz, $CDCl_3$, 293 K) spectrum of the reaction mixture after purification - cyclopropylmethyl bromide.

Synthesis of cyclopropane-allylamines (32a and 33a)

(*E*)-3-cyclopropyl-*N,N*-diethylprop-2-en-1-amine (32a)

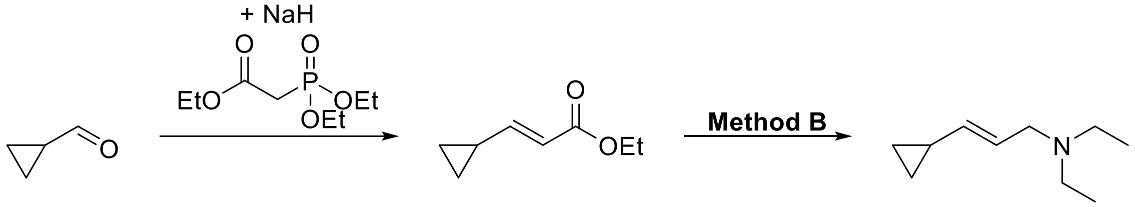

Under argon atmosphere, triethyl phoshonoacetate (3.57 mL, 18.0 mmol) and 50 mL anhydrous THF were charged in a 250 mL Schlenk flask. At -20 °C, NaH (0.720 g, 18.0 mmol) was added and the solution stirred 1 h at room temperature. Afterwards, cyclo-propane-carboxaldehyde (1.12 mL, 15.0 mmol) was slowly added at -20 °C, and the mixture was slowly warmed up and stirred 2 h at room temperature. The reaction mixture was carefully quenched with a saturated aqueous NH_4Cl solution, and the mixture was stirred 1h. Then, the product was extracted with Et_2O (3 x 50 mL), and the solvent was evaporated. Finally, ethyl (*E*)-3-cyclopropylacrylate was purified by vacuum distillation, yielding a colorless oil (1.91 g, 13.7 mmol, 91%).

Next, (*E*)-3-cyclopropylacrylate was charged together with a stirring bar in a Schlenk flask. Then, 100 mL of anhydrous THF was added, and the solution was cooled to -78 °C. DIBAL-H (1 M in THF, 30.0 mmol, 2.2 equiv.) was added dropwise and the solution was then allowed to warm slowly to room temperature. After, full consumption of the *α,β*-unsaturated ester was detected, the reaction was quenched with MeOH and the mixture was stirred for 1 h. The *α,β*-unsaturated alcohol was then extracted with Et_2O, and the organic solution was washed with saturated aqueous NaCl solution. Finally, the crude oil was purified by column chromatography (1.00 g, 10.2 mmol, 75%).

The allylamine synthesis was performed according to the procedure of Craig *et al.* with small modifications.[14] (*E*)-(3-ethoxyprop-1-en-1-yl)cyclopropane (0.95 g, 9.68 mmol) was charged with triphenylphosphine (2.79 g, 10.65 mmol, 1.1 equiv.) and 40 mL THF. Then, *N*-bromosuccinimide (1.90 g, 10.65 mmol, 1.1 equiv.) was added in portions under stir-ring. After 10 min of stirring, diethylamine (1.49 g, 20.3 mmol, 2.1 equiv.) was added dropwise to the reaction mixture and the solution was heated to 60 °C for 2 h. Then, 100 mL Et_2O were added, and the resulting suspension was filtrated. The filtrate was ex-tracted with 1M HCl, washed with Et_2O and made alkaline by the addition of 2M NaOH. The aqueous solution was extracted with Et_2O, and the combined organic solutions were dried with sodium sulphate. After, filtration the solvent was removed *in vacuo* and a

yellowish oil was received. Finally, (*E*)-3-cyclopropyl-*N,N*-diethylprop-2-en-1-amine (**32a**) was obtained by fractional distillation.

Chemical Formula: $C_{10}H_{19}N$

Molecular Weight: 153.2690

Appearance: colorless oil

Isolated Yield: 68% (1.01 g, 6.59 mmol)

^{1}H NMR (300 MHz, $CDCl_3$): δ = 5.55 (dtd, *J* = 15.2, 6.8, 0.6 Hz, 1H), 5.13 (ddt, *J* = 15.3, 8.5, 1.3 Hz, 1H), 3.01 (dd, *J* = 6.8, 1.3 Hz, 2H), 2.50 (q, *J* = 7.2 Hz, 4H), 1.46 - 1.29 (m, 1H), 1.01 (t, *J* = 7.1 Hz, 6H), 0.72 - 0.61 (m, 2H), 0.39 - 0.28 (m, 2H).

^{13}C NMR (75 MHz, $CDCl_3$): δ = 137.22, 124.81, 55.25, 46.54, 13.54, 11.81, 6.70.

GC-MS: *m/z* (%): 154.17 ($[M+H]^+$, 2), 153.16 (M^+, 19), 139.14 (4), 138.14 (39), 124.11 (4), 99.11 (9), 86.11 (20), 84.10 (5), 82.09 (9), 81.10 (100), 80.09 (5), 79.08 (31), 77.06 (4), 73.10 (4), 71.09 (5), 67.08 (4), 58.09 (12), 56.08 (11), 53.07 (12).

^{1}H NMR and ^{13}C NMR spectrum of **32a**.

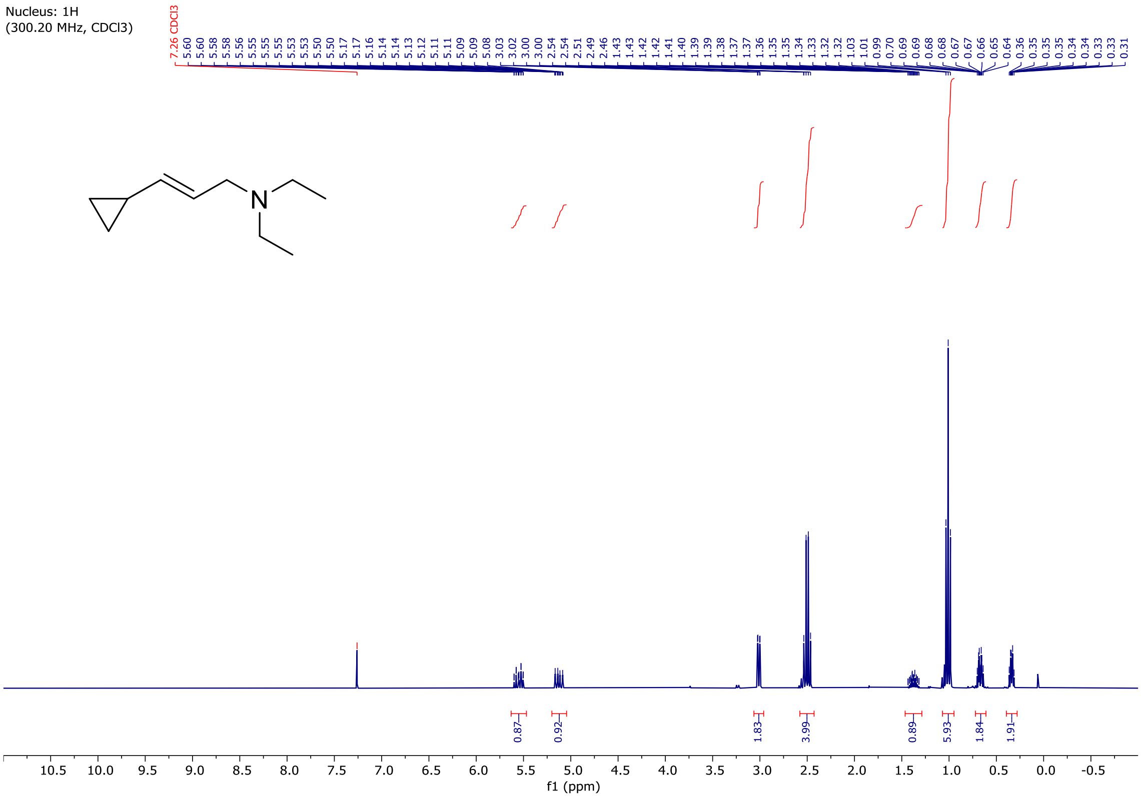

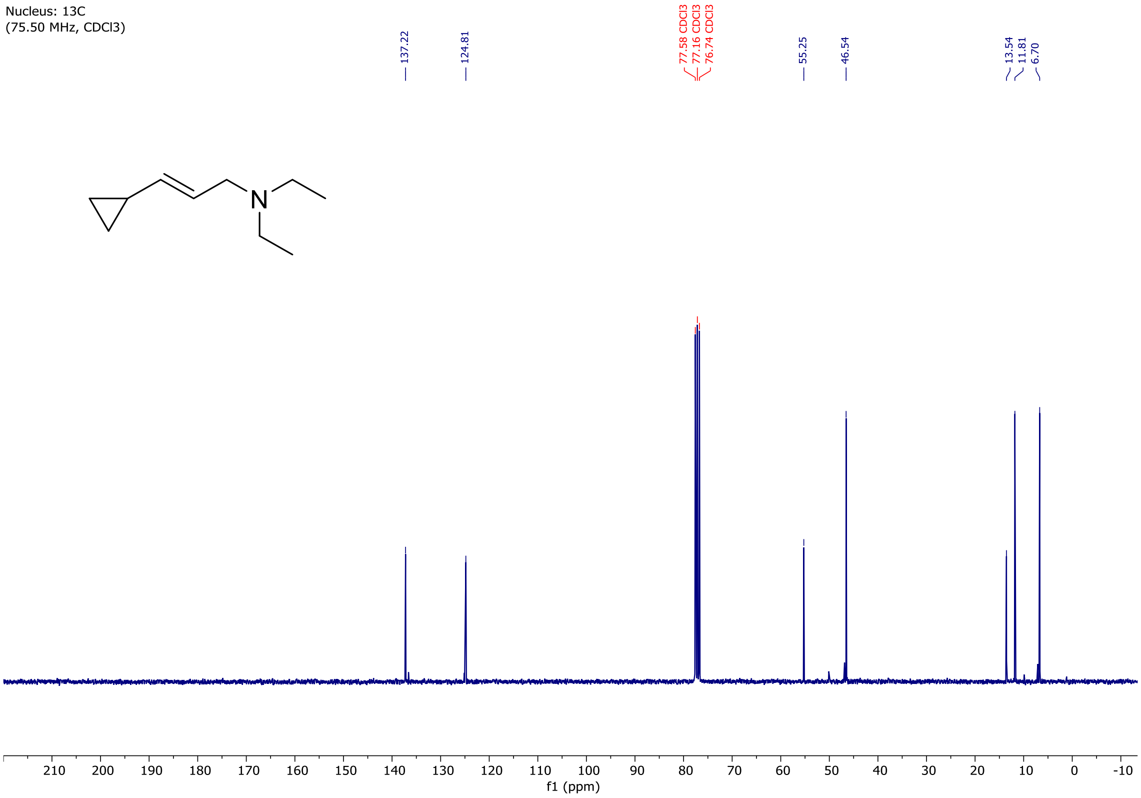

Isomerization of allylamine 32a:

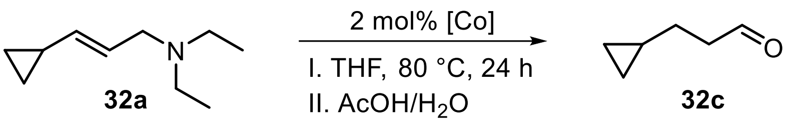

Conv. (32a): 99%
Yield (32c): 86%

The isomerization of **32a** was carried out with the same procedure that was used for the initial ligand screening (section 3). The catalysis was performed with **Co(acac)$_2$** (5.2 mg, 20 µmol), **L15** (15.2 mg, 40 µmol), **DIBAL-H** (40 µmol) and **32a** (153.3 mg, 1 mmol) in 1 mL anhydrous THF. The conversion of **32a** was determined by GC analysis using *n*-hexadecane as an internal standard. After hydrolysis of the enamine and extraction, **32c** was purified by column chromatography (using *n*-pentane/Et_2O).

Isolated Yield: 86% (84.3 mg, 0.86 mmol)

^{1}H NMR (300 MHz, CDCl$_3$): δ = 9.80 (t, *J* = 1.9 Hz, 1H), 2.54 (td, *J* = 7.2, 1.9 Hz, 2H), 1.59 - 1.50 (m, 2H), 0.76 - 0.67 (m, 1H), 0.51 - 0.41 (m, 2H), 0.10 - 0.02 (m, 2H).

^{13}C NMR (75 MHz, CDCl$_3$): δ = 202.96, 44.25, 27.55, 10.64, 4.82.

^{1}H NMR and ^{13}C NMR spectrum of **32c**.[9]

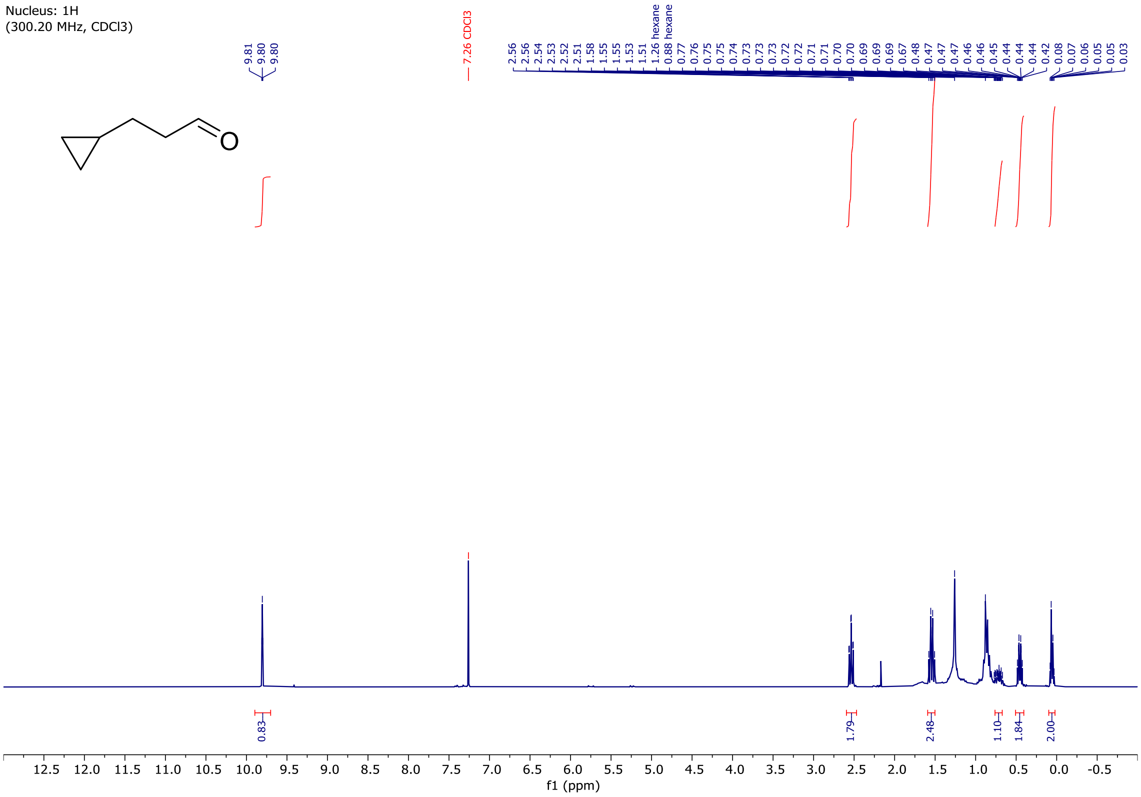

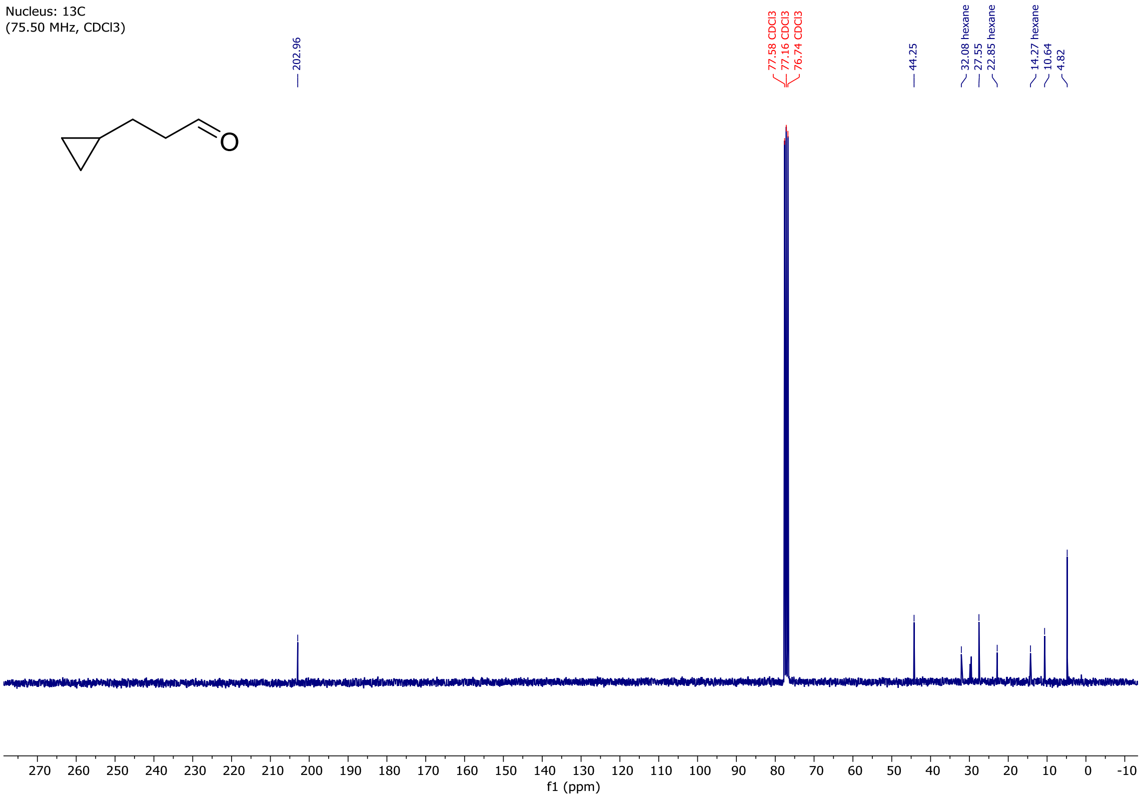

[9]Due to the low boiling point of **32c**, traces of *n*-hexane could not be removed.

Synthesis of 2-cyclopropylidene-*N,N*-diethylethan-1-amine (33a)

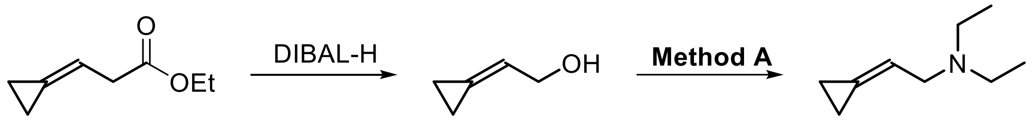

Prepared according to **Method B.** First, ethyl 3-cyclopropylidenepropanoate (1.85 g, 13.20 mmol) was reduced with DIBAL-H (29.0 mmol, 2.2 equiv.), yielding 2-cyclopropylideneethan-1-ol as colorless oil (0.91 g, 10.82 mmol, 82% yield).

Next, **33a** was obtained from the reaction of: 2-cyclopropylideneethan-1-ol (0.91 g, 10.82 mmol), triphenylphosphine (3.12 g, 11.90 mmol, 1.1 equiv.), *N*-bromosuccinimide (2.12 g, 11.90 mmol, 1.1 equiv.) and diethylamine (1.66 g, 22.72 mmol, 2.1 equiv.). Finally, the target product (**33a**) was purified by fractional distillation.

Chemical Formula: $C_9H_{17}N$

Molecular Weight: 139.2420

Appearance: colorless oil

Isolated Yield: 42% (633 mg, 4.54 mmol)

^{1}H NMR (300 MHz, CDCl$_3$): δ = 5.93 - 5.79 (m, 1H), 3.24 (dt, *J* = 6.9, 1.3 Hz, 2H), 2.53 (q, *J* = 7.2 Hz, 4H), 1.04 (t, *J* = 7.2 Hz, 10H).

^{13}C NMR (75 MHz, CDCl$_3$): δ = 124.88, 115.25, 77.58, 77.16, 76.74, 54.27, 46.78, 11.91, 2.54, 1.93.

GC-MS: *m/z* (%): 139.13 (M^+, 13), 125.12 (4), 124.11 (40), 96.08 (6), 94.07 (4), 87.11 (6), 86.12 (100), 84.09 (5), 70.08 (5), 68.07 (4), 67.07 (22), 65.06 (9), 58.08 (17), 56.07 (23), 55.07 (5).

^{1}H NMR, ^{13}C NMR and ^{13}C dept-135 NMR spectrum of **33a**

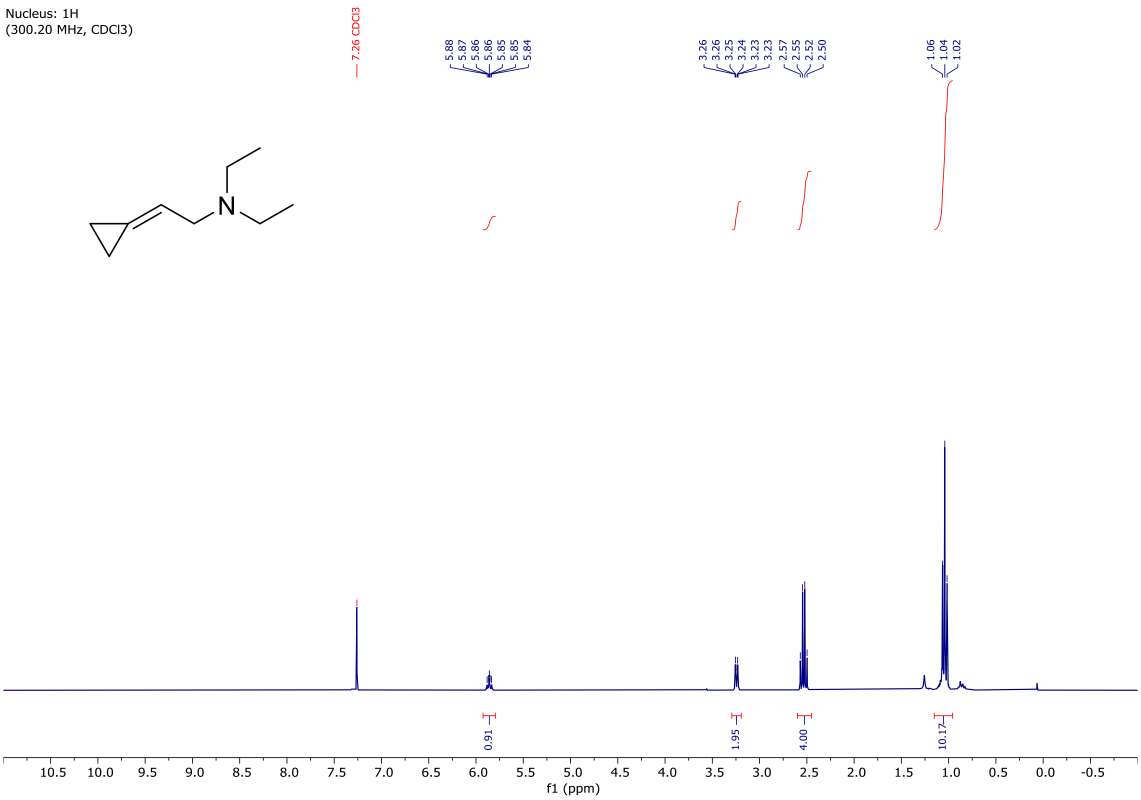

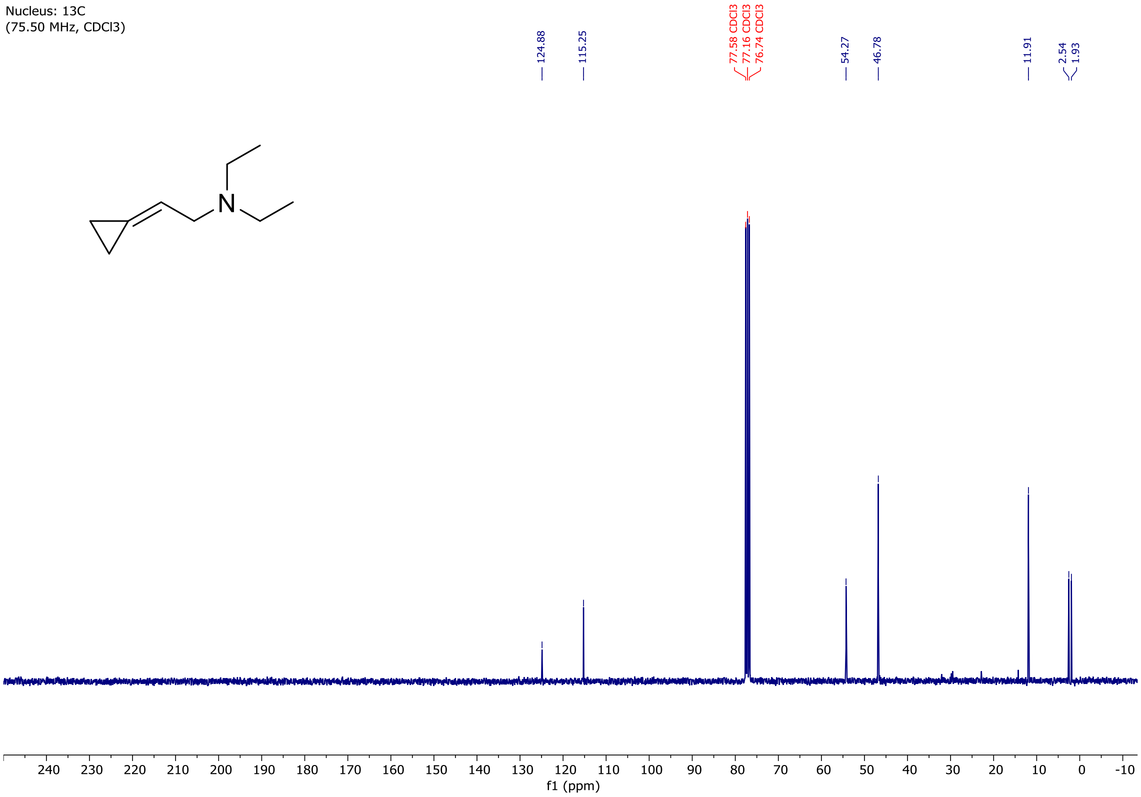

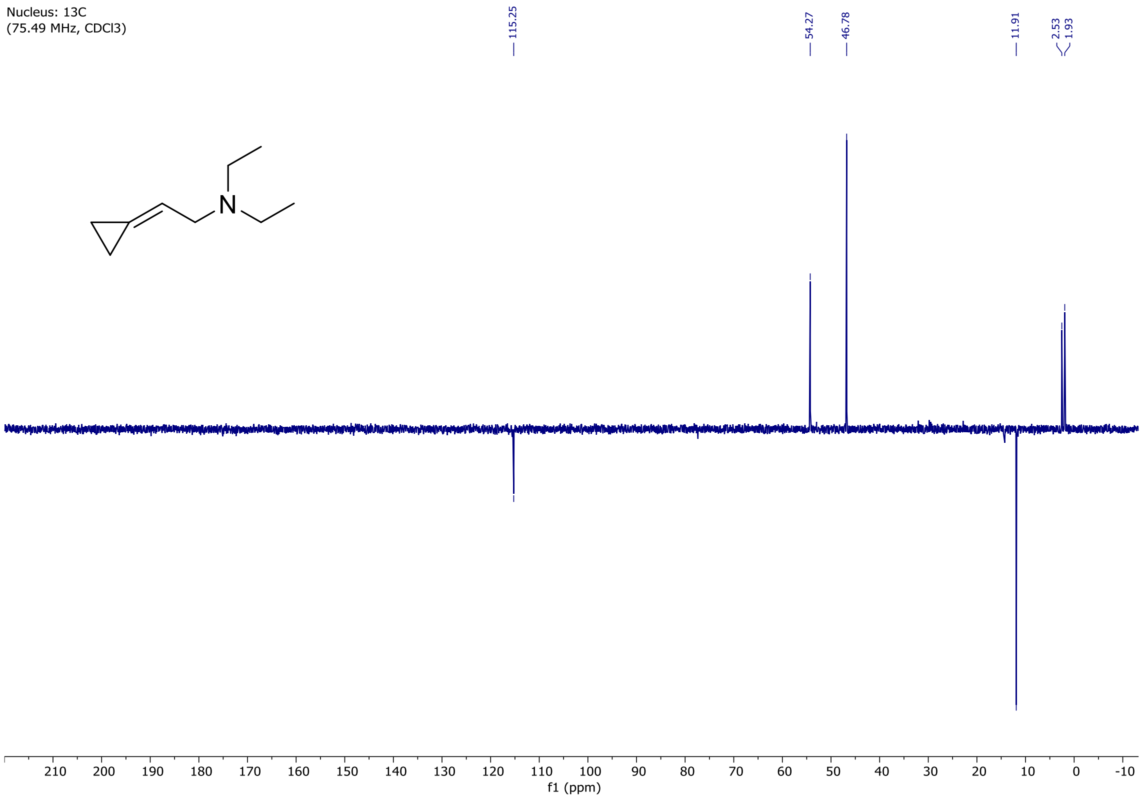
Nucleus: 13C
(75.49 MHz, CDCl3)
115.25
54.27
46.78
11.91
2.53
1.93
N
210 200 190 180 170 160 150 140 130 120 110 100 90 80 70 60 50 40 30 20 10 0 -10
f1 (ppm)

9.8 Deuterium Labelling Experiments

9.8.1 Synthesis of compound 1a-*D2* (34a)

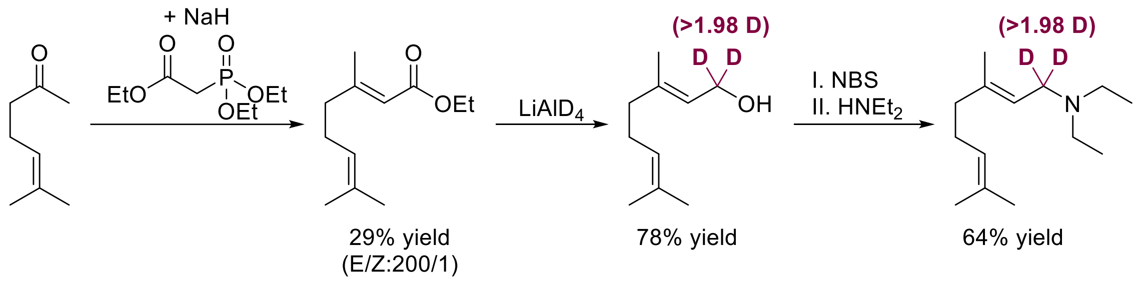

Ethyl (*E*)-3,7-dimethylocta-2,6-dienoate

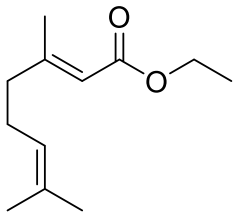

Under argon atmosphere, a 100 mL Schlenk flask was charged with NaH (1.65 g, 52.30 mmol, 1.65 equiv.) and 60 mL anhydrous THF was added. At -20 °C, triethyl phosphonoacetate (10.66 g, 47.54 mmol, 1.50 equiv.) was dropwise added and the reaction mixture was stirred for 30 min. Then, 6-methylhept-5-en-2-one (4.00 g, 31.70 mmol, 1.0 equiv.) was slowly added and the solution stirred overnight. Afterwards, the reaction mixture was quenched with 10 mL saturated aqueous NH_4Cl, followed by extraction with Et_2O (3x150 mL). The received yellowish organic solution was dried with $MgSO_4$ and the solvent was removed *in vacuo*. Next, the crude oil was purified with flash column chromatography (pentane + 0.5% Et_2O), to achieve effective separation of the *E/Z* diastomeres three rounds of flash column were performed.

Chemical Formula: $C_{12}H_{20}O_2$

Molecular Weight: 196.2900

Isolated Yield: 29% (1.805 g, 9.19 mmol)

^{1}H NMR (300 MHz, MeOD-*d4*): δ = 5.66 (tq, *J* = 1.4, 0.7 Hz, 1H), 5.14 (tdt, *J* = 7.3, 2.9, 1.4 Hz, 1H), 4.11 (q, *J* = 7.1 Hz, 2H), 2.63 (dd, *J* = 8.5, 7.1 Hz, 2H), 2.25 - 2.08 (m, 2H), 1.90 (d, *J* = 1.4 Hz, 3H), 1.71 - 1.58 (m, 6H), 1.25 (t, *J* = 7.1 Hz, 3H).

^{13}C NMR (75 MHz, MeOD-*d4*): δ = 167.92, 161.79, 133.06, 124.77, 117.08, 60.56, 34.37, 27.87, 25.88, 25.45, 17.69, 14.64.

^{1}H NMR and ^{13}C NMR spectrum of ethyl (*E*)-3,7-dimethylocta-2,6-dienoate

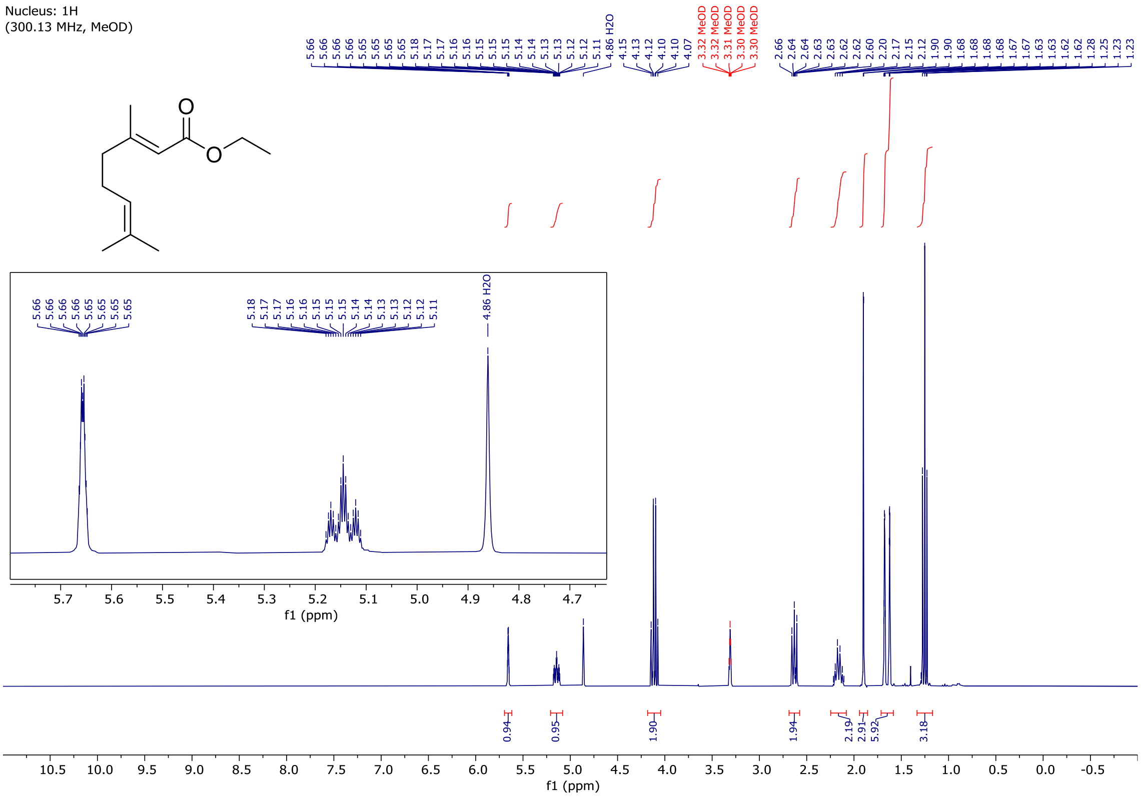

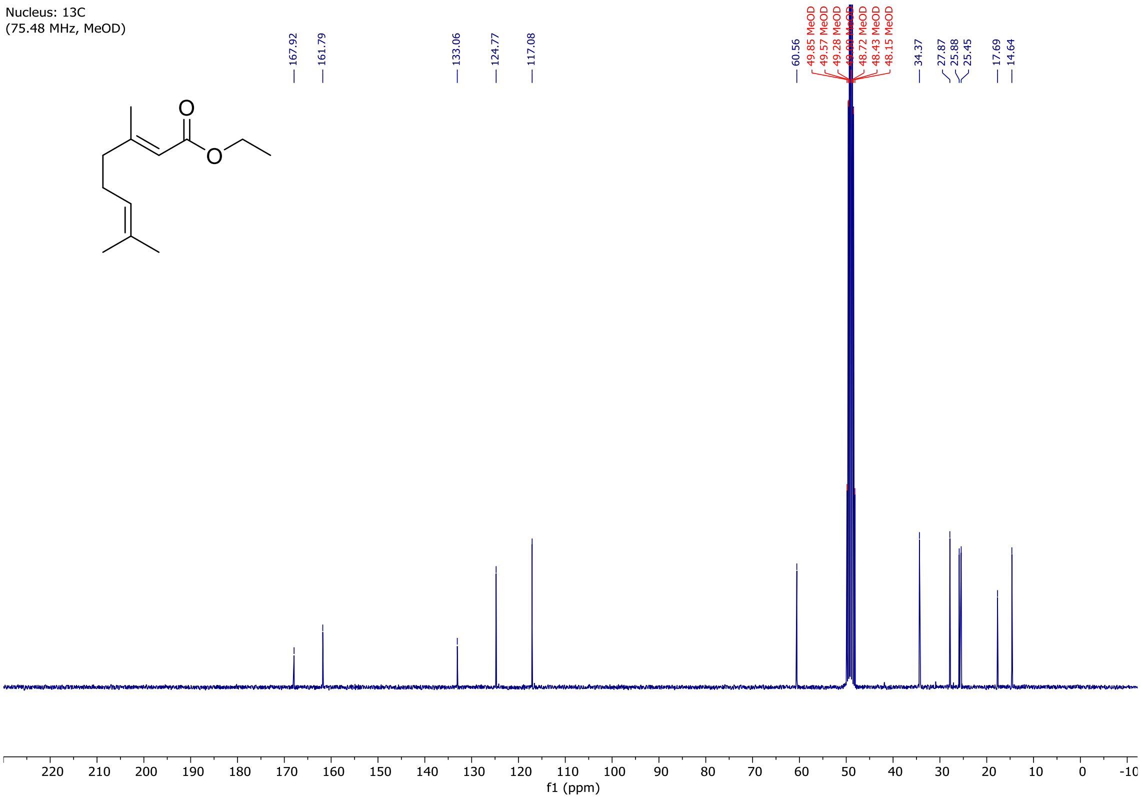

1,1-D_2-geraniol

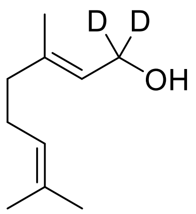

First, lithium aluminum deuteride (115.5 mg, 2.75 mmol, 0.6 equiv.) was weighed into a 100 mL Schlenk flask. Then 20 mL anhydrous THF was added, and the solution was cooled to -78 °C. Next, ethyl (*E*)-3,7-dimethylocta-2,6-dienoate (900 mg, 4.59 mmol, 1.0 equiv.) was dissolved in 5 mL THF and the mixture was dropwise added to the lithium aluminum deuteride solution/suspension. The reaction mixture was stirred at -78 °C for 1 h and then slowly warmed to rt. Afterwards, the reaction mixture was quenched with 10 mL saturated aqueous NH_4Cl, followed by extraction with Et_2O (3x100 mL). The combined organic solutions were dried with $MgSO_4$. Then, the solvent was removed *in vacuo* and a yellow oil was received. Finally, the crude oil was purified with flash column chromatography.

Chemical Formula: $C_{10}H_{16}D_2O$

Molecular Weight: 156.2652

Appearance: colorless oil

Isolated Yield: 78% (559.5 mg, 3.58 mmol)

^{1}H NMR (300 MHz, $CDCl_3$): δ = 5.44 (s, 1H), 5.16 - 5.04 (m, 1H), 2.13 - 2.05 (m, 4H), 1.75 (d, *J* = 1.5 Hz, 3H), 1.71 - 1.66 (m, 3H), 1.64 - 1.56 (m, 3H), 1.13 (s, 1H).

^{13}C NMR (75 MHz, $CDCl_3$): δ = 140.27, 132.62, 124.45, 123.96, 32.09, 26.67, 25.80, 23.56, 17.80.

^{1}H NMR and ^{13}C NMR spectrum of (*E*)-3,7-dimethylocta-2,6-dien-1,1-d_2-1-ol

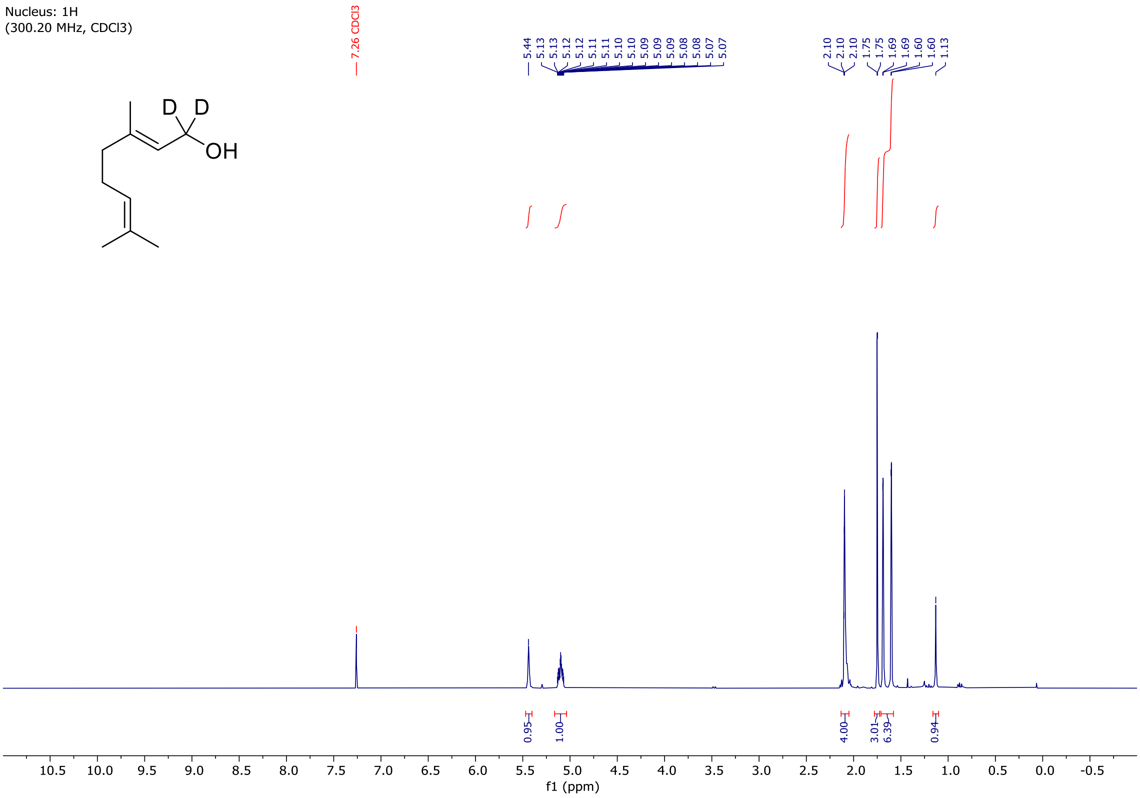

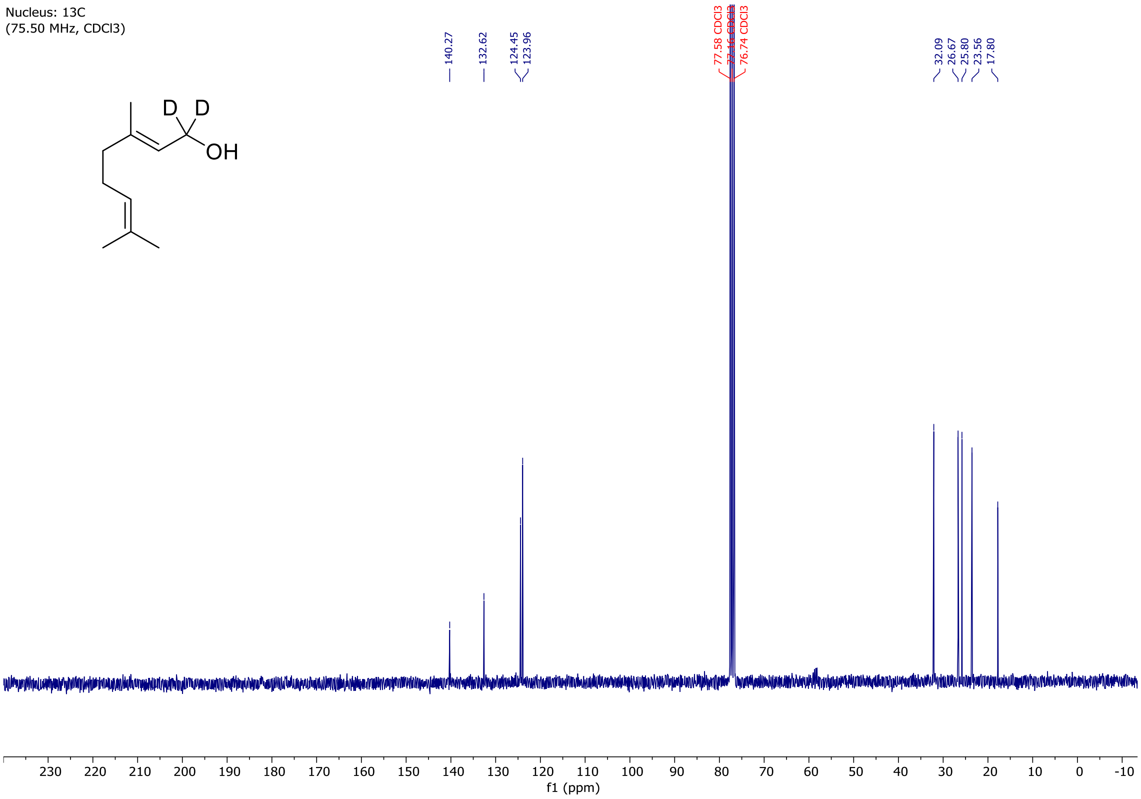

Ethyl (*E*)-3,7-dimethylocta-2,6-dienoate (1a-*D₂*)

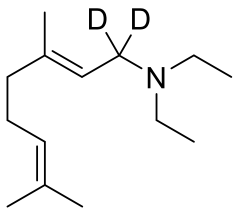

A 50 mL round bottom flask was charged with 1,1-***D*$_2$**-geraniol (559 mg, 3.58 mmol, 1.0 equiv.), triphenylphosphine (1032.0 mg, 3.93 mmol, 1.1 equiv.) and 15 mL THF. Then, *N*-bromo-succinimide (700.4 mg, 3.93 mmol, 1.1 equiv.) was added in portions under stirring. After 10 min of stirring, diethylamine (549.4 mg, 7.51 mmol, 2.1 equiv.) was added dropwise to the reaction mixture and the solution was heated to 60 °C for 2 h. Then, 100 mL Et_2O were added, and the resulting suspension was filtrated. The filtrate was extracted with 1M HCl, washed with Et_2O and made alkaline by the addition of 2M NaOH. The aqueous solution was extracted with Et_2O, and the combined organic solutions were dried with sodium sulphate. After filtration, the solvent was removed *in vacuo*. Finally, the target product was obtained by fractional distillation.

Chemical Formula: $C_{14}H_{25}D_2N$

Molecular Weight: 211.3892

Appearance: colorless oil

Isolated Yield: 64% (484.0 mg, 2.289 mmol)

^{1}H NMR (300 MHz, $CDCl_3$): δ = 5.24 (s, 1H), 5.11 (qt, *J* = 5.3, 3.1 Hz, 1H), 2.50 (q, *J* = 7.2 Hz, 4H), 2.05 (d, *J* = 3.3 Hz, 4H), 1.72 (d, *J* = 1.4 Hz, 3H), 1.70 - 1.64 (m, 3H), 1.63 - 1.58 (m, 3H), 1.02 (t, *J* = 7.1 Hz, 6H).

^{13}C NMR (75 MHz, $CDCl_3$): δ = 137.94, 131.84, 124.29, 122.72, 46.78, 32.34, 26.73, 25.85, 23.69, 17.77, 12.04.

^{1}H NMR and ^{13}C NMR spectrum of compound 1a-D_2

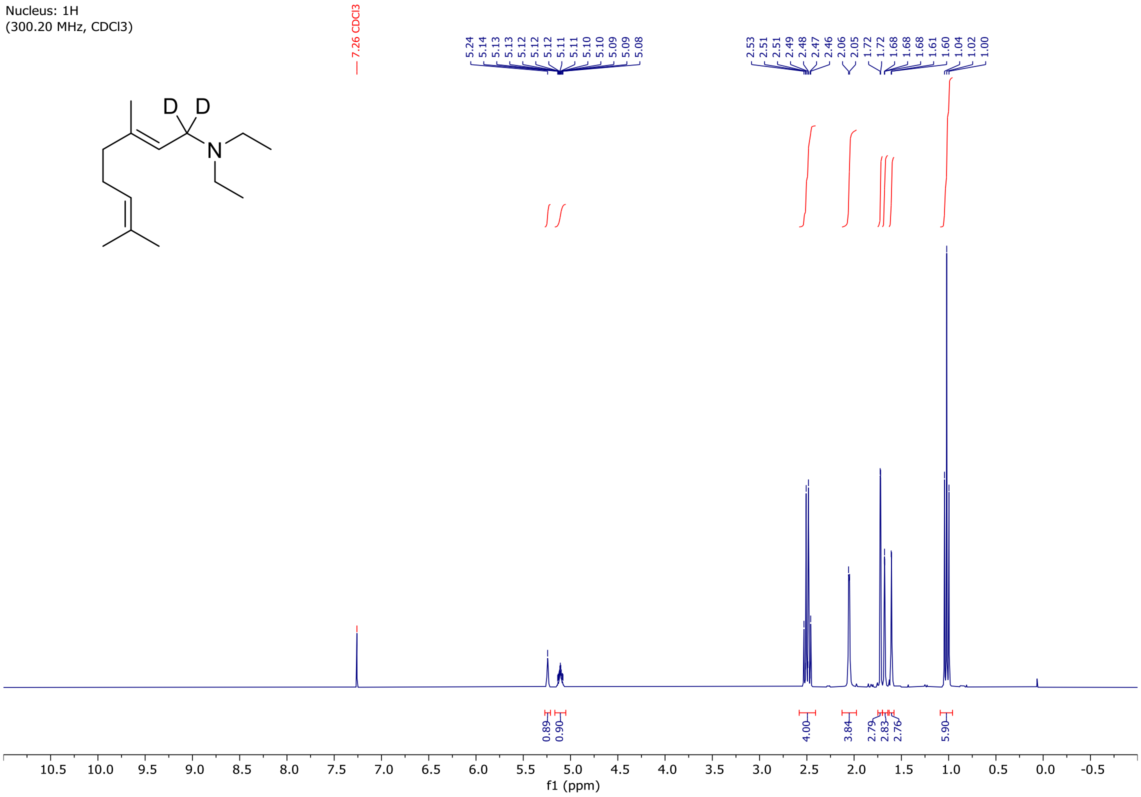

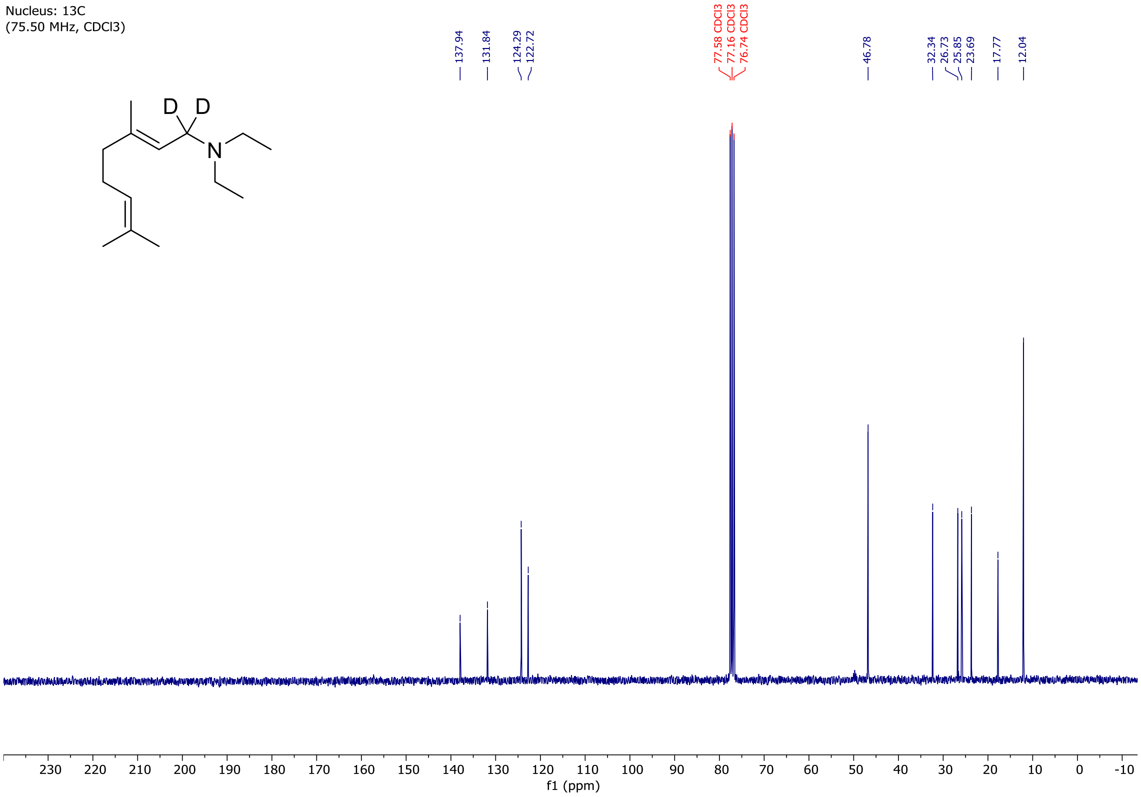

Deuterium incorporation of 1a-*D*$_2$:

- Duplet of -CH_2-NR_2 (**1a**): 3.04 ppm
- Deuterium incorporation in γ-position of **1a-*D*$_2$**: >1.98 D

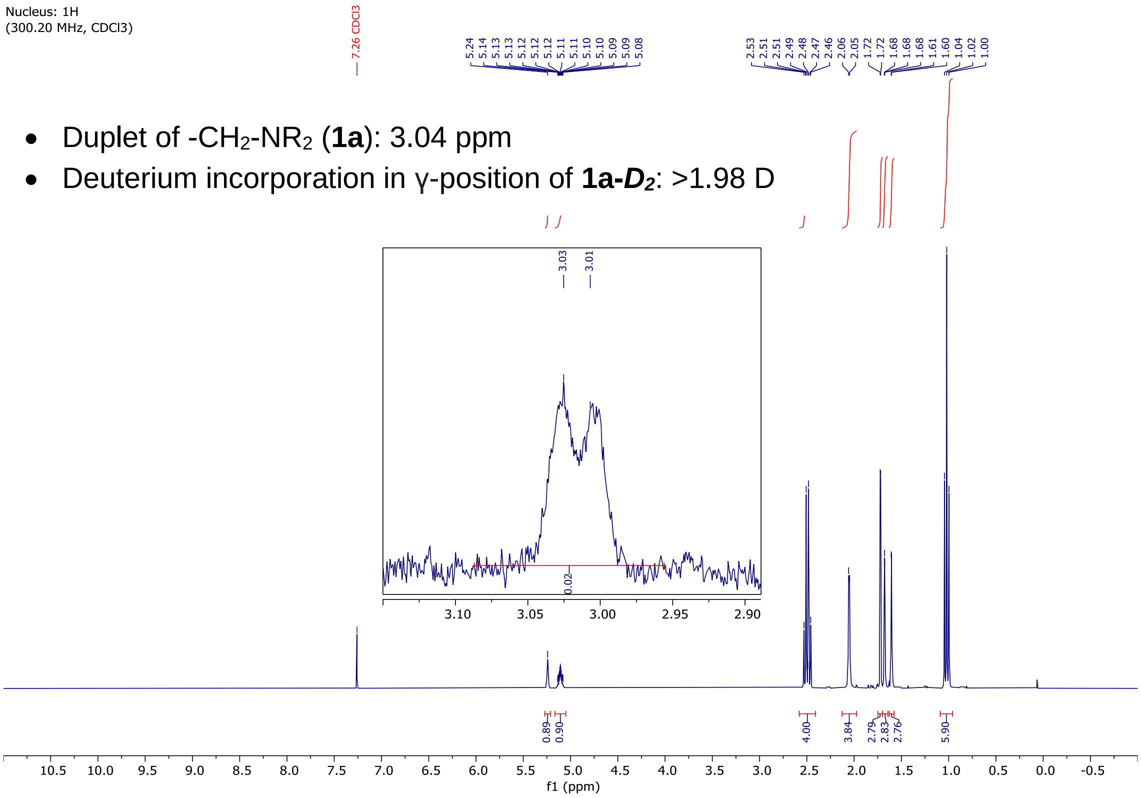

Comparison: ^{1}H NMR spectra of 1a and 1a-*D*$_2$.

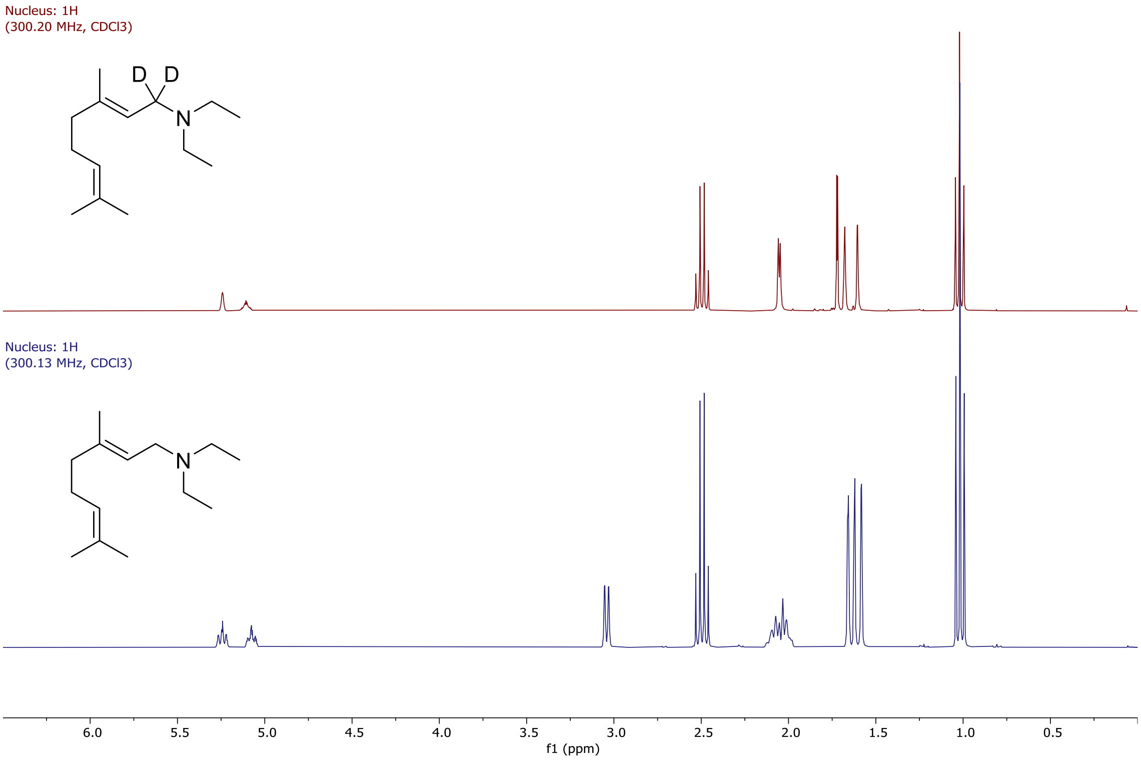

9.8.2 Isomerization of 1a using DIBAL-D as reductant

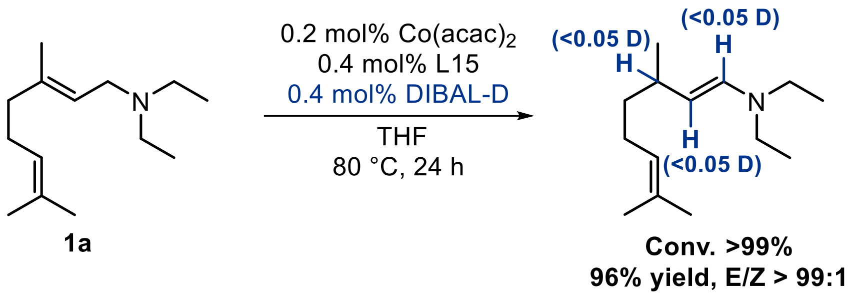

Under argon atmosphere, a 5 mL Schlenk pressure tube was charged with **Co(acac)$_2$** (1.3 mg, 5 µmol), **L15** (3.8 mg, 10 µmol) and a stirring bar. Then, 1 mL anhydrous THF was injected followed by the addition of **DIBAL-D** (10 µmol). Afterwards, **1a** (523.5 mg, 2.5 mmol) was added to the catalyst solution. The Schlenk pressure tube was sealed and heated for 24 h. After the reaction mixture cooled down to rt, the solvent was removed *in vacuo*. Finally, a vacuum distillation of the crude oil was performed, and the enamine (**1b**) was obtained as a colorless oil (502.5 mg, 2.4 mmol, 96% yield).

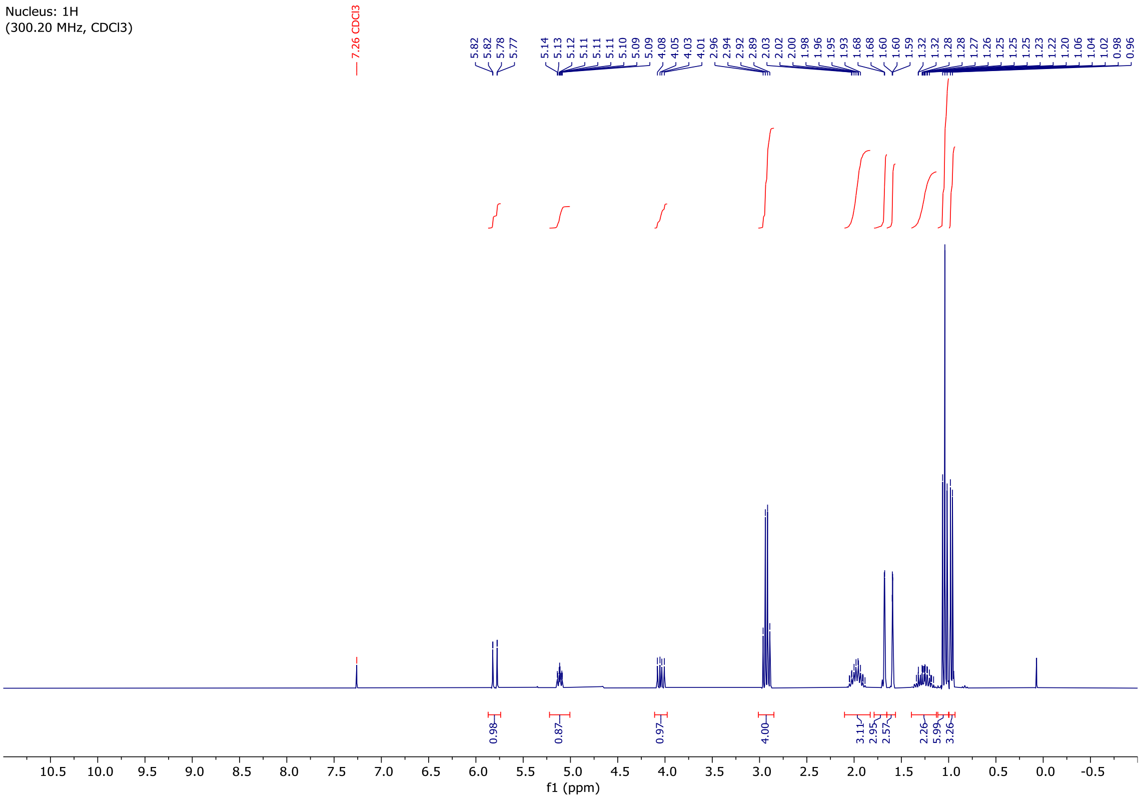

Figure S29: ^{1}H NMR (300 MHz, $CDCl_3$, 293 K) spectrum after distillation of the reaction mixture using DIBAL-D.

9.8.3 Isomerization of 1a in THF-d_8

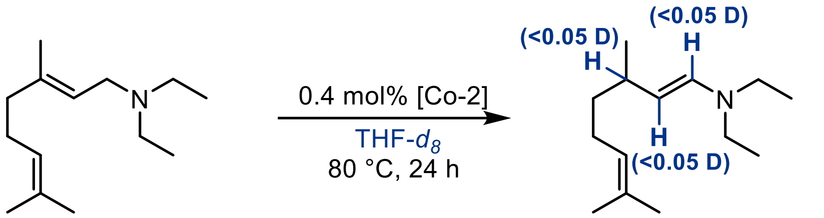

1a

Conv. >99%
97% yield, E/Z > 99:1

In an argon filled glovebox, **Co-2** (9.6 mg, 6 µmol) was weighed into a 5 mL Schlenk pressure tube. Then, 0.8 mL anhydrous THF-d_8 was added, and the solution was stirred for 5 min. Afterwards, **1a** (314.1 mg, 1.5 mmol) was injected to the catalyst mixture and the Schlenk tube was sealed. After heating for 24 h at 80°C, the solvent was removed *in vacuo*. The received crude oil was purified by vacuum distillation, yielding the enamine (**1b**) as a colorless oil (304.7 mg, 1.46 mmol, 97% yield).

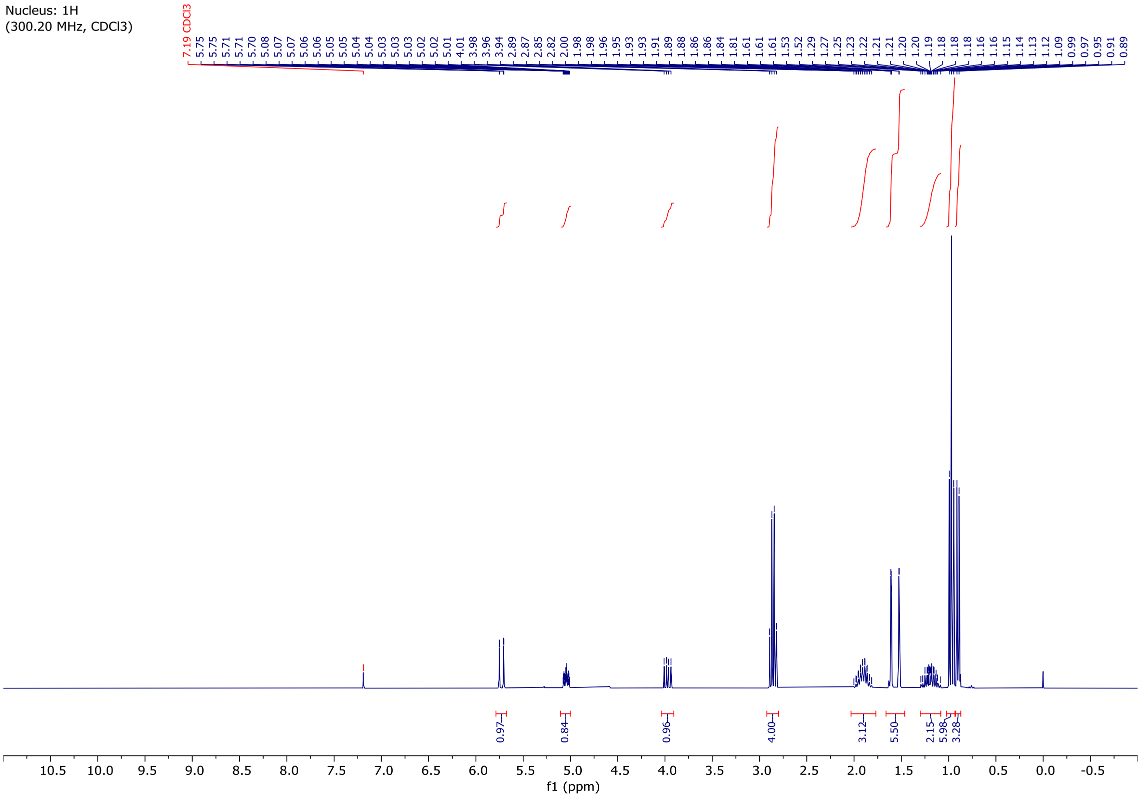

Figure S30: ^{1}H NMR (300 MHz, $CDCl_3$, 293 K) spectrum after distillation - isomerization of **1a** with **Co-2** in THF-d_8.

9.8.4 Isomerization of 1a-D_2 (34a)

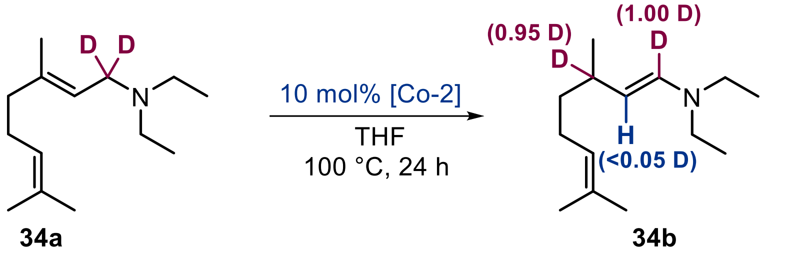

Conv. 98%; 89% yield

First, a 5 mL Schlenk pressure tube was charged with **Co-2** (47.6 mg, 30 µmol) and a stirring bar. Then, 0.8 mL anhydrous THF was added, and the solution/suspension was stirred for 5 min. Next, **1a-*D*₂** (63.4 mg, 0.3 mmol) was injected and the Schlenk pressure tube was sealed. The reaction mixture was heated for 24 h at 100 °C. Afterwards, the solvent was removed *in vacuo* and the received crude oil was purified by vacuum distillation.[10] The deuterated enamine was obtained as colorless oil (56.4 mg, 0.27 mmol, 89% yield).

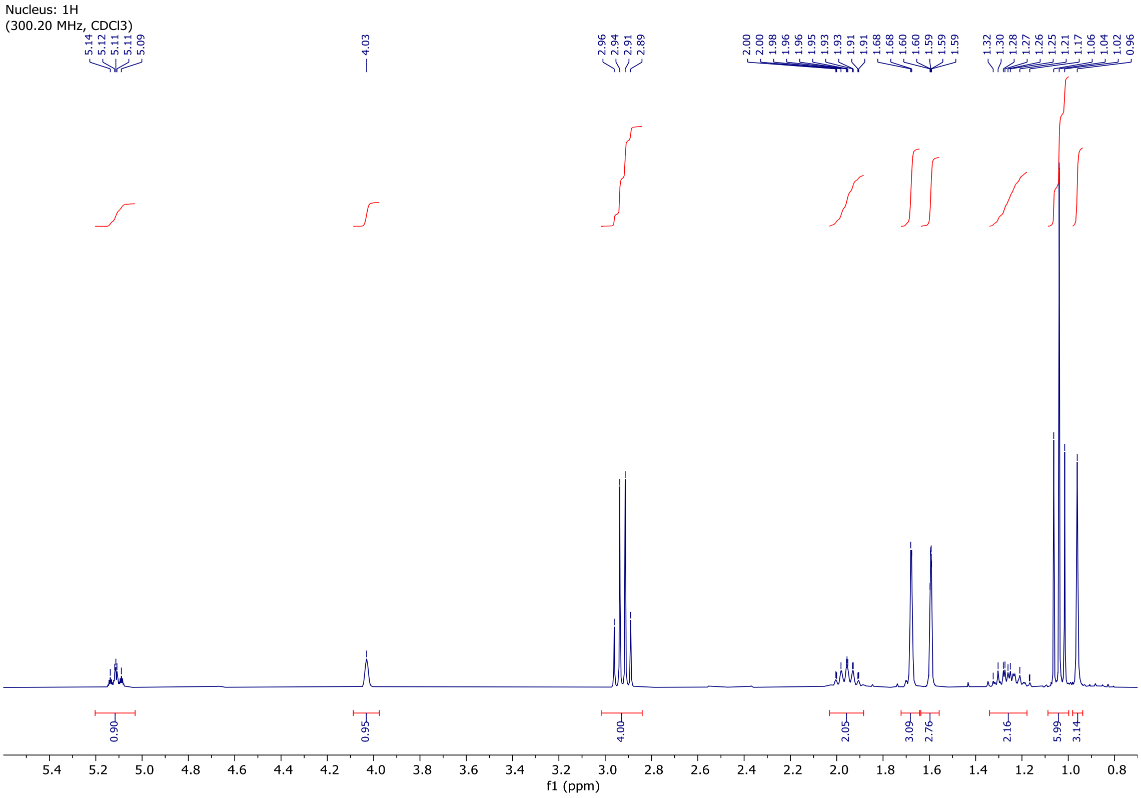

Figure S31: ^{1}H NMR (300 MHz, $CDCl_3$, 293 K) spectrum (0.7 - 5.6 ppm) of **34b**.

[10]Distillation until drying is required.

Comparison of 1a, 1b and 34b:

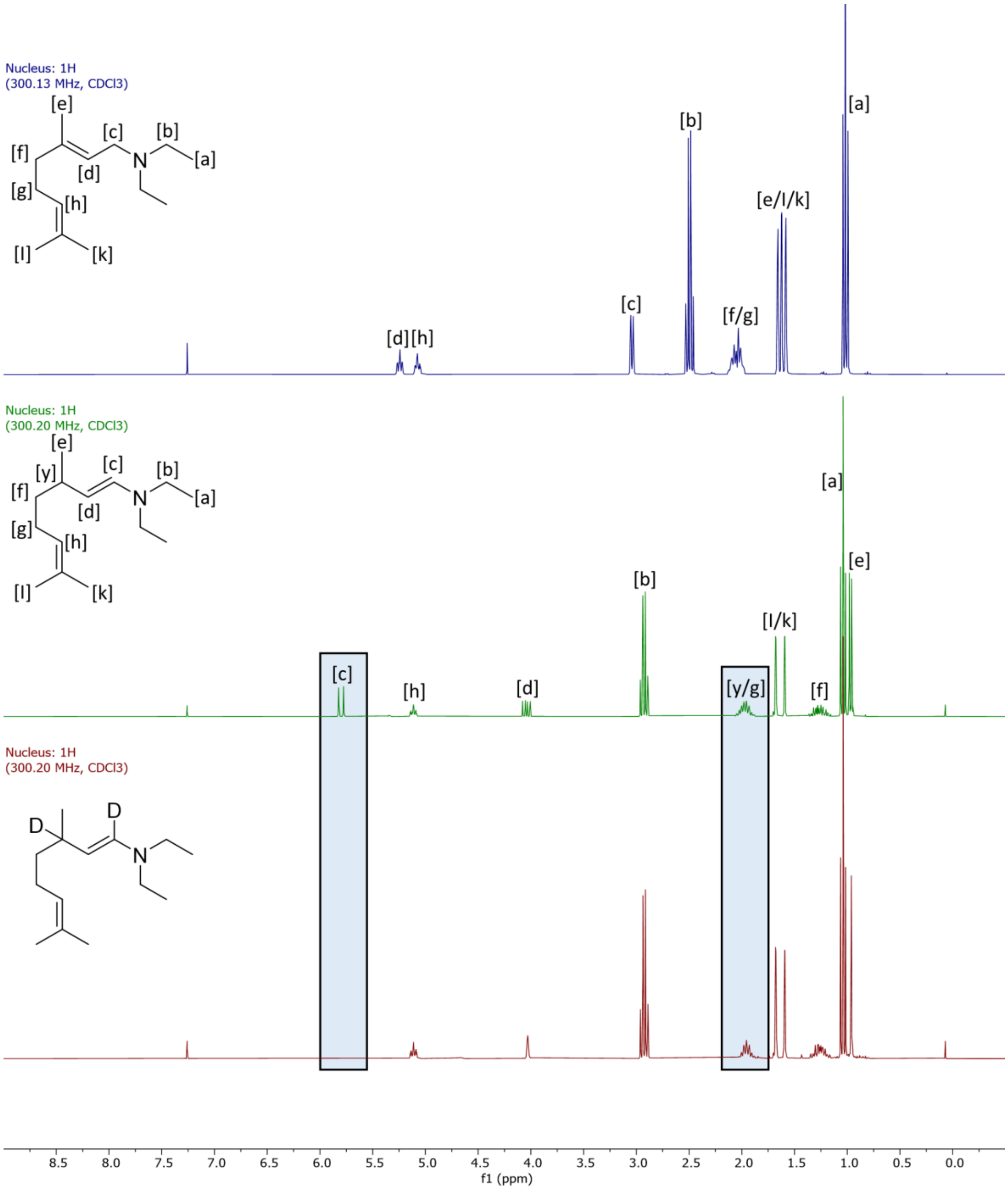

Figure S32: ^{1}H NMR (300 MHz, $CDCl_3$, 293 K) spectra of **1a**, **1b** and **34b**.

^{1}H NMR and ^{13}C NMR spectrum of **34b**

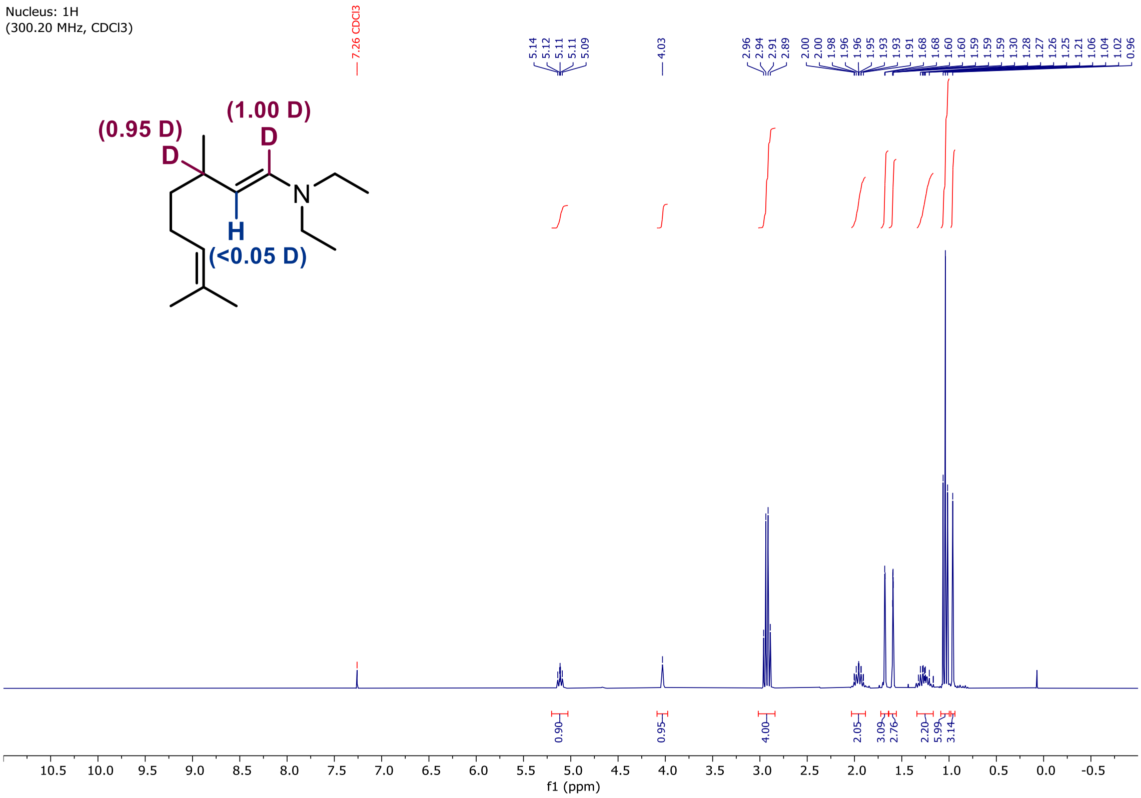

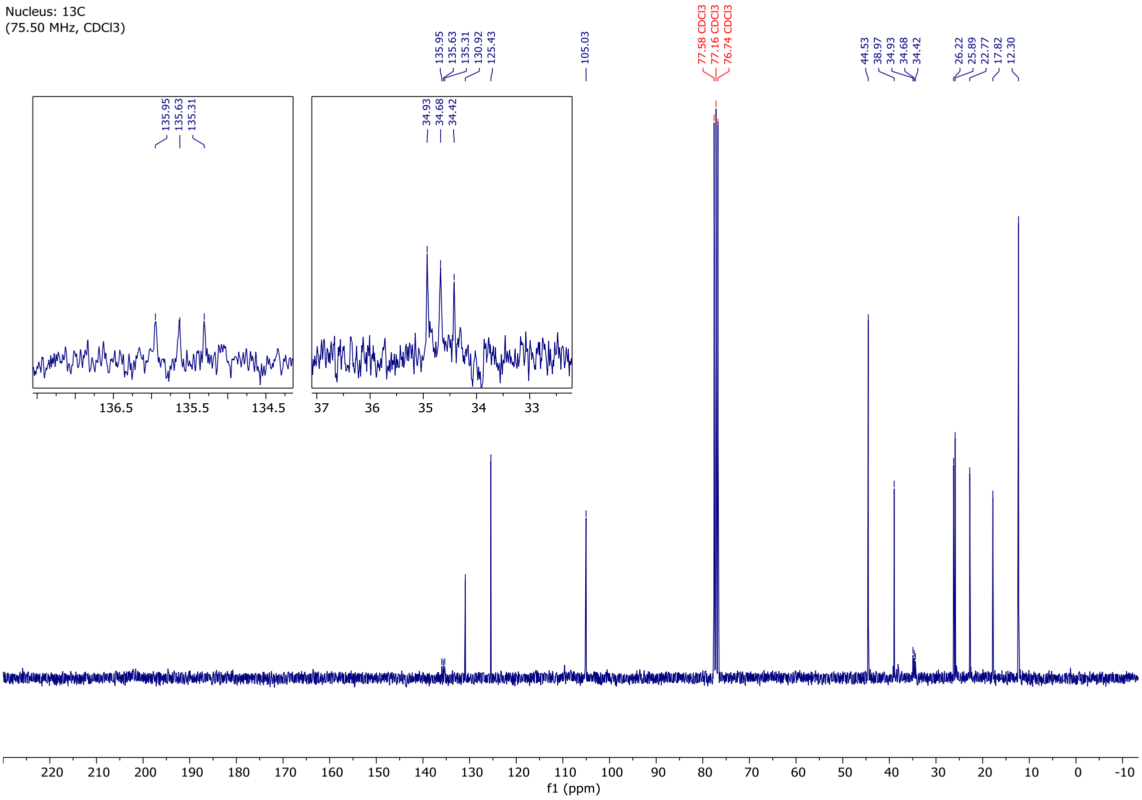

9.8.5 Synthesis of compound 22a-D_2 (35a)

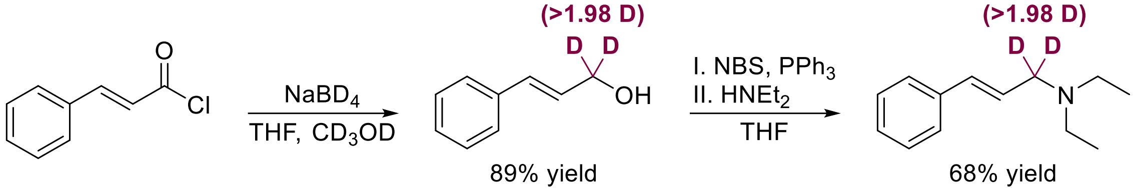

(*E*)-3-phenylprop-2-en-1,1-d$_2$-1-ol[11]

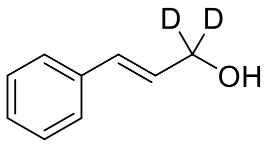

Freshly prepared cinnamoyl chloride (1.33 g, 7.96 mmol) was charged in a 100 mL Schlenk flask with 40 mL anhydrous THF. At room temperature, $NaBD_4$ (1.00 g, 23.89 mmol, 3.0 equiv.) was added in portions to the solution. The reaction mixture was then cooled to 0 °C and 15 mL anhydrous CD_3OD was slowly added over 1 h using a Schlenk dropping funnel. Afterwards, the reaction mixture was warmed up and stirred for 2 h at room temperature. Next, the reaction mixture was quenched with 10 mL D_2O, followed by the addition of 5 mL 1 M HCl and extraction with Et_2O (3x150 mL). The received colorless organic solution was dried with $MgSO_4$ and the solvent was removed *in vacuo*. Finally, the crude oil was purified with flash column chromatography (pentane + 40% Et_2O), yielding a white crystalline solid.[13]

Chemical Formula: $C_9H_8D_2O$

Molecular Weight: 136.1902

Appearance: white solid

Isolated Yield: 89% (961 mg, 7.06 mmol)

^{1}H NMR (300 MHz, CD_2Cl_2): δ = 7.45 - 7.37 (m, 2H), 7.36 - 7.28 (m, 2H), 7.28 - 7.20 (m, 1H), 6.61 (d, *J* = 15.9 Hz, 1H), 6.37 (d, *J* = 15.9 Hz, 1H), 1.54 (s, 1H).

^{13}C NMR (75 MHz, CD_2Cl_2): δ = 167.92, 161.79, 133.06, 124.77, 117.08, 60.56, 34.37, 27.87, 25.88, 25.45, 17.69, 14.64.

[11]The selective synthesis of deuterium-labeled cinnamyl alcohol via the reduction of ethyl cinnamate with lithium aluminum deuteride, as reported by Wiest *et al.*, did not provide the product exclusively. In addition to the target product (3-phenylprop-2-en-1,1-d$_2$-1-ol), we also observed the reduction of the double bond (3-phenylpropane-1,1-d$_2$-1-ol, approx. 10 %).

^{1}H NMR and ^{13}C NMR spectrum of (*E*)-3-phenylprop-2-en-1,1-d_2-1-ol

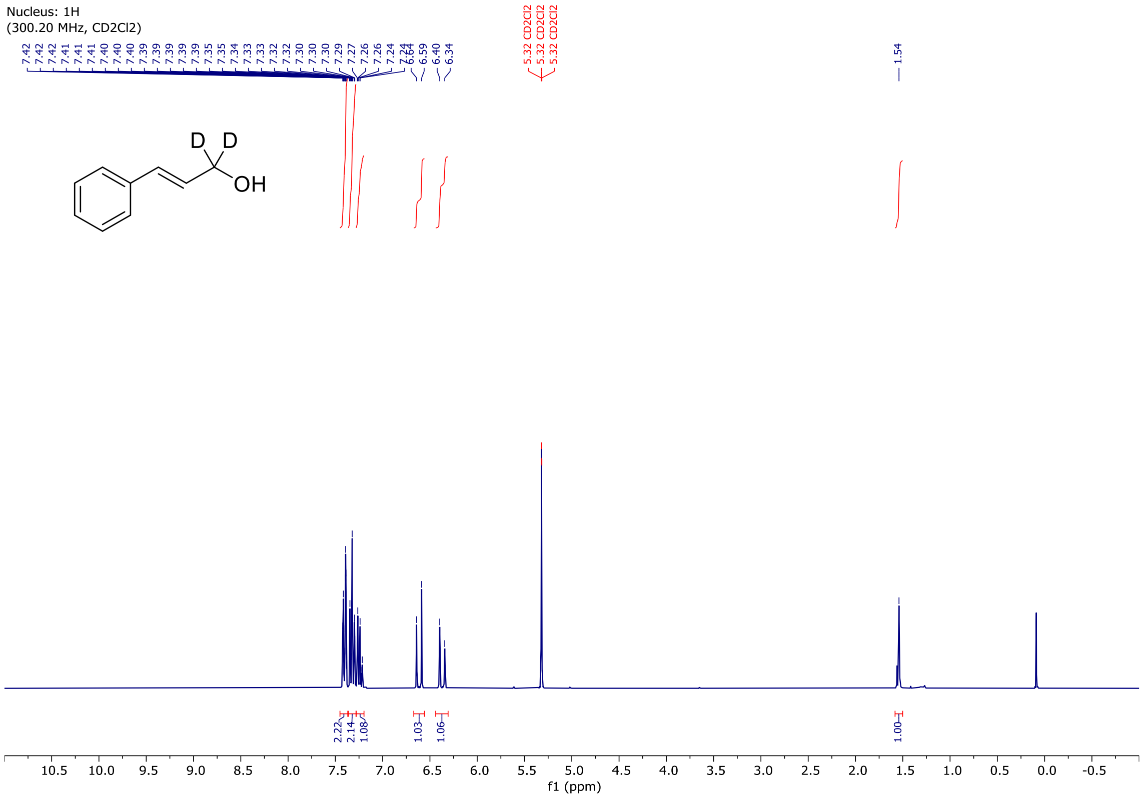

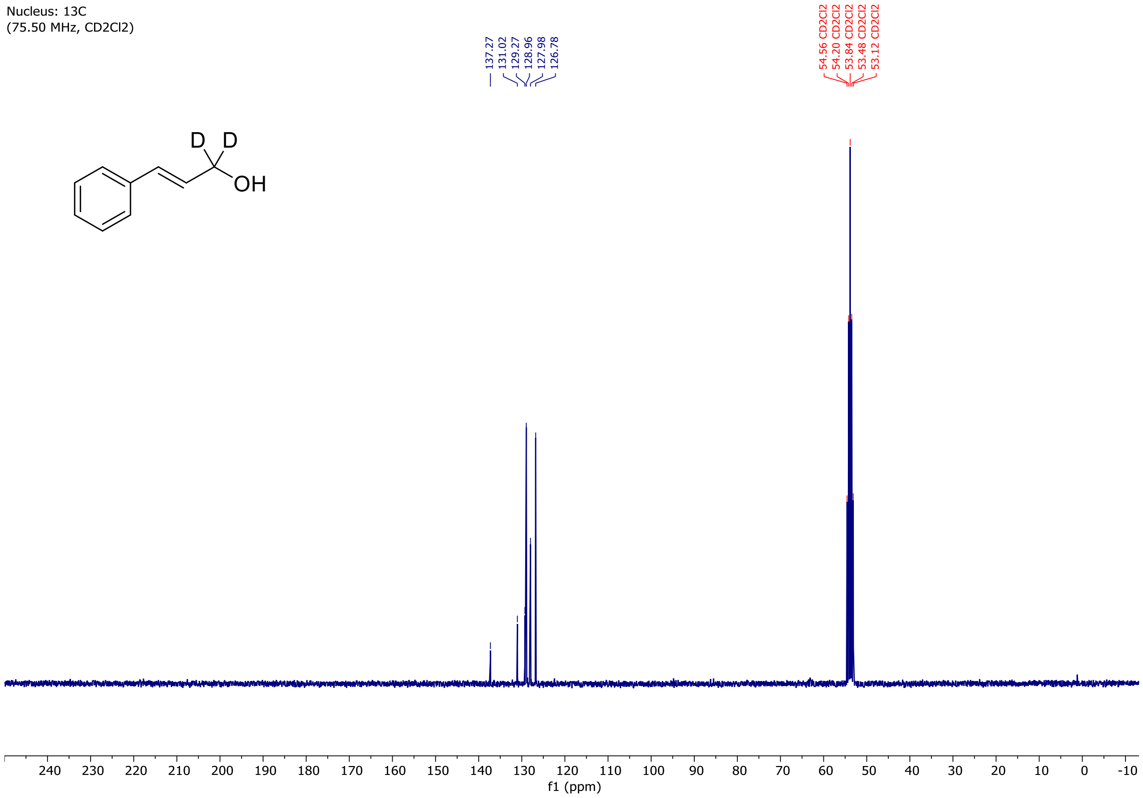

(*E*)-*N,N*-diethyl-3-phenylprop-2-en-1-amine-1,1-d_2

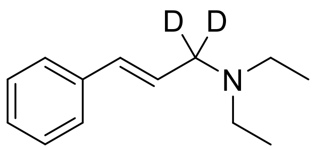

First, a 50 mL round bottom flask was charged with (*E*)-3-phenylprop-2-en-1,1-d_2-1-ol (950 mg, 6.98 mmol, 1.0 equiv.), triphenylphosphine (2.10 g, 8.02 mmol, 1.1 equiv.) and 15 mL THF. Next, *N*-bromosuccinimide (1.43 g, 8.02 mmol, 1.1 equiv.) was added in portions under stirring. After 10 min of stirring, diethylamine (549.4 mg, 7.51 mmol, 2.1 equiv.) was added dropwise to the reaction mixture and the solution was heated to 60 °C for 2 h. Then, 100 mL Et_2O were added, and the resulting suspension was filtrated. The filtrate was extracted with 1M HCl, washed with Et_2O and made alkaline by the addition of 2M NaOH. The aqueous solution was extracted with Et_2O, and the combined organic solutions were dried with sodium sulphate. After filtration, the solvent was removed *in vacuo*. Finally, the target product was obtained by fractional distillation.

Chemical Formula: $C_{13}H_{17}D_2N$

Molecular Weight: 191.3142

Appearance: light yellowish oil

Isolated Yield: 68% (907 mg, 4.74 mmol)

^{1}H NMR (300 MHz, CD_2Cl_2): 7.42 - 7.35 (m, 2H), 7.34 - 7.26 (m, 2H), 7.25 - 7.17 (m, 1H), 6.51 (d, *J* = 15.9 Hz, 1H), 6.27 (d, *J* = 15.9 Hz, 1H), 2.54 (q, *J* = 7.1 Hz, 4H), 1.03 (t, *J* = 7.1 Hz, 6H).

^{13}C NMR (75 MHz, CD_2Cl_2): δ = 137.84, 131.96, 128.88, 128.76, 127.55, 126.55, 47.12, 12.18.

^{1}H NMR and ^{13}C NMR spectrum of (*E*)-*N*,*N*-diethyl-3-phenylprop-2-en-1-amine-1,1-d_2

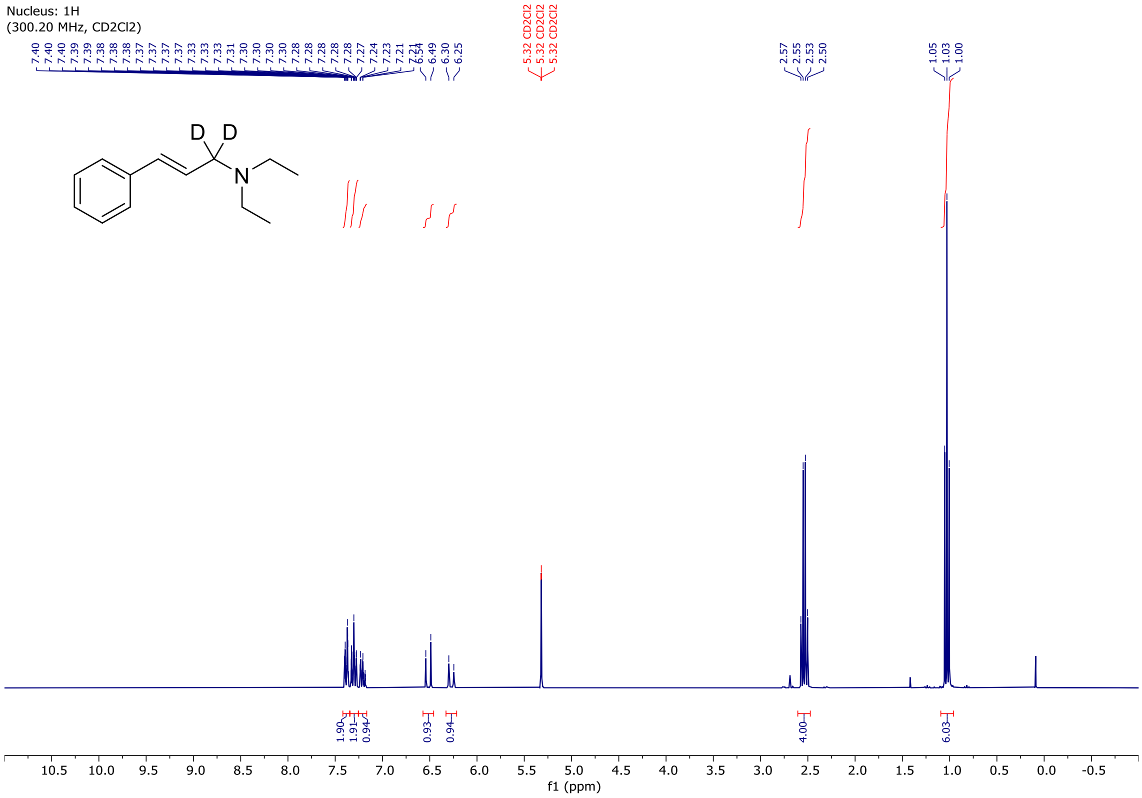

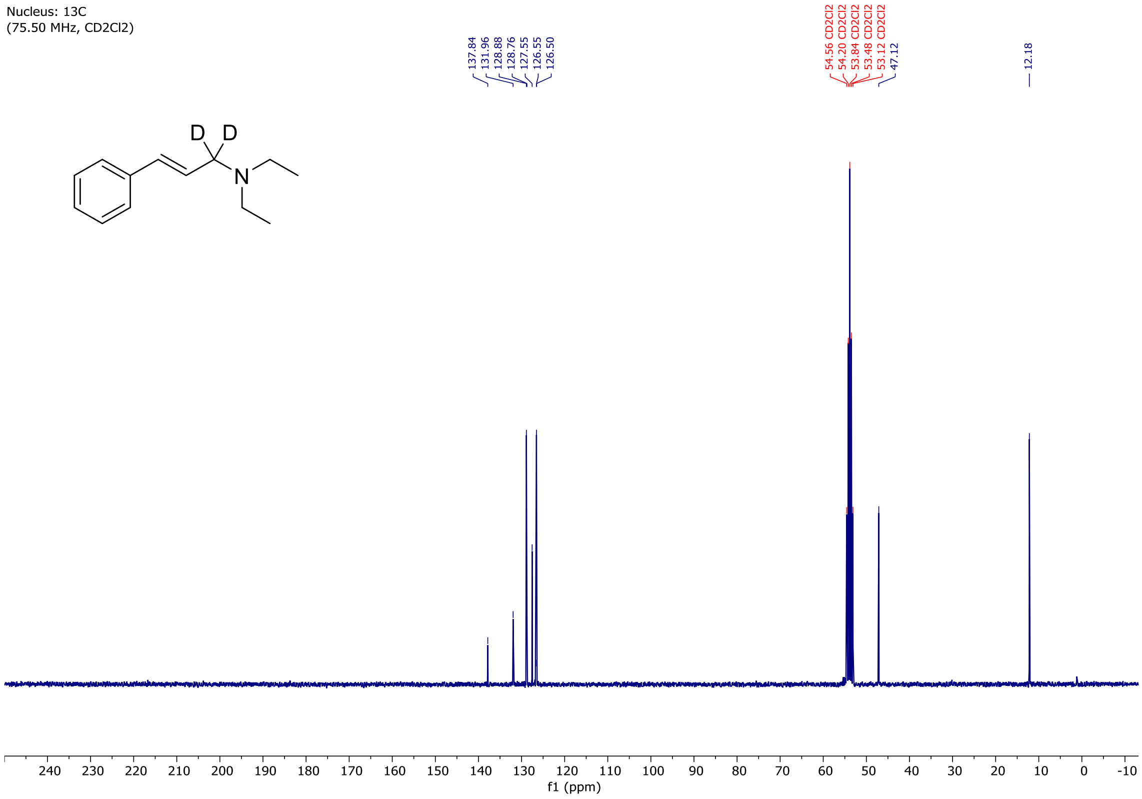

Deuterium incorporation of 35a (22a-D_2):

- Duplet of -CH_2-NR_2 (**22a**): 3.22 ppm
- Deuterium incorporation in γ-position of **22a-*D*₂**: >1.98 D

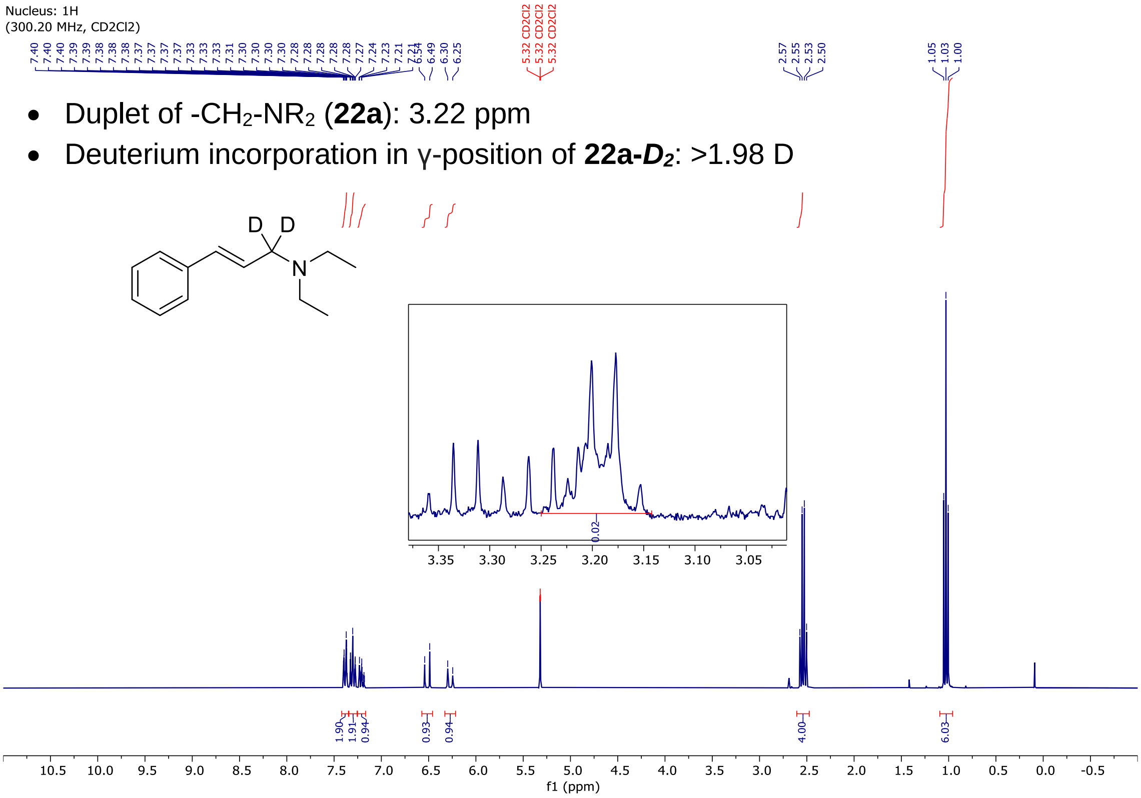

Comparison: ^{1}H NMR spectra of 22a and 35a (22a-D_2).

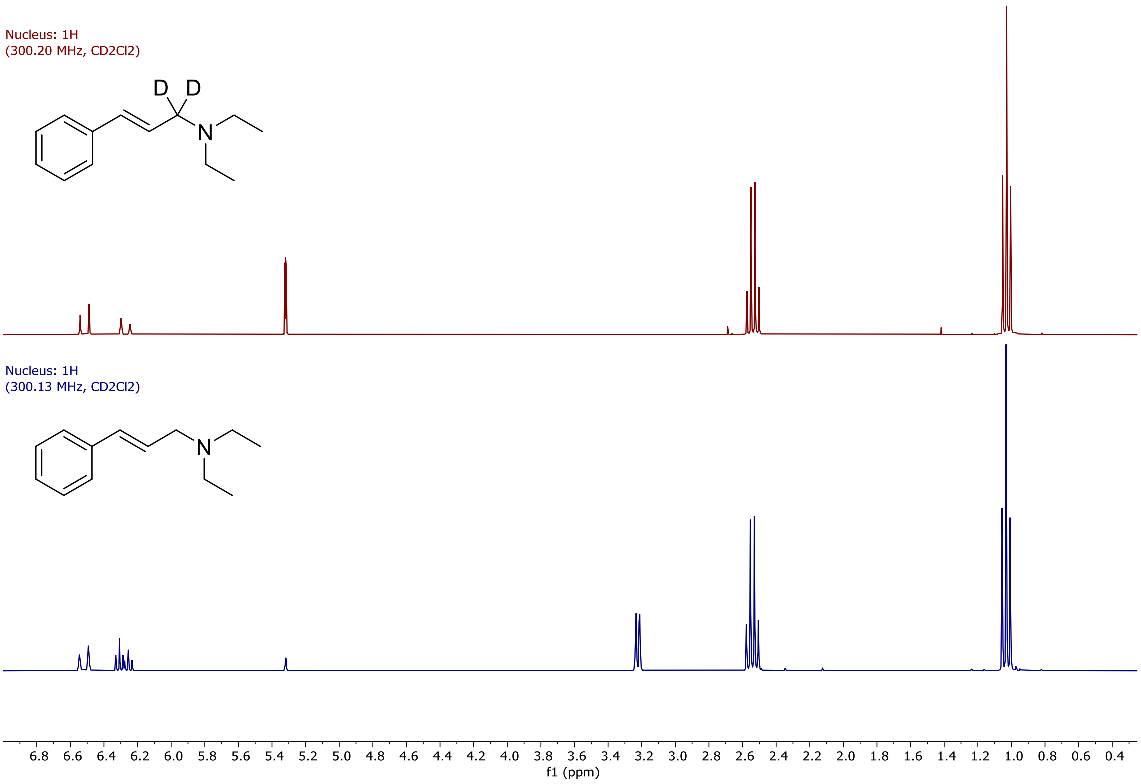

9.8.6 Isomerization of 22a-D_2 (35a)

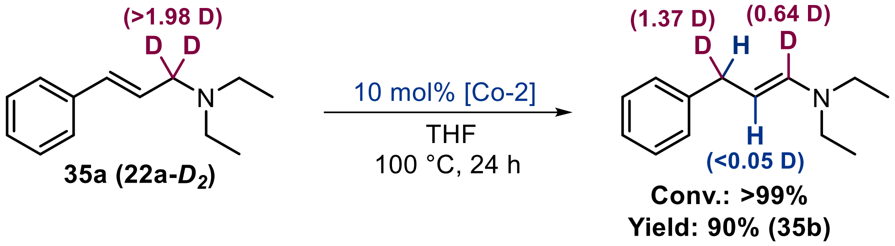

Under argon atmosphere, **Co-2** (47.6 mg, 30 µmol) was weighed into a 5 mL Schlenk pressure tube. Then, 0.8 mL anhydrous THF was added, and the solution/suspension was stirred for approx. 5 min. Afterwards, **22a-*D*₂** (57.4 mg, 0.3 mmol) was injected and the Schlenk pressure tube was sealed, and the reaction mixture was heated for 24 h at 100 °C. Afterwards, the solvent was removed *in vacuo* and the received crude oil was purified by vacuum distillation.[12] The deuterated enamine was obtained as colorless oil (51.7 mg, 0.27 mmol, 90% yield).

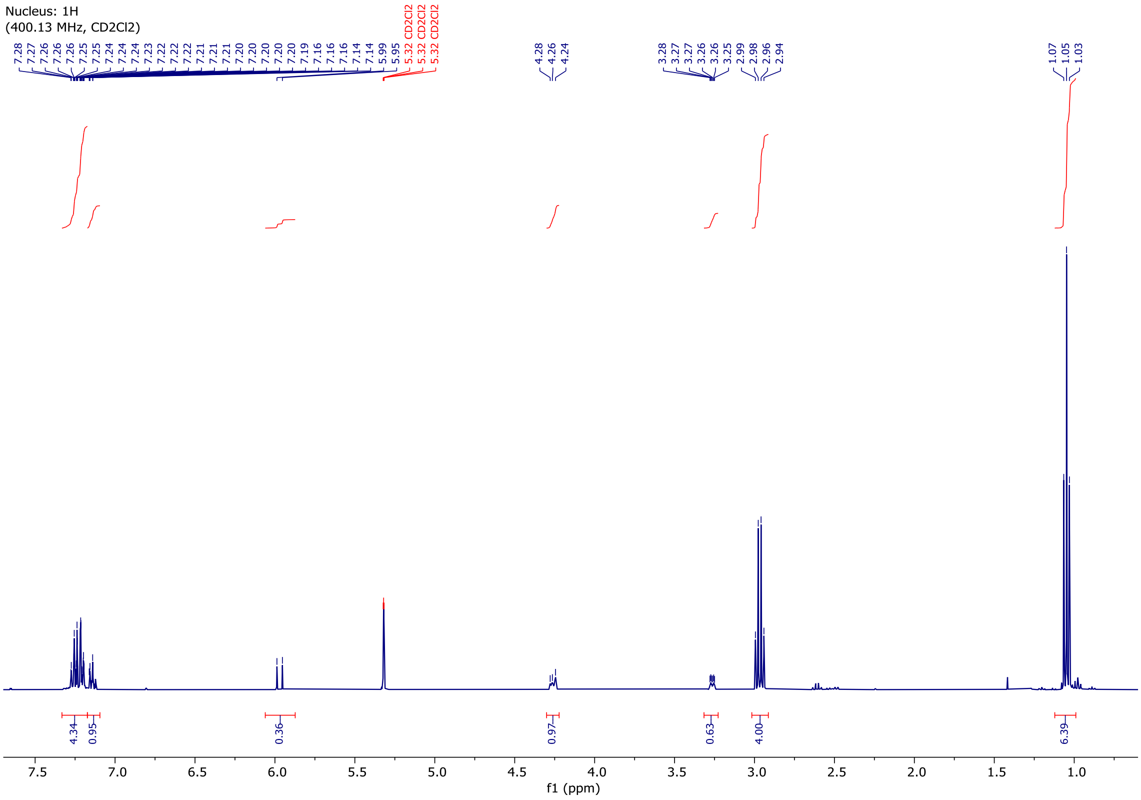

Figure S33: ^{1}H NMR (300 MHz, $CDCl_3$, 293 K) spectrum (0.6 – 7.7 ppm) of **35b.**

[12]Distillation until drying is required.

Comparison of 22a, 22b and 35b:

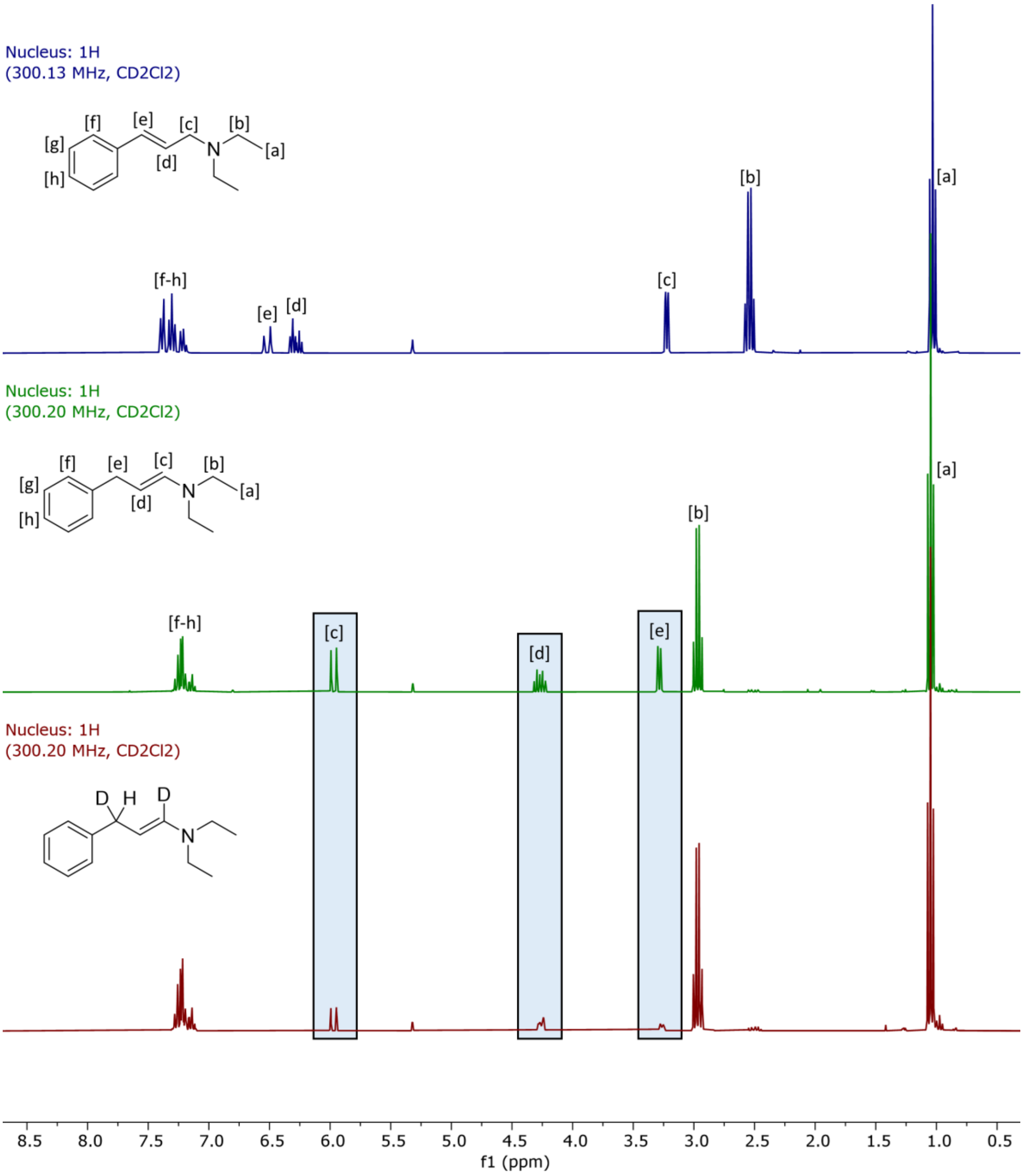

Figure S34: ^{1}H NMR (300 MHz, $CDCl_3$, 293 K) spectra of **22a**, **22b** and **35b**.

^{1}H NMR and ^{13}C NMR spectrum of **35b** (**48 h reaction time**):

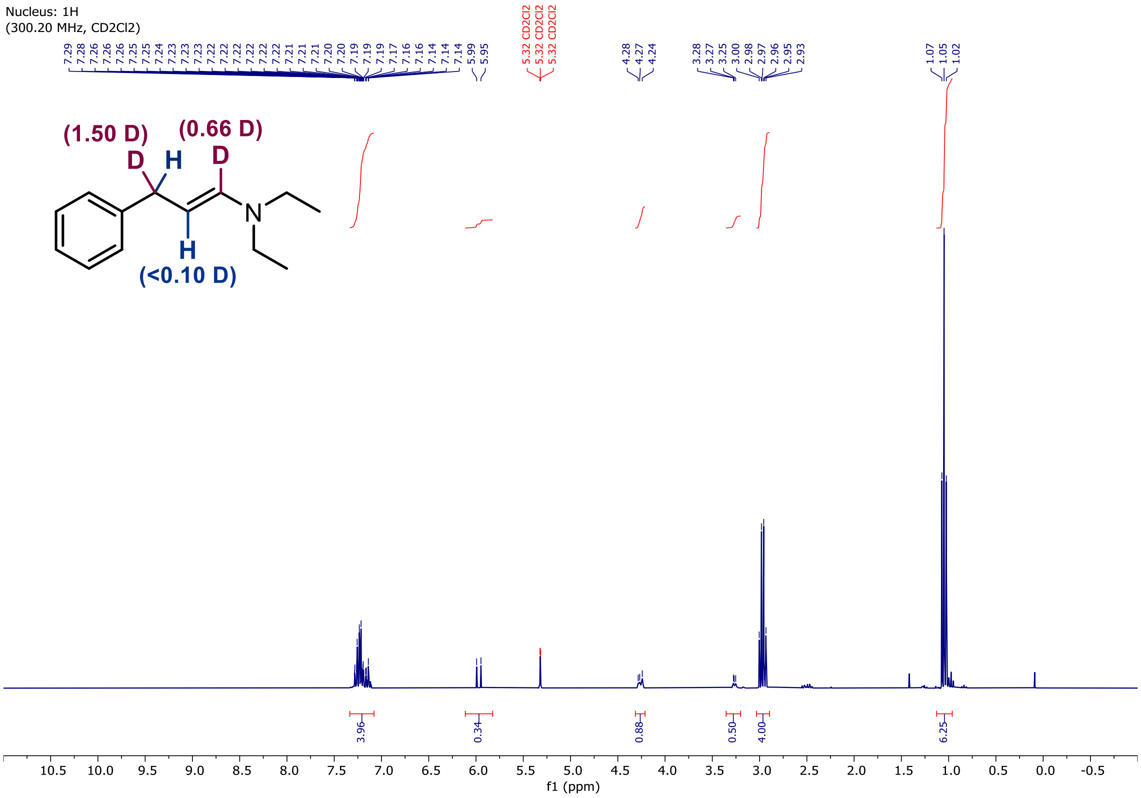

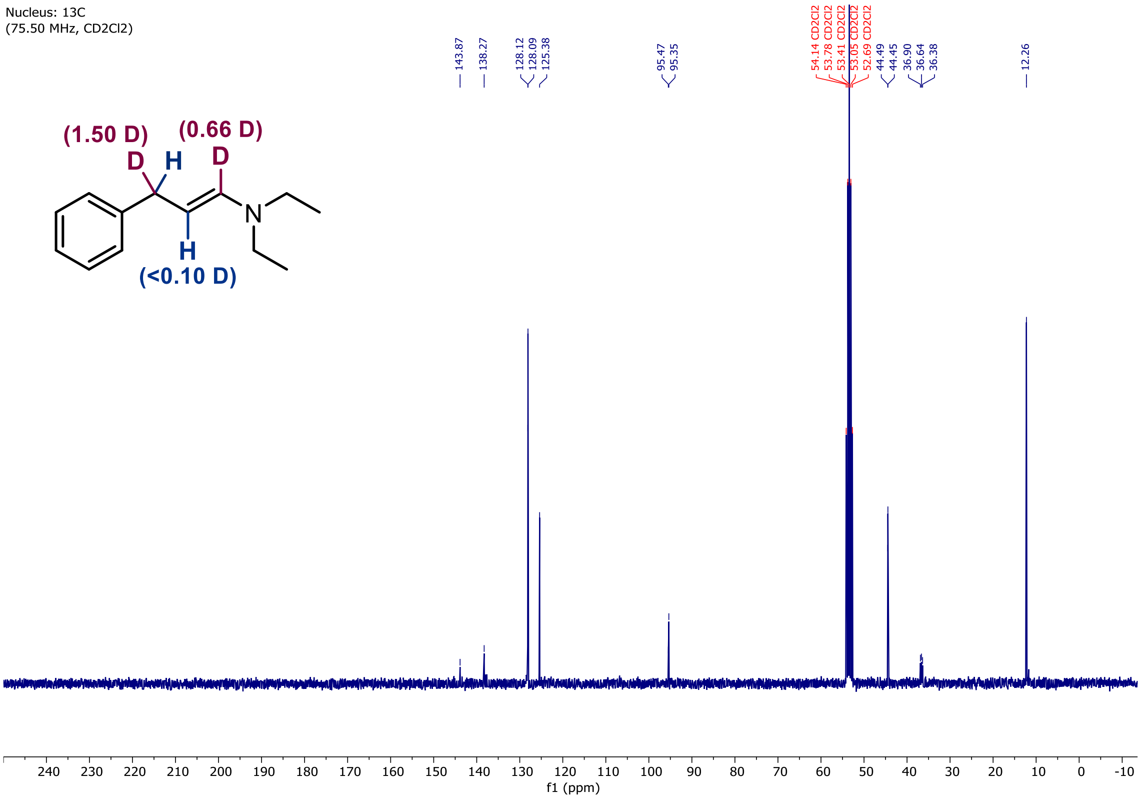

9.8.7 Crossover-Experiment

I. Aromatic Allylamines: 35a (22a-*D*₂) and 25a

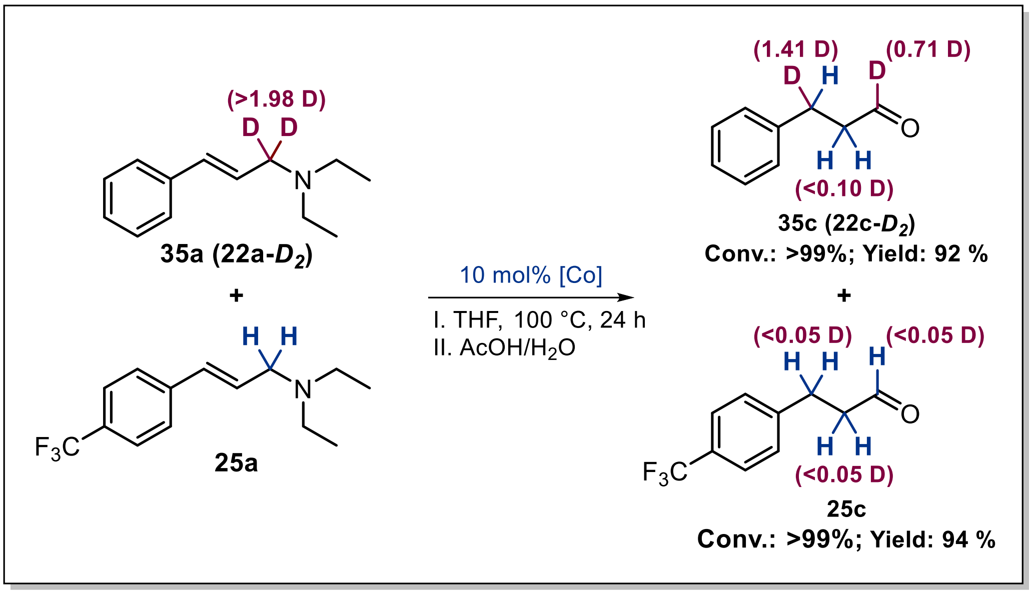

In an argon-filled glovebox, **Co-2** (47.6 mg, 30 µmol) was weighed into a 5 mL Schlenk pressure tube equipped with a stirring bar, followed by the addition of 1.2 mL of anhydrous THF. The mixture was stirred for 5 min. Subsequently, (**35a**) **22a-*D*$_2$** (28.7 mg, 0.15 mmol) and **25a** (38.6 mg, 0.15 mmol) were injected under argon atmosphere, and the Schlenk pressure tube was sealed. The reaction mixture was then heated to 100 °C for 24 h, afterwards the solvent was removed *in vacuo* and a vacuum distillation was carried out (until drying is required). The obtained clear oil (enamine mixture) was hydrolyzed with 3 mL of a 5% acetic acid solution. After 30 min of stirring, the product mixture was extracted with diethyl ether, followed by two washing steps with distilled water. Then, the organic solution was dried with sodium sulfate and the solvent was removed under reduced pressure. Finally, both products were purified and separated by flash column chromatography using a gradient of diethyl ether and *n*-pentane.

Comparison of 25c and 35c:

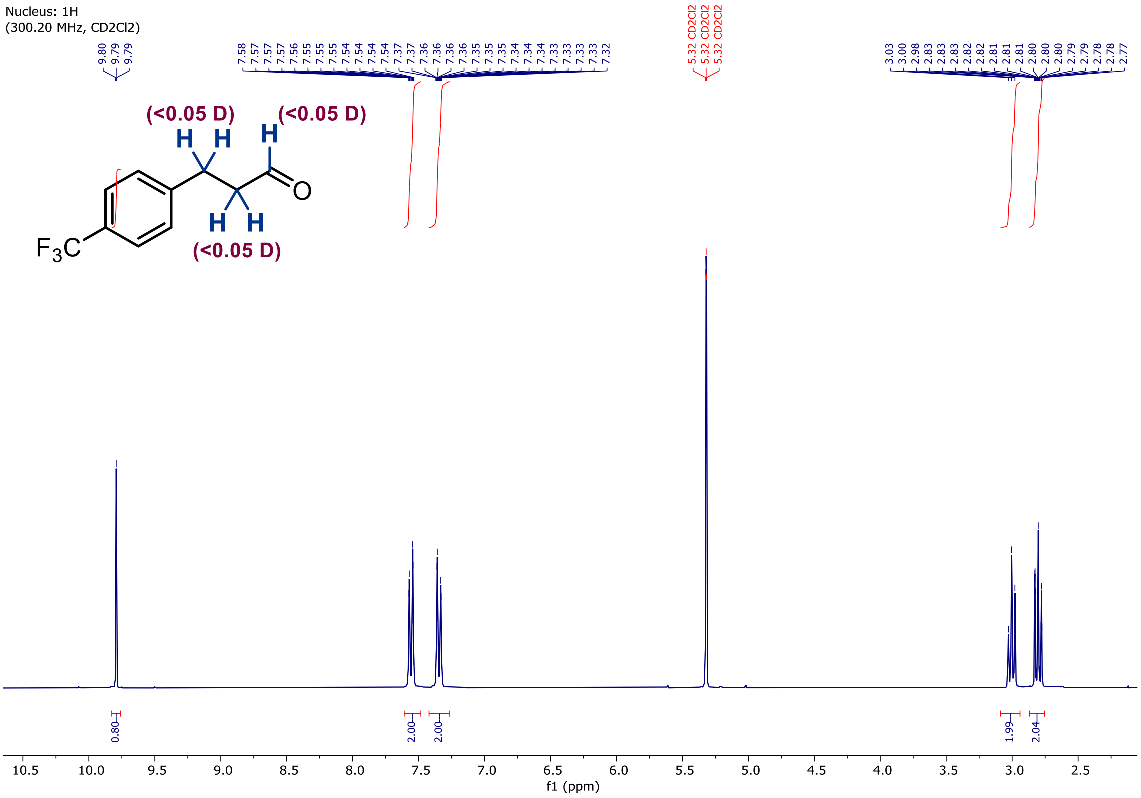

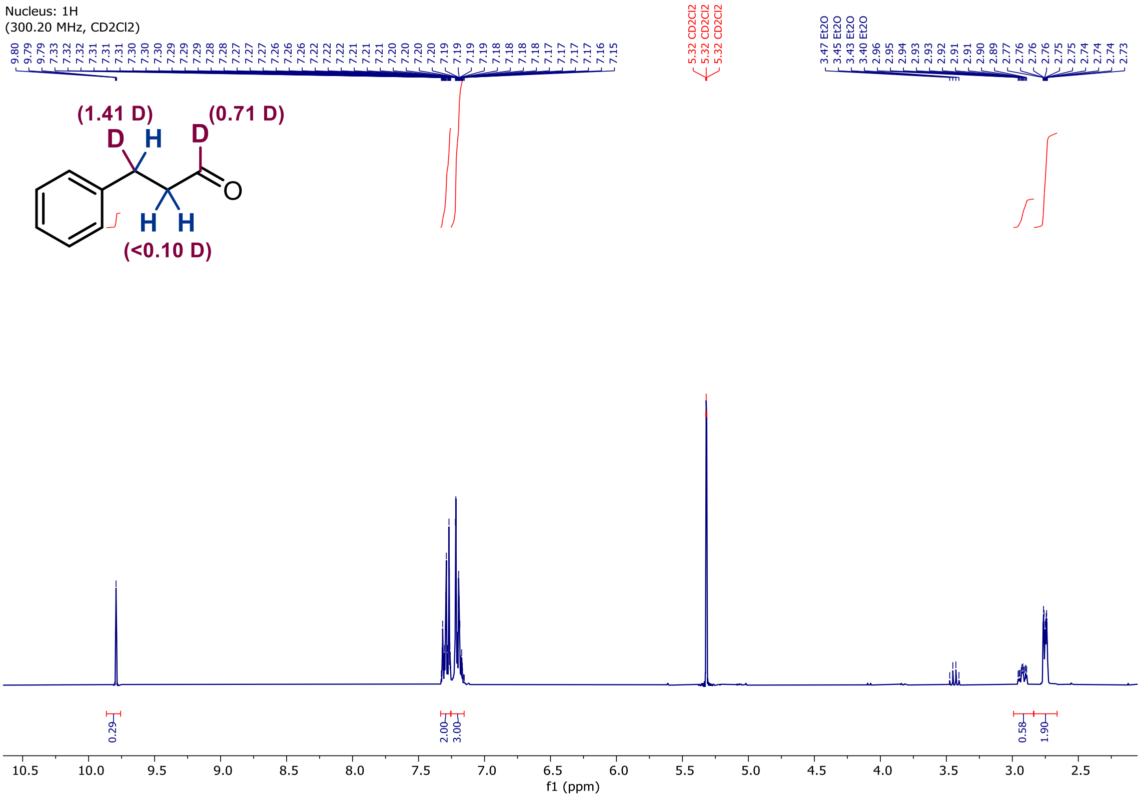

^{1}H NMR and ^{13}C NMR spectrum of **25c**

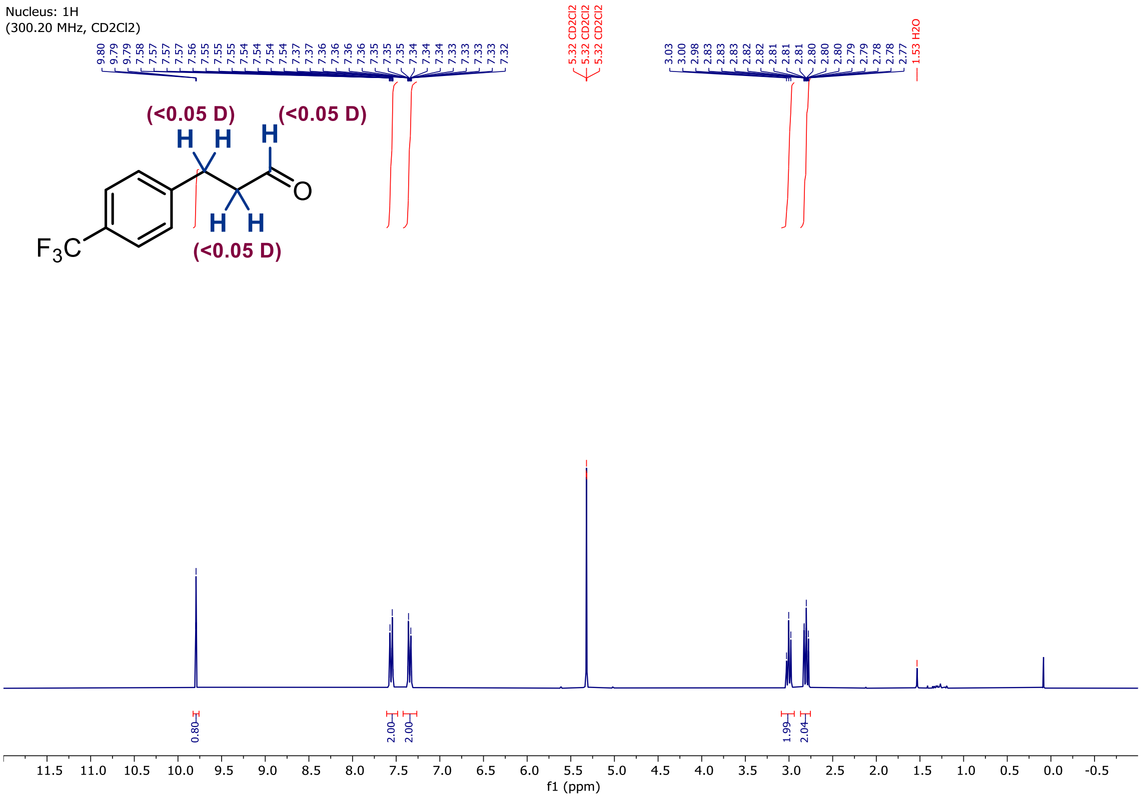

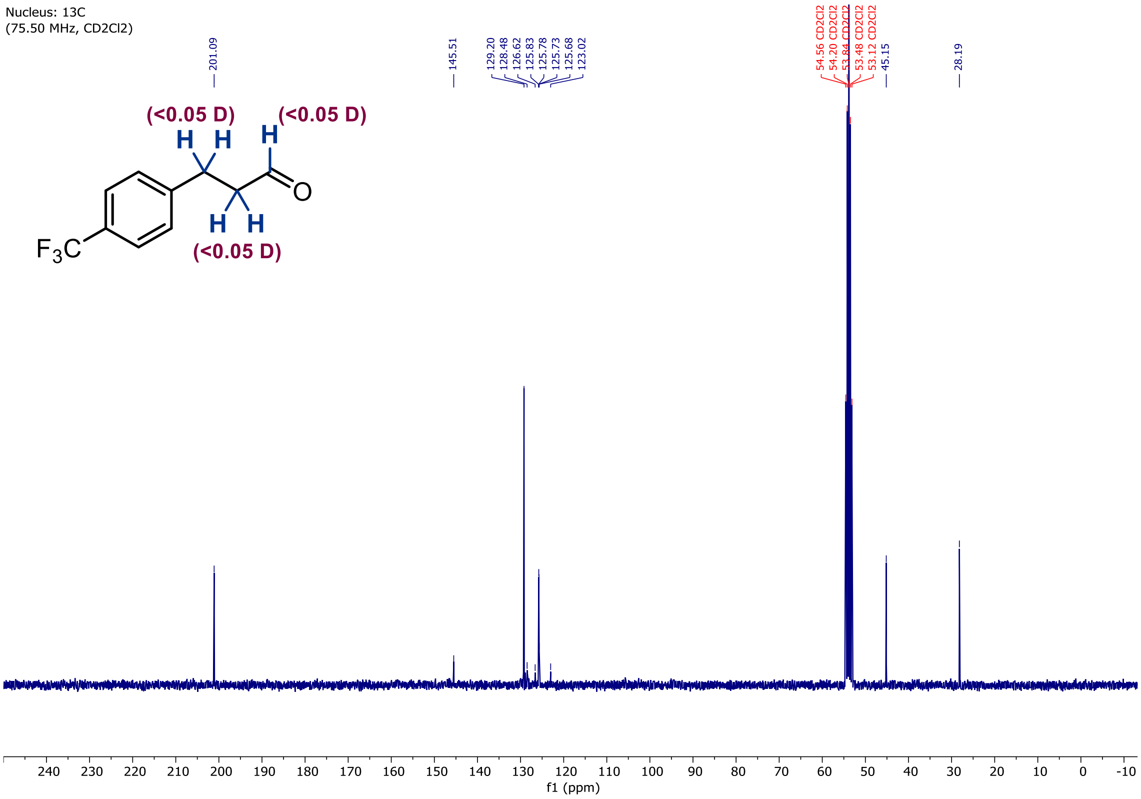

^{1}H NMR and ^{13}C NMR spectrum of **35c (22c-*D*$_2$)**

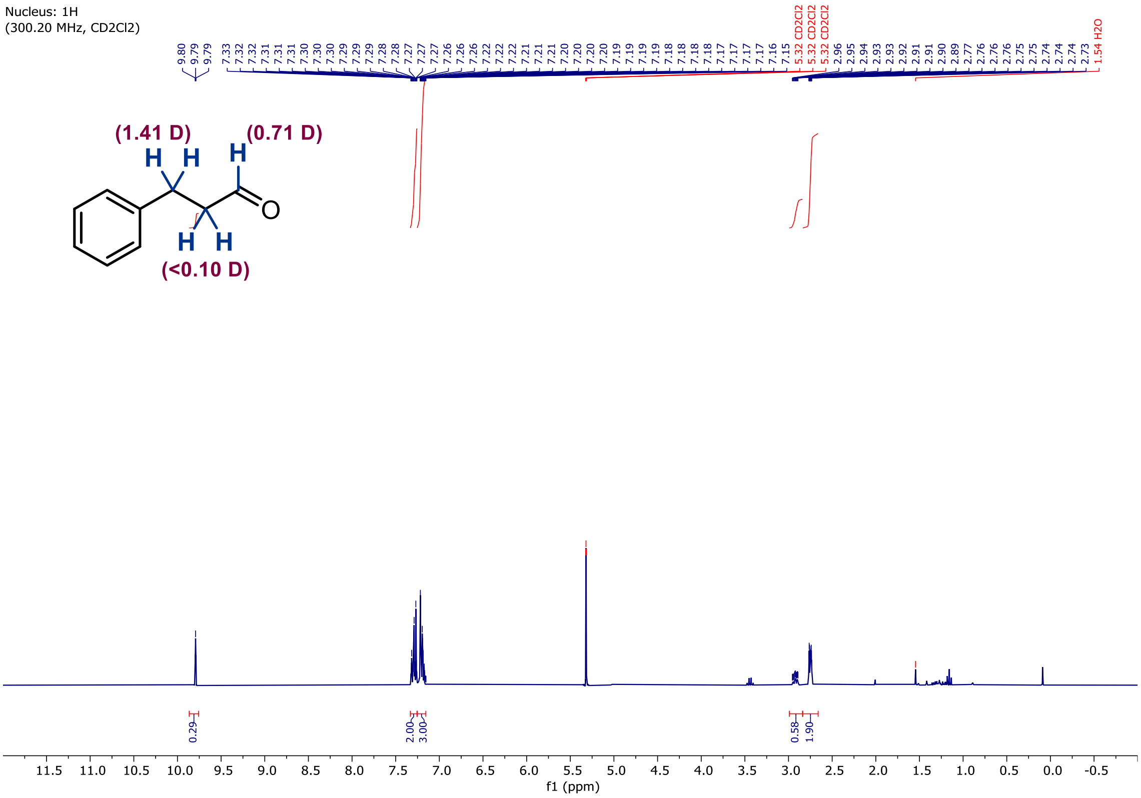

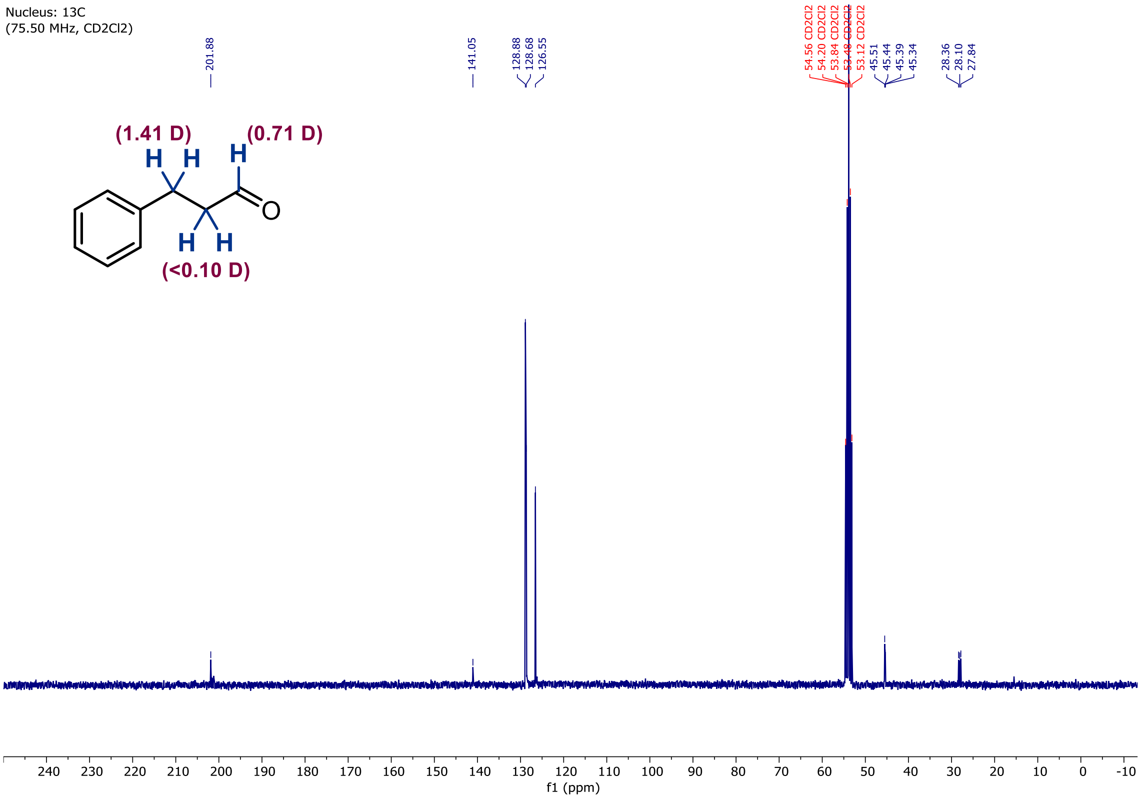

II. Aromatic Allylamines: 34a (22a-D_2) and 25a

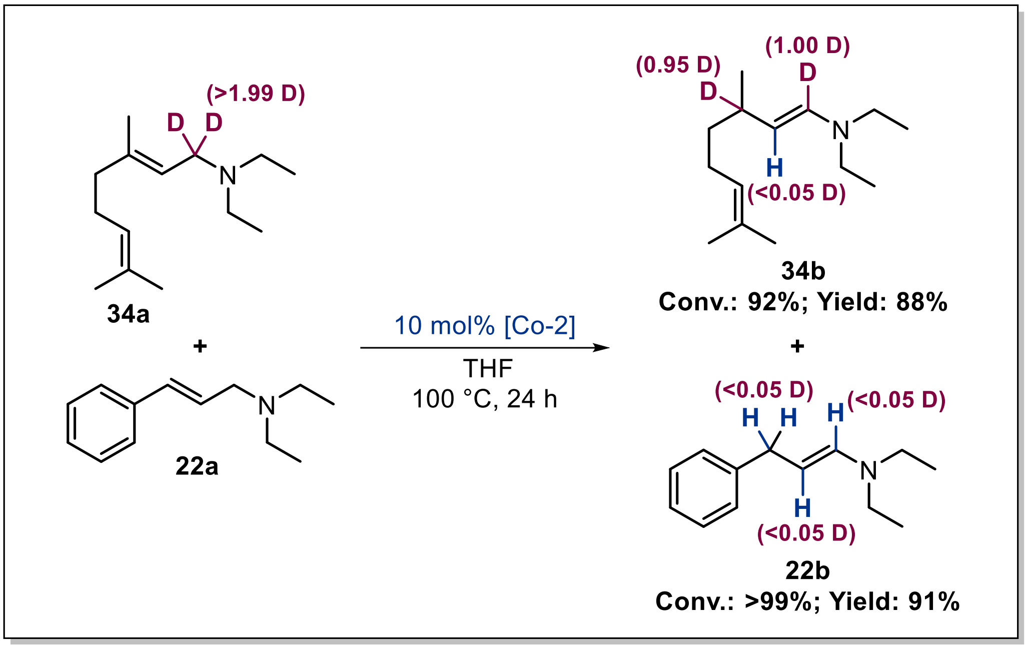

Under argon atmosphere, a 5 mL Schlenk pressure tube was charged with **Co-2** (47.6 mg, 30 µmol) and a stirring bar. Then, 0.8 mL anhydrous THF was added, and the solution was stirred for 5 min. Next, **34a** (**1a-D_2**) (31.7 mg, 0.15 mmol) and **22a** (28.4 mg, 0.15 mmol) were injected and the Schlenk pressure tube was sealed. The reaction mixture was heated for 24 h at 100 °C. Afterwards, the solvent was removed *in vacuo* and the received crude oil was purified by vacuum distillation (until drying is necessary!). To avoid hydrolysis of the enamines, the NMR was performed under argon using anhydrous deuterated solvents.

*Important note: Yields were calculated from the distilled mixture; the products were not separated! After distillation a colorless oil (56.2 mg) was received, containing both enamines **34b** and **22b**. According to the ^{1}H NMR the ratio of **34b** to **22b** was 51:49, whereby **22a** was completely converted and **34a** was 92% converted. Based on these data the yield was calculated:*

$$n_{combined} = 0.3\text{ mmol} \cdot \frac{56.2\ mg}{60.1\ mg} = 0.2805\text{ mmol}$$

$$n_{34b} = n_{combined} \cdot ratio_{34b/22b} \cdot conversion_{34a} = 0.2805\text{ mmol} \cdot 0.512 \cdot 0.92 = 0.132\text{ mmol}$$

$$Yield_{34b} = \frac{0.132\ mmol}{0.15\ mmol} \cdot 100 = \underline{88\%}$$

$n_{22b} = n_{combined} \cdot ratio_{22b/34b} \cdot conversion_{22a} = 0.2805\ mmol \cdot 0.488 \cdot 1 = 0.137\ mmol$

$$Yield_{20ab} = \frac{0.137\ mmol}{0.15\ mmol} \cdot 100 = \underline{91\%}$$

^{1}H NMR - Crossover Experiment:

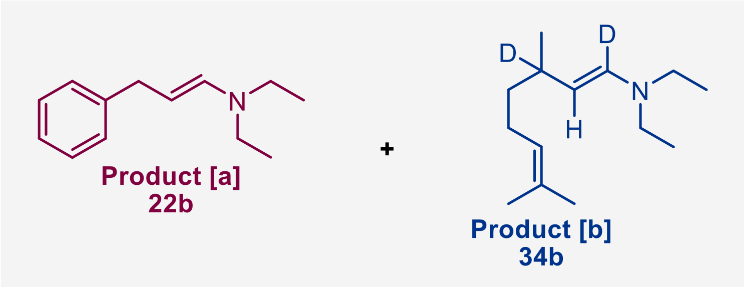

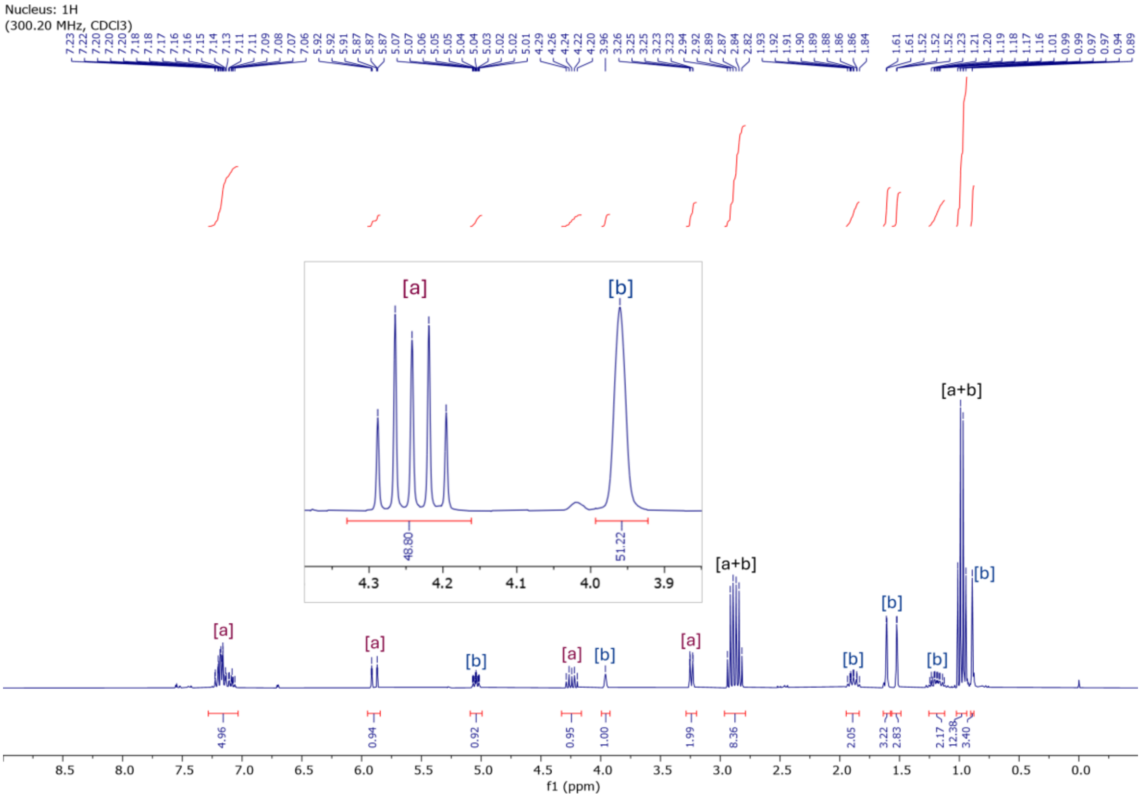

Figure S35: ^{1}H NMR (300 MHz, $CDCl_3$, 293 K) spectra - crossover experiment.

Comparison of 34a, 34b and crossover experiment - ^{1}H NMR:

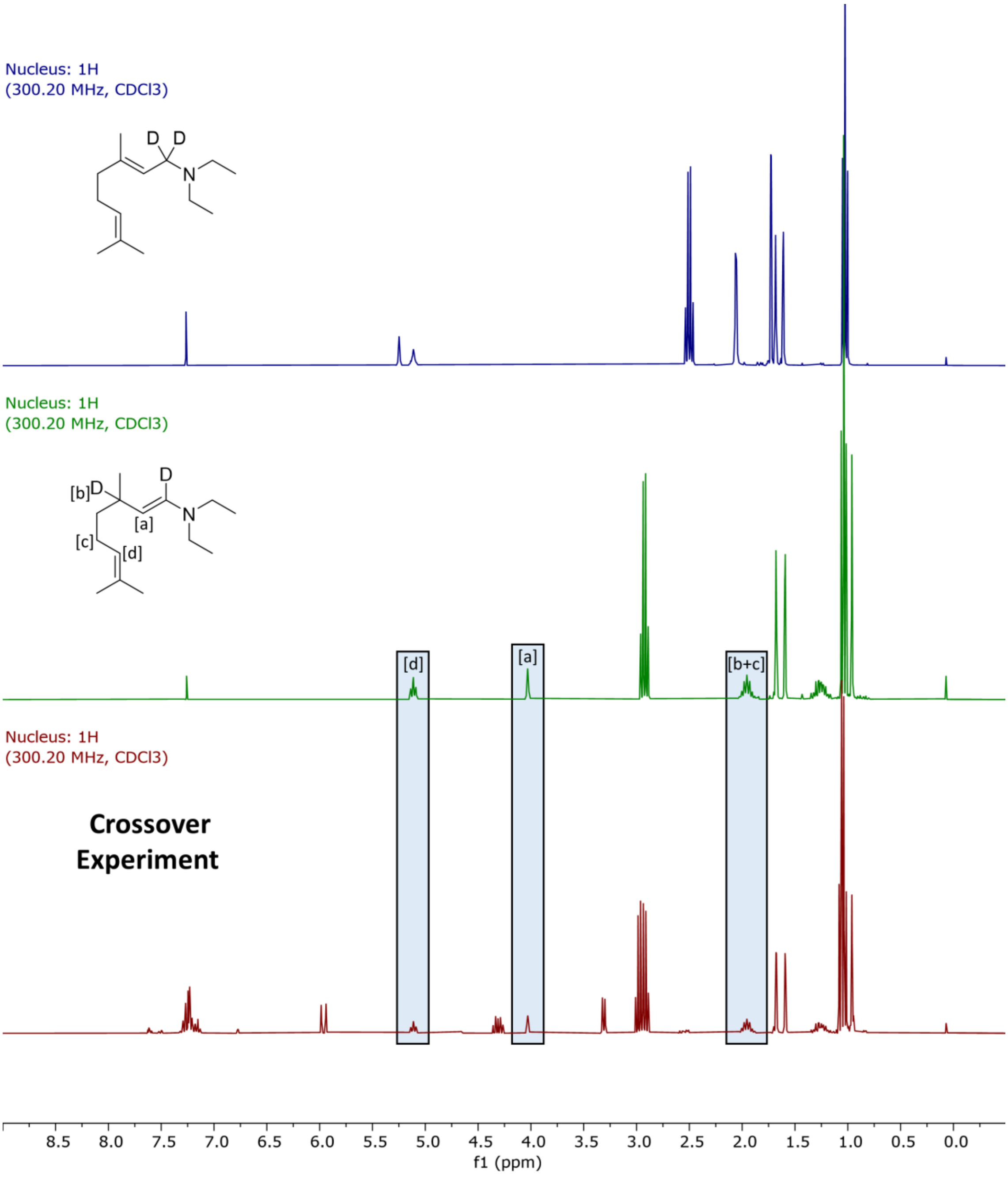

Figure S36: ^{1}H NMR (300 MHz, $CDCl_3$, 293 K) spectra of **34a**, **34b** and **crossover experiment**.

Comparison of 22a, 22b and crossover experiment - ^{1}H NMR:

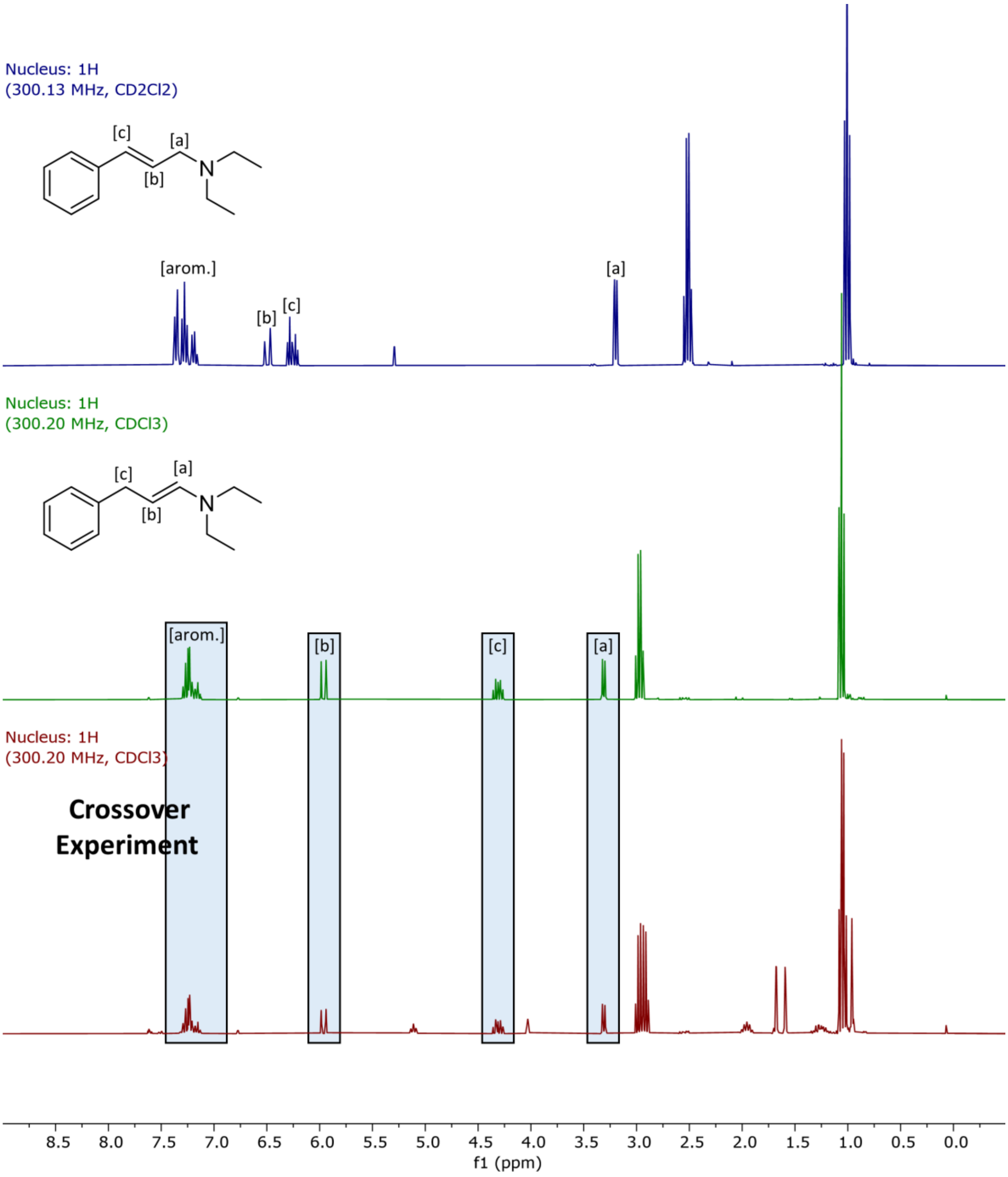

Figure S37: ^{1}H NMR (300 MHz, $CDCl_3$, 293 K) spectra of **22a**, **22b** and **crossover experiment**.

9.9 Isomerization of 3,3-dimethylbutene

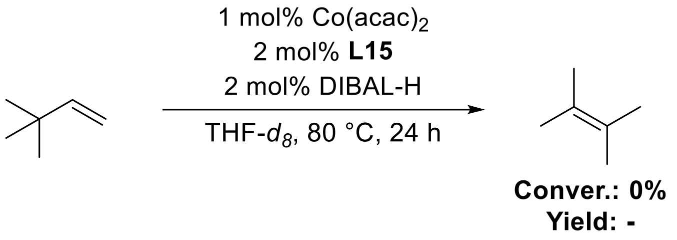

In an argon-filled glovebox, a 5 mL Schlenk pressure tube was charged with $Co(acac)_2$ (2.6 mg, 10 µmol), **L15** (7.6 mg, 20 µmol), and a stirring bar. Next, 1 mL of anhydrous THF-d_8 was injected, followed by the addition of DIBAL-H (20 µmol). Anhydrous 3,3-dimethylbutene (84.2 mg, 1 mmol) was then added, and the Schlenk tube was heated for 24 h. After the reaction time was completed, the mixture was cooled to room temperature, and the THF-d_8 solution was transferred to a J-Young tube.

<u>^{1}H NMR of the crude mixture:</u>

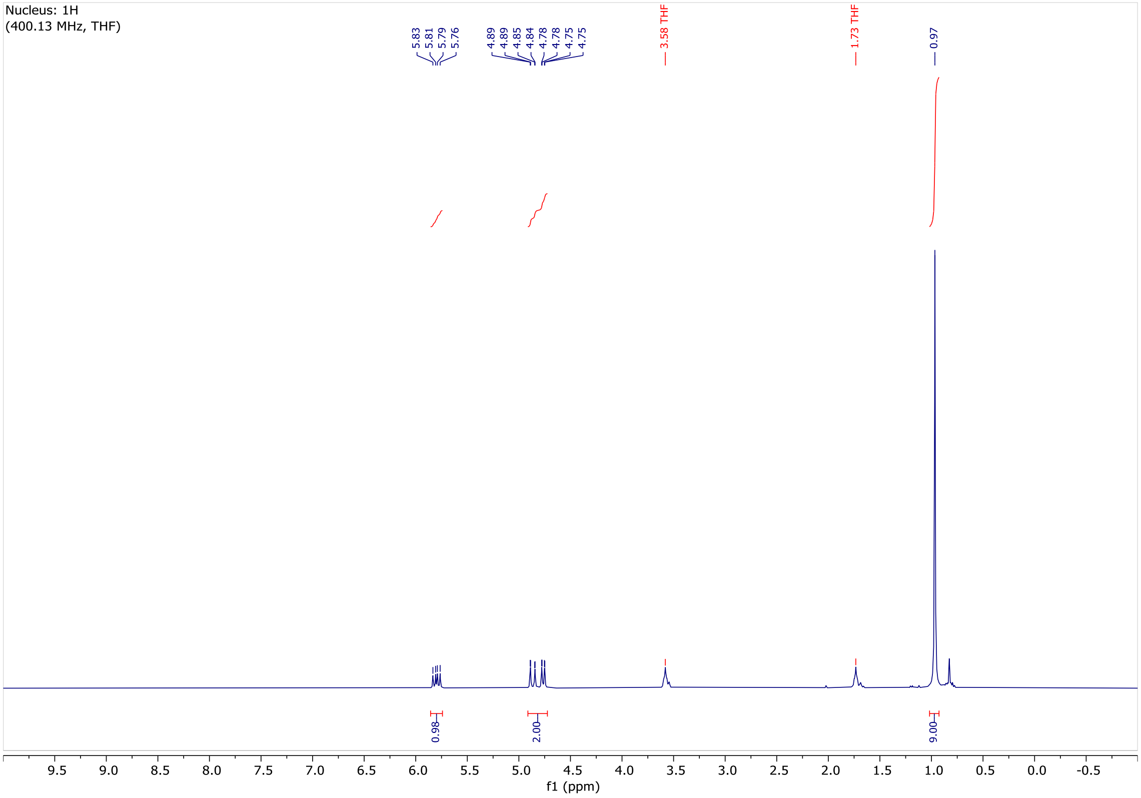

Figure S38: ^{1}H NMR (400 MHz, THF-d_8, 293 K) spectrum of the reaction mixture.

9.10 Kinetic Studies

General procedure:

In an argon-filled glovebox, the catalyst stock solution - comprising $Co(acac)_2$, DIBAL-H, and ligand in a 1:2:2 ratio - was freshly prepared to ensure the integrity of the Co catalyst. A defined volume of this stock solution was then transferred into a 25 mL Schlenk tube, followed by the addition of substrate **1a** and 100 µL of hexadecane, which served as the internal standard (ISDT). Subsequently, the Schlenk tube was sealed with a septum. Finally, the sealed Schlenk tube was removed from the glovebox and placed into a pre-heated oil bath to initiate the reaction.

To monitor the reaction progress, approx. 50 µL of the reaction mixture was withdrawn at each time point and analyzed by GC-chromatography. The yield was calculated based on the combined amounts of the enamine-product **1b** and the hydrolyzed aldehyde **1c**, as determined by GC-chromatography.

Kinetic profile of L1

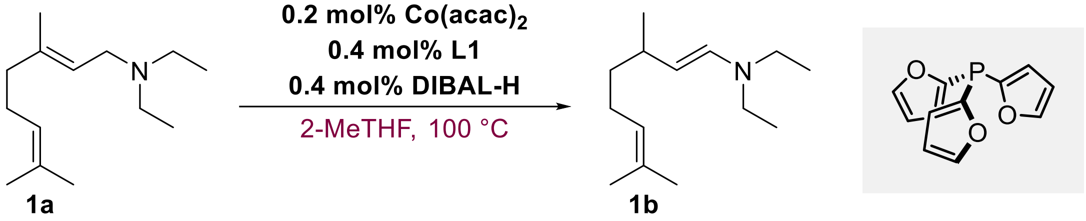

The reaction of the Co/DIBAL-H/**L1** catalytic system was performed according to the general procedure with the following modification:

- Total volume of solvent: 2 ml anhydrous 2-MeTHF
- Catalyst: 5 µmol [Co]
- Substrate (**1a**): 643 µL (2.5 mmol)
- 100 µL ISTD (hexadecane)
- Reaction temperature: 100 °C

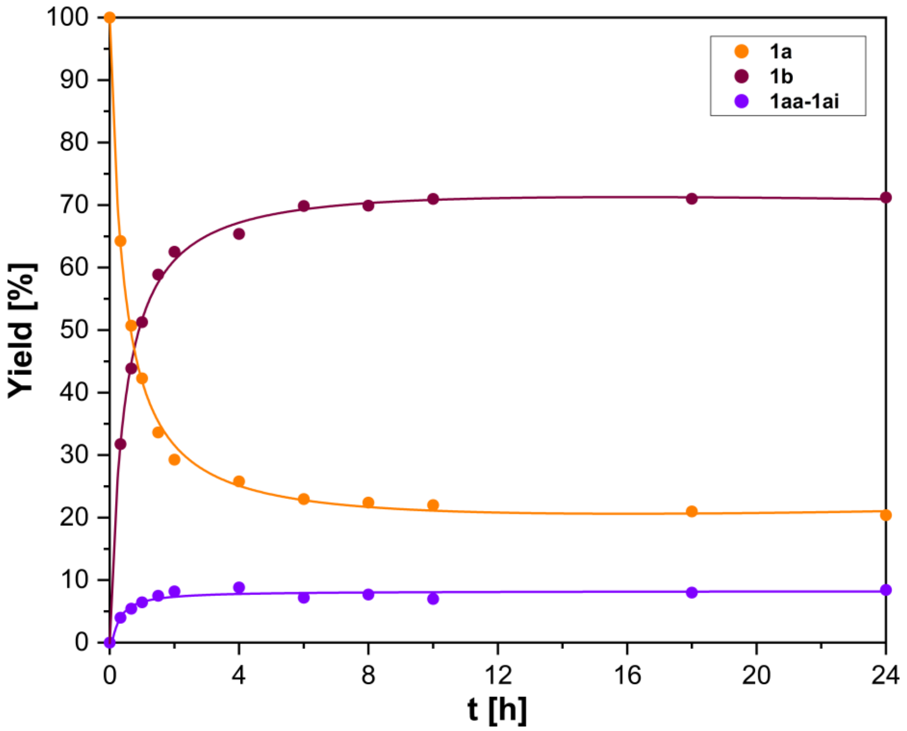

Figure S39: Time course of the formation of product **1b** and byproducts **1ba-1bi** using the Co/DIBAL-H/**L1** catalytic system.

Kinetic profile of L15

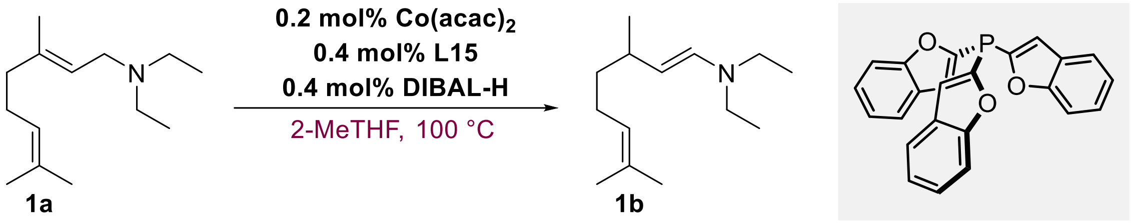

The reaction of the Co/DIBAL-H/**L15** catalytic system was performed according to the general procedure with the following modification:

- Total volume of solvent: 2 ml anhydrous 2-MeTHF
- Catalyst: 5 µmol [Co]
- Substrate (**1a**): 643 µL (2.5 mmol)
- 100 µL ISTD (hexadecane)
- Reaction temperature: 100 °C

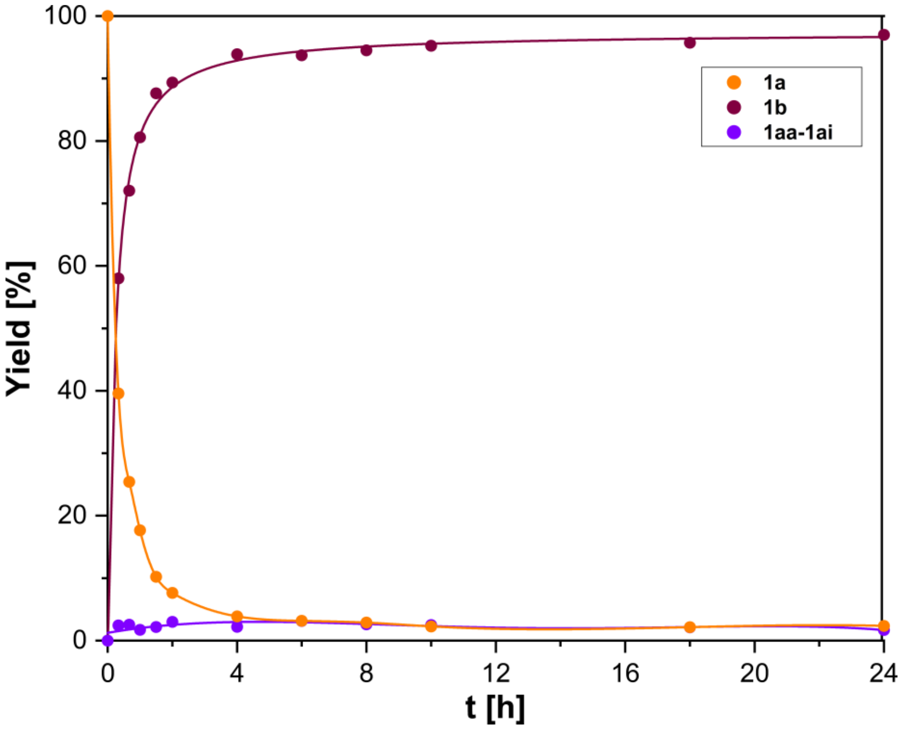

Figure S40: Time course of the formation of product **1b** and byproducts **1ba-1bi** using the Co/DIBAL-H/**L15** catalytic system.

Variation of the reaction temperature

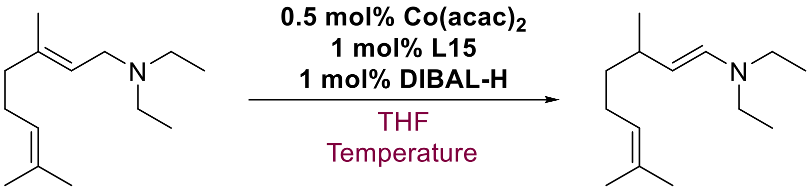

1a **1b**

The reaction of the Co/DIBAL-H/**L15** catalytic system was performed according to the general procedure with the following modification:

- Total volume of solvent: 2.4 ml anhydrous THF
- Catalyst: 12.5 µmol [Co]
- Substrate (**1a**): 643 µL (2.5 mmol)
- 120 µL ISTD (hexadecane)

In total, three identical reaction samples were prepared and heated to 25°C, 40°C, and 60°C, respectively.

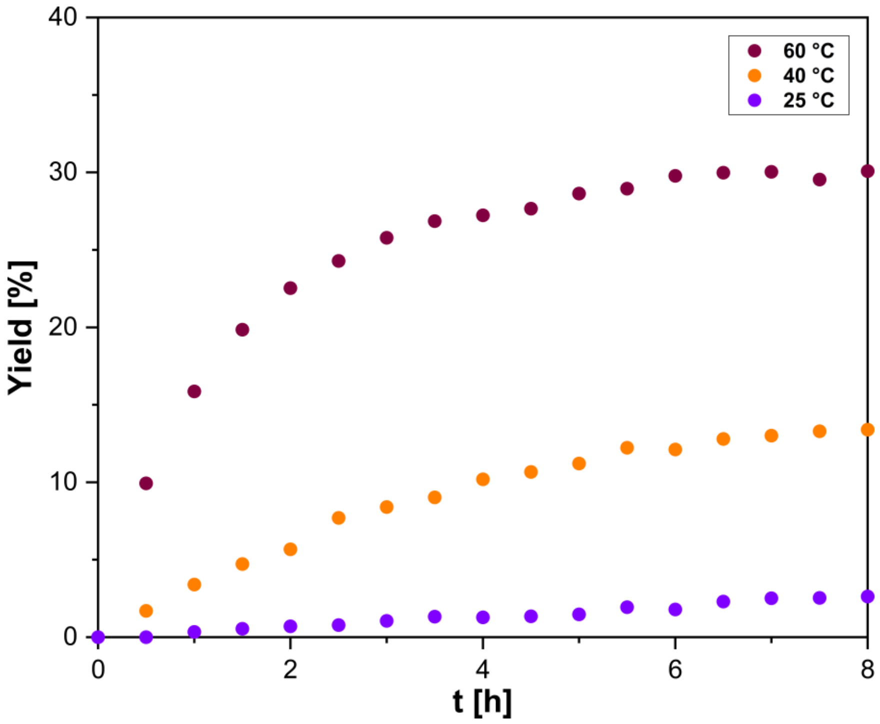

Figure S41: Time course of the formation of product **1b** at different temperatures – catalytic system: Co/DIBAL-H/**L15**.

Variation of the catalyst loading

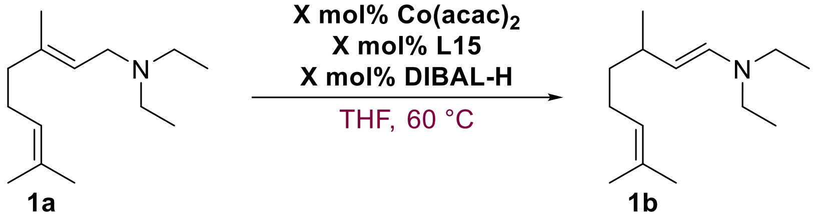

The reaction of the Co/DIBAL-H/**L15** catalytic system was performed according to the general procedure with the following modification:

- Total volume of solvent: 2.4 ml anhydrous THF
  I. 0.2 mol% catalyst loading: 5 µmol [Co]
  II. 0.4 mol% catalyst loading: 10 µmol [Co]
  III. 0.6 mol% catalyst loading: 15 µmol [Co]
- Substrate (**1a**): 643 µL (2.5 mmol)
- 120 µL ISTD (hexadecane)
- Reaction temperature: 60 °C

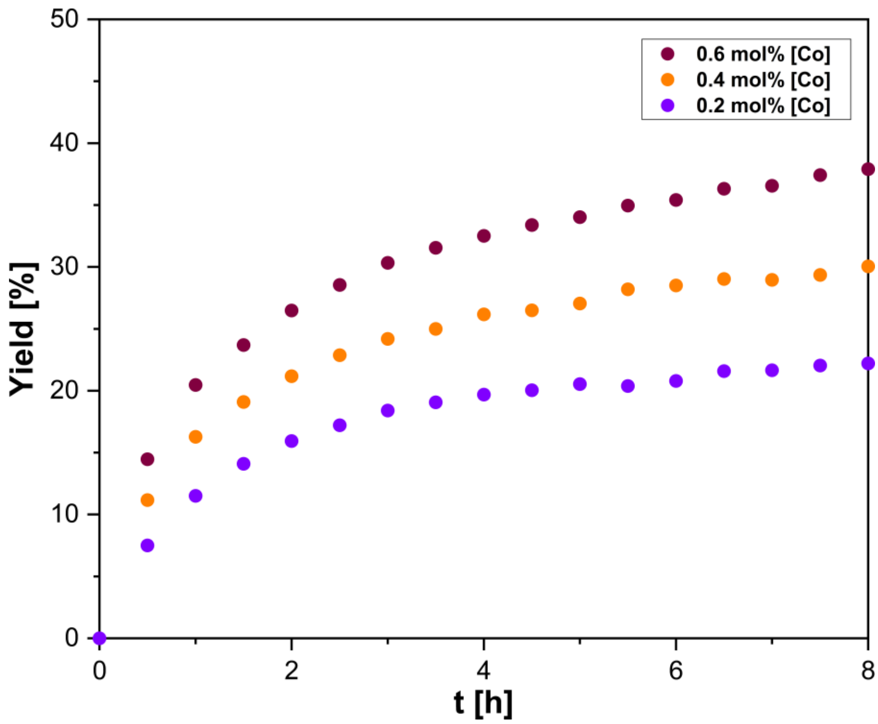

Figure S42: Time course of the formation of product **1b** with different catalyst concentrations – catalytic system: Co/DIBAL-H/**L15**.

VTNA overlay – Determination catalyst order

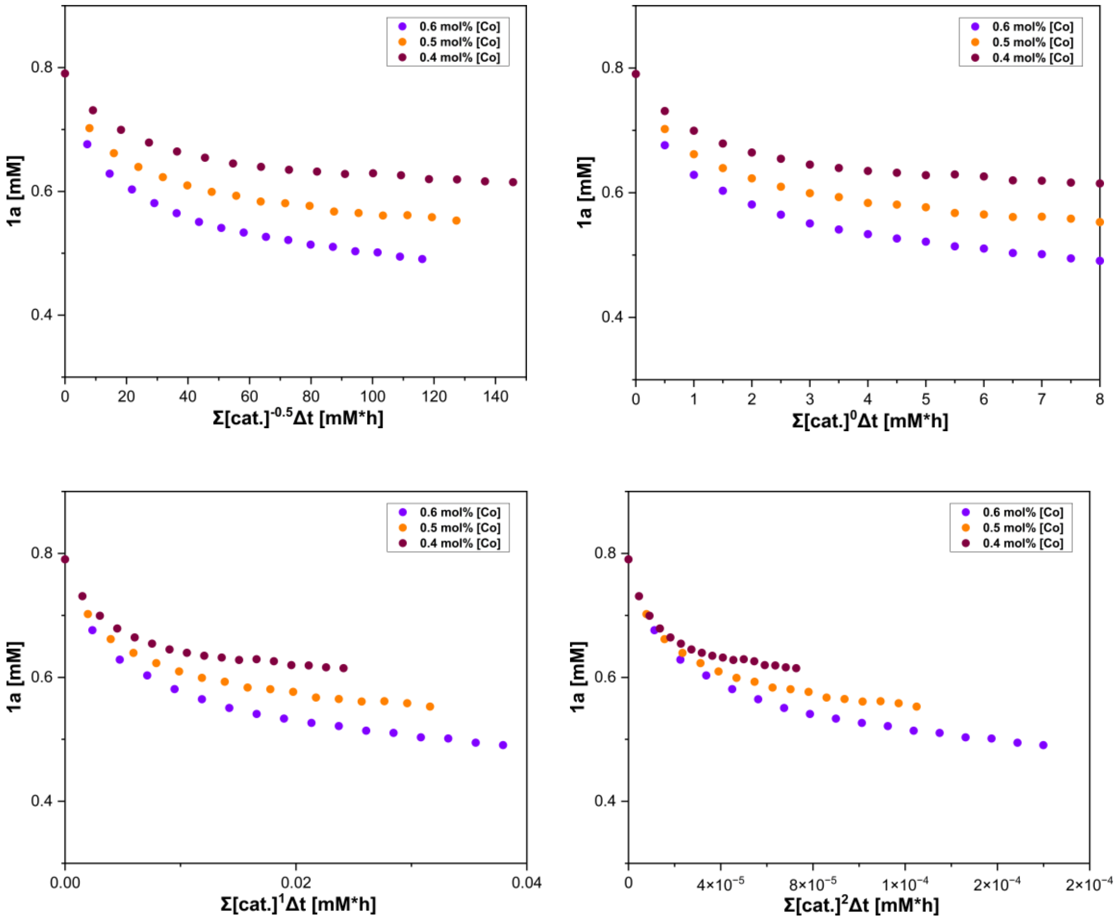

Variation of the substrate concentration

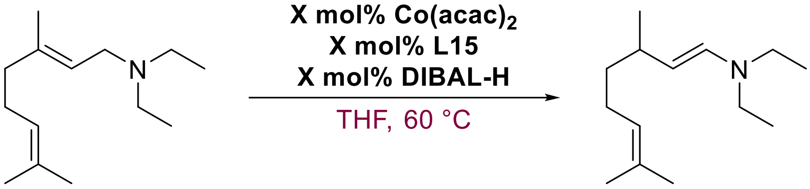

1a **1b**

The reaction of the Co/DIBAL-H/**L15** catalytic system was performed according to the general procedure with the following modification:

- Catalyst: 12.5 µmol [Co]
    I. 2.0 mmol of substrate **1a**: 514 µL + 2.529 mL THF
    II. 2.5 mmol of substrate **1a**: 643 µL + 2.400 mL THF
    III. 3.0 mmol of substrate **1a**: 772 µL + 2271 mL THF
- 120 µL ISTD (hexadecane)
- Reaction temperature: 60 °C

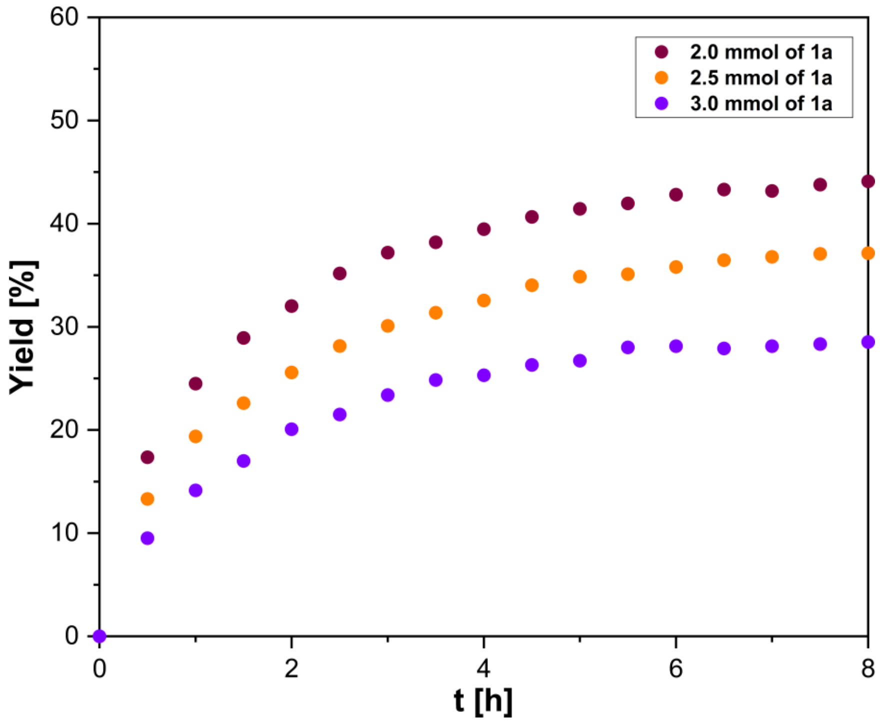

Figure S43: Time course of the formation of product **1b** with different substrate concentrations – catalytic system: Co/DIBAL-H/**L15**.

VTNA overlay – Determination substrate order

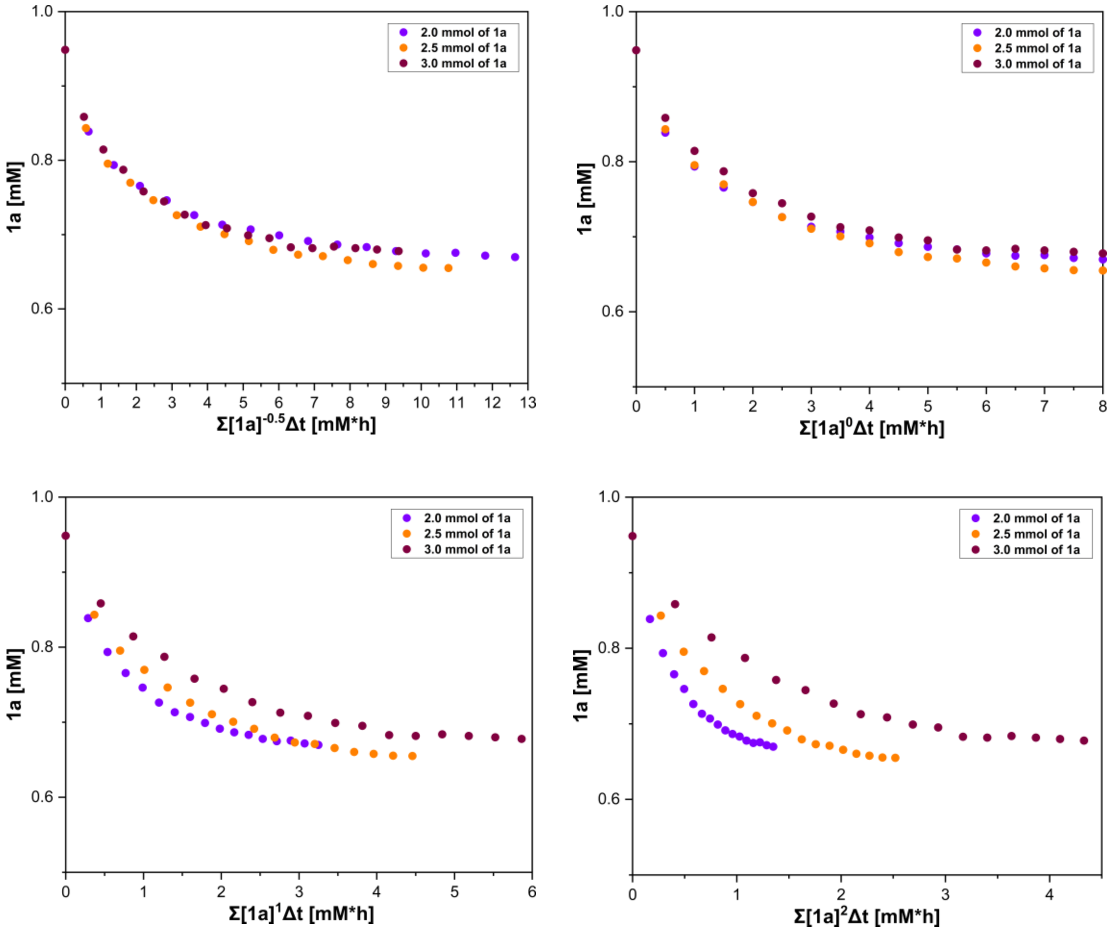

Comparison of the catalyst activity with and without product

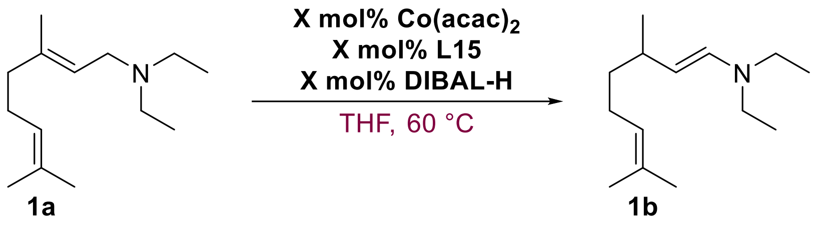

The reaction of the Co/DIBAL-H/**L15** catalytic system was performed according to the general procedure with the following modification:

- Catalyst: 12.5 µmol [Co]
    I. 2.5 mmol of **1a**: 643 µL + 2.400 mL THF
    II. 2.5 mmol of **1a** + 0.5 mmol **1b**: 643 µL (**1a**) + 129 µL (**1b**) + 2.271 mL THF
    III. 3.0 mmol of **1a**: 772 µL +2271 mL THF
- 120 µL ISTD (hexadecane)
- Reaction temperature: 60 °C

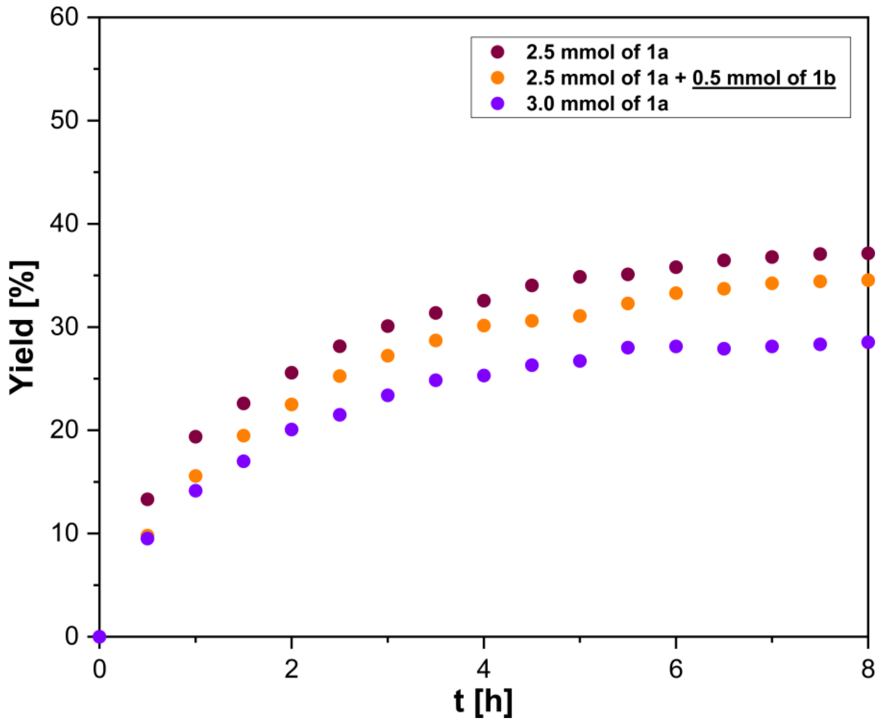

Figure S44: Time course of the formation of product **1b** with and without initial product addition – catalytic system: Co/DIBAL-H/**L15**.

10 Substrate Synthesis - Allylamine Derivatives

10.1 Geranylamine Derivatives

The aliphatic- and aromatic-geranylamine derivatives, that have been used for the catalytic experiments, were mainly synthesized according to the procedure of Craig *et al.* with minor modifications as shown in **Method A.**[14]

Method A:

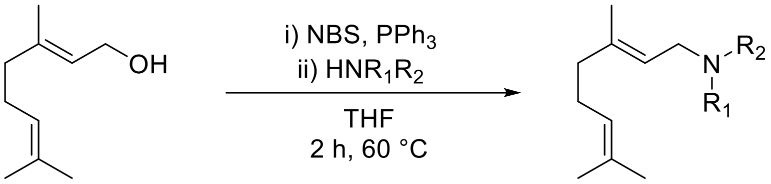

First, a 250 mL round bottom flask was charged with geraniol (1 equiv.), triphenylphosphine (1.1 equiv.) and THF (2-3 mL per 1 mmol). Then, *N*-bromosuccinimide (1.1 equiv.) was added in portions under stirring. After 10 min of stirring, the selected amine (2.1 equiv.) was added dropwise to the reaction mixture and the solution was heated to 60 °C for 2 h. Then, 50 - 100 mL Et_2O were added, and the resulting suspension was filtrated. The filtrate was extracted with 2 M HCl, washed with Et_2O, and made alkaline by the addition of 2M NaOH. The aqueous solution was extracted several times with Et_2O, and the combined organic solutions were dried with magnesium sulphate. After filtration, the solvent was removed *in vacuo*. Finally, the desired allylamine was obtained by fractional distillation or column chromatography as colorless oil.[13]

[13]All geranyl amines were stored in a fridge at 5 °C under argon atmosphere.

(*E*)-*N,N*-diethyl-3,7-dimethylocta-2,6-dien-1-amine ((*E*)-1a)

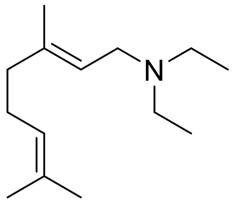

Known Compound.[15] Prepared according to **Method A** with: geraniol (7.71 g, 50 mmol), triphenylphosphine (14.43 g, 55 mmol), *N*-bromosuccinimide (9.79 g, 55 mmol) and diethylamine (7.68 g, 105 mmol).

Chemical Formula: $C_{14}H_{27}N$

Molecular Weight: 209.3770

Appearance: colorless oil

Yield: 83% (8.689 g, 41.5 mmol)

^{1}H NMR (300 MHz, CDCl$_3$): δ = 5.24 (ddq, *J* = 6.8, 5.5, 1.3 Hz, 1H), 5.07 (tdd, *J* = 6.9, 2.9, 1.4 Hz, 1H), 3.04 (dd, *J* = 6.8, 0.9 Hz, 2H), 2.50 (q, *J* = 7.2 Hz, 4H), 2.16 - 1.95 (m, 4H), 1.62 (td, *J* = 11.3, 1.0 Hz, 9H), 1.02 (t, *J* = 7.2 Hz, 6H).

^{13}C NMR (75 MHz, CDCl$_3$): δ = 137.72, 131.55, 124.35, 121.95, 77.58, 77.16, 76.74, 50.67, 46.77, 39.96, 26.55, 25.82, 17.79, 16.40, 11.94.

GC-MS: *m/z* (%): 209.21 (M^+, 14) 208.2 ($[M-H]^+$, 11), 194.17 (18), 141.13 (11), 140.13 (83), 137.12 (6), 126.11 (32), 124.10 (17), 110.09 (12), 95.08 (12), 86.09 (79), 81.06 (41), 74.09 (10), 73.09 (26), 72.08 (25), 69.08 (96), 67.06 (15), 58.07 (100), 56.05 (16).

(*Z*)-*N,N*-diethyl-3,7-dimethylocta-2,6-dien-1-amine ((*Z*)-1a)

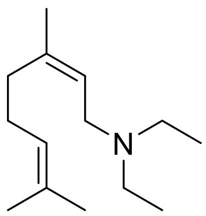

Known Compound.[15] Prepared according to **Method A** with: Nerol (7.71 g, 50 mmol), triphenylphosphine (14.43 g, 55 mmol), *N*-bromosuccinimide (9.79 g, 55 mmol) and diethylamine (7.68 g, 105 mmol).

Chemical Formula: $C_{14}H_{27}N$

Molecular Weight: 209.3770

Appearance: light yellow oil

Yield: 64% (6.721 g, 32.1 mmol)

¹H NMR (300 MHz, CDCl₃): δ = 5.30 - 5.20 (m, 1H), 5.14 - 5.04 (m, 1H), 3.07 (dq, *J* = 6.8, 1.3 Hz, 2H), 2.52 (q, *J* = 7.2 Hz, 4H), 2.05 (d, *J* = 3.3 Hz, 4H), 1.72 (d, *J* = 1.3 Hz, 3H), 1.67 (d, *J* = 1.2 Hz, 3H), 1.60 (d, *J* = 1.3 Hz, 3H), 1.03 (t, *J* = 7.1 Hz, 6H).

^{13}C NMR (75 MHz, $CDCl_3$): δ = 138.12, 131.76, 124.08, 122.22, 50.23, 46.56, 32.20, 26.56, 25.72, 23.56, 17.64, 11.67.

GC-MS: *m/z* (%): 210.16 ($[M+H]^+$, 13), 209.16 (M^+, 75), 194.12 (27), 152.08 (100), 140.08 (36), 139.08 (36), 138.08 (17), 126.07 (37), 124.06 (62), 121.04 (28), 110.04 (15), 95.04 (15), 93.03 (81), 92.05 (15), 86.06 (35), 81.04 (55), 80.04 (26), 74.07 (18), 73.07 (13), 72.06 (25), 69.05 (54), 67.04 (14), 58.06 (72).

(*E*)-*N*-ethyl-3,7-dimethylocta-2,6-dien-1-amine (2a)

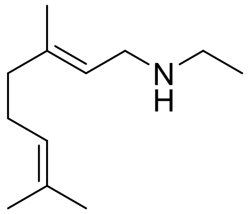

Known Compound.[16] Prepared according to **Method A** with: geraniol (3.86 g, 25 mmol), triphenylphosphine (7.21 g, 27.5 mmol), *N*-bromosuccinimide (4.90 g, 27.5 mmol) and ethanamine (52.5 mmol).

Chemical Formula: $C_{12}H_{23}N$

Molecular Weight: 181.3230

Appearance: colorless oil

Yield: 24% (1.088 g, 6.0 mmol)

^{1}H NMR (300 MHz, $CDCl_3$): δ = 5.37 - 5.20 (m, 1H), 5.15 - 5.02 (m, 1H), 3.22 (dd, *J*=6.8, 0.8, 2H), 2.64 (q, *J*=7.1, 2H), 2.16 - 1.94 (m, 4H), 1.72 - 1.55 (m, 9H), 1.18 - 1.13 (m, 1H), 1.11 (t, *J*=7.1, 3H).

^{13}C NMR (75 MHz, $CDCl_3$): δ = 137.54, 131.63, 124.31, 123.20, 47.29, 43.85, 39.77, 26.65, 25.82, 17.81, 16.40, 15.53.

GC-MS: *m/z* (%): 182.18 ($[M+H]^+$, 3), 181.17 (M^+, 21), 180.16 ($[M-H]^+$, 7), 166.15 (22), 136.11 (14), 124.09 (40), 121.08 (19), 113.10 (22), 112.10 (100), 111.09 (40), 98.09 (77), 96.08 (23), 94.07 (14), 93.06 (97), 82.07 (19), 81.07 (25), 80.06 (20), 69.08 (97).

(*E*)-*N*-ethyl-*N*,3,7-trimethylocta-2,6-dien-1-amine (3a)

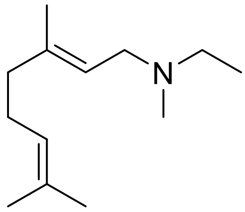

Prepared according to **Method A** with: geraniol (3.86 g, 25 mmol), triphenylphosphine (7.21 g, 27.5 mmol), *N*-bromosuccinimide (4.90 g, 27.5 mmol) and *N*-methylethanamine (52.5 mmol).

Chemical Formula: $C_{13}H_{25}N$

Molecular Weight: 195.3500

Appearance: colorless oil

Yield: 54% (2.637 g, 13.5 mmol)

^{1}H NMR (300 MHz, CDCl$_3$): δ = 5.25 (tq, *J*=7.0, 1.3, 1H), 5.08 (tdd, *J*=6.8, 2.8, 1.4, 1H), 2.94 (d, *J*=6.1, 2H), 2.39 (q, *J*=7.2, 2H), 2.18 (s, 3H), 2.14 - 1.96 (m, 4H), 1.70 - 1.56 (m, 9H), 1.06 (t, *J*=7.2, 3H).

^{13}C NMR (75 MHz, CDCl$_3$): δ = 138.19, 131.61, 124.34, 121.91, 77.59, 77.16, 76.74, 55.10, 51.31, 41.62, 39.95, 26.58, 25.84, 17.81, 16.46, 12.79.

GC-MS: *m/z* (%): 196.25 ($[M+H]^+$, 3), 195.25 (M^+, 16), 194.24 ($[M-H]^+$, 13), 180.21 (14), 127.16 (15), 126.16 (100), 112.14 (29), 110.12 (16), 96.11 (9), 95.11 (8), 93.10 (19), 81.10 (28), 72.12 (95), 69.11 (85), 67.10 (12), 60.12 (18), 59.11 (31), 58.11 (28).

(*E*)-*N,N*,3,7-tetramethylocta-2,6-dien-1-amine (4a)

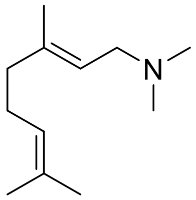

Known Compound.[17] Prepared according **Method A** with: geraniol (7.71 g, 50 mmol), triphenylphosphine (14.43 g, 55 mmol), *N*-bromosuccinimide (9.79 g, 55 mmol) and dimethylamine (105 mmol).

Chemical Formula: $C_{12}H_{23}N$

Molecular Weight: 181.3230

Appearance: colorless oil

Yield: 48% (4.352 g, 24.0 mmol)

^{1}H NMR (300 MHz, CDCl$_3$): δ = 5.24 (tq, *J*=7.0, 1.3, 1H), 5.08 (ddq, *J*=8.3, 5.6, 1.4, 1H), 2.87 (dd, *J*=7.0, 1.0, 2H), 2.21 (s, 6H), 2.15 - 1.95 (m, 4H), 1.72 - 1.53 (m, 9H).

^{13}C NMR (75 MHz, CDCl$_3$): δ = 138.45, 131.62, 124.30, 121.86, 57.17, 45.31, 39.90, 26.59, 25.83, 17.80, 16.44.

GC-MS: *m/z* (%): 181.15 (M$^+$, 10), 180.15 ([M-H]$^+$, 8), 166.13 (5), 124.07 (5), 113.09 (14), 112.08 (100), 111.08 (7), 98.07 (31), 97.06 (5), 96.05 (19), 93.05 (28), 82.04 (10), 81.04 (8), 79.04 (5), 69.05 (45), 68.05 (7), 67.03 (12), 58.04 (69), 53.03 (9).

(*E*)-*N,N*-diisopropyl-3,7-dimethylocta-2,6-dien-1-amine (5a)

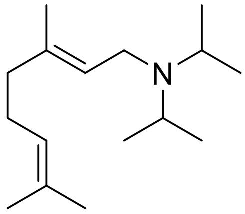

Prepared according to **Method A**[14] with: geraniol (3.08 g, 20 mmol), triphenylphosphine (5.76 g, 22 mmol), *N*-bromosuccinimide (3.91 g, 22 mmol) and diisopropylamine (41.9 mmol).

Chemical Formula: $C_{16}H_{31}N$

Molecular Weight: 237.4310

Appearance: yellowish oil

Yield: 40% (1.91 g, 8.04 mmol)

^{1}H NMR (300 MHz, CDCl$_3$): δ = 5.25 - 5.02 (m, 2H), 3.13 - 2.97 (m, 4H), 2.15 - 1.94 (m, 4H), 1.71 - 1.56 (m, 9H), 1.01 (d, *J* = 6.6 Hz, 12H).

^{13}C NMR (75 MHz, CDCl$_3$): δ = 135.03, 131.43, 126.18, 124.52, 48.26, 43.00, 39.98, 26.63, 25.84, 20.86, 17.81, 16.24.

GC-MS: *m/z* (%): 237.27 (M$^+$, 7), 223.25 (7), 222.26 (38), 168.18 (6),138.13 (3), 137.13 (3), 114.12 (9), 101.12 (9), 100.11 (3), 95.09 (6), 87.11 (6), 86.12 (100), 84.09 (5), 81.08 (29), 70.09 (18), 69.09 (67), 67.07 (6), 55.08 (4).

---

[14]The reaction was carried out at 80°C in a pressure tube.

(*E*)-1-(3,7-dimethylocta-2,6-dien-1-yl)pyrrolidine (6a)

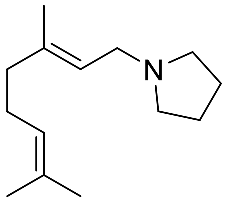

Known Compound.[18] Prepared according to **Method A** with: geraniol (7.71 g, 50 mmol), triphenylphosphine (14.43 g, 55 mmol), *N*-bromosuccinimide (9.79 g, 55 mmol) and pyrrolidine (7.47 g, 105 mmol).

Chemical Formula: $C_{14}H_{25}N$

Molecular Weight: 207.3610

Appearance: colorless to yellowish oil

Yield: 51% (5.288 g, 25.5 mmol)

^{1}H NMR (300 MHz, $CDCl_3$): δ = 5.31 (ddq, *J*=6.9, 5.5, 1.3, 1H), 5.08 (tdd, *J*=5.5, 2.9, 1.4, 1H), 3.07 (dd, *J*=7.0, 0.8, 2H), 2.56 - 2.43 (m, 4H), 2.16 - 1.93 (m, 4H), 1.83 - 1.70 (m, 4H), 1.69 - 1.54 (m, 9H).

^{13}C NMR (75 MHz, $CDCl_3$): δ = 137.39, 131.56, 124.36, 122.11, 77.58, 77.16, 76.74, 54.14, 53.55, 39.88, 26.59, 25.82, 23.58, 17.80, 16.48.

GC-MS: *m/z* (%): 207.19 (M^+,11), 206.19 ($[M-H]^+$, 13), 150.11 (8), 139.12 (14), 138.12 (100), 124.10 (42), 122.09 (22), 96.08 (8), 93.07 (13), 84.08 (67), 81.07 (8), 72.08 (15), 71.08 (34), 70.08 (68), 69.08 (39), 68.08 (10), 67.07 (16), 55.07 (12), 53.06 (11).

(*E*)-4-(3,7-dimethylocta-2,6-dien-1-yl)morpholine (7a)

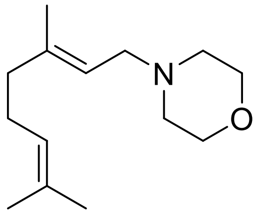

Known Compound.[14] Prepared according to **Method A** with: geraniol (7.71 g, 50 mmol), triphenylphosphine (14.43 g, 55 mmol), *N*-bromosuccinimide (9.79 g, 55 mmol) and morpholine (9.15 g, 105 mmol).

Chemical Formula: $C_{14}H_{25}NO$

Molecular Weight: 223.3600

Appearance: colorless oil

Yield: 46% (5.137 g, 23.0 mmol)

^{1}H NMR (300 MHz, CDCl$_3$): δ = 5.32 - 5.17 (m, 1H), 5.07 (ddt, *J*=6.8, 5.4, 1.5, 1H), 3.78 - 3.65 (m, 4H), 2.96 (dq, *J*=7.0, 0.8, 2H), 2.54 - 2.35 (m, 4H), 2.15 - 1.97 (m, 4H), 1.71 - 1.62 (m, 6H), 1.62 - 1.55 (m, 3H).

^{13}C NMR (75 MHz, CDCl$_3$): δ = 139.42, 131.70, 124.24, 120.45, 67.19, 56.56, 53.75, 39.93, 26.53, 25.82, 17.81, 16.55.

GC-MS: *m/z* (%): 223.20 (M$^+$, 16), 222.19 ([M-H]$^+$, 12), 155.11 (16), 154.12 (100), 140.10 (33), 138.08 (11), 124.11 (60), 100.07 (85), 95.08 (10), 94.07 (10), 93.07 (28), 88.07 (24), 87.07 (88), 86.07 (58), 81.07 (13), 80.06 (8) 79.06 (8), 69.07 (69).

(*E*)-*N*-(3,7-dimethylocta-2,6-dien-1-yl)aniline (8a)

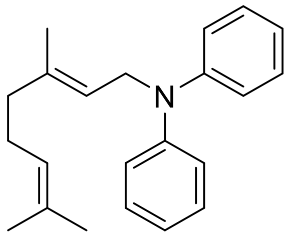

Known Compound.[1] Prepared according to **Method A** with: geraniol (3.86 g, 25 mmol), triphenylphosphine (7.21 g, 27.5 mmol), *N*-bromosuccinimide (4.90 g, 27.5 mmol) and diphenylamine (8.88 g, 52.5 mmol).

Chemical Formula: $C_{22}H_{27}N$

Molecular Weight: 305.4650

Appearance: colorless oil

Yield: 51% (3.369 g, 11.0 mmol)

^{1}H NMR (300 MHz, CD$_2$Cl$_2$): δ = 7.32 - 7.16 (m, 4H), 7.03 - 6.87 (m, 6H), 5.35 (dq, *J*=6.1, 1.3, 1H), 5.05 (tt, *J*=6.8, 1.5, 1H), 4.31 (dd, *J*=6.0, 1.0, 2H), 2.13 - 1.94 (m, 4H), 1.64 (dd, *J*=5.2, 1.2, 6H), 1.58 (q, *J*=0.7, 3H).

^{13}C NMR (101 MHz, CD$_2$Cl$_2$): δ = 148.60, 138.09, 131.85, 129.50, 124.38, 121.83, 121.43, 121.31, 50.87, 39.85, 26.78, 25.79, 17.76, 16.37.

GC-MS: *m/z* (%): 306.23 ([M+H]$^+$, 9), 305.23 (M$^+$, 36), 290.19 (4), 248.14 (7), 236.16 (9), 235.13 (5), 222.14 (8), 194.10 (5), 182.09 (6), 170.09 (15), 169.10 (100), 168.11 (27), 167.10 (39), 166.12 (6), 104.04 (4), 81.07 (7), 77.05 (11), 69.09 (25).

(*E*)-*N*-(3,7-dimethylocta-2,6-dien-1-yl)-*N*-methylaniline (9a)

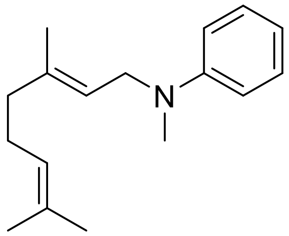

Prepared according to **Method A**[15] with: geraniol (7.71 g, 50 mmol), triphenylphosphine (13.11 g, 55 mmol), *N*-bromosuccinimide (8.90 g, 55 mmol) and *N*-methylaniline (10.71 g, 100 mmol).

Chemical Formula: $C_{17}H_{25}N$

Molecular Weight: 243.3940

Appearance: colorless oil

Yield: 32% (3.89 g, 16.0 mmol)

^{1}H NMR (300 MHz, CD_2Cl_2): δ = 7.26 - 7.11 (m, 2H), 6.72 (dt, *J* = 7.9, 1.0 Hz, 2H), 6.65 (tt, *J* = 7.2, 1.1 Hz, 1H), 5.19 (tq, *J* = 6.3, 1.3 Hz, 1H), 5.07 (ddq, *J* = 8.3, 5.8, 1.4 Hz, 1H), 3.90 (d, *J* = 7.3 Hz, 2H), 2.87 (s, 3H), 2.16 - 1.96 (m, 4H), 1.77 - 1.54 (m, 9H).

^{13}C NMR (101 MHz, CD_2Cl_2): δ = 150.40, 138.51, 131.85, 129.35, 124.42, 121.23, 116.56, 113.28, 50.75, 39.99, 38.07, 26.85, 25.80, 17.76, 16.34.

GC-MS: *m/z* (%): 244.23 ([M+H]$^{+}$, 7), 243.23 (M^{+}, 35), 186.14 (14), 174.14 (21), 173.12 (10), 160.12 (12), 158.10 (7), 132.08 (5), 120.09 (14), 108.09 (10), 107.09 (100), 106.08 (23), 105.07 (5), 104.06 (8), 93.08 (10), 91.07 (5), 81.08 (12), 80.07 (6), 79.07 (7), 77.06 (18), 69.10 (57).

(2*E*,6*E*)-*N*,*N*-diethyl-3,7,11-trimethyldodeca-2,6,10-trien-1-amine (10a)

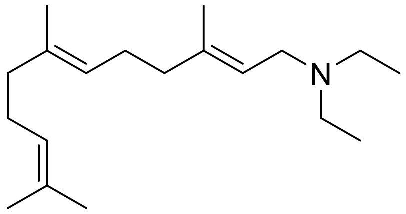

Known Compound. Prepared according to **Method A** with: *trans,trans*-Farnesol (5.56 g, 25 mmol), triphenylphosphine (7.21 g, 27.5 mmol), *N*-bromosuccinimide (4.90 g, 27.5 mmol) and diethylamine (3.84 g, 52.5 mmol).

Chemical Formula: $C_{19}H_{35}N$

Molecular Weight: 277.4960

---

[15]The reaction was carried out at 80°C in a pressure tube.

Appearance: slightly yellow oil

Yield: 39% (2.706 g, 9.75 mmol)

^{1}H NMR (300 MHz, CD_2Cl_2): δ = 5.26 (ddt, *J* = 6.8, 5.5, 1.3 Hz, 1H), 5.09 (dddt, *J* = 9.8, 4.2, 2.9, 1.4 Hz, 2H), 3.06 (dq, *J* = 6.9, 0.9 Hz, 2H), 2.50 (q, *J* = 7.1 Hz, 4H), 2.18 - 1.90 (m, 8H), 1.71 - 1.62 (m, 6H), 1.61 - 1.53 (m, 6H), 1.02 (t, *J* = 7.1 Hz, 6H).

^{13}C NMR (101 MHz, CD_2Cl_2): δ = 137.84, 135.23, 131.40, 124.52, 124.18, 121.84, 50.66, 46.81, 39.96, 39.87, 26.90, 26.54, 25.82, 17.81, 16.48, 16.15, 11.95.

GC-MS: *m/z* (%): 277.23 (M^+, 23), 276.23 ($[M-H]^+$, 11), 262.21 (15), 209.18 (15), 208.17 (82), 141.11 (14), 140.11 (100), 139.10 (13), 138.09 (12), 137.09 (11), 126.09 (85), 124.08 (21), 110.06 (18), 95.05 (12), 93.03 (16), 86.07 (57), 81.03 (44), 74.07 (21), 73.05 (30), 72.05 (27), 69.05 (87), 67.03 (21), 58.04 (74), 56.03 (16).

10.2 Aliphatic and Aromatic Substrates

The aliphatic and aromatic allylamine derivatives **11a-31a** were mainly synthesized, according to the procedure of **Method B**, starting from commercially available carboxylic acids, *α,β*-unsaturated esters or *α,β*-unsaturated alcohol.

Method B:

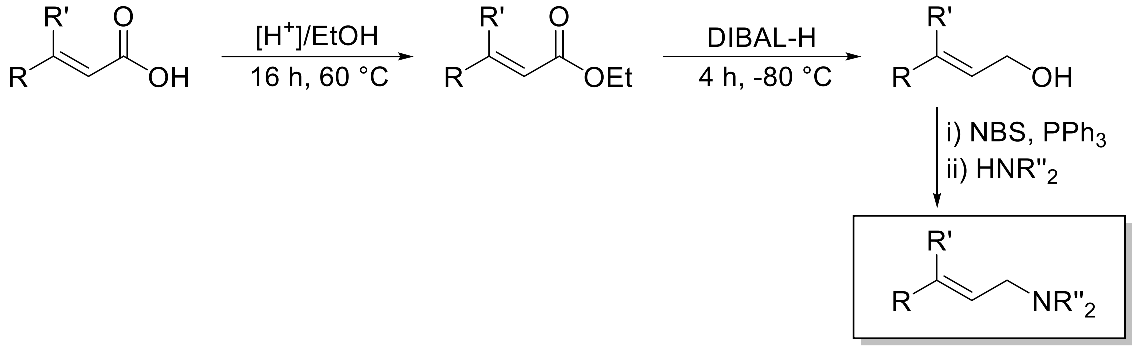

The carboxylic acid was charged together with a stirring bar in a Schlenk flask. After, flushing the flask with argon anhydrous EtOH was added, and the suspension was stirred for approximately 5 min. Then, catalytic amounts of concentrated H_2SO_4 were slowly added and the mixture was heated at 60 °C overnight (16 h). After the reaction mixture was cooled down to rt, the solution was passed over a pad of silica. The pure *α,β*-unsaturated ester was obtained without further purification by removing the solvent.

Next, the selected *α,β*-unsaturated ester was charged together with a stirring bar in a Schlenk flask. Then, anhydrous DCM was injected, and the solution was cooled to -78 °C. DIBAL-H (1 M in *n*-hexane) was dropwise added and the solution was allowed to warm slowly to room temperature. After, full consumption of the *α,β*-unsaturated ester was detected, the reaction was quenched with MeOH, and the mixture was stirred for 1 h. The *α,β*-unsaturated alcohol was then extracted with DCM, and the organic layer was washed with saturated aqueous NaCl solution. The desired *α,β*-unsaturated alcohol was purified by fractional distillation or column chromatography.

The allylamine synthesis was performed according to the procedure of Craig *et al.* with small modifications.[14] The selected *α,β*-unsaturated alcohol (1 equiv.) was charged with triphenylphosphine (1.1 equiv.) and THF (2 mL per 1 mmol). Then, *N*-bromosuccinimide (1.1 equiv.) was added in portions under stirring. After 10 min of stirring, the selected amine (2.1 equiv.) was added dropwise to the reaction mixture, and the solution was heated to 60 °C for 2 h. Then, 50 - 100 mL Et_2O were added, and the resulting

suspension was filtrated. The filtrate was extracted with 1M HCl, washed with Et_2O and made alkaline by the addition of 2M NaOH. The aqueous solution was extracted with Et_2O, and the combined organic solutions were dried with sodium sulphate. After, filtration the solvent was removed *in vacuo*. Finally, the desired allylamine was obtained by fractional distillation or column chromatography.[14] After purification, all synthesized allylamine derivatives were dried and stored for at least three days over 3 Å molecular sieves under an argon atmosphere.

(*E*)-*N,N*-diethylhex-2-en-1-amine (11a)

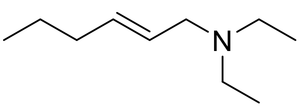

Known compound.[19] Prepared according to **Method B** with: (*E*)-hex-2-en-1-ol (5.01 g, 50 mmol), triphenylphosphine (14.425 g, 55 mmol), *N*-bromosuccinimide (9.790 g, 55 mmol) and diethylamine (8.776 g, 120 mmol).

Chemical Formula: $C_{10}H_{21}N$

Molecular Weight: 155.2850

Appearance: colorless oil

Isolated Yield: 69% (5.358 g, 34.50 mmol)

^{1}H NMR (300 MHz, $CDCl_3$): δ = 5.66 - 5.38 (m, 2H), 3.03 (d, *J*=5.5, 2H), 2.51 (q, *J*=7.2, 4H), 2.09 - 1.92 (m, 2H), 1.39 (h, *J*=7.3, 2H), 1.01 (t, *J*=7.2, 6H), 0.88 (t, *J*=7.4, 3H).

^{13}C NMR (75 MHz, $CDCl_3$): δ = 133.81, 127.18, 55.33, 46.55, 34.64, 22.57, 13.79, 11.77.

GC-MS: *m/z* (%): 155.14 (M^+, 31), 154.14 ($[M-H]^+$, 16), 141.13 (11), 140.12 (100), 126.11 (12), 112.09 (22), 110.07 (8), 98.08 (8), 96.07 (9), 86.08 (54), 84.07 (6), 83.07 (34), 73.07 (22), 72.06 (16), 67.04 (6), 58.05 (96), 56.04 (22), 55.04 (62), 53.04 (6).

(*E*)-3-cyclohexyl-*N*,*N*-diethylprop-2-en-1-amine (12a)

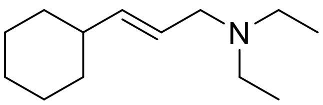

Known compound.[20] Prepared according to **Method B.** First, ethyl (*E*)-3-cyclohexylacrylate was prepared from the corresponding carboxylic acid in ethanol with quantitative yield. Then, the *α,β*-unsaturated ester (3.00 g, 16.46 mmol) was reduced with DIBAL-H (41.15 mmol, 2.5 equiv.) as described in **Method B**, yielding (*E*)-3-cyclohexylprop-2-en-1-ol as colorless oil (1.73 g, 12.35 mmol, 75% yield). Finally, **12a** was obtained from the reaction of: (*E*)-3-cyclohexylprop-2-en-1-ol (1.73 g, 12.35 mmol), triphenylphosphine (3.562 g, 13.58 mmol), *N*-bromosuccinimide (2.417 g, 13.58 mmol) and diethylamine (1.896 g, 25.92 mmol).

Chemical Formula: $C_{13}H_{25}N$

Molecular Weight: 195.3500

Appearance: colorless oil

Isolated Yield: 58% (1.399 g, 7.16 mmol)

^{1}H NMR (300 MHz, CDCl$_3$): δ = 5.64 - 5.34 (m, 2H), 3.08 - 2.98 (m, 2H), 2.50 (q, *J*=7.1, 4H), 1.77 - 1.59 (m, 5H), 1.36 - 1.05 (m, 5H), 1.01 (t, *J*=7.2, 6H).

^{13}C NMR (75 MHz, CDCl$_3$): δ = 139.87, 124.40, 55.51, 46.55, 40.62, 33.12, 26.36, 26.19, 11.79.

GC-MS: *m/z* (%): 196.23 ([M+H]$^+$, 4), 195.23 (M$^+$, 23), 194.22 ([M-H]$^+$, 15), 181.21 (17), 180.23 (100), 123.13 (21), 122.12 (12), 112.13 (45), 98.11 (5), 86.12 (46), 82.09 (7), 81.10 (71), 79.08 (11), 74.11 (13), 73.11 (10), 72.10 (20), 69.09 (7), 67.08 (28), 58.10 (64), 56.08 (10), 55.09 (9).

(*E*)-*N,N*-diethyl-4,4,4-trifluorobut-2-en-1-amine (13a)

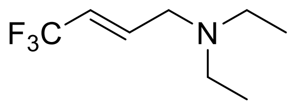

First, (*E*)-4,4,4-trifluorobut-2-en-1-ol was prepared according to the literature.[21] Afterwards, **13a** was synthesized following the procedure of **Method B** with: (*E*)-4,4,4-trifluorobut-2-en-1-ol (1.40 g, 11.10 mmol), triphenylphosphine (3.204 g, 12.21 mmol), *N*-bromosuccinimide (2.174 g, 12.21 mmol) and diethylamine (1.705 g, 23.32 mmol).

Chemical Formula: $C_8H_{14}F_3N$

Molecular Weight: 181.2022

Appearance: colorless oil

Isolated Yield: 51% (1.020 g, 5.63 mmol)

^{1}H NMR (300 MHz, CDCl$_3$): δ = 6.42 (dtq, *J*=16.0, 5.9, 2.1, 1H), 5.82 (dqt, *J*=15.7, 6.4, 1.7, 1H), 3.22 - 3.11 (m, 2H), 2.51 (q, *J*=7.1, 4H), 1.02 (t, *J*=7.2, 6H).

^{13}C NMR (75 MHz, CDCl$_3$): δ = 138.84 (q, *J*=6.2), 121.40, 120.54, 120.10, 119.65, 119.21, 53.75, 47.30, 12.04.

^{19}F NMR (282 MHz, CDCl$_3$): δ = -64.03.

GC-MS: *m/z* (%): 182.17 ([M+H]$^+$, 2), 181.17 (M$^+$, 22), 180.17 ([M-H]$^+$, 5), 167.16 (11), 166.18 (100), 162.17 (7), 152.12 (2), 138.09 (15), 109.06 (11), 98.09 (2), 91.07 (7), 89.06 (6), 86.13 (6), 59.08 (2), 58.11 (2), 56.10 (7).

HRMS (ESI): *m/z* calcd. for $C_8H_{14}F_3N$: 182.1151 [M+H]$^+$, found: 182.1154.

Ethyl (*E*)-4-(diethylamino)but-2-enoate (14a)

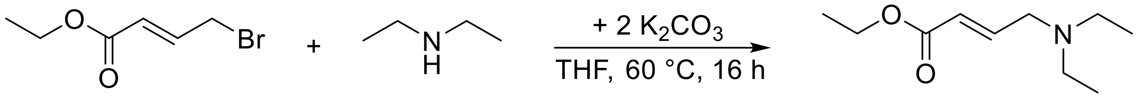

A 100 mL round bottom flask was charged with ethyl (*E*)-4-bromobut-2-enoate (3.50 g, 18.13 mmol, 1 equiv.) and K_2CO_3 (6.26 g, 45.33 mmol, 2.5 equiv.). After 50 mL THF were added, diethylamine (1.46 g, 19.94 mmol, 1.1 equiv.) was dropwise injected and the reaction mixture was heated for 2 h at 50 °C. Next, 20 mL of water and 40 mL of ethyl acetate were added for extraction, and the organic layer was washed once with 20 mL of saturated brine, dried over anhydrous sodium sulfate, and concentrated under reduced pressure. The received orange crude oil was purified by column chromatography followed by vacuum distillation.

Chemical Formula: $C_{10}H_{19}NO_2$

Molecular Weight: 185.2670

Appearance: yellowish oil

Isolated Yield: 34% (1.14 g, 6.15 mmol)

^{1}H NMR (300 MHz, CDCl$_3$): δ = 6.98 (dt, *J* = 15.7, 6.2 Hz, 1H), 5.97 (dt, *J* = 15.7, 1.7 Hz, 1H), 4.18 (q, *J* = 7.1 Hz, 2H), 3.21 (dd, *J* = 6.2, 1.7 Hz, 2H), 2.52 (q, *J* = 7.1 Hz, 4H), 1.28 (t, *J* = 7.1 Hz, 3H), 1.02 (t, *J* = 7.1 Hz, 6H).

^{13}C NMR (75 MHz, CDCl$_3$): δ = 166.50, 146.69, 122.86, 60.41, 54.26, 47.32, 14.38, 12.06.

GC-MS: *m/z* (%): 186.13 ([M+H]$^+$, 2), 185.13 (M$^+$, 17), 184.13 ([M-H]$^+$, 3), 171.11 (11), 170.11 (100), 157.10 (3), 156.09 (34), 142.08 (16), 140.09 (13), 114.07 (3), 112.08 (13), 110.08 (4), 96.07 (4), 86.09 (14), 85.03 (23), 82.06 (4), 68.04 (5).

(*E*)-*N,N*-diethyl-2-methylbut-2-en-1-amine (15a)

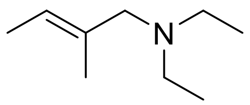

(*E*)-2-methylbut-2-en-1-ol was synthesized by reduction of (*E*)-2-methylbut-2-enal (3.00 g, 35.66 mmol, 1.0 equiv.) with sodium borohydride (1.35 g, 35.66 mmol, 1.0 equiv.) in MeOH at 0 °C. The (*E*)-2-methylbut-2-en-1-ol was obtained after distillation as colorless oil (2.87 g, 33.32 mmol, 93% yield).[22] Afterwards, **15a** was synthesized following the procedure of **Method B** with: (*E*)-2-methylbut-2-en-1-ol (2.10 g, 24.38 mmol), triphenylphosphine (7.03 g, 26.82 mmol), *N*-bromosuccinimide (4.77 g, 26.82 mmol) and diethylamine (3.75 g, 51.20 mmol).

Chemical Formula: $C_9H_{19}N$

Molecular Weight: 141.2580

Appearance: colorless oil

Isolated Yield: 72% (2.479 g, 17.55 mmol)

^{1}H NMR (300 MHz, $CDCl_3$): δ = 5.42 - 5.28 (m, 1H), 2.90 - 2.83 (m, 2H), 2.44 (q, *J*=7.1, 4H), 1.66 - 1.55 (m, 6H), 0.98 (t, *J*=7.1, 6H).

^{13}C NMR (75 MHz, $CDCl_3$): δ = 134.64, 121.12, 77.58, 77.16, 76.74, 62.28, 46.59, 14.85, 13.41, 11.68.

GC-MS: *m/z* (%): 142.12 ($[M+H]^+$, 3), 141.12 (M^+, 26), 140.12 ($[M-H]^+$, 3), 127.10 (5), 126.10 (48), 110.06 (3), 87.09 (6), 86.08 (100), 82.05 (3), 73.06 (13), 72.05 (11), 70.06 (5), 69.05 (40), 67.03 (3), 58.04 (56), 56.03 (10), 55.05 (4), 53.04 (4).

***N,N*-diethylcyclohex-2-en-1-amine (16a)**

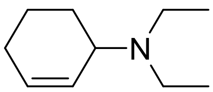

Known compound.[19] Prepared according to **Method B** with: cyclohex-2-en-1-ol (2.00 g, 20.38 mmol), triphenylphosphine (5.879 g, 22.41 mmol), *N*-bromosuccinimide (3.990 g, 22.41 mmol) and diethylamine (3.130 g, 42.79 mmol).

Chemical Formula: $C_{10}H_{19}N$

Molecular Weight: 153.2690

Appearance: colorless oil

Isolated Yield: 43% (1.34 g, 8.76 mmol)

^{1}H NMR (300 MHz, $CDCl_3$): δ = 5.66 - 5.38 (m, 2H), 3.03 (d, *J*=5.5, 2H), 2.51 (q, *J*=7.2, 4H), 2.09 - 1.92 (m, 2H), 1.39 (h, *J*=7.3, 2H), 1.01 (t, *J*=7.2, 6H), 0.88 (t, *J*=7.4, 3H).

^{13}C NMR (75 MHz, $CDCl_3$): δ = 133.81, 127.18, 55.33, 46.55, 34.64, 22.57, 13.79, 11.77.

GC-MS: *m/z* (%): 154.13 (([M+H]$^+$, 4), 153.13 (M$^+$, 36), 152.14 ([M-H]$^+$, 5), 138.11 (13), 126.12 (6), 125.11 (64), 124.13 (7), 111.10 (9), 110.10 (100), 96.08 (15), 84.08 (5), 82.07 (10), 81.07 (36), 79.07 (16).

***N,N*-diethyl-3-methylbut-2-en-1-amine (17a)**

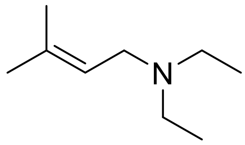

Known compound. Prepared according to **Method B** with: 3-methylbut-2-en-1-ol (2.50 g, 29.026 mmol), tri-phenylphosphine (8.374 g, 31.93 mmol), *N*-bromosuccin-imide (5.683 g, 31.93 mmol) and diethylamine (4.458 g, 60.95 mmol).

Chemical Formula: $C_9H_{19}N$

Molecular Weight: 141.2580

Appearance: colorless oil

Isolated Yield: 68% (3.856 g, 19.74 mmol)

^{1}H NMR (300 MHz, $CDCl_3$) δ = 5.25 (tdt, *J*=6.9, 2.9, 1.4, 1H), 3.03 (dp, *J*=7.0, 1.1, 2H), 2.50 (q, *J*=7.2, 4H), 1.72 (q, *J*=1.3, 3H), 1.64 (d, *J*=1.5, 3H), 1.02 (t, *J*=7.2, 6H).

^{13}C NMR (75 MHz, $CDCl_3$) δ = 134.28, 122.02, 50.73, 46.79, 26.08, 18.08, 11.95.

GC-MS: *m/z* (%): 142.14 ([M+H]$^+$, 3), 141.14 (M$^+$, 30), 140.13 ([M-H]$^+$, 15), 127.12 (3), 126.12 32, 110.09 (3), 98.09 (8), 86.10 (25), 82.07 (4), 73.10 (24), 72.09 (15), 70.09 (6), 69.08 (59), 67.07 (4), 59.09 (4), 58.09 (100), 57.16 (3), 56.07 (12), 53.07 (4).

(*E*)-*N,N*-diethyl-3-methylhept-2-en-1-amine (18a)

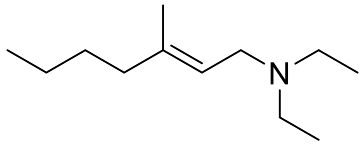

Ethyl (*E*)-3-methylhept-2-enoate was synthesized according to the literature.[23] The *α,β*-unsaturated ester (3.405 g, 20.0 mmol) was reduced with DIBAL-H (50.0 mmol, 2.5 equiv.) as described in **Method B**, yielding (*E*)-3-cyclohexylprop-2-en-1-ol as colorless oil (2.077 g, 16.20 mmol, 81% yield). The desired product **18a** was obtained by **Method B** with: (*E*)-3-methylhept-2-en-1-ol (2.07 g, 16.14 mmol), triphenylphosphine (4.658 g, 17.76 mmol), *N*-bromosuccinimide (3.161 g, 17.76 mmol) and diethylamine (2.480 g, 33.90 mmol).

Chemical Formula: $C_{12}H_{25}N$

Molecular Weight: 183.3390

Appearance: colorless oil

Isolated Yield: 70% (2.071 g, 11.30 mmol)

^{1}H NMR (300 MHz, $CDCl_3$): δ = 5.24 (tq, *J*=6.8, 1.3, 1H), 3.05 (dd, *J*=6.8, 0.9, 2H), 2.50 (q, *J*=7.1, 4H), 2.05 - 1.94 (m, 2H), 1.62 (d, *J*=2.2, 3H), 1.45 - 1.20 (m, 4H), 1.02 (t, *J*=7.2, 6H), 0.88 (t, *J*=7.1, 3H).

^{13}C NMR (75 MHz, $CDCl_3$): δ = 138.17, 121.58, 50.66, 46.82, 39.64, 30.14, 22.45, 16.37, 14.12, 11.96.

GC-MS: *m/z* (%): 183.17 (M^+, 31), 182.16 ($[M-H]^+$, 20), 168.15 (18),140.11 (19),126.10 (39), 124.08 (7), 111.09 (14), 110.07 (7), 98.07 (10), 86.08 (36), 73.06 (25), 72.06 (18), 70.05 (7), 69.05 (61), 67.04 (9), 58.05 (100), 57.06 (6), 56.04 (14), 55.04 (31).

***N,N*-diethylprop-2-en-1-amine (19a)**

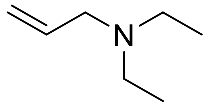

Known compound.[1] Prepared according to **Method B** with: prop-2-en-1-ol (2.00 g, 34.44 mmol), triphenylphosphine 9.935 g, 37.88 mmol), *N*-bromosuccinimide (6.742 g, 37.88 mmol) and diethylamine (5.289 g, 72.31 mmol).

Chemical Formula: $C_7H_{15}N$

Molecular Weight: 113.2040

Appearance: colorless oil

Isolated Yield: 57% (2.222 g, 19.63 mmol)

^{1}H NMR (300 MHz, $CDCl_3$): δ = 5.96 - 5.77 (m, 1H), 5.25 - 5.05 (m, 2H), 3.08 (dt, *J*=6.6, 1.3, 2H), 2.51 (q, *J*=7.2, 4H), 1.02 (t, *J*=7.2, 6H).

^{13}C NMR (75 MHz, $CDCl_3$): δ = 117.16, 56.37, 46.73, 11.80.

GC-MS: *m/z* (%): 114.11 ($[M+H]^+$, 2), 113.11 (M^+, 27), 112.12 ($[M-H]^+$, 5) 99.11 (8), 98.11 (100), 96.09 (2), 87.11 (1), 86.11 (21), 84.09 (2), 82.08 (1), 70.07 (8), 68.06 (2), 58.08 (7), 57.08 (2), 56.07 (22), 55.10 (1), 54.06 (2).

***N,N*-diethylbut-3-en-1-amine (20a)**

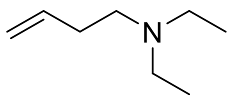

Known compound.[24] Prepared according to **Method B** with: but-3-en-1-ol (2.50 g, 34.67 mmol), triphenylphosphine (10.002 g, 38.14 mmol), *N*-bromosuccinimide (6.788 g, 38.14 mmol) and diethylamine (5.325 g, 72.81 mmol).

Chemical Formula: $C_8H_{17}N$

Molecular Weight: 127.2310

Appearance: colorless oil

Isolated Yield: 66% (2.911 g, 22.88 mmol)

^{1}H NMR (300 MHz, $CDCl_3$) δ = 5.80 (ddt, *J*=17.0, 10.2, 6.8, 1H), 5.13 - 4.92 (m, 2H), 2.63 - 2.43 (m, 6H), 2.27 - 2.13 (m, 2H), 1.02 (t, *J*=7.2, 6H).

^{13}C NMR (75 MHz, $CDCl_3$) δ = 137.19, 115.50, 52.42, 46.97, 31.56, 11.86.

GC-MS: *m/z* (%): 127.17 (M^+, 1), 126.16 ($[M-H]^+$, 1), 112.14 (3), 110.13 (1), 96.12 (1), 87.14 (10), 86.18 (100), 84.13 (2), 72.12 (1), 70.11 (1), 69.11 (1), 59.11 (1), 58.11 (26), 57.13 (1), 56.10 (5), 55.10 (7).

N,N-diethylpent-4-en-1-amine (21a)

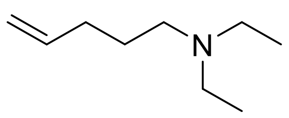

Known compound.[24] Prepared according to **Method B** with: pent-4-en-1-ol (2.50 g, 29.03 mmol), triphenylphosphine (8.374 g, 31.93 mmol), *N*-bromosuccinimide (5.683 g, 31.93 mmol) and diethylamine (4.458 g, 60.95 mmol).

Chemical Formula: $C_9H_{19}N$

Molecular Weight: 141.2580

Appearance: colorless oil

Isolated Yield: 61% (2.501 g, 17.71 mmol)

^{1}H NMR (300 MHz, $CDCl_3$): δ = 5.81 (ddt, *J*=16.8, 10.2, 6.6, 1H), 5.08 - 4.88 (m, 2H), 2.50 (q, *J*=7.2, 4H), 2.45 - 2.36 (m, 2H), 2.10 - 1.98 (m, 2H), 1.60 - 1.46 (m, 2H), 1.00 (t, *J*=7.2, 6H).

^{13}C NMR (75 MHz, $CDCl_3$): δ = 138.85, 114.56, 52.53, 47.07, 31.94, 26.37, 11.86.

GC-MS: *m/z* (%): 141.14 (M^+, 2), 140.14 ($[M-H]^+$, 1), 127.12 (1), 126.12 (10), 113.11 (5), 112.11 (1), 99.10 (1), 98.09 (1), 87.11 (7), 86.13 (100), 84.10 (2), 72.10 (16), 71.11 (2), 70.09 (1), 69.09 (1), 58.09 (14), 57.11 (1), 56.08 (5).

(*E*)-*N,N*-diethyl-3-phenylprop-2-en-1-amine (22a)

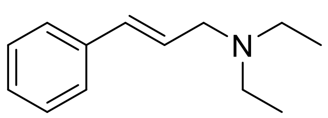

The synthesis of **22a** was performed according to the procedure of Limberger *et al.* [25] (*E*)-(3-chloroprop-1-en-1-yl)benzene (20 g, 0.13 mol, 1 equiv.) and potassium carbonate (72.4 g, 0.52 mol, 4 equiv.) were weighed in a 500 mL round bottom flask, followed by the addition of 150 mL of toluene and 150 mL *tert*-butyl alcohol. Under stirring, diethylamine (38.3 g, 0.52 mol, 4 equiv.) was added and the reaction mixture was heated for 24 h at 90 °C. After, filtration and removing the solvent *in vacuo*, a yellowish oil was obtained. The product was extracted with 1M HCl, washed with Et_2O and made alkaline by the addition of 2M NaOH. The aqueous solution was extracted with Et_2O, and the combined organic solutions were dried with sodium sulphate. After filtration, the solvent was removed *in vacuo*, yielding an orange oil. Finally, **22a** was obtained by vacuum distillation.

Chemical Formula: $C_{13}H_{19}N$

Molecular Weight: 189.3020

Appearance: colorless oil

Isolated Yield: 97% (24.1 g, 0.127 mmol)

^{1}H NMR (300 MHz, CD_2Cl_2): δ = 7.43 - 7.36 (m, 2H), 7.36 - 7.27 (m, 2H), 7.27 - 7.13 (m, 1H), 6.52 (d, *J*=15.9, 1H), 6.28 (dt, *J*=15.9, 6.5, 1H), 3.22 (dd, *J*=6.5, 1.4, 2H), 2.54 (q, *J*=7.1, 4H), 1.03 (t, *J*=7.1, 6H).

^{13}C NMR (75 MHz, CD_2Cl_2): δ = 137.83, 131.87, 128.92, 128.89, 127.55, 126.55, 55.92, 47.16, 12.15.

GC-MS: *m/z* (%): 190.16 ($[M+H]^+$, 3), 189.16 (M^+, 22), 188.16 ($[M+H]^+$, 11), 174.13 (16), 160.11 (7), 118.09 (13), 117.1 (100), 116.10 (10), 115.08 (42), 98.10 (32), 91.07 (20), 89.05 (3), 86.11 (10), 77.05 (4), 65.05 (3), 56.06 (7).

(*E*)-3-(4-(tert-butyl)phenyl)-*N,N*-diethylprop-2-en-1-amine (23a)

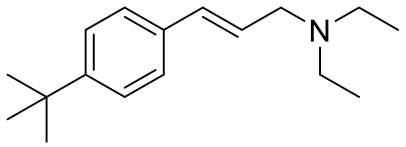

Prepared according to **Method B.** First, ethyl (*E*)-3-(4-(tert-butyl)phenyl)acrylate was prepared from the corresponding carboxylic acid in ethanol. Then, the *α,β*-unsaturated ester (3.5 g, 15.07 mmol) was reduced with DIBAL-H (37.66 mmol, 2.5 equiv.) as described in **Method B**, yielding (*E*)-3-(4-(tert-butyl)phenyl)prop-2-en-1-ol (2.379 g, 12.50 mmol, 83% yield). Finally, **23a** was obtained from the reaction of: (*E*)-3-(4-(tert-butyl)phenyl)prop-2-en-1-ol (2.379 g, 12.50 mmol), triphenylphosphine (3.606 g, 13.75 mmol), *N*-bromosuccinimide (2.447g, 13.75 mmol) and diethylamine (1.920 g, 26.25 mmol).

Chemical Formula: $C_{17}H_{27}N$

Molecular Weight: 245.4100

Appearance: colorless oil

Isolated Yield: 70% (2.147 g, 8.75 mmol)

^{1}H NMR (300 MHz, CD_2Cl_2): δ = 7.30 - 7.17 (m, 4H), 6.40 (dtd, *J*=15.9, 1.4, 0.4, 1H), 6.14 (dt, *J*=15.9, 6.6, 1H), 3.12 (dd, *J*=6.6, 1.4, 2H), 2.45 (q, *J*=7.2, 4H), 1.22 (s, 9H), 0.93 (t, *J*=7.1, 6H).

^{13}C NMR (75 MHz, CD_2Cl_2): δ = 150.74, 135.00, 131.74, 127.93, 126.24, 125.82, 55.94, 47.11, 34.81, 31.44, 12.11.

GC-MS: *m/z* (%): 246.31 ($[M+H]^+$, 10), 245.30 (M^+, 50), 244.30 ($[M+H]^+$, 24), 230.28 (14), 216.26 (29), 188.2 (15),173.19 (22), 161.18 (11), 143.14 (8), 129.11 (8), 128.11 (13), 117.11 (30), 115.10 (16), 107.64 (13), 99.15 (8), 98.15 (100), 93.63 (19), 86.15 (14).

HRMS (ESI): *m/z* calcd. for $C_{17}H_{27}N$: 246.2216 $[M+H]^+$, found: 246,2222.

(*E*)-3-(4-chlorophenyl)-*N,N*-diethylprop-2-en-1-amine (24a)

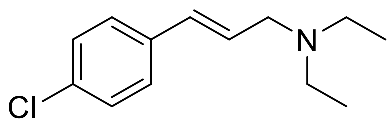

Prepared according to **Method B.** The ethyl (*E*)-3-(4-chlorophenyl)acrylate was prepared from the corresponding carboxylic acid in ethanol. Then, the *α,β*-unsaturated ester (3.0 g, 14.24 mmol) was reduced with DIBAL-H (35.60 mmol, 2.5 equiv.) as described in **Method B**, yielding (*E*)-3-(4-chlorophenyl)prop-2-en-1-ol (1.90 g, 11.27 mmol, 79% yield). Finally, **24a** was obtained from the reaction of: (*E*)-3-(4-chlorophenyl)prop-2-en-1-ol (1.90 g, 11.27 mmol), triphenylphosphine (3.25 g, 12.40 mmol), *N*-bromosuccinimide (2.21 g, 12.40 mmol) and diethylamine (1.731 g, 23.66 mmol).

Chemical Formula: $C_{13}H_{18}ClN$

Molecular Weight: 223.7440

Appearance: colorless oil

Isolated Yield: 58% (1.604 g, 6.54 mmol)

^{1}H NMR (300 MHz, CD_2Cl_2): δ = 7.38 - 7.22 (m, 4H), 6.48 (dt, *J*=15.9, 1.4, 1H), 6.27 (dt, *J*=15.9, 6.5, 1H), 3.21 (dd, *J*=6.5, 1.4, 2H), 2.53 (q, *J*=7.1, 4H), 1.02 (t, *J*=7.1, 6H).

^{13}C NMR (75 MHz, CD_2Cl_2) δ: = 136.45, 132.96, 130.61, 129.81, 128.97, 127.85, 55.80, 47.17, 30.13, 12.08.

GC-MS: *m/z* (%): 225.13 ($[M+2H]^+$, 7), 224.13 ($[M+H]^+$, 6), 223.13 (M^+, 20), 222.13 ($[M-H]^+$, 9), 210.10 (5), 208.10 (16), 194.08 (5), 153.03 (34), 152.04 (11), 151.04 (100), 125.01 (6), 116.06 (32), 115.06 (43), 98.10 (40), 86.10 (10), 56.07 (7).

(*E*)-*N,N*-diethyl-3-(4-(trifluoromethyl)phenyl)prop-2-en-1-amine (25a)

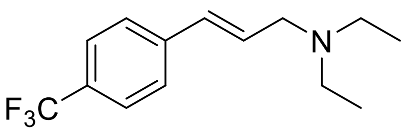

Prepared according to **Method B.** The ethyl *E*)-3-(4-(trifluoromethyl)phenyl)acrylate was prepared from the corresponding carboxylic acid in ethanol with quantitative yield. Then, the *α,β*-unsaturated ester (3.0 g, 12.29 mmol) was reduced with DIBAL-H (30.71 mmol, 2.5 equiv.) as described in **Method B**, yielding (*E*)-3-(4-(trifluoromethyl)phenyl)prop-2-en-1-ol (2.06 g, 10.20 mmol, 83% yield). Finally, **25a** was obtained from the reaction of: (E)-3-(4-(trifluoromethyl)phenyl)prop-2-en-1-ol (1.90 g, 9.40 mmol), triphenylphosphine (2.711 g, 10.34 mmol), *N*-bromosuccinimide (1.84 g, 10.34 mmol) and diethylamine (1.44 g, 29.74 mmol).

Chemical Formula: $C_{14}H_{18}F_3N$

Molecular Weight: 257.3002

Appearance: colorless oil

Isolated Yield: 71% (1.717 g, 6.67 mmol)

^{1}H NMR (300 MHz, CD_2Cl_2): δ = 7.61 - 7.45 (m, 4H), 6.57 (d, *J*=16.0, 1H), 6.41 (dt, *J*=15.9, 6.3, 1H), 3.24 (dd, *J*=6.3, 1.2, 2H), 2.54 (q, *J*=7.1, 4H), 1.03 (t, *J*=7.1, 6H).

^{13}C NMR (75 MHz, CD_2Cl_2): δ = 141.49, 132.14, 130.45, 129.26, 128.83, 126.78, 126.62, 125.80 (q, *J*=3.9), 123.02, 55.79, 47.25, 30.12, 12.09.

^{19}F NMR (282 MHz, CD_2Cl_2): δ = -62.74.

GC-MS: *m/z* (%): 258.19 ($[M+H]^+$,3), 257.19 (M^+, 21), 256.19 ($[M-H]^+$,8), 243.16 (5), 242.16 (33), 238.19 (4), 228.14 (3), 186.09 (14), 185.11 (100), 183.08 (4), 166.08 (4), 165.08 (29), 164.09 (6), 159.06 (4), 145.06 (5), 116.07 (8), 115.07 (10), 98.11 (17), 86.13 (7), 56.09 (4).

HRMS (ESI): *m/z* calcd. for $C_{14}H_{18}F_3N$: 258.1464 $[M+H]^+$, found: 258.1469.

(*E*)-*N,N*-diethyl-3-(4-methoxyphenyl)prop-2-en-1-amine (26a)

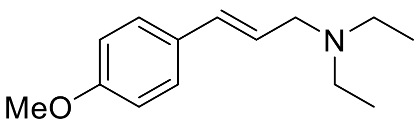

Prepared according to **Method B.** The ethyl (*E*)-3-(4-methoxyphenyl)acrylate was prepared from the corresponding carboxylic acid in ethanol. Then, the *α,β*-unsaturated ester (3.0 g, 14.24 mmol) was reduced with DIBAL-H (35.60 mmol, 2.5 equiv.) as described in **Method B**, yielding (*E*)-3-(4-methoxyphenyl)prop-2-en-1-ol (1.924 g, 10.11 mmol, 71% yield). Finally, **26a** was obtained from the reaction of: (*E*)-3-(4-methoxyphenyl)prop-2-en-1-ol (1.90 g, 11.27 mmol), triphenylphosphine (3.25 g, 12.40 mmol), *N*-bromosuccinimide (2.21 g, 12.40 mmol) and diethylamine (1.731 g, 23.66 mmol).

Chemical Formula: $C_{14}H_{21}NO$

Molecular Weight: 219.3280

Appearance: colorless oil

Isolated Yield: 82% (2.023 g, 9.22 mmol)

^{1}H NMR (300 MHz, CD_2Cl_2): δ = 7.36 - 7.22 (m, 2H), 6.91 - 6.78 (m, 2H), 6.44 (d, *J*=15.9, 1H), 6.13 (dt, *J*=15.9, 6.7, 1H), 3.78 (s, 3H), 3.19 (dd, *J*=6.7, 1.4, 2H), 2.53 (q, *J*=7.1, 4H), 1.02 (t, *J*=7.1, 6H).

^{13}C NMR (75 MHz, CD_2Cl_2): δ = 159.45, 131.46, 130.55, 127.67, 126.37, 114.26, 55.90, 55.62, 54.20, 53.84, 53.48, 47.02, 30.10, 12.03.

GC-MS: *m/z* (%): 220.20 ($[M+H]^+$, 5), 219.21 (M^+, 34), 218.20 ($[M-H]^+$, 14), 204.16 (5), 190.14 (19), 148.10 (14), 147.11 (100), 135.08 (12), 132.06 (7), 131.07 (7), 121.07 (7), 117.07 (8), 116.07 (5), 115.07 (14), 104.07 (5), 103.06 (7), 98.12 (53), 91.07 (16), 86.12 (8), 56.09 (6).

(*E*)-3-(3-bromo-5-methylphenyl)-*N,N*-diethylprop-2-en-1-amine (27a)

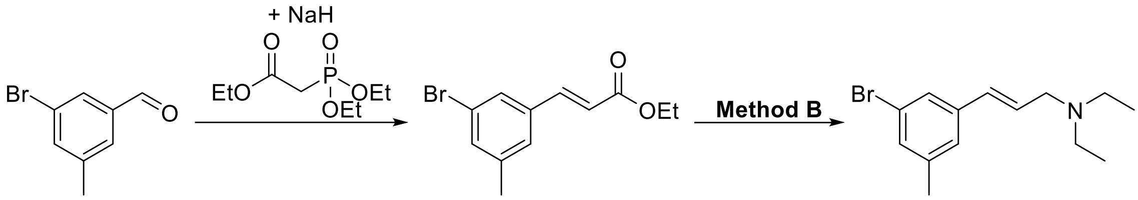

A 250 mL Schlenk flask was charged with ethyl 2-(diethoxyphosphoryl)acetate (3.40 g, 15.17 mmol, 1.08 equiv.) and 40 mL anhydrous Et_2O were added. After the solution was cooled down to 0°C, a 1 M NaH-Et_2O-solution (14.04 mmol, 1 equiv.) was slowly injected under stirring. Then, the solution stirred for 30 min and 3-bromo-5-methylbenzaldehyde (2.80 g, 14.04 mmol, 1 equiv.) was dropwise added. The solution was allowed to stir overnight. Next, the reaction mixture was quenched with 20 mL H_2O. Then, the product was extracted with EtOAc, and the combined organic fractions were dried over magnesium sulfate. After the solvent was removed *in vacuo* a yellow oil was obtained. Finally, ethyl (*E*)-3-(3-bromo-5-methylphenyl)acrylate was isolated by column chromatography (pentane/Et_2O) as a colorless oil (2.96 g, 11.00 mmol, 78% yield). As the next step, the *α,β*-unsaturated ester (1.9 g, 7.06 mmol) was reduced with DIBAL-H (17.65 mmol, 2.5 equiv.) as described in **Method B**, yielding (*E*)-3-(3-bromo-5-methylphenyl)prop-2-en-1-ol (1.170 g, 5.15 mmol, 73% yield). Finally, **27a** was obtained from the reaction of: (*E*)-3-(3-bromo-5-methylphenyl)prop-2-en-1-ol (1.10 g, 4.84 mmol), triphenylphosphine (1.397 g, 5.328 mmol), *N*-bromosuccinimide (0.948 g, 5.33 mmol) and diethylamine (0.744 g, 10.17 mmol).

Chemical Formula: $C_{14}H_{20}BrN$

Molecular Weight: 282.2250

Appearance: yellow oil

Isolated Yield: 65% (888.5 mg, 3.15 mmol)

^{1}H NMR (300 MHz, CD_2Cl_2): δ = 7.33 (ddd, *J*=2.5, 1.2, 0.6, 1H), 7.19 (tq, *J*=1.4, 0.7, 1H), 7.12 (tq, *J*=1.4, 0.7, 1H), 6.42 (ddt, *J*=15.9, 1.3, 0.7, 1H), 6.27 (dt, *J*=15.9, 6.3, 1H), 3.21 (dd, *J*=6.3, 1.3, 2H), 2.53 (q, *J*=7.1, 4H), 2.31 (q, *J*=0.7, 3H), 1.02 (t, *J*=7.1, 6H).

^{13}C NMR (75 MHz, CD_2Cl_2) δ: = 140.79, 139.79, 130.96, 130.50, 126.48, 126.17, 122.70, 55.70, 47.21, 21.23, 12.16.

GC-MS: *m/z* (%): 283.17 ([M+H]$^+$, 18), 282.17 (M$^+$, 9), 281.17 ([M-H]$^+$, 18), 280.17 (6), 268.15 (11), 266.15 (12), 254.12 (16), 252.12 (17), 211.08 (29), 210.10 (5), 209.08 (30), 131.13 (15), 130.14 (100), 129.14 (22), 128.12 (12), 115.10 (21), 98.14 (35), 86.14 (11).

HRMS (ESI): *m/z* calcd. for $C_{14}H_{20}BrN$: 282.0852 [M+H]$^+$, found: 282.0857.

(*E*)-*N,N*-diethyl-4-phenylbut-3-en-1-amine (28a)

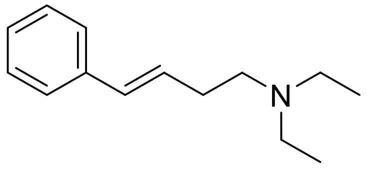

Prepared according to **Method B.** First, ethyl (*E*)-4-phenylbut-3-enoate was prepared from the corresponding carboxylic acid in ethanol. Then, the *α,β*-unsaturated ester (3.0 g, 15.77 mmol) was reduced with DIBAL-H (39.42 mmol, 2.5 equiv.) as described in **Method B**, yielding (*E*)-4-phenylbut-3-en-1-ol (2.10 g, 14.2 mmol, 90% yield). Finally, **28a** was obtained from the reaction of: (*E*)-4-phenylbut-3-en-1-ol (2.0 g, 13.5 mmol), triphenylphosphine (3.89 g, 14.84 mmol), *N*-bromosuccinimide (2.64 g, 14.84 mmol) and diethylamine (2.07 g, 28.34 mmol).

Chemical Formula: $C_{14}H_{21}N$

Molecular Weight: 203.3290

Appearance: colorless oil

Isolated Yield: 70% (1.92 g, 9.45 mmol)

^{1}H NMR (300 MHz, CD_2Cl_2): δ = 7.37 - 7.28 (m, 2H), 7.27 - 7.18 (m, 2H), 7.17 - 7.08 (m, 1H), 5.79 (tq, *J* = 6.6, 1.3 Hz, 1H), 3.15 (dq, *J* = 6.6, 1.0 Hz, 2H), 2.45 (q, *J* = 7.1 Hz, 4H), 1.97 (dt, *J* = 1.4, 0.9 Hz, 3H), 0.95 (t, *J* = 7.1 Hz, 6H).

^{13}C NMR (75 MHz, CD_2Cl_2): δ = 144.12, 136.59, 128.53, 127.08, 126.85, 126.00, 51.79, 47.44, 16.21, 12.31.

GC-MS: *m/z* (%): 204.16 ([M+H]$^+$, 4), 203.16 (M$^+$, 23), 202.15 ([M-H]$^+$, 14), 189.14 (6), 188.14 (43), 174.11 (69), 132.09 (14), 131.11 (100), 129.08 (15), 128.08 (8),117.07 (4), 116.07 (13), 115.06 (17), 105.07 (4), 98.11 (34), 91.07 (32), 86.12 (15), 77.06 (5).

(*E*)-*N,N*-diethyl-3-phenylbut-2-en-1-amine (29a)

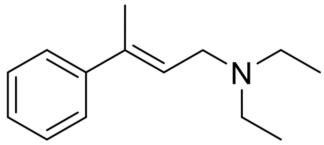

Known compound.[20] Prepared according to **Method B.** First, the *α,β*-unsaturated ester (2.50 g, 13.14 mmol) was reduced with DIBAL-H (32.9 mmol, 2.5 equiv.) as described in **Method B**, yielding (*E*)-3-phenylbut-2-en-1-ol as colorless oil (1.636 g, 11.04 mmol, 84% yield). Finally, **29a** was obtained from the reaction of: (*E*)-3-cyclohexylprop-2-en-1-ol (1.60 g, 10.80 mmol), triphenylphosphine (3.115 g, 11.88 mmol), *N*-bromosuccinimide (2.114 g, 11.88 mmol) and diethylamine (1.658 g, 22.67 mmol).

Chemical Formula: $C_{14}H_{21}N$

Molecular Weight: 203.3290

Appearance: colorless oil

Isolated Yield: 70% (1.537 g, 7.56 mmol)

^{1}H NMR (300 MHz, CD_2Cl_2): δ = 7.37 - 7.28 (m, 2H), 7.27 - 7.18 (m, 2H), 7.17 - 7.08 (m, 1H), 5.79 (tq, *J* = 6.6, 1.3 Hz, 1H), 3.15 (dq, *J* = 6.6, 1.0 Hz, 2H), 2.45 (q, *J* = 7.1 Hz, 4H), 1.97 (dt, *J* = 1.4, 0.9 Hz, 3H), 0.95 (t, *J* = 7.1 Hz, 6H).

^{13}C NMR (75 MHz, CD_2Cl_2): δ = 144.12, 136.59, 128.53, 127.08, 126.85, 126.00, 51.79, 47.44, 16.21, 12.31.

GC-MS: *m/z* (%): 204.16 ([M+H]$^+$, 4), 203.16 (M$^+$, 23), 202.15 ([M-H]$^+$, 14), 189.14 (6), 188.14 (43), 174.11 (69), 132.09 (14), 131.11 (100), 129.08 (15), 128.08 (8),117.07 (4), 116.07 (13), 115.06 (17), 105.07 (4), 98.11 (34), 91.07 (32), 86.12 (15), 77.06 (5).

(*Z*)-*N,N*-diethyl-3-(4-fluorophenyl)-3-phenylprop-2-en-1-amine (30a)

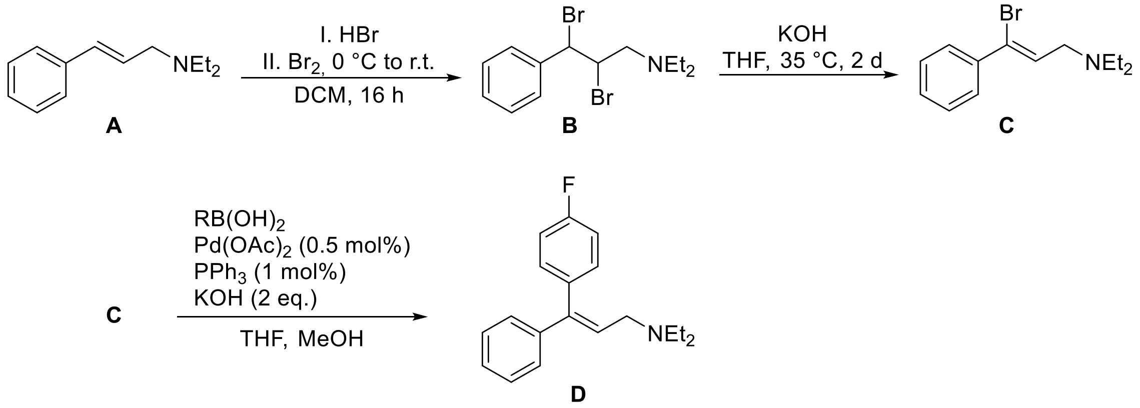

(*E*)-*N,N*-diethyl-3-phenylprop-2-en-1-amine (**A**, 14.00 g, 73.96 mmol, 1 equiv.) was added to a 250 mL round-bottom flask, and 1 M HBr (73.96 mmol, 1 equiv.) was added dropwise at 0 °C. After stirring for 10 minutes, the solvent was removed *in vacuo*. The obtained red oil was dissolved in 70 mL DCM, dried with sodium sulfate and filtrated. Afterwards, bromine (14.18 g, 88.75 mmol, 1.2 equiv.) was added dropwise at 0 °C over 30 minutes. The reaction mixture was allowed to warm up slowly and stir overnight. The remaining bromine was quenched by the addition of aqueous sodium bisulfite. Next, the organic layer was separated, dried with sodium sulfate and the solvent was removed *in vacuo*. Finally, 2,3-dibromo-*N,N*-diethyl-3-phenylpropan-1-amine (**B**) was obtained as orange oil and was used without purification.

Then, a 1 L round-bottom flask was charged with **B** (24.19 g, 69.96 mmol, 1 equiv.), potassium hydroxide (7.85 g, 0.140 mol, 2 equiv.) and 350 mL THF. The reaction mixture was heated for 3 d at 40 °C. The received orange solution was filtrated, and the solvent was removed *in vacuo*. The, desired product 3-bromo-*N,N*-diethyl-3-phenylprop-2-en-1-amine (**C**) was obtained as a red oil after two rounds of flash column chromatography (5.254 g, 19.59 mmol, 28 % yield).

Finally, **D** was obtained by Suzuki coupling of (4-fluorophenyl)boronic acid and **C**. Under argon atmosphere, a 100 mL Schlenk flask was charged with **C** (1.400 g, 5.22 mmol), $Pd(OAc)_2$ (5.9 mg, 0.026 mmol, 0.5 mol%), PPh_3 (13.7 mg, 0.052 mmol, 1 mol%), (4-fluorophenyl)boronic acid (1.46 g, 10.44 mmol, 2 equiv.) and KOH (585.8 mg, 10.44 mmol, 2 equiv.). Next, 25 mL anhydrous MeOH and 25 mL anhydrous THF were added, and the reaction mixture was stirred overnight at room temperature. Then, 40 mL diethyl ether was added, and the organic layer was washed with 1 M NaOH and brine. Afterwards, the product solution was dried with sodium sulfate, and the solvent was removed *in vacuo*. The received yellow oil was purified by column chromatography, yielding **D** as a slightly yellow oil (1.01 g, 3.57 mmol, 68% yield).

Chemical Formula: $C_{19}H_{22}FN$

Molecular Weight: 283.3904

Appearance: yellow oil

^{1}H NMR (300 MHz, CD_2Cl_2): δ = 7.43 - 7.27 (m, 3H), 7.26 - 7.11 (m, 4H), 7.03 - 6.89 (m, 2H), 6.14 (t, *J* = 6.7 Hz, 1H), 3.11 (d, *J* = 6.7 Hz, 2H), 2.48 (q, *J* = 7.1 Hz, 4H), 0.93 (t, *J* = 7.1 Hz, 6H).

^{13}C NMR (75 MHz, CD_2Cl_2): δ = 162.50 (d, *J* = 245.5 Hz), 142.33, 140.15, 139.24 (d, *J* = 3.2 Hz), 130.20, 129.28 (d, *J* = 7.9 Hz), 128.57, 128.39 (d, *J* = 1.6 Hz), 127.58, 115.18 (d, *J* = 21.4 Hz), 52.15, 47.41, 12.22.

^{19}F NMR (282 MHz, CD_2Cl_2): δ = -116.62.

GC-MS: *m/z* (%): 283.16 (M^+, 38), 282.15 ($[M-H]^+$, 34), 254.12 (27), 212.09 (18), 211.08 (100), 210.11 (9), 209.07 (17), 207.09 (10), 206.12 (17), 196.05 (31), 192.10 (10), 188.12 (10), 183.04 (17), 174.10 (10), 133.02 (37), 115.04 (30), 109.02 (13), 98.09 (58), 91.05 (13), 86.09 (13), 56.06 (8).

(2*E*,2'*E*)-3,3'-(1,4-phenylene)bis(*N*,*N*-diethylprop-2-en-1-amine) (31a)

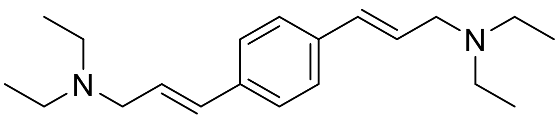

Prepared according to **Method B.** Diethyl 3,3'-(1,4-phenylene)(2*E*,2'*E*)-diacrylate was prepared from the corresponding carboxylic acid in ethanol with quantitative yield. Then, the *α,β*-unsaturated ester (3.0 g, 10.94 mmol) was reduced with DIBAL-H (54.68 mmol, 5.0 equiv.) as described in **Method B**, yielding (2E,2'E)-3,3'-(1,4-phenylene)bis(prop-2-en-1-ol) (1.560 g, 8.20 mmol, 75% yield). Finally, allylamine **31a** was obtained from the reaction of: (2*E*,2'*E*)-3,3'-(1,4-phenylene)bis(prop-2-en-1-ol) (1.56 g, 8.20 mmol), triphenylphosphine (2.366 g, 9.02 mmol), *N*-bromosuccinimide (1.606 g, 9.02 mmol) and diethylamine (1.259 g, 17.22 mmol).

Chemical Formula: $C_{20}H_{32}N_2$

Molecular Weight: 300.4900

Appearance: colorless oil

Isolated Yield: 45% (1.109 g, 3.69 mmol)

^{1}H NMR (300 MHz, CD_2Cl_2): δ = 7.32 (s, 4H), 6.49 (dt, *J*=15.9, 1.4, 2H), 6.26 (dt, *J*=15.9, 6.5, 2H), 3.21 (dd, *J*=6.5, 1.4, 4H), 2.53 (q, *J*=7.1, 8H), 1.02 (t, *J*=7.1, 12H).

^{13}C NMR (75 MHz, CD_2Cl_2): δ = 136.75, 131.58, 128.62, 126.72, 55.94, 47.15, 12.12.

GC-MS: *m/z* (%): 301.35 ($[M+H]^+$, 8), 300.35 (M^+, 33), 299.35 ($[M-H]^+$, 10), 271.30 (23), 229.27 (27), 228.27 (49), 227.26 (12), 156.14 (8), 155.14 (23), 135.17 (8), 129.11 (28), 128.12 (11), 115.09 (13), 112.15 (21), 106.63 (18), 99.15 (8), 98.15 (100), 86.14 (12).

11 Isomerization of Allylamine Derivatives

11.1 General Procedure: Isomerization of Allylamine Derivatives

Under argon atmosphere, a 5 mL Schlenk pressure tube was charged with **cobalt(II) acetylacetonate** (1 equiv.), **L15** (2 equiv.) and a stirring bar. Then, 2-MeTHF respectively THF was injected (0.8 - 1.5 mL, see section 11.2). Afterwards, **DIBAL-H** (2 equiv.) was dropwise added to the precatalyst mixture as a THF-solution (0.2 mL). After 5 min of stirring, the selected allylamine derivative was added to the catalyst solution. The Schlenk pressure tube was sealed and heated for 24 h. Then, the reaction mixture was cooled down to room temperature. The conversion of the substrate was determined with GC analysis using *n*-hexadecane as internal standard or by ^{1}H NMR. The solvent was removed *in vacuo*, followed by a vacuum distillation of the crude oil. The distilled enamine was then hydrolyzed with 3 mL of a 5% acetic acid solution. After 30 min of stirring, the aldehyde was extracted with diethyl ether followed by washing steps with a sodium carbonate solution and distilled water. The organic solution was dried with sodium sulfate and the solvent was removed *in vacuo*. Finally, the received product was purified by column chromatography or vacuum distillation (see section 11.2).

11.2 Isolated Products

(*E*)-*N,N*-diethyl-3,7-dimethylocta-1,6-dien-1-amine ((*E*)-1b)

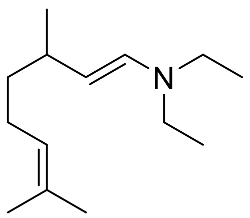

Known Compound.[1] Prepared by following the general experimental procedure with 1.3 mg **Co(acac)₂** (5 µmol), 3.8 mg **L15** (10 µmol), 10 µmol **DIBAL-H** and **1a** (1.047 g, 5 mmol) in 1.5 mL 2-MeTHF at 100 °C. The citronellal-enamine **(*E*)-1b** was isolated by vacuum distillation.

Chemical Formula: $C_{14}H_{27}N$

Molecular Weight: 209.3770

Appearance: colorless oil

Isolated Yield: 99 % (1.033 g, 4.93 mmol)

^{1}H NMR (300 MHz, $CDCl_3$): δ = 5.80 (dd, *J* = 13.9, 0.8 Hz, 1H), 5.11 (tdt, *J* = 7.2, 2.8, 1.4 Hz, 1H), 4.04 (dd, *J* = 13.9, 8.1 Hz, 1H), 2.93 (q, *J* = 7.1 Hz, 4H), 2.10 - 1.84 (m, 3H), 1.73 - 1.53 (m, 5H), 1.37 - 1.15 (m, 2H), 1.04 (t, *J* = 7.1 Hz, 6H), 0.97 (d, *J* = 6.7 Hz, 3H).

^{13}C NMR (75 MHz, $CDCl_3$): δ = 135.95, 130.92, 125.42, 105.26, 44.57, 39.08, 35.21, 26.26, 25.90, 22.89, 17.82, 12.31.

3,7-Dimethyloct-6-enal ((*E*)-1c)

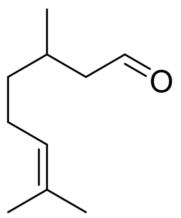

Known Compound.[1] After hydrolysis of citronellal-enamine (**1b**) and extraction (section 11.1), product **(*E*)-1c** was purified by column chromatography.

Chemical Formula: $C_{10}H_{18}O$

Molecular Weight: 154.2530

Appearance: colorless oil

Isolated Yield: 98% (752.0 mg, 4.88 mmol)

^{1}H NMR (300 MHz, $CDCl_3$): δ = 9.75 (dd, *J* = 2.6, 2.0 Hz, 1H), 5.08 (ddt, *J* = 8.5, 5.6, 1.4 Hz, 1H), 2.40 (ddd, *J* = 16.0, 5.6, 2.1 Hz, 1H), 2.22 (ddd, *J* = 15.9, 7.9, 2.6 Hz, 1H), 2.13 -

1.93 (m, 3H), 1.64 (dd, *J* = 24.8, 1.1 Hz, 6H), 1.44 - 1.18 (m, 2H), 0.97 (d, *J* = 6.6 Hz, 3H).

^{13}C NMR (75 MHz, $CDCl_3$): δ = 203.18, 131.91, 124.17, 51.14, 37.08, 27.91, 25.83, 25.53, 20.00, 17.79.

GC-MS: *m/z* (%): 154.11 (M^+, 15), 139.08 (14), 136.09 (15), 121.07 (44), 112.06 (15), 111.05 (30), 110.08 (22), 109.07 (22), 97.04 (15), 95.06 (77), 84.04 (20), 83.04 (18), 81.05 (18), 70.06 (16), 69.05 (100), 67.04 (32), 56.05 (25), 55.04 (44), 53.03 (15).

(*E*)-3,7,11-trimethyldodeca-6,10-dienal (10c)

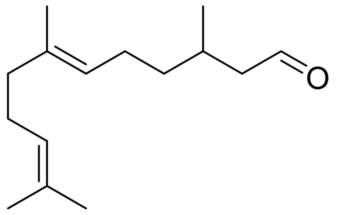

Known Compound.[26] Prepared by following the general experimental procedure with 15.4 mg **Co(acac)₂** (60 µmol), 45.9 mg **L15** (120 µmol), 120 µmol **DIBAL-H** and **10a** (416.3 mg, 1.5 mmol) in 1.5 mL 2-MeTHF at 100 °C (reaction time: 48 h). After hydrolysis of the enamine and extraction, **10c** was purified by column chromatography.

Chemical Formula: $C_{15}H_{26}O$

Molecular Weight: 222.3720

Appearance: colorless oil

Isolated Yield: 79% (262.5 mg, 1.18 mmol)

^{1}H NMR (300 MHz, $CDCl_3$): δ = 9.75 (dd, *J* = 2.6, 2.1 Hz, 1H), 5.09 (tdt, *J* = 6.8, 3.6, 1.7 Hz, 2H), 2.41 (ddd, *J* = 15.9, 5.6, 2.1 Hz, 1H), 2.23 (ddd, *J* = 15.9, 7.8, 2.6 Hz, 1H), 2.14 - 1.91 (m, 7H), 1.68 (q, *J* = 1.3 Hz, 3H), 1.60 (dd, *J* = 1.5, 0.8 Hz, 6H), 1.45 - 1.20 (m, 2H), 0.97 (d, *J* = 6.6 Hz, 3H).

^{13}C NMR (75 MHz, $CDCl_3$): δ = 203.17, 135.55, 131.51, 124.44, 124.07, 51.15, 39.85, 37.06, 27.94, 26.82, 25.84, 25.43, 20.04, 17.83, 16.13.

GC-MS: *m/z* (%): 222.19 (M^+, 1), 204.17 (2), 180.12 (4), 179.12 (26), 161.10 (12), 123.09 (25), 121.07 (6), 109.07 (29), 108.07 (5), 107.06 (10), 97.04 (5), 95.06 (10), 93.05 (13), 83.06 (6), 81.06 (16), 79.05 (6), 70.07 (7), 69.07 (100), 68.07 (6), 67.05 (18), 55.05 (12).

Hexanal (11c)

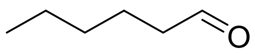

Known Compound.[27] Prepared by following the general experimental procedure with 3.9 mg **Co(acac)₂** (15 µmol), 11.5 mg **L15** (30 µmol), 30 µmol **DIBAL-H** and **11a** (236.0 mg, 1.5 mmol) in 1 mL 2-MeTHF at 100 °C. After hydrolysis of the enamine and extraction, **11c** was purified by distillation.

Chemical Formula: $C_6H_{12}O$

Molecular Weight: 100.1610

Appearance: colorless oil

Isolated Yield: 95% (142.7 mg, 1.43 mmol)

^{1}H NMR (300 MHz, $CDCl_3$): δ = 9.76 (t, *J* = 1.9 Hz, 1H), 2.41 (td, *J* = 7.4, 1.9 Hz, 2H), 1.67- 1.59 (m, 2H), 1.35 - 1.27 (m, 4H), 0.93 - 0.86 (m, 3H).

^{13}C NMR (75 MHz, $CDCl_3$): δ = 203.10, 44.01, 31.44, 22.53, 21.89, 13.99.

GC-MS: *m/z* (%): 100.04 (M^+, 1), 85.09 (2), 83.08 (3), 82.08 (28), 81.07 (3), 73.06 (2), 72.05 (33), 71.06 (14), 70.08 (2), 69.06 (2), 67.05 (17), 58.05 (12), 57.05 (70), 56.07 (100), 55.07 (23), 54.07 (4), 53.05 (5).

3-Cyclohexylpropanal (12c)

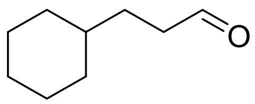

Known Compound.[28] Prepared by following the general experimental procedure with 3.9 mg **Co(acac)₂** (15 µmol), 11.5 mg **L15** (30 µmol), 30 µmol **DIBAL-H** and **12a** (296.1 mg, 1.5 mmol) in 1 mL 2-MeTHF at 100 °C. After hydrolysis of the enamine and extraction, **12c** was purified by vacuum distillation.

Chemical Formula: $C_9H_{16}O$

Molecular Weight: 140.2260

Appearance: colorless oil

Isolated Yield: 90% (189.3 mg, 1.35 mmol)

^{1}H NMR (300 MHz, $CDCl_3$): δ = 9.76 (t, *J* = 1.9 Hz, 1H), 2.48 - 2.36 (m, 2H), 1.69 (ddd, *J* = 11.9, 2.9, 1.2 Hz, 5H), 1.52 (dt, *J* = 8.5, 7.1 Hz, 2H), 1.33 - 1.09 (m, 4H), 0.99 - 0.80 (m, 2H).

^{13}C NMR (75 MHz, $CDCl_3$): δ = 203.26, 41.69, 37.34, 33.19, 29.52, 26.62, 26.34.

GC-MS: *m/z* (%): 140.12 (M^+, 3), 123.11 (3), 122.10 (30), 107.08 (7), 97.10 (18), 96.11 (76), 95.10 (10), 94.09 (30), 93.08 (12), 83.09 (26), 82.09 (12), 81.10 (100), 80.08 (17), 79.08 (13), 68.08 (23), 67.08 (48), 55.09 (59), 54.08 (15).

(*E*)-*N,N*-diethyl-4,4,4-trifluorobut-1-en-1-amine (13b)

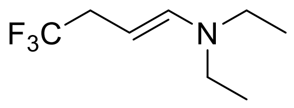

Prepared by following the general experimental procedure with 2.6 mg **Co(acac)$_2$** (10 µmol), 7.6 mg **L15** (20 µmol), 20 µmol **DIBAL-H** and **13a** (181.2 mg, 1 mmol) in 1 mL THF at 80 °C. The enamine (**13b**) was isolated by vacuum distillation.

Chemical Formula: $C_8H_{14}F_3N$

Molecular Weight: 181.2022

Appearance: colorless oil

Isolated Yield: 91% (164.1 mg, 0.91 mmol)

^{1}H NMR (300 MHz, $CDCl_3$): δ = 6.07 - 5.95 (m, 1H), 3.94 (dt, *J* = 13.7, 7.4 Hz, 1H), 3.00 (q, *J* = 7.1 Hz, 4H), 2.67 (qdd, *J* = 10.8, 7.4, 1.0 Hz, 2H), 1.06 (t, *J* = 7.1 Hz, 6H).

^{13}C NMR (75 MHz, $CDCl_3$): δ = 142.11, 126.86 (d, *J* = 276.5), 82.12 (q, *J* = 3.8), 68.13, 44.83, 35.84 (q, *J* = 29.7), 25.77, 12.74.

^{19}F NMR (282 MHz, $CDCl_3$): δ = -68.35.

GC-MS: *m/z* (%): 182.14 ($[M+H]^+$, 10), 181.16 (M^+, 82), 180.16 ($[M-H]^+$, 5), 167.12 (12), 166.15 (100), 162.14 (6), 152.10 (25), 138.07 (26), 112.13 (36), 98.09 (29), 82.09 (6), 68.07 (9), 56.08 (31).

2-methylbutanal (15c)

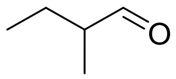

Known Compound.[29] Prepared by following the general experimental procedure with 5.1 mg **Co(acac)$_2$** (20 µmol), 15.3 mg **L15** (40 µmol), 40 µmol **DIBAL-H** and **15a** (141.3 mg, 1 mmol) in 1 mL THF at 100 °C (reaction time: 48 h). After hydrolysis of the enamine and extraction, **15c** was purified by distillation.

Chemical Formula: $C_5H_{10}O$

Molecular Weight: 86.1340

Appearance: colorless oil

Isolated Yield: 65% (56.0 mg, 0.65 mmol)

^{1}H NMR (300 MHz, CDCl$_3$): δ = 9.62 (d, *J* = 1.9 Hz, 1H), 2.27 (qd, *J* = 6.9, 1.9 Hz, 1H), 1.84 - 1.36 (m, 2H), 1.09 (d, *J* = 7.0 Hz, 3H), 0.99 - 0.90 (m, 3H).

^{13}C NMR (75 MHz, CDCl$_3$): δ = 205.51, 47.90, 23.67, 13.00, 11.48.

***N,N*-diethylcyclohex-1-en-1-amine (16b)**

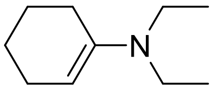

Known Compound.[1] Prepared by following the general experimental procedure with 2.6 mg **Co(acac)$_2$** (10 µmol), 7.6 mg **L15** (20 µmol), 20 µmol **DIBAL-H** and **16a** (153.3 mg, 1 mmol) in 1 mL THF at 80 °C. The enamine (**16b**) was isolated by vacuum distillation.

Chemical Formula: $C_{10}H_{19}N$

Molecular Weight: 153.2690

Appearance: colorless oil

Isolated Yield: 94% (144.1 mg, 0.94 mmol)

^{1}H NMR (400 MHz, CDCl$_3$): δ = 4.52 (td, *J* = 3.3, 1.1 Hz, 1H), 2.97 (q, *J* = 7.1 Hz, 4H), 2.14 - 2.03 (m, 4H), 1.73 - 1.61 (m, 2H), 1.60 - 1.48 (m, 2H), 0.99 (t, *J* = 7.0 Hz, 6H).

^{13}C NMR (101 MHz, CDCl$_3$): δ = 143.17, 99.23, 42.49, 27.70, 25.25, 24.05, 23.44, 12.35.

3-Methylbutanal (17c)

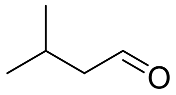

Known Compound.[30] Prepared by following the general experimental procedure with 2.5 µmol **Co(acac)₂**, 5 µmol **L15**, 5 µmol **DIBAL-H** and **17a** (353.1 mg, 2.5 mmol) in 1 mL THF at 100 °C. After hydrolysis of the enamine and extraction, **17c** was purified by distillation.

Chemical Formula: $C_5H_{10}O$

Molecular Weight: 86.1340

Appearance: colorless oil

Isolated Yield: 92% (198.1 mg, 2.3 mmol)

^{1}H NMR (300 MHz, CDCl$_3$): δ = 9.75 (t, *J* = 2.3 Hz, 1H), 2.35 - 2.09 (m, 4H), 0.98 (d, *J* = 6.6 Hz, 9H).

^{13}C NMR (75 MHz, CDCl$_3$): δ = 203.03, 52.74, 23.63, 22.72, 22.36.

3-Methylheptanal (18c)

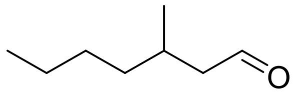

Known Compound.[31] Prepared by following the general experimental procedure with 2.6 mg **Co(acac)₂** (10 µmol), 7.6 mg **L15** (20 µmol), 20 µmol **DIBAL-H** and **18a** (183.3 mg, 1.0 mmol) in 1 mL 2-MeTHF at 100 °C. After hydrolysis of the enamine and extraction, **18c** was purified by vacuum distillation.

Chemical Formula: $C_8H_{16}O$

Molecular Weight: 128.2150

Appearance: colorless oil

Yield: 97% (124.4 mg, 0.97 mmol)

^{1}H NMR (300 MHz, CDCl$_3$): δ = 9.75 (t, 1H), 2.47 - 2.15 (m, 2H), 2.10 - 1.92 (m, 1H), 1.40 - 1.17 (m, 6H), 0.95 (d, *J* = 6.7 Hz, 3H), 0.92 - 0.84 (m, 3H).

^{13}C NMR (75 MHz, CDCl$_3$): δ = 203.30, 51.24, 36.74, 29.28, 28.30, 22.90, 20.13, 14.18.

GC-MS: *m/z* (%): 127.06 ([M-H]$^{+}$, 4),126.07 ([M-2H]$^{2+}$, 31), 112.10 (10), 111.09 (100), 97.08 (7), 90.99 (3), 84.01 (8), 83.05 (6), 78.01 (6), 70.05 (20), 69.04 (69), 61.00 (6), 55.97 (16), 55.04 (36).

Propionaldehyde (19c)

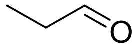

Known Compound. Prepared by following the general experimental procedure with 2.5 µmol **Co(acac)₂**, 5 µmol **L15**, 5 µmol **DIBAL-H** and **19a** (283.0 mg, 2.5 mmol) in 1 mL THF at 80 °C. After hydrolysis of the enamine and extraction, **19c** was purified by distillation.

Chemical Formula: C_3H_6O

Molecular Weight: 58.0800

Appearance: colorless oil

GC-Yield: 98% (142.3 mg, 2.45 mmol)

Butyraldehyde (20c)

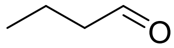

Known Compound. Prepared by following the general experimental procedure with 15 µmol **Co(acac)₂**, 30 µmol **L15**, 30 µmol **DIBAL-H** and **20a** (190.9 mg, 1.5 mmol) in 1 mL THF at 80 °C. After hydrolysis of the enamine and extraction, **20c** was purified by distillation.

Chemical Formula: C_4H_8O

Molecular Weight: 72.1070

Appearance: colorless oil

GC-Yield: 89% (97.3 mg, 1.35 mmol)

^{1}H NMR (300 MHz, CDCl$_3$): δ = 9.76 (t, *J* = 1.9 Hz, 1H), 2.40 (td, *J* = 7.3, 1.9 Hz, 2H), 1.66 (h, *J* = 7.3 Hz, 2H), 0.96 (t, *J* = 7.4 Hz, 3H).

^{13}C NMR (75 MHz, CDCl$_3$): δ = 202.99, 45.90, 15.76, 13.83.

(*E*)-*N,N*-diethyl-3-phenylprop-1-en-1-amine (22b)[16]

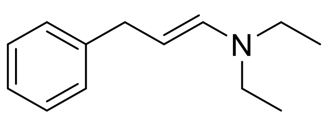

Prepared by following the general experimental procedure with 3.9 mg **Co(acac)$_2$** (15 µmol), 11.5 mg **L15** (30 µmol), 30 µmol **DIBAL-H** and **22a** (284.0 mg, 1.5 mmol) in 1 mL 2-MeTHF at 100 °C. The enamine **22b** was obtained after vacuum distillation of the reaction mixture.

Chemical Formula: $C_{13}H_{19}N$

Molecular Weight: 189.3020

Appearance: colorless oil

Isolated Yield: 97% (275.4 mg, 1.45 mmol)

^{1}H NMR (300 MHz, CD_2Cl_2): δ = 7.33 - 7.09 (m, 4H), 6.03 - 5.91 (m, 1H), 4.27 (dt, *J* = 13.9, 7.1 Hz, 1H), 3.33 - 3.24 (m, 2H), 2.97 (q, *J* = 7.1 Hz, 4H), 1.05 (t, *J* = 7.1 Hz, 6H).

^{13}C NMR (75 MHz, CD_2Cl_2): δ = 144.32, 138.71, 128.56, 128.52, 125.81, 95.98, 54.56, 54.20, 53.84, 53.48, 53.12, 44.93, 37.47, 12.70.

3-phenylpropanal (22c)

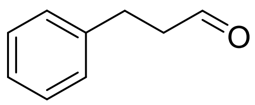

Known Compound.[32] After hydrolysis of **22b** and extraction, **22c** was purified by column chromatography.

Chemical Formula: $C_9H_{10}O$

Molecular Weight: 134.1780

Appearance: colorless oil

Isolated Yield: 90% (181.1 mg, 1.35 mmol)

^{1}H NMR (300 MHz, CD_2Cl_2): δ = 9.79 (t, *J* = 1.4, 1H), 7.36 - 7.14 (m, 5H), 2.94 (t, *J* = 7.5, 2H), 2.82 - 2.70 (m, 2H).

^{13}C NMR (75 MHz, CD_2Cl_2): δ = 201.85, 141.08, 128.89, 128.69, 126.56, 54.20, 53.84, 53.48, 45.63, 28.46.

---

[16]NMR was recorded under inert conditions.

GC-MS: *m/z* (%): 135.08 ([M+H]$^+$, 10), 134.09 (M$^+$, 94), 133.09 ([M+H]$^+$, 16), 116.07 (7), 115.06 (11), 105.08 (41), 104.09 (6), 103.07 (19), 93.08 (6), 92.08 (84), 91.08 (100), 79.08 (21), 78.08 (43), 77.07 (29), 65.07 (15), 63.06 (7), 51.07 (14).

3-(4-(tert-butyl)phenyl)propanal (23c)

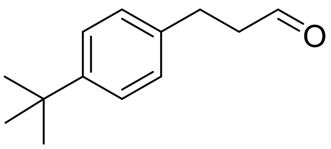

Known Compound.[32] Prepared by following the general experimental procedure with 3.9 mg **Co(acac)$_2$** (15 µmol), 11.5 mg **L15** (30 µmol), 30 µmol **DIBAL-H** and **23a** (368.1 mg, 1.5 mmol) in 1 mL 2-MeTHF at 100 °C. After hydrolysis and extraction, **23c** was purified by vacuum distillation.

Chemical Formula: $C_{13}H_{18}O$

Molecular Weight: 190.2860

Appearance: colorless oil

Isolated Yield: 92% (262.6 mg, 1.38 mmol)

^{1}H NMR (300 MHz, CDCl$_3$): δ = 9.83 (t, *J* = 1.5 Hz, 1H), 7.37 - 7.28 (m, 2H), 7.19 - 7.08 (m, 2H), 3.02 - 2.89 (m, 2H), 2.84 - 2.72 (m, 2H), 2.09 (s, 2H), 1.31 (s, 9H).

^{13}C NMR (75 MHz, CD$_2$Cl$_2$): δ = 201.99, 176.82, 149.33, 137.34, 128.09, 125.64, 45.39, 34.53, 31.51, 27.72, 20.99.

GC-MS: *m/z* (%): 191.08 ([M+H]$^+$, 2), 190.07 (M$^+$, 18), 173.07 (3), 172.06 (23), 147.05 (24), 146.05 (100), 145.11 (6), 144.07 (6), 133.04 (11), 129.05 (11), 128.04 (12), 118.06 (20), 117.06 (89), 115.04 (25), 105.05 (41), 104.05 (37), 103.05 (13), 92.05 (19), 91.05 (45), 78.04 (11), 77.04 (14).

3-(4-chlorophenyl)propanal (24c)

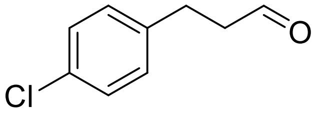

Known Compound.[33] Prepared by following the general experimental procedure with 5.1 mg **Co(acac)$_2$** (20 µmol), 15.3 mg **L15** (40 µmol), 40 µmol **DIBAL-H** and **24a** (223.74 mg, 1 mmol) in 1 mL 2-MeTHF at 100 °C. After hydrolysis of the enamine and extraction, **24c** was purified by vacuum distillation.

Chemical Formula: C_9H_9ClO

Molecular Weight: 168.6200

Appearance: colorless oil

Isolated Yield: 91% (153.4 mg, 0.91 mmol)

^{1}H NMR (300 MHz, CD_2Cl_2): δ = 9.78 (t, *J*=1.3, 1H), 7.31 - 7.22 (m, 2H), 7.20 - 7.11 (m, 2H), 2.94 - 2.86 (m, 2H), 2.81 - 2.70 (m, 2H).

^{13}C NMR (75 MHz, CD_2Cl_2): δ = 201.43, 139.71, 132.17, 130.19, 128.91, 45.42, 27.75.

GC-MS: *m/z* (%): 170.02 ($[M+2H]^+$, 25), 169.03 ($[M+H]^+$, 9), 168.02 (M^+, 74), 167.03 ($[M-H]^+$, 5), 139.01 (11), 133.05 (57), 128.00 (8), 127.00 (35), 126.01 (25), 125.01 (100), 115.04 (13), 114.00 (10), 112.00 (29), 105.06 (13), 103.04 (33), 102.05 (9), 101.02 (7), 91.05 (37), 89.03 (16), 77.04 (26), 75.02 (10), 63.03 (7), 51.04 (9).

3-(4-(trifluoromethyl)phenyl)propanal (25c)

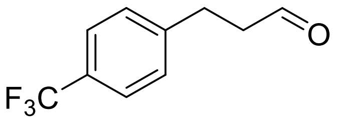

Known Compound.[33] Prepared by following the general experimental procedure with 5.1 mg **Co(acac)₂** (20 µmol), 15.3 mg **L15** (40 µmol), 40 µmol **DIBAL-H** and **25a** (257.3 mg, 1 mmol) in 1 mL 2-MeTHF at 100 °C. After hydrolysis of the enamine and extraction, **25c** was purified by vacuum distillation.

Chemical Formula: $C_{10}H_9F_3O$

Molecular Weight: 202.1762

Appearance: colorless oil

Isolated Yield: 87% (175.9 mg, 0.87 mmol)

^{1}H NMR (300 MHz, $CDCl_3$): δ = 9.83 (t, *J* = 1.2 Hz, 1H), 7.60 - 7.49 (m, 2H), 7.37 - 7.25 (m, 2H), 3.02 (t, *J* = 7.4 Hz, 2H), 2.88 - 2.75 (m, 2H).

^{13}C NMR (75 MHz, $CDCl_3$): δ = 200.74, 144.67, 128.83, 126.16, 125.67 (q, *J* = 3.7 Hz), 122.56, 44.99, 27.96.

^{19}F NMR (282 MHz, $CDCl_3$): δ = -62.45.

GC-MS: *m/z* (%): 203.06 ([M+2H]$^+$, 9), 202.06 (75), 201.11 (6), 184.05 (7), 183.06 (21), 173.04 (12), 161.05 (9), 160.05 (100), 159.05 (58), 153.05 (17), 151.04 (11), 146.03 (23), 145.04 (10), 133.06 (51), 127.04 (18), 119.02 (7),115.05 (9), 109.05 (21), 105.07 (15), 103.06 (10), 91.06 (42), 77.05 (9).

3-(4-methoxyphenyl)propanal (26c)

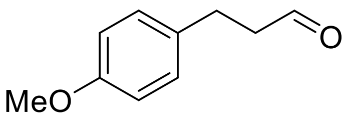

Known Compound.[33] Prepared by following the general experimental procedure with 5.1 mg **Co(acac)$_2$** (20 µmol), 15.3 mg **L15** (40 µmol), 40 µmol **DIBAL-H** and **26a** (219.3 mg, 1 mmol) in 1 mL 2-MeTHF at 100 °C (reaction time: 48 h). After hydrolysis of the enamine and extraction, **26c** was purified by vacuum distillation.

Chemical Formula: $C_{10}H_{12}O_2$

Molecular Weight: 164.2040

Appearance: colorless oil

Isolated Yield: 77% (126.4 mg, 0.77 mmol)

^{1}H NMR (300 MHz, CD_2Cl_2): δ = 9.77 (t, *J* = 1.5, 1H), 7.18 - 7.05 (m, 2H), 6.88 - 6.77 (m, 2H), 3.76 (s, 3H), 2.93 - 2.82 (m, 2H), 2.77 - 2.66 (m, 2H).

^{13}C NMR (75 MHz, CD_2Cl_2): δ = 202.10, 158.53, 132.95, 129.59, 114.23, 55.56, 45.88, 27.59.

GC-MS: *m/z* (%): 165.08 ([M+H]$^+$, 7), 164.09 (M$^+$, 54), 163.19 ([M-H]$^+$, 2), 135.07 (3), 134.07 (2), 122.07 (14), 121.09 (100), 119.06 (3), 109.06 (3), 108.06 (30), 105.07 (4), 103.06 (4), 92.05 (3), 91.06 (14), 89.05 (3), 79.06 (4), 78.06 (9), 77.05 (13), 65.05 (5).

3-(3-bromo-5-methylphenyl)propanal (27c)

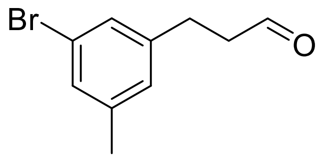

Prepared by following the general experimental procedure with 5.1 mg **Co(acac)₂** (20 µmol), 15.3 mg **L15** (40 µmol), 40 µmol **DIBAL-H** and **27a** (141.1 mg, 0.5 mmol) in 1 mL 2-MeTHF at 100 °C (reaction time: 48 h). After hydrolysis of the enamine and extraction, **27c** was purified by column chromatography (using *n*-pentane/ EtOAc).

Chemical Formula: $C_{10}H_{11}BrO$

Molecular Weight: 227.1010

Appearance: white sticky oil

Isolated Yield: 82% (93.1 mg, 0.41 mmol)

^{1}H NMR (300 MHz, CDCl$_3$): δ = 9.81 (t, *J* = 1.2 Hz, 1H), 7.23 - 7.09 (m, 2H), 6.99 - 6.85 (m, 1H), 2.96 - 2.83 (m, 2H), 2.82 - 2.68 (m, 2H), 2.30 (s, 3H).

^{13}C NMR (75 MHz, CDCl3): δ = 201.09, 142.54, 140.47, 130.19, 128.48, 128.07, 122.52, 77.58, 77.16, 76.74, 45.13, 27.75, 21.23.

GC-MS: *m/z* (%): 229.00 ([M+2H]$^+$, 8), 227.99 (([M+H]$^+$, 73), 227.01 (M$^+$, 9), 225.99 ([M-H]$^+$, 75), 199.98 (27), 197.99 (28), 185.97 (61), 184.97 (48), 183.97 (63), 182.97 (44), 169.95 (18), 147.06 (11), 119.07 (100), 118.06 (63), 117.06 (55), 115.04 (41), 105.06 (67), 104.05 (33), 103.04 (29), 91.05 (56), 77.04 (25).

3-phenylbutanal (29c)

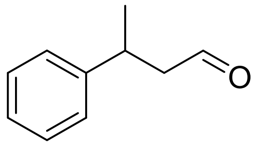

Known Compound.[34] Prepared by following the general experimental procedure with 15.4 mg **Co(acac)₂** (60 µmol), 45.9 mg **L15** (120 µmol), 120 µmol **DIBAL-H** and **29a** (305.0 mg, 1.5 mmol) in 1 mL 2-MeTHF at 100 °C. After hydrolysis of the enamine and extraction, **29c** was purified by vacuum distillation.

Chemical Formula: $C_{10}H_{12}O$

Molecular Weight: 148.2050

Appearance: colorless oil

Isolated Yield: 89% (197.9 mg, 1.34 mmol)

^{1}H NMR (400 MHz, CD_2Cl_2): δ = 9.68 (t, *J* = 2.0 Hz, 1H), 7.34 - 7.27 (m, 2H), 7.26 - 7.17 (m, 3H), 3.35 (h, *J* = 7.1 Hz, 1H), 2.79 - 2.60 (m, 2H), 1.30 (d, *J* = 7.0 Hz, 3H).

^{13}C NMR (101 MHz, CD_2Cl_2) δ = 202.03, 146.19, 128.96, 127.19, 126.79, 54.38, 54.11, 53.84, 53.57, 53.30, 52.03, 34.64, 22.44.

GC-MS: *m/z* (%): 149.08 ($[M+H]^+$, 7), 148.08 (M^+, 61), 147.08 ($[M+H]^+$, 11), 134.06 (5), 133.05 (45), 130.07 (17), 115.04 (11), 106.07 (35), 105.08 (100), 104.07 (11), 103.06 (23), 92.06 (5), 91.05 (48), 79.05 (25), 78.05 (21), 77.05 (30), 65.04 (5), 51.04 (11).

3-(4-fluorophenyl)-3-phenylpropanal (30c)

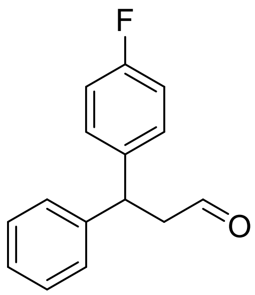

Known Compound.[34] Prepared by following the general experimental procedure with 10.3 mg **Co(acac)₂** (40 µmol), 30.6 mg **L15** (80 µmol), 80 µmol **DIBAL-H** and **30a** (283.4 mg, 1 mmol) in 1 mL 2-MeTHF at 100 °C (reaction time: 48 h). After hydrolysis of the enamine and extraction, **30c** was purified by column chromatography.

Chemical Formula: $C_{15}H_{13}FO$

Molecular Weight: 228.2664

Appearance: white solid

Isolated Yield: 77% (175.8 mg, 0.77 mmol)

^{1}H NMR (300 MHz, CD_2Cl_2): δ = 9.71 (t, *J* = 1.7 Hz, 1H), 7.39 - 7.25 (m, 2H), 7.25 - 7.15 (m, 5H), 7.06 - 6.93 (m, 2H), 4.61 (t, *J* = 7.7 Hz, 1H), 3.16 (ddd, *J* = 7.8, 1.7, 1.1 Hz, 2H).

^{13}C NMR (75 MHz, CD_2Cl_2): δ = 200.86, 163.57, 143.76, 139.84, 129.70, 129.59, 129.14, 128.01, 127.13, 115.90, 115.61, 49.80, 44.53, 1.18.

^{19}F NMR (282 MHz, CD_2Cl_2): δ = -117.18.

GC-MS: *m/z* (%): 229.10 ($[M+H]^+$, 9), 228.10 (M^+, 55), 210.00 (30), 186.00 (20), 185.00 (100), 184.00 (15), 183.00 (53), 170.00 (13), 166.00 (8), 165.00 (52), 133.00 (7), 123.00 (8), 121.00 (8), 109.00 (8), 105.00 (14),103.00 (9), 101.00 (7), 77.00 (10).

3,3'-(1,4-phenylene)dipropanal (31c)

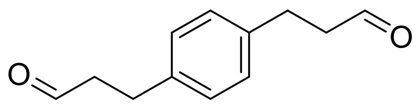

Known Compound.[35] Prepared by following the general experimental procedure with 5.1 mg **Co(acac)$_2$** (20 µmol), 15.3 mg **L15** (40 µmol), 40 µmol **DIBAL-H** and **31a** (150.2 mg, 0.5 mmol) in 1 mL 2-MeTHF at 100 °C (reaction time: 48 h). After hydrolysis of the enamine and extraction, **31c** was purified by column chromatography (using *n*-pentane/ EtOAc).

Chemical Formula: $C_{12}H_{14}O_2$

Molecular Weight: 190.2420

Appearance: white sticky oil

Isolated Yield: 78% (74.2 mg, 0.39 mmol)

^{1}H NMR (300 MHz, CD_2Cl_2): δ = 9.78 (t, *J* = 1.5 Hz, 2H), 7.13 (s, 4H), 2.91 (ddd, *J* = 7.8, 7.1, 1.0 Hz, 4H), 2.78 - 2.69 (m, 4H).

^{13}C NMR (75 MHz, CD_2Cl_2): δ = 201.91, 138.90, 128.83, 45.61, 28.02.

GC-MS: *m/z* (%): 191.09 ([M+H]$^+$, 3), 190.08 (M$^+$, 19), 173.07 (3), 172.06 (22), 147.07 (25), 146.07 (100), 133.05 (11), 129.05 (11), 128.05 (13), 119.07 (8), 118.07 (21), 117.07 (89), 116.06 (9), 115.05 (25), 105.06 (42), 104.05 (39), 103.05 (13), 92.06 (20), 91.05 (46), 79.05 (8), 78.04 (11), 77.04 (14).

11.3 Up-scale Experiment

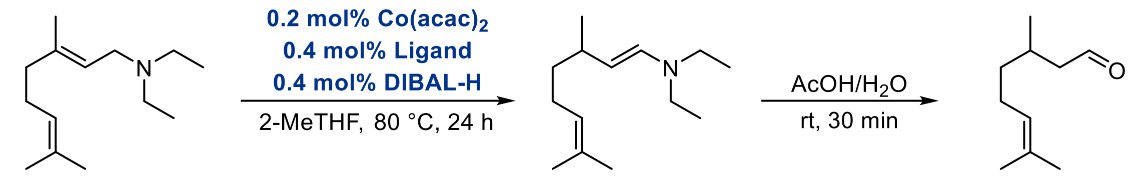

1a - 440 g
(2.1 mol)

1b

1c - 311 g
96% yield

Under argon atmosphere, a 2 L Schlenk flask was charged with $Co(acac)_2$ (1.08 g, 4.2 mmol), **L15** (3.21 g, 8.4 mmol, 2 equiv.) and a stirring bar. Then, 250 mL anhydrous 2-MeTHF was added, followed by the addition of DIBAL-H (8.4 mL, 1 M, 8.4 mmol, 2 equiv.) and the catalyst solution was stirred for approximately 5 min.[17] Next, the anhydrous substrate **1a** (439.9 g, 2.1 mol) was slowly injected and the Schlenk flask was equipped with a condenser. The reaction mixture was heated for 24 h at 80 °C. Afterwards, the solvent was removed under reduced pressure, which yielded a brown crude oil. Then, the Schlenk flask was equipped with a distillation bridge and citronellal enamine **1b** was purified by vacuum distillation. The received colorless product **1b** was hydrolyzed with 400 ml of a 5% acetic acid solution. After 30 min of stirring, the citronellal (**1c**) was extracted with diethyl ether. The organic solution was dried with sodium sulfate and the solvent was removed *in vacuo*. Finally, the obtained product mixture was purified by fractional distillation, yielding citronellal (**1c**) as colorless oil (311 g, 96% yield).

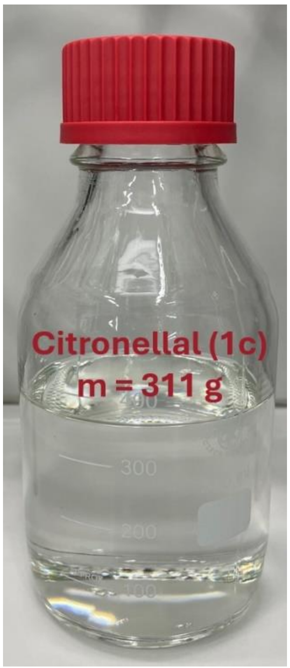

[17]Note: hydrogen is released by the reduction of the Co-precatalyst!

^{1}H NMR and ^{13}C NMR spectrum of **1c** from the up-scale experiment

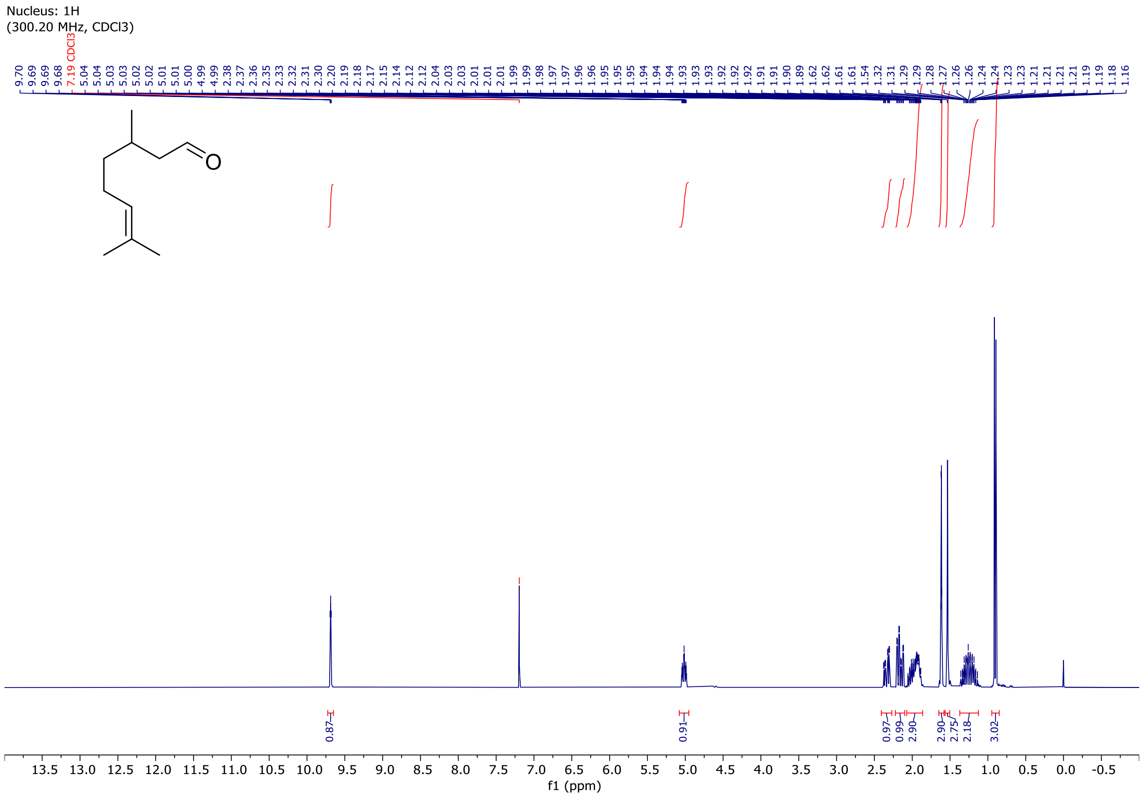

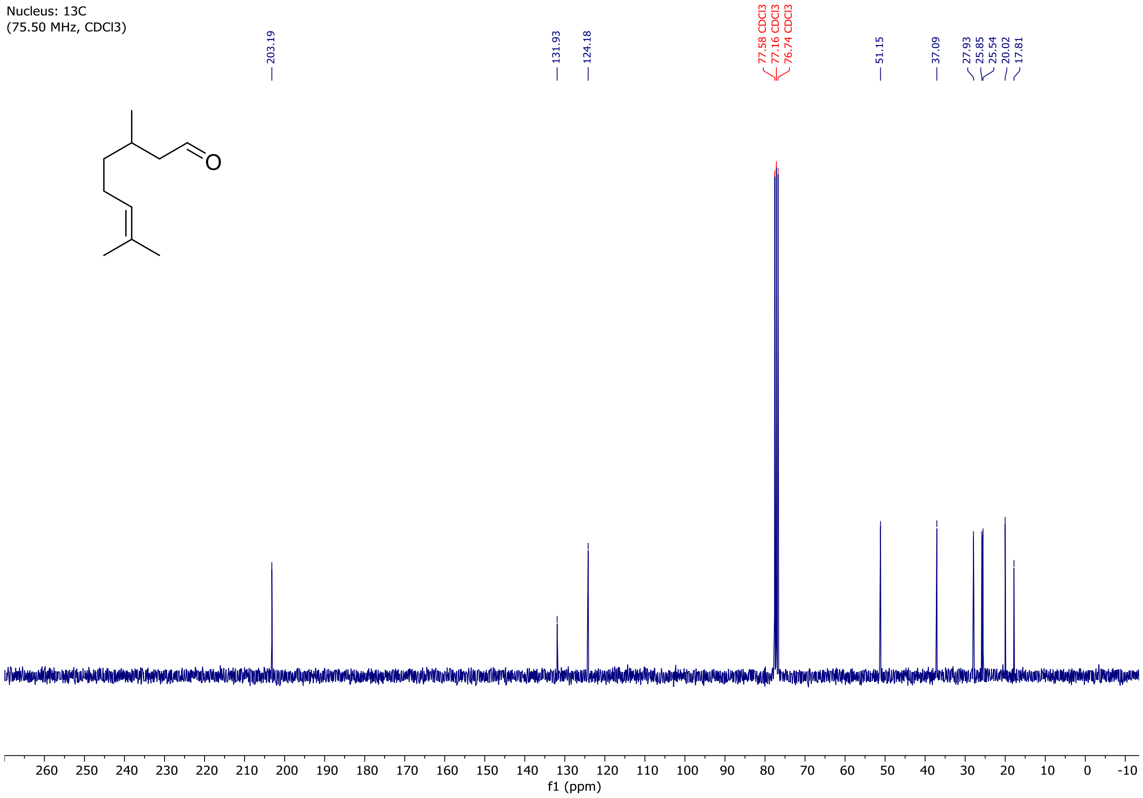

11.4 Failed Substrates

The unsuccessfully tested substrates from the substrate-scope for the Co-catalyzed isomerization of allylamines are summarized in **Table S13**. The reactions have been carried out with the same procedure as described for the initial screening of phosphine ligands (section 3).

Table S13: Unsuccessfully tested allylamine derivatives.

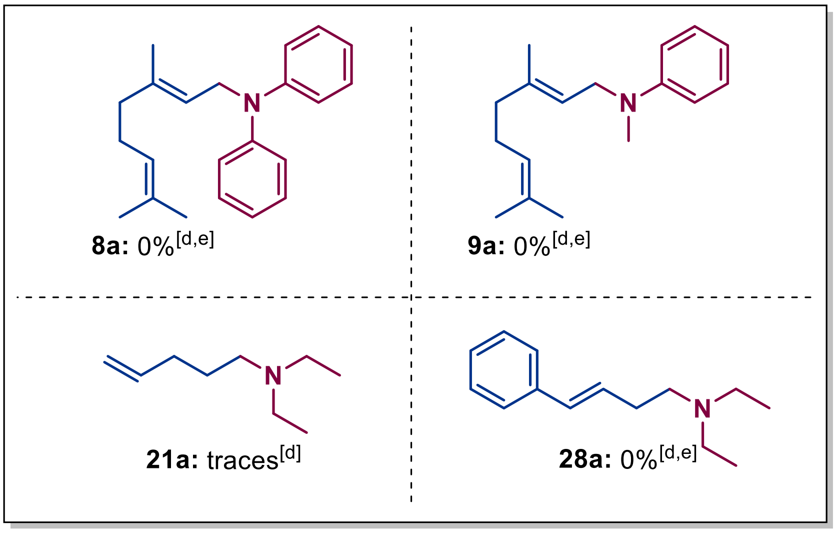

Entry	Substrate	$Co(acac)_2$ [mol%]	DIBAL-H [mol%]	**L15** [mol%]	Yield [%]	Conv. [%]
1[b]	**8a**	4	8	8	-	-
2[b]	**9a**	4	8	8	-	-
3[a]	**21a**	4	8	8	traces	99%
4[b]	**28a**	4	8	8	-	-

Reaction conditions: 1.0 mmol substrate, 2-MeTHF, $Co(acac)_2$, **L15**, DIBAL-H, 100 °C. [a]Reaction time: 24 h. [b]Reaction time: 48 h. The substrate conversions of **8a/9a/21a** were determined by GC using *n*-hexadecane as internal standard. The conversion of **28a** was analyzed by ^1H NMR.

Table S14: Comparison of catalysts for the isomerization of **28a**.

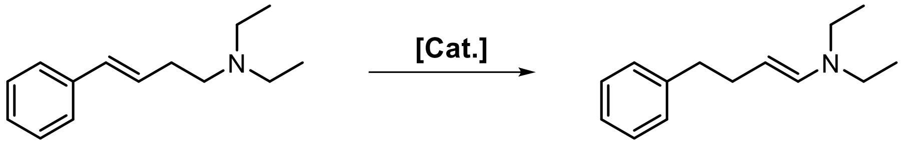

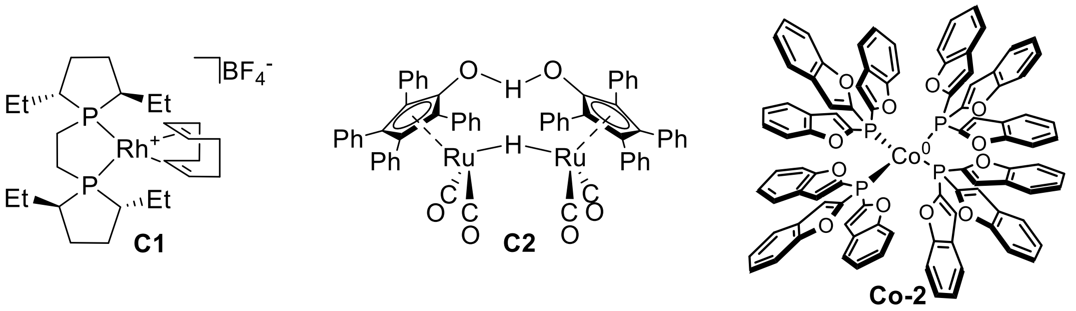

Entry	Catalyst	Cat.-load. [mol%]	Temperature [°C]	Yield [mol%]	Conv. [mol%]
1[a]	**C1**	2	80	-	traces
2[a]	**C1**	4	100	-	traces
3[a]	**C2**	4	100	-	-
4[a]	**Co-2**	4	100	-	traces

[a]General reaction conditions: Substrate **28a** (0.5 mmol), THF-d_8 (1.5 mL), Co(acac)$_2$, **L15**, DIBAL-H, 24 h. The conversion of **28a** was determined by ^{1}H NMR.

12 Computational Methods and Models

All structures have been optimized at first at the M06L[36] level of density functional theory in combination with the all-electron SVP basis set[37] under the consideration of van der Waals dispersion corrections using the GD3[38] parameters (M06L-GD3/SVP) in gas phase. All optimized structures were further characterized either as energy minimums without imaginary frequencies or transition states with only one imaginary frequency by frequency calculations at the same level of theory. Based on the M06L-GD3/SVP optimized gas phase geometries, single-point energy calculation at the B3PW91[39] level of theory with the all-electron TZVP basis set[40] along with thermal correction to Gibbs free energies at 298 K under the consideration of solvation effect based on solute electron density (SMD[41]) model for water as solvent using self-consistent reaction field theory as well as van der Waals dispersion corrections were carried out (B3PW91-GD3-SCRF/TZVP//M06L-GD3/SVP). We therefore used the corrected Gibbs free energy (ΔG) at 298 K for our energetic discussion and comparison. All calculations were carried out by using the Gaussian 16 program.[42]

DFT computational studies with [Co(L)$_4$] and [Co(L)$_3$] complexes.

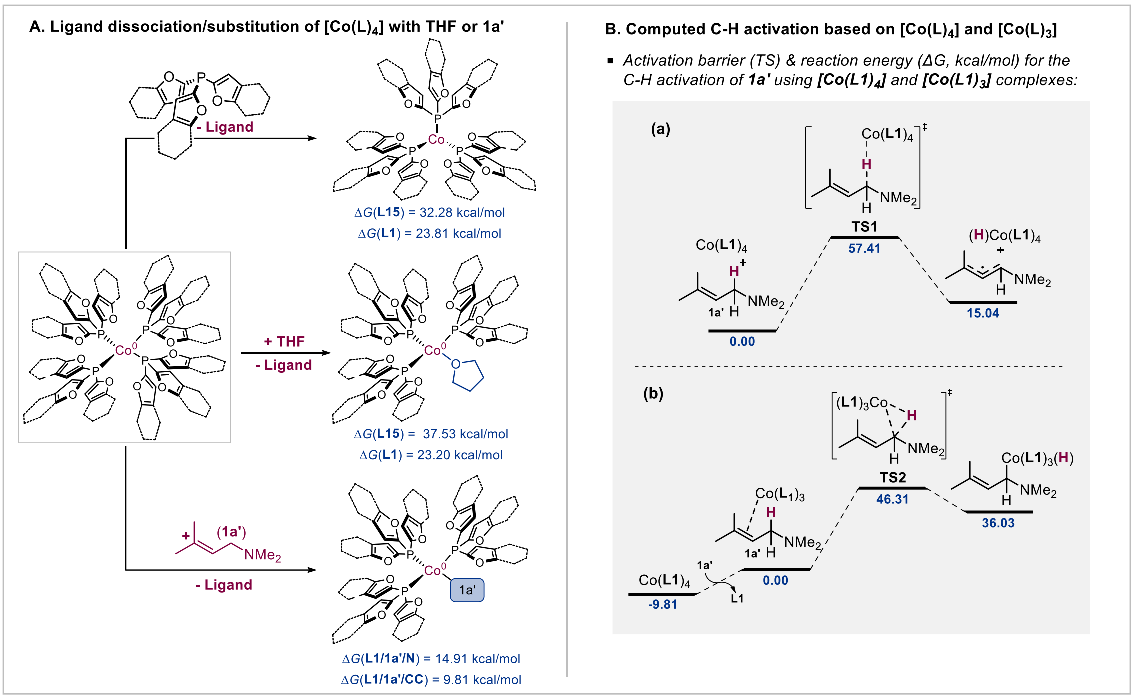

Figure S45: (A) Computed reaction energy (ΔG, kcal/mol) for the ligand dissociation/substitution of [Co(**L**)$_4$] using **L1** and **L15**. (B) Computed C-H activation barrier (TS) and reaction energy (Δ*G*, kcal/mol) for the C-H activation based on [Co(**L1**)$_4$] and [Co(**L1**)$_3$].

Computed ligand dissociation for CoL$_4^-$ and cationic CoL$_4^+$

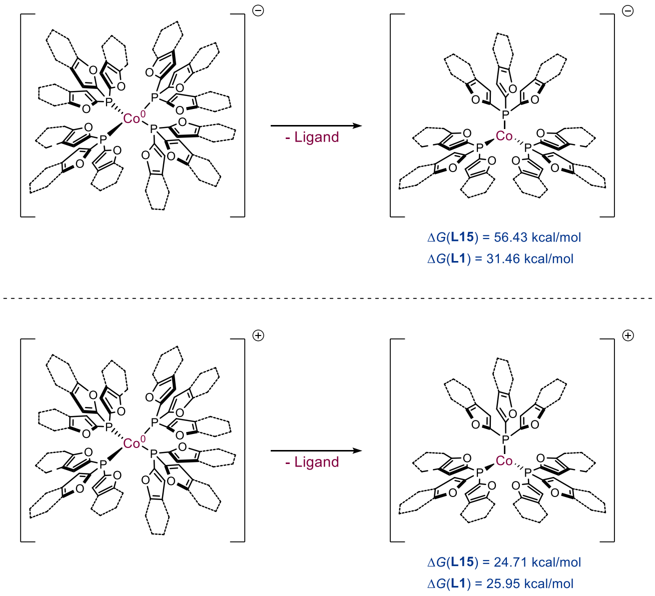

Figure S46: Computed reaction energy (ΔG, kcal/mol) for the ligand dissociation of [Co(**L**)$_4$]$^-$ and [Co(**L**)$_4$]$^+$ using **L1** and **L15**.

Table S15: M06L-GD3/SVP computed total electronic energies (HF, au), zero-point vibrational energies (ZPE, au), sum of electronic and thermal enthalpies (Htot, au), sum of electronic and thermal free energies (Gtot, au), number of Imaginary frequencies (NImag) in gas phase, as well as B3PW91-DG3-SCRF/TZVP//M06L-GD3/SVP/SP computed sum of electronic and thermal free energies (Gtot, au) in solution.

	M06L-GD3/SVP/gas	B3PW91-GD3-SCRF/TZVP
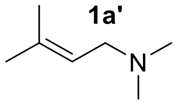<br>**1a'**	HF=-330.208847<br>ZPE= 0.208724<br>NImag=0<br>Htot= -329.988570<br>Gtot= -330.036455	Htot= -330.294415<br>Gtot= -330.339426
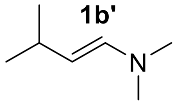<br>**1b'**	HF=-330.2151162<br>ZPE= 0.209453<br>NImag=0<br>Htot= -329.994181<br>Gtot= -330.041181	Htot= -330.300538<br>Gtot= -330.345565
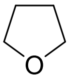	HF=-232.242455<br>ZPE= 0.116681<br>NImag=0<br>Htot= -232.119993<br>Gtot= -232.153029	Htot= -232.338276<br>Gtot= -232.371271
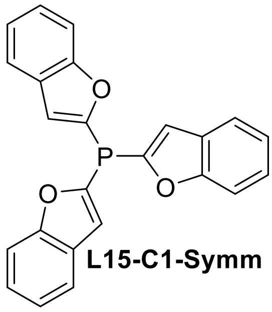<br>**L15-C1-Symm**	HF=-1489.5194364<br>ZPE= 0.324356<br>NImag=0<br>Htot= -1489.173012<br>Gtot=-1489.248959	Htot= -1490.188438<br>Gtot= -1490.260314

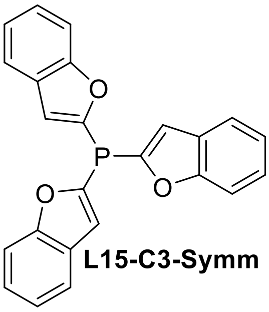<br>**L15-C3-Symm**	HF=-1489.519076<br>ZPE= 0.324142<br>NImag=0<br>Htot= -1489.172816<br>Gtot= -1489.248026	Htot= -1490.188143<br>Gtot= -1490.259070
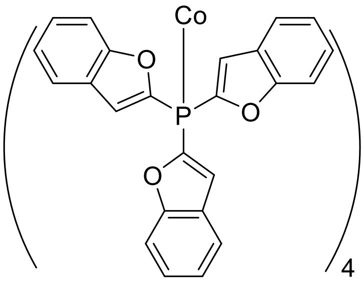<br>**[Co(L15)$_4$] - Radical**	HF=-7340.8513483<br>ZPE= 1.303156<br>NImag=0<br>Htot= -7339.457441<br>Gtot= -7339.680360	Htot= -7343.712317<br>Gtot= -7343.925358
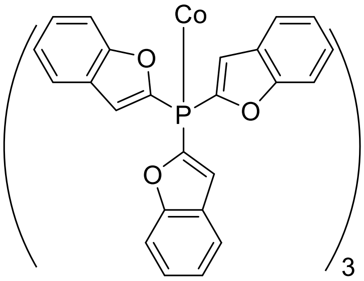<br>**[Co(L15)$_3$] - Radical**	HF=-5851.2136908<br>ZPE= 0.976737<br>NImag=0<br>Htot= -5850.167982<br>Gtot= -5850.349009	Htot= -5853.431115<br>Gtot= -5853.604044
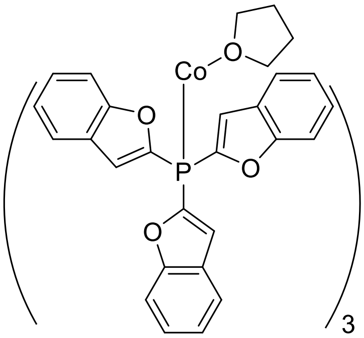<br>**[Co(L15)$_3$(THF)] - Radical**	HF=-6083.5025534<br>ZPE= 1.095546<br>NImag=0<br>Htot= -6082.332184<br>Gtot= -6082.522990	Htot= -6085.796982<br>Gtot= -6085.976437

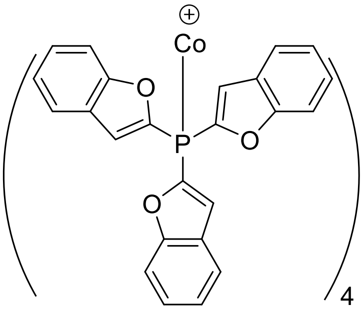<br>**[Co(L15)$_4$]-Cation-Singlet**	HF=-7340.6629476<br>ZPE= 1.304691<br>NImag=0<br>Htot= -7339.267397<br>Gtot= -7339.491714	Htot= -7343.547391<br>Gtot= -7343.760126
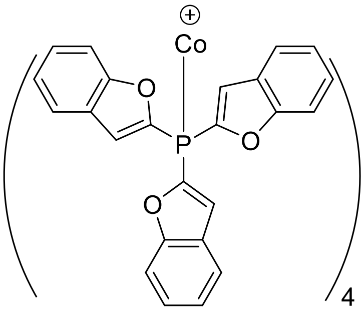<br>**[Co(L15)$_4$]-Cation-Triplet**	HF=-7340.6977905<br>ZPE= 1.304587<br>NImag=0<br>Htot= -7339.302207<br>Gtot= -7339.526696	Htot= -7343.588678<br>Gtot= -7343.801479
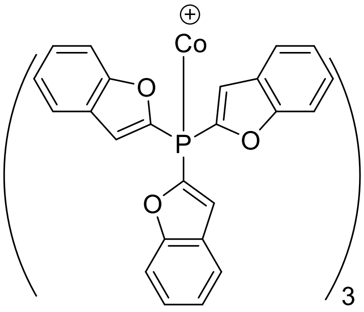<br>**[Co(L15)$_3$]-Cation-Singlet**	HF=-5851.0368582<br>ZPE= 0.978377<br>NImag=0<br>Htot= -5849.989893<br>Gtot= -5850.168563	Htot= -5853.275511<br>Gtot= -5853.446032
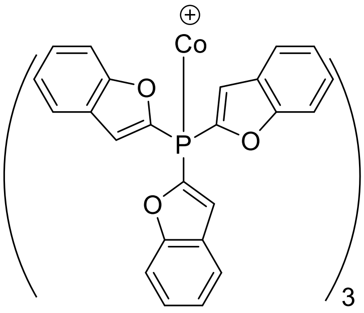<br>**[Co(L15)$_3$]-Cation-Triplet**	HF=-5851.0670054<br>ZPE= 0.977469<br>NImag=0<br>Htot= -5850.021100<br>Gtot= -5850.202028	Htot= -5853.328515<br>Gtot= -5853.501784

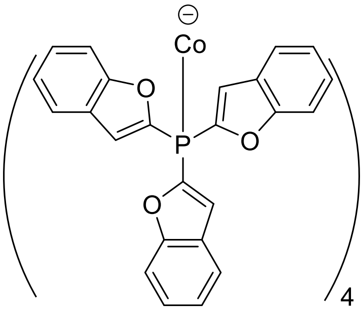<br>**[Co(L15)$_4$]-Anion**	HF=-7340.921889<br>ZPE=1.300272<br>NImag=0<br>Htot=-7339.530643<br>Gtot=-7339.756420	Htot= -7343.816985<br>Gtot= -7344.032557
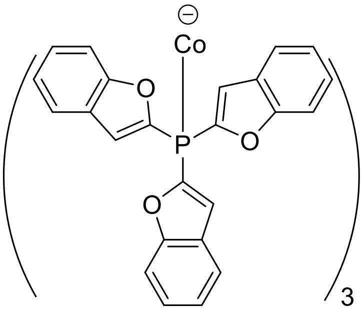<br>**[Co(L15)$_3$]-Anion**	HF=-5851.2639978<br>ZPE= 0.973800<br>NImag=0<br>Htot= -5850.221961<br>Gtot= -5850.400434	Htot= -5853.512540<br>Gtot= -5853.682316
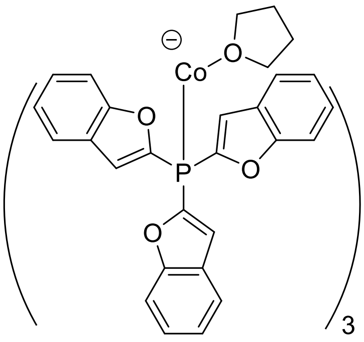<br>**[Co(L15)$_3$(THF)] - Anion**	HF=-6083.5498738<br>ZPE= 1.091570<br>NImag=0<br>Htot= -6082.384885<br>Gtot= -6082.570810	Htot= -6085.873674<br>Gtot= -6086.048938
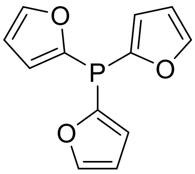<br>**L1-C1-Symm**	HF=-1028.9392595<br>ZPE= 0.182524<br>NImag=0<br>Htot= -1028.742203<br>Gtot= -1028.800441	Htot= -1029.396835<br>Gtot= -1029.452014
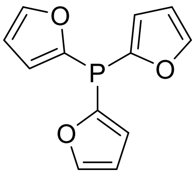<br>**L1-C3-Symm**	HF=-1028.9388237<br>ZPE= 0.182431<br>NImag=0<br>Htot= -1028.741857<br>Gtot= -1028.798795	Htot= -1029.396737<br>Gtot= -1029.451036

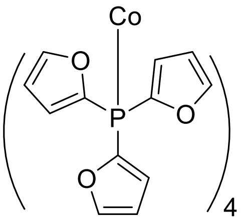<br>**$[Co(L1)_4]$-Radical**	HF=-5498.471443<br>ZPE= 0.735633<br>NImag=0<br>Htot= -5497.675592<br>Gtot= -5497.836579	Htot= -5500.498752<br>Gtot= -5500.652340
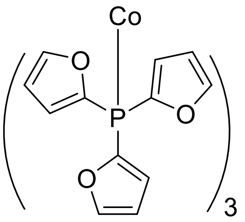<br>**$[Co(L1)_3]$-Radical**	HF=-4469.463635<br>ZPE= 0.551655<br>NImag=0<br>Htot= -4468.866322<br>Gtot= -4468.994498	Htot= -4471.037333<br>Gtot= -4471.162374
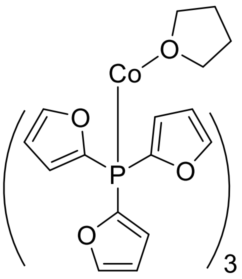<br>**$[Co(L1)_3(THF)]$-Radical**	HF=-4701.7300857<br>ZPE= 0.669372<br>NImag=0<br>Htot= -4701.008091<br>Gtot= -4701.153969	Htot= -4703.398896<br>Gtot= -4703.534618
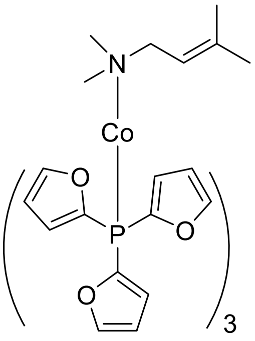<br>**$[Co(L1)_3(1a')]$**<br>***N*-Coordination-Radical**	HF=-4799.7126224<br>ZPE= 0.762738<br>NImag=0<br>Htot= -4798.892049<br>Gtot= -4799.047666	Htot= -4801.369822<br>Gtot= -4801.515982

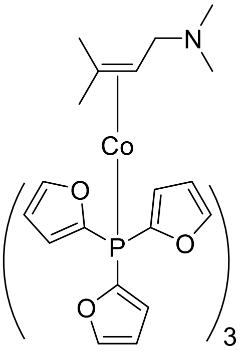 **[Co(L1)$_3$(1a')]** **C=C-Coordination-Radical**	HF=-4799.7262114<br>ZPE= 0.764056<br>NImag=0<br>Htot= -4798.905478<br>Gtot= -4799.055045	Htot= -4801.382269<br>Gtot= -4801.524108
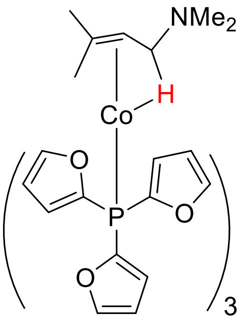 **[Co(L1)$_3$(1a')]** **C=C-Coordination** **CH-activation-TS**	HF=-4799.6564564<br>ZPE= 0.757384<br>NImag=1 (-540.0678)<br>Htot= -4798.842602<br>Gtot= -4798.990603	Htot= -4801.310071<br>Gtot= -4801.450312
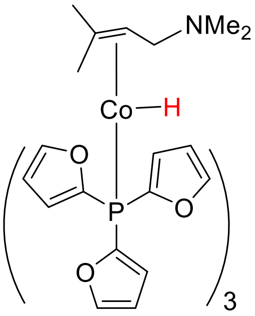 **[Co(L1)$_3$(H)(η^1-allyl)]**	HF=-4799.6754119<br>ZPE= 0.760050<br>NImag=0<br>Htot= -4798.858862<br>Gtot= -4799.006145	Htot= -4801.324220<br>Gtot= -4801.466689
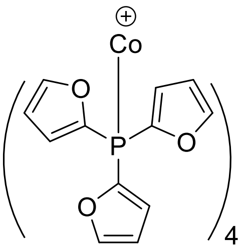 **[Co(L1)$_4$]-Cation-Singlet**	HF=-5498.3031024<br>ZPE= 0.739295<br>NImg=0<br>Htot= -5497.503835<br>Gtot= -5497.661106	Htot= -5500.347766<br>Gtot= -5500.497863

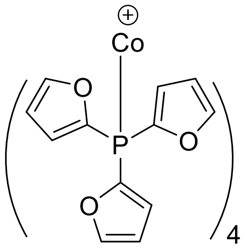<br>**$[Co(L1)_4]$-Cation-Triplet**	HF=-5498.3243045<br>ZPE= 0.736485<br>NImag=0<br>Htot= -5497.526169<br>Gtot= -5497.694294	Htot= -5500.385429<br>Gtot= -5500.539481
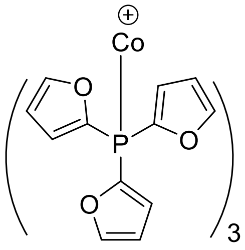<br>**$[Co(L1)_3]$-Cation-Singlet**	HF=-4469.2811862<br>ZPE=0.553663<br>NImag=0<br>Htot=-4468.682130<br>Gtot=-4468.809155	Htot= -4470.884717<br>Gtot= -4471.011429
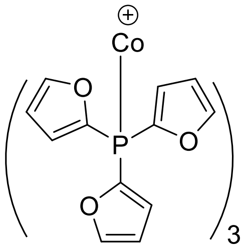<br>**$[Co(L1)_3]$-Cation-Triplet**	HF=-4469.302216<br>ZPE= 0.552718<br>NImag=0<br>Htot= -4468.703226<br>Gtot= -4468.835561	Htot= -4470.922089<br>Gtot= -4471.046644
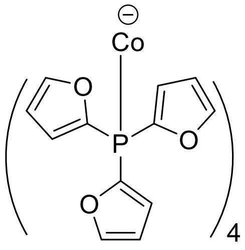<br>**$[Co(L1)_4]$-Anion-Singlet**	HF=-5498.517744<br>ZPE= 0.733328 NImag=0<br>Htot= -5497.723588<br>Gtot= -5497.885196	Htot= -5500.595542<br>Gtot= -5500.738564
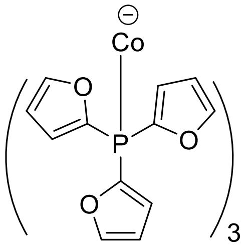<br>**$[Co(L1)_3]$-Anion-Singlet**	HF=-4469.4913693<br>ZPE= 0.547939<br>NImag=0<br>Htot= -4468.897377<br>Gtot= -4469.026397	Htot= -4471.112619<br>Gtot= -4471.236419

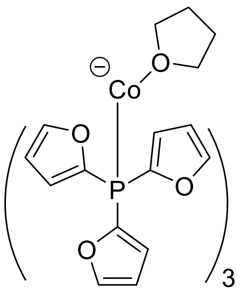<br>**[Co(L1)$_3$(THF)]-Anion-Singlet**	HF=-4701.752886<br>ZPE= 0.666050<br>NIMag=0<br>Htot= -4701.034137<br>Gtot= -4701.179413	Htot= -4703.463619<br>Gtot= -4703.599064
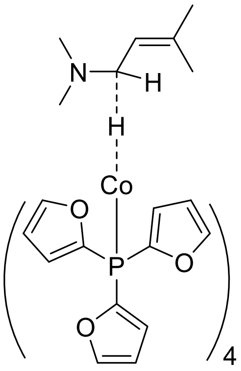<br>**[Co(L1)$_4$(1a')]**<br>**CH-Activation-TS**	HF=-5828.6400452<br>ZPE= 0.941659<br>NImag= 1 (-1086)<br>Htot= -5827.628017<br>Gtot= -5827.802276	Htot= -5830.739232<br>Gtot= -5830.900273
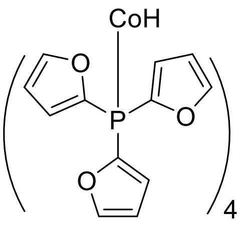<br>**[Co(L1)$_4$H]**	HF=-5499.0715609<br>ZPE=0.743659<br>NIMag=0<br>Htot=-5498.268344<br>Gtot= -5498.429539	Htot= -5501.092394<br>Gtot= -5501.245052
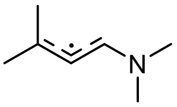<br>**Allyl Radical**<br>**Allyl-amine-SVP-Radical**<br>**-M06L-FOpt**	HF=-329.5843724<br>ZPE=0.195529<br>NImag=0<br>Htot=-329.377018<br>Gtot= -329.425451	Htot= -329.676724<br>Gtot= -329.722753

Table S16: M06L-GD3/SVP optimized coordinates.

1a' 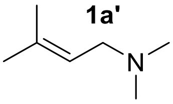	C,0,2.1200088712,-0.5116043942,-0.1988122151<br>C,0,0.9630805159,0.0289860881,0.2246522068<br>H,0,0.9480876822,1.1077203546,0.4254418928<br>C,0,2.3054082099,-1.9533606986,-0.5529477066<br>H,0,3.0372374886,-2.4356689907,0.1153471446<br>H,0,2.7197035691,-2.0530021113,-1.5692354612<br>H,0,1.3811849435,-2.5414638924,-0.510312953<br>C,0,3.3477015555,0.3300947633,-0.3519627946<br>H,0,4.1704861166,-0.0428119207,0.2798123958<br>H,0,3.1705665389,1.3803390939,-0.0874060534<br>H,0,3.7279857037,0.2997881277,-1.386079603<br>C,0,-0.3452292201,-0.6499210365,0.4537580105<br>H,0,-0.640249842,-0.5071985346,1.5088439449<br>H,0,-0.2615792541,-1.7530467555,0.3193062613<br>N,0,-1.4116592912,-0.0907907086,-0.3631614766<br>C,0,-2.7103868212,-0.5408957016,0.0640887964<br>H,0,-2.8747481705,-0.2905440489,1.1223012347<br>H,0,-2.86044876,-1.6399667087,-0.0448793264<br>H,0,-3.4982903167,-0.0452276243,-0.5215265612<br>C,0,-1.2022715509,-0.3190136521,-1.7709626483<br>H,0,-0.2244132325,0.0743809818,-2.082201765<br>H,0,-1.9744690692,0.1969057404,-2.3596992187<br>H,0,-1.2332322547,-1.3975849751,-2.0527296106
1b' 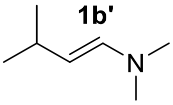	C,0,0.1939042621,0.1134708038,1.2183971667<br>H,0,0.5095022538,-0.777842674,1.7752147233<br>C,0,-0.0608409334,0.030983031,-0.1024595129<br>H,0,-0.3480246341,0.9382080552,-0.6496855521<br>N,0,-0.0478040066,-1.0995544535,-0.8964855452<br>C,0,0.0468791446,1.3749450843,2.0186228272<br>H,0,-0.2310508995,2.1853307388,1.3185047083<br>C,0,1.3618221444,1.7628699607,2.6878595778<br>H,0,2.1650757696,1.9061715232,1.9516931755<br>H,0,1.6944816459,0.9768858114,3.3845587133<br>H,0,1.2655479257,2.6922454583,3.2693371462<br>C,0,-1.0670242722,1.2539682334,3.0554109003<br>H,0,-2.0342234147,1.0257897392,2.5862711419<br>H,0,-1.183884731,2.1797169442,3.6393528759<br>H,0,-0.8521132805,0.4435690858,3.7701250506

	C,0,0.2330460974,-0.9404606905,-2.2986781293<br>H,0,-0.2426522699,-0.0241124895,-2.6733350754<br>H,0,-0.1766426823,-1.7858527226,-2.8708052157<br>H,0,1.3144981593,-0.8715848457,-2.5329443281<br>C,0,0.35803204,-2.3383192881,-0.2957556252<br>H,0,-0.2402330927,-2.5375998595,0.6056472459<br>H,0,1.4248156802,-2.3510023541,0.00770594<br>H,0,0.195551912,-3.165183847,-0.9994978122
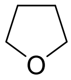	H,0,0.0047118172,-0.0313448409,0.0341388827<br>C,0,-0.0241007203,0.0110227806,1.1315368432<br>C,0,1.0365415817,0.9183721308,1.7280216936<br>O,0,0.5338603429,1.3387923186,2.9848308377<br>C,0,-0.8415450484,1.0136924327,3.0924452449<br>C,0,-1.2944334855,0.6281160113,1.6959274327<br>H,0,0.0966844611,-1.0195603359,1.5028596715<br>H,0,1.2168386094,1.7988031582,1.0795068375<br>H,0,2.0093686823,0.4216560608,1.8732275067<br>H,0,-1.3872440579,1.8752804557,3.5096775778<br>H,0,-0.9765476131,0.171953965,3.8006034124<br>H,0,-1.5714868446,1.5252078487,1.1188321769<br>H,0,-2.1619069825,-0.0459381352,1.6889847399
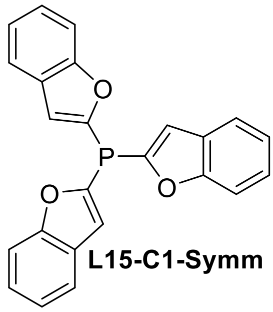**L15-C1-Symm**	P,0,0.4235756515,-0.2310096173,-0.0815977119<br>C,0,-0.474927083,0.3468704126,1.3896714029<br>C,0,-0.5680303234,-1.7136798782,-0.4219769884<br>C,0,-0.2590246328,0.8747651417,-1.3425829535<br>C,0,-1.5113743672,-0.1670167105,2.1154876664<br>C,0,-1.4552011399,-2.0442828547,-1.4066355156<br>O,0,-1.602143613,1.1760233719,-1.3375390498<br>H,0,-2.0665495709,-1.071360748,1.8777223355<br>H,0,-1.8157628111,-1.3789855726,-2.1870033244<br>C,0,-1.709346721,0.725055631,3.219519747<br>C,0,-1.8144718772,-3.41375644,-1.1830442042<br>C,0,-1.8140221759,2.0269307271,-2.3722678765<br>C,0,-0.7431503236,1.7375088351,3.0598633415<br>C,0,-1.0909678786,-3.808616699,-0.0412130972<br>C,0,-0.6086861648,2.2832928516,-3.0557373205<br>O,0,0.0108175574,1.4994809426,1.9549583478<br>O,0,-0.32829595,-2.7790049586,0.412187534

	C,0,0.3850237646,1.5243891022,-2.3580194379<br>C,0,-0.6061966275,2.814886175,3.9255612702<br>C,0,-1.1702441868,-5.076216658,0.5210739307<br>C,0,-0.6259803229,3.1448051969,-4.1643631008<br>H,0,0.1561361704,3.5777432381,3.7628835719<br>H,0,-0.5919004189,-5.3390431256,1.4076467782<br>H,0,0.2905322215,3.3649069236,-4.7156860665<br>C,0,-1.4883481891,2.8606549636,5.00211579<br>C,0,-2.019631645,-5.9813968558,-0.1103322194<br>C,0,-1.8377485773,3.7092773618,-4.5412939927<br>H,0,-1.4200278709,3.686212095,5.7138179153<br>H,0,-2.1148901062,-6.9928348133,0.2903796252<br>H,0,-1.8746484014,4.382103228,-5.4006103443<br>C,0,-2.5873129272,0.7959939804,4.3126732579<br>C,0,-2.6653442054,-4.3450354907,-1.7990551055<br>C,0,-3.0350017788,2.5840304609,-2.73490431<br>H,0,-3.3497308178,0.0295332436,4.4670841231<br>H,0,-3.2427406782,-4.0733306163,-2.6852417161<br>H,0,-3.9454860105,2.3620020964,-2.1771671917<br>C,0,-2.4641450496,1.8658368639,5.1912534542<br>C,0,-2.7556451418,-5.6198229193,-1.2527427465<br>C,0,-3.0244866404,3.433292426,-3.8373548589<br>H,0,-3.1379241422,1.9390511398,6.0477030169<br>H,0,-3.4115495462,-6.3590683294,-1.717590665<br>H,0,-3.9585653103,3.8965218695,-4.1624008652<br>H,0,1.4498348613,1.4600200086,-2.5691094463
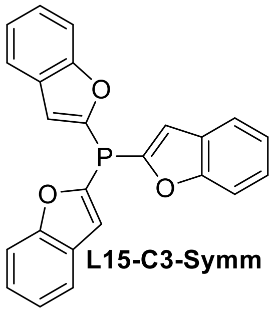<br>**L15-C3-Symm**	P,0,0.2975547157,0.,0.<br>C,0,-0.5834532959,0.4402585491,1.5253991933<br>C,0,-0.5834532959,-1.5411637269,-0.3814245089<br>C,0,-0.5834532959,1.1009051778,-1.1439746844<br>C,0,-1.560219231,-0.1850735967,2.2475502052<br>C,0,-1.560219231,-1.8538987756,-1.2840535389<br>C,0,-1.560219231,2.0389723723,-0.9634966663<br>H,0,-2.0589365462,-1.1114384106,1.9709468502<br>H,0,-2.0589365462,-1.1511708365,-1.9480073234<br>H,0,-2.0589365462,2.262609247,-0.0229395268<br>C,0,-1.7838665793,0.6253940468,3.4080507975<br>C,0,-1.7838665793,-3.2641555914,-1.1624182669<br>C,0,-1.7838665793,2.6387615446,-2.2456325307

	C,0,-0.8928168711,1.7094032464,3.2840354161<br>C,0,-0.8928168711,-3.6987597205,-0.1616310713<br>C,0,-0.8928168711,1.989356474,-3.1224043447<br>O,0,-0.1585241237,1.5886737879,2.1470318612<br>O,0,-0.1585241237,-2.6537210285,0.302315928<br>O,0,-0.1585241237,1.0650472406,-2.4493477893<br>C,0,-0.8039979418,2.7427901997,4.2082195445<br>C,0,-0.8039979418,-5.0158201301,0.2712162179<br>C,0,-0.8039979417,2.2730299304,-4.4794357624<br>H,0,-0.099294276,3.5640853241,4.0726525929<br>H,0,-0.099294276,-5.3090632683,1.0502621355<br>H,0,-0.099294276,1.7449779441,-5.1229147283<br>C,0,-1.6559708634,2.6652633224,5.3067802405<br>C,0,-1.6559708634,-5.9284381618,-0.3452043752<br>C,0,-1.6559708634,3.2631748393,-4.9615758653<br>H,0,-1.6228628965,3.4512410511,6.064267038<br>H,0,-1.6228628965,-6.9774298357,-0.0432710942<br>H,0,-1.6228628965,3.5261887847,-6.0209959438<br>C,0,-2.6327756106,0.572269561,4.5250485673<br>C,0,-2.6327756106,-4.2049417931,-1.766924306<br>C,0,-2.6327756106,3.6326722321,-2.7581242613<br>H,0,-3.336344664,-0.2530075656,4.6529686573<br>H,0,-3.336344664,-3.9030852775,-2.5455953078<br>H,0,-3.336344664,4.156092843,-2.1073733495<br>C,0,-2.5565527847,1.5960639816,5.4615340591<br>C,0,-2.5565527847,-5.5278592296,-1.3485350754<br>C,0,-2.5565527847,3.931795248,-4.1129989837<br>H,0,-3.208173518,1.5735299947,6.3376475928<br>H,0,-3.2081735179,-6.275328813,-1.8061068474<br>H,0,-3.2081735179,4.7017988183,-4.5315407454
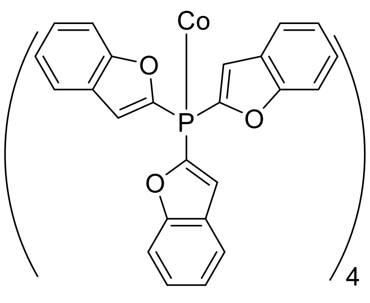<br>**[Co(L15)$_4$] - Radical**	Co,0,-0.1073942868,-0.131267912,0.0858128883<br>P,0,0.6610542612,1.5552226786,-1.0910803653<br>P,0,0.7717173922,0.2591050838,2.03431825<br>P,0,0.6963518225,-1.8419698768,-0.9870936681<br>P,0,-2.2481506193,0.0336913779,0.0075386199<br>O,0,-0.1098529535,3.8451066966,-2.4508040015<br>O,0,-0.1233923572,0.3074152126,4.6662731067<br>O,0,-0.1652174419,-4.1166513503,-2.338659559<br>C,0,-0.3193435334,3.06542375,-1.3353695045

C,0,-0.2823779971,-0.2618065143,3.422768132
C,0,-0.3480454508,-2.7634115723,-2.1591326624
C,0,-1.2817381201,3.59874342,-0.528312917
C,0,-1.2435451615,-1.2316581114,3.4399625576
C,0,-1.338426349,-2.2835206565,-2.9657849788
H,0,-1.6203248177,3.1630875747,0.409806886
H,0,-1.5459087936,-1.8275725328,2.5804252705
H,0,-1.6626942767,-1.2452312882,-3.001115971
C,0,-1.7189401249,4.8045104376,-1.1639876528
C,0,-1.7262454754,-1.300637949,4.7857286799
C,0,-1.8257434285,-3.4011670064,-3.7159444329
C,0,-2.6637966429,5.8032312335,-0.8807003985
C,0,-2.6823320482,-2.0651839459,5.4723674446
C,0,-2.8215595349,-3.6031436288,-4.6840124226
H,0,-3.2666039709,5.7581882309,0.0301454472
H,0,-3.2579837917,-2.8346717583,4.9520758017
H,0,-3.4281661332,-2.7675905826,-5.0424815766
C,0,-2.8171012917,6.8437919295,-1.7889779484
C,0,-2.8790016394,-1.8163116098,6.8254715053
C,0,-3.0194601111,-4.890459424,-5.1695306012
H,0,-3.5462059678,7.6320552832,-1.5882860116
H,0,-3.6166070489,-2.4013465381,7.3795605864
H,0,-3.7886619678,-5.0701372953,-5.9240857543
C,0,-2.0525350101,6.9033065362,-2.9684154391
C,0,-2.1480138178,-0.8221205536,7.5001936211
C,0,-2.2481478465,-5.9726291185,-4.7082094668
H,0,-2.2013548696,7.7347530036,-3.6610164466
H,0,-2.3307098169,-0.6520783774,8.5636209338
H,0,-2.4316937633,-6.9709486096,-5.1117569692
C,0,-1.1106735173,5.9242626797,-3.2736949867
C,0,-1.1950824953,-0.0488601059,6.841475459
C,0,-1.255967235,-5.7993112507,-3.7466170857
H,0,-0.514542666,5.9522843062,-4.1868625209
H,0,-0.6234452966,0.7295594736,7.3488258677
H,0,-0.654706433,-6.6290750789,-3.3723013834
C,0,-0.9664468641,4.8944673779,-2.3519567355
C,0,-1.0051566522,-0.3171335746,5.4917392069
C,0,-1.0695750813,-4.505758046,-3.2746061953
O,0,3.139284738,1.4383454181,-0.0320806675

	O,0,3.0297014325,-1.134715971,1.5095979456
	O,0,3.2810645703,-1.1519292438,-1.4927888938
	C,0,2.2010661583,2.3263155903,-0.4946511833
	C,0,2.3290626271,-0.5616064596,2.5377508867
	C,0,2.2057337425,-1.8290255735,-2.0050957965
	C,0,2.6134434217,3.6180989881,-0.3191307958
	C,0,2.9203587124,-0.8287795112,3.7424963108
	C,0,2.4788097147,-2.3219310771,-3.2522991029
	H,0,2.0495395252,4.5095660691,-0.5887120825
	H,0,2.5506785724,-0.4980240104,4.711230613
	H,0,1.7934818229,-2.8883522909,-3.8793834513
	C,0,3.9066207912,3.5465555848,0.2964977773
	C,0,4.083682337,-1.6162307154,3.4584309434
	C,0,3.8241255074,-1.9327068948,-3.5534923375
	C,0,4.8519418744,4.4700665526,0.7685554152
	C,0,5.1137008301,-2.2100302595,4.2063804671
	C,0,4.7015592712,-2.0920254577,-4.6367773702
	H,0,4.6765669126,5.5442070819,0.6751762179
	H,0,5.1393637699,-2.1176534183,5.2943390264
	H,0,4.3936147292,-2.646538792,-5.5260520777
	C,0,6.005029491,3.9837356947,1.3744300317
	C,0,6.0984091399,-2.9180846308,3.5285098741
	C,0,5.961284594,-1.5098719485,-4.5569101475
	H,0,6.7517545429,4.6866240331,1.7509624352
	H,0,6.9075113492,-3.3875001303,4.0925056597
	H,0,6.6586044989,-1.6192366554,-5.390690885
	C,0,6.2336029051,2.6025544641,1.5184808713
	C,0,6.0783931868,-3.0467170799,2.1268742653
	C,0,6.3588936696,-0.7732990517,-3.4258634754
	H,0,7.148473495,2.2580943508,2.0054560064
	H,0,6.8699696701,-3.6119872111,1.6299069554
	H,0,7.353791145,-0.3232474018,-3.4018965667
	C,0,5.3146598215,1.6639505799,1.0554370135
	C,0,5.0693367693,-2.4711125578,1.3603347826
	C,0,5.5101914451,-0.6063375713,-2.3337122692
	H,0,5.4672860147,0.5882094531,1.1682396397
	H,0,5.0289132396,-2.564742644,0.2737741564
	H,0,5.7991604391,-0.0361923202,-1.4488327935
	C,0,4.1692819873,2.1710871294,0.4527104569

C,0,4.0917407825,-1.7698549051,2.0574716122
C,0,4.2580230798,-1.2011579924,-2.4303795105
O,0,0.3386855846,0.4480616522,-3.5558110515
O,0,0.2470647343,2.9061020071,2.1599520563
O,0,0.661301031,-2.959924896,1.4810501055
C,0,1.2013428295,1.1969482455,-2.7922734564
C,0,1.1826082631,1.964375891,2.5150015965
C,0,1.1729363898,-3.1276982863,0.2144540961
C,0,2.3704065762,1.434335727,-3.4574844971
C,0,2.2874562831,2.5514224134,3.0647821949
C,0,2.1074332515,-4.1227868496,0.1929425716
H,0,3.2204748378,1.990591318,-3.0671034101
H,0,3.1751371838,2.0305781555,3.4166896272
H,0,2.6646694974,-4.4400670456,-0.6864903903
C,0,2.2440637702,0.8024953258,-4.7371060515
C,0,2.0428231388,3.9634543181,3.0547583888
C,0,2.2162300731,-4.6122484019,1.5347778021
C,0,3.0591092615,0.6485486052,-5.8681873019
C,0,2.7591759173,5.1110870258,3.4265760016
C,0,3.0004911233,-5.56877488,2.1987283176
H,0,4.0594070037,1.0864108598,-5.8980184933
H,0,3.7554849083,5.0272001044,3.8665244012
H,0,3.7313579779,-6.1684771871,1.6516948122
C,0,2.5702252067,-0.092779886,-6.9377300898
C,0,2.1772345754,6.3540054596,3.2036364614
C,0,2.8340249441,-5.7230857971,3.5695758026
H,0,3.1912199991,-0.2291785419,-7.8261462878
H,0,2.7201481641,7.2602758329,3.4815775078
H,0,3.4409497478,-6.4559643728,4.1058275277
C,0,1.2926266471,-0.6803132236,-6.9003537101
C,0,0.9003747449,6.4731291898,2.6253895642
C,0,1.9039821723,-4.9493209405,4.2871078775
H,0,0.9441206618,-1.2608355282,-7.7573794683
H,0,0.4758850957,7.4659629661,2.4615396908
H,0,1.8042276216,-5.0941025998,5.3650308231
C,0,0.4603444469,-0.537554593,-5.791923764
C,0,0.1655117581,5.3497564955,2.2533753873
C,0,1.1119569382,-3.9946316774,3.6546816607
H,0,-0.5306563395,-0.99338729,-5.7439228867

H,0,-0.8230753495,5.4263280034,1.7972884643
H,0,0.3953989374,-3.3769566149,4.1990110088
C,0,0.9677838002,0.2064681569,-4.7338211761
C,0,0.7680862038,4.1175958147,2.4775951148
C,0,1.2987014036,-3.850157371,2.2848362978
O,0,-2.3874406183,1.0149634606,-2.4836044617
O,0,-2.5371055202,1.7038628439,2.0897542192
O,0,-2.2677495664,-2.6187618852,0.3524619788
C,0,-3.0524523826,1.0188253542,-1.2820787835
C,0,-3.1316095288,0.619138317,1.4897050102
C,0,-3.004765996,-1.602778399,-0.2163377643
C,0,-4.0211950347,1.9836343464,-1.2611394573
C,0,-4.0561943206,0.0499516658,2.3219838192
C,0,-3.9817075255,-2.106922344,-1.0283585106
H,0,-4.6690460447,2.2073190333,-0.4156062759
H,0,-4.6469963839,-0.8357858726,2.0953991373
H,0,-4.6852369876,-1.5138473682,-1.6097873543
C,0,-3.958967114,2.6497297765,-2.5295017416
C,0,-4.0391351551,0.8133474187,3.5360429899
C,0,-3.8524201491,-3.5352482349,-0.9877714818
C,0,-4.6149119387,3.7343565687,-3.133230212
C,0,-4.6817469524,0.7434680602,4.7819511307
C,0,-4.487013426,-4.624244649,-1.6058298586
H,0,-5.4085652171,4.2707043425,-2.6084847637
H,0,-5.4251524452,-0.0304537909,4.985459648
H,0,-5.317878187,-4.4690556132,-2.2975373867
C,0,-4.2156842111,4.1203177047,-4.4068252728
C,0,-4.3337482283,1.6702609261,5.757411937
C,0,-4.0198024521,-5.9037182009,-1.3292812704
H,0,-4.704053931,4.9707959747,-4.88779925
H,0,-4.8123466362,1.6226736739,6.7382063692
H,0,-4.4921669587,-6.7630024553,-1.810618643
C,0,-3.1855644225,3.4479308586,-5.0894672134
C,0,-3.3661927217,2.6621911427,5.5163820127
C,0,-2.940345985,-6.1190582027,-0.4535001818
H,0,-2.8945359029,3.7859574006,-6.0865178509
H,0,-3.1113509687,3.3662651204,6.3116513935
H,0,-2.5954803681,-7.1388124298,-0.2685624416
C,0,-2.5185342619,2.3688631105,-4.5161958022

	C,0,-2.7141918002,2.7567127906,4.289478165<br>C,0,-2.2926945091,-5.0578398347,0.1739951867<br>H,0,-1.704794496,1.8461381687,-5.020772721<br>H,0,-1.9463704896,3.505444271,4.0886795695<br>H,0,-1.4420712782,-5.2000305818,0.843101933<br>C,0,-2.9263671925,2.0059446353,-3.2386712051<br>C,0,-3.0725563036,1.8171191806,3.3310914685<br>C,0,-2.7726832539,-3.7883155033,-0.1200417591
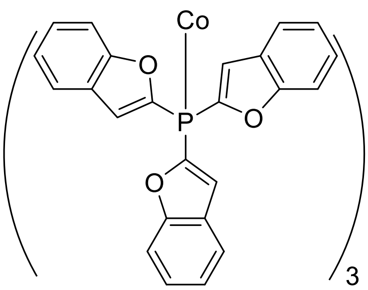<br>**[Co(L15)$_3$] - Radical**	Co,0,0.0616932498,-0.1011267954,0.0465599053<br>P,0,0.5362310073,-1.920129068,-0.9253066573<br>P,0,0.9359381407,1.4962172429,-1.1393806832<br>P,0,0.7793217571,0.2903174798,2.0119722657<br>O,0,0.1582681909,3.9778786835,-2.1283070055<br>O,0,-0.0351588819,0.1258337876,4.6652451903<br>O,0,-0.199752334,-4.1638130586,-2.4142794539<br>C,0,-0.1480404177,2.9391515912,-1.28098742<br>C,0,-0.3184663555,-0.219921917,3.3649376161<br>C,0,-0.4818930234,-2.850087112,-2.1189654365<br>C,0,-1.3140510844,3.1749158497,-0.6102162371<br>C,0,-1.4410704675,-0.9955282771,3.295585508<br>C,0,-1.5029567937,-2.3799279832,-2.8944178748<br>H,0,-1.7413609819,2.5144297962,0.1418006063<br>H,0,-1.8521436202,-1.4173165605,2.3802009774<br>H,0,-1.8954966253,-1.3656463227,-2.8573815762<br>C,0,-1.7840974514,4.4543302298,-1.0449821695<br>C,0,-1.9044889091,-1.1682569371,4.6391340259<br>C,0,-1.8939868031,-3.4559327702,-3.7546242037<br>C,0,-2.8763092788,5.2839169212,-0.7441219263<br>C,0,-2.9634790645,-1.8464190361,5.2634156945<br>C,0,-2.8413263242,-3.6399737502,-4.7743256081<br>H,0,-3.6351519849,4.9692845597,-0.0242290006<br>H,0,-3.6846372177,-2.4175272453,4.6749588709<br>H,0,-3.5080557179,-2.8270403092,-5.0710214431<br>C,0,-2.9648132005,6.5155175101,-1.3811940283<br>C,0,-3.0698035545,-1.7749880106,6.6471111451<br>C,0,-2.9102444511,-4.8806321932,-5.3964687653<br>H,0,-3.8053629907,7.1769230327,-1.1605687946<br>H,0,-3.8856489317,-2.2968476358,7.151815453<br>H,0,-3.6389030301,-5.044103523,-6.1934286155

C,0,-1.9925406159,6.9314428511,-2.3090561419
C,0,-2.1468108581,-1.0432493338,7.4157843753
C,0,-2.0600691749,-5.9361823183,-5.020172454
H,0,-2.0954358568,7.9060747757,-2.7909121717
H,0,-2.2620943329,-1.0090991404,8.5012700325
H,0,-2.1427578035,-6.8979838737,-5.5309750972
C,0,-0.9011650554,6.1275677129,-2.6268608035
C,0,-1.0870033687,-0.3623381122,6.8221566353
C,0,-1.1133702827,-5.7811001482,-4.010935236
H,0,-0.1386641815,6.4355625924,-3.3432679842
H,0,-0.3624336254,0.2092304159,7.4035126544
H,0,-0.4463421713,-6.5890084301,-3.7075933332
C,0,-0.8266515454,4.9009018084,-1.978581717
C,0,-0.9933942037,-0.4482386615,5.438690258
C,0,-1.0537662081,-4.5323881016,-3.4038290278
O,0,3.3576036722,1.3925765815,0.034091327
O,0,2.9627648717,-1.2235499964,1.5380697927
O,0,3.1172812089,-1.2269088223,-1.3779459335
C,0,2.4702412411,2.2680611421,-0.5438858948
C,0,2.3300687231,-0.5306073614,2.5335135865
C,0,2.0514894358,-1.8797261287,-1.9398447088
C,0,2.9010384938,3.5604176182,-0.4205101592
C,0,3.0022372073,-0.6510587448,3.7195292899
C,0,2.3496396443,-2.3161400997,-3.2022498365
H,0,2.3760630198,4.4430795847,-0.7796838099
H,0,2.6983221749,-0.2040435193,4.6635577578
H,0,1.6798100808,-2.8522098537,-3.8711928862
C,0,4.1509956497,3.5024761149,0.2783732872
C,0,4.1528442943,-1.4625092788,3.4515010356
C,0,3.701988143,-1.9154767156,-3.4578838118
C,0,5.0869749282,4.4373980904,0.7464903521
C,0,5.2477863324,-1.9452940943,4.1874353199
C,0,4.5997937765,-2.0229171518,-4.5307570103
H,0,4.9412336403,5.5061081327,0.5740124038
H,0,5.3485108347,-1.7228195268,5.2519597194
H,0,4.3084435185,-2.5312086534,-5.4526891907
C,0,6.1906773454,3.9700931969,1.4506759064
C,0,6.2020957113,-2.7089367515,3.5265719432
C,0,5.8593170445,-1.4491895079,-4.3972841663

H,0,6.92952245,4.6813982766,1.8268236504
H,0,7.0618934505,-3.0917992859,4.0809180299
H,0,6.5719413647,-1.518366424,-5.2224471124
C,0,6.3788681411,2.5967842249,1.6950377115
C,0,6.0886013291,-3.002496448,2.1542424791
C,0,6.2391262612,-0.7762149374,-3.2215081638
H,0,7.2552970823,2.26732598,2.2572029467
H,0,6.8602721468,-3.6054225894,1.6704209674
H,0,7.2361686163,-0.3354333439,-3.154173475
C,0,5.469347725,1.6468393721,1.2370863228
C,0,5.0129631184,-2.5415761286,1.4014592847
C,0,5.3698685332,-0.6618106088,-2.1387634748
H,0,5.5942312263,0.5782151121,1.4279102526
H,0,4.9004386005,-2.7585323538,0.3377422412
H,0,5.644223082,-0.1417002361,-1.2191150869
C,0,4.3721458337,2.1356254891,0.5362669806
C,0,4.0683894196,-1.7805972978,2.081025148
C,0,4.1160598087,-1.2404553418,-2.2930099174
O,0,0.4229942944,0.4851566532,-3.5792329794
O,0,0.2537402205,2.9211871786,2.0880580969
O,0,0.5511705027,-2.9790554635,1.5573001459
C,0,1.3778805154,1.1807897227,-2.8731470012
C,0,1.1962047623,2.0007690797,2.4898305986
C,0,1.0277082265,-3.1984365659,0.2836380547
C,0,2.5069833868,1.3677209438,-3.6177308564
C,0,2.3093976548,2.6249284167,2.9794921836
C,0,1.9929757732,-4.1654298529,0.2867610485
H,0,3.4096598978,1.8771618834,-3.2856497087
H,0,3.2082735267,2.131853368,3.3429493713
H,0,2.5368165363,-4.5100875734,-0.5910546437
C,0,2.2595258563,0.7505161695,-4.8875806554
C,0,2.0658706858,4.0335174917,2.8756905381
C,0,2.1643637833,-4.5718831253,1.6495876519
C,0,2.9904270333,0.556059536,-6.068895445
C,0,2.7939160406,5.2011230938,3.1497459196
C,0,3.0125412496,-5.45085189,2.3413477856
H,0,4.0064890174,0.9451820283,-6.1649134494
H,0,3.7938183161,5.1436644018,3.5858437892
H,0,3.7436855996,-6.0598998131,1.8049818893

	C,0,2.3981794571,-0.1623037644,-7.1012553434<br>C,0,2.2202836614,6.4271973515,2.8316635524<br>C,0,2.9142639954,-5.5109186179,3.7262749371<br>H,0,2.9519975256,-0.3292243137,-8.0279559914<br>H,0,2.7730165521,7.3482000529,3.0309869818<br>H,0,3.5736743109,-6.1798568233,4.2837724217<br>C,0,1.0988112291,-0.6864552104,-6.9777290218<br>C,0,0.9395803063,6.5097931163,2.2558151817<br>C,0,1.9876288912,-4.7214242744,4.4311485463<br>H,0,0.6675883945,-1.2516947752,-7.806866741<br>H,0,0.5220798705,7.4894320406,2.0129307653<br>H,0,1.9446058322,-4.7893935841,5.5203511459<br>C,0,0.3483599349,-0.5004023091,-5.8188108264<br>C,0,0.1901149565,5.3666272928,1.9864876556<br>C,0,1.1289663302,-3.8465210894,3.7704999122<br>H,0,-0.6580934404,-0.9066591614,-5.7058308047<br>H,0,-0.802296356,5.4155914202,1.5351443889<br>H,0,0.4132626343,-3.2162295055,4.3023178799<br>C,0,0.9577341245,0.2194540595,-4.7986897322<br>C,0,0.783261562,4.1514642826,2.3070285119<br>C,0,1.2479606005,-3.7953188,2.3865128003
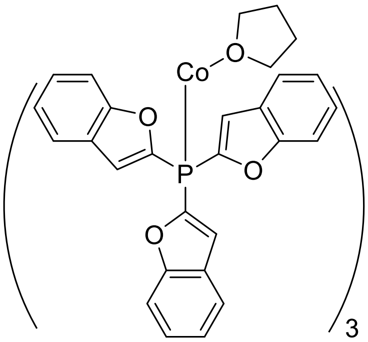<br>**[Co(L15)$_3$(THF)] - Radical**	Co,0,0.0048777217,0.3323822479,0.1831290079<br>P,0,0.2968544327,-0.7237710114,1.9891071808<br>P,0,-1.8863738592,0.1157962131,-0.8059415415<br>P,0,1.5086778567,-0.4627020279,-1.1557539789<br>O,0,0.3969643339,2.3493910489,0.3888461226<br>C,0,1.1769688683,2.9490844842,1.4358541453<br>H,0,2.24973911,2.8864771526,1.1717188642<br>H,0,1.0150918846,2.3643270115,2.3512028715<br>C,0,-0.099529571,3.3639230338,-0.5103072905<br>H,0,-1.202118429,3.3313656511,-0.4744163729<br>H,0,0.2138951373,3.1085699263,-1.5315365236<br>C,0,0.4614209384,4.6730787001,0.0124993747<br>H,0,1.4233818114,4.9037359023,-0.4729955935<br>H,0,-0.210886653,5.5216362368,-0.1707562448<br>C,0,0.6846575948,4.3752498046,1.4886062344<br>H,0,1.393642047,5.0582725044,1.974590993<br>H,0,-0.266284073,4.4259784665,2.0456083133<br>C,0,-3.0326645263,1.2285969315,0.0630796467

C,0,-2.8471530715,1.8348620051,1.2750335431
H,0,-1.9427908942,1.7633909449,1.8773422909
C,0,-4.0451159324,2.562366852,1.5593159642
C,0,-4.8844730342,2.338280547,0.4490140342
O,0,-4.2706051632,1.5320477926,-0.4559131516
C,0,-2.9603248302,-1.3614630949,-0.8140185401
C,0,-4.0958835928,-1.656894355,-0.1096426391
H,0,-4.6347905963,-0.9753056394,0.5443493285
C,0,-4.4230214783,-3.0161209199,-0.4184368432
C,0,-3.4185902387,-3.4472046761,-1.3080182187
O,0,-2.5348634728,-2.4438939919,-1.5416110056
C,0,-2.0996214856,0.6142089602,-2.5506776789
C,0,-3.0285285759,0.373087094,-3.522334605
H,0,-3.9380346675,-0.2099130697,-3.3895827287
C,0,-2.5604154605,1.0281715608,-4.7084715217
C,0,-1.3429872701,1.6340380291,-4.3412600295
O,0,-1.063775484,1.3786956323,-3.0400701941
C,0,1.9091531281,-0.6270499236,2.8517710936
C,0,2.49975075,-1.2703455814,3.9027522513
H,0,2.0313257474,-2.0407729528,4.5117043887
C,0,3.8268859029,-0.7441140768,4.0132525517
C,0,3.9283923095,0.2173881419,2.9870811852
O,0,2.7684777292,0.2939834156,2.2973763997
C,0,0.074814967,-2.5314503368,2.0215719519
C,0,0.9607625271,-3.5713209234,2.0912018147
H,0,2.0228278003,-3.4974800245,2.3097149371
C,0,0.2124770427,-4.7626424746,1.8247798418
C,0,-1.1129055504,-4.3359801135,1.6189622328
O,0,-1.1909901241,-2.980044291,1.7256743161
C,0,-0.8441617307,-0.2136840422,3.3195993194
C,0,-2.0798788326,-0.6446651575,3.7177013024
H,0,-2.5476425258,-1.5774655359,3.4130395619
C,0,-2.6123842038,0.3753717524,4.5733269651
C,0,-1.626131668,1.3798770127,4.6237965418
O,0,-0.5592065599,1.0265022445,3.8559963922
C,0,2.5245711321,-1.9185063755,-0.740886687
C,0,2.490774618,-3.2005134632,-1.2125246643
H,0,1.8984011489,-3.5492015908,-2.0551895885
C,0,3.3657538358,-3.9645332441,-0.3728085755

C,0,3.8801974196,-3.0489414678,0.5649161469
O,0,3.3532127224,-1.8134812474,0.3510109869
C,0,1.087634251,-0.8851273805,-2.8709288983
C,0,1.5479381386,-0.4823201239,-4.0924760732
H,0,2.4270342349,0.133515104,-4.2677798021
C,0,0.6283253376,-1.0029164392,-5.0611437943
C,0,-0.3388902945,-1.7072082245,-4.3188800069
O,0,-0.0556817087,-1.6340929406,-2.9928646288
C,0,2.8020560012,0.7890954391,-1.4228500813
C,0,4.0665978431,0.9466196478,-0.9262072805
H,0,4.6240999543,0.1972306485,-0.3705446252
C,0,4.4733040184,2.2784072867,-1.2638723752
C,0,3.3848078961,2.8375326933,-1.9617485761
O,0,2.3732878414,1.9362076022,-2.0536787571
C,0,-4.5098264145,3.3598421011,2.6170369122
H,0,-3.8863610209,3.5345872293,3.497570937
C,0,-5.785172238,3.9025429008,2.5190567097
H,0,-6.1699903544,4.5227468325,3.3316791515
C,0,-6.1603443633,2.8775010153,0.3338292522
H,0,-6.7798280565,2.6833871959,-0.5426709384
C,0,-6.5973834251,3.6674157783,1.3944223979
H,0,-7.5947416303,4.1108861845,1.3527629298
C,0,-3.0098134233,1.1730319588,-6.0309108727
H,0,-3.9447544592,0.7114775527,-6.3565048894
C,0,-2.2363370864,1.9144669524,-6.9159382072
H,0,-2.5703011601,2.038061277,-7.9486277059
C,0,-1.025508688,2.5078636983,-6.5141469485
H,0,-0.4422585619,3.0788456887,-7.2401979486
C,0,-0.5537457334,2.3761506162,-5.2112422139
H,0,0.3907786669,2.8129770997,-4.8799514129
C,0,-5.4338308286,-3.9207479497,-0.0547550907
H,0,-6.2300044961,-3.6229616684,0.631156462
C,0,-3.3643153117,-4.7250336818,-1.8485372247
H,0,-2.5565027757,-5.0231343228,-2.5186110488
C,0,-4.3767148238,-5.6042571084,-1.4692655291
H,0,-4.3741056482,-6.6235887228,-1.8617232752
C,0,-5.3960363166,-5.2060735509,-0.586631289
H,0,-6.1735137353,-5.9234962465,-0.3141786106
C,0,0.5047638306,-6.1333210678,1.7504575357

H,0,1.5252260267,-6.4926883867,1.9024878131

C,0,-0.5364174155,-7.0152815804,1.4815870056

H,0,-0.3318146112,-8.0865360445,1.4178845811

C,0,-1.852347616,-6.5576398349,1.2954532272

H,0,-2.649241715,-7.2757073634,1.0877660513

C,0,-2.1680752262,-5.2020491275,1.3665732882

H,0,-3.1872819825,-4.8382548014,1.2251072602

C,0,4.7772815875,-3.3861449636,1.5693982625

H,0,5.1530273238,-2.6378631905,2.2704792889

C,0,5.1545649377,-4.7268148029,1.6325530592

H,0,5.8545717553,-5.0485155521,2.406683948

C,0,4.6530872915,-5.6708902383,0.719224841

H,0,4.9733277146,-6.711940666,0.8029457737

C,0,3.764729565,-5.3071532065,-0.2877002326

H,0,3.3808021229,-6.0494145557,-0.9908386037

C,0,0.4864035804,-0.9323777809,-6.4562029103

H,0,1.2081265223,-0.3825287847,-7.0644013075

C,0,-0.6025511282,-1.5660268657,-7.0420606646

H,0,-0.7354279554,-1.5142668109,-8.1251225126

C,0,-1.5468460831,-2.2668238295,-6.270212583

H,0,-2.3939890001,-2.7459033629,-6.766376814

C,0,-1.4312238609,-2.35195232,-4.8854960263

H,0,-2.1635070451,-2.8688087379,-4.2645562701

C,0,5.6187921858,3.0652119011,-1.0622931351

H,0,6.4855134382,2.6641920561,-0.531450636

C,0,5.6270196087,4.3645834167,-1.558400142

H,0,6.5092377382,4.9912000813,-1.410490061

C,0,3.3773396837,4.1290732822,-2.4723539066

H,0,2.5130130358,4.5209438525,-3.0122437448

C,0,4.5243058081,4.8907368583,-2.253431739

H,0,4.5668097562,5.9144040805,-2.6315066653

C,0,4.9434843696,-0.9782432638,4.8327292194

H,0,4.9012856211,-1.7146874272,5.6382066373

C,0,6.1042824259,-0.2532132698,4.5909880144

H,0,6.9844497174,-0.4224708645,5.215170454

C,0,6.1724194712,0.7003742042,3.5581060671

H,0,7.101981783,1.2515359383,3.3998166799

C,0,5.0789279314,0.9567058998,2.7352989717

H,0,5.1105756011,1.6954507142,1.9320038073

	C,0,-1.7486944784,2.5485643696,5.3625681558<br>H,0,-0.9519893105,3.2941635783,5.3804979732<br>C,0,-3.7987311166,0.5647298329,5.299806325<br>H,0,-4.589182497,-0.1884671259,5.2829921617<br>C,0,-3.9442832937,1.732090401,6.0426350625<br>H,0,-4.8616811928,1.8944546157,6.6128341361<br>C,0,-2.9355264786,2.7095017331,6.0782981119<br>H,0,-3.0814771472,3.6116830602,6.6764941967
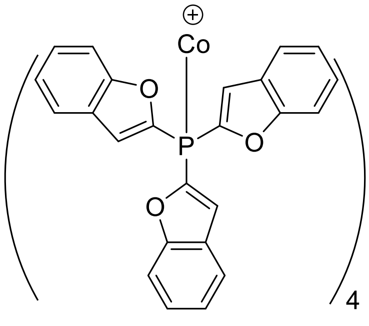<br>**[Co(L15)$_4$]-Cation-Singlet**	Co,0,0.0169793488,0.0762485717,0.0246799649<br>P,0,0.4390009863,1.5367307698,-1.5211678433<br>P,0,0.5986727523,0.0725479428,2.131675687<br>P,0,1.1783042162,-1.6306064672,-0.7633064941<br>P,0,-2.1311623613,0.2811568304,0.2132041433<br>O,0,-0.5532828499,3.7024327333,-2.871150177<br>O,0,-0.3529959463,0.3801606264,4.6892260206<br>O,0,0.265198838,-3.7278575094,-2.267693626<br>C,0,-0.5354874146,3.0512578423,-1.6643793657<br>C,0,-0.4807618558,-0.3312482838,3.5219552082<br>C,0,0.0703452228,-2.3983719502,-1.9721172447<br>C,0,-1.293140247,3.6976071191,-0.7295777443<br>C,0,-1.3685001211,-1.3600555214,3.651652093<br>C,0,-0.9467833976,-1.866185344,-2.7101241191<br>H,0,-1.4426013643,3.3694365213,0.2983163174<br>H,0,-1.6297493823,-2.0645339695,2.8642658736<br>H,0,-1.2964855779,-0.8392765514,-2.6677772009<br>C,0,-1.8364890002,4.8489713202,-1.3840443955<br>C,0,-1.8442305487,-1.3100757414,5.0009231965<br>C,0,-1.439429694,-2.9233193359,-3.5374106185<br>C,0,-2.688943073,5.9031975691,-1.0167017777<br>C,0,-2.7476877424,-2.0569545409,5.7745240914<br>C,0,-2.461022465,-3.0512631192,-4.4915051234<br>H,0,-3.0881647902,5.973759101,-0.0019961087<br>H,0,-3.2733357556,-2.9147882939,5.3491888013<br>H,0,-3.0875620625,-2.1954257381,-4.7566043475<br>C,0,-3.0135611701,6.8501212108,-1.9792065908<br>C,0,-2.9516659432,-1.6763384461,7.0945543833<br>C,0,-2.6540086189,-4.2930861754,-5.0825066711<br>H,0,-3.6731624359,7.6799938853,-1.717700629<br>H,0,-3.6453121155,-2.244267259,7.7178830527

	H,0,-3.4401756058,-4.4197497191,-5.8296784644<br>C,0,-2.5091177816,6.7653457524,-3.2905692562<br>C,0,-2.2804790837,-0.5717476917,7.6510241764<br>C,0,-1.8533280827,-5.3989287213,-4.7387639095<br>H,0,-2.7864594703,7.5298096651,-4.0192596431<br>H,0,-2.4664973223,-0.303872765,8.6931923583<br>H,0,-2.0339850659,-6.3594902591,-5.2257761475<br>C,0,-1.6662879749,5.7293432573,-3.6812100173<br>C,0,-1.385156144,0.1884156335,6.9044424002<br>C,0,-0.8370860663,-5.2988740223,-3.7931353699<br>H,0,-1.2720180338,5.647194316,-4.6947339153<br>H,0,-0.8620808904,1.0504994732,7.320050678<br>H,0,-0.2139378286,-6.1491966035,-3.5137587519<br>C,0,-1.3516726115,4.791572691,-2.7056090366<br>C,0,-1.1884055229,-0.2100394605,5.5881779244<br>C,0,-0.6575349805,-4.0487272833,-3.2125114181<br>O,0,2.8855822744,1.2670712241,-0.3962411442<br>O,0,3.0764331644,-0.9910046131,1.7749693864<br>O,0,3.886046949,-1.3162105457,-1.1748616058<br>C,0,1.9661135603,2.1959247296,-0.8166152001<br>C,0,2.0998920896,-0.7637222771,2.7100983856<br>C,0,2.6920617768,-1.6470154177,-1.7624582061<br>C,0,2.4142299926,3.4728848474,-0.6153679076<br>C,0,2.4049085823,-1.3478686397,3.9061700503<br>C,0,2.8385723577,-1.8574353756,-3.106276818<br>H,0,1.8742650387,4.3858072952,-0.8601960749<br>H,0,1.7887556928,-1.3299259974,4.8026181794<br>H,0,2.0487696451,-2.1256097591,-3.8064084943<br>C,0,3.7165000542,3.3490248403,-0.0350868755<br>C,0,3.6617502405,-2.0093421426,3.7196587905<br>C,0,4.2222817735,-1.6400349823,-3.3990805403<br>C,0,4.6984293467,4.2448867541,0.4182268892<br>C,0,4.4964377347,-2.8139962934,4.5113052448<br>C,0,5.022044163,-1.6909528228,-4.5519304374<br>H,0,4.5474669492,5.3234771231,0.3407119859<br>H,0,4.2407760541,-3.0317639192,5.5502092171<br>H,0,4.5886602914,-1.9570996906,-5.5183126877<br>C,0,5.8534027944,3.7208495854,0.9822565393<br>C,0,5.646759864,-3.3358630933,3.9341341052

C,0,6.3734688642,-1.3950449974,-4.4274698887
H,0,6.6303831181,4.3969281629,1.3449800732
H,0,6.3084007321,-3.9681443389,4.5294326219
H,0,7.0165334464,-1.4283555924,-5.3091645017
C,0,6.0467059728,2.3307562413,1.1016817689
C,0,5.9739425999,-3.0767702766,2.5908738175
C,0,6.9365577799,-1.054513749,-3.1840597409
H,0,6.9657210014,1.9570934472,1.5581991947
H,0,6.8794102347,-3.5161194456,2.1670634439
H,0,8.0021017976,-0.8241771772,-3.1246704404
C,0,5.0947554623,1.4214479629,0.651421639
C,0,5.1635723193,-2.2827357442,1.7835854447
C,0,6.1668215356,-1.003885134,-2.0249235766
H,0,5.2216976957,0.3433536468,0.7443221014
H,0,5.3866911678,-2.1027987436,0.7319230981
H,0,6.5991381481,-0.7357678317,-1.0597941684
C,0,3.9470448439,1.9641525887,0.085552985
C,0,4.0222623043,-1.7616581794,2.3815393443
C,0,4.8162639261,-1.3019444971,-2.1684136145
O,0,0.0590593063,0.6633682095,-4.0733901963
O,0,0.2747497078,2.7976173392,1.9919922431
O,0,0.7958098693,-3.0497085398,1.5259367084
C,0,0.9687854329,1.2348777318,-3.2219397629
C,0,1.1047252668,1.7896661462,2.4155237053
C,0,1.5287563957,-3.0041344444,0.3653315236
C,0,2.2040285942,1.3336425015,-3.7974172481
C,0,2.2525863515,2.2905687588,2.9684481803
C,0,2.4689902447,-3.9923566603,0.3307017341
H,0,3.1002406526,1.7331755397,-3.3256481719
H,0,3.0739770121,1.7025427456,3.3729770945
H,0,3.1788681327,-4.1634086886,-0.475984535
C,0,2.0765431896,0.778054049,-5.1111707333
C,0,2.1390842966,3.7151221599,2.9085466688
C,0,2.339077108,-4.7155308794,1.5611075049
C,0,2.9424864737,0.5497767216,-6.1923446792
C,0,2.9436582338,4.8009435865,3.2887591191
C,0,2.9993780076,-5.7962208691,2.1671403406
H,0,3.9902093276,0.8535703986,-6.1416682276
H,0,3.9110007535,4.6372655226,3.767955011

H,0,3.8227679163,-6.3036586881,1.6605332157
C,0,2.4326550046,-0.0810865079,-7.3204672996
C,0,2.4812479255,6.0836666421,3.0271309118
C,0,2.5822958422,-6.1976139154,3.4299750309
H,0,3.0874315105,-0.2705073164,-8.1735575007
H,0,3.0896079316,6.9446158484,3.311467005
H,0,3.0834237428,-7.0341305773,3.9209619972
C,0,1.0865947678,-0.4849595157,-7.390701508
C,0,1.2384349804,6.3019845351,2.402666804
C,0,1.5261294063,-5.5484716359,4.0953087708
H,0,0.7249612491,-0.9856559137,-8.290965828
H,0,0.9075347882,7.3253532773,2.214404722
H,0,1.2275057699,-5.8925566643,5.0876449864
C,0,0.2046684523,-0.2628473338,-6.3362298199
C,0,0.4168528606,5.2435838991,2.0248379547
C,0,0.8541488437,-4.4741271172,3.5181142286
H,0,-0.8383729147,-0.580592556,-6.3743349985
H,0,-0.5494897544,5.4049890272,1.5449661972
H,0,0.0400003125,-3.9551429488,4.0276398631
C,0,0.7327462605,0.3718391983,-5.2191348103
C,0,0.8996379512,3.9665945882,2.2895112581
C,0,1.2903594058,-4.0852462208,2.2574666508
O,0,-2.6004143902,1.0320120861,-2.3285673996
O,0,-2.3918675497,1.8491181452,2.3601225734
O,0,-1.9001082416,-2.3994868604,0.5064652199
C,0,-3.0854650464,1.1489354569,-1.0490673858
C,0,-2.9836069663,0.792377342,1.7181613356
C,0,-2.6846301868,-1.4182115433,-0.0492899783
C,0,-4.0774542975,2.0850670571,-0.9771934603
C,0,-3.972389971,0.2361153301,2.4797005174
C,0,-3.6231994976,-1.9497294442,-0.8905763853
H,0,-4.609998316,2.3755954256,-0.0735637338
H,0,-4.5745854865,-0.6282489838,2.2062053325
H,0,-4.3465928522,-1.3820639883,-1.4731937094
C,0,-4.2299997198,2.6131608567,-2.3009813559
C,0,-4.0100420662,0.9900814929,3.6983640842
C,0,-3.4218612628,-3.3669785856,-0.8755873452
C,0,-5.0205822721,3.6076390813,-2.8994773984
C,0,-4.7427015253,0.9343213934,4.8960397859

	C,0,-4.003032264,-4.4688262379,-1.5249229948<br>H,0,-5.7515424995,4.1694957202,-2.3147373779<br>H,0,-5.5199070358,0.1819076307,5.0438901132<br>H,0,-4.8363994603,-4.3351803383,-2.2174171723<br>C,0,-4.8395539296,3.8628379074,-4.2521877453<br>C,0,-4.4443655252,1.8564274814,5.8897118824<br>C,0,-3.4775421318,-5.7282259323,-1.2728318271<br>H,0,-5.4374580729,4.6368443814,-4.7374729748<br>H,0,-4.9959975065,1.8271834561,6.83158847<br>H,0,-3.904899681,-6.5989760008,-1.7741830216<br>C,0,-3.8954223,3.1503760551,-5.0147112193<br>C,0,-3.4382081502,2.8266934051,5.7180352762<br>C,0,-2.3971251763,-5.9104190277,-0.3885594857<br>H,0,-3.7795009262,3.3845377021,-6.0750257449<br>H,0,-3.2318851677,3.5300387429,6.5276177419<br>H,0,-2.0105027612,-6.917274318,-0.2180676408<br>C,0,-3.0974288879,2.1611756911,-4.4473537654<br>C,0,-2.6955064437,2.9035598691,4.5443817894<br>C,0,-1.8049140276,-4.8382810322,0.2715272385<br>H,0,-2.3445757271,1.6152129364,-5.0164799794<br>H,0,-1.9003003104,3.6360095263,4.3953041689<br>H,0,-0.9634839455,-4.9678673854,0.9534105809<br>C,0,-3.2899007716,1.9270933426,-3.0922563995<br>C,0,-3.0082510636,1.9700072312,3.564546309<br>C,0,-2.3384522481,-3.584336136,-0.0020237785
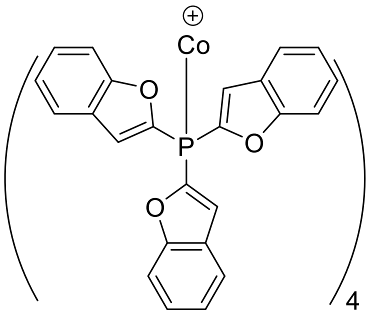<br>**[Co(L15)$_4$]-Cation-Triplet**	Co,0,0.1102905111,-0.1807400073,0.1224713245<br>P,0,0.7368623427,1.6386936943,-1.1196873325<br>P,0,0.7514955343,0.3869135736,2.2592305687<br>P,0,0.6829621834,-2.0203219772,-1.1613761051<br>P,0,-2.1673955479,-0.0760415871,0.05175706<br>O,0,-0.1128980738,3.898570882,-2.4152308473<br>O,0,-0.227818433,0.417014039,4.832019104<br>O,0,-0.2638824046,-4.2213156428,-2.4877696154<br>C,0,-0.2970081632,3.1049790261,-1.3087811206<br>C,0,-0.3150705937,-0.1717713147,3.5969955204<br>C,0,-0.4137111149,-2.8686910553,-2.3021002066<br>C,0,-1.2689405517,3.6017326699,-0.4886447836<br>C,0,-1.2216911685,-1.193866759,3.5847235827<br>C,0,-1.4177743548,-2.3584067268,-3.0734963658

H,0,-1.5935797027,3.1540787598,0.448613543
H,0,-1.4598834077,-1.8169723058,2.7230438734
H,0,-1.7176577261,-1.3121532793,-3.1039533567
C,0,-1.7432147869,4.8002076976,-1.1116670678
C,0,-1.7566810073,-1.267625345,4.9106129677
C,0,-1.9598401049,-3.4649998756,-3.8023772978
C,0,-2.7168348094,5.7672316467,-0.8132250734
C,0,-2.7091634927,-2.0652644519,5.5651108963
C,0,-2.9987903026,-3.6398141034,-4.7305678567
H,0,-3.3143962216,5.6967293606,0.0991735554
H,0,-3.2242757004,-2.8695645574,5.0349993835
H,0,-3.5977515059,-2.7904044273,-5.0668126691
C,0,-2.9012194032,6.8118562096,-1.7097464722
C,0,-2.9780651727,-1.8019205838,6.9018626764
C,0,-3.2445233437,-4.9212328599,-5.2064181826
H,0,-3.6509318185,7.5773030713,-1.4992679973
H,0,-3.713013593,-2.4097170008,7.4334810865
H,0,-4.0460627886,-5.0832261864,-5.9298833067
C,0,-2.1394328772,6.9075709928,-2.8894691314
C,0,-2.3211048888,-0.7658420767,7.5916045468
C,0,-2.4785709562,-6.0214383938,-4.7777064986
H,0,-2.3122408315,7.7441363909,-3.5696901575
H,0,-2.5589802427,-0.5897964773,8.6426905705
H,0,-2.7007860114,-7.0136430784,-5.175859027
C,0,-1.1707471437,5.9611625404,-3.2097635753
C,0,-1.373702526,0.039171196,6.966149567
C,0,-1.4443865713,-5.8753115548,-3.8580842261
H,0,-0.5762916657,6.0198682969,-4.1223011066
H,0,-0.8593914297,0.847775708,7.4868471837
H,0,-0.8471426667,-6.71974146,-3.5120544737
C,0,-0.9984101843,4.9243545374,-2.3006356875
C,0,-1.1144505405,-0.2406389314,5.6302683665
C,0,-1.2130651164,-4.5869040849,-3.3921119279
O,0,3.1550920004,1.4787036401,0.0360999973
O,0,2.8763588643,-1.0611696079,1.4847576032
O,0,3.1734805691,-1.1136131607,-1.605423476
C,0,2.2573511522,2.3811788537,-0.4788140642
C,0,2.2975462975,-0.4913082713,2.5922186683
C,0,2.2053952724,-1.9347453582,-2.1241781672

	C,0,2.6861849231,3.6671019078,-0.3052190097
	C,0,2.9828117095,-0.8110426522,3.7308106176
	C,0,2.5852260017,-2.4653019568,-3.3247685071
	H,0,2.1520173921,4.565774383,-0.6094650169
	H,0,2.7150904569,-0.4977291007,4.738515579
	H,0,1.9905454815,-3.1333551322,-3.9446811079
	C,0,3.9530074759,3.5781694424,0.3604963627
	C,0,4.0847278587,-1.6289806545,3.3200887584
	C,0,3.8935897008,-1.9442229764,-3.5932022875
	C,0,4.8981687806,4.4947732839,0.8465820153
	C,0,5.1542913187,-2.2731919375,3.964293591
	C,0,4.8289183869,-2.0633873249,-4.6330073743
	H,0,4.7482136848,5.5694089537,0.7234657539
	H,0,5.2771691451,-2.2070101221,5.0470333389
	H,0,4.6236131825,-2.6950557944,-5.4997035986
	C,0,6.0190939957,3.9986630755,1.5008548307
	C,0,6.0505041998,-2.9942001102,3.187155478
	C,0,6.0143998319,-1.345755066,-4.5353748055
	H,0,6.7674303016,4.6933639069,1.8879252379
	H,0,6.8896499915,-3.5024921741,3.6661039866
	H,0,6.7564218253,-1.4221176833,-5.3327661843
	C,0,6.2157222897,2.6160220355,1.6776220902
	C,0,5.9046168013,-3.0843726746,1.7895261878
	C,0,6.2821230009,-0.5152705263,-3.4310151209
	H,0,7.108763799,2.2650403612,2.1987052891
	H,0,6.6330065341,-3.6580375577,1.2126784611
	H,0,7.2236585771,0.0364273916,-3.3930459658
	C,0,5.297457218,1.684101857,1.2006300359
	C,0,4.854439364,-2.459262681,1.1248684626
	C,0,5.373929478,-0.3819905678,-2.383841033
	H,0,5.4336883941,0.6083042491,1.3334435004
	H,0,4.7251258281,-2.5210549422,0.043609286
	H,0,5.5623948082,0.2599871358,-1.5214043245
	C,0,4.1830609637,2.2015825082,0.5500444305
	C,0,3.9635994942,-1.7472147638,1.9210005709
	C,0,4.1970914118,-1.1108985539,-2.4991110553
	O,0,0.4547106585,0.3726317272,-3.5100400403
	O,0,0.2645542595,3.032302983,2.2397688865
	O,0,0.6530391307,-2.9558912214,1.3778204454

	C,0,1.25210017,1.2487574002,-2.8099355625<br>C,0,1.1747336824,2.0923491918,2.6578655136<br>C,0,1.1270828371,-3.2287540493,0.1119508303<br>C,0,2.3769276883,1.5682602065,-3.5137283982<br>C,0,2.2884933621,2.6772980896,3.1912237084<br>C,0,2.0090458284,-4.2702550954,0.1291045563<br>H,0,3.1727229237,2.2274063077,-3.1721904433<br>H,0,3.1642278007,2.157171275,3.5740346994<br>H,0,2.5311466047,-4.6668446162,-0.7395500228<br>C,0,2.2945055622,0.8546950944,-4.7531640902<br>C,0,2.073239809,4.0913511316,3.1122698641<br>C,0,2.1172049502,-4.6899133241,1.4928989848<br>C,0,3.1056410236,0.7235355978,-5.8906856655<br>C,0,2.8107685819,5.2370075938,3.4492776744<br>C,0,2.8624524665,-5.6572124571,2.186424703<br>H,0,4.0548005211,1.2583389109,-5.9645146948<br>H,0,3.7950077165,5.1501573539,3.9141117255<br>H,0,3.5426009002,-6.3256540534,1.6548437253<br>C,0,2.6737762056,-0.1129015093,-6.9122677753<br>C,0,2.2600484837,6.4797211577,3.163358397<br>C,0,2.7180699148,-5.7331295106,3.5648291599<br>H,0,3.2890330784,-0.232057404,-7.8065191332<br>H,0,2.8157548897,7.3855256494,3.4142482702<br>H,0,3.2911739925,-6.4735049222,4.126311317<br>C,0,1.4571099695,-0.8137614836,-6.8220019337<br>C,0,0.9953720136,6.6008011028,2.5573301515<br>C,0,1.8472977244,-4.8726286698,4.2588474692<br>H,0,1.1501971574,-1.4607489044,-7.6464351701<br>H,0,0.5963603434,7.5950292461,2.346575203<br>H,0,1.7596337761,-4.9622784909,5.3435329578<br>C,0,0.6316387818,-0.6988791259,-5.7058164068<br>C,0,0.2395448094,5.4811404494,2.2207816842<br>C,0,1.0957485823,-3.9060549687,3.5973478908<br>H,0,-0.3126246339,-1.2403913752,-5.6228549953<br>H,0,-0.7406009585,5.5617522372,1.7478682825<br>H,0,0.4247886704,-3.2288904801,4.1280564212<br>C,0,1.0840873952,0.1377601772,-4.6929019265<br>C,0,0.8113335071,4.2476176442,2.5092805623<br>C,0,1.2605677875,-3.8385679436,2.2182315765

	O,0,-2.2088716391,0.9503876141,-2.4208973342
	O,0,-2.3843997805,1.6101413237,2.114122915
	O,0,-2.1095669765,-2.7274845471,0.3146359761
	C,0,-2.9174522596,0.9241467857,-1.245507615
	C,0,-3.0015521094,0.529916429,1.5325365913
	C,0,-2.8900902318,-1.7115146364,-0.1876347082
	C,0,-3.9179913704,1.8551925052,-1.2473477806
	C,0,-3.9884776549,0.0304434918,2.3344050749
	C,0,-3.9233227035,-2.2046239652,-0.9330107782
	H,0,-4.6031009508,2.0511165966,-0.4244151928
	H,0,-4.6102744482,-0.8343948552,2.1105516367
	H,0,-4.6679981875,-1.6075609175,-1.4563243048
	C,0,-3.8382687641,2.5289645198,-2.5098875678
	C,0,-3.9966235087,0.8424932225,3.5159783444
	C,0,-3.7925979815,-3.6322819133,-0.911901499
	C,0,-4.5204338223,3.5904601468,-3.1267106999
	C,0,-4.7112428405,0.8486306136,4.7247709902
	C,0,-4.4801530171,-4.7136645294,-1.4871650079
	H,0,-5.3522322174,4.0891542808,-2.625281386
	H,0,-5.4965151355,0.1143238268,4.9151390256
	H,0,-5.3621008788,-4.5503071008,-2.1096789097
	C,0,-4.0993466831,3.9963247042,-4.3857415651
	C,0,-4.3835155787,1.8059793154,5.6754742091
	C,0,-4.0008125378,-5.9949948846,-1.2505639098
	H,0,-4.6084510449,4.8258149882,-4.8806904747
	H,0,-4.9199635434,1.8242528254,6.6263173767
	H,0,-4.514701471,-6.8505464621,-1.6934685671
	C,0,-3.0232393208,3.367445708,-5.0398455699
	C,0,-3.3684622616,2.7533555608,5.4453603171
	C,0,-2.8597295374,-6.2197901525,-0.4570980994
	H,0,-2.7202952719,3.7190065625,-6.0283456448
	H,0,-3.1387399497,3.4885749564,6.219421484
	H,0,-2.5130977781,-7.2430707559,-0.2982246006
	C,0,-2.3291305172,2.3138209209,-4.4529270948
	C,0,-2.6438201697,2.7705777811,4.257253108
	C,0,-2.159444202,-5.1678392139,0.1254645333
	H,0,-1.4838811415,1.8258506804,-4.9398815666
	H,0,-1.8453457405,3.4892489324,4.0692771502
	H,0,-1.2665404494,-5.3214515749,0.7341149871

	C,0,-2.7610750862,1.9292185778,-3.1896870014
	C,0,-2.9815168185,1.7988204607,3.3235006755
	C,0,-2.652884521,-3.8942349698,-0.1281588641
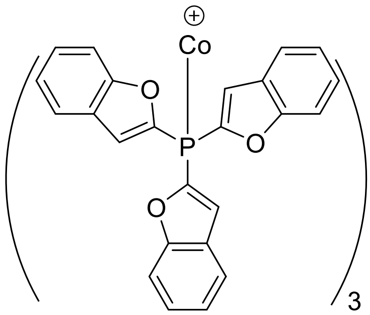	Co,0,0.6706507289,-0.6349263472,0.1711358298
	P,0,0.958567991,-2.0743735935,-1.4198459715
	P,0,1.0403895364,1.0925971366,-0.9855682109
	P,0,1.1529527677,0.2935275174,2.1039595982
	O,0,-0.0807140382,3.5212893903,-1.6020261483
	O,0,0.1045079795,0.0256869811,4.6354323765
	O,0,0.1895233537,-4.084708639,-3.1177908165
**[Co(L15)$_3$]-Cation-Singlet**	C,0,-0.2527316956,2.3436351797,-0.918444567
	C,0,-0.1595033243,-0.0239032625,3.2925469292
	C,0,-0.1761679754,-2.8991303232,-2.5311750521
	C,0,-1.4180584855,2.3414908259,-0.2089585246
	C,0,-1.4701344645,-0.3286871802,3.0499737665
	C,0,-1.4275738568,-2.5087185871,-2.9175776236
	H,0,-1.7614710937,1.5350517058,0.4352851961
	H,0,-1.9219501228,-0.4523018099,2.0668013063
	H,0,-1.9254855317,-1.5937566253,-2.6027372594
	C,0,-2.0306919169,3.6144642538,-0.4341061012
	C,0,-2.089028681,-0.4966904823,4.3300497078
	C,0,-1.8971026777,-3.5107867113,-3.8237939099
	C,0,-3.1920106263,4.2658945091,0.013127378
	C,0,-3.3738884191,-0.8282600207,4.791238356
	C,0,-3.0632652818,-3.7126253721,-4.5811976495
	H,0,-3.8894292606,3.7668488735,0.6886739056
	H,0,-4.1906638364,-1.011976217,4.0907023437
	H,0,-3.8894887207,-3.00030371,-4.5385019599
	C,0,-3.4251723132,5.5639052244,-0.4214373982
	C,0,-3.5755609847,-0.9168697669,6.1615418924
	C,0,-3.1358441409,-4.8433324813,-5.3824709865
	H,0,-4.3195515551,6.0918114428,-0.0852073047
	H,0,-4.565127558,-1.1727291401,6.5445496908
	H,0,-4.0314899822,-5.0237781728,-5.9797847967
	C,0,-2.5317424784,6.2180111993,-1.2907682674
	C,0,-2.5293612485,-0.6829854785,7.0737783415
	C,0,-2.0769233777,-5.7693725993,-5.4419182487
	H,0,-2.7508677061,7.2381948749,-1.6121478819
	H,0,-2.7283856916,-0.7605098706,8.1444445354

H,0,-2.1729328626,-6.6479105323,-6.0829875968
C,0,-1.3755225156,5.5951807848,-1.7511505324
C,0,-1.2472236638,-0.3543342002,6.6447727888
C,0,-0.9109357824,-5.5934738159,-4.7037929764
H,0,-0.674578813,6.0905473552,-2.4237278983
H,0,-0.4290530297,-0.1683438151,7.3410968443
H,0,-0.0841895549,-6.3033087816,-4.7408952038
C,0,-1.1562533314,4.2989689414,-1.3016513566
C,0,-1.0635071766,-0.2701011682,5.270018992
C,0,-0.8544781265,-4.4553220664,-3.908463338
O,0,3.5210104435,1.2336082671,0.0302846483
O,0,3.3216136029,-1.1984887316,1.7117452267
O,0,3.5174942929,-1.3870171669,-1.6459429289
C,0,2.5352895675,2.0115256416,-0.5278105156
C,0,2.6029617864,-0.590163988,2.7142081746
C,0,2.5004380472,-2.009162125,-2.3280091231
C,0,2.9030757938,3.3273692324,-0.5727342774
C,0,3.1401292298,-0.8555783774,3.9428995233
C,0,2.8487351197,-2.2764567896,-3.6229330181
H,0,2.299463581,4.1435281184,-0.963055295
H,0,2.7473997057,-0.5032155256,4.8938918807
H,0,2.2173682742,-2.7490217339,-4.3723859149
C,0,4.2141277717,3.3938048789,0.0014448566
C,0,4.2818665683,-1.6885765373,3.7073066183
C,0,4.1833675048,-1.7821858124,-3.7817365475
C,0,5.1309695708,4.4167357197,0.2925769292
C,0,5.2526947717,-2.3185704673,4.5048197786
C,0,5.1009411875,-1.7021127936,-4.8415852228
H,0,4.911274368,5.4546370589,0.03415614
H,0,5.2404207178,-2.2132775224,5.5913444925
H,0,4.8552318849,-2.1091207749,-5.8245887506
C,0,6.3142949317,4.0741474178,0.9336146599
C,0,6.2268058332,-3.0800638848,3.8735132363
C,0,6.3202532573,-1.0801731243,-4.6068822413
H,0,7.0418610486,4.8525457827,1.1721199364
H,0,6.9908700873,-3.5787791537,4.4729533445
H,0,7.0492365467,-1.001828872,-5.4157872454
C,0,6.5995487976,2.7416345517,1.2894697218
C,0,6.2592761818,-3.2217665314,2.4719490985

	C,0,6.6411599133,-0.5454575168,-3.3444699096
	H,0,7.538555427,2.514305194,1.7982299824
	H,0,7.0471622363,-3.8244988382,2.0154191572
	H,0,7.6087435796,-0.0605069136,-3.2006644173
	C,0,5.7110359369,1.7078132368,1.0086838922
	C,0,5.312064357,-2.6089254337,1.6578985323
	C,0,5.7526954991,-0.6173858293,-2.2753169581
	H,0,5.9126311234,0.6715664919,1.2884436675
	H,0,5.3161692343,-2.7028849091,0.5700796119
	H,0,5.9829748833,-0.2011130051,-1.2934754843
	C,0,4.5337088253,2.0711369493,0.3634444984
	C,0,4.3396644243,-1.859182823,2.3101848415
	C,0,4.5360797009,-1.2381173236,-2.5314366805
	O,0,0.3697574517,0.1740434264,-3.4335552762
	O,0,0.6164014597,2.9192987751,2.0368563887
	O,0,0.4966115821,-2.6375985282,1.0713801707
	C,0,1.3342835226,0.8811855742,-2.7573440906
	C,0,1.5771025123,2.0183136508,2.4166918384
	C,0,1.3165346884,-3.092203179,0.0111800943
	C,0,2.3862162152,1.1974192929,-3.5687571055
	C,0,2.6953070673,2.6425727929,2.8933660642
	C,0,2.2380160252,-3.9844418675,0.4618368225
	H,0,3.2818693348,1.73990257,-3.27119711
	H,0,3.5991268933,2.1543528564,3.2518816042
	H,0,2.9925848616,-4.4723245308,-0.1509620966
	C,0,2.0744432247,0.6513092961,-4.8567243616
	C,0,2.4349800467,4.0482582881,2.7925439607
	C,0,2.0161804432,-4.1365985078,1.868854679
	C,0,2.7301282658,0.5766466721,-6.0951831249
	C,0,3.1578526202,5.2218274948,3.0598031014
	C,0,2.6190556041,-4.9019748792,2.8798180741
	H,0,3.7066285594,1.0438663439,-6.2387753965
	H,0,4.1662218777,5.1741759872,3.4761382823
	H,0,3.4727283129,-5.5442472424,2.6550850172
	C,0,2.1098870275,-0.1199326352,-7.1249154356
	C,0,2.5603929781,6.4404967939,2.7654136196
	C,0,2.1033935435,-4.8179711001,4.166234599
	H,0,2.6006706133,-0.1931933633,-8.0975249954
	H,0,3.1035486929,7.367149184,2.9614547432

	H,0,2.5617480759,-5.3994232112,4.9684968536<br>C,0,0.8552890781,-0.7317703513,-6.9456433542<br>C,0,1.263898073,6.5114786864,2.2208179876<br>C,0,0.9873339516,-4.0139968436,4.4530417343<br>H,0,0.3979543406,-1.2675834253,-7.7798369839<br>H,0,0.8282486564,7.4890896579,2.0047398365<br>H,0,0.5842953378,-3.9935271254,5.4678449799<br>C,0,0.1801486402,-0.6674279091,-5.7290677379<br>C,0,0.5216288327,5.3645532021,1.9544890942<br>C,0,0.3678036346,-3.2477960072,3.4680521563<br>H,0,-0.7927904515,-1.1378516319,-5.5787315907<br>H,0,-0.4853914714,5.4034836164,1.5352107726<br>H,0,-0.5164849802,-2.6506302705,3.6827432177<br>C,0,0.821453263,0.0267684163,-4.7107775753<br>C,0,1.1406802536,4.1558349635,2.2493912581<br>C,0,0.9293212706,-3.3064153945,2.2003111672
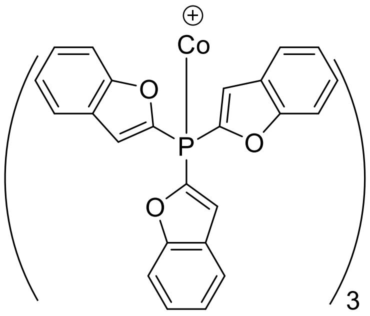<br>**[Co(L15)$_3$]-Cation-Triplet**	Co,0,0.0059149474,-0.0353454018,-0.0000258844<br>P,0,0.790463957,-1.950815491,-0.9648984677<br>P,0,0.832011049,1.7120198911,-1.180657237<br>P,0,0.865788523,0.2211318492,2.1012676828<br>O,0,0.2553827936,4.2521105239,-2.0585347761<br>O,0,0.0715401653,-0.0315894042,4.7152706318<br>O,0,-0.1041401864,-4.0674565988,-2.4538249061<br>C,0,-0.2071742308,3.16477473,-1.363311521<br>C,0,-0.2365941032,-0.3275443843,3.4127397472<br>C,0,-0.3526334382,-2.7711559152,-2.0836218697<br>C,0,-1.4717162773,3.3748255015,-0.8894347056<br>C,0,-1.3795132761,-1.0721256162,3.3260401392<br>C,0,-1.4636880684,-2.2758849899,-2.707351569<br>H,0,-2.0496714414,2.6736165983,-0.2899831756<br>H,0,-1.8166369738,-1.4592459052,2.4071033079<br>H,0,-1.8534721827,-1.2648710396,-2.6001586504<br>C,0,-1.8428906454,4.6947931481,-1.2973377831<br>C,0,-1.8337462952,-1.2748623194,4.6681651293<br>C,0,-1.9581836073,-3.3259441722,-3.5451521156<br>C,0,-2.9617515877,5.5252350877,-1.1191584411<br>C,0,-2.9065427835,-1.9468288155,5.2769692252<br>C,0,-3.0283923523,-3.4786420453,-4.4425804805<br>H,0,-3.8392599472,5.1749246495,-0.5719856341

H,0,-3.6487603203,-2.4767887512,4.6771114608
H,0,-3.7308175578,-2.6622729701,-4.6223062476
C,0,-2.9189781091,6.8052029437,-1.6547226979
C,0,-2.9958713335,-1.91900059,6.6618508796
C,0,-3.1687531203,-4.6968029999,-5.0930352936
H,0,-3.7753846255,7.4702617604,-1.5291957562
H,0,-3.8203871236,-2.4337884782,7.1584751138
H,0,-3.9922750982,-4.8401577974,-5.7950438244
C,0,-1.7922371883,7.2674917455,-2.3607064658
C,0,-2.0445506115,-1.2384670754,7.4447602684
C,0,-2.2718716436,-5.7577903889,-4.8659574202
H,0,-1.7972655266,8.2803336277,-2.7682387726
H,0,-2.15045135,-1.2377689895,8.5313834621
H,0,-2.4175677505,-6.7013717719,-5.3954495564
C,0,-0.671912015,6.4650666274,-2.5529464591
C,0,-0.9714383762,-0.5664035182,6.8678318375
C,0,-1.2044324753,-5.6337201395,-3.9824346696
H,0,0.2069858017,6.8109500646,-3.0976495518
H,0,-0.228196649,-0.0349763508,7.4628138864
H,0,-0.5031802361,-6.4475335073,-3.7961133383
C,0,-0.7315176049,5.188028854,-2.0090615266
C,0,-0.8962161866,-0.6072176458,5.4811520346
C,0,-1.0766116224,-4.4064383917,-3.3436746309
O,0,3.2148543779,1.4332635965,-0.0041707737
O,0,3.067180022,-1.1777671088,1.4655078383
O,0,3.3136735975,-1.1436977691,-1.4847614087
C,0,2.3638903282,2.3845109462,-0.5113361361
C,0,2.4343330722,-0.5667661148,2.5154378261
C,0,2.2796865076,-1.8916912547,-1.9811736857
C,0,2.8392444666,3.6483987006,-0.29968414
C,0,3.0997525889,-0.7758811459,3.6916691673
C,0,2.5763649943,-2.4084329802,-3.212509047
H,0,2.3494210289,4.5745453123,-0.5915297116
H,0,2.7899405759,-0.4112248211,4.6686891774
H,0,1.9239798075,-3.0290521289,-3.8227760731
C,0,4.0835373219,3.4912523075,0.3936251861
C,0,4.2449676475,-1.5718738251,3.3640802511
C,0,3.901251087,-1.9591854344,-3.5211563997
C,0,5.043923389,4.361022405,0.9340747082

C,0,5.322859575,-2.1254706471,4.0753104916
C,0,4.7870449366,-2.1119699343,-4.5995076399
H,0,4.9343614233,5.4431845777,0.8369036471
H,0,5.4146546756,-1.9830250785,5.1537161504
H,0,4.5108378241,-2.7125343126,-5.4685103523
C,0,6.1264712379,3.8073927708,1.6056679313
C,0,6.268020969,-2.8572350889,3.3693795463
C,0,6.0153940921,-1.4672720112,-4.535242472
H,0,6.8854604016,4.4633876674,2.036584636
H,0,7.1143241489,-3.2966315966,3.9008750025
H,0,6.7209777161,-1.5697806841,-5.3621634032
C,0,6.2719499272,2.4135016632,1.7415628353
C,0,6.1635049509,-3.0456877967,1.9775458967
C,0,6.3745110997,-0.6789329625,-3.4253520881
H,0,7.1375838803,2.0158166405,2.2751928994
H,0,6.9300149022,-3.6250028878,1.4586949986
H,0,7.3482077526,-0.1849589245,-3.4140619583
C,0,5.337368252,1.5285627688,1.2113307863
C,0,5.106802851,-2.5106414545,1.2478595726
C,0,5.5190019804,-0.518071547,-2.3388593347
H,0,5.4368637601,0.4459881274,1.3136618924
H,0,5.0090778473,-2.6473045912,0.1698283259
H,0,5.7840306341,0.087407216,-1.4702468941
C,0,4.257324926,2.1031316883,0.5504898608
C,0,4.167468242,-1.7866788296,1.9740921497
C,0,4.2960677142,-1.1732022254,-2.421690866
O,0,0.4075009018,0.4091851192,-3.4856951991
O,0,0.191743277,2.7976092182,1.9668302024
O,0,0.6835318137,-2.9136798621,1.5371039334
C,0,1.308964902,1.2336766345,-2.8507038272
C,0,1.1673664668,1.9618289289,2.4648978125
C,0,1.2260636567,-3.1630500985,0.296576629
C,0,2.4283550517,1.4306498045,-3.6059064726
C,0,2.1991270215,2.6676219366,3.0128262309
C,0,2.1585431216,-4.1577064132,0.3519574806
H,0,3.2909032466,2.0304228416,-3.32123111
H,0,3.1007086023,2.2461967976,3.4535953791
H,0,2.7387479822,-4.5275635883,-0.4914856326
C,0,2.2294145799,0.6882965393,-4.8149170888

	C,0,1.8664841059,4.0522379913,2.8500014948<br>C,0,2.2350621531,-4.5628318511,1.7231928689<br>C,0,2.9800994749,0.4518492287,-5.9767667747<br>C,0,2.5007219755,5.27090376,3.1373923122<br>C,0,3.0116855927,-5.4748252445,2.4565124529<br>H,0,3.9646462864,0.9056471084,-6.1073160196<br>H,0,3.4693965392,5.2895133283,3.6412904555<br>H,0,3.7490370098,-6.1080308388,1.9591555166<br>C,0,2.4425368109,-0.3851113951,-6.9458831926<br>C,0,1.8694245522,6.4470145842,2.7523497808<br>C,0,2.8244163191,-5.5399100359,3.8303830002<br>H,0,3.0078381832,-0.5849941101,-7.8584722869<br>H,0,2.3447765437,7.4072701522,2.9623120442<br>H,0,3.4213356818,-6.2366044913,4.4220654893<br>C,0,1.1800554072,-0.9843309619,-6.7795706107<br>C,0,0.6213444764,6.4300114117,2.1021476731<br>C,0,1.8802090374,-4.7246268347,4.4821877672<br>H,0,0.7905454507,-1.6364334172,-7.5639873963<br>H,0,0.1520776483,7.3744228963,1.8182133926<br>H,0,1.7612424632,-4.805004167,5.5645448966<br>C,0,0.4110833361,-0.7603596119,-5.6403237112<br>C,0,-0.0342163281,5.2355198909,1.8160960612<br>C,0,1.0943632615,-3.8153534815,3.7804710343<br>H,0,-0.5694564142,-1.2185860511,-5.5015496132<br>H,0,-1.0040927039,5.2136336303,1.317724494<br>H,0,0.361596855,-3.175711124,4.2756797934<br>C,0,0.9666651933,0.0787087755,-4.6823156396<br>C,0,0.6206864571,4.0700752595,2.1951956283<br>C,0,1.3041301589,-3.7575006294,2.4075429794
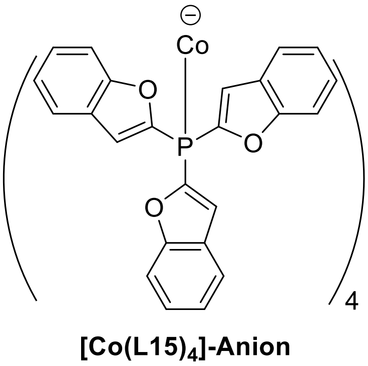<br>**[Co(L15)$_4$]-Anion**	Co,0,-0.0038606908,0.0083877108,-0.2246466591<br>P,0,0.3373634296,1.9328366649,0.5227835595<br>P,0,-1.836128769,-0.6860495859,0.5100953618<br>P,0,1.516144407,-1.2544098693,0.4629053095<br>P,0,-0.0307382689,0.0317791491,-2.3060281021<br>O,0,0.5626385927,4.5924568282,-0.3580841311<br>O,0,-4.2583572041,-1.7774177156,-0.4011530358<br>O,0,3.6830248045,-2.7588547408,-0.5047359616<br>C,0,-0.117647137,3.3984796196,-0.4775724033<br>C,0,-2.8842731987,-1.7781327909,-0.5231734769

	C,0,2.9801773816,-1.5749564722,-0.5918070516
	C,0,-1.1532240737,3.5064089347,-1.3602198096
	C,0,-2.4703952012,-2.70733484,-1.4334023304
	C,0,3.5604618626,-0.720806288,-1.4844338025
	H,0,-1.8251306348,2.6906154115,-1.6221824065
	H,0,-1.4300310807,-2.8854875199,-1.7010184827
	H,0,3.1789071017,0.2704260107,-1.7243397082
	C,0,-1.1452066421,4.8561490094,-1.8343969721
	C,0,-3.6504741459,-3.3519275985,-1.9228953994
	C,0,4.7143419703,-1.392219468,-1.9993004532
	C,0,-1.9087966749,5.6118313305,-2.7377901936
	C,0,-3.9346689554,-4.3651127381,-2.8519397635
	C,0,5.723840308,-1.0942508564,-2.927929004
	H,0,-2.7568607809,5.1618270896,-3.2607829304
	H,0,-3.126801134,-4.8681908547,-3.3899923339
	H,0,5.7396152644,-0.1288000863,-3.440754846
	C,0,-1.5574204642,6.9395410072,-2.9570609113
	C,0,-5.2639678219,-4.7080020257,-3.0759355943
	C,0,6.6959172541,-2.055323869,-3.1846160105
	H,0,-2.1412286759,7.5435586995,-3.656412473
	H,0,-5.5039240959,-5.4955437471,-3.7949126701
	H,0,7.4900280861,-1.8402903836,-3.9042535678
	C,0,-0.4606813979,7.5234470925,-2.2989013021
	C,0,-6.3105099448,-4.0582477913,-2.3982857495
	C,0,6.6774317822,-3.3042029585,-2.5389335862
	H,0,-0.2100682785,8.5685751616,-2.4968835216
	H,0,-7.3440573548,-4.3498430756,-2.6008993443
	H,0,7.4550309141,-4.0376384879,-2.7662865098
	C,0,0.316816671,6.7935681567,-1.4018750652
	C,0,-6.0555858831,-3.0455001084,-1.47569469
	C,0,5.6838585035,-3.6267217053,-1.6166937355
	H,0,1.1788712821,7.2253145502,-0.8908099317
	H,0,-6.8544867131,-2.5217442899,-0.9482878141
	H,0,5.6461787405,-4.594097161,-1.1132483939
	C,0,-0.0519455489,5.4713272635,-1.1889803993
	C,0,-4.7226784714,-2.7213721563,-1.2576061914
	C,0,4.7251536128,-2.653062018,-1.3665734251
	O,0,-0.5188818314,1.6062343659,3.0959554019
	O,0,-1.1012973196,-1.3634050761,3.0525196175

	O,0,1.7673849106,-0.4178000887,3.0574665676<br>C,0,-0.520337337,2.4960001263,2.0500434915<br>C,0,-1.8889375762,-1.763982539,2.0015143819<br>C,0,2.492118368,-0.8244846041,1.963871001<br>C,0,-1.2935089592,3.5876968504,2.3417452323<br>C,0,-2.4617360277,-2.9805260587,2.2604514174<br>C,0,3.8355333011,-0.7001479814,2.1980246375<br>H,0,-1.4710347177,4.4346499593,1.6819614518<br>H,0,-3.1201598479,-3.5290626797,1.5898633554<br>H,0,4.6296205899,-0.9417459739,1.4946713473<br>C,0,-1.8139460317,3.38052975,3.6597867048<br>C,0,-2.0127838351,-3.3763523234,3.5614963622<br>C,0,3.9739396682,-0.1843284048,3.5263101244<br>C,0,-2.6570461425,4.0799620217,4.5362381505<br>C,0,-2.2026184385,-4.4812246862,4.4050511975<br>C,0,5.0397579974,0.1726298983,4.3655966285<br>H,0,-3.0771617707,5.0472881572,4.2502715418<br>H,0,-2.8476307697,-5.3090176474,4.1005432642<br>H,0,6.073345192,0.0647796429,4.0279010571<br>C,0,-2.9674455142,3.501369853,5.7629137901<br>C,0,-1.5298419604,-4.5109409314,5.6224757488<br>C,0,4.7501433193,0.6901616391,5.6240604021<br>H,0,-3.6277169541,4.0297369786,6.4555867214<br>H,0,-1.6616288186,-5.366882609,6.289510797<br>H,0,5.5681985452,0.9782332927,6.2894178902<br>C,0,-2.4640644587,2.2397100608,6.1259269879<br>C,0,-0.6647142066,-3.4710605186,6.006918412<br>C,0,3.4234060244,0.8694089196,6.0536689667<br>H,0,-2.743273859,1.8060578515,7.0892469068<br>H,0,-0.1353106634,-3.5365025237,6.9605677898<br>H,0,3.2305265067,1.2966646728,7.0406880378<br>C,0,-1.6187773545,1.5269197589,5.2762668224<br>C,0,-0.4633174983,-2.3591116686,5.1899384401<br>C,0,2.3461464951,0.5160682414,5.2416824823<br>H,0,-1.2371062254,0.5356329695,5.5283805644<br>H,0,0.2251697605,-1.5542189278,5.455182566<br>H,0,1.3101241726,0.6685235463,5.5506702347<br>C,0,-1.3063109193,2.1283252135,4.0632591938<br>C,0,-1.1598645855,-2.3375959086,3.9879497371

	C,0,2.6550344817,-0.0174945012,3.9961376699
	O,0,3.0408548595,2.125229183,0.1659689768
	O,0,-3.3516370898,1.5725686728,0.2443967204
	O,0,0.2931251207,-3.6725539264,0.1172804865
	C,0,2.027390939,2.4155468714,1.0506297323
	C,0,-3.1012387047,0.5124613889,1.0852041623
	C,0,1.1072365369,-2.968527489,0.9751369893
	C,0,2.5349755695,2.8738512159,2.2350669549
	C,0,-3.7628459036,0.6681960327,2.2719788484
	C,0,1.3150663207,-3.6701635829,2.1304953016
	H,0,1.9483267584,3.1644834738,3.1041070398
	H,0,-3.7253853259,-0.0229141884,3.1116411066
	H,0,1.9132625639,-3.3342416729,2.9750269483
	C,0,3.9600113332,2.8880162285,2.0947696321
	C,0,-4.4869173874,1.9003669974,2.1810715825
	C,0,0.5917410427,-4.8995642237,2.0019117331
	C,0,5.0518883497,3.2316963653,2.9050981126
	C,0,-5.3380870622,2.6368598889,3.0179310846
	C,0,0.3744292703,-6.0328254294,2.7991427745
	H,0,4.8951864707,3.5981083628,3.9224808236
	H,0,-5.5857533109,2.2723968451,4.0177829085
	H,0,0.8252563829,-6.1086943859,3.7915723321
	C,0,6.3358378726,3.070982014,2.3932780541
	C,0,-5.8390268622,3.8497030381,2.5548190552
	C,0,-0.4464838674,-7.0432503853,2.3078312431
	H,0,7.1985350987,3.3267240524,3.0140417437
	H,0,-6.4991706806,4.4395183104,3.1961013858
	H,0,-0.6323984806,-7.9304426304,2.9187665149
	C,0,6.5500299837,2.5733420067,1.096247859
	C,0,-5.5052861519,4.3412605389,1.2808467849
	C,0,-1.0532318657,-6.9442043382,1.0436112786
	H,0,7.5718397191,2.4490603654,0.7296067572
	H,0,-5.9079196533,5.3026039262,0.9527034502
	H,0,-1.6992600297,-7.7523775506,0.6921901483
	C,0,5.4814971998,2.2334926527,0.26641288
	C,0,-4.6675648213,3.6250524956,0.4260902104
	C,0,-0.8471105416,-5.8320027059,0.2276292644
	H,0,5.6283602902,1.8381894192,-0.7407955235
	H,0,-4.3908695946,3.9945772753,-0.5634346307

H,0,-1.3154543017,-5.7344732842,-0.7538317797
C,0,4.2080856176,2.4054160336,0.793947346
C,0,-4.1823931097,2.4144785414,0.9043676348
C,0,-0.0233237288,-4.8353688131,0.7351181873
O,0,1.7336119366,2.0528576024,-2.6319533902
O,0,-2.6651567325,0.5620376513,-2.5991552602
O,0,0.8290766112,-2.504889768,-2.6773800688
C,0,0.6059999415,1.5042940569,-3.1965409678
C,0,-1.6298178041,-0.1229527485,-3.1914084126
C,0,0.8978922662,-1.2490564492,-3.2347013631
C,0,0.0748602388,2.3523924562,-4.1300446935
C,0,-2.1033501492,-0.9740556997,-4.1522565049
C,0,1.8698154202,-1.2023378081,-4.1968529493
H,0,-0.8301767007,2.1642702322,-4.7048371762
H,0,-1.4913969444,-1.6462558512,-4.7509567447
H,0,2.1405536222,-0.3185867338,-4.7717936543
C,0,0.9046844341,3.5214940971,-4.146668075
C,0,-3.5302336855,-0.8349655344,-4.1608968773
C,0,2.4694990177,-2.5036878462,-4.2423529864
C,0,0.9040927367,4.7569169391,-4.8133770337
C,0,-4.602509859,-1.4269607333,-4.8464729468
C,0,3.5221225424,-3.1113113357,-4.9447186527
H,0,0.1275164375,4.9970998848,-5.5436391685
H,0,-4.4256670272,-2.1967856317,-5.6015239027
H,0,4.0963533233,-2.5500268059,-5.6858380209
C,0,1.9015904674,5.6766541162,-4.5080390423
C,0,-5.8958960622,-1.0278952655,-4.5273158959
C,0,3.8311813639,-4.4373372417,-4.6614015624
H,0,1.906242788,6.6490110533,-5.0071097718
H,0,-6.7426207387,-1.4909342438,-5.040360024
H,0,4.658113548,-4.9202491717,-5.1882038577
C,0,2.8942555029,5.3929257769,-3.5535836421
C,0,-6.1416862575,-0.0548296332,-3.5426671225
C,0,3.1162326306,-5.1671768858,-3.6955830966
H,0,3.6536276163,6.1458394938,-3.3286669887
H,0,-7.1720245265,0.2243421503,-3.3090518098
H,0,3.3960766956,-6.2032396227,-3.4897775441
C,0,2.9161639721,4.1774259154,-2.8724473462
C,0,-5.0970101297,0.5464932736,-2.8435592534

	C,0,2.0694668926,-4.5885610896,-2.9805114338<br>H,0,3.6595972875,3.9445771791,-2.1082350574<br>H,0,-5.2638232321,1.285002416,-2.0576373112<br>H,0,1.5166049904,-5.1264359217,-2.2084714164<br>C,0,1.9109459524,3.2738157216,-3.1916667258<br>C,0,-3.8138028727,0.1307083864,-3.1745690086<br>C,0,1.7801369124,-3.2624991226,-3.27550259
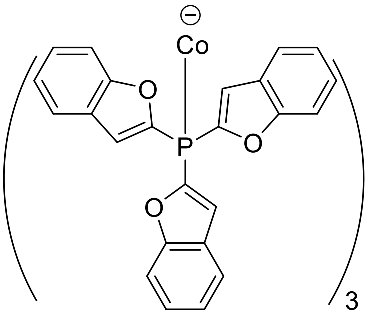<br>**[Co(L15)$_3$]-Anion**	Co,0,0.1751364608,-0.0166127476,-0.0341600794<br>P,0,0.6925713518,-1.8492512039,-0.7927926646<br>P,0,0.8096618454,1.5367225445,-1.2172174192<br>P,0,0.6911755464,0.221386283,1.9425916579<br>O,0,0.1585274448,3.9528636956,-2.5218515154<br>O,0,-0.2075244045,0.0818769462,4.6194511012<br>O,0,-0.3338208359,-4.1524976504,-2.0798407976<br>C,0,-0.1256518048,3.0981647691,-1.4765779451<br>C,0,-0.3700716005,-0.3703957994,3.3244043595<br>C,0,-0.4247072972,-2.7866045132,-1.9109210412<br>C,0,-1.1335383516,3.5999412808,-0.7009056726<br>C,0,-1.3801902103,-1.2886401233,3.2577395566<br>C,0,-1.3951385644,-2.2613149732,-2.7190273331<br>H,0,-1.5214916359,3.1074727973,0.1885659495<br>H,0,-1.6812731898,-1.7947672391,2.3431897131<br>H,0,-1.6372244407,-1.2014864312,-2.7702441621<br>C,0,-1.5060514938,4.8595096466,-1.2687584594<br>C,0,-1.8846680469,-1.4509091321,4.5866610868<br>C,0,-1.9471825395,-3.3498040081,-3.4654975462<br>C,0,-2.4318596155,5.872712148,-0.9715757938<br>C,0,-2.8754533356,-2.2283100711,5.2077356825<br>C,0,-2.9327077005,-3.5062357304,-4.4537112201<br>H,0,-3.0932695976,5.786318071,-0.1058850587<br>H,0,-3.4842998215,-2.9227981397,4.6240830035<br>H,0,-3.4882471899,-2.6423245915,-4.8268239835<br>C,0,-2.4893684182,6.9878434923,-1.8009481823<br>C,0,-3.0625672586,-2.0979865925,6.5801340591<br>C,0,-3.185017376,-4.7820069324,-4.9478062696<br>H,0,-3.2023513416,7.7863780002,-1.5809929714<br>H,0,-3.8280297759,-2.6980730697,7.0784742715<br>H,0,-3.9466228463,-4.9196772642,-5.7194553299<br>C,0,-1.6470610706,7.1112971814,-2.919911072

C,0,-2.2853470327,-1.2093528411,7.3433025274
C,0,-2.4793586623,-5.9023704176,-4.4753627036
H,0,-1.7171133788,8.0016869851,-3.5494154619
H,0,-2.4576896111,-1.1325290162,8.4195770086
H,0,-2.7013512707,-6.8900211255,-4.8867611236
C,0,-0.7216476713,6.1192596924,-3.2398809302
C,0,-1.2955895488,-0.4252570259,6.7519125505
C,0,-1.4977605332,-5.775689694,-3.4931960991
H,0,-0.0589058223,6.1958175459,-4.1032505042
H,0,-0.6820823193,0.2703360932,7.3267692234
H,0,-0.9378401512,-6.6321334877,-3.1142679254
C,0,-0.6730849954,5.0151559084,-2.3977726738
C,0,-1.1181469018,-0.5713931911,5.3818978712
C,0,-1.2530428641,-4.494270633,-3.0157304412
O,0,3.3299486109,1.4590864476,-0.2000370016
O,0,3.0265095583,-1.1013574003,1.5035708935
O,0,3.2972905999,-1.3540147083,-1.4547512152
C,0,2.3527043569,2.3316838364,-0.6161682444
C,0,2.2726672795,-0.5474746193,2.504014506
C,0,2.1441466748,-1.9332500774,-1.9236832046
C,0,2.6926193945,3.6227978192,-0.308739824
C,0,2.8756129591,-0.7281513274,3.7201314535
C,0,2.3119073899,-2.3722866324,-3.2111130691
H,0,2.0882789355,4.5054941771,-0.5119487379
H,0,2.4792439709,-0.3811989638,4.6720810117
H,0,1.5547976457,-2.862200349,-3.8202764442
C,0,3.9638368268,3.5652674393,0.3490822595
C,0,4.0960674666,-1.4370194751,3.4718459757
C,0,3.6597461936,-2.0575789279,-3.5799381845
C,0,4.8442885118,4.4880140862,0.9337626866
C,0,5.1641241437,-1.9270430015,4.2409529617
C,0,4.4665415196,-2.2261895208,-4.7151947264
H,0,4.614134849,5.5563450312,0.9267452627
H,0,5.1756371255,-1.7983374116,5.3260049156
H,0,4.0708292744,-2.710279569,-5.6113801219
C,0,6.0019358208,4.009679919,1.5427300137
C,0,6.2054382282,-2.5810956174,3.5915666105
C,0,5.7708190214,-1.7407610077,-4.6823903252
H,0,6.6955213833,4.7149511596,2.0083589152

H,0,7.0439910958,-2.9691693426,4.1754162042
H,0,6.41072053,-1.8590312606,-5.5608895224
C,0,6.2975783042,2.635871767,1.5788688912
C,0,6.2036941825,-2.7591485408,2.1956199174
C,0,6.2813841551,-1.0859665239,-3.5480508219
H,0,7.2082284911,2.2932459735,2.0758932581
H,0,7.0377841555,-3.2809513669,1.7200460588
H,0,7.3052884315,-0.7046105965,-3.5617810025
C,0,5.4451047168,1.6977835192,0.9962972317
C,0,5.1563635605,-2.2879721453,1.4079570446
C,0,5.5032985327,-0.912742205,-2.4029616747
H,0,5.6474217743,0.6249313808,1.0292005423
H,0,5.1248490402,-2.4211737714,0.325072609
H,0,5.8743647519,-0.3986718553,-1.5139945404
C,0,4.2972205038,2.1953014794,0.3919989016
C,0,4.1255043837,-1.6368740248,2.0759444505
C,0,4.2097016381,-1.4158682521,-2.4510473659
O,0,0.4113663902,0.6002362247,-3.7387398115
O,0,0.0138506776,2.8061078494,2.3823880258
O,0,0.7478841096,-3.0888301819,1.6187456017
C,0,1.3432910958,1.236166165,-2.9465077832
C,0,1.0468683036,1.9104424691,2.5774375741
C,0,1.2627396411,-3.1816199221,0.3452941012
C,0,2.5375203047,1.3526744999,-3.6023654136
C,0,2.1812913094,2.5676312538,2.9729839278
C,0,2.2897146566,-4.0845086248,0.3101587693
H,0,3.4394907893,1.8058076825,-3.1956137969
H,0,3.1449410783,2.1060315046,3.1791014555
H,0,2.8787558951,-4.326715706,-0.5728514177
C,0,2.3554376148,0.7672877235,-4.8967784732
C,0,1.8571621191,3.9619735121,3.0211694072
C,0,2.4549273718,-4.5801068781,1.6441534499
C,0,3.1511725167,0.560855221,-6.0330988749
C,0,2.5451258504,5.1488864798,3.3151893617
C,0,3.332099943,-5.4577565152,2.3007685691
H,0,4.1923093444,0.8921305433,-6.0489782846
H,0,3.5958491559,5.1202815662,3.6137815449
H,0,4.1200610905,-5.9755101422,1.7481513792
C,0,2.5934104107,-0.0961316133,-7.1261501306

	C,0,1.8657932397,6.3591906964,3.1984673545<br>C,0,3.1897708404,-5.637182032,3.6727785992<br>H,0,3.2022996374,-0.2709901607,-8.0171359944<br>H,0,2.3898104308,7.2937890232,3.4152739386<br>H,0,3.8721988806,-6.3082473438,4.2005859736<br>C,0,1.264833003,-0.5535822588,-7.1051384476<br>C,0,0.5183423339,6.406840837,2.8034879825<br>C,0,2.1946245753,-4.9622037307,4.4019220446<br>H,0,0.8616817089,-1.0767350948,-7.9757306662<br>H,0,0.0169265582,7.3732469301,2.711185349<br>H,0,2.1200609299,-5.117760037,5.4808583463<br>C,0,0.4488572211,-0.3517676688,-5.9912771349<br>C,0,-0.1918184164,5.2392207235,2.5216930434<br>C,0,1.3099335857,-4.0858732685,3.7765922901<br>H,0,-0.5838146851,-0.7038981553,-5.9541840131<br>H,0,-1.2374492496,5.2563672245,2.2097936296<br>H,0,0.5424912084,-3.5357091975,4.3252093369<br>C,0,1.0206809728,0.31238613,-4.9143528169<br>C,0,0.5037018018,4.0434432266,2.6379594494<br>C,0,1.4701936439,-3.9185029318,2.4065607639
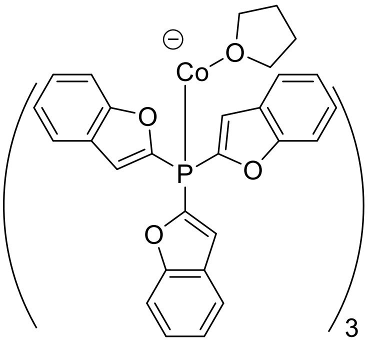<br>**[Co(L15)$_3$(THF)] - Anion**	Co,0,0.093422475,0.2563709519,0.1002001193<br>P,0,0.2572831418,-0.7435480924,1.8948215251<br>P,0,-1.7992351562,0.1615559912,-0.739884667<br>P,0,1.5543832901,-0.4028508133,-1.1894063044<br>O,0,0.4104984265,2.3073813305,0.4243496534<br>C,0,1.1611093113,2.8781330383,1.4943116969<br>H,0,2.2439342361,2.805266225,1.2735358812<br>H,0,0.9578774286,2.2867033153,2.3978417576<br>C,0,-0.0057771237,3.3225636333,-0.4990364083<br>H,0,-1.1088309696,3.3002384499,-0.5489752179<br>H,0,0.3767624784,3.0732806439,-1.4991876234<br>C,0,0.5316511193,4.6281533106,0.0647798653<br>H,0,1.5151324387,4.8620938366,-0.3750848379<br>H,0,-0.1312391773,5.4803931424,-0.1388901198<br>C,0,0.6888390954,4.3130842469,1.5462318083<br>H,0,1.3876166016,4.9810499245,2.068307736<br>H,0,-0.2843615065,4.3704586162,2.0635828139<br>C,0,-2.978288798,1.29882562,0.0808896598<br>C,0,-2.7732794073,1.9238282661,1.2792659422

	H,0,-1.855889018,1.8346619309,1.8586142575<br>C,0,-3.9525488695,2.6814058942,1.5604680591<br>C,0,-4.8047099782,2.4602759953,0.4569861364<br>O,0,-4.2179423378,1.6265223644,-0.4369933469<br>C,0,-2.8983635191,-1.3057283038,-0.7210084306<br>C,0,-4.0600227792,-1.574595026,-0.0453635594<br>H,0,-4.6110131916,-0.8681088849,0.5710499172<br>C,0,-4.3959655374,-2.9352277166,-0.3315211575<br>C,0,-3.3684910101,-3.3997483395,-1.1801826838<br>O,0,-2.4646250801,-2.4166841943,-1.4040956606<br>C,0,-2.0737557663,0.6041805034,-2.5038969961<br>C,0,-2.9058355662,0.1759580378,-3.5007214062<br>H,0,-3.7072042325,-0.5505596396,-3.380724207<br>C,0,-2.5026701812,0.8534517969,-4.6977170098<br>C,0,-1.4176551867,1.6673490422,-4.3124243885<br>O,0,-1.1600338408,1.513554191,-2.9940760802<br>C,0,1.8567852421,-0.7048581711,2.8173490038<br>C,0,2.4410630628,-1.4354247145,3.8151479838<br>H,0,1.9678757812,-2.2584520968,4.3461689376<br>C,0,3.7652163454,-0.9199302922,3.987704642<br>C,0,3.8752475038,0.1270261355,3.0481681674<br>O,0,2.7251941993,0.2586786034,2.356129372<br>C,0,0.0224701771,-2.5590966628,1.9311548958<br>C,0,0.9113348206,-3.6015452038,1.9096143915<br>H,0,1.9879667945,-3.5266855951,2.0391709901<br>C,0,0.1473989728,-4.7911058752,1.6943159859<br>C,0,-1.1929515288,-4.3651524721,1.6066396598<br>O,0,-1.2636796351,-3.0126293522,1.7332692238<br>C,0,-0.8760270679,-0.2685978868,3.2605165311<br>C,0,-2.1597887312,-0.6270755946,3.5923113033<br>H,0,-2.6758817448,-1.510393412,3.2247461122<br>C,0,-2.664208364,0.3886844491,4.4644713094<br>C,0,-1.6163498552,1.3238548111,4.6022498554<br>O,0,-0.5407818741,0.928750598,3.87335469<br>C,0,2.598026015,-1.8581539155,-0.8040049332<br>C,0,2.5317971805,-3.1587157743,-1.2304620703<br>H,0,1.8971593809,-3.5237278174,-2.0341362174<br>C,0,3.4220474442,-3.9116301951,-0.4011303111<br>C,0,3.9834086956,-2.9761836988,0.4928669857

O,0,3.4729856205,-1.7403261274,0.2555283261
C,0,1.1251052644,-0.8574667116,-2.9091717134
C,0,1.459141962,-0.3672586768,-4.142826294
H,0,2.2336942136,0.3696169885,-4.3389473643
C,0,0.5736277533,-0.9845709002,-5.084545368
C,0,-0.2490075847,-1.8363896557,-4.3194418585
O,0,0.0862678875,-1.7566303061,-3.0094372523
C,0,2.8671415668,0.827727068,-1.5256037229
C,0,4.0705797151,1.0906701411,-0.9108380825
H,0,4.6156999963,0.3907009055,-0.2830048563
C,0,4.417300796,2.439621579,-1.2262009231
C,0,3.3680041567,2.917104007,-2.0419266538
O,0,2.4377853639,1.9473023871,-2.2217146558
C,0,-4.3943837507,3.5050799093,2.6073917375
H,0,-3.7621216606,3.6754080129,3.4827690817
C,0,-5.6584260805,4.0766008862,2.5104379677
H,0,-6.0233282636,4.7153376648,3.3187299328
C,0,-6.0678411009,3.0292895076,0.343122186
H,0,-6.6960912816,2.835512456,-0.5277905701
C,0,-6.4831000128,3.8448572392,1.3947789407
H,0,-7.4716227656,4.3090561416,1.3524977626
C,0,-2.9120729601,0.8756899916,-6.0400395178
H,0,-3.7411673151,0.2498605688,-6.3792544883
C,0,-2.2320128072,1.7002980297,-6.9303073763
H,0,-2.5364361029,1.7254543525,-7.9798453806
C,0,-1.147447738,2.4922130872,-6.5138353501
H,0,-0.6274482105,3.1178715723,-7.2435739417
C,0,-0.7183854359,2.4876630853,-5.1879048013
H,0,0.1308178609,3.0808301005,-4.8421890655
C,0,-5.4363286104,-3.816543664,0.00652318
H,0,-6.2498008736,-3.493560855,0.6608040218
C,0,-3.3241317179,-4.6855219623,-1.7012704491
H,0,-2.4950307745,-5.0095942922,-2.3323845392
C,0,-4.3689483801,-5.5405131106,-1.3509049487
H,0,-4.3708250753,-6.5657560738,-1.7286895733
C,0,-5.4094557776,-5.1095985441,-0.5105731875
H,0,-6.2120412663,-5.8069229145,-0.2565344862
C,0,0.4293542609,-6.1622822994,1.5879640762
H,0,1.459504056,-6.5219841463,1.6482860615

C,0,-0.632301821,-7.0451337246,1.408775655
H,0,-0.432211098,-8.1163735372,1.3214053994
C,0,-1.9592272453,-6.5889317335,1.3416554443
H,0,-2.772529557,-7.30521744,1.1992978161
C,0,-2.2642502543,-5.231188349,1.445571777
H,0,-3.2904481065,-4.8629095958,1.3894275662
C,0,4.901462592,-3.3015559455,1.4808431748
H,0,5.307521251,-2.5392772137,2.1491344369
C,0,5.257783733,-4.6483926279,1.5797849637
H,0,5.9712865332,-4.9576337033,2.3472930389
C,0,4.7119339519,-5.6089952514,0.7122068654
H,0,5.0113860707,-6.6547680156,0.8210243818
C,0,3.8003390951,-5.258181786,-0.2812038673
H,0,3.3777923718,-6.0161247516,-0.9453342071
C,0,0.3590922239,-0.9129278913,-6.4700252927
H,0,0.9646467575,-0.2512415809,-7.0943747971
C,0,-0.653695315,-1.6872858765,-7.027497847
H,0,-0.840216069,-1.6332871951,-8.1033654836
C,0,-1.4577577147,-2.5237717926,-6.2348634052
H,0,-2.2533760589,-3.1080736869,-6.7039305226
C,0,-1.2665076099,-2.6131293334,-4.856086119
H,0,-1.8921412852,-3.2369069115,-4.2160348426
C,0,5.4761539669,3.3149182729,-0.9251509644
H,0,6.3103491117,2.9856069721,-0.3001039991
C,0,5.4430755507,4.6095120783,-1.4407332555
H,0,6.2597069085,5.2990744092,-1.2110936899
C,0,3.3229016834,4.1981590197,-2.5711578173
H,0,2.4878452625,4.519122623,-3.1976008833
C,0,4.3857722907,5.0501464705,-2.25177685
H,0,4.3910017659,6.0714980533,-2.6397318144
C,0,4.8751033552,-1.2189156314,4.7945892304
H,0,4.828270256,-2.0246794529,5.5311804132
C,0,6.0375810826,-0.4730392052,4.6286495683
H,0,6.9124261041,-0.6951845192,5.2448616868
C,0,6.1145393405,0.5638939217,3.6806159245
H,0,7.04518284,1.126443384,3.5749551273
C,0,5.0268726866,0.8853338396,2.8713807985
H,0,5.0640767347,1.6842357022,2.128095487
C,0,-1.6993531441,2.4688568963,5.3807474382

	H,0,-0.8568948559,3.1588211916,5.4587786114
	C,0,-3.8676234881,0.633140804,5.1471058882
	H,0,-4.7070285505,-0.0605768699,5.0597817069
	C,0,-3.9719495262,1.7795524109,5.9319803295
	H,0,-4.9051703453,1.9818459998,6.4640067809
	C,0,-2.9068185816,2.6858954095,6.0522770423
	H,0,-3.0214725737,3.5751994511,6.676829501
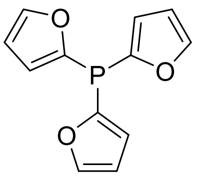	P,0,-1.1257490845,-0.0000000094,-0.0000000126
**L1-C1-Symm**	C,0,-0.239968002,-0.6425323225,-1.4483423097
	C,0,-0.2399680001,1.5755673684,0.1677218335
	C,0,-0.2399680067,-0.9330350792,1.2806204424
	O,0,-0.6952549881,-0.2468060629,-2.6723034945
	O,0,-0.6952549885,2.4376857187,1.1224114179
	O,0,-0.6952549983,-2.1908796864,1.5498920408
	C,0,0.065413425,-0.8594577245,-3.604809753
	C,0,0.0654134267,3.5515856572,1.0580926476
	C,0,0.0654134121,-2.6921279679,2.546717073
	C,0,0.997947887,-1.6606565166,-3.0122290316
	C,0,0.9979478924,3.4389950932,0.0679437831
	C,0,0.9979478778,-1.7783386173,2.9442852203
	C,0,0.8007226069,-1.5211115564,-1.6090100101
	C,0,0.8007226125,2.1539992937,-0.5128162479
	C,0,0.800722603,-0.6328877769,2.1218262289
	H,0,-0.1762276413,-3.7022327545,2.8635664152
	H,0,-0.176227624,-0.6288049103,-4.6380108307
	H,0,-0.1762276238,4.331037631,1.774444382
	H,0,1.7369858807,-1.9099246751,3.7306114012
	H,0,1.7369858909,-2.2758419372,-3.519348988
	H,0,1.7369858987,4.1857665673,-0.2112624381
	H,0,1.35623873,0.3026937129,2.1364889305
	H,0,1.3562387299,-2.0016005742,-0.8061040212
	H,0,1.356238738,1.6989068185,-1.3303849358
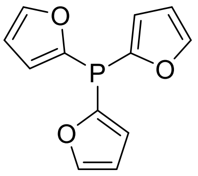	P,0,-1.1257490845,-0.0000000094,-0.0000000126
**L1-C3-Symm**	C,0,-0.239968002,-0.6425323225,-1.4483423097
	C,0,-0.2399680001,1.5755673684,0.1677218335
	C,0,-0.2399680067,-0.9330350792,1.2806204424
	O,0,-0.6952549881,-0.2468060629,-2.6723034945
	O,0,-0.6952549885,2.4376857187,1.1224114179

	O,0,-0.6952549983,-2.1908796864,1.5498920408
	C,0,0.065413425,-0.8594577245,-3.604809753
	C,0,0.0654134267,3.5515856572,1.0580926476
	C,0,0.0654134121,-2.6921279679,2.546717073
	C,0,0.997947887,-1.6606565166,-3.0122290316
	C,0,0.9979478924,3.4389950932,0.0679437831
	C,0,0.9979478778,-1.7783386173,2.9442852203
	C,0,0.8007226069,-1.5211115564,-1.6090100101
	C,0,0.8007226125,2.1539992937,-0.5128162479
	C,0,0.800722603,-0.6328877769,2.1218262289
	H,0,-0.1762276413,-3.7022327545,2.8635664152
	H,0,-0.176227624,-0.6288049103,-4.6380108307
	H,0,-0.1762276238,4.331037631,1.774444382
	H,0,1.7369858807,-1.9099246751,3.7306114012
	H,0,1.7369858909,-2.2758419372,-3.519348988
	H,0,1.7369858987,4.1857665673,-0.2112624381
	H,0,1.35623873,0.3026937129,2.1364889305
	H,0,1.3562387299,-2.0016005742,-0.8061040212
	H,0,1.356238738,1.6989068185,-1.3303849358
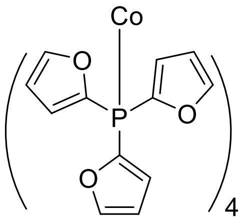 **[Co(L1)$_4$]-Radical**	Co,0,0.0059789657,-0.1292193884,0.1141606508
	P,0,0.72954511,1.5437017858,-1.1124856827
	P,0,0.9291791174,0.2816767557,2.0309526485
	P,0,0.8141589026,-1.8512859857,-0.9250145267
	P,0,-2.1310917394,0.0200038955,0.049426654
	O,0,-0.059067544,3.8704536314,-2.383444986
	O,0,0.1400368677,0.2814631049,4.6892123101
	O,0,0.1459602724,-4.0408055866,-2.4935979226
	C,0,-0.2789819949,3.0419697894,-1.3169254534
	C,0,-0.0963596226,-0.2280322641,3.4425830078
	C,0,-0.1570726277,-2.7423825255,-2.1791695689
	C,0,-1.2445415494,3.5828915738,-0.5100515382
	C,0,-1.1245358125,-1.1321220915,3.507694192
	C,0,-1.2140649055,-2.3247889661,-2.9436888024
	H,0,-1.6092816905,3.1404110958,0.4148833948
	H,0,-1.5184835768,-1.6972077279,2.6645848617
	H,0,-1.6577630328,-1.3321051365,-2.9059067158
	C,0,-1.6365916275,4.8109570844,-1.1137461993
	C,0,-1.5378208254,-1.1816487023,4.8683834403
	C,0,-1.5757064095,-3.4226786461,-3.7742683458

C,0,-0.8847035861,4.9290054983,-2.2479517643
C,0,-0.7369735906,-0.2985413169,5.5355140146
C,0,-0.7173068122,-4.4353826691,-3.4523837196
O,0,3.3519303938,1.5919186017,-0.4002783805
O,0,3.5756533295,-0.3936605013,1.7985878502
O,0,3.4825066644,-1.3606789373,-1.1538170105
C,0,2.2729736511,2.3864613934,-0.6354859074
C,0,2.4645739784,-0.548702211,2.5709149625
C,0,2.3961635899,-1.8415081625,-1.8181835892
C,0,2.6100361041,3.6960149207,-0.4045244232
C,0,2.7134121176,-1.4906451671,3.5378621189
C,0,2.7393281683,-2.1171763995,-3.1178874003
H,0,1.9481914045,4.5532954552,-0.5078728025
H,0,2.0088438407,-1.8255909393,4.2963163072
H,0,2.0742830222,-2.5117839344,-3.8828745309
C,0,3.9765171567,3.6948601717,-0.0033773376
C,0,4.0530561863,-1.9317964378,3.341684741
C,0,4.1164877929,-1.7803692526,-3.2511400321
C,0,4.3706772475,2.3878174243,-0.0188439164
C,0,4.5238296156,-1.2290060128,2.270743471
C,0,4.5106065146,-1.3275522042,-2.0252782826
O,0,0.4041094711,0.3191842919,-3.5315828329
O,0,0.4811949188,2.9596543775,2.1290474871
O,0,0.5199879845,-3.0581155876,1.4881155961
C,0,1.1976949378,1.1822725867,-2.8355448747
C,0,1.3686884875,1.9846069045,2.4783225056
C,0,1.1458888911,-3.177325307,0.2794072067
C,0,2.2530157946,1.5642562696,-3.6241699928
C,0,2.4580551248,2.5547638451,3.0846617387
C,0,1.9262717158,-4.3045928561,0.2865387881
H,0,3.0499862909,2.2442271383,-3.3298728407
H,0,3.3248722163,2.0186769648,3.4648400711
H,0,2.5365518508,-4.6521657234,-0.5442853171
C,0,2.086319714,0.9046499853,-4.8737579731
C,0,2.2179891581,3.9573203245,3.1111213075
C,0,1.762982911,-4.9068135408,1.5656225337
C,0,0.9459980246,0.1627100804,-4.7569196164
C,0,1.0028992163,4.1421130273,2.5172331253
C,0,0.8954474673,-4.1036101763,2.2502821314

	O,0,-2.3721168224,1.0059603986,-2.4581490436<br>O,0,-2.5404984326,1.6730890771,2.1657072242<br>O,0,-2.2542827487,-2.6597827522,0.3641776221<br>C,0,-2.9898189096,0.9630715659,-1.2434196924<br>C,0,-3.0773001427,0.6093924684,1.4985294501<br>C,0,-2.9364418931,-1.6015715695,-0.1645756144<br>C,0,-4.0817985246,1.7927675343,-1.2615014315<br>C,0,-4.147144695,0.1274775869,2.2099874097<br>C,0,-4.0038707006,-2.0714601258,-0.8865515702<br>H,0,-4.7560561516,1.9704970135,-0.4260180692<br>H,0,-4.7614487516,-0.7255007063,1.9282394691<br>H,0,-4.7183158117,-1.4579765082,-1.432243678<br>C,0,-4.1308276969,2.3711450999,-2.5622988902<br>C,0,-4.2714748441,0.9472706645,3.3682861868<br>C,0,-3.9683260047,-3.4929178866,-0.7903098593<br>C,0,-3.0657888611,1.856133902,-3.2435583858<br>C,0,-3.2670487168,1.867802596,3.2858748208<br>C,0,-2.8824741715,-3.7915013316,-0.0190666661<br>H,0,0.4755885981,-4.1322184803,3.2523363953<br>H,0,0.415400715,-0.5123145274,-5.4233208118<br>H,0,0.399643268,5.0188582547,2.297560746<br>H,0,2.2316750506,-5.8154404728,1.9359680387<br>H,0,2.7338510899,0.962744887,-5.7454335261<br>H,0,2.8664621535,4.732985223,3.5115535253<br>H,0,4.5972747113,-2.6761400089,3.9178239998<br>H,0,4.7351444616,-1.8613740143,-4.141655515<br>H,0,4.5863668419,4.5520238797,0.2719047194<br>H,0,5.4748977879,-1.2107830195,1.745920468<br>H,0,5.4541465659,-0.9621330291,-1.6296661108<br>H,0,5.3027077371,1.881439367,0.2163538172<br>H,0,-0.8352469383,5.6717780641,-3.0391331664<br>H,0,-0.6816614116,0.0271372971,6.5703731862<br>H,0,-0.6073003703,-5.4601304908,-3.7962331779<br>H,0,-2.3886729306,5.5124663187,-0.7603527587<br>H,0,-2.3374164602,-1.7816245474,5.2964175098<br>H,0,-2.3777547428,-3.4634083541,-4.5075322417<br>H,0,-2.6790344098,1.9895026565,-4.2501598173<br>H,0,-2.9472407051,2.6905082973,3.9190727107<br>H,0,-2.4375440812,-4.7192238108,0.331441076

	H,0,-4.8565402077,3.085546763,-2.9433546052
	H,0,-5.0042550339,0.8613069348,4.1670805645
	H,0,-4.6527202015,-4.205447976,-1.2444389939
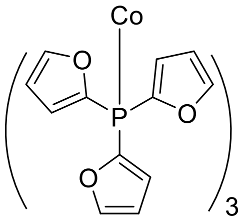 **[Co(L1)$_3$]-Radical**	Co,0,0.3911219493,0.5564434942,-0.0443181539
	P,0,1.7641269938,1.6937709932,-1.2681612948
	P,0,1.2053267488,0.4794631881,1.9342458008
	P,0,0.5302682669,-1.478871511,-0.8098612498
	O,0,-0.7963532296,2.0287675188,-2.4291467989
	O,0,0.9232711264,-0.5035618517,4.5236633476
	O,0,-0.6435112379,-3.3580452888,-2.463795965
	C,0,0.0322269631,2.1132755214,-1.3181259355
	C,0,0.3625316619,-0.3791373369,3.2818605624
	C,0,-0.4567758838,-2.0210473103,-2.2367212773
	C,0,-0.7338172738,2.3410955678,-0.1359736807
	C,0,-0.8813017587,-0.9544572477,3.2846582956
	C,0,-1.0594145598,-1.301185416,-3.2345684956
	H,0,-0.4320493993,2.9458029937,0.7224353557
	H,0,-1.5513367291,-0.9992425226,2.4282611083
	H,0,-1.0448753543,-0.217158401,-3.3179793591
	C,0,-2.1040444531,2.2826886134,-0.6074550619
	C,0,-1.0920493496,-1.4673334374,4.5943550463
	C,0,-1.64455282,-2.2476147823,-4.1221864242
	C,0,-2.069222995,2.0975997898,-1.9503058262
	C,0,0.0380209935,-1.1627735145,5.2982781989
	C,0,-1.3611021161,-3.4772978241,-3.6000107226
	O,0,4.0379395658,3.0271685155,-0.5968199487
	O,0,3.8328262069,0.2420660273,1.2752804663
	O,0,3.2002252387,-1.2115047902,-1.3308180182
	C,0,2.7120833001,3.1839877959,-0.8915003257
	C,0,2.8351512867,-0.3215479212,2.0132029073
	C,0,2.1761786262,-2.1075072463,-1.2709900892
	C,0,2.3351184781,4.4764438391,-0.6271294292
	C,0,3.2717945747,-1.5101534132,2.5396747004
	C,0,2.6251027039,-3.3346170775,-1.6925426766
	H,0,1.3344790781,4.8826156707,-0.7574364053
	H,0,2.6913079688,-2.1733863104,3.1768330221
	H,0,2.0207610594,-4.2370384389,-1.757149282
	C,0,3.4996045658,5.1507775593,-0.1628896565
	C,0,4.6162193259,-1.6784499077,2.0970401149

C,0,3.9982852367,-3.1742293084,-2.0249702061
C,0,4.5001676973,4.2230993311,-0.1692939248
C,0,4.902305202,-0.5807821487,1.3390188508
C,0,4.2871695013,-1.8598259131,-1.7875765619
O,0,1.7508181803,0.1776048765,-3.4811776048
O,0,0.616712629,2.9509320441,2.906678794
O,0,-1.2589344811,-2.9153335714,0.6434352134
C,0,2.3018401468,1.2967033688,-2.9358499817
C,0,1.6454669479,2.0774931773,2.6921766515
C,0,0.0700596344,-2.7963168962,0.3628355861
C,0,3.3016348227,1.7598457862,-3.753666112
C,0,2.8336428131,2.6926509663,2.9935674814
C,0,0.783865872,-3.6557399964,1.1582128056
H,0,3.9207935193,2.6346950967,-3.5688979403
H,0,3.8212400294,2.2477839459,2.9059171844
H,0,1.8642541921,-3.7800577963,1.1487392081
C,0,3.3510938125,0.8733395383,-4.8677456934
C,0,2.5099809157,4.0096952741,3.4287189368
C,0,-0.1676447724,-4.3433333515,1.9661426583
C,0,2.3842542804,-0.0656772722,-4.6478801783
C,0,1.1521322206,4.1087859241,3.3586688218
C,0,-1.3896726872,-3.8561570581,1.6086192962
H,0,-2.406220961,-4.070908075,1.9245141575
H,0,2.0301829204,-0.9296428867,-5.2036183483
H,0,0.4435463701,4.8985519557,3.589014752
H,0,0.0291270143,-5.1087679594,2.7128081315
H,0,4.0152628121,0.9273318005,-5.7268384944
H,0,3.1988762539,4.7865170494,3.7514895252
H,0,5.2888119318,-2.5028917902,2.3208588412
H,0,4.6847516074,-3.9310673462,-2.3964983241
H,0,3.5855224813,6.1922292013,0.1370127966
H,0,5.7858505225,-0.2466254602,0.8032508993
H,0,5.1858883931,-1.2584843777,-1.891837874
H,0,5.5547968681,4.2578487789,0.0876042913
H,0,-2.8521897552,2.0119939968,-2.6998422359
H,0,0.3481949103,-1.3413645911,6.3240440882
H,0,-1.6021191763,-4.4920371699,-3.9037466491
H,0,-3.0036539565,2.4103812091,-0.0088794406
H,0,-1.9653244369,-1.9953324195,4.9692327203

	H,0,-2.2102630344,-2.0456734711,-5.0285059708
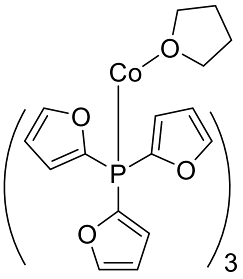 **[Co(L1)$_3$(THF)]-Radical**	Co,0,0.8509733058,0.1474455771,0.6841265282
	P,0,0.5656678516,-1.9745013486,0.9904846186
	P,0,2.9408160634,0.5699335316,0.9569441656
	P,0,-0.7504744439,1.5036906645,1.1147017395
	O,0,0.8372706012,0.3066720472,-1.4691280476
	C,0,-0.1927558472,-0.3417344151,-2.2104545491
	H,0,-1.1819480675,-0.0909113474,-1.7734617958
	H,0,-0.0510684171,-1.4258307036,-2.106383857
	C,0,1.2108172257,1.5405589516,-2.1005048673
	H,0,2.2903420814,1.4919208073,-2.3159429296
	H,0,1.0507358998,2.3674026609,-1.38931257
	C,0,0.3541406977,1.6415617039,-3.3527166964
	H,0,-0.5446056671,2.2453884292,-3.1531142523
	H,0,0.8842736024,2.1138524024,-4.1909161458
	C,0,-0.0348260361,0.1924019005,-3.6157156194
	H,0,-0.9423990091,0.0840119972,-4.2247689263
	H,0,0.7777967184,-0.3436287785,-4.1315639611
	C,0,4.1839940302,-0.2250699244,-0.1163599387
	C,0,5.1493773754,-1.176216076,0.0907802479
	H,0,5.4134590965,-1.6188768561,1.0475597791
	C,0,5.7185351221,-1.4600926847,-1.1833552208
	H,0,6.5157108836,-2.1649776353,-1.4076918259
	C,0,5.0593914841,-0.6701812583,-2.077755608
	H,0,5.1382001919,-0.5212092219,-3.1505192172
	O,0,4.1238359947,0.0767642291,-1.4459061205
	C,0,3.5667331616,0.0030378096,2.5709682531
	C,0,3.9878670621,0.6679818143,3.6951502629
	H,0,4.1398092093,1.7425060327,3.7781882545
	C,0,4.1976587452,-0.3215941521,4.6986771447
	H,0,4.5399885268,-0.1678892795,5.718995682
	C,0,3.892573848,-1.5171096044,4.1169598327
	H,0,3.9020381334,-2.5465971548,4.463571544
	O,0,3.5048543709,-1.3330899594,2.8351981682
	C,0,3.5919993603,2.267194696,1.0120122368
	C,0,2.9140905422,3.4589707168,0.9892118285
	H,0,1.8324878313,3.5635893964,0.915560634
	C,0,3.8935166805,4.4871736134,1.0850326675

	H,0,3.7296831305,5.5619220339,1.0981427605
	C,0,5.0994755051,3.8487250779,1.1524028083
	H,0,6.1258122757,4.1988151237,1.2232250535
	O,0,4.9357897919,2.511515804,1.110615329
	C,0,-0.9006387597,-3.0195014259,0.6331606296
	C,0,-1.7093527315,-3.8101995808,1.4090173355
	H,0,-1.5671825954,-4.0249918323,2.4656768668
	C,0,-2.7499777419,-4.2862329503,0.5592271522
	H,0,-3.5720082483,-4.9477995795,0.8237218452
	C,0,-2.5062698431,-3.7518327439,-0.6719978421
	H,0,-3.0015057148,-3.8270367082,-1.6353078577
	O,0,-1.3933587193,-2.9824114054,-0.6375561474
	C,0,0.6181146117,-2.2526096034,2.7777824094
	C,0,0.2840715843,-1.3787016246,3.7797691637
	H,0,-0.037242467,-0.3469670575,3.6393707586
	C,0,0.4377823557,-2.0830768866,5.0060186449
	H,0,0.2653056395,-1.7073327907,6.0112475224
	C,0,0.8559887183,-3.3371740376,4.6675855261
	H,0,1.1097280264,-4.2236549461,5.2419457063
	O,0,0.9656610019,-3.4555297177,3.3239665701
	C,0,1.8703763597,-3.0798232695,0.3527580853
	C,0,2.7277016261,-4.0258500013,0.852743855
	H,0,2.757809499,-4.3731264846,1.881139149
	C,0,3.5434463403,-4.4521776287,-0.2341802694
	H,0,4.3401748954,-5.1921354904,-0.2126092858
	C,0,3.1278907975,-3.7416401775,-1.3208896074
	H,0,3.4423012559,-3.7067235051,-2.3594266146
	O,0,2.1244459605,-2.9034691693,-0.9814100066
	C,0,-0.5413586831,2.4213953531,2.6713814756
	C,0,0.5146399381,2.4472661076,3.5468807416
	H,0,1.440676829,1.8838769997,3.4326577089
	C,0,0.1575003225,3.3421189275,4.5941348639
	H,0,0.7477830292,3.614013278,5.4655522374
	C,0,-1.0884921367,3.8041403128,4.2800216404
	H,0,-1.7688640477,4.5020060626,4.7599173204
	O,0,-1.5230758172,3.2578569996,3.1253096693
	C,0,-1.2246149854,2.8879039444,0.0146848314
	C,0,-2.1785002458,3.0445531673,-0.9601814053
	H,0,-2.9816051259,2.3444954058,-1.1804845368

	C,0,-1.9146915863,4.2936926748,-1.5950710906
	H,0,-2.4729166451,4.7552653748,-2.4062528222
	C,0,-0.8183541527,4.8108003542,-0.9713549197
	H,0,-0.2552411768,5.7326699758,-1.0809566962
	O,0,-0.3919393959,3.970267857,0.0018177453
	C,0,-2.4452211287,0.8416880918,1.3376422658
	C,0,-3.6951871646,1.3749069949,1.5350617065
	H,0,-3.926452214,2.4371427394,1.5782345571
	C,0,-4.5919014358,0.2807105682,1.6795833721
	H,0,-5.6656403111,0.3187908019,1.8489328417
	C,0,-3.8274695623,-0.8467996577,1.5640495145
	H,0,-4.0425904288,-1.9117504085,1.5949926405
	O,0,-2.5379889821,-0.521354896,1.3576953289
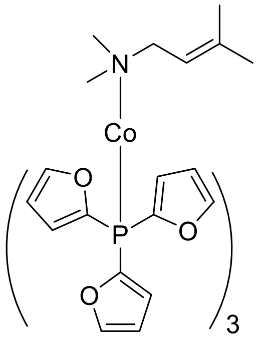<br>**[Co(L1)$_3$(1a')]**<br>***N*-Coordination-Radical**	Co,0,0.950380678,0.147981742,0.5692487586<br>P,0,0.6830123463,-1.946518488,1.061335799<br>P,0,3.0330102816,0.7210394781,0.7448506182<br>P,0,-0.6748693696,1.4191784982,1.1719425496<br>N,0,0.7051984862,0.1053642026,-1.6310695804<br>C,0,4.3526576632,0.0778519962,-0.3426877282<br>C,0,5.0667806837,-1.093094319,-0.3541081861<br>H,0,5.0740014489,-1.8416148441,0.4359992811<br>C,0,5.7658853255,-1.1291577766,-1.5950658508<br>H,0,6.4435722822,-1.9028348503,-1.9486034983<br>C,0,5.4280674304,0.0151022834,-2.2563567964<br>H,0,5.7098913951,0.4431193935,-3.2134786741<br>O,0,4.5699273274,0.7522529809,-1.5104583328<br>C,0,3.6981972548,0.0811755408,2.3172623915<br>C,0,4.9471900733,-0.0580470485,2.8666242355<br>H,0,5.8798491613,0.2326464323,2.3882574433<br>C,0,4.7671003325,-0.6433235027,4.1512643111<br>H,0,5.536354634,-0.9048121716,4.8740008473<br>C,0,3.4202602457,-0.8162067256,4.3020695131<br>H,0,2.7946569326,-1.2212306958,5.0933801543<br>O,0,2.767480013,-0.3831467431,3.2044093559<br>C,0,3.5636961508,2.4597665554,0.8077235484<br>C,0,2.8146208706,3.6057133214,0.7461795356<br>H,0,1.7337444267,3.6401175944,0.6200535764<br>C,0,3.7239086983,4.6937090985,0.875075728<br>H,0,3.493119603,5.7561328315,0.8707974451

	C,0,4.9623629618,4.1328657727,1.0047536246<br>H,0,5.9599460116,4.5463856358,1.1249662193<br>O,0,4.8834435826,2.7866920388,0.9669914738<br>C,0,-0.628152263,-2.9784484347,0.3068417005<br>C,0,-1.9100190065,-3.2437768988,0.7163917812<br>H,0,-2.3342774202,-2.9869144554,1.6853070827<br>C,0,-2.5551381363,-3.902505354,-0.3693340614<br>H,0,-3.5762006417,-4.2755787058,-0.4038553998<br>C,0,-1.6271020182,-3.9938564863,-1.3644663014<br>H,0,-1.6317957243,-4.4218831655,-2.3622520611<br>O,0,-0.4576651352,-3.434311159,-0.9682576706<br>C,0,0.2039887276,-2.2277080653,2.7892997869<br>C,0,-0.1174865372,-1.3422351043,3.7838193901<br>H,0,-0.0737292219,-0.2577852734,3.6995243106<br>C,0,-0.4870776655,-2.1146434194,4.9197564542<br>H,0,-0.8048804892,-1.7519491712,5.8942643682<br>C,0,-0.365821094,-3.420970725,4.536053238<br>H,0,-0.5343305104,-4.3672411532,5.0425712585<br>O,0,0.0473897334,-3.5052102224,3.2573741481<br>C,0,2.1015851613,-3.1044106377,0.9872980387<br>C,0,2.9594703686,-3.5930702081,1.9402163571<br>H,0,2.8665384606,-3.4522376041,3.0148984309<br>C,0,3.9616623755,-4.3302809901,1.2441840031<br>H,0,4.802117736,-4.8708437141,1.6731939926<br>C,0,3.6497802053,-4.2350368047,-0.0802559534<br>H,0,4.0993701939,-4.6226477353,-0.989516697<br>O,0,2.5337456011,-3.4870787264,-0.2479595098<br>C,0,-0.40190501,2.2704256486,2.7567355221<br>C,0,0.7321330841,2.3784487145,3.5221776699<br>H,0,1.685798725,1.901864212,3.3052148764<br>C,0,0.41002228,3.2232482015,4.6201556249<br>H,0,1.0637726018,3.5334463062,5.4312081315<br>C,0,-0.8959525549,3.5815842733,4.4428916666<br>H,0,-1.5801604048,4.2125157338,5.0033876806<br>O,0,-1.3979285433,3.0187682358,3.3252507622<br>C,0,-1.3871422243,2.8203069572,0.2189907518<br>C,0,-2.6527831965,3.1383250939,-0.2051079269<br>H,0,-3.5525791502,2.5612422598,-0.0066526227<br>C,0,-2.5481307079,4.3609176236,-0.9295520177

	H,0,-3.350044849,4.9161051813,-1.4104935349
	C,0,-1.2294469264,4.7064286349,-0.9007351515
	H,0,-0.661143035,5.5416929297,-1.298798144
	O,0,-0.5173709372,3.7815810878,-0.2149157189
	C,0,-2.2880248511,0.6339860695,1.5577918318
	C,0,-2.9776660385,0.3764794108,2.7149443558
	H,0,-2.7150634533,0.735168253,3.7070225149
	C,0,-4.0986496177,-0.4272620168,2.3539210531
	H,0,-4.8714979587,-0.8173352524,3.0119129688
	C,0,-4.0178121647,-0.6041577883,1.0043603951
	H,0,-4.6255500508,-1.1322044464,0.2755608437
	O,0,-2.9235492266,0.0227900647,0.51325545
	C,0,1.1590191072,1.3889031702,-2.1601109624
	C,0,1.5153693754,-0.9739916287,-2.1969258418
	C,0,-0.7366029869,-0.1063581576,-1.9275876971
	H,0,1.5468260691,-0.9334459136,-3.302779668
	H,0,2.5433552294,-0.9104053813,-1.820506896
	H,0,1.1089257615,-1.9477224241,-1.898697936
	C,0,-1.074461809,-0.3206190607,-3.3680563685
	H,0,-1.0613580139,-0.9702208794,-1.3288448627
	H,0,-1.2790372026,0.7573230002,-1.5189209372
	H,0,1.0020940335,1.4668672072,-3.2542993029
	H,0,0.6263191181,2.2112177058,-1.665022538
	H,0,2.232490711,1.5118403728,-1.9635201153
	C,0,-1.5746902304,0.5905899461,-4.2246032319
	H,0,-0.8906043183,-1.3293197605,-3.7604449221
	C,0,-1.8758729766,0.231252804,-5.644856545
	C,0,-1.8733949069,2.0092863832,-3.8568681385
	H,0,-1.3174418029,0.8717728231,-6.3467117788
	H,0,-1.6314343666,-0.8140594671,-5.8732661207
	H,0,-2.9411402634,0.3893005952,-5.8797509408
	H,0,-1.2788149451,2.7056366953,-4.4711012575
	H,0,-2.9281048161,2.2574795955,-4.0594599957
	H,0,-1.6744144875,2.2460950693,-2.8050250758

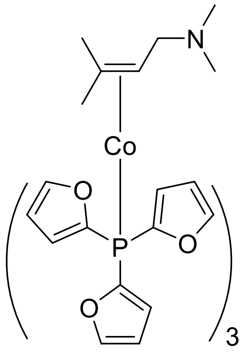

[Co(L1)$_3$(1a')]
C=C-Coordination-Radical

Co,0,-0.0209924637,0.2658306306,-0.5929267906
P,0,1.9716335842,-0.5690709361,-0.2132598542
P,0,-0.2791818118,1.8749711795,0.8880555769
P,0,-1.270181214,-1.4609749502,0.1574111223
C,0,-0.0942748907,0.265064554,-2.6769849996
C,0,-1.0376993266,1.1713105887,-2.1161088009
C,0,0.0973671675,3.5317152415,0.212693373
C,0,1.1591445298,4.3888456148,0.3330701904
H,0,2.0447897664,4.221650838,0.9409858464
C,0,0.864195787,5.5134444838,-0.4911218931
H,0,1.4735654883,6.4011570092,-0.6435194141
C,0,-0.3549731265,5.2627088247,-1.0501249788
H,0,-0.9978336505,5.8100940406,-1.7323921114
O,0,-0.8268072931,4.0674005802,-0.6315757128
C,0,0.7760511851,1.9780607737,2.3757520895
C,0,0.5350511875,2.0687942413,3.7233083168
H,0,-0.4392020334,2.2040397114,4.1874836699
C,0,1.8005047128,1.9720313091,4.3712455085
H,0,2.0014964247,2.0069426179,5.4390756359
C,0,2.7232982694,1.8336511547,3.376798673
H,0,3.8039691564,1.7272089313,3.3599234765
O,0,2.1159901326,1.8311357565,2.1691076412
C,0,-1.8797995179,2.2694998898,1.6467101299
C,0,-3.0997836459,1.6484798281,1.5835494011
H,0,-3.3167181432,0.7151641424,1.0687313815
C,0,-4.0098101816,2.4583926729,2.3182591738
H,0,-5.0686419714,2.2786866408,2.4857746633
C,0,-3.2829122696,3.522067286,2.770453173
H,0,-3.5308672533,4.3992015831,3.3616204249
O,0,-1.9994231456,3.4255981673,2.3736305674
C,0,2.6770670012,-2.0380163991,-1.0431125005
C,0,3.2792174177,-3.1756968161,-0.5687202927
H,0,3.4447448105,-3.4215468945,0.4778979821
C,0,3.6379046398,-3.950617117,-1.7086681144
H,0,4.1374969993,-4.9164698984,-1.721839838
C,0,3.2270224008,-3.2348953259,-2.7950076866
H,0,3.2794476012,-3.3988034908,-3.8673564474
O,0,2.641786743,-2.0810535528,-2.4044343342
C,0,2.5583381322,-1.0312834841,1.4512078231

	C,0,1.9152053392,-1.5796231393,2.5283595752<br>H,0,0.8545306839,-1.8096292839,2.5853060364<br>C,0,2.9040057877,-1.8000110604,3.5285064697<br>H,0,2.7594763476,-2.2179412467,4.5216845752<br>C,0,4.0807648964,-1.3636984644,2.9923896861<br>H,0,5.0996953279,-1.3090868257,3.3652182474<br>O,0,3.8875875689,-0.9007099533,1.7383186828<br>C,0,3.250063425,0.617326824,-0.7269895698<br>C,0,4.512324661,0.4855430934,-1.2459610413<br>H,0,5.0471136943,-0.4529993929,-1.3781761841<br>C,0,4.9573115291,1.7982380308,-1.5660231225<br>H,0,5.9115083598,2.0885170193,-1.9989504179<br>C,0,3.9339960958,2.6357514199,-1.2224719904<br>H,0,3.7913097511,3.7112527843,-1.2790257347<br>O,0,2.9000372199,1.9335483933,-0.7185053743<br>C,0,-1.8013321754,-1.6179563694,1.8934986936<br>C,0,-1.4646406688,-0.9303254566,3.030112917<br>H,0,-0.7429539647,-0.1185473622,3.092331825<br>C,0,-2.2330151387,-1.4836809015,4.0911435952<br>H,0,-2.2299180345,-1.1854344101,5.1364458204<br>C,0,-2.9932411678,-2.4671516458,3.5253852286<br>H,0,-3.7356098029,-3.1565099779,3.9176109304<br>O,0,-2.7444541115,-2.5613227655,2.2053803249<br>C,0,-2.8912849205,-1.8674362967,-0.5944664916<br>C,0,-3.3003088629,-2.7698728458,-1.5409342603<br>H,0,-2.6796208463,-3.5343021238,-2.0014528416<br>C,0,-4.6740841282,-2.4927258807,-1.7973754085<br>H,0,-5.3336422898,-3.0079384394,-2.4911471546<br>C,0,-5.00866714,-1.4480799261,-0.9875755639<br>H,0,-5.9264259892,-0.894701126,-0.8129485912<br>O,0,-3.9383309812,-1.0647356583,-0.2517913472<br>C,0,-0.522928356,-3.1188821678,-0.003284846<br>C,0,-0.2185786025,-4.0999407629,0.9051850737<br>H,0,-0.4168359123,-4.0630279313,1.9742372235<br>C,0,0.3729715513,-5.1658800543,0.169421503<br>H,0,0.7398631309,-6.113372194,0.5560433088<br>C,0,0.3864209498,-4.7620010098,-1.1326610597<br>H,0,0.7386705526,-5.2130728291,-2.0552793795<br>O,0,-0.1426642723,-3.5232916935,-1.2493340709

	C,0,1.1496564605,0.8732230512,-3.2822790797<br>C,0,-0.5046262607,-1.0178811481,-3.3459714879<br>C,0,-2.5306045058,1.0461527888,-2.1754182664<br>H,0,-0.7283452233,2.225206275,-2.1218698869<br>H,0,0.9698750889,1.1140822797,-4.3481294367<br>H,0,1.4344300268,1.8146851674,-2.789722827<br>H,0,2.0177274905,0.1976131126,-3.2590294417<br>H,0,-0.7014028371,-0.8475471127,-4.4224341875<br>H,0,0.28885733,-1.7751585049,-3.2882527568<br>H,0,-1.4138329121,-1.461771765,-2.9199864721<br>N,0,-3.1268412357,1.8731967058,-3.2198997071<br>H,0,-2.8315954622,-0.0179327153,-2.3053655278<br>H,0,-2.9796121538,1.376917256,-1.2229899698<br>C,0,-4.5606316143,1.8908336104,-3.1114192864<br>C,0,-2.7000552032,1.4946105057,-4.5419729608<br>H,0,-3.1248284001,2.1801871362,-5.2904204693<br>H,0,-1.6051750624,1.5486403847,-4.6246082944<br>H,0,-3.0072502658,0.4598022862,-4.8250483101<br>H,0,-4.9968223683,2.581631923,-3.8488458667<br>H,0,-5.0305229516,0.891245179,-3.2745799466<br>H,0,-4.8629686456,2.2334327874,-2.1098521567
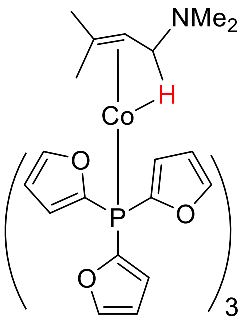<br><br>**[Co(L1)$_3$(1a')]**<br>**C=C-Coordination**<br>**CH-activation-TS**	Co,0,-0.3847927698,0.0530492428,-0.5644393974<br>P,0,1.8737398602,-0.4014518225,-0.391054113<br>P,0,-0.2989857252,1.8425323566,0.7656913805<br>P,0,-1.258996695,-1.7135933675,0.2080676888<br>C,0,-0.5121011386,-0.3282105816,-2.8187899452<br>C,0,-0.7111219387,1.0119002889,-2.4225223786<br>C,0,0.1194935829,3.5405624794,0.1821660783<br>C,0,0.6852169465,4.616934822,0.8181700283<br>H,0,0.9636393755,4.6560125861,1.8690859065<br>C,0,0.8162629133,5.6466236681,-0.1547154226<br>H,0,1.218506756,6.6470264546,-0.0123997882<br>C,0,0.3267001518,5.1269011331,-1.3189706192<br>H,0,0.2032817569,5.5184076054,-2.3245017198<br>O,0,-0.0902718033,3.8570903118,-1.1269479243<br>C,0,0.7987303472,1.9350407586,2.2205570978<br>C,0,0.6177544206,1.8885433632,3.5797796008<br>H,0,-0.3395457335,1.8690685878,4.0962232608<br>C,0,1.9182426427,1.9122602626,4.1635972693

H,0,2.1686861364,1.89062188,5.2212770563
C,0,2.7976281788,1.975216759,3.1229976763
H,0,3.8808854333,2.0109653044,3.0495926831
O,0,2.1321085881,1.9912305432,1.9471421378
C,0,-1.8670690187,2.2208801773,1.6121820794
C,0,-3.081690584,1.5825411713,1.6324916785
H,0,-3.3238529431,0.6593903111,1.1098093334
C,0,-3.9356342774,2.3500867796,2.4726091821
H,0,-4.9733741899,2.1463152389,2.7245962724
C,0,-3.185617529,3.4069935623,2.9030219126
H,0,-3.391761323,4.2569903083,3.5475004591
O,0,-1.9402439273,3.3443535099,2.3939783182
C,0,2.8360359988,-1.738233641,-1.1966089639
C,0,2.9976244809,-3.049920408,-0.8296333365
H,0,2.5967358878,-3.5094812158,0.0723403966
C,0,3.7845488672,-3.6615647117,-1.8455017345
H,0,4.1183525233,-4.6957276196,-1.8912124701
C,0,4.0558803149,-2.6791874815,-2.7535139164
H,0,4.6242610593,-2.6455045405,-3.6780357651
O,0,3.4926173648,-1.5116072318,-2.3675866824
C,0,2.4496408568,-0.8485647419,1.2805502124
C,0,1.8042751802,-1.373815746,2.3665382699
H,0,0.7348225031,-1.5382573565,2.4516224038
C,0,2.803552239,-1.6702149796,3.3369246523
H,0,2.6585944395,-2.0940884659,4.3274473295
C,0,3.9896303064,-1.299907806,2.7745103889
H,0,5.0195715985,-1.3160138054,3.1196755761
O,0,3.7926560089,-0.8045181954,1.5346142982
C,0,2.982170473,0.9806194607,-0.7918868957
C,0,4.3420588242,1.1566718174,-0.8037413049
H,0,5.0785563387,0.3831225988,-0.6017386543
C,0,4.572119235,2.5243049907,-1.1155749668
H,0,5.5292514454,3.0312274923,-1.2128244399
C,0,3.3367324657,3.0857021859,-1.2794839842
H,0,2.9898916302,4.0870097779,-1.5215478969
O,0,2.3739946482,2.1628892995,-1.095941073
C,0,-1.76362812,-1.8655756538,1.9647895014
C,0,-1.5882504107,-1.0405055239,3.0447363172
H,0,-1.0122746234,-0.1182042616,3.0424659954

	C,0,-2.283876402,-1.6287315912,4.1372421657
	H,0,-2.3639594326,-1.2517372845,5.1537451908
	C,0,-2.8451897036,-2.7721808207,3.6451899451
	H,0,-3.4659056196,-3.548399664,4.0841678175
	O,0,-2.5446113022,-2.9276353079,2.3413273286
	C,0,-2.8724287353,-2.2153833487,-0.495299304
	C,0,-3.3618442716,-3.3948269214,-0.9897851497
	H,0,-2.8249991599,-4.3398752925,-1.031018366
	C,0,-4.6874305823,-3.1288742509,-1.4378982487
	H,0,-5.3843499693,-3.8259912286,-1.8968472816
	C,0,-4.916737106,-1.8099514537,-1.1755176676
	H,0,-5.7700249376,-1.1530359724,-1.316270272
	O,0,-3.8271578471,-1.2486048167,-0.6020757876
	C,0,-0.4549117867,-3.3471583486,0.0413464469
	C,0,-0.1032758883,-4.3454576171,0.914058718
	H,0,-0.2619807121,-4.3352510609,1.9898449652
	C,0,0.4695280077,-5.3886956453,0.130449747
	H,0,0.8605881771,-6.3410584803,0.4803457505
	C,0,0.4312336958,-4.9528736908,-1.1621190878
	H,0,0.7568152962,-5.3760639384,-2.1078012778
	O,0,-0.1167992997,-3.7203978975,-1.2274276937
	C,0,0.7833910506,-0.6862427308,-3.4820920761
	C,0,-1.617438389,-1.2696940742,-3.2057162651
	C,0,-1.9784389571,1.596620513,-2.0522833205
	H,0,0.1434756269,1.6963488977,-2.4813636741
	H,0,0.6887976604,-0.6430893051,-4.5841335393
	H,0,1.6104071751,-0.0143319252,-3.2090689114
	H,0,1.0843637933,-1.7193566422,-3.2457825401
	H,0,-1.7607333997,-1.2766830393,-4.3043142693
	H,0,-1.3673265801,-2.305274834,-2.933550115
	H,0,-2.5817704661,-1.0203182705,-2.7532688658
	N,0,-3.1789579527,1.3341168855,-2.6914362691
	H,0,-1.8857163821,0.3655781105,-0.535686855
	H,0,-1.9672717708,2.5092162059,-1.444499066
	C,0,-3.2348332681,1.1983315089,-4.1270846247
	C,0,-4.364185094,1.8619379426,-2.0775886659
	H,0,-5.2587154074,1.3867399829,-2.5073829577
	H,0,-4.3535095243,1.6554699971,-0.9977984212
	H,0,-4.4764010234,2.958367192,-2.2121695229

	H,0,-3.9413089101,0.4069739608,-4.4295693862
	H,0,-3.5511050833,2.1399530932,-4.6193334971
	H,0,-2.2453788483,0.9385316313,-4.5190244459
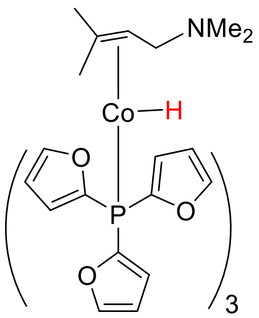	Co,0,-0.478967744,-0.3164770474,-0.5938994725
	P,0,1.7288421095,-0.5354803995,-0.6760056157
	P,0,-0.6045901737,1.5242136074,0.6019675999
	P,0,-0.9811945722,-2.1581840213,0.4690290827
	C,0,-1.0514477871,-0.7563433655,-2.5454389085
	C,0,-0.9392836567,0.6916468705,-2.5747392454
	C,0,0.144916057,3.0597991639,-0.0513219545
**[Co(L1)$_3$(H)(η^1-allyl)]**	C,0,0.3147736241,4.2965395345,0.5187608051
	H,0,-0.007402462,4.5776480431,1.5186889108
	C,0,0.9817182569,5.1032391756,-0.4415590382
	H,0,1.2849756592,6.142698596,-0.3436780433
	C,0,1.1804414765,4.2973566892,-1.5261275763
	H,0,1.6538961398,4.4490198309,-2.491485392
	O,0,0.6837880693,3.0655759542,-1.302752757
	C,0,-0.0261227694,1.7948706834,2.319795875
	C,0,-0.6484978314,1.718436847,3.5396324382
	H,0,-1.712795565,1.5615609184,3.7015934936
	C,0,0.3632706407,1.9018131172,4.5256313559
	H,0,0.2410773715,1.9119528574,5.6061014236
	C,0,1.5282333409,2.0826097684,3.8384405537
	H,0,2.5562203319,2.2642353801,4.1378665063
	O,0,1.3053751001,2.0220154467,2.5070267585
	C,0,-2.3175540759,2.0880224561,0.9040790981
	C,0,-3.5371418761,1.4635673029,0.8161574143
	H,0,-3.7017162348,0.4462998268,0.4653753863
	C,0,-4.5112192466,2.3996579409,1.2661247736
	H,0,-5.586850734,2.2556694385,1.3332262243
	C,0,-3.8202792027,3.5303334649,1.5960983646
	H,0,-4.1150200753,4.505159323,1.9743731571
	O,0,-2.5006668692,3.3547370149,1.3869257458
	C,0,2.6246010558,-1.9118257419,-1.4922808019
	C,0,2.9618328761,-3.1587622324,-1.0381943245
	H,0,2.765592805,-3.5484073028,-0.0412649775
	C,0,3.6112723737,-3.8201978934,-2.1186066342
	H,0,4.0188007918,-4.8284820713,-2.1257535392
	C,0,3.6315566843,-2.9260413255,-3.1489480454

H,0,4.0213155733,-2.9538319411,-4.1618828046
O,0,3.0429368252,-1.7674102946,-2.7784596665
C,0,2.4547916721,-0.774683503,0.967624917
C,0,1.8759578629,-0.8427802347,2.2061794945
H,0,0.8210911376,-0.6904256479,2.4170868249
C,0,2.9199242796,-1.0955552196,3.1365782342
H,0,2.8283660676,-1.2081753251,4.2143641171
C,0,4.0666914272,-1.1638790052,2.3969861798
H,0,5.1089566491,-1.3396394062,2.648742407
O,0,3.8049368063,-0.9734280824,1.0902181183
C,0,2.7617836709,0.8490565139,-1.2558871592
C,0,3.5880328523,1.7334322698,-0.6133900243
H,0,3.8214916874,1.7305572755,0.4488298365
C,0,4.0657642057,2.6300478028,-1.610893957
H,0,4.7496984762,3.4645499555,-1.4758909218
C,0,3.4932999199,2.2318484938,-2.7837287108
H,0,3.5595430979,2.5821318329,-3.8097990678
O,0,2.6929350678,1.1618117486,-2.5785041545
C,0,-1.1765106611,-2.1103269421,2.290407544
C,0,-1.9942295375,-1.2902493432,3.0285534658
H,0,-2.7281337475,-0.5955979817,2.6226412256
C,0,-1.6778091101,-1.5230025718,4.3965724408
H,0,-2.1258933614,-1.051958407,5.2684093801
C,0,-0.684504514,-2.456870452,4.4031577158
H,0,-0.1191720852,-2.9521536188,5.1866411203
O,0,-0.3755558404,-2.8225520151,3.1324495024
C,0,-2.6464451501,-2.8228143459,0.1028958045
C,0,-3.1048839033,-4.075123933,-0.2164358865
H,0,-2.4967225147,-4.9693702843,-0.328277839
C,0,-4.5154277477,-3.9646796139,-0.3755157667
H,0,-5.215645014,-4.7538484253,-0.6379937614
C,0,-4.8218443223,-2.6588669369,-0.1317023648
H,0,-5.7486044687,-2.0931230346,-0.121364296
O,0,-3.6990480271,-1.9595085238,0.1595532233
C,0,-0.1117419993,-3.7474115646,0.2504940042
C,0,0.4722721621,-4.7175598606,1.0287291301
H,0,0.5507702581,-4.703446711,2.1103700645
C,0,0.9254681888,-5.7337190294,0.1395833283
H,0,1.435617039,-6.6579342257,0.4007916248

	C,0,0.5927904233,-5.3170660534,-1.1162569366<br>H,0,0.7360444698,-5.731709803,-2.1097025959<br>O,0,-0.0290934984,-4.1212877674,-1.0636697526<br>C,0,-0.0155867775,-1.4694647707,-3.3812698554<br>C,0,-2.4181741306,-1.3636574687,-2.7526876836<br>C,0,-1.9931370757,1.5901859415,-2.4138672385<br>H,0,0.0074951576,1.0934677185,-2.9546061217<br>H,0,-0.3792760346,-1.6155247567,-4.4179599762<br>H,0,0.9196680583,-0.8983205744,-3.4548918477<br>H,0,0.2320689623,-2.4697566579,-2.9923311807<br>H,0,-2.7301709126,-1.2564118924,-3.8107764925<br>H,0,-2.4137139383,-2.4430875481,-2.5361244569<br>H,0,-3.2041243951,-0.9150568571,-2.1317724134<br>N,0,-1.9733746811,2.9448718766,-2.6589389925<br>H,0,-2.9329361128,1.2336057073,-1.9796888675<br>C,0,-3.073555753,3.7621561122,-2.2302539477<br>C,0,-1.1348430725,3.5264711132,-3.6703532515<br>H,0,-0.8281520388,4.542240547,-3.3772059441<br>H,0,-0.225477642,2.9332810657,-3.8173572762<br>H,0,-1.654425925,3.603237682,-4.6469057596<br>H,0,-2.7114984823,4.7018734091,-1.777809046<br>H,0,-3.7526412531,4.0364282396,-3.0616411914<br>H,0,-3.6608540991,3.234551242,-1.4679980859<br>H,0,-1.9560091597,-0.2515123683,-0.3877431498
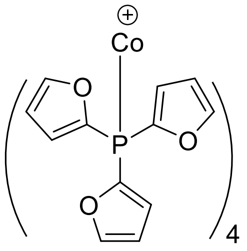<br>**[Co(L1)$_4$]-Cation-Singlet**	Co,0,-0.0212412374,-0.026544057,0.0108410118<br>P,0,1.2793720011,1.3831921469,-1.0304315038<br>P,0,0.5022482563,0.6182056799,2.0281953834<br>P,0,0.5281895833,-1.8097505809,-1.1230063298<br>P,0,-2.1417088424,-0.4122584722,0.2143525189<br>O,0,0.509460683,3.7944610527,-2.063267758<br>O,0,-0.1580653074,0.4620541774,4.6617121041<br>O,0,-0.2544988825,-4.0396869933,-2.4910686352<br>C,0,0.2641371526,2.8755089332,-1.0813009026<br>C,0,-0.3605701966,-0.1177361912,3.4416494971<br>C,0,-0.4916524826,-2.7043787266,-2.3186685766<br>C,0,-0.737458172,3.3339668705,-0.2627017882<br>C,0,-1.1537654235,-1.2266682991,3.5902482312<br>C,0,-1.4426656145,-2.2891108971,-3.2131071012<br>H,0,-1.1548851563,2.8113203053,0.5957277438

	H,0,-1.4752827671,-1.8953782924,2.794786563
	H,0,-1.8203363359,-1.2741999533,-3.305100705
	C,0,-1.1175653478,4.6075109653,-0.7668128108
	C,0,-1.4554758429,-1.3259214028,4.9766417938
	C,0,-1.8090109365,-3.4339561317,-3.974849024
	C,0,-0.3284208911,4.8321093054,-1.858470375
	C,0,-0.8235026736,-0.2739589136,5.5754157648
	C,0,-1.0563491306,-4.4646597827,-3.4902178517
	O,0,3.918940383,1.2701325505,-0.2729013743
	O,0,2.6688148354,-0.840190817,1.3297278798
	O,0,3.2004143272,-1.3936529719,-1.4304717263
	C,0,2.863103834,2.0964641095,-0.5211766751
	C,0,2.2036579319,0.0366741558,2.2614593532
	C,0,2.0863127684,-1.8736353939,-2.0481867414
	C,0,3.23916278,3.3993650609,-0.3090938938
	C,0,3.1306648836,0.1698368957,3.2660081732
	C,0,2.4093500534,-2.3044451192,-3.310255622
	H,0,2.6042095152,4.2754561485,-0.4200114459
	H,0,3.023128636,0.78996789,4.1536634791
	H,0,1.723369821,-2.7354517199,-4.0364366018
	C,0,4.602100871,3.3643669117,0.0938627304
	C,0,4.2266909624,-0.6638626597,2.9125832255
	C,0,3.804971386,-2.0746014331,-3.4673990102
	C,0,4.9585919013,2.0459646513,0.0980249744
	C,0,3.886938587,-1.2512461926,1.7258031195
	C,0,4.2290360708,-1.5170375507,-2.2962760056
	O,0,0.6334341195,0.5329790304,-3.5319298624
	O,0,-0.4155836578,3.0085933177,2.9781142087
	O,0,0.4553173793,-2.7329906746,1.4309622179
	C,0,1.6296653993,1.0835113885,-2.7794922815
	C,0,0.7059214004,2.3217247553,2.6160576579
	C,0,0.8715015759,-3.0316572353,0.1683370753
	C,0,2.7304976335,1.3103472877,-3.563663798
	C,0,1.7929392159,3.1569213043,2.6722875378
	C,0,1.5852137577,-4.2020824773,0.1853921906
	H,0,3.6716006447,1.7463292498,-3.2345365975
	H,0,2.8204603334,2.8873055187,2.4408401978
	H,0,2.0422167162,-4.6793875486,-0.6787655244
	C,0,2.3864317325,0.8740475924,-4.8732082768

C,0,1.3057115308,4.4254033626,3.0946883294
C,0,1.5962823788,-4.6461734004,1.536529405
C,0,1.1053162401,0.4117887247,-4.7927914261
C,0,-0.0386395005,4.2745592569,3.2663435321
C,0,0.8987206792,-3.7109944639,2.2475249752
O,0,-1.9840324842,0.7993787597,-2.2159509608
O,0,-2.692174312,1.3521828004,2.156534891
O,0,-2.2647572472,-3.1034378438,0.2145394952
C,0,-2.7098325089,0.7327357395,-1.060940439
C,0,-3.1246096351,0.186000043,1.6009040286
C,0,-2.9495663657,-1.9804252319,-0.1521466072
C,0,-3.6659485677,1.7193927721,-1.0651184594
C,0,-4.1695158889,-0.3156975815,2.3321001273
C,0,-4.075342313,-2.3357746258,-0.8493566375
H,0,-4.3805363001,1.9180201322,-0.2688409622
H,0,-4.7088364013,-1.2381783544,2.1278196641
H,0,-4.8040800704,-1.6502050921,-1.27742481
C,0,-3.5176489667,2.4177687951,-2.2937585573
C,0,-4.3852238631,0.6013451098,3.3991426065
C,0,-4.0795651512,-3.7566484568,-0.9074155269
C,0,-2.4774209352,1.8205106018,-2.9470568706
C,0,-3.4568828847,1.5902980453,3.2446846193
C,0,-2.9561388655,-4.165587801,-0.2468808679
H,0,0.6442808622,-3.6003605202,3.2984730227
H,0,0.4178906045,-0.0201391305,-5.515046966
H,0,-0.8311377785,4.9421999217,3.5913087217
H,0,2.0639465974,-5.5432599442,1.934238787
H,0,3.0108895898,0.8971117255,-5.7625497118
H,0,1.8821595868,5.3310589013,3.2641203781
H,0,5.1480180803,-0.8141924203,3.4697255894
H,0,4.4161305601,-2.2926203618,-4.3394036882
H,0,5.2394526308,4.2078075237,0.3463525177
H,0,4.3818688412,-1.958533799,1.0664332076
H,0,5.1929484429,-1.1676548662,-1.9362143564
H,0,5.879097919,1.5200686495,0.3350930055
H,0,-0.2508348148,5.6489243613,-2.570188211
H,0,-0.7599605691,0.0755407696,6.6015754388
H,0,-0.9877642649,-5.5205746688,-3.7350656509
H,0,-1.8840290029,5.2709624346,-0.3744000457

	H,0,-2.0678375013,-2.0777209263,5.468035428<br>H,0,-2.541929327,-3.4897602575,-4.7755598877<br>H,0,-1.9911226285,1.9956618093,-3.9028887701<br>H,0,-3.2218839292,2.4944728104,3.798108065<br>H,0,-2.5260915312,-5.1389686653,-0.0275179156<br>H,0,-4.1041416372,3.2608705605,-2.6494825849<br>H,0,-5.1329001639,0.53540808,4.1851880043<br>H,0,-4.8180825176,-4.3964301879,-1.3833496164
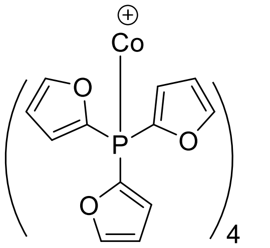<br>**[Co(L1)$_4$]-Cation-Triplet**	Co,0,-0.1155434259,-0.1742487629,-0.0754218843<br>P,0,0.6907234498,1.6562481334,-1.190059311<br>P,0,0.7761833203,0.1452592807,2.0014882273<br>P,0,1.1169138369,-1.9330470434,-0.9041293035<br>P,0,-2.3789190637,0.1227249216,0.1111805132<br>O,0,-0.0429590242,4.0194356848,-2.3635188678<br>O,0,-0.0567461064,0.1662880605,4.6189846355<br>O,0,0.2963387516,-4.1057873803,-2.3740064459<br>C,0,-0.2903384001,3.1637781726,-1.3275084334<br>C,0,-0.2701191173,-0.364162633,3.3796248603<br>C,0,0.042777458,-2.801531423,-2.0609443549<br>C,0,-1.2718768342,3.6853664571,-0.5250873792<br>C,0,-1.2808687346,-1.2884260852,3.4419769905<br>C,0,-1.0591693113,-2.366469665,-2.753711779<br>H,0,-1.6604116752,3.2264017901,0.3820921301<br>H,0,-1.6551827431,-1.8734392456,2.6034989717<br>H,0,-1.4817878964,-1.3634156719,-2.7111621319<br>C,0,-1.6437080554,4.9315595647,-1.1010487991<br>C,0,-1.7080067785,-1.3277355326,4.7981750845<br>C,0,-1.4998657006,-3.4658079831,-3.5393969994<br>C,0,-0.8658096769,5.0776913653,-2.2145327531<br>C,0,-0.9306311264,-0.421928581,5.4625083501<br>C,0,-0.6400822691,-4.4919300814,-3.2638559505<br>O,0,3.2685523926,1.4562512569,-0.4459923658<br>O,0,3.3403457062,-0.6491961281,1.582501287<br>O,0,3.7914737525,-1.4498180482,-1.2699757798<br>C,0,2.2702140947,2.3526476843,-0.6666788239<br>C,0,2.3002022295,-0.7165067112,2.4579540616<br>C,0,2.6426189071,-1.8761712737,-1.8610878313<br>C,0,2.7198847194,3.6229349754,-0.4144861804<br>C,0,2.6501792333,-1.4954074168,3.5317100042

C,0,2.8541460656,-2.0716879308,-3.2029008226
H,0,2.1415902742,4.539558904,-0.5099159877
H,0,2.0205762517,-1.7272942058,4.388233654
H,0,2.116400346,-2.416606231,-3.9241805181
C,0,4.0798949082,3.4889457911,-0.0145358073
C,0,3.9883231934,-1.9199250454,3.2982485689
C,0,4.2164556443,-1.7435757105,-3.443892487
C,0,4.3548390937,2.152013325,-0.0518223993
C,0,4.351410451,-1.3773681983,2.0995650639
C,0,4.7326004557,-1.3740416841,-2.236083801
O,0,0.3078569055,0.3737823704,-3.5622193886
O,0,0.3980740623,2.8315978555,2.0073113337
O,0,0.542174366,-3.2629029194,1.362535177
C,0,1.1132667651,1.2584797588,-2.903776302
C,0,1.2391357733,1.8391175465,2.4171618279
C,0,1.4788615366,-3.150204305,0.373151817
C,0,2.1534079239,1.6255092067,-3.7179301034
C,0,2.3148090895,2.3839597884,3.0693727027
C,0,2.5124987953,-4.0161803713,0.6139226089
H,0,2.9547606823,2.3132101885,-3.4549236418
H,0,3.1456728935,1.8302997211,3.5022920556
H,0,3.3967841636,-4.1428900667,-0.0066799405
C,0,1.9655988161,0.9369370265,-4.9478152341
C,0,2.1139701881,3.7919639889,3.0647112968
C,0,2.1867914333,-4.7032245047,1.8166249427
C,0,0.8309691383,0.1934842104,-4.7951820887
C,0,0.9365492077,4.0041868034,2.407609724
C,0,0.9835046268,-4.2055289645,2.2244472746
O,0,-2.4416398264,1.0540681852,-2.3900315792
O,0,-2.541061087,1.7217254436,2.2650530456
O,0,-2.3129516019,-2.5470383184,0.3563223566
C,0,-3.1314969015,1.0774574047,-1.2126550527
C,0,-3.1892641446,0.7232749869,1.5979316055
C,0,-3.0762334387,-1.5226776531,-0.1324517436
C,0,-4.1728038258,1.9637765326,-1.3060840554
C,0,-4.2736597525,0.3085640979,2.3284604996
C,0,-4.133008343,-2.0392144529,-0.8383186133
H,0,-4.8843331308,2.2003601267,-0.5175577106
H,0,-4.9658604489,-0.4840267406,2.0515949289

	H,0,-4.8985927543,-1.4627714159,-1.3539607092<br>C,0,-4.1156041944,2.5099805052,-2.6194239367<br>C,0,-4.2921801894,1.1075860877,3.5047548313<br>C,0,-4.0135950325,-3.4551607657,-0.7642544632<br>C,0,-3.0448383872,1.9227423436,-3.2299017277<br>C,0,-3.2174078656,1.9447824262,3.4109900504<br>C,0,-2.8897151797,-3.703904444,-0.0293801355<br>H,0,0.339154,-4.4048672917,3.0762197344<br>H,0,0.2890785521,-0.4907339247,-5.4427017206<br>H,0,0.3719962559,4.8958920498,2.1489482621<br>H,0,2.7742064335,-5.4668468082,2.3198223379<br>H,0,2.5939042864,0.9830073147,-5.8336704907<br>H,0,2.7624743979,4.5532335522,3.4906772336<br>H,0,4.6017198485,-2.551403499,3.9357512895<br>H,0,4.7484167796,-1.7830837188,-4.3907537106<br>H,0,4.766170483,4.2832928769,0.2675110447<br>H,0,5.2565596617,-1.4207858121,1.5005334322<br>H,0,5.719750314,-1.0587052395,-1.9106122086<br>H,0,5.2391624375,1.5610228289,0.1696044356<br>H,0,-0.7907823363,5.8460612806,-2.9784351498<br>H,0,-0.8882997401,-0.0882161958,6.4951849056<br>H,0,-0.5716627665,-5.5208626676,-3.6050094254<br>H,0,-2.3964285096,5.6291390834,-0.7424748008<br>H,0,-2.4963711902,-1.9406854784,5.2276966434<br>H,0,-2.3500834582,-3.4981561988,-4.2159495601<br>H,0,-2.5999509137,2.0091412426,-4.217501438<br>H,0,-2.8163985462,2.7254528467,4.0510499144<br>H,0,-2.3858319964,-4.6120099381,0.2910284258<br>H,0,-4.7814461839,3.2499015448,-3.0557310145<br>H,0,-5.0084831337,1.0648356077,4.3209967441<br>H,0,-4.6736386844,-4.1973219753,-1.2056915982
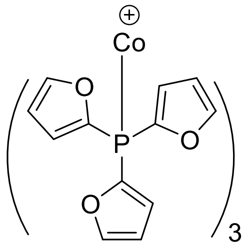<br>**[Co(L1)$_3$]-Cation-Singlet**	Co,0,0.5410474515,0.631125963,-0.2818667611<br>P,0,1.767042642,1.488232034,-1.745742739<br>P,0,0.8695819837,0.4832973725,1.8798223427<br>P,0,0.9683179361,-1.4483826247,-0.7433173462<br>O,0,-0.7620137867,2.4196756971,-2.3017471982<br>O,0,0.0886364989,-0.7965904161,4.1875169893<br>O,0,-0.5359690289,-3.253557676,-2.1602601981<br>C,0,0.280452067,2.3791244072,-1.4061349783

C,0,-0.2320147149,-0.5145208964,2.8905719576
C,0,-0.325927769,-1.9341415027,-1.8887139495
C,0,-0.1921476312,2.6256135623,-0.0913675995
C,0,-1.4992482242,-0.963237508,2.6218605726
C,0,-1.2037175862,-1.1658644287,-2.6120089727
H,0,0.3965854649,3.0731610366,0.7102276871
H,0,-2.0159623814,-0.8772475511,1.6672590038
H,0,-1.2415695309,-0.077609367,-2.6201966522
C,0,-1.6140463508,2.8497467827,-0.2590714771
C,0,-1.9781575183,-1.5629263634,3.8187936385
C,0,-1.9991600205,-2.0692859562,-3.3660680757
C,0,-1.8857491319,2.7089971029,-1.5760527297
C,0,-0.9699534943,-1.4328257308,4.7306595278
C,0,-1.5456903125,-3.3194878658,-3.0502589778
O,0,4.4113504207,1.8071420242,-1.2340091667
O,0,3.2960787583,-0.3498217291,1.0562285909
O,0,3.3707619437,-0.8932543778,-1.8806580688
C,0,3.190941064,2.4149219692,-1.186411855
C,0,2.5304766341,-0.168033838,2.1739383101
C,0,2.5534184379,-1.9041654058,-1.4760711359
C,0,3.2877284466,3.6008406974,-0.4990109333
C,0,3.2431786804,-0.5476819211,3.2817548718
C,0,3.1386421663,-3.1092105562,-1.7725777823
H,0,2.4751781523,4.2974850139,-0.303250819
H,0,2.8860957167,-0.5127392972,4.3084846795
H,0,2.7153463515,-4.0900951156,-1.5663333439
C,0,4.6502157933,3.7303920208,-0.119293089
C,0,4.5110056428,-0.9911081003,2.8135009824
C,0,4.3807322603,-2.8110506044,-2.3977796379
C,0,5.2826138274,2.6159964883,-0.5923727579
C,0,4.4848908938,-0.8487348913,1.454186613
C,0,4.4671073288,-1.4478620486,-2.4380698197
O,0,1.3390379429,0.1713681633,-4.0428577176
O,0,-0.2087048655,2.6256885042,3.1350394229
O,0,-0.4051582575,-3.1929293001,0.8147700153
C,0,2.0221326685,1.2083395428,-3.4832426035
C,0,0.9731792846,2.0114070933,2.8428153769
C,0,0.8413518061,-2.726537743,0.5238449547
C,0,2.8992385022,1.7375033734,-4.3975637213

	C,0,2.018189443,2.8056094091,3.2405864438<br>C,0,1.7469412199,-3.2341630017,1.4228873898<br>H,0,3.5820885199,2.5668092924,-4.2261265121<br>H,0,3.0755320334,2.5731729794,3.1318643827<br>H,0,2.8180061441,-3.0368500736,1.4397976861<br>C,0,2.7237956088,0.9894173164,-5.5916616241<br>C,0,1.4376028339,3.9674649631,3.8256106455<br>C,0,1.0076847927,-4.0644695561,2.3082271724<br>C,0,1.765096099,0.0547344458,-5.315577605<br>C,0,0.0864055324,3.8015402388,3.7371879738<br>C,0,-0.290555637,-4.0009823551,1.8886963889<br>H,0,-1.209257938,-4.4693563205,2.2287827545<br>H,0,1.2937905507,-0.7292051429,-5.9009762912<br>H,0,-0.7640432771,4.3958298313,4.0572881671<br>H,0,1.391037421,-4.6478110426,3.1411151216<br>H,0,3.239256608,1.1267450411,-6.5384606514<br>H,0,1.9563786075,4.812814643,4.270044792<br>H,0,5.3398536831,-1.3670878042,3.408140016<br>H,0,5.1173790693,-3.5171336371,-2.7721811666<br>H,0,5.1087078514,4.5533186948,0.4224437675<br>H,0,5.20358607,-1.0400772486,0.661284623<br>H,0,5.2090172337,-0.7460649779,-2.8090099805<br>H,0,6.3138072701,2.2761480182,-0.569156491<br>H,0,-2.7976855354,2.8054337298,-2.158686628<br>H,0,-0.8595246105,-1.7340751556,5.7682429446<br>H,0,-1.8328112111,-4.3181836774,-3.3661729652<br>H,0,-2.3216583714,3.109961429,0.5243654376<br>H,0,-2.9455721634,-2.0288456262,3.9868820637<br>H,0,-2.8044380101,-1.8285804457,-4.0547642851
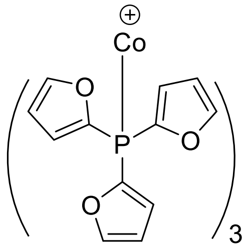<br>**[Co(L1)$_3$]-Cation-Triplet**	Co,0,0.2401640076,0.4665606379,-0.1612207788<br>P,0,1.738091496,1.6844198191,-1.2959398167<br>P,0,1.1790367008,0.5646371692,1.8975934721<br>P,0,0.5632999467,-1.6984265542,-0.7143087521<br>O,0,-0.6427243602,2.2822110037,-2.5304889956<br>O,0,0.8069260845,-0.4933724759,4.4107837041<br>O,0,-0.6121931458,-3.4852715686,-2.4319455686<br>C,0,0.0468958287,2.3367684422,-1.35333968<br>C,0,0.2786726236,-0.3284255821,3.1632721441<br>C,0,-0.4835154786,-2.1699499841,-2.0972982425

	C,0,-0.8617459312,2.5116161806,-0.3001307167<br>C,0,-0.9755960888,-0.8837191359,3.1169355113<br>C,0,-1.1973639287,-1.3974331425,-2.977365932<br>H,0,-0.6157515509,2.7720406059,0.7289973967<br>H,0,-1.6322147811,-0.9037385398,2.2485181885<br>H,0,-1.2532423842,-0.3102505983,-2.9670842416<br>C,0,-2.1525469151,2.6225789902,-0.9133954335<br>C,0,-1.224683883,-1.4268923219,4.4056844912<br>C,0,-1.7984723368,-2.2922556082,-3.9036000475<br>C,0,-1.958792226,2.470288136,-2.2497634836<br>C,0,-0.1067747726,-1.1598279013,5.1443123248<br>C,0,-1.4084694974,-3.5440730221,-3.5193832922<br>O,0,4.1096601335,2.807552746,-0.6709638865<br>O,0,3.7806292776,0.3713459982,1.2107954402<br>O,0,3.1635586023,-1.1721964872,-1.3241037598<br>C,0,2.7737199883,3.0518153157,-0.7844276635<br>C,0,2.7927469894,-0.2344596827,1.9252799396<br>C,0,2.234652261,-2.1631858542,-1.2107492546<br>C,0,2.4809920382,4.2951857587,-0.2824404628<br>C,0,3.2513709768,-1.4183113067,2.442295249<br>C,0,2.7742732268,-3.3486239023,-1.6450340564<br>H,0,1.4956509974,4.7547116729,-0.24077546<br>H,0,2.6860741676,-2.1037311093,3.0693812286<br>H,0,2.2570707624,-4.3054550139,-1.6820784959<br>C,0,3.7169200316,4.848032537,0.1508221658<br>C,0,4.6052932383,-1.5395773343,2.0173344738<br>C,0,4.108775294,-3.0630434766,-2.0401608559<br>C,0,4.6675162317,3.9047041004,-0.1119227009<br>C,0,4.870629895,-0.4229947988,1.2787046201<br>C,0,4.2850306236,-1.7251531533,-1.8251331981<br>O,0,1.6550279159,0.1665157176,-3.4744936984<br>O,0,0.6638638145,3.1478624018,2.5525166581<br>O,0,-1.103009781,-3.147797845,0.8343730493<br>C,0,2.2066535137,1.3107402453,-2.975390684<br>C,0,1.6098037891,2.1622473719,2.6211223107<br>C,0,0.2069440872,-2.988190527,0.4955084531<br>C,0,3.1300804481,1.8055683966,-3.8609097811<br>C,0,2.7371608663,2.6505152194,3.229287214<br>C,0,0.9879036301,-3.8208581824,1.2550856491

	H,0,3.7318946217,2.7011862651,-3.7252704905
	H,0,3.6514335047,2.0937565117,3.4209882513
	H,0,2.0700449324,-3.9130043162,1.1949245874
	C,0,3.1310395356,0.9163554444,-4.9707218175
	C,0,2.4627627254,4.0067257156,3.5573921396
	C,0,0.0979836576,-4.5362846714,2.1055323702
	C,0,2.2142134598,-0.0545983511,-4.6819217011
	C,0,1.1923133531,4.2535383415,3.1266708253
	C,0,-1.1549396289,-4.0892916132,1.8044356773
	H,0,-2.1475928044,-4.3428197879,2.1643216149
	H,0,1.8536402825,-0.9301113797,-5.2149897739
	H,0,0.5445846502,5.1244147187,3.1618673564
	H,0,0.3534182552,-5.2982115764,2.8372204262
	H,0,3.7319989497,0.9896822636,-5.87326206
	H,0,3.1232143312,4.7112767863,4.0561320637
	H,0,5.2993933367,-2.3443327929,2.2453464887
	H,0,4.8441243327,-3.7576832466,-2.4374404221
	H,0,3.8816333904,5.8269379458,0.5932070386
	H,0,5.7535249521,-0.0558040916,0.7634670228
	H,0,5.1202387873,-1.0459744429,-1.9729028012
	H,0,5.7449708827,3.8723459183,0.0179579555
	H,0,-2.6244057517,2.4747661295,-3.1079732038
	H,0,0.1754886741,-1.3718659064,6.171459235
	H,0,-1.6157336969,-4.5395768977,-3.9007374334
	H,0,-3.0994593709,2.8086213912,-0.4138094289
	H,0,-2.1154969189,-1.9477925744,4.7464105613
	H,0,-2.44249487,-2.0456891412,-4.7434202258
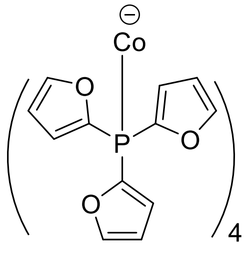 **$[Co(L1)_4]$-Anion-Singlet**	27 -0.00162 0.00489 -0.13283
	15 1.6434 1.0677 0.59974
	15 -1.7413 0.85889 0.65119
	15 0.13077 -1.96599 0.55111
	15 -0.03981 0.05614 -2.20159
	8 3.42724 3.14044 -0.00887
	8 -4.43226 1.39086 0.08186
	8 1.00899 -4.53072 -0.16115
	6 2.21077 2.56961 -0.28494
	6 -3.3277 0.64418 -0.24085
	6 1.10319 -3.18525 -0.41202
	6 1.55944 3.3295 -1.21987

6	-3.66062	-0.25166	-1.22162
6	2.03963	-2.97392	-1.3883
1	0.56761	3.11225	-1.60883
1	-2.97383	-0.97799	-1.65029
1	2.3234	-1.99508	-1.76874
6	2.41561	4.42382	-1.5305
6	-5.03914	-0.04808	-1.51354
6	2.54768	-4.25257	-1.75465
6	3.53529	4.25105	-0.76556
6	-5.45003	0.96439	-0.69278
6	1.88057	-5.15711	-0.97725
8	1.44047	1.06353	3.33879
8	-1.632	0.53296	3.37657
8	0.37064	-1.86201	3.28838
6	1.50959	1.87413	2.24641
6	-2.36841	0.2482	2.26647
6	0.98106	-2.29363	2.14861
6	1.26955	3.17001	2.63163
6	-3.3687	-0.6304	2.60154
6	2.2445	-2.73814	2.45433
1	1.25279	4.03573	1.97186
1	-4.10867	-1.04004	1.91563
1	2.96793	-3.1331	1.74235
6	1.04408	3.14282	4.03846
6	-3.22802	-0.89737	3.99429
6	2.40901	-2.56601	3.85933
6	1.16388	1.83597	4.41155
6	-2.15881	-0.15863	4.41124
6	1.23581	-2.03373	4.31162
8	3.67131	-0.69568	0.05666
8	-1.22749	3.5241	0.25748
8	-2.43679	-2.82733	0.11939
6	3.2765	0.28159	0.92305
6	-1.87826	2.64612	1.07331
6	-1.35003	-2.99427	0.92757
6	4.20241	0.38339	1.93067
6	-2.43159	3.34119	2.11963
6	-1.67607	-3.86801	1.93439
1	4.14596	1.07368	2.76926

1	-3.0029	2.90113	2.93389
1	-1.0095	-4.18307	2.7338
6	5.21459	-0.58059	1.66046
6	-2.1043	4.71313	1.92481
6	-3.03004	-4.25411	1.72483
6	4.83518	-1.20553	0.50638
6	-1.37002	4.76089	0.7735
6	-3.43834	-3.58778	0.60471
8	2.625	0.38231	-2.67152
8	-1.66094	2.22026	-2.54228
8	-1.10527	-2.40829	-2.66734
6	1.39301	0.70664	-3.16121
6	-1.32656	1.0213	-3.10197
6	-0.23998	-1.48948	-3.18519
6	1.54671	1.60733	-4.18641
6	-2.19112	0.75867	-4.13594
6	0.40763	-2.04772	-4.25924
1	0.73477	2.05359	-4.75807
1	-2.17416	-0.13552	-4.75666
1	1.16535	-1.55175	-4.86317
6	2.94662	1.84313	-4.32232
6	-3.09855	1.85628	-4.2044
6	-0.08917	-3.37719	-4.39646
6	3.55033	1.06964	-3.37276
6	-2.72521	2.71327	-3.20853
6	-1.00657	-3.53764	-3.39894
1	-4.37484	-3.54284	0.05446
1	5.26214	-2.00623	-0.09223
1	-0.8883	5.56269	0.21941
1	-3.63079	-4.93119	2.32915
1	6.10627	-0.79255	2.24728
1	-2.3689	5.5575	2.55839
1	-3.84036	-1.55509	4.60797
1	3.28673	-2.803	4.45736
1	0.81721	3.98466	4.68943
1	-1.66527	-0.02172	5.36973
1	0.87365	-1.73026	5.29037
1	1.08416	1.31317	5.36091
1	4.46572	4.80445	-0.66581

	1 -6.39679 1.48205 -0.56017
	1 1.90382 -6.24159 -0.90398
	1 2.23415 5.22835 -2.2404
	1 -5.6474 -0.57291 -2.24765
	1 3.30018 -4.47745 -2.5079
	1 4.58293 0.90997 -3.07379
	1 -3.10404 3.66791 -2.85341
	1 -1.64141 -4.36132 -3.08303
	1 3.4449 2.51103 -5.02221
	1 -3.9325 1.9896 -4.89069
	1 0.20425 -4.12619 -5.12944
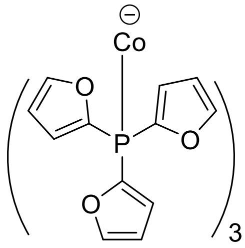	Co,0,0.5179050294,0.5315878527,-0.0611658844
	P,0,1.8392606222,1.6565283707,-1.2114161126
	P,0,1.283518711,0.5118268407,1.8732028751
	P,0,0.3824769351,-1.4290813383,-0.7414170285
	O,0,-0.6706691851,1.9347781813,-2.478971882
	O,0,1.108295211,-0.4068298188,4.5292874808
**$[Co(L1)_3]$-Anion-Singlet**	O,0,-0.8724134015,-3.309330368,-2.3968287377
	C,0,0.0953187093,2.0465139747,-1.3071992822
	C,0,0.5348931669,-0.3729070824,3.2828495024
	C,0,-0.5623852726,-1.9833914987,-2.2202756497
	C,0,-0.7693058827,2.2286614847,-0.1854192138
	C,0,-0.6390055933,-1.0774388939,3.3091139248
	C,0,-1.0254161645,-1.2707807946,-3.296509185
	H,0,-0.5361890878,2.8082681198,0.7109150197
	H,0,-1.2969047645,-1.2070992638,2.4518589385
	H,0,-0.9014061304,-0.1982124106,-3.4248055547
	C,0,-2.1003517754,2.1861872635,-0.7512407481
	C,0,-0.791456429,-1.5789770625,4.6322699607
	C,0,-1.6443085326,-2.2049586587,-4.1756110441
	C,0,-1.9742102323,2.0021182984,-2.0886622498
	C,0,0.3001194384,-1.1366684254,5.3250487513
	C,0,-1.5249514549,-3.4253332807,-3.57360438
	O,0,4.0854360863,3.1405091391,-0.6915318022
	O,0,3.9565612706,0.4077413963,1.2891264808
	O,0,3.1072024507,-1.4058848135,-1.1249572401
	C,0,2.7252915707,3.2011122563,-0.8599730225
	C,0,2.9636415594,-0.2138657295,1.9851620123
	C,0,2.000920799,-2.1949142053,-1.2234404754

C,0,2.2802577033,4.4374541341,-0.4546071451
C,0,3.4313171969,-1.4149806838,2.4600734447
C,0,2.367500512,-3.4019373543,-1.7682205126
H,0,1.2414614483,4.7607723595,-0.4611481504
H,0,2.8592733525,-2.1261135012,3.0526821907
H,0,1.6886863318,-4.2294158154,-1.964682566
C,0,3.4256574909,5.1709472987,-0.0350562241
C,0,4.7847518432,-1.5264152719,2.0270129468
C,0,3.7679232865,-3.3381863453,-2.015163295
C,0,4.4930433015,4.337389687,-0.205285046
C,0,5.048719867,-0.3876851381,1.322366037
C,0,4.1577859138,-2.0936697349,-1.6041874388
O,0,1.9401821603,0.1673675047,-3.4423819755
O,0,0.5638864909,2.8914598257,3.0007544419
O,0,-1.475061531,-2.9358128934,0.6118072012
C,0,2.4270424868,1.3151679071,-2.8866804417
C,0,1.6493030159,2.1207394769,2.6840439372
C,0,-0.1380027095,-2.8069851813,0.3625881351
C,0,3.4150080414,1.8205251618,-3.7010841421
C,0,2.792668479,2.8503670268,2.8968109603
C,0,0.5524941684,-3.6808114959,1.1675747396
H,0,3.9896025371,2.7239331664,-3.5102860368
H,0,3.8068186809,2.5074907425,2.7109479114
H,0,1.6337675197,-3.799590731,1.1848559467
C,0,3.5192182652,0.9358539542,-4.8131686938
C,0,2.3821193323,4.1264318783,3.380198132
C,0,-0.4171583028,-4.3822454424,1.9414020444
C,0,2.5936267239,-0.0473383127,-4.6029139913
C,0,1.0197502379,4.0937372943,3.4253356732
C,0,-1.631439886,-3.8919353246,1.5592550781
H,0,-2.656154439,-4.110880496,1.846015824
H,0,2.2873424333,-0.9303123644,-5.157463335
H,0,0.2588036847,4.8103400167,3.72045259
H,0,-0.2387567594,-5.1546095843,2.6869892658
H,0,4.191116321,1.0175131703,-5.6655277957
H,0,3.0184938075,4.9668042275,3.6503152979
H,0,5.4752307025,-2.3468232672,2.2115755295
H,0,4.4070486963,-4.1065959947,-2.4464476651
H,0,3.4567021876,6.1896911237,0.3463659114

H,0,5.9240971053,-0.0055623348,0.804740747
H,0,5.1113033895,-1.5728918387,-1.5859464843
H,0,5.5617391169,4.4422223761,-0.0402190939
H,0,-2.7052961987,1.8878978812,-2.8870231091
H,0,0.6275876567,-1.2525904763,6.3552745338
H,0,-1.8558151674,-4.426677055,-3.8369696909
H,0,-3.0408206949,2.2958229557,-0.2127703476
H,0,-1.6015215947,-2.192662421,5.0206910473
H,0,-2.1273858584,-2.0000896437,-5.1290628392

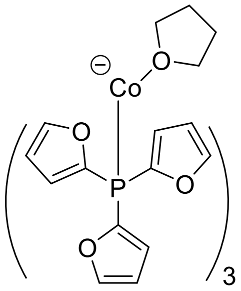

[Co(L1)$_3$(THF)]-Anion-Singlet

Co,0,0.9324631414,0.1727142922,0.5137716474
P,0,0.5424874438,-1.8022005923,0.976851947
P,0,2.8746511521,0.6288528812,1.0490268807
P,0,-0.5973687969,1.3536365431,1.2117135582
O,0,1.0187886452,0.364891174,-1.5853910162
C,0,0.0634243193,-0.3169949389,-2.3825937344
H,0,-0.9617299443,-0.0473064355,-2.061615719
H,0,0.1998507652,-1.3942530514,-2.2090475643
C,0,1.2728321635,1.6585229416,-2.1415164098
H,0,2.3628762471,1.8096684231,-2.1199828116
H,0,0.8166852875,2.4314872797,-1.5001918603
C,0,0.6704560073,1.6379556448,-3.5465955557
H,0,-0.2613030436,2.2248460287,-3.5653052576
H,0,1.341194905,2.0702430789,-4.3032097723
C,0,0.3686089853,0.1612264565,-3.7854589033
H,0,-0.4546906139,-0.0082888931,-4.4940959015
H,0,1.257141822,-0.3646740921,-4.1707660279
C,0,4.1787232233,-0.0683360687,-0.0605851728
C,0,4.8577817063,-1.2639260434,-0.0843419327
H,0,4.8890864868,-1.9894330205,0.724258432
C,0,5.4608967701,-1.3710376504,-1.3705251975
H,0,6.0753679427,-2.1878264445,-1.7451910869
C,0,5.1122457335,-0.2410946931,-2.0508033581
H,0,5.3314842086,0.1399357657,-3.0442539162
O,0,4.3363240128,0.552313287,-1.2707294292
C,0,3.6100209112,0.0685203948,2.6412863275
C,0,3.9460277471,0.7199123128,3.8050131106
H,0,3.9977651456,1.7996046391,3.9322099657
C,0,4.22752187,-0.2867610138,4.7756207868
H,0,4.5276923553,-0.1447259573,5.8118046272

C,0,4.0501116466,-1.4811262179,4.1410312904
H,0,4.1314249215,-2.5191951149,4.4516131125
O,0,3.6757449282,-1.2774969975,2.8567233806
C,0,3.4929521122,2.3541834515,1.1265637245
C,0,2.7848207401,3.5179636981,0.9669723815
H,0,1.7086593133,3.5692378733,0.8032137021
C,0,3.7176064995,4.5884748785,1.0714743668
H,0,3.5159921649,5.6552253953,1.0001085453
C,0,4.9352545956,4.0032424073,1.2792442574
H,0,5.9407459607,4.3967085102,1.4084285346
O,0,4.8183712547,2.6603040363,1.3146316586
C,0,-1.0197098139,-2.6430893173,0.4375819175
C,0,-1.5210915818,-3.9198200584,0.5032226612
H,0,-1.0088707731,-4.7657619663,0.9566881876
C,0,-2.8069019697,-3.898006014,-0.1089897947
H,0,-3.5022268108,-4.7262066108,-0.23472473
C,0,-3.0030178627,-2.6065784949,-0.5113469134
H,0,-3.8206741901,-2.0899908678,-1.0072102264
O,0,-1.935443419,-1.8486879805,-0.1974707175
C,0,0.4963927429,-2.2449613309,2.7554566302
C,0,0.6652157691,-1.4177639605,3.8318275583
H,0,0.8704497927,-0.3505020289,3.7512934074
C,0,0.5127269051,-2.2180787313,4.9981630837
H,0,0.5845892855,-1.898977169,6.0358645341
C,0,0.2535899542,-3.4840248302,4.5526867019
H,0,0.0613690088,-4.4299572672,5.0532485857
O,0,0.2388605299,-3.5174107236,3.2014381082
C,0,1.6725668449,-3.1606456641,0.4535379452
C,0,2.5794660469,-3.9685331554,1.0959009344
H,0,2.6955829161,-4.0488931608,2.1742102287
C,0,3.3133470047,-4.6549602182,0.0842689904
H,0,4.1139387115,-5.380015899,0.2194336731
C,0,2.8108626879,-4.2187202821,-1.1066765987
H,0,3.0423244433,-4.4369357756,-2.1452678234
O,0,1.826182496,-3.3146986981,-0.8983201513
C,0,-0.3761936948,2.4665915397,2.6609556626
C,0,0.6684637266,2.5683433251,3.5432959113
H,0,1.5894850571,1.9902810044,3.4850925063
C,0,0.3102105784,3.5595007129,4.5007153438

	H,0,0.8973711415,3.9087810096,5.347489807
	C,0,-0.9330877178,3.9948245475,4.1378316967
	H,0,-1.6152381638,4.7351229766,4.5485603872
	O,0,-1.361174873,3.3440211183,3.0364497148
	C,0,-1.3417829125,2.6157502677,0.0741630155
	C,0,-2.176058774,2.5086099764,-1.0199689406
	H,0,-2.7998943689,1.6459842525,-1.2494333207
	C,0,-2.0564693663,3.7200544112,-1.7603522237
	H,0,-2.5788992949,3.993944722,-2.6755801112
	C,0,-1.1493003571,4.4882171104,-1.0886616809
	H,0,-0.7380761917,5.4811597704,-1.2454157175
	O,0,-0.716108475,3.8338720697,0.019870233
	C,0,-2.2183100555,0.6251941632,1.7216061166
	C,0,-3.5488931382,0.9348695085,1.5688732521
	H,0,-3.9343304599,1.8377010697,1.1004400957
	C,0,-4.293237494,-0.1309800111,2.1548723375
	H,0,-5.3753650719,-0.2333356763,2.2152308638
	C,0,-3.3676799947,-1.0143451052,2.6331179546
	H,0,-3.427806562,-1.9713006853,3.1454479514
	O,0,-2.1227473358,-0.5654510091,2.3801613121
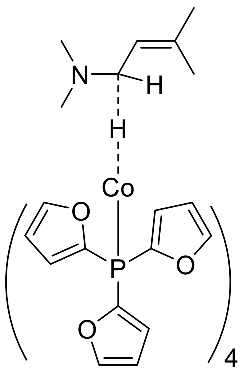<br>**[Co(L1)$_4$(1a')]**<br>**CH-Activation-TS**	Co,0,-0.0316338111,0.0514334095,0.0070931561
	P,0,1.8296481022,-0.4788644701,-1.072654648
	P,0,-0.7515411418,1.5312111658,-1.4208743979
	P,0,0.9565360186,0.8409118832,1.7651739576
	P,0,-0.720273639,-1.9698987921,0.3337771861
	C,0,2.900272382,0.8705798941,-1.6731090918
	C,0,4.0751286962,0.9336416259,-2.3779677447
	H,0,4.6770060343,0.0829880644,-2.690716278
	C,0,4.3326145126,2.3132524284,-2.613488786
	H,0,5.1775240876,2.7508268167,-3.1401937865
	C,0,3.2999764668,2.9919837058,-2.0281828179
	H,0,3.0569459985,4.0447201513,-1.9137301315
	O,0,2.4366922159,2.1294051441,-1.4617752567
	C,0,3.1885709504,-1.5200475902,-0.4214226011
	C,0,4.4132667138,-1.2363867342,0.1328256711
	H,0,4.8368095702,-0.2459094518,0.2647591726
	C,0,4.9994804029,-2.4839350749,0.4854514721
	H,0,5.9697962168,-2.6492653368,0.9473013854
	C,0,4.0938443271,-3.4403902553,0.1281467942

	H,0,4.074891875,-4.5253439603,0.1840275791
	O,0,3.0005091916,-2.8678795389,-0.4165908093
	C,0,1.5111987881,-1.4346934216,-2.5917431036
	C,0,2.2849215861,-2.1893743085,-3.4352780393
	H,0,3.3550639102,-2.3613084317,-3.3346847658
	C,0,1.4101373719,-2.7157009198,-4.426007731
	H,0,1.6649411221,-3.3633560948,-5.2617913833
	C,0,0.165237651,-2.2457233572,-4.1118895992
	H,0,-0.8186630536,-2.3668433551,-4.5561883394
	O,0,0.2182373084,-1.4729538123,-3.0123667874
	C,0,-1.2412230522,-2.347078377,2.0476958842
	C,0,-1.8784052993,-3.4081230979,2.6401780344
	H,0,-2.2519531371,-4.2852137318,2.116458506
	C,0,-1.9424174058,-3.1267048399,4.0331075445
	H,0,-2.3802652555,-3.74041002,4.8167979804
	C,0,-1.3336993118,-1.914230529,4.1959272677
	H,0,-1.1336434892,-1.2859190027,5.0593768107
	O,0,-0.9131030244,-1.4342943141,3.0087318334
	C,0,-0.3400104434,1.5640790859,-3.214099918
	C,0,-1.0703340708,1.840737498,-4.3456204948
	H,0,-2.1100773285,2.1553960176,-4.3801208046
	C,0,-0.1967712378,1.6506932954,-5.4539675565
	H,0,-0.4291729666,1.7779357686,-6.5084703403
	C,0,1.0033788181,1.2711105671,-4.9262228137
	H,0,1.9711167055,1.0142770658,-5.3483643516
	O,0,0.9218979984,1.218563677,-3.5817358012
	C,0,-0.275621813,3.2685041428,-1.0611581667
	C,0,0.1298597628,4.3347791536,-1.8202713521
	H,0,0.2715794005,4.3267949002,-2.8995740514
	C,0,0.3101157867,5.4262159119,-0.9210041204
	H,0,0.6283543033,6.4385599651,-1.1601279358
	C,0,-0.000965669,4.9466415546,0.3194593918
	H,0,-0.0212730694,5.3819253409,1.3145874657
	O,0,-0.3472013358,3.6443126024,0.242796715
	C,0,2.331616187,2.0672047902,1.6425154466
	C,0,2.5048591238,3.3847944066,1.9861955876
	H,0,1.774816155,4.018805849,2.4781542474
	C,0,3.8271278638,3.7430499131,1.5949794683
	H,0,4.3118887835,4.7093010675,1.7123628034

C,0,4.3736257201,2.6225890597,1.0431223384
H,0,5.3380336261,2.3911963998,0.5996468751
O,0,3.4803453209,1.6074138756,1.0721495478
C,0,0.21457396,-3.5344525949,0.0917236704
C,0,0.6948338937,-4.518028897,0.9175323264
H,0,0.6224960033,-4.528732323,2.002226776
C,0,1.2685068028,-5.5123018044,0.0732758644
H,0,1.7385354619,-6.4462963063,0.3740001822
C,0,1.0925401166,-5.0669033444,-1.2049070945
H,0,1.3491766931,-5.4627465938,-2.1832566557
O,0,0.4508560994,-3.8807547621,-1.2025888827
C,0,-2.1489770785,-2.5573784471,-0.6374615127
C,0,-2.7386936549,-2.0148816905,-1.7457254839
H,0,-2.5369330542,-1.0211037289,-2.1321357149
C,0,-3.6141801448,-3.0050142013,-2.2728557823
H,0,-4.2528570015,-2.928985054,-3.1504136868
C,0,-3.486483064,-4.0925008671,-1.4550487262
H,0,-3.9421194864,-5.0790555654,-1.4462319004
O,0,-2.6040719723,-3.8415839599,-0.4666957511
C,0,-2.5111712942,1.9833055398,-1.7427376838
C,0,-3.2008723567,3.1636925486,-1.6186769743
H,0,-2.8211132259,4.082480807,-1.1776199683
C,0,-4.4859258447,2.9544555903,-2.1950984593
H,0,-5.2998922677,3.6705840722,-2.2776240059
C,0,-4.4889275457,1.6661923631,-2.6445821669
H,0,-5.2209928533,1.0502574658,-3.157724866
O,0,-3.3075096246,1.0717470253,-2.3702533038
C,0,0.2366328781,1.6512620734,3.2506599745
C,0,0.1928656953,1.320743963,4.5821237984
H,0,0.5609295934,0.3946966227,5.016602933
C,0,-0.3615722048,2.4423114136,5.2654598375
H,0,-0.5456861852,2.5441709092,6.332383109
C,0,-0.6167787757,3.3772175542,4.3055638226
H,0,-1.0311416779,4.3826653608,4.322104565
O,0,-0.2650685505,2.9056384145,3.087932938
C,0,1.9468135126,-0.4196038525,2.6557521736
C,0,2.8905370962,-0.3528241898,3.6499916128
H,0,3.2195357577,0.5602929522,4.1434380523
C,0,3.3320916302,-1.6819785405,3.8919049544

	H,0,4.0777085874,-2.0168736148,4.6091781453<br>C,0,2.6369005087,-2.4681887071,3.0167133555<br>H,0,2.645009362,-3.5315418821,2.7943899953<br>O,0,1.8054036207,-1.7195764124,2.2673984006<br>H,0,-1.5610591542,0.2069280825,0.7342154164<br>C,0,-3.1077111388,0.4283782027,1.5833824453<br>H,0,-2.9963797303,-0.5063359564,2.1366459229<br>C,0,-4.1727308982,0.4445210444,0.6156643463<br>H,0,-4.3623451844,1.3946019166,0.102873248<br>C,0,-5.0304312296,-0.5795257319,0.3407398729<br>C,0,-6.139839622,-0.4090179902,-0.6439188576<br>H,0,-7.0911811675,-0.798775954,-0.2455052694<br>H,0,-5.9462272919,-0.98533402,-1.5676698368<br>H,0,-6.2928088014,0.6404427208,-0.9279675605<br>C,0,-4.9926471955,-1.9192825961,0.9950935214<br>H,0,-5.0566481843,-2.7238606814,0.2441574833<br>H,0,-5.8814391398,-2.0542287162,1.6357377635<br>H,0,-4.1025039359,-2.1054657254,1.6050583282<br>N,0,-2.8920062034,1.5270376067,2.4246506119<br>C,0,-3.3059138803,1.3279980492,3.7923890317<br>H,0,-2.8330133868,0.4254477737,4.2065419801<br>H,0,-4.4052983163,1.209711074,3.897768235<br>H,0,-2.994931667,2.1820983967,4.4088140168<br>C,0,-3.1507309998,2.8340268654,1.8967245011<br>H,0,-4.2255704129,3.0428103405,1.7094232039<br>H,0,-2.6060120308,2.9644735767,0.9536824032<br>H,0,-2.7752023935,3.5817087275,2.6048533541
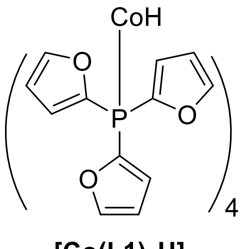<br>**[Co(L1)$_4$H]**	Co,0,0.185958594,-0.0013187056,0.0009051694<br>P,0,0.725246264,-1.0779338018,-1.7502951984<br>P,0,0.7271592851,2.0524887485,-0.057616499<br>P,0,0.7278959294,-0.9760861025,1.8089802285<br>P,0,-1.9621573937,-0.0017762538,0.0014831929<br>H,0,1.67050575,-0.0015151334,-0.0016283452<br>O,0,-0.1342648936,-2.1746826566,-4.1479401093<br>O,0,-0.1300236499,4.6789021546,0.1735463538<br>O,0,-0.1324375848,-2.4882215164,3.9676355086<br>C,0,-0.3018256431,-1.1584446737,-3.2484133005<br>C,0,-0.2987341413,3.3951360984,0.6134415184<br>C,0,-0.2988354615,-2.2284690285,2.6353743441

	C,0,-1.2162038744,-0.2622871538,-3.7357514956
	C,0,-1.2139992772,3.3774854955,1.6326041357
	C,0,-1.2123952542,-3.1032986622,2.1095533252
	H,0,-1.5304871979,0.6480835745,-3.2286337106
	H,0,-1.5301779975,2.4876759703,2.1738306363
	H,0,-1.5263493383,-3.1273749895,1.0677655464
	C,0,-1.6322902708,-0.7570238524,-5.0043444475
	C,0,-1.6291530261,4.7253079399,1.8281190524
	C,0,-1.6274756163,-3.947448357,3.178497593
	C,0,-0.9441730897,-1.9209957831,-5.1974109041
	C,0,-0.9400954128,5.4670022051,0.9113508391
	C,0,-0.941087927,-3.5226684854,4.2803288647
	O,0,3.3515673283,-0.3377647781,-1.7987905565
	O,0,3.349480506,1.7177797805,0.6190557977
	O,0,3.3529649829,-1.3909326524,1.1886639803
	C,0,2.2451313293,-0.4978945923,-2.5754528828
	C,0,2.245337615,2.4763956478,0.8601079361
	C,0,2.2469647832,-1.981661163,1.7185237841
	C,0,2.5298405397,-0.1064374851,-3.8588184429
	C,0,2.5301438587,3.3961636718,1.8371227259
	C,0,2.5302246678,-3.2890410548,2.0214235176
	H,0,1.8380545365,-0.1210203477,-4.6984049112
	H,0,1.8397241439,4.1352959238,2.2380671094
	H,0,1.8378937637,-4.0077779712,2.4549002268
	C,0,3.8892636268,0.3199021452,-3.8601981085
	C,0,3.8874401795,3.1809371239,2.21294282
	C,0,3.8888801409,-3.5051480608,1.651658895
	C,0,4.3340968868,0.1559979335,-2.5804306045
	C,0,4.3307907725,2.1483052707,1.4388240718
	C,0,4.3343716593,-2.3159378509,1.1515797227
	O,0,0.4195879504,-3.6211711395,-0.7781481983
	O,0,0.4207355933,2.4862467764,-2.7445611953
	O,0,0.4147367513,1.1274990983,3.5354545983
	C,0,1.2208425166,-2.8225623319,-1.5412375554
	C,0,1.2278746433,2.7383838624,-1.6736092294
	C,0,1.2278285281,0.0806691751,3.2113495307
	C,0,2.3337325618,-3.5293811067,-1.9196215429
	C,0,2.3468384815,3.4080955102,-2.0984318336
	C,0,2.3512167458,0.121020641,3.9972277664

	H,0,3.1505539299,-3.1469042944,-2.5282055514<br>H,0,3.1685592446,3.7348308921,-1.4645346241<br>H,0,3.1783598776,-0.5846082096,3.9573586438<br>C,0,2.1977094214,-4.8333268041,-1.3650119182<br>C,0,2.2090561901,3.5825927027,-3.5042775538<br>C,0,2.2095402869,1.2486389564,4.8545089588<br>C,0,1.0162660149,-4.8275881596,-0.6821273381<br>C,0,1.0197948266,3.0022132781,-3.8382312293<br>C,0,1.0144937637,1.8197895186,4.5266080806<br>O,0,-2.2405601058,-2.3954289103,-1.2332063705<br>O,0,-2.2517970591,2.2600991566,-1.4603170231<br>O,0,-2.2516941485,0.1372179427,2.6896501937<br>C,0,-2.8401133443,-1.1841431608,-1.0694836124<br>C,0,-2.8437911523,1.5150190113,-0.4867417567<br>C,0,-2.8427431479,-0.3385464686,1.5594477801<br>C,0,-3.9403936965,-1.1085628507,-1.8839125971<br>C,0,-3.9420175566,2.1824918343,-0.0093219068<br>C,0,-3.937060192,-1.0902993825,1.9013771311<br>H,0,-4.6035375695,-0.2506876904,-1.9764446168<br>H,0,-4.5995407662,1.8354382515,0.7853163749<br>H,0,-4.5930031221,-1.6095397995,1.2054415016<br>C,0,-4.0111380382,-2.3467641659,-2.5848760923<br>C,0,-4.019751858,3.4056660207,-0.7353499148<br>C,0,-4.0129391473,-1.0710059702,3.3238739492<br>C,0,-2.948810999,-3.0860054307,-2.1506401395<br>C,0,-2.9632156851,3.397764982,-1.5996911872<br>C,0,-2.9594957996,-0.3124533614,3.7464734096<br>H,0,0.475857237,2.692975652,4.8849053064<br>H,0,0.4864657106,-5.5703571256,-0.0916778265<br>H,0,0.4857774472,2.8695765017,-4.7753338285<br>H,0,2.9086035015,1.5993901864,5.6099578817<br>H,0,2.8912890211,-5.6664035162,-1.4508443277<br>H,0,2.9062613882,4.0665664208,-4.1840738873<br>H,0,4.4581585594,3.7157591258,2.9682873298<br>H,0,4.4594213325,-4.4269355935,1.7356967609<br>H,0,4.4606346127,0.7082470969,-4.6998633684<br>H,0,5.2768219493,1.6198693763,1.3609911606<br>H,0,5.2821109781,-1.9823764461,0.7384708166<br>H,0,5.2820980501,0.3447550474,-2.0845759207

	H,0,-0.9337807795,-2.6607418245,-5.9928832771<br>H,0,-0.9287734653,6.5239119756,0.660530388<br>H,0,-0.930998484,-3.833551321,5.3211994211<br>H,0,-2.3572316013,-0.3149742099,-5.6838037067<br>H,0,-2.3542898443,5.0985522642,2.5475049711<br>H,0,-2.3510626872,-4.7584053994,3.1411502688<br>H,0,-2.569856113,-4.0758146922,-2.3899907828<br>H,0,-2.5903491998,4.0977767411,-2.3423476553<br>H,0,-2.5866940724,-0.0155723654,4.7229428729<br>H,0,-4.7455479181,-2.6467702077,-3.3283386386<br>H,0,-4.7549497223,4.1985483317,-0.6217201518<br>H,0,-4.7448629337,-1.5684239641,3.9555508829
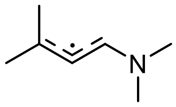<br>**Allyl Radical**	C,0,2.2681449686,-0.6371532642,-0.300221906<br>C,0,0.9745663901,-0.2376891572,-0.6496161371<br>H,0,0.8927722662,0.6284162908,-1.3163840046<br>C,0,2.4973983724,-1.8015238292,0.6066678445<br>H,0,3.5341259304,-1.8389291323,0.9701806613<br>H,0,2.3033939172,-2.77074665,0.1101436425<br>H,0,1.8408997409,-1.7784491971,1.4922846632<br>C,0,3.4630714132,0.0422941314,-0.8743003729<br>H,0,4.1415829576,0.4239053795,-0.0903040202<br>H,0,3.1911295497,0.8911953226,-1.5166132858<br>H,0,4.0811513026,-0.6421469654,-1.4846613245<br>C,0,-0.2005893544,-0.8515734028,-0.2354997227<br>H,0,-0.1653348471,-1.7566472081,0.3790298499<br>N,0,-1.4811459101,-0.3996130187,-0.480262035<br>C,0,-2.5646187046,-1.343307335,-0.4236918244<br>H,0,-2.3754165169,-2.0815214984,0.3670311232<br>H,0,-2.715174578,-1.9019957242,-1.3689174375<br>H,0,-3.5086198642,-0.8318629271,-0.1853816577<br>C,0,-1.6763354108,0.7638288231,-1.2979058521<br>H,0,-1.0606602874,1.5988723011,-0.9333211393<br>H,0,-2.7277780978,1.076046072,-1.2515887368<br>H,0,-1.4176742376,0.6017439891,-2.3636293279

13 NMR Spectra

13.1 Furan- and Benzofuran-Phosphines

^{1}H NMR, ^{13}C NMR and ^{31}P NMR spectrum of chlorodi(furan-2-yl)phosphane

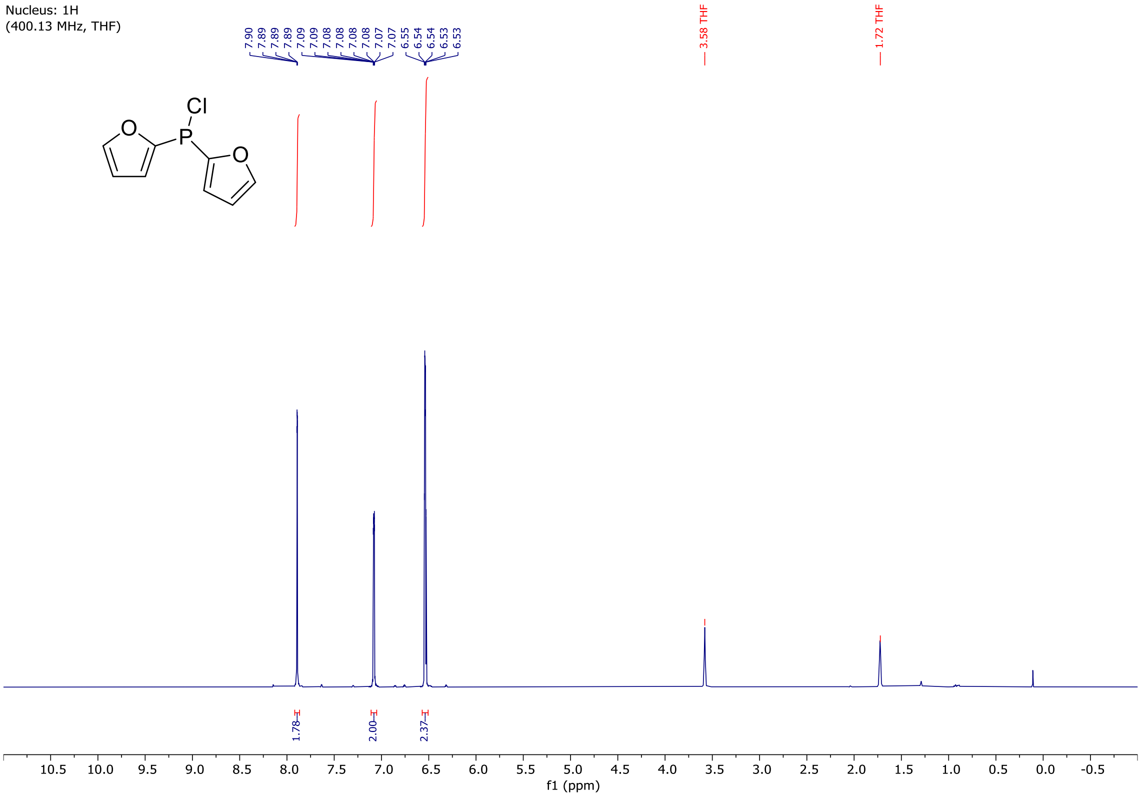

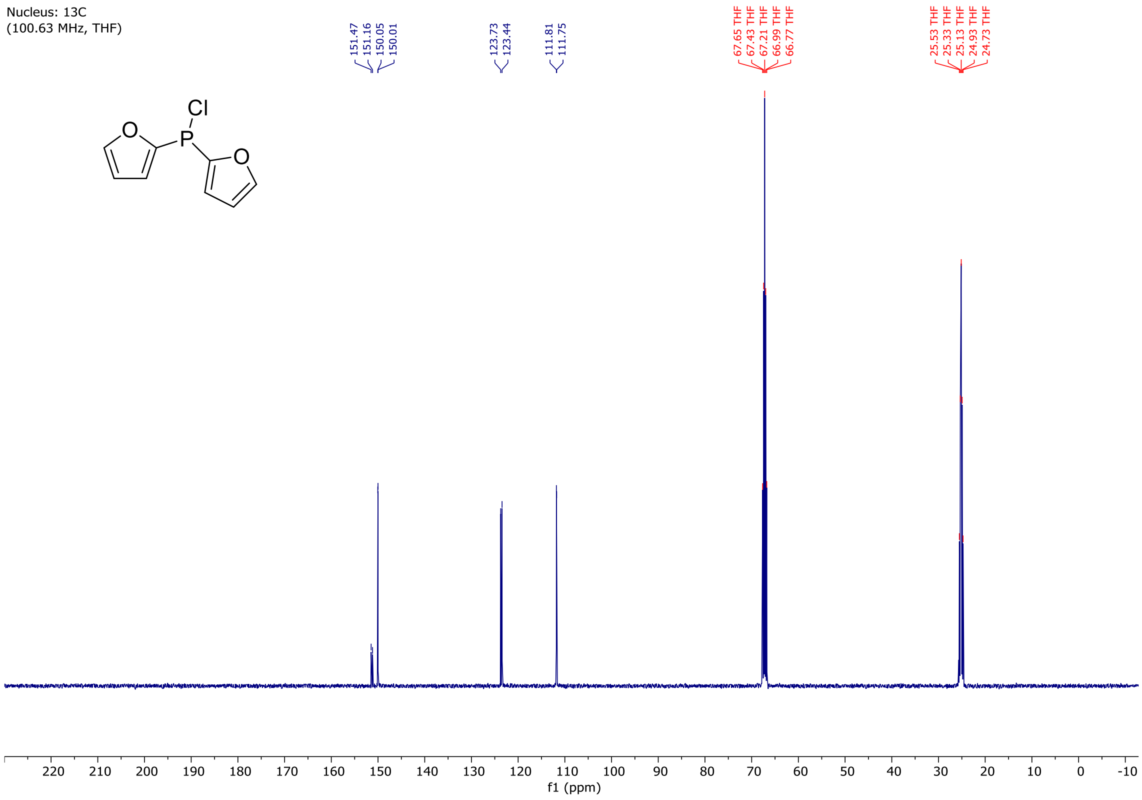
Nucleus: 13C
(100.63 MHz, THF)
151.47
151.16
150.05
150.01
123.73
123.44
111.81
111.75
67.65 THF
67.43 THF
67.21 THF
66.99 THF
66.77 THF
25.53 THF
25.33 THF
25.13 THF
24.93 THF
24.73 THF
Cl
P
O
f1 (ppm)

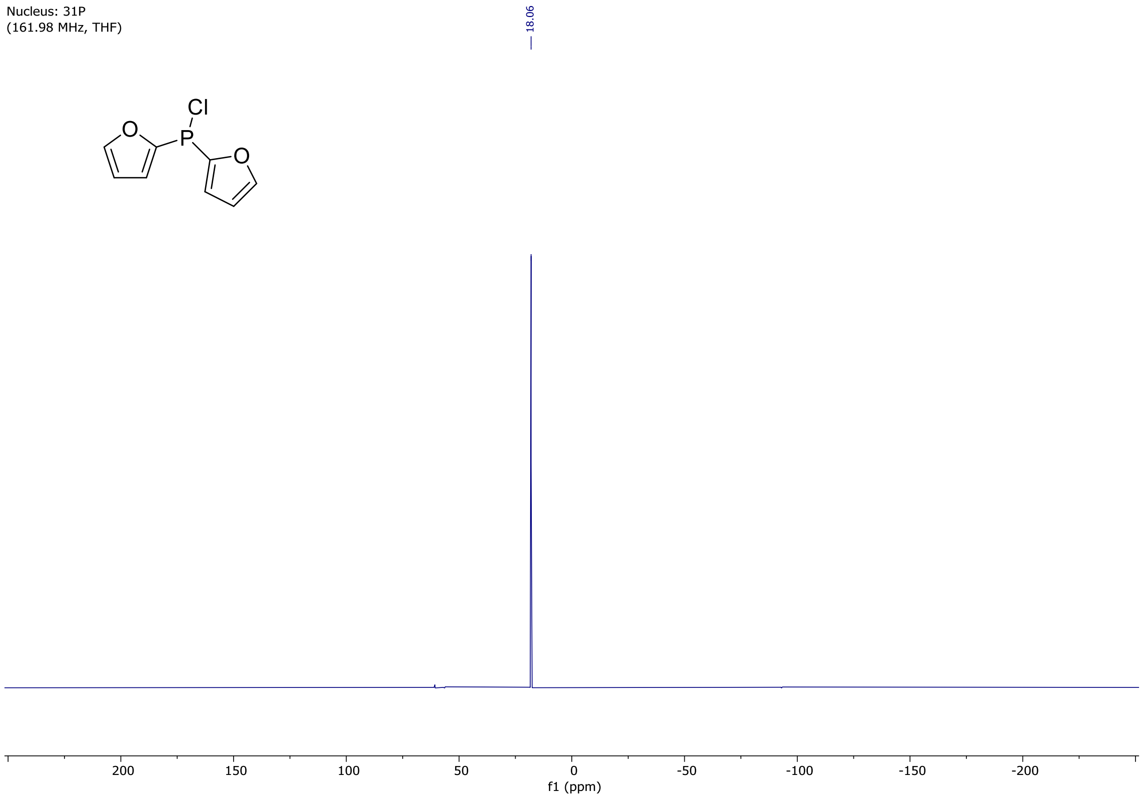
Nucleus: 31P
(161.98 MHz, THF)
18.06
Cl
P
O
f1 (ppm)

^{1}H NMR, ^{13}C NMR and ^{31}P NMR spectrum of **L5**

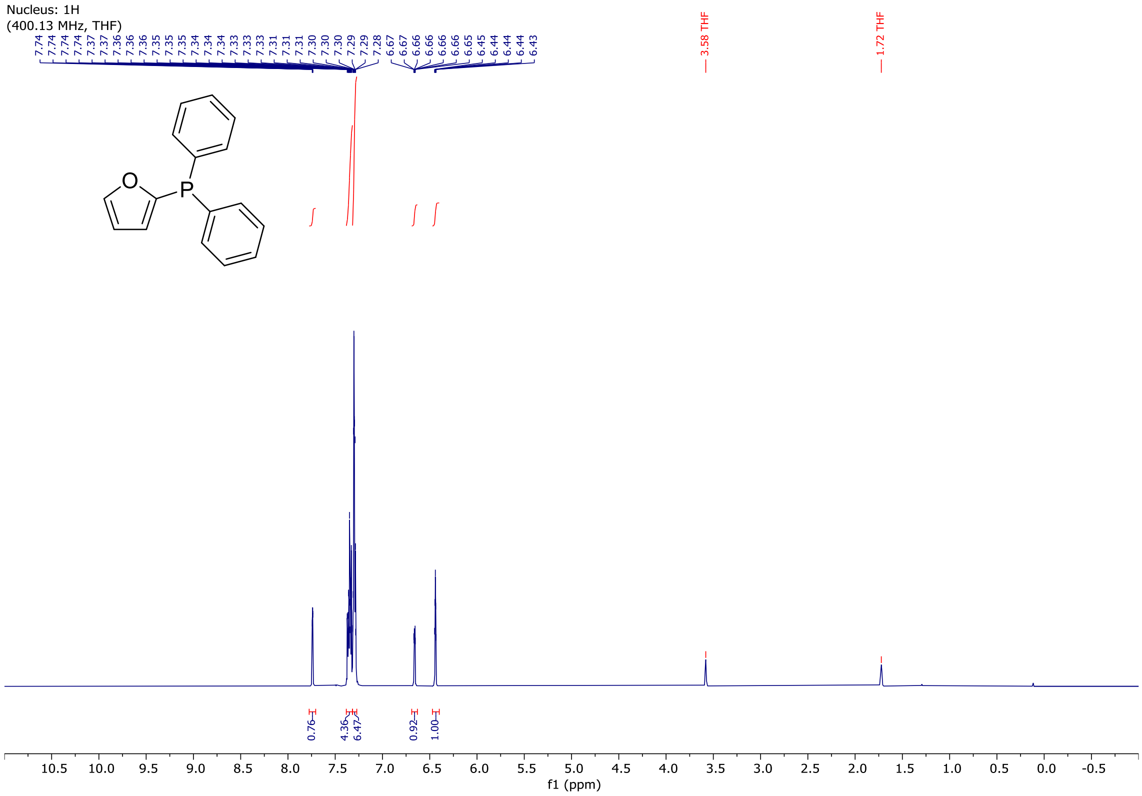

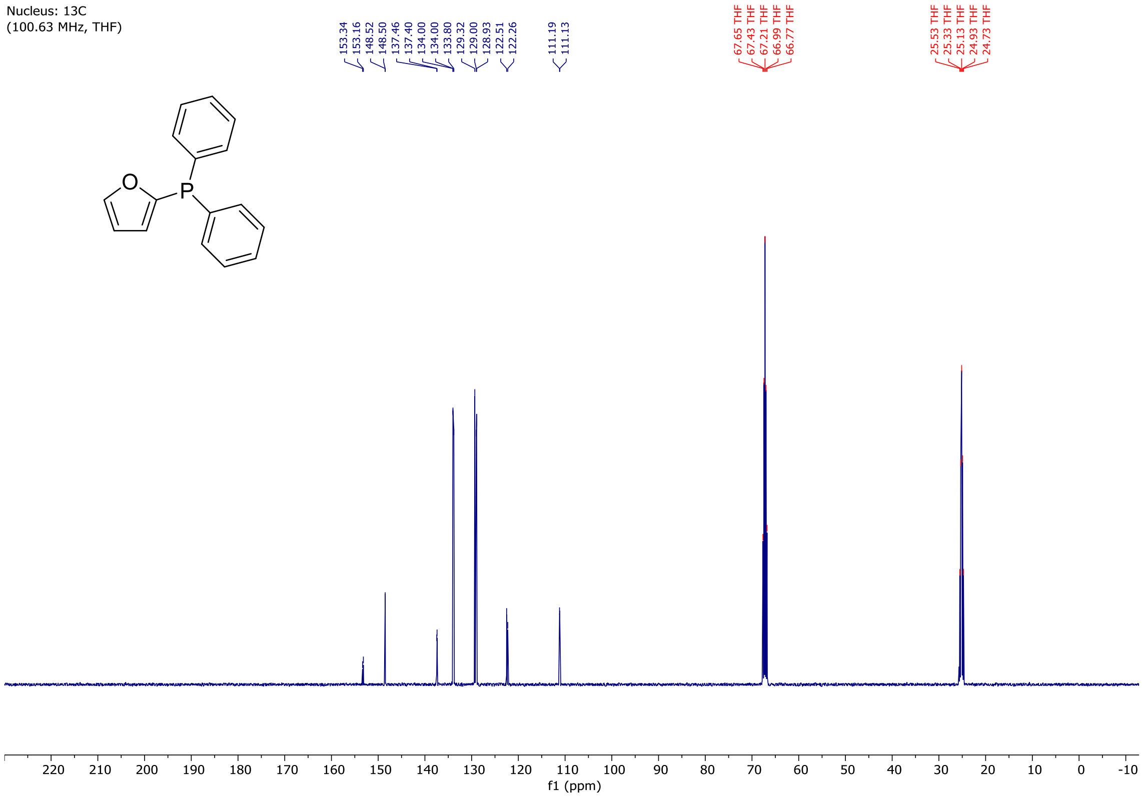

Nucleus: 31P
(121.52 MHz, THF)

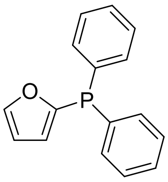

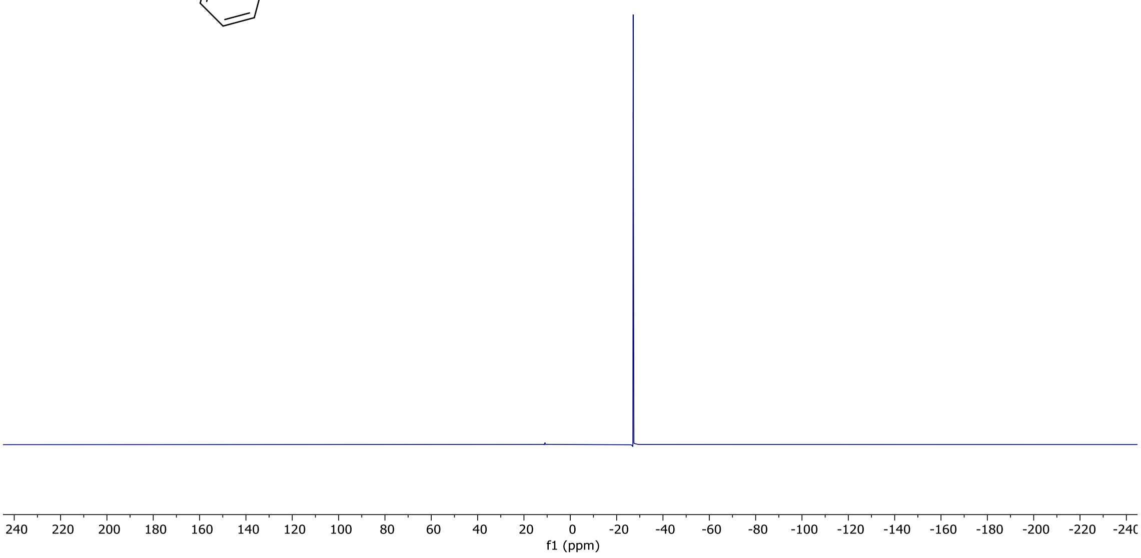

^{1}H NMR, ^{13}C NMR and ^{31}P NMR spectrum of **L6**

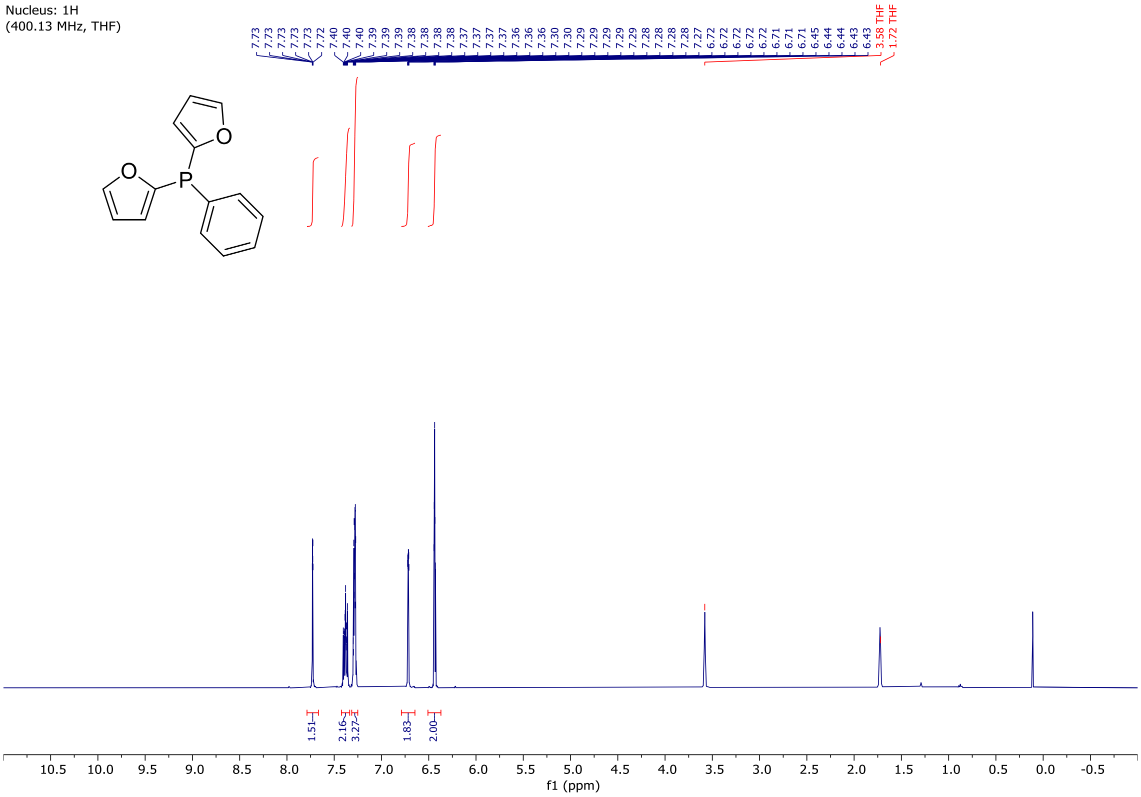

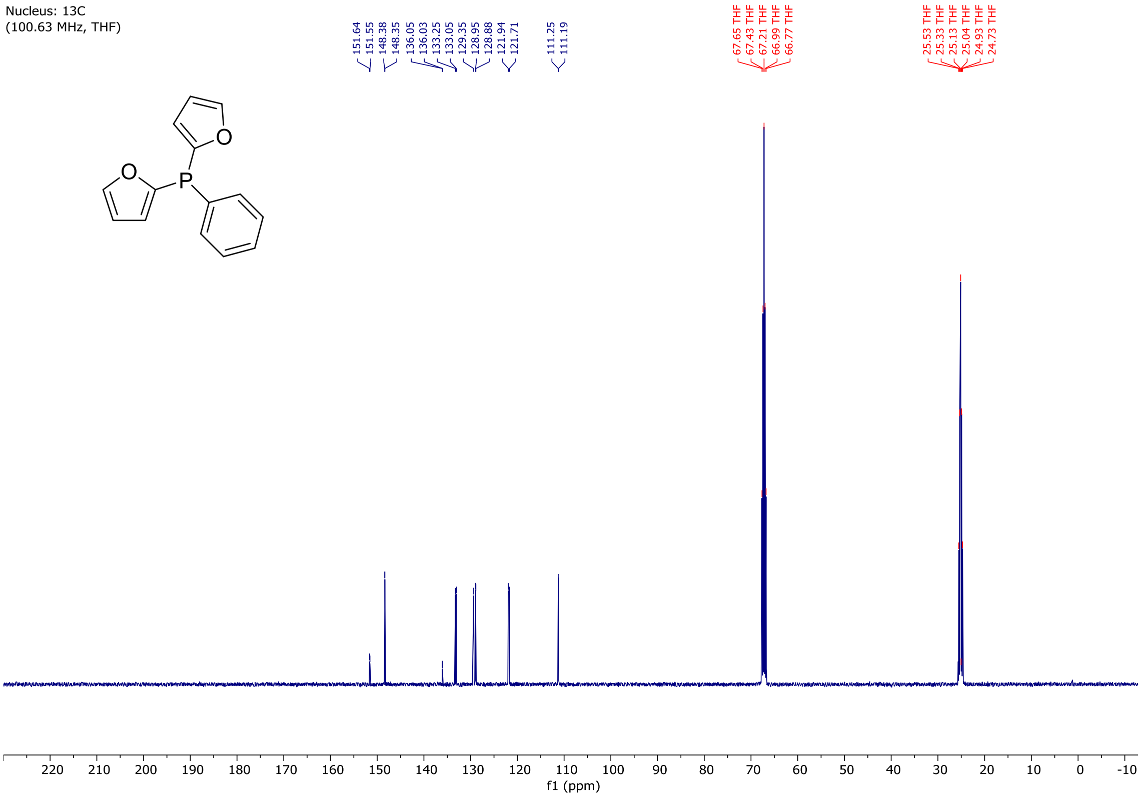

Nucleus: 31P
(161.98 MHz, THF)

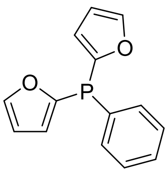

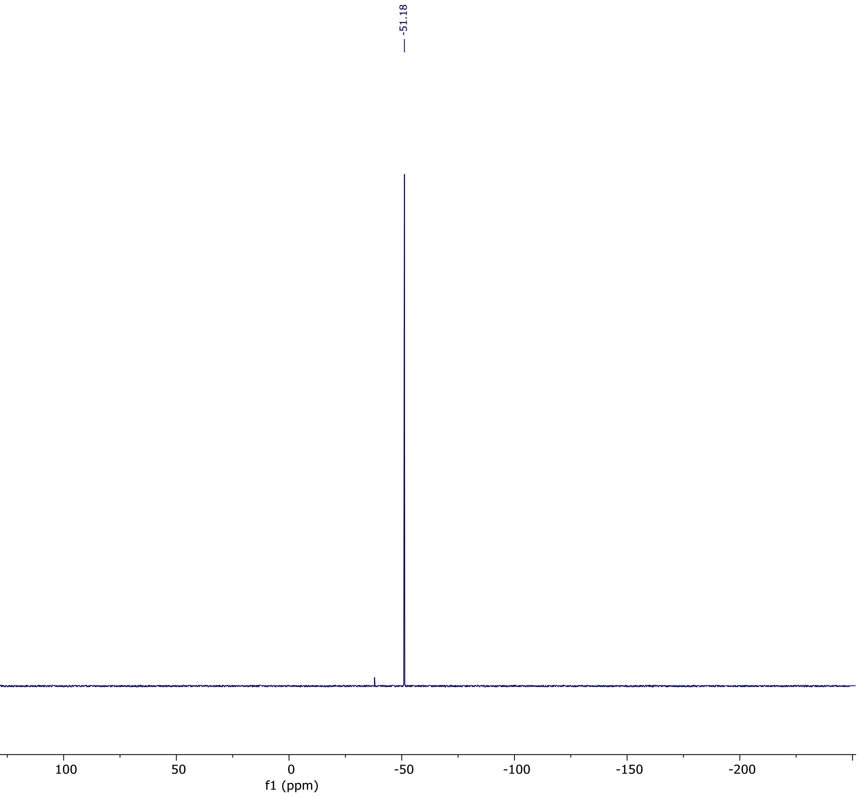

^{1}H NMR, ^{13}C NMR and ^{31}P NMR spectrum of **L7**

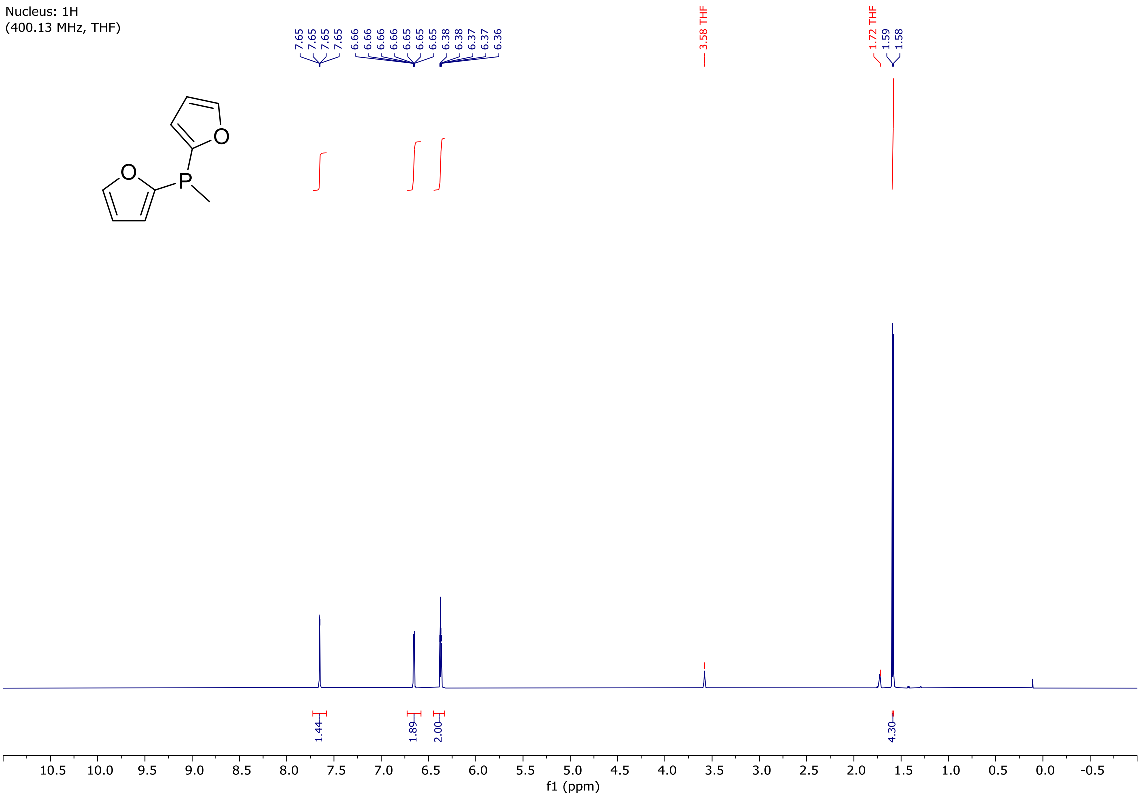

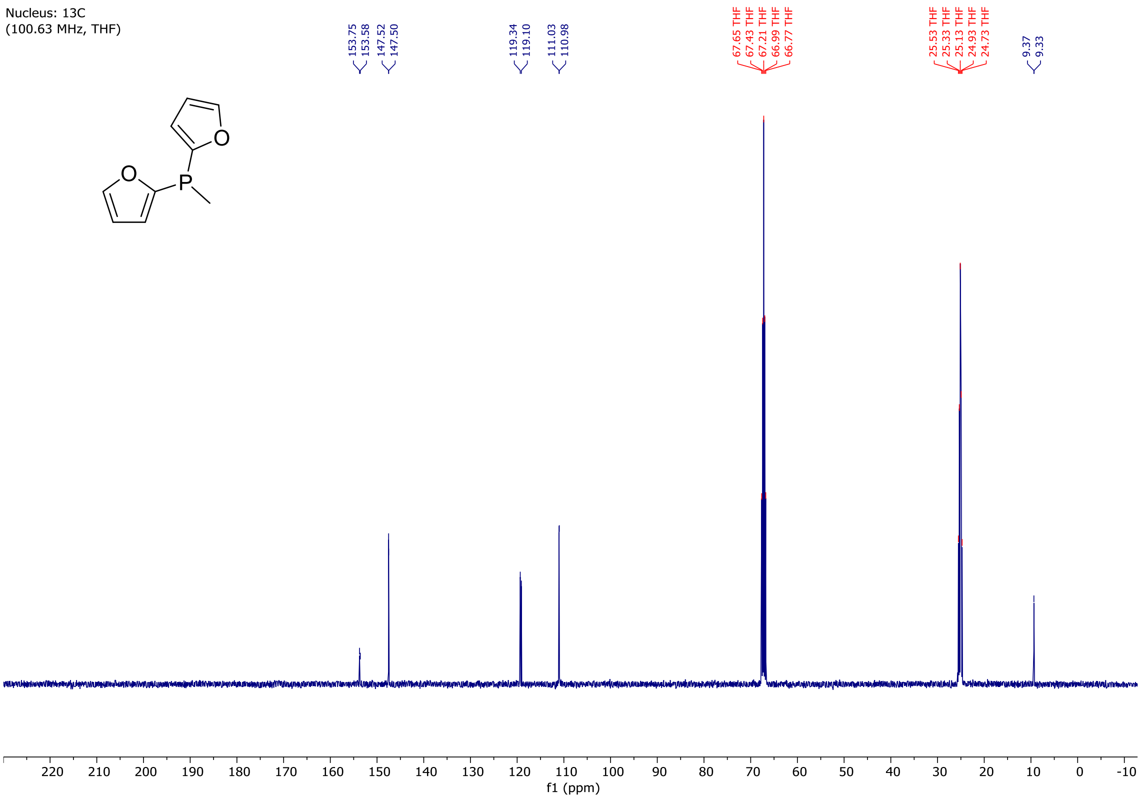

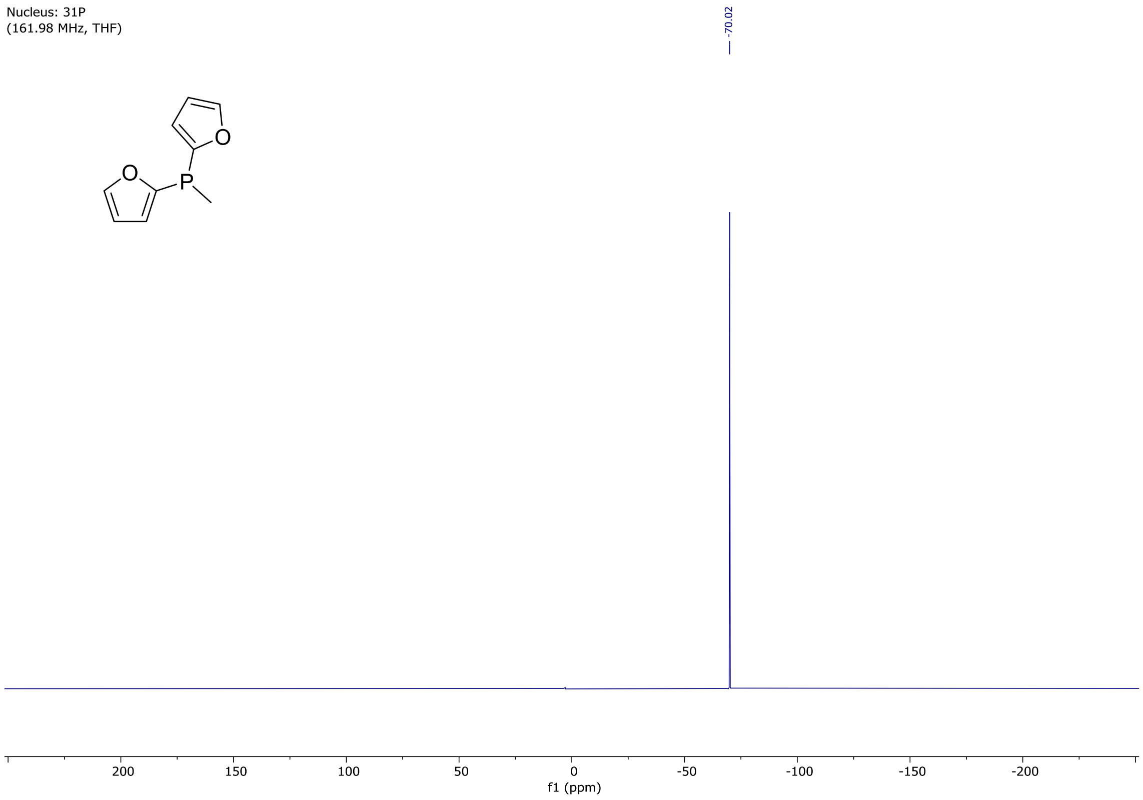
Nucleus: 31P
(161.98 MHz, THF)
-70.02
O
P
O
200
150
100
50
0
-50
-100
-150
-200
f1 (ppm)

^{1}H NMR, ^{13}C NMR and ^{31}P NMR spectrum of **L8**

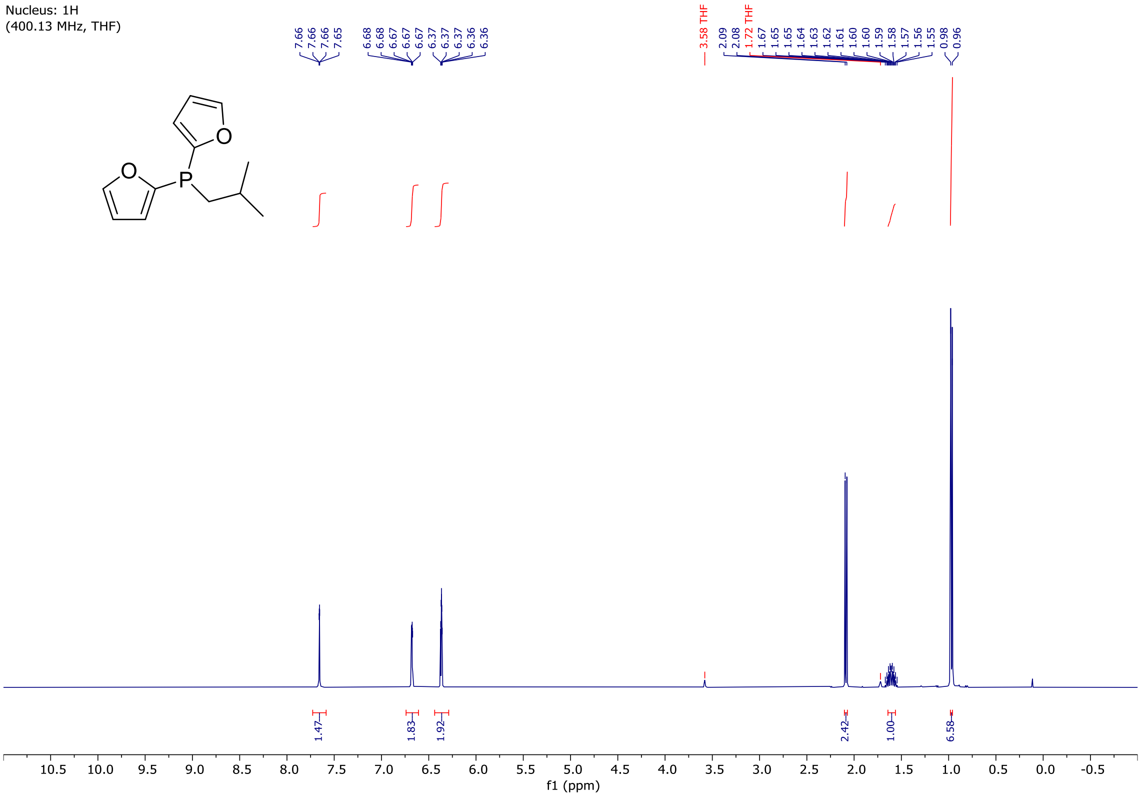

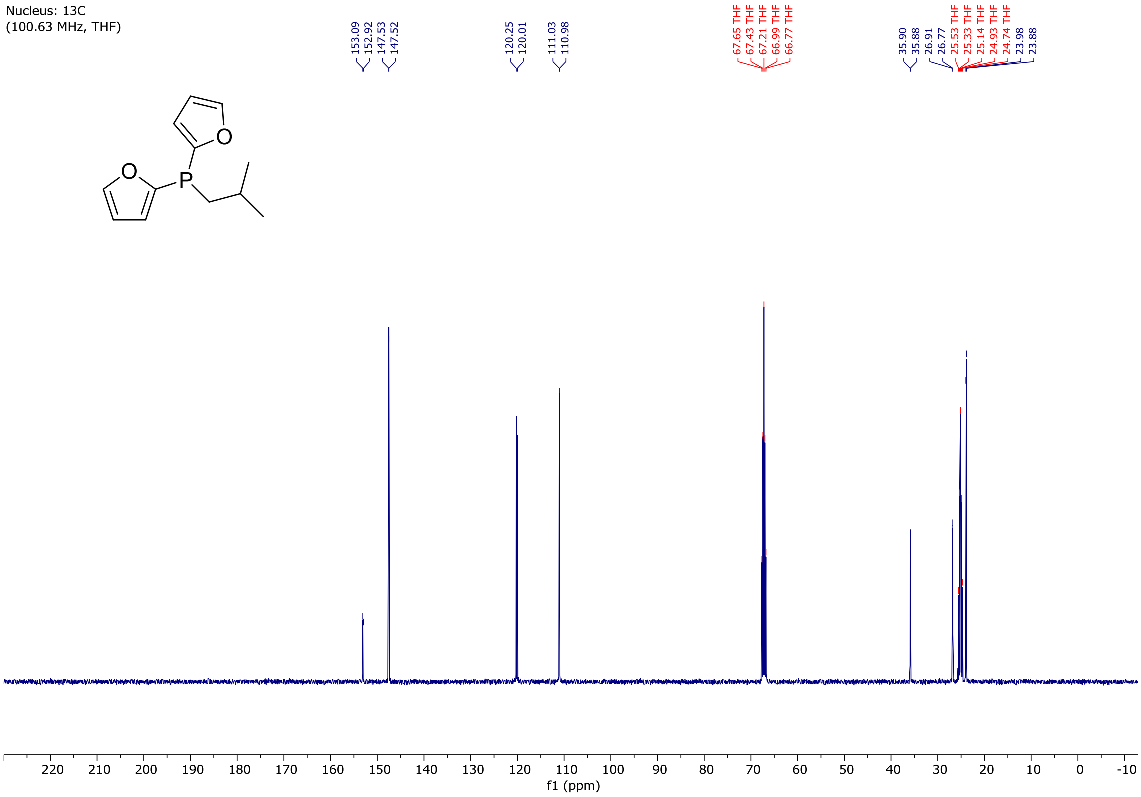

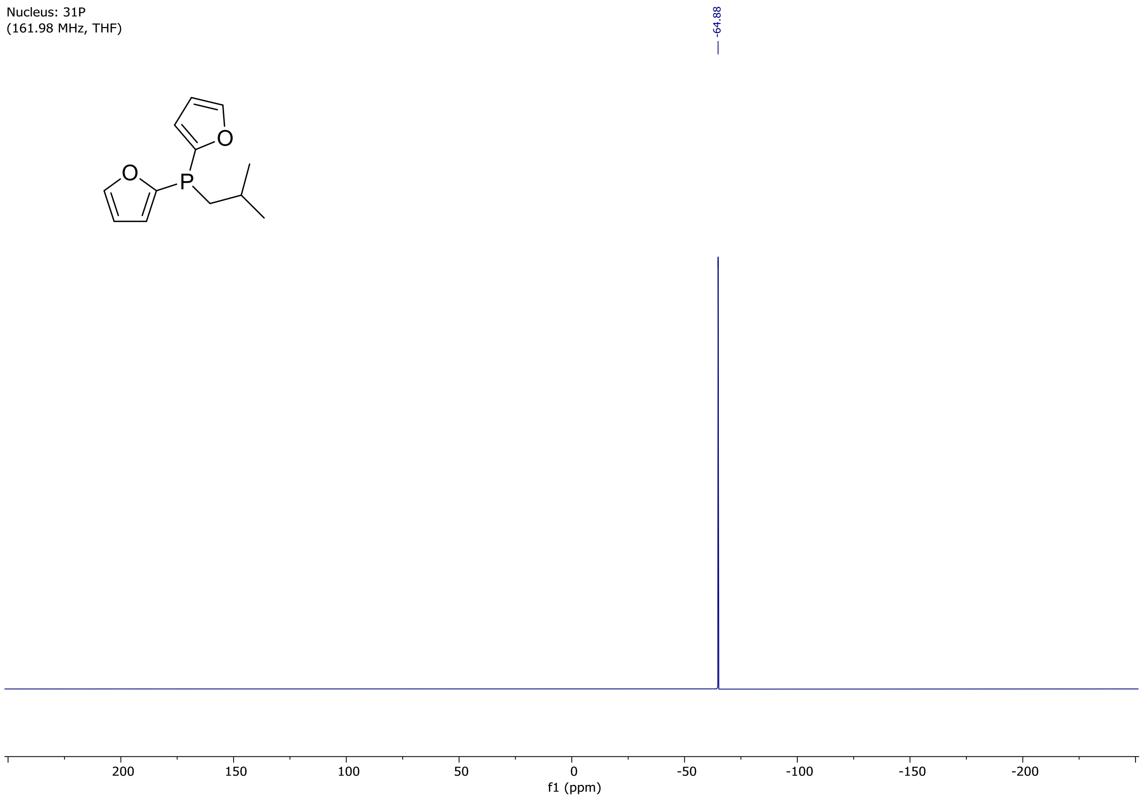
Nucleus: 31P
(161.98 MHz, THF)
-64.88
200
150
100
50
0
-50
-100
-150
-200
f1 (ppm)

^{1}H NMR, ^{13}C NMR and ^{31}P NMR spectrum of **L9**

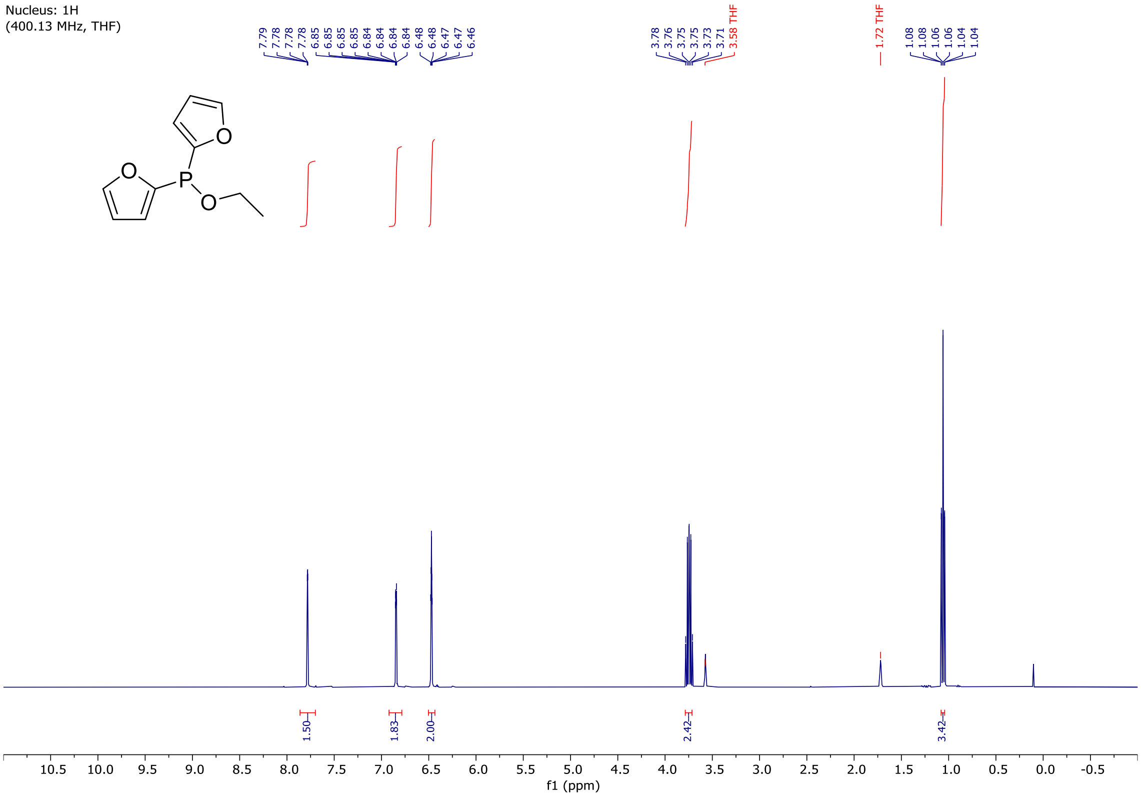

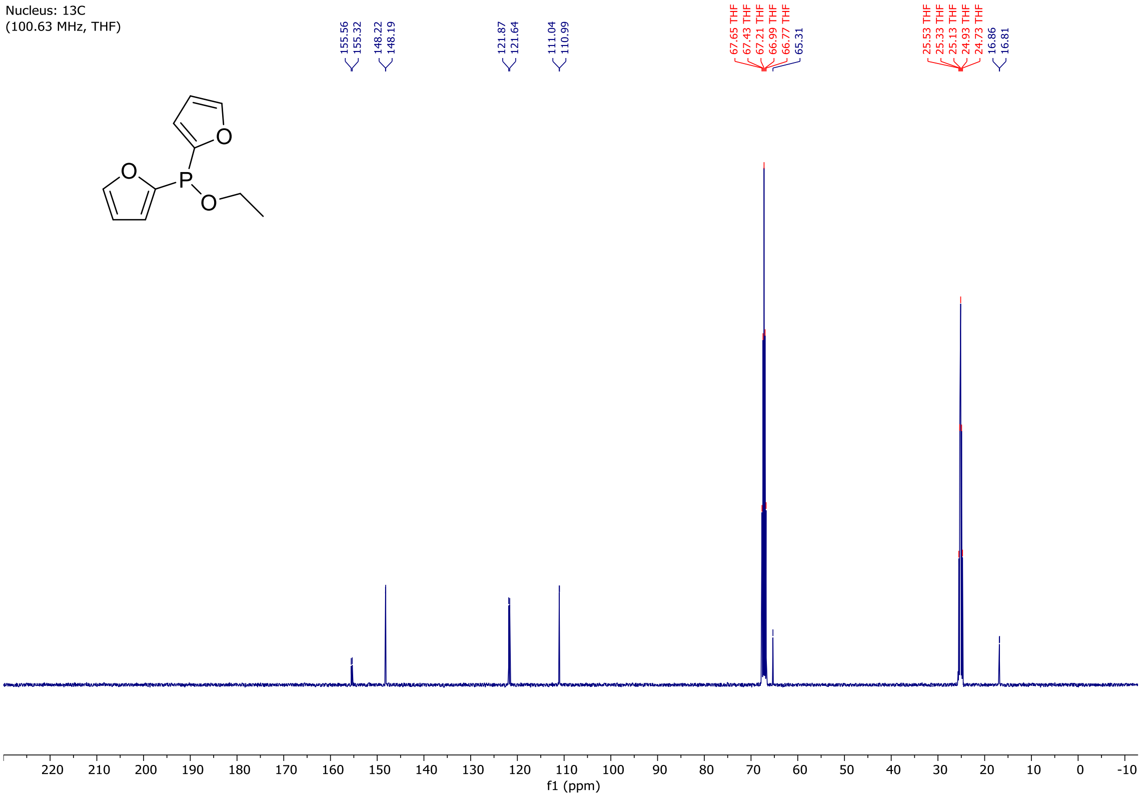

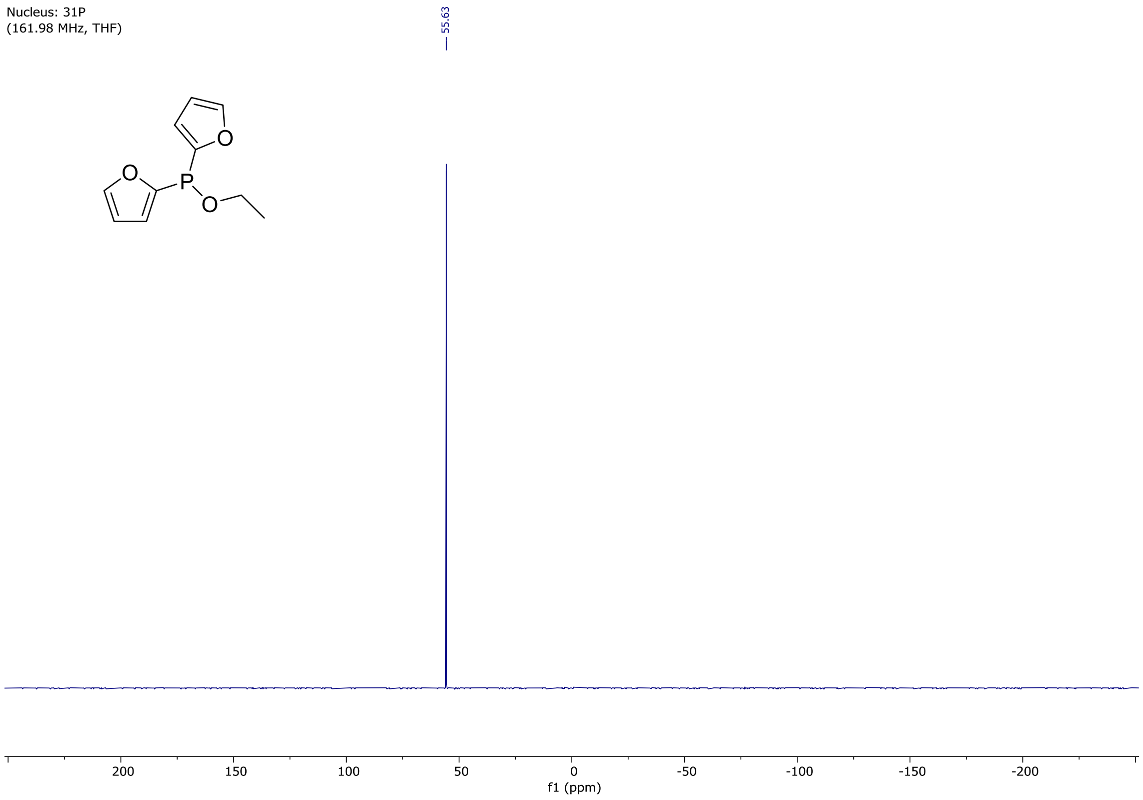
Nucleus: 31P
(161.98 MHz, THF)
55.63
200
150
100
50
0
-50
-100
-150
-200
f1 (ppm)

^{1}H NMR, ^{13}C NMR and ^{31}P NMR spectrum of **L10**

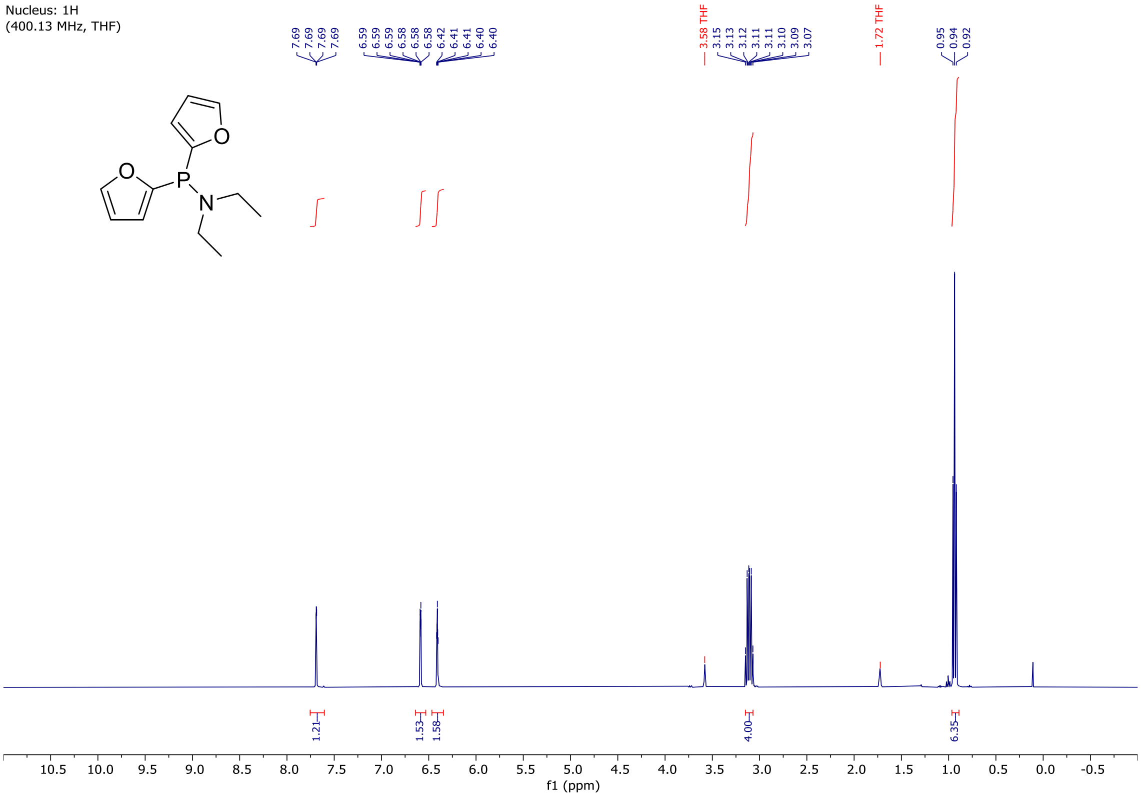

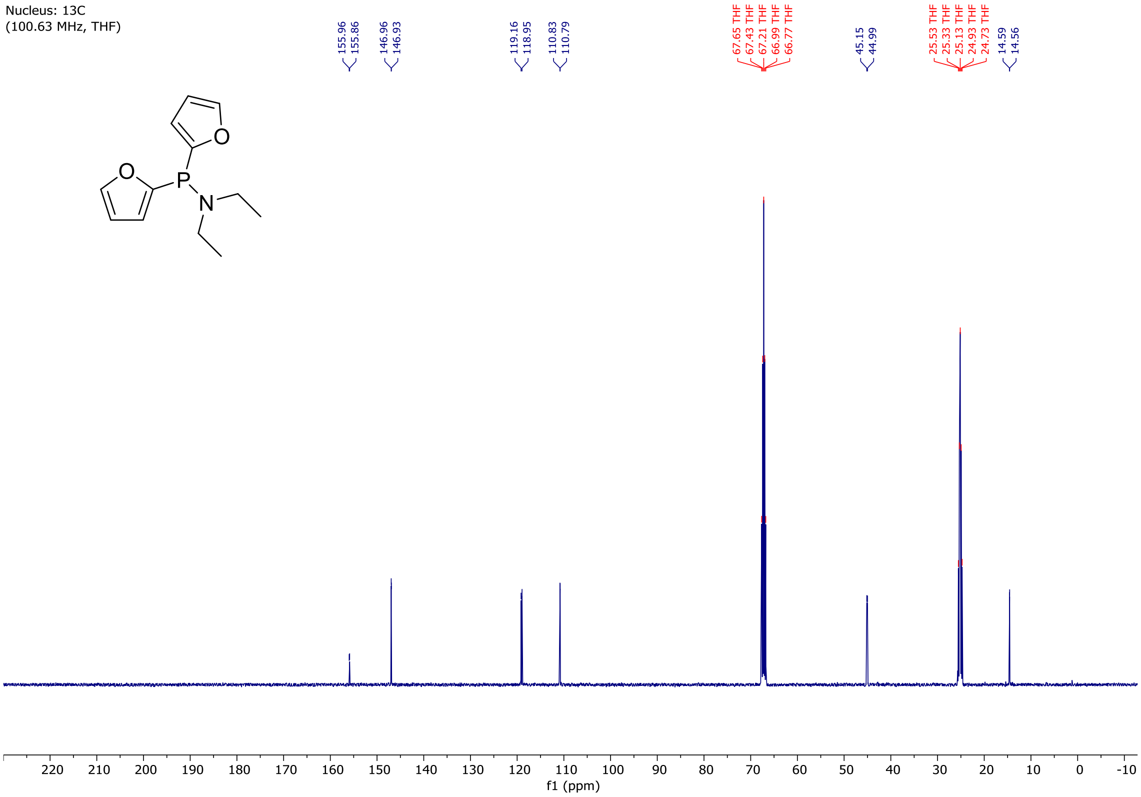

Nucleus: 31P
(161.98 MHz, THF)

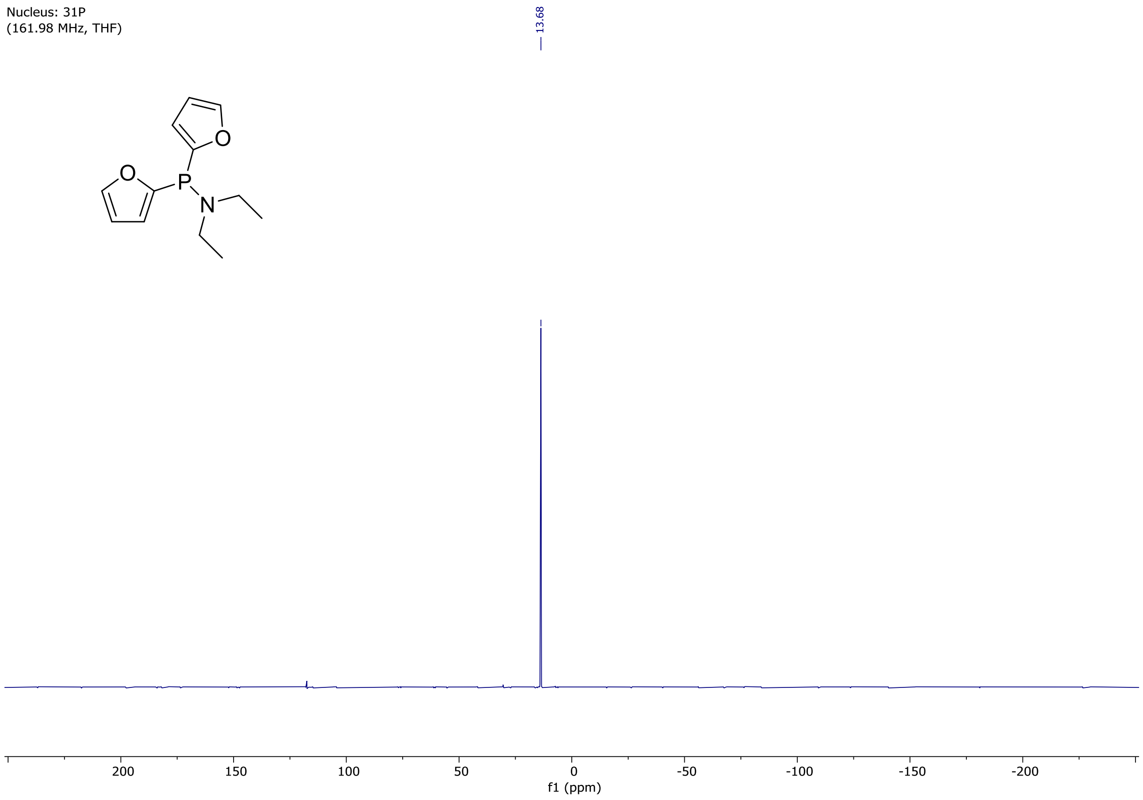

^{1}H NMR, ^{13}C NMR and ^{31}P NMR spectrum of **L11**

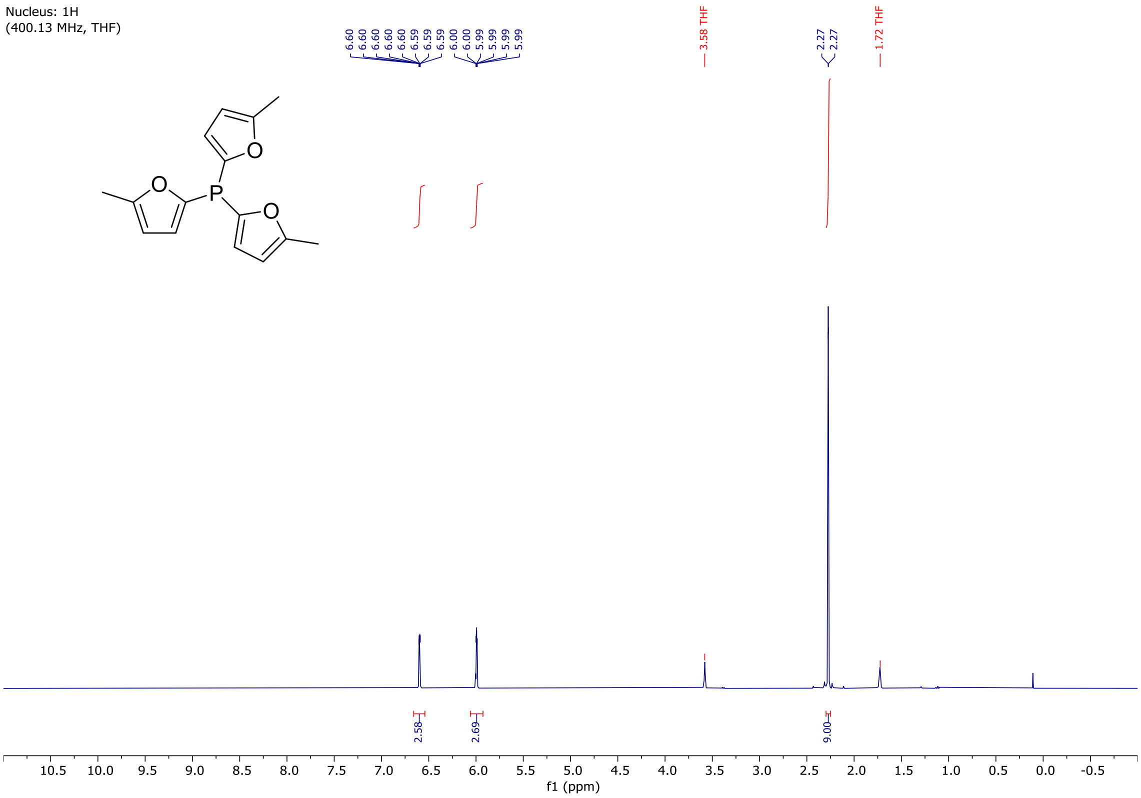

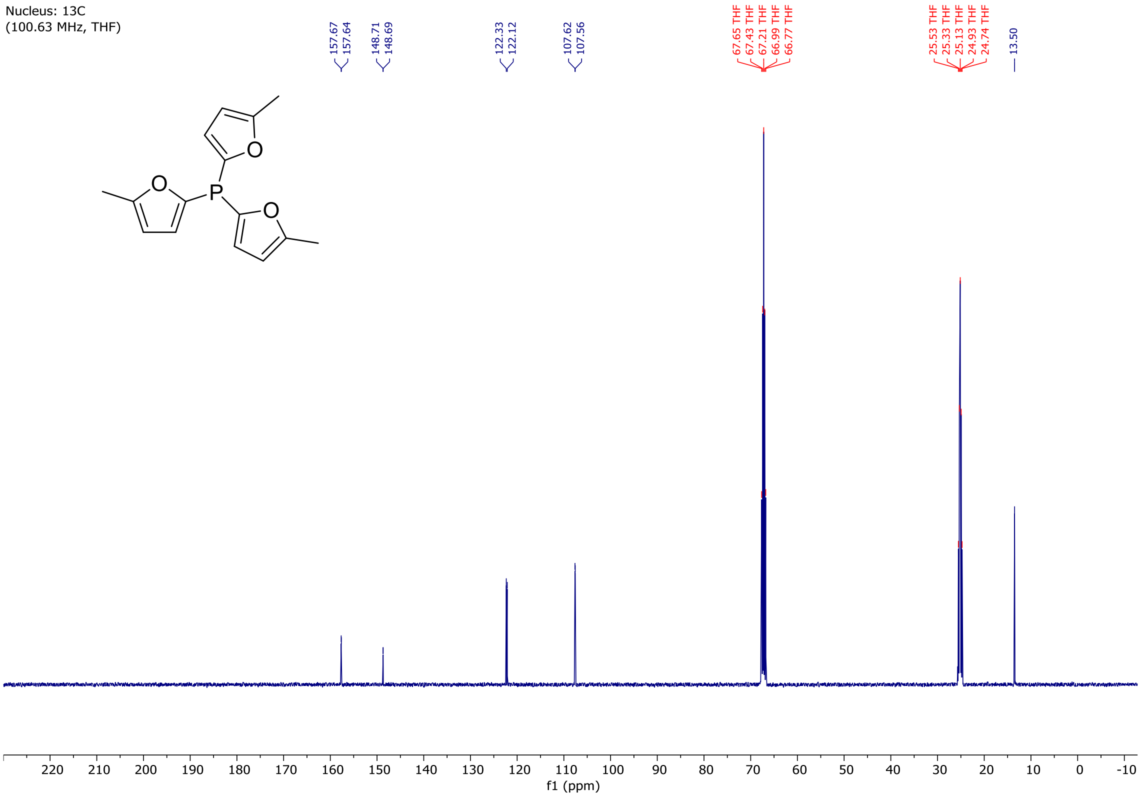

Nucleus: 31P
(161.98 MHz, THF)

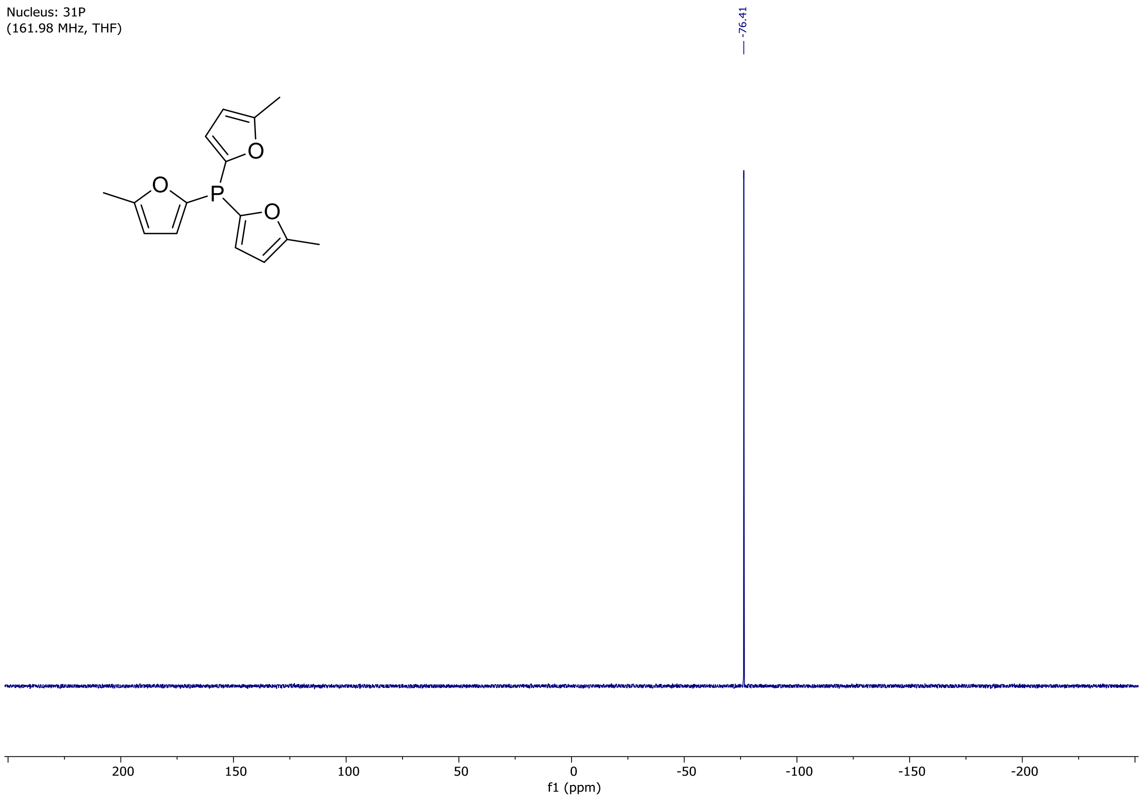

^{1}H NMR, ^{13}C NMR and ^{31}P NMR spectrum of **L12**

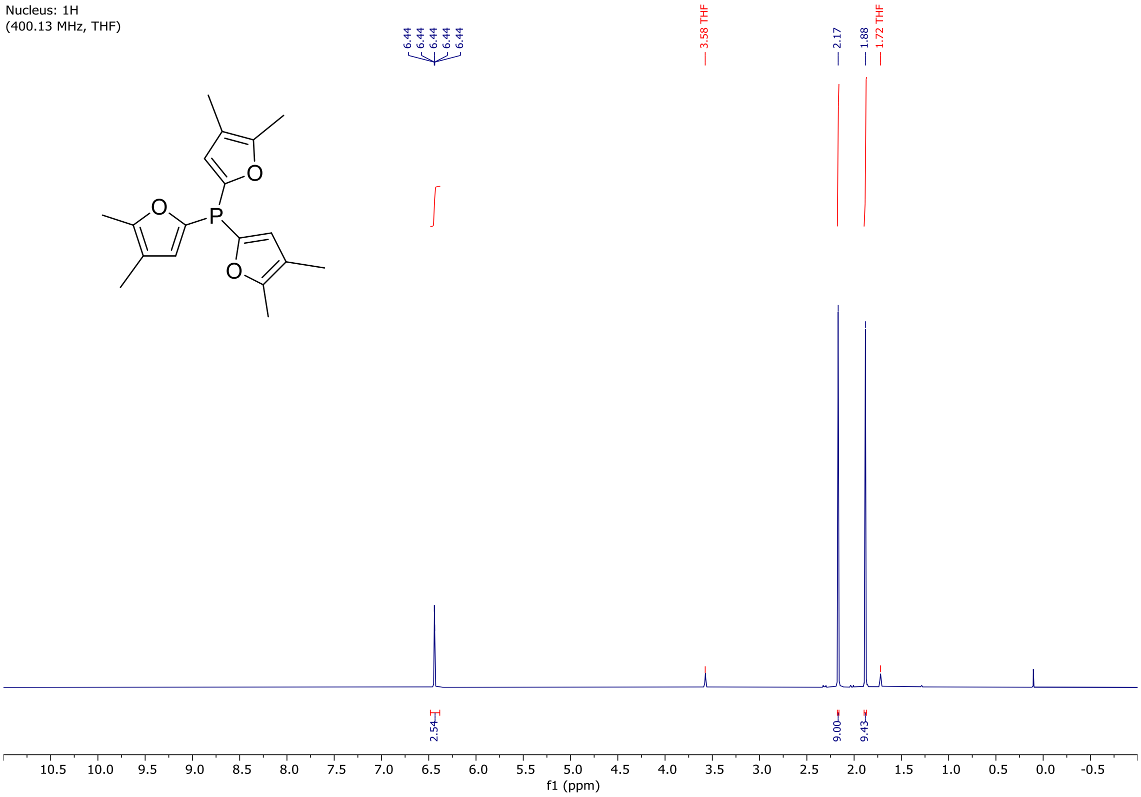

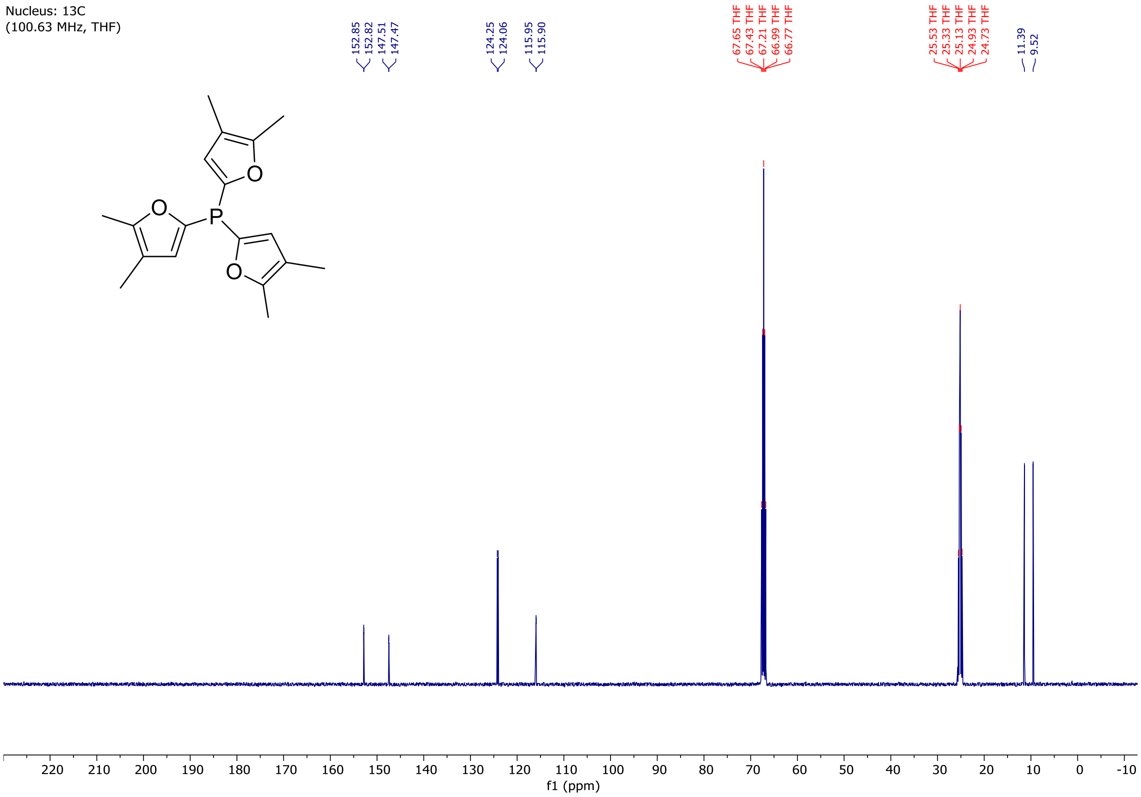

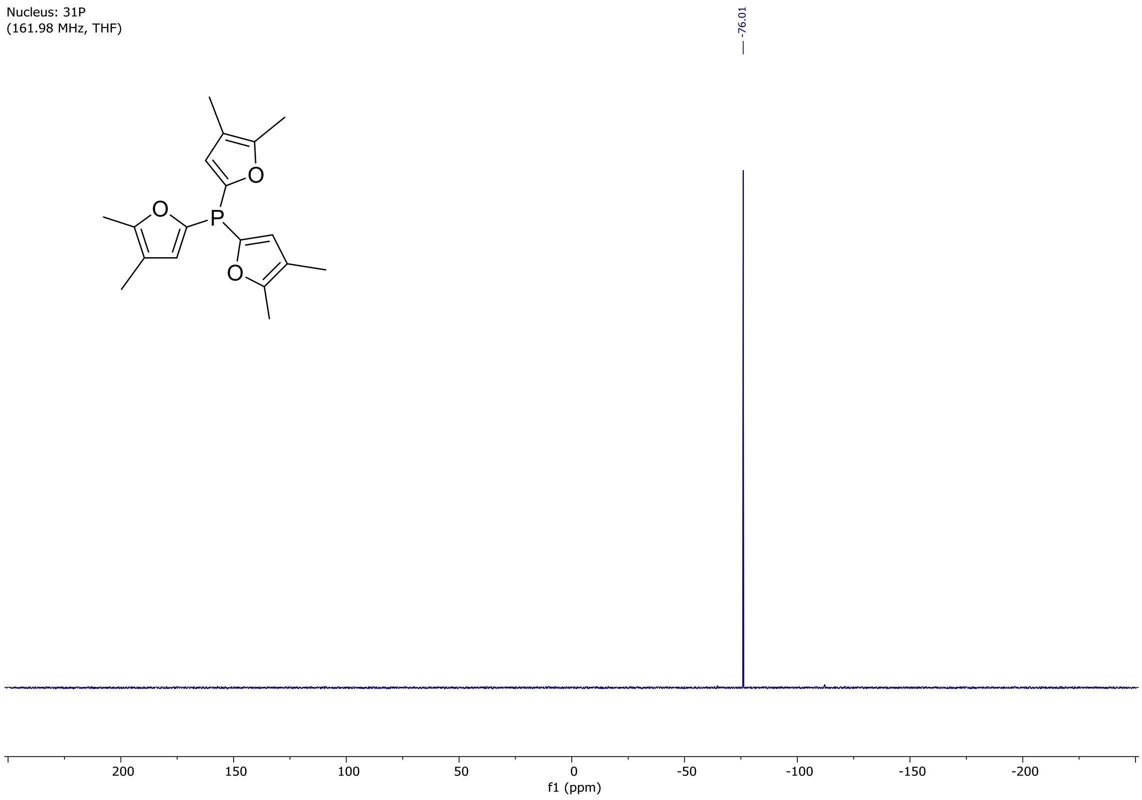
Nucleus: 31P
(161.98 MHz, THF)
-76.01
200
150
100
50
0
-50
-100
-150
-200
f1 (ppm)

^{1}H NMR, ^{13}C NMR and ^{31}P NMR spectrum of **L13**

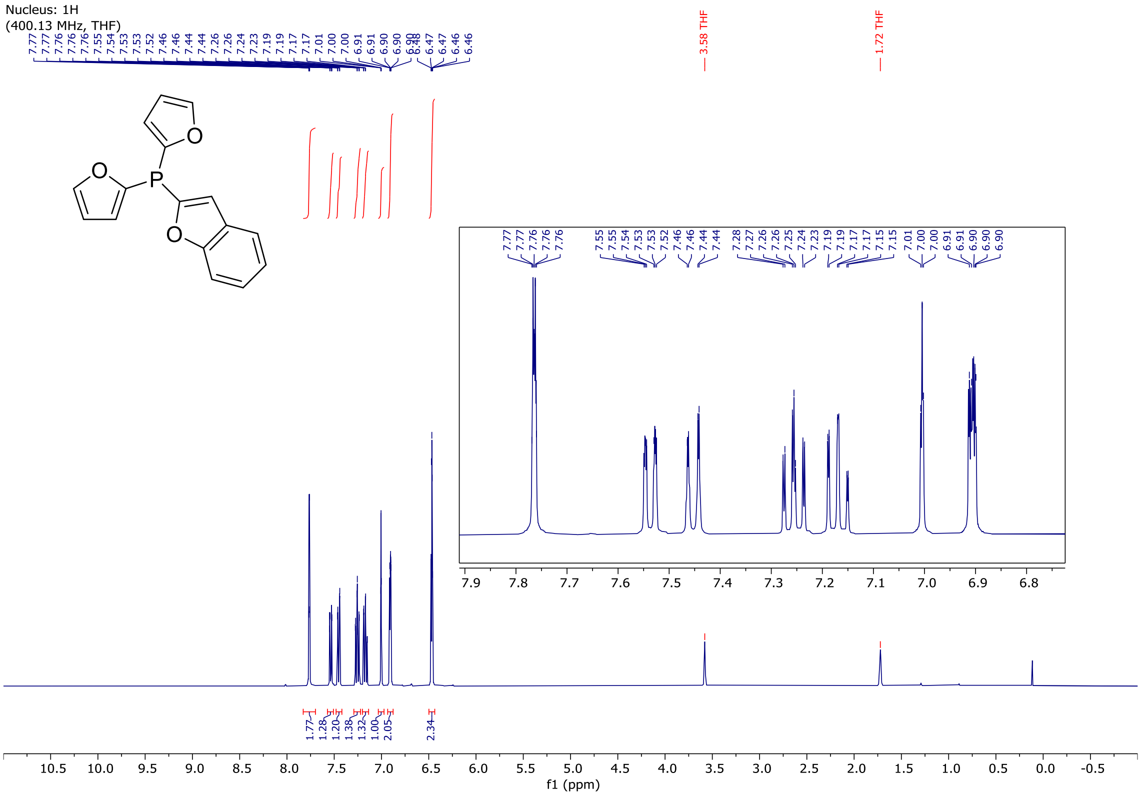

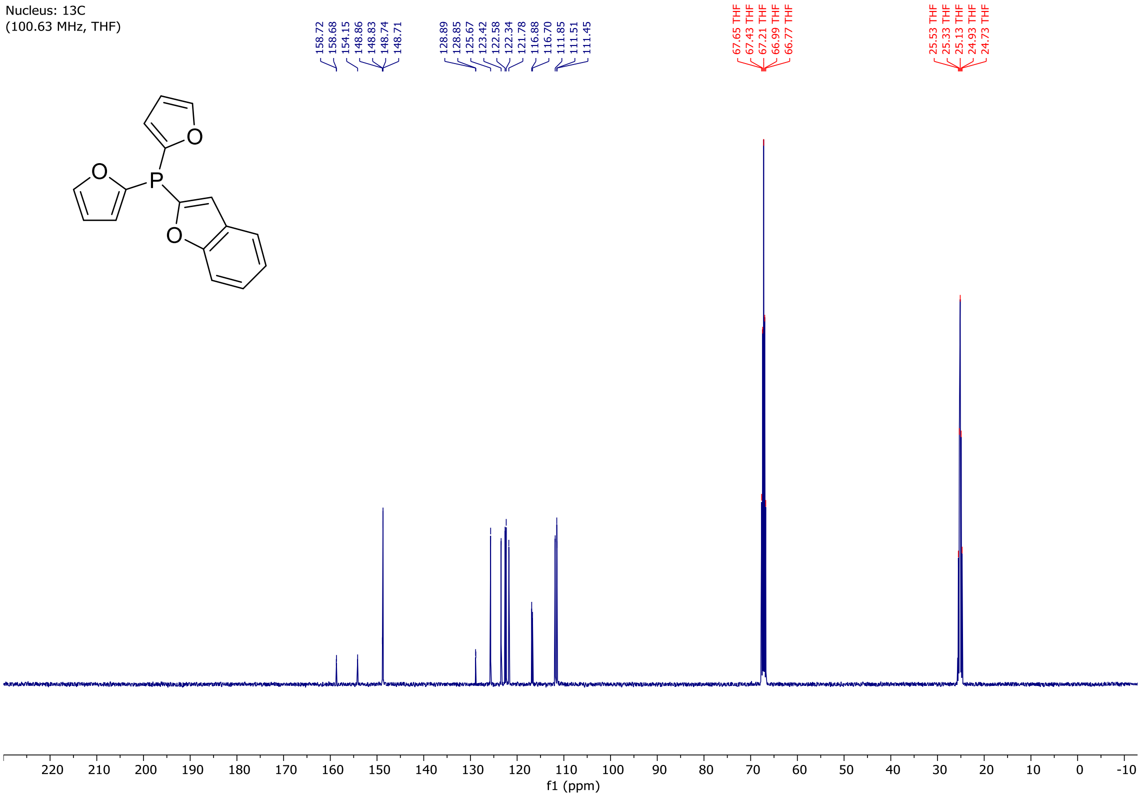

Nucleus: 31P
(121.52 MHz, THF)

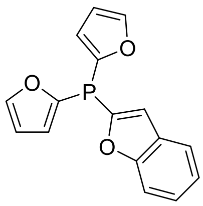

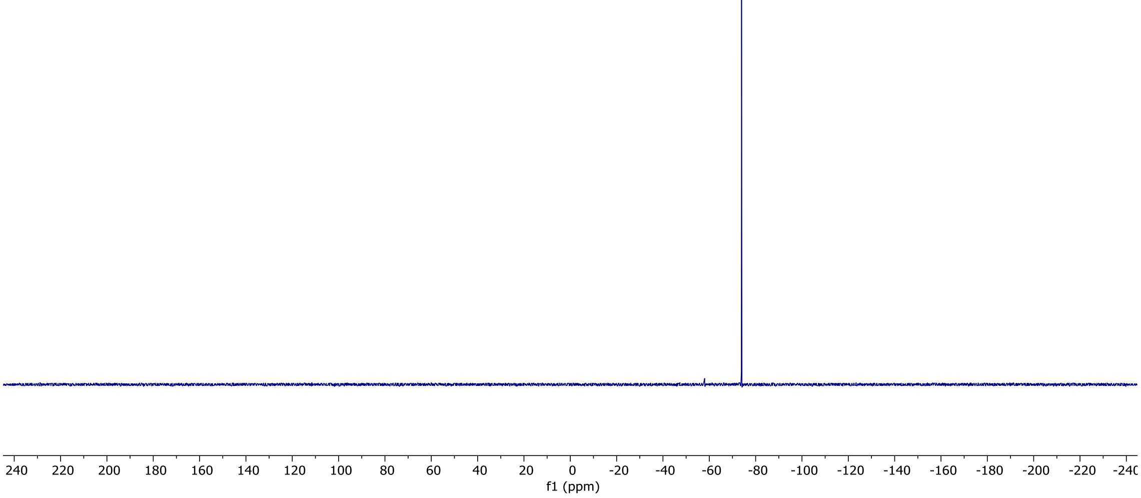

^{1}H NMR, ^{13}C NMR and ^{31}P NMR spectrum of **L14**

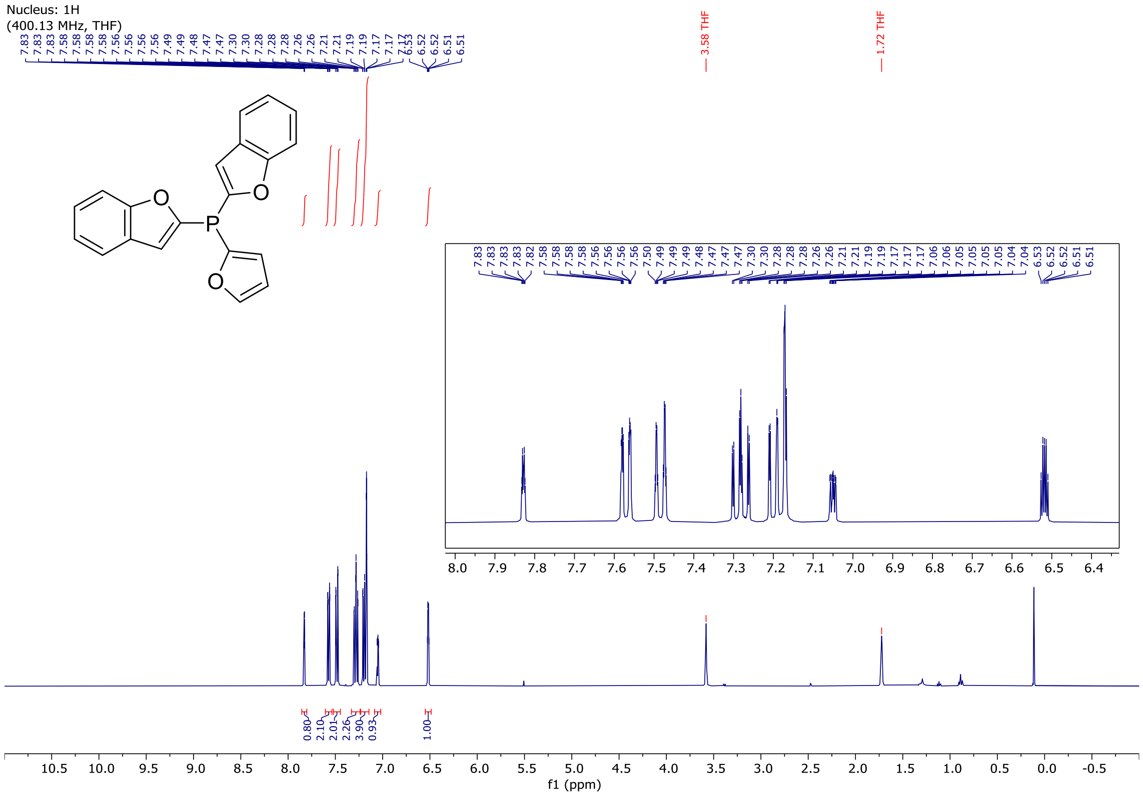

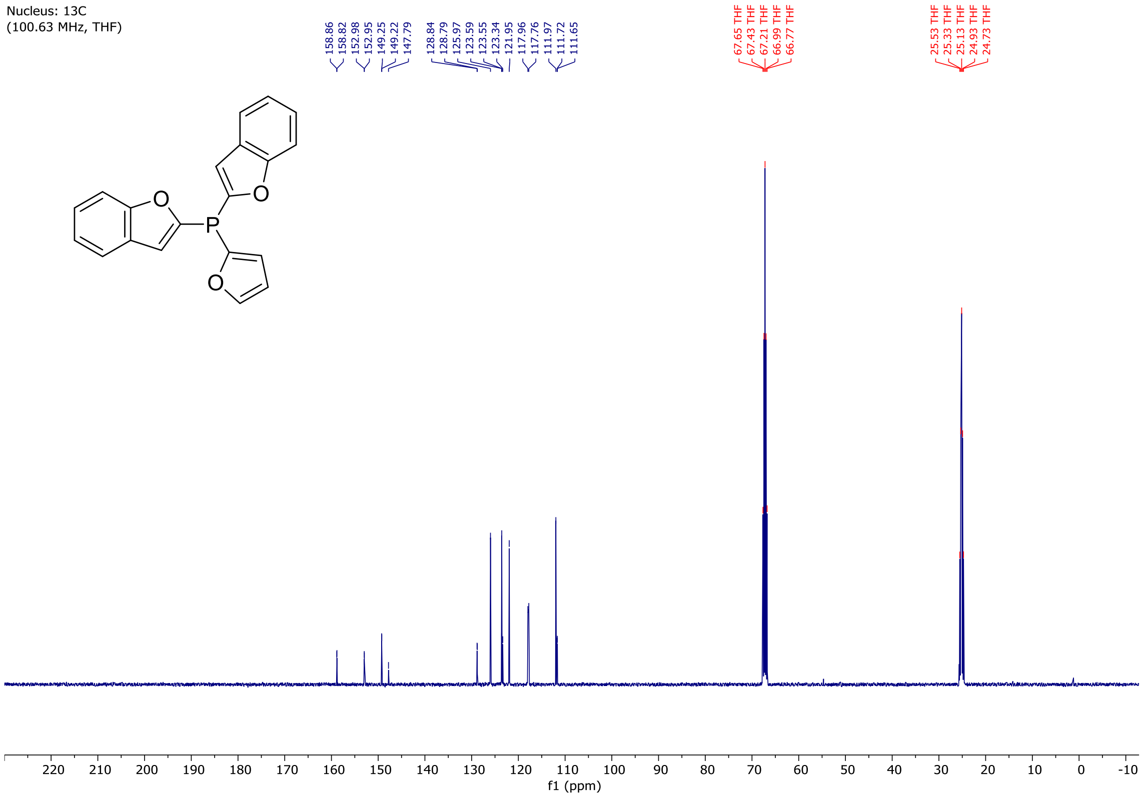

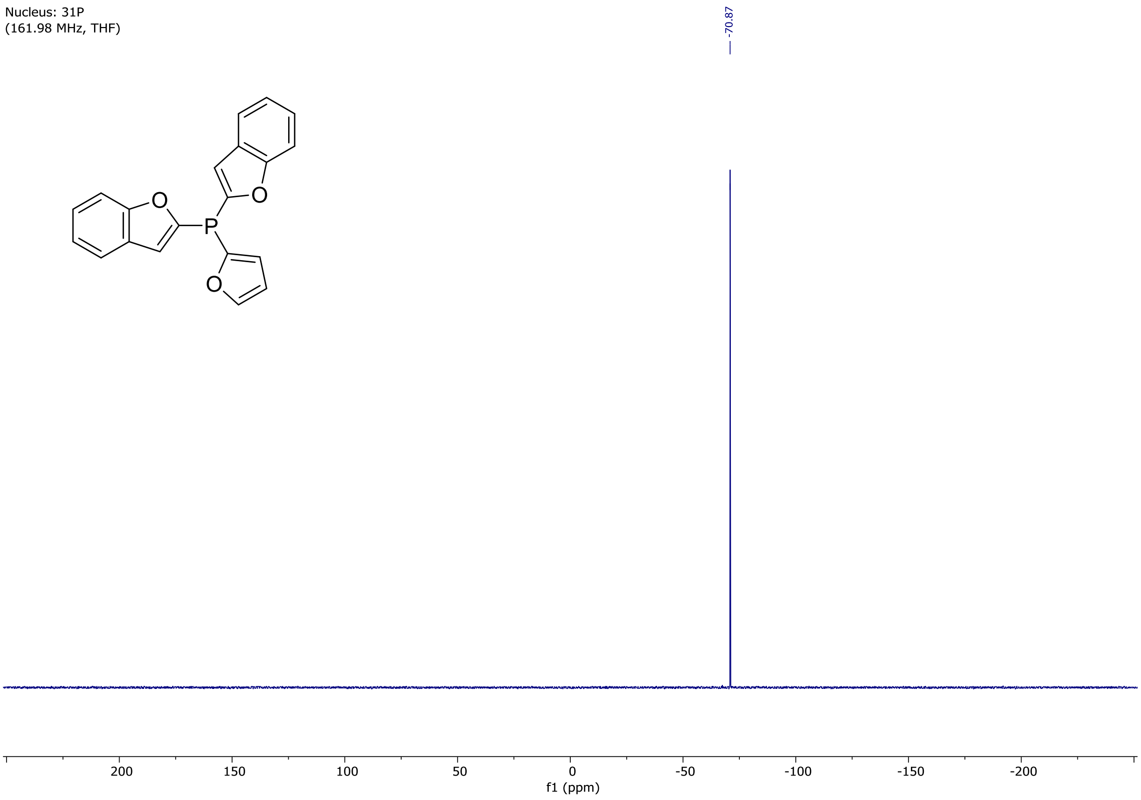
Nucleus: 31P
(161.98 MHz, THF)
-70.87
O
P
O
O
200
150
100
50
0
-50
-100
-150
-200
f1 (ppm)

^{1}H NMR, ^{13}C NMR and ^{31}P NMR spectrum of **L15**

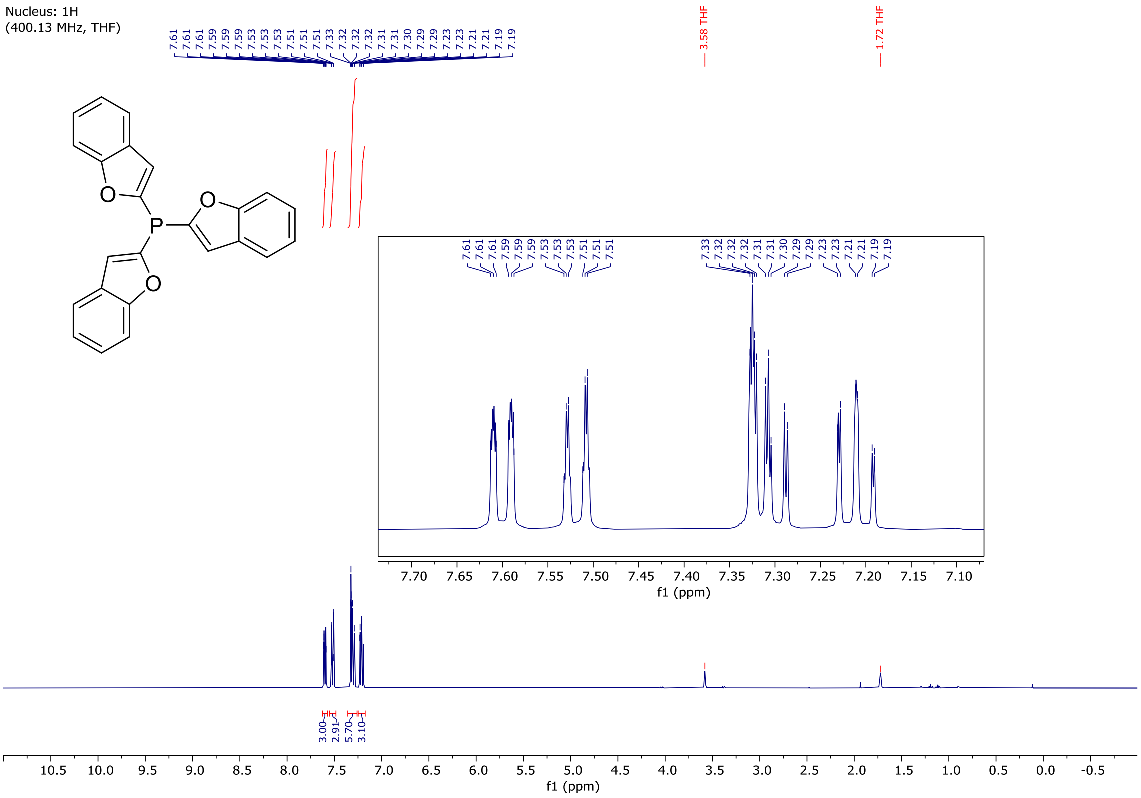

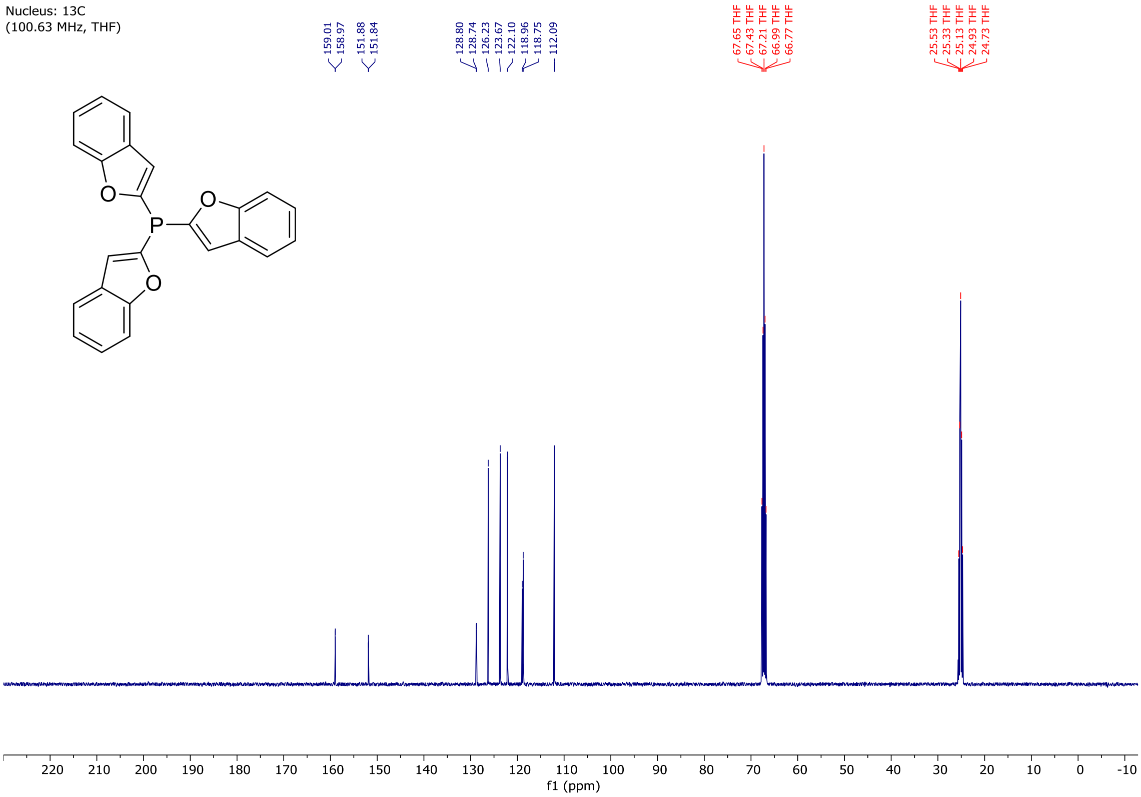

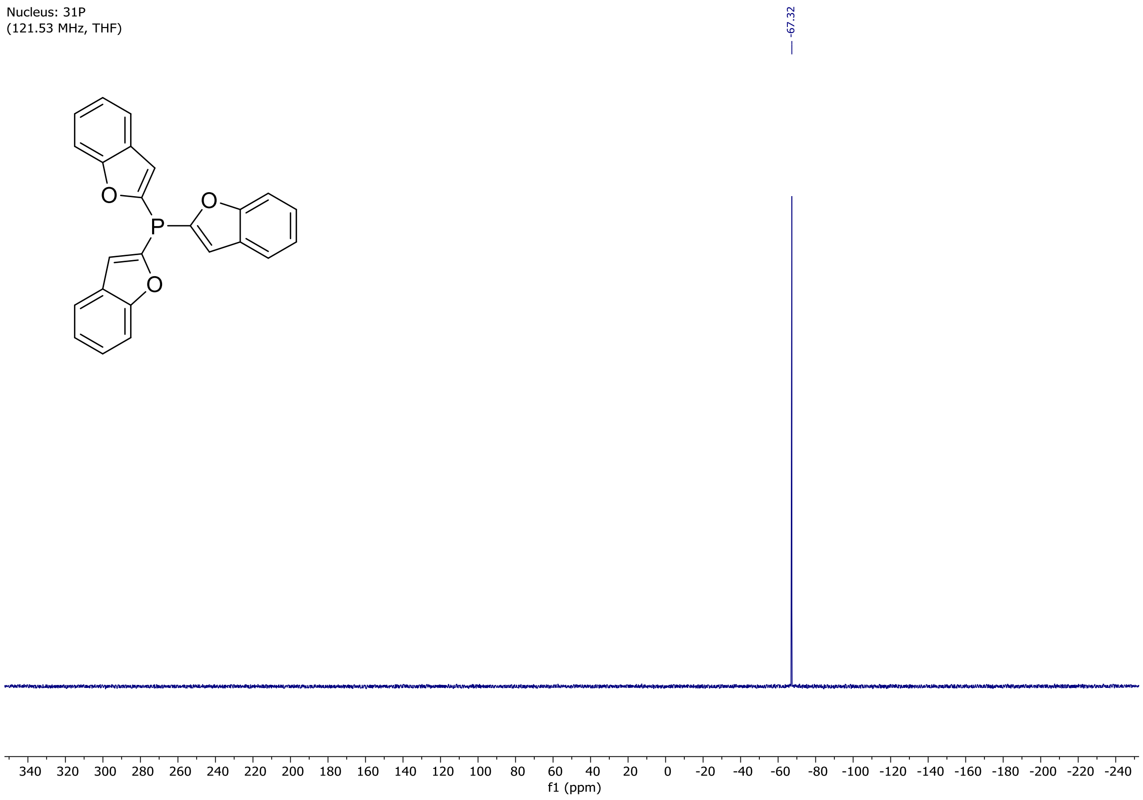
Nucleus: 31P
(121.53 MHz, THF)
-67.32
O
P
O
O
340 320 300 280 260 240 220 200 180 160 140 120 100 80 60 40 20 0 -20 -40 -60 -80 -100 -120 -140 -160 -180 -200 -220 -240
f1 (ppm)

^{1}H NMR, ^{13}C NMR and ^{31}P NMR spectrum of **L16**

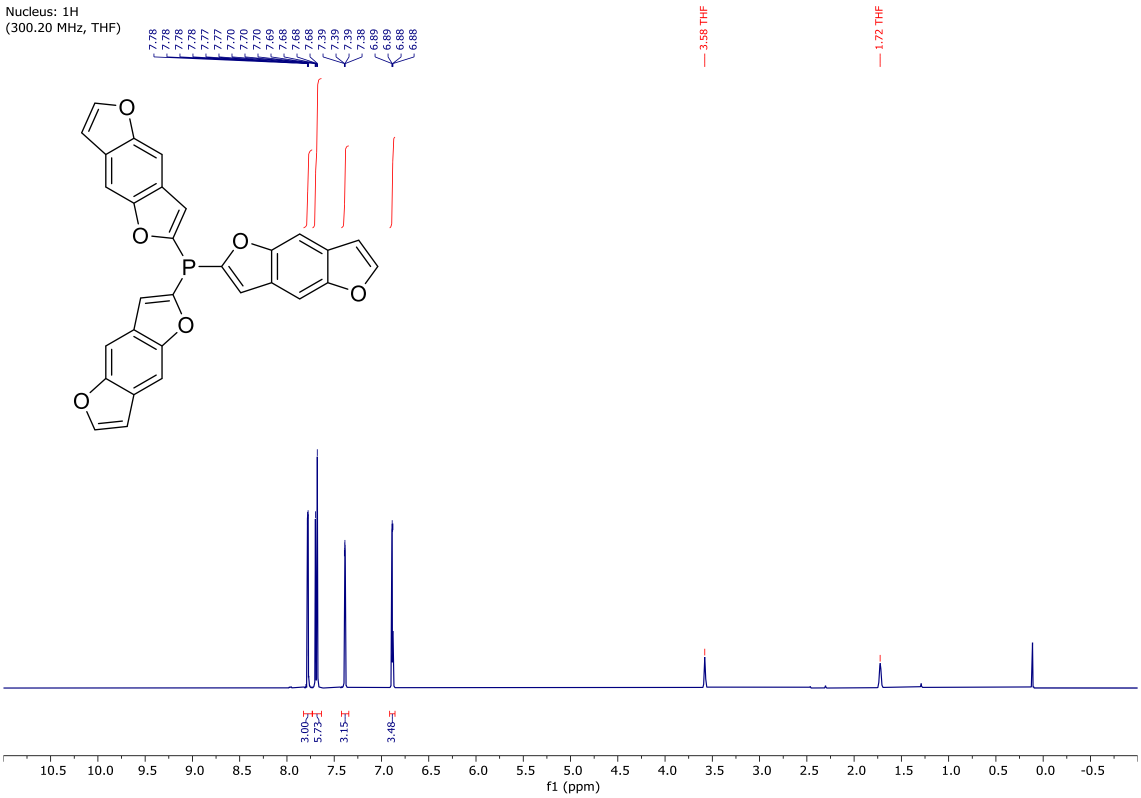

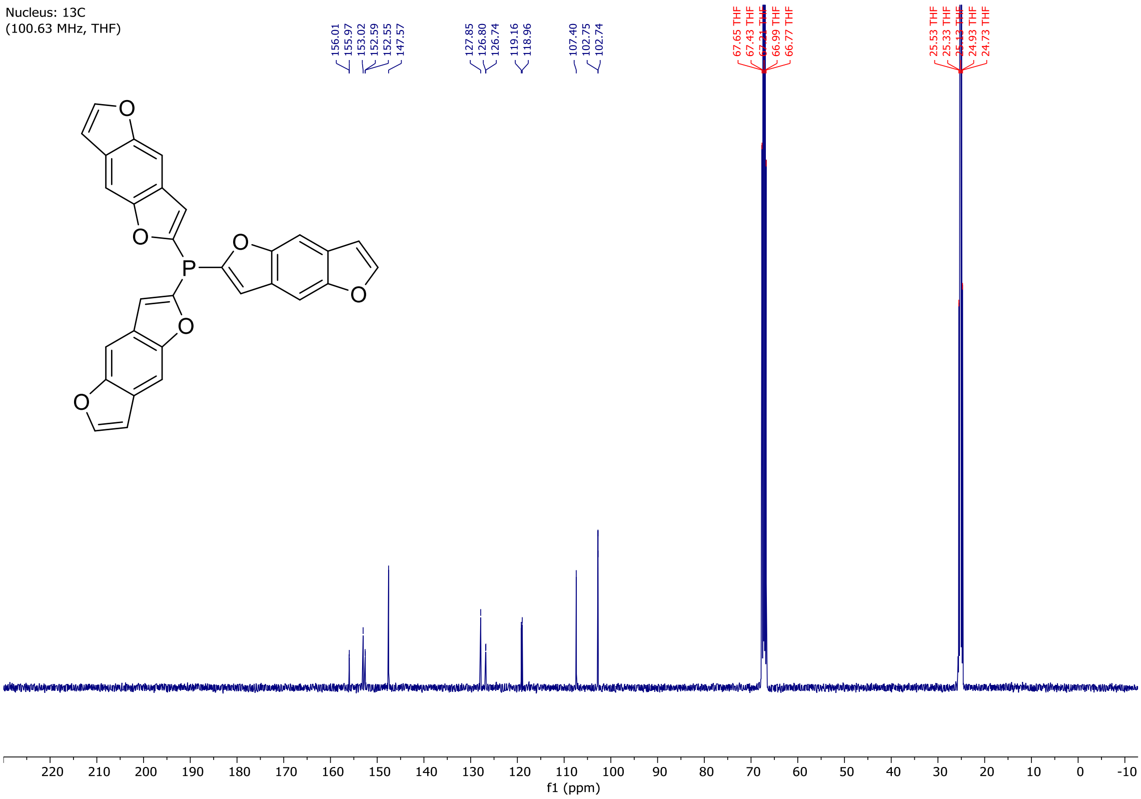

Nucleus: 31P
(121.53 MHz, THF)

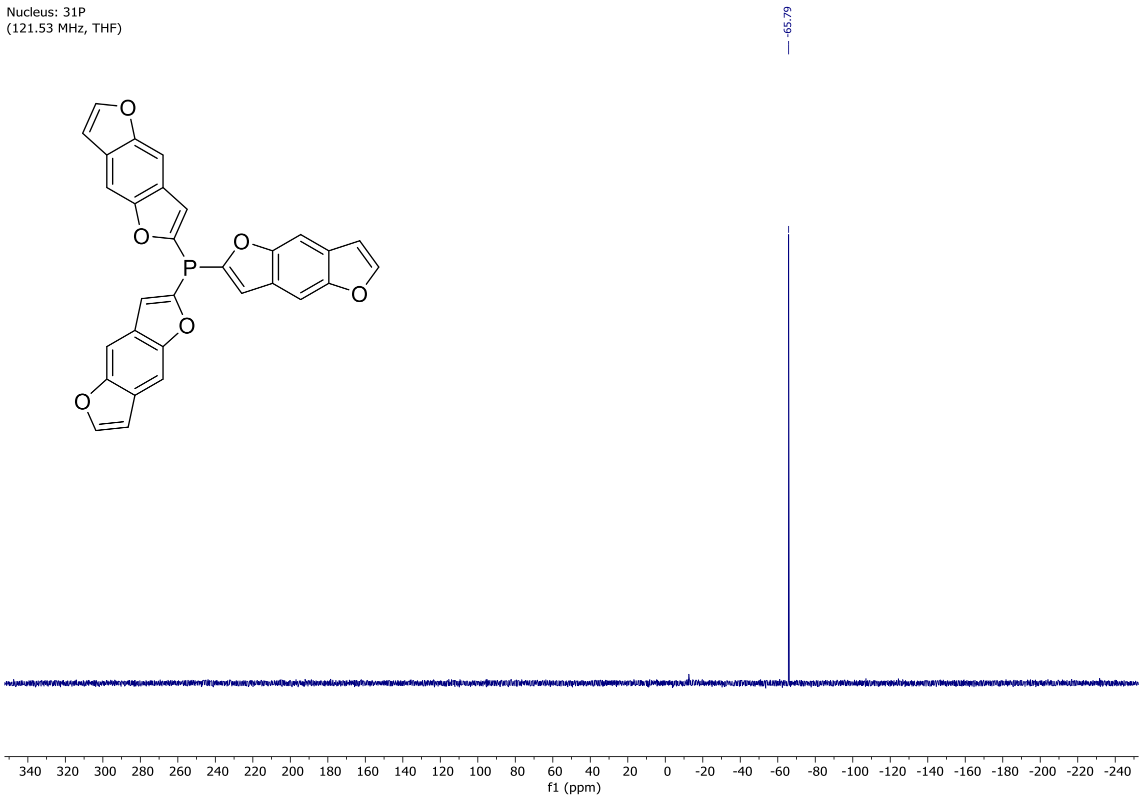

^{1}H NMR, ^{13}C NMR and ^{31}P NMR spectrum of **L17**

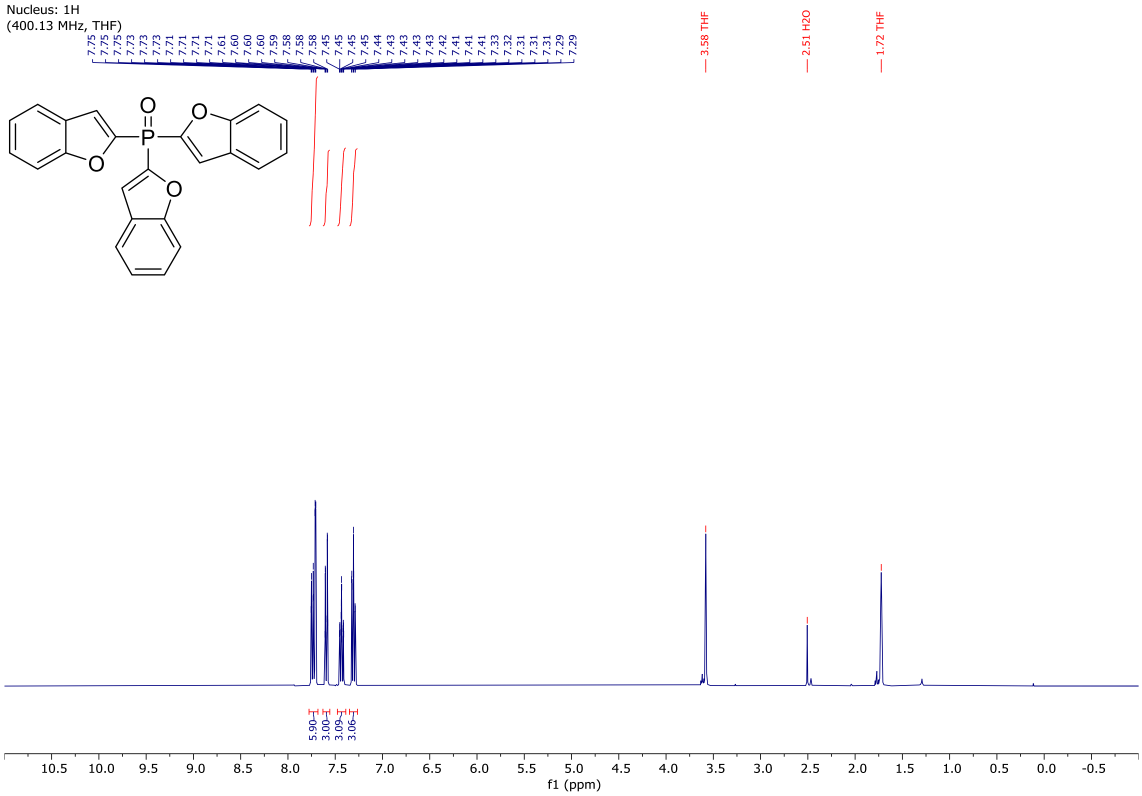

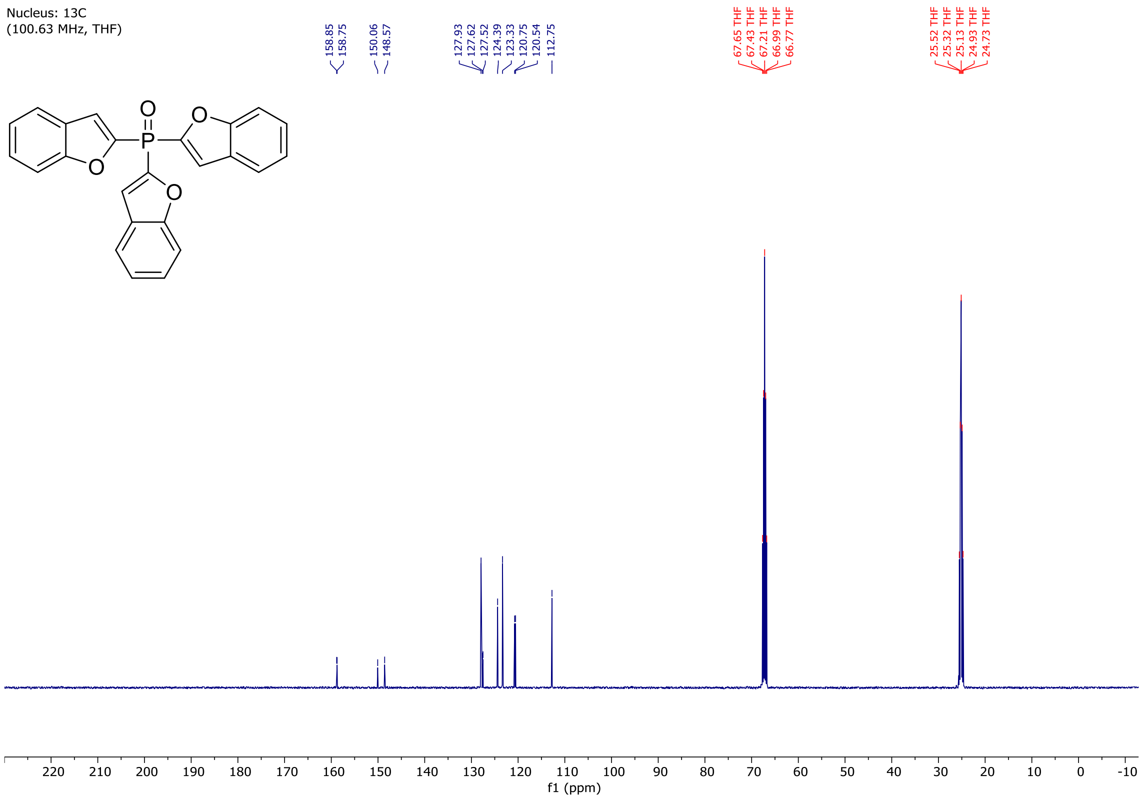

Nucleus: 31P
(161.99 MHz, THF)

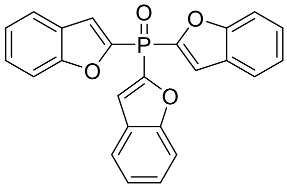

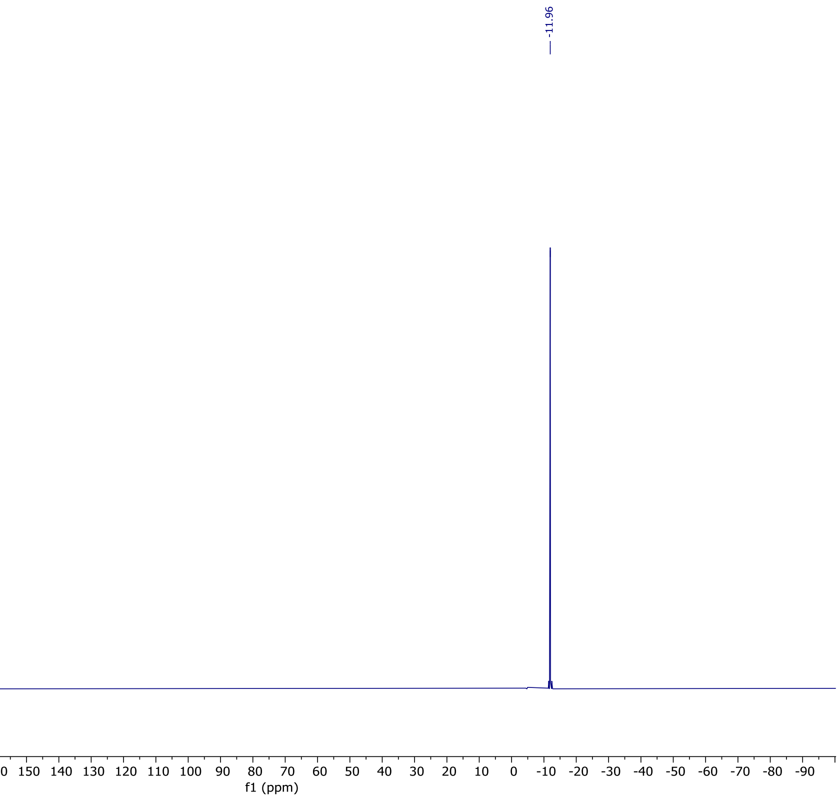

^{1}H NMR, ^{13}C NMR and ^{31}P NMR spectrum of **L18**

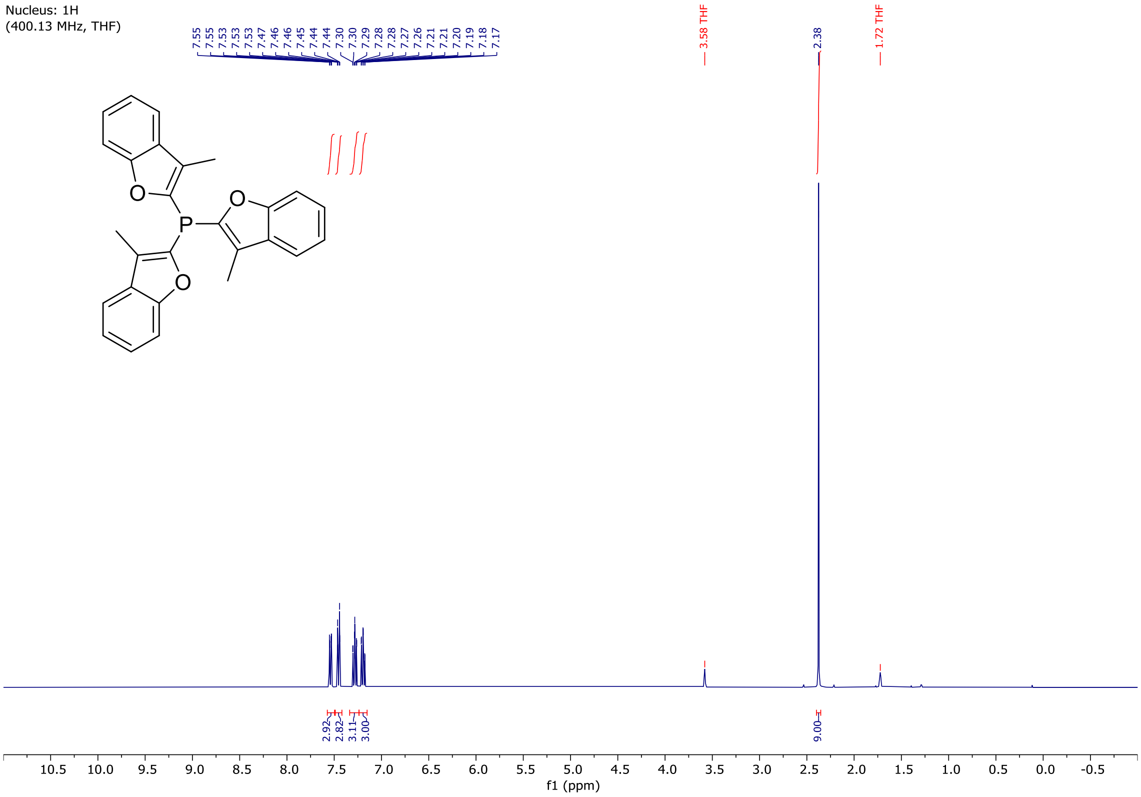

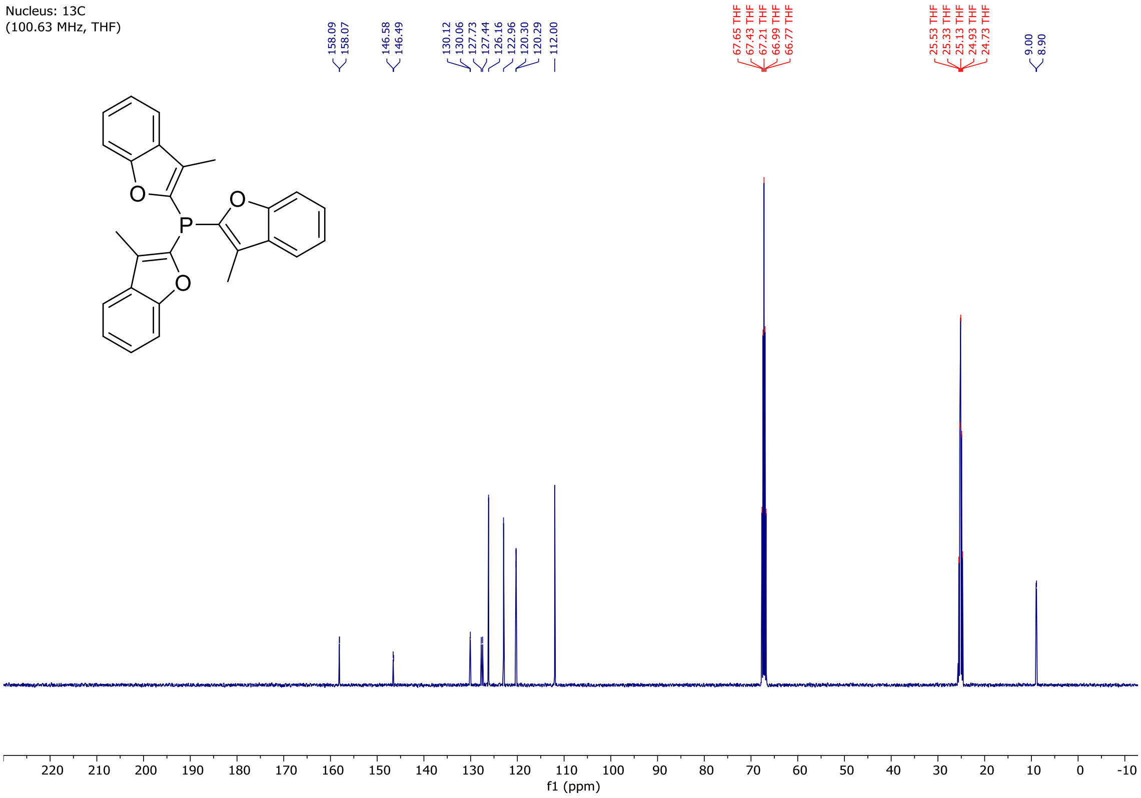

Nucleus: 31P
(121.53 MHz, THF)

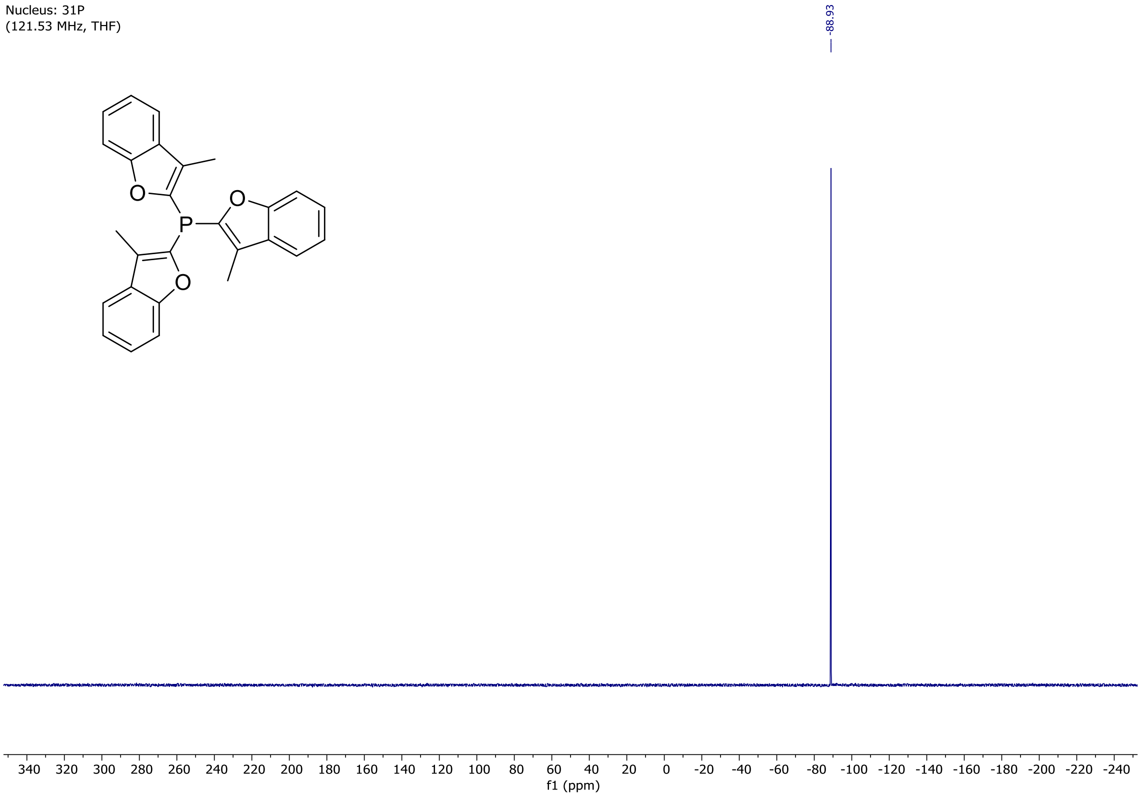

^{1}H NMR, ^{13}C NMR and ^{31}P NMR spectrum of di(benzofuran-2-yl)chlorophosphane

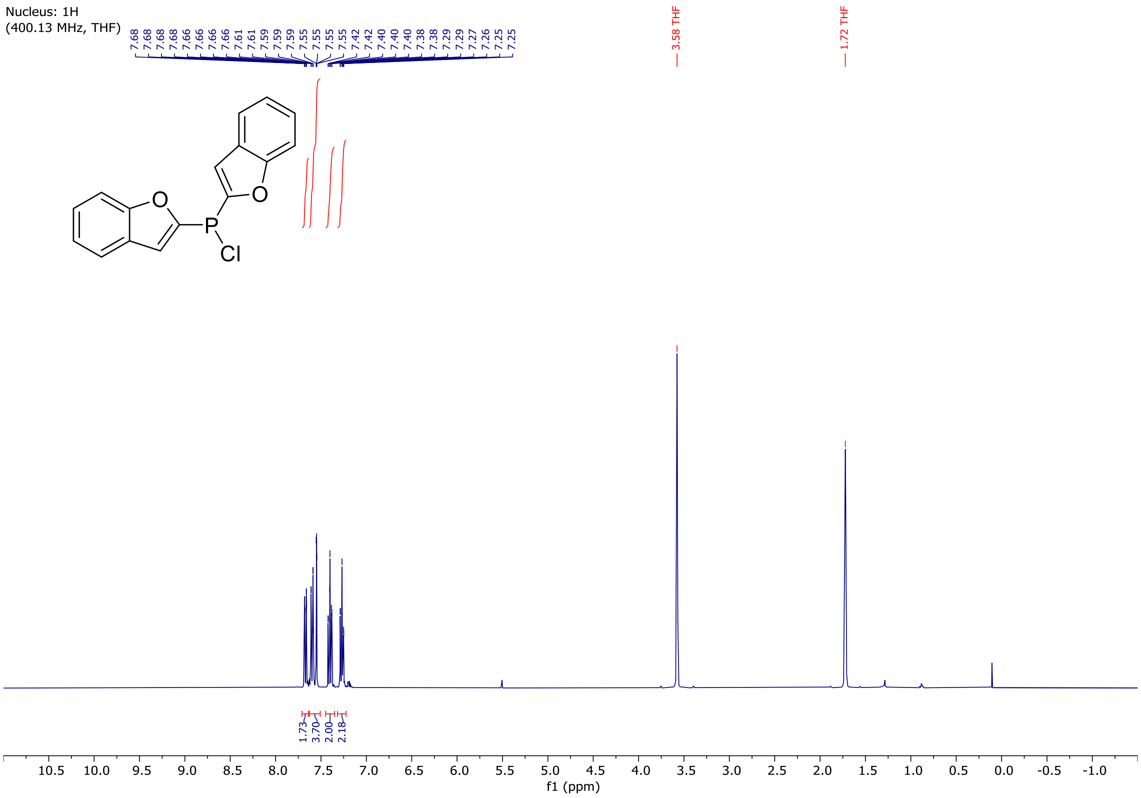

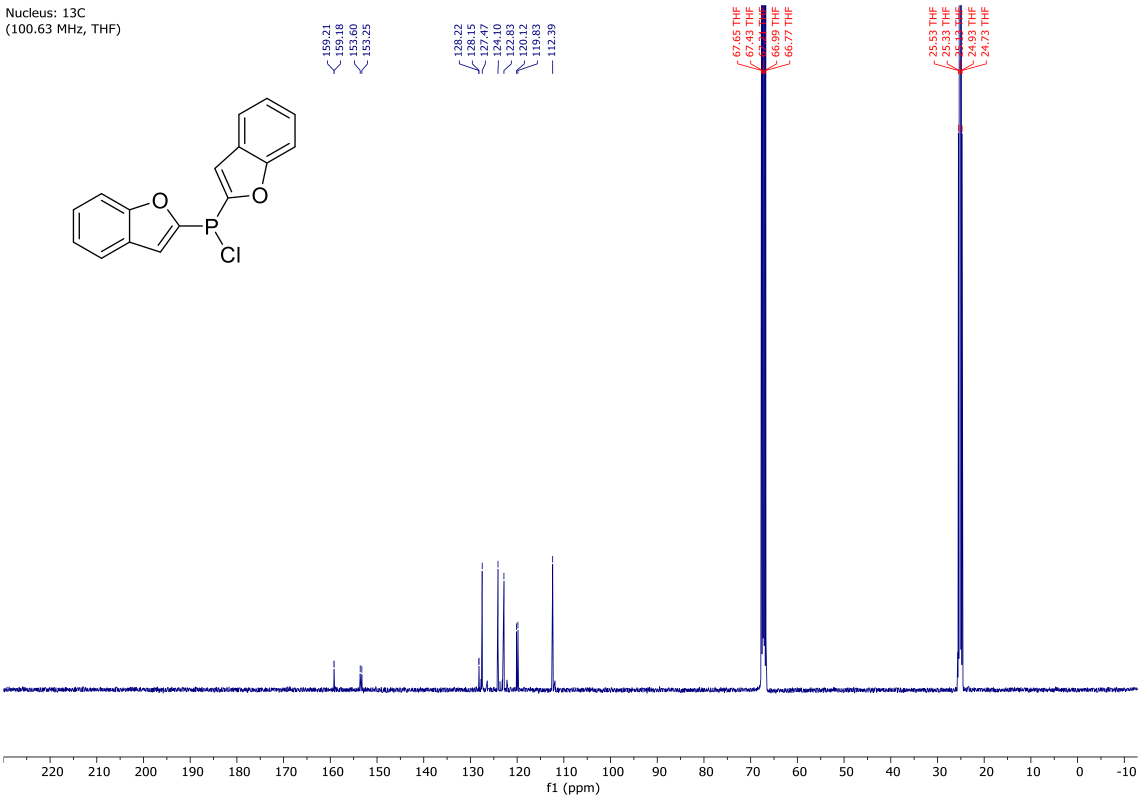

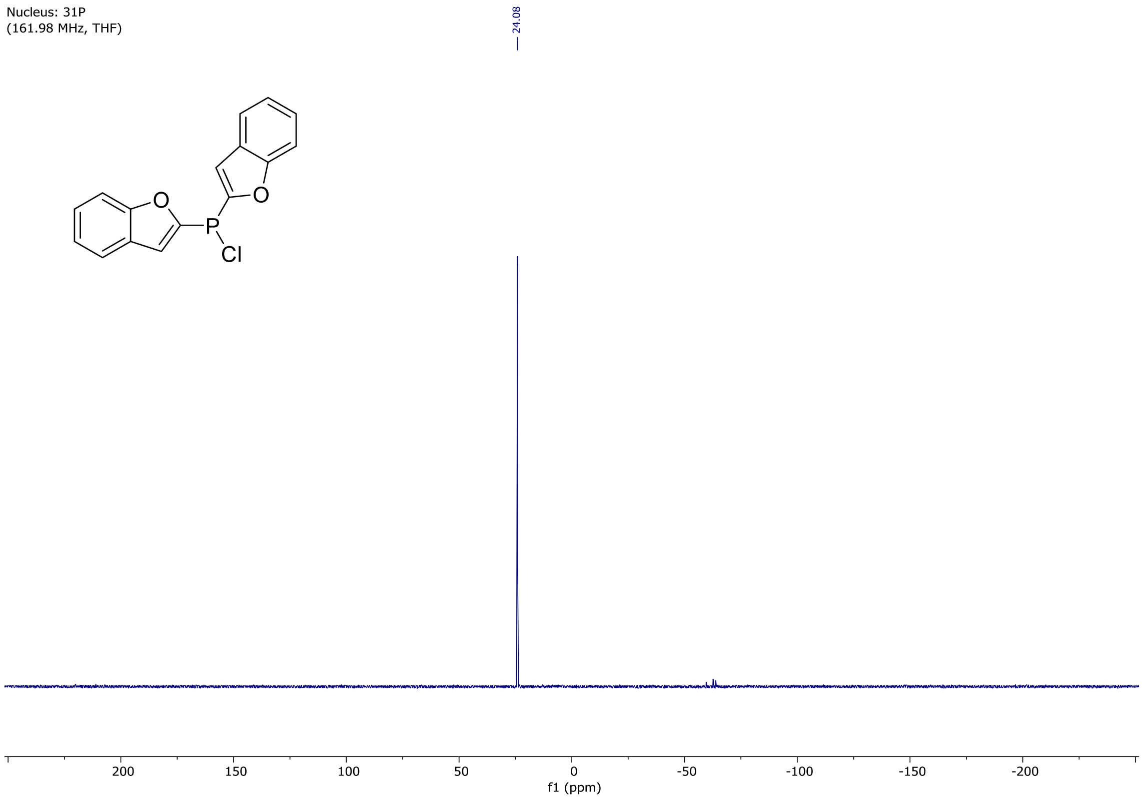
Nucleus: 31P
(161.98 MHz, THF)
24.08
O
O
P
Cl
200
150
100
50
0
-50
-100
-150
-200
f1 (ppm)

^{1}H NMR, ^{13}C NMR and ^{31}P NMR spectrum of **L19**

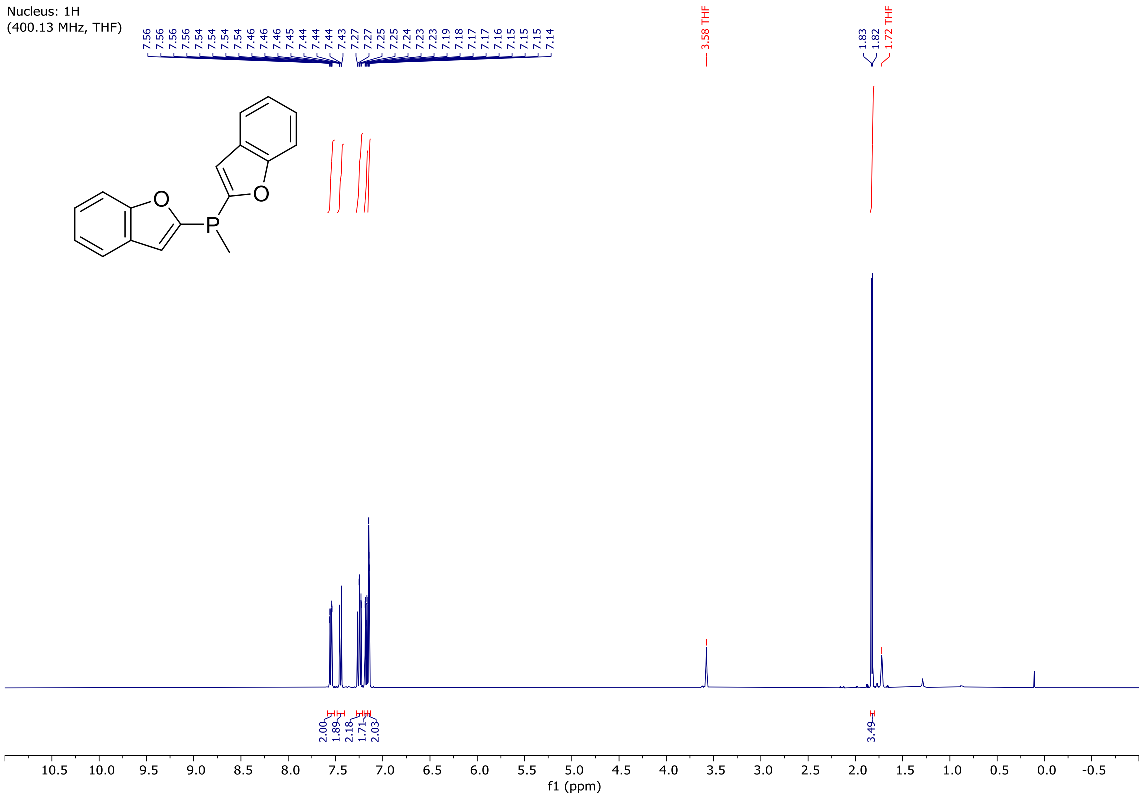

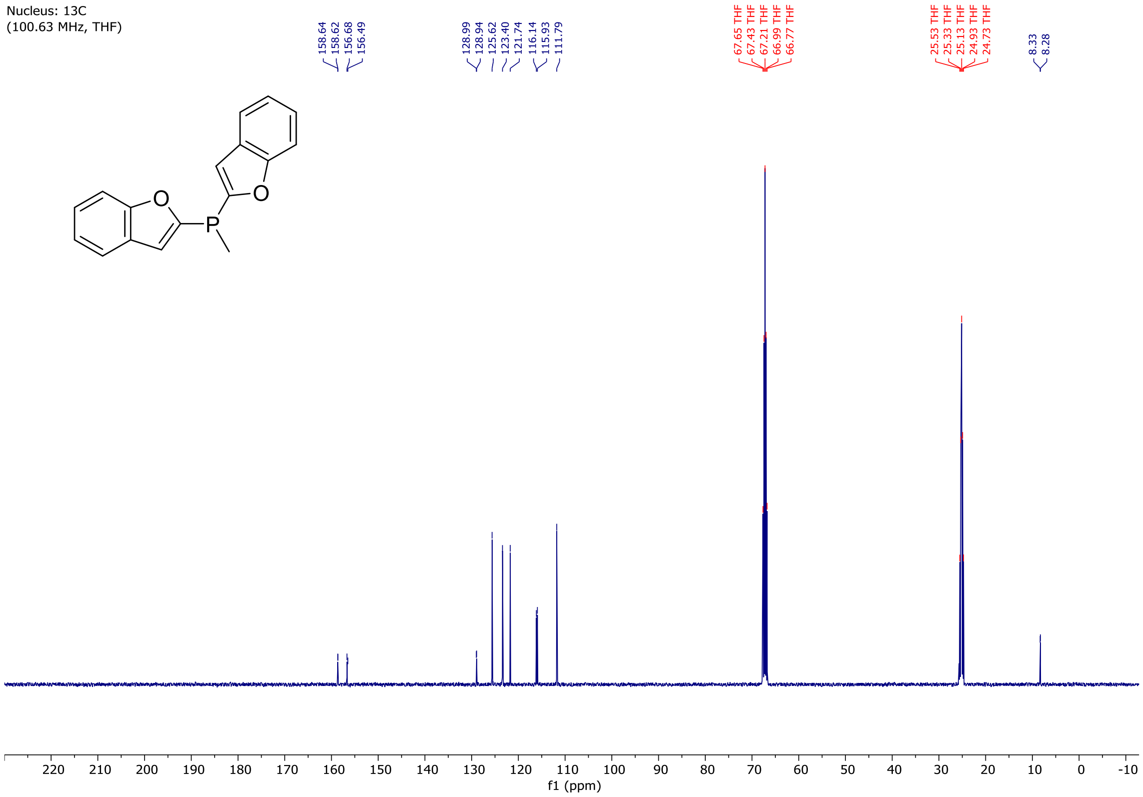

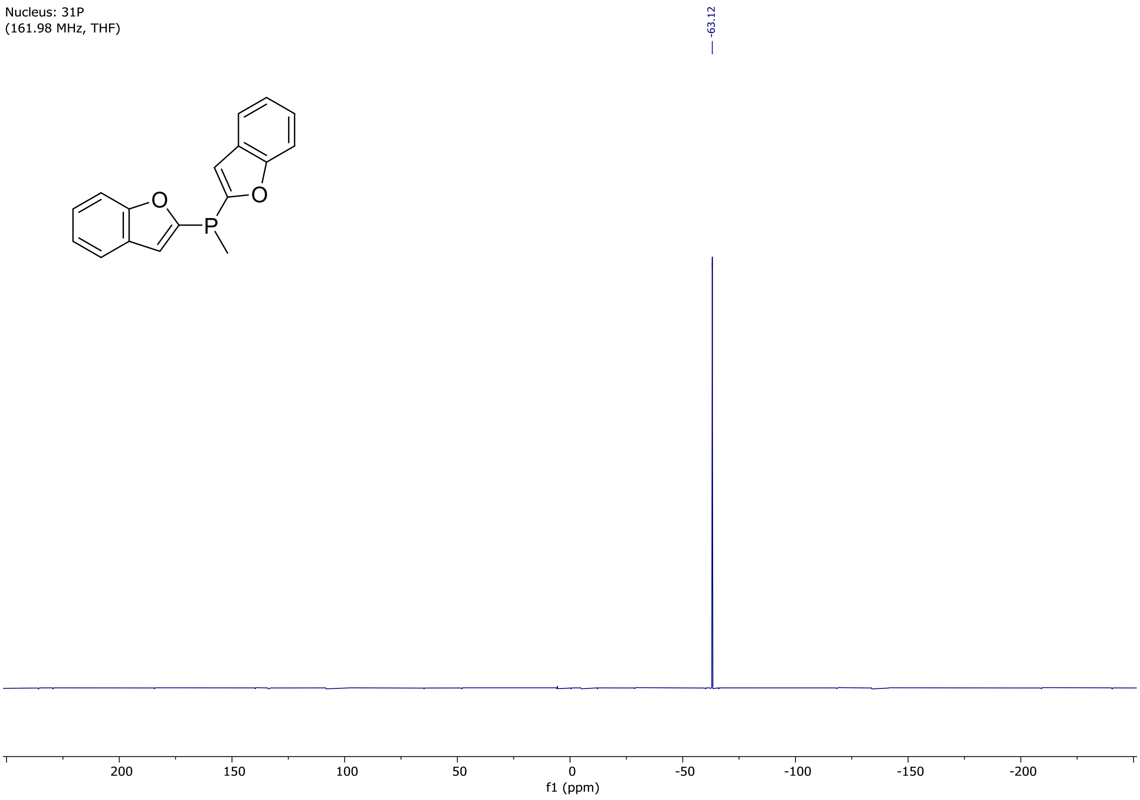
Nucleus: 31P
(161.98 MHz, THF)
-63.12
O
P
O
200
150
100
50
0
-50
-100
-150
-200
f1 (ppm)

^{1}H NMR, ^{13}C NMR and ^{31}P NMR spectrum of **L20**

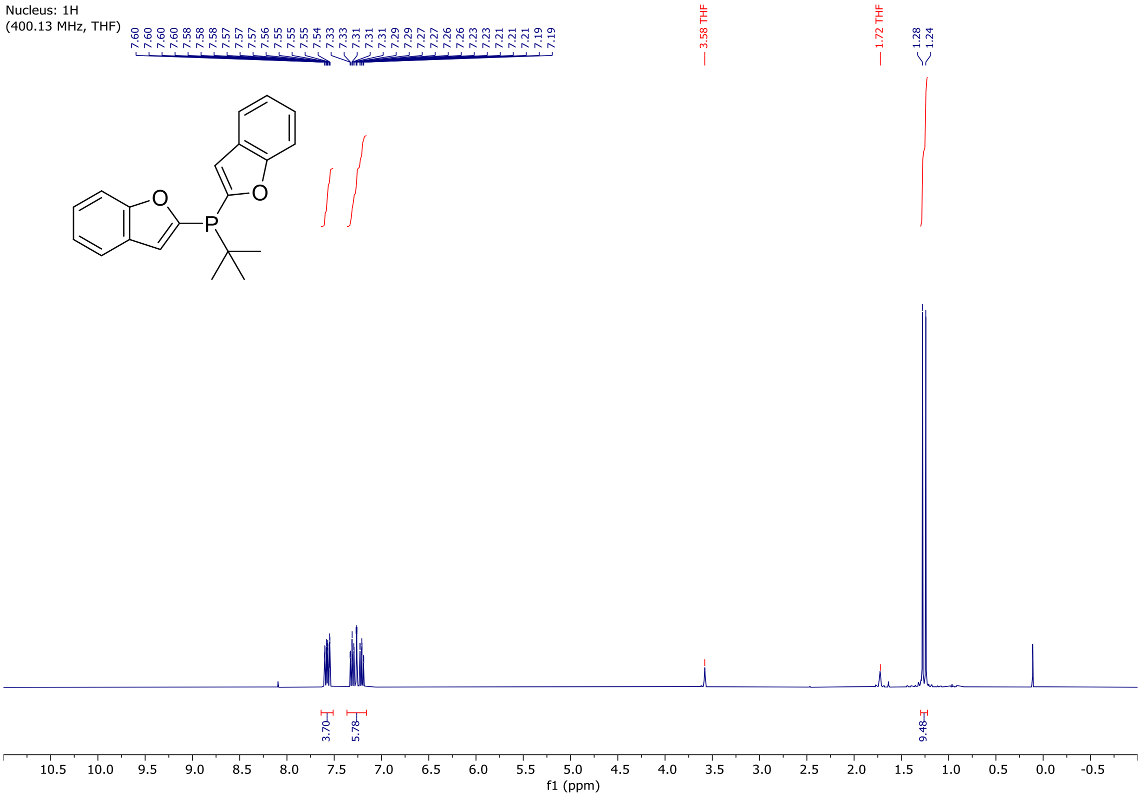

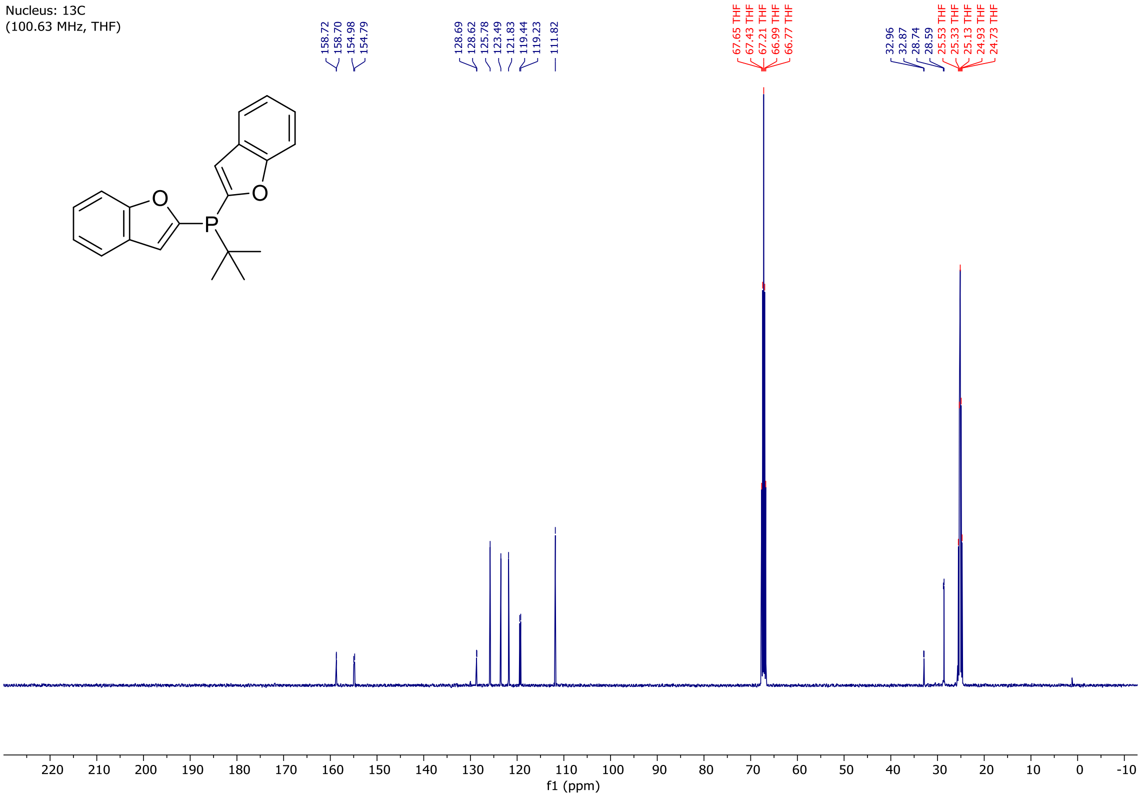

Nucleus: 31P
(121.52 MHz, THF)

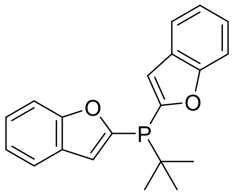

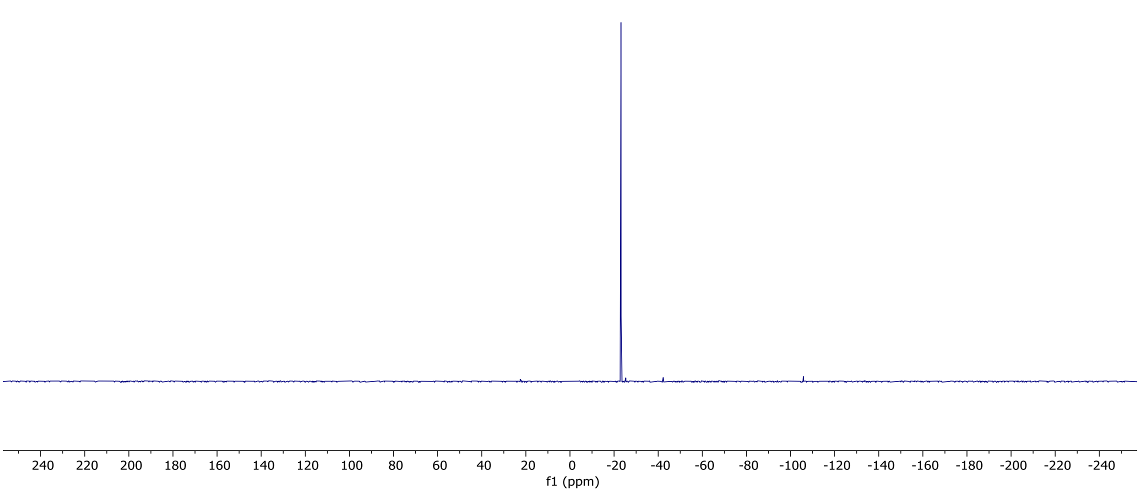

^{1}H NMR, ^{13}C NMR and ^{31}P NMR spectrum of **L21**

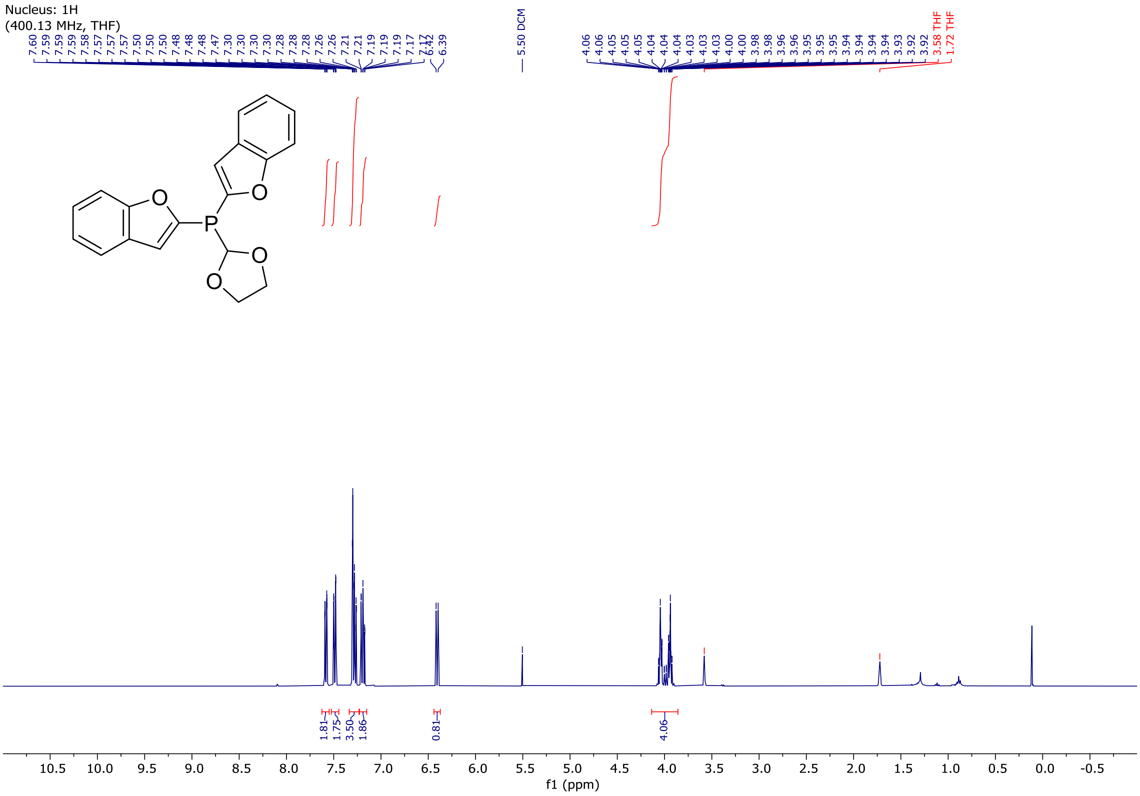

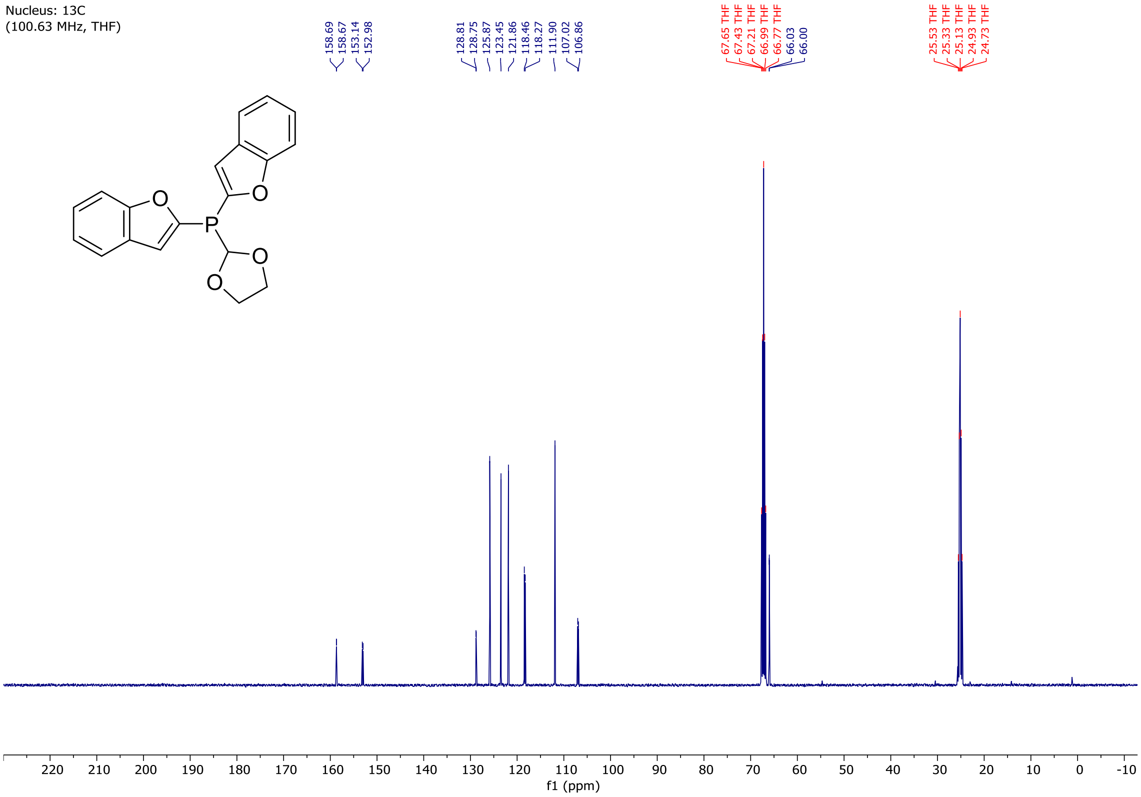

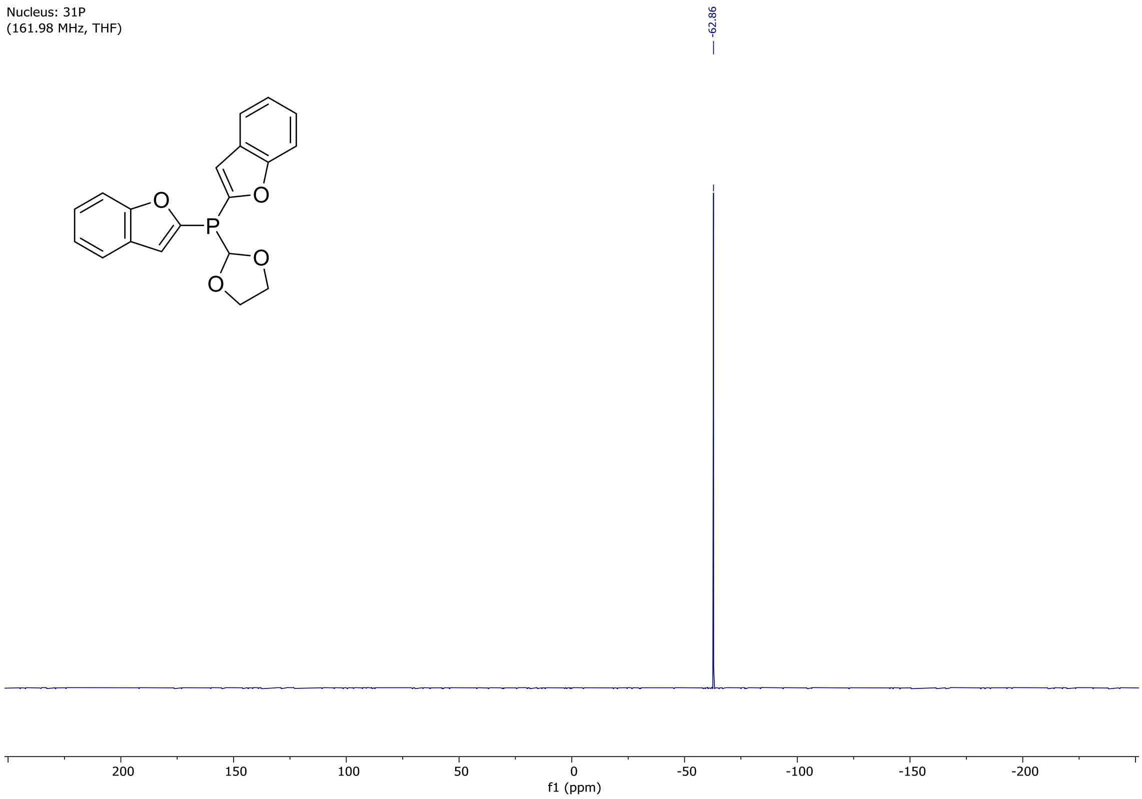
Nucleus: 31P
(161.98 MHz, THF)
-62.86
O
P
O
O
O
200
150
100
50
0
-50
-100
-150
-200
f1 (ppm)

^{1}H NMR, ^{13}C NMR and ^{31}P NMR spectrum of **L22**

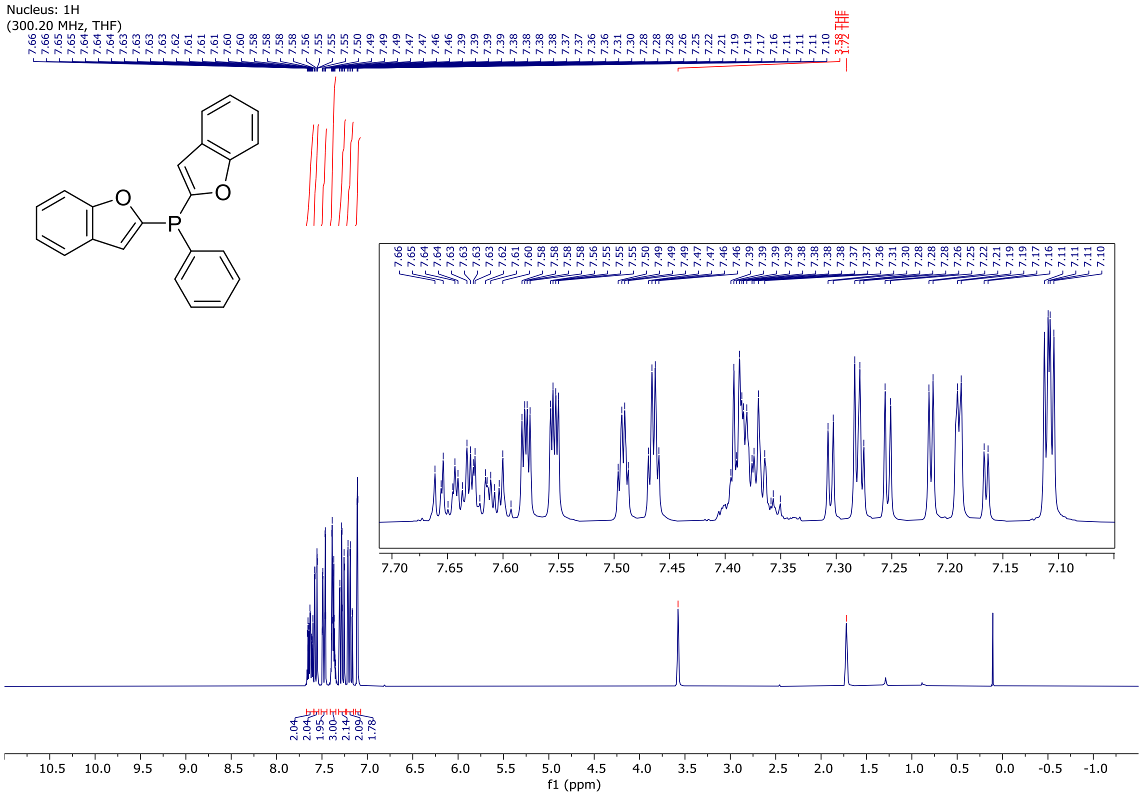

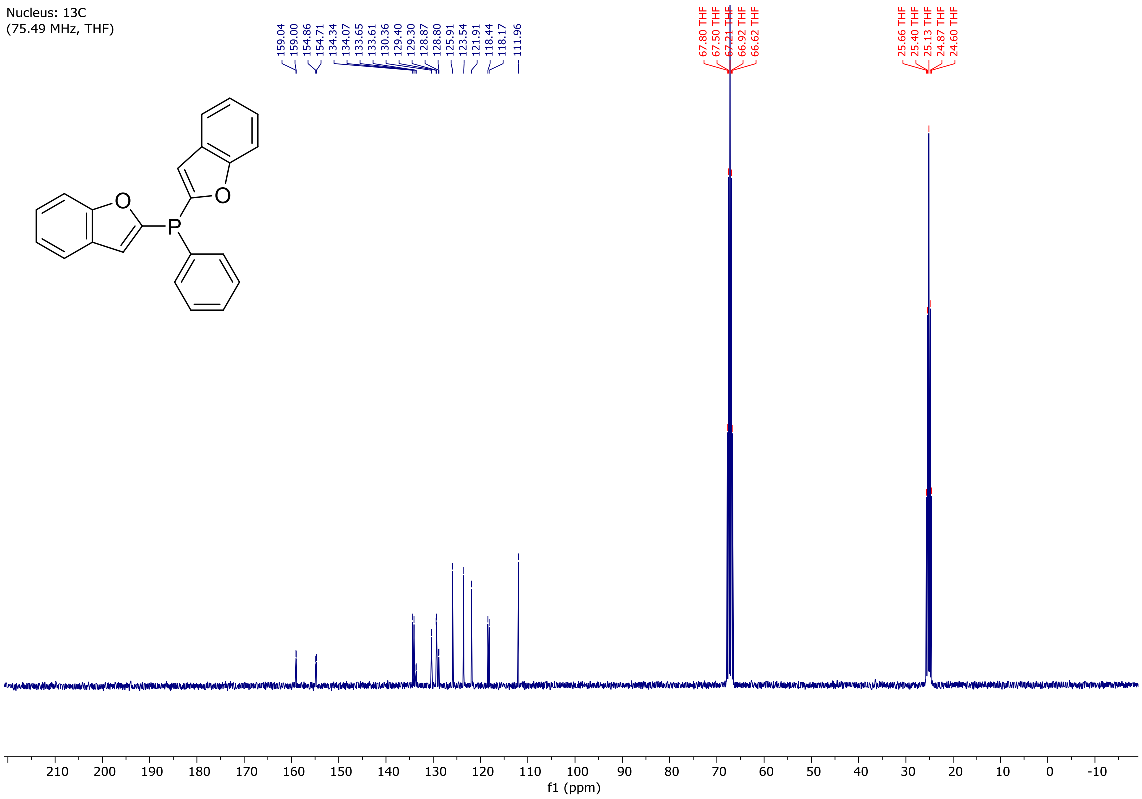

Nucleus: 31P
(161.98 MHz, THF)

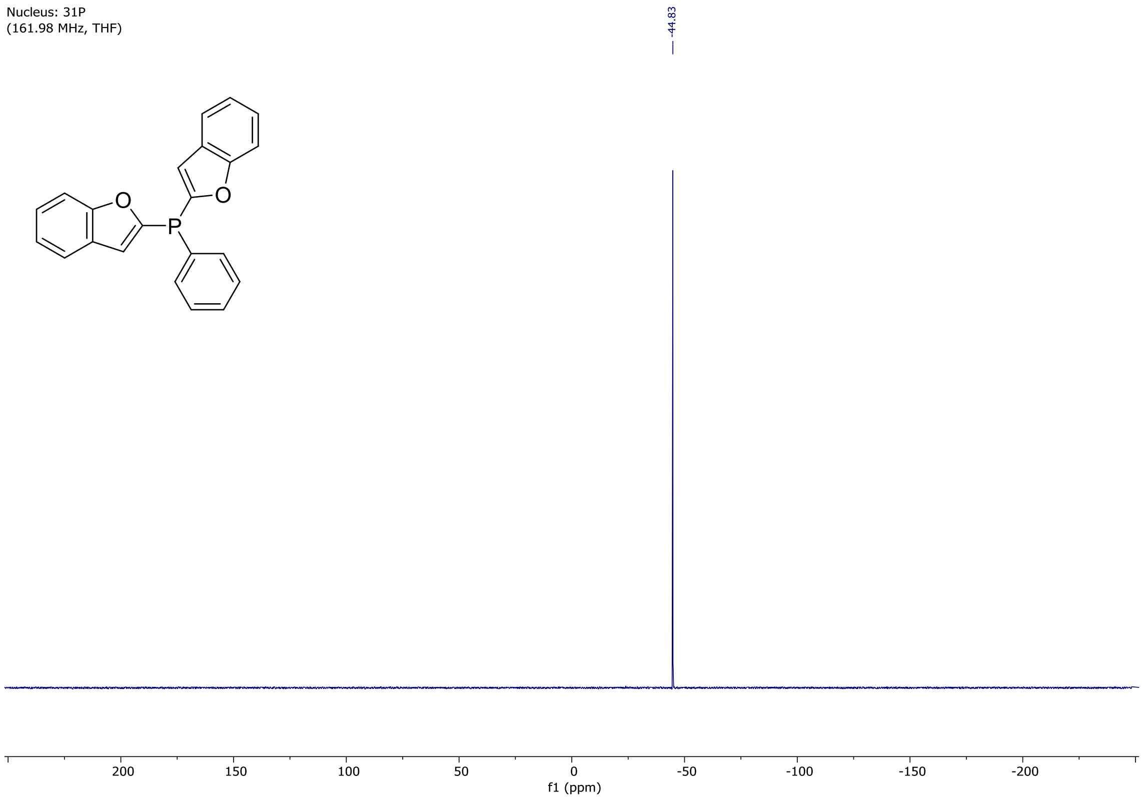

^{1}H NMR, ^{13}C NMR and ^{31}P NMR spectrum of **L23**

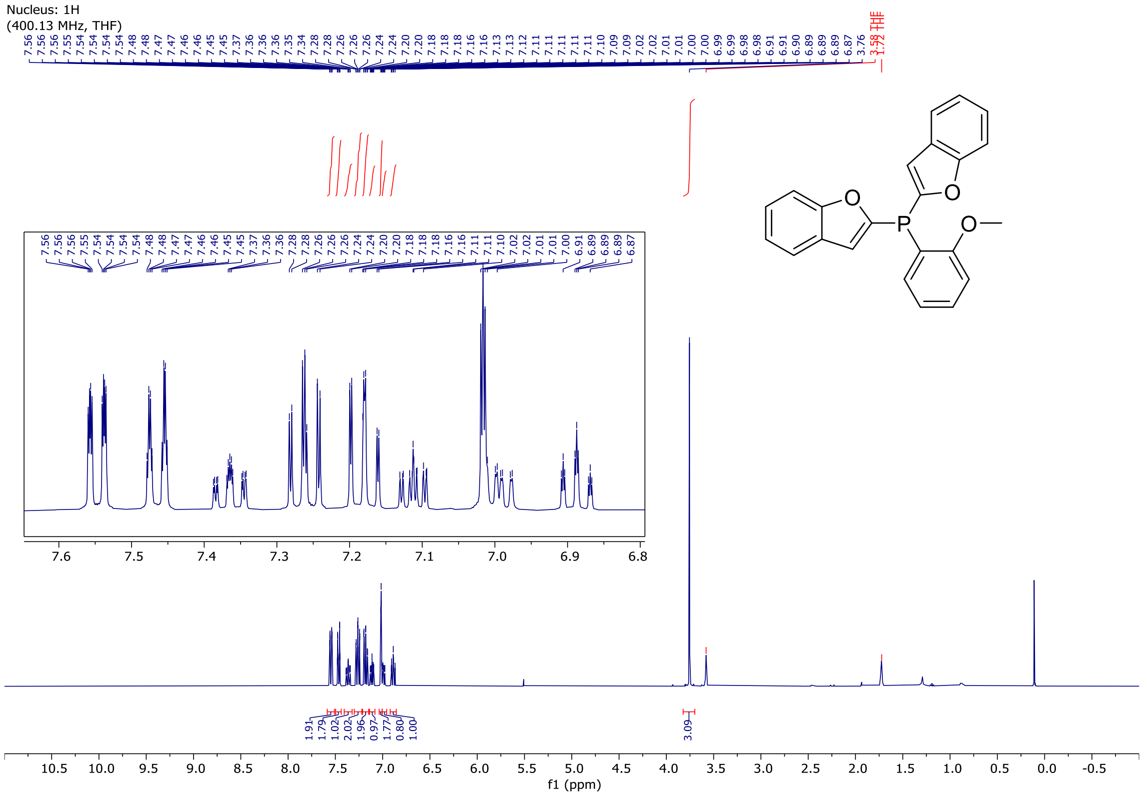

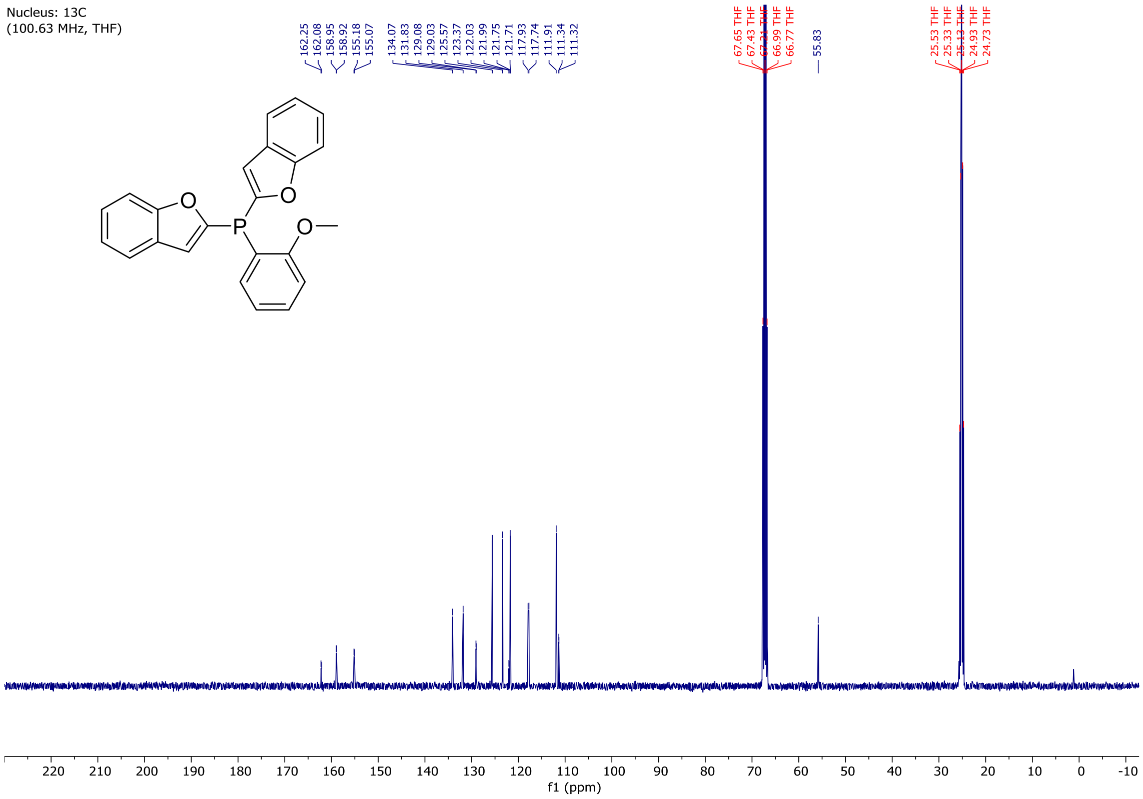

Nucleus: 31P
(161.98 MHz, THF)

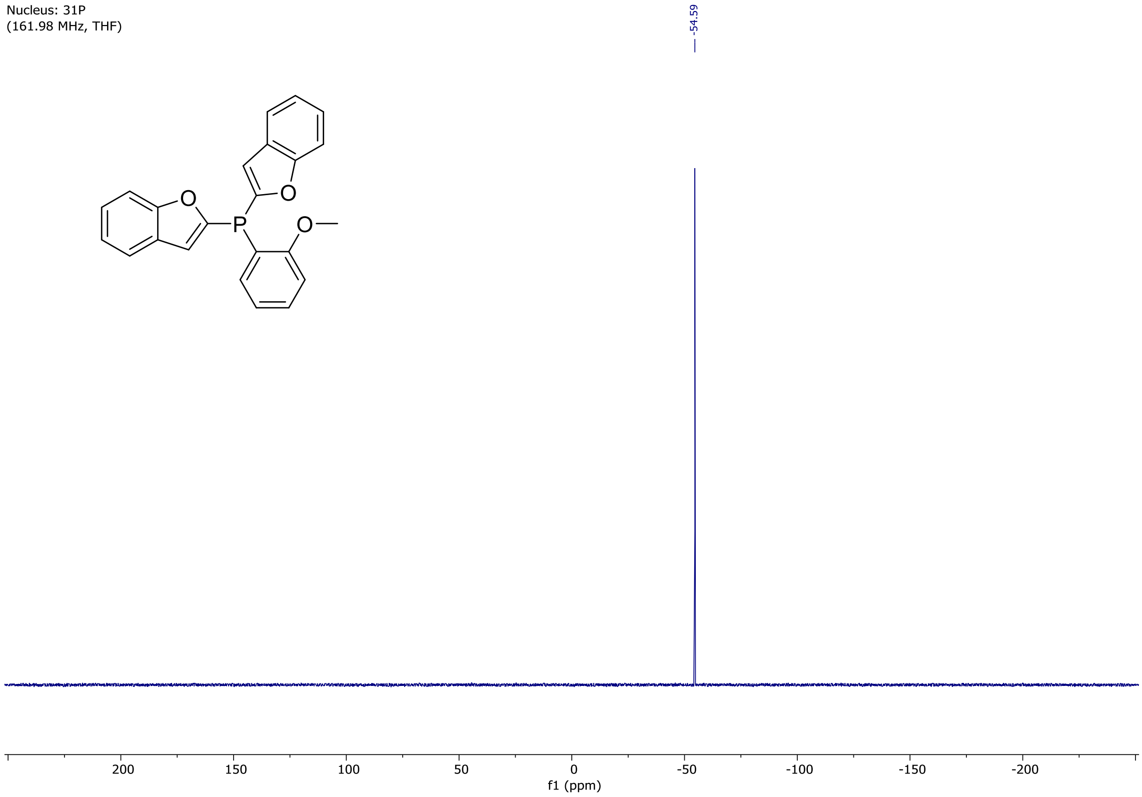

^{1}H NMR, ^{13}C NMR and ^{31}P NMR spectrum of **L24**

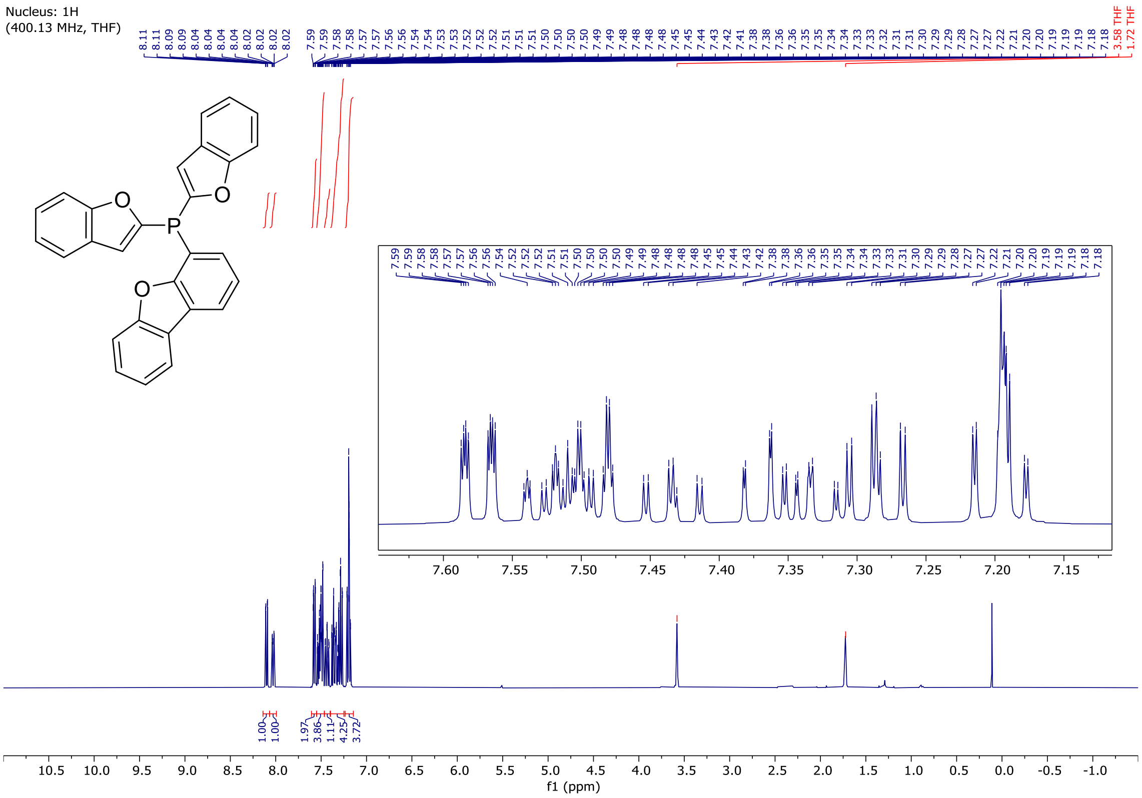

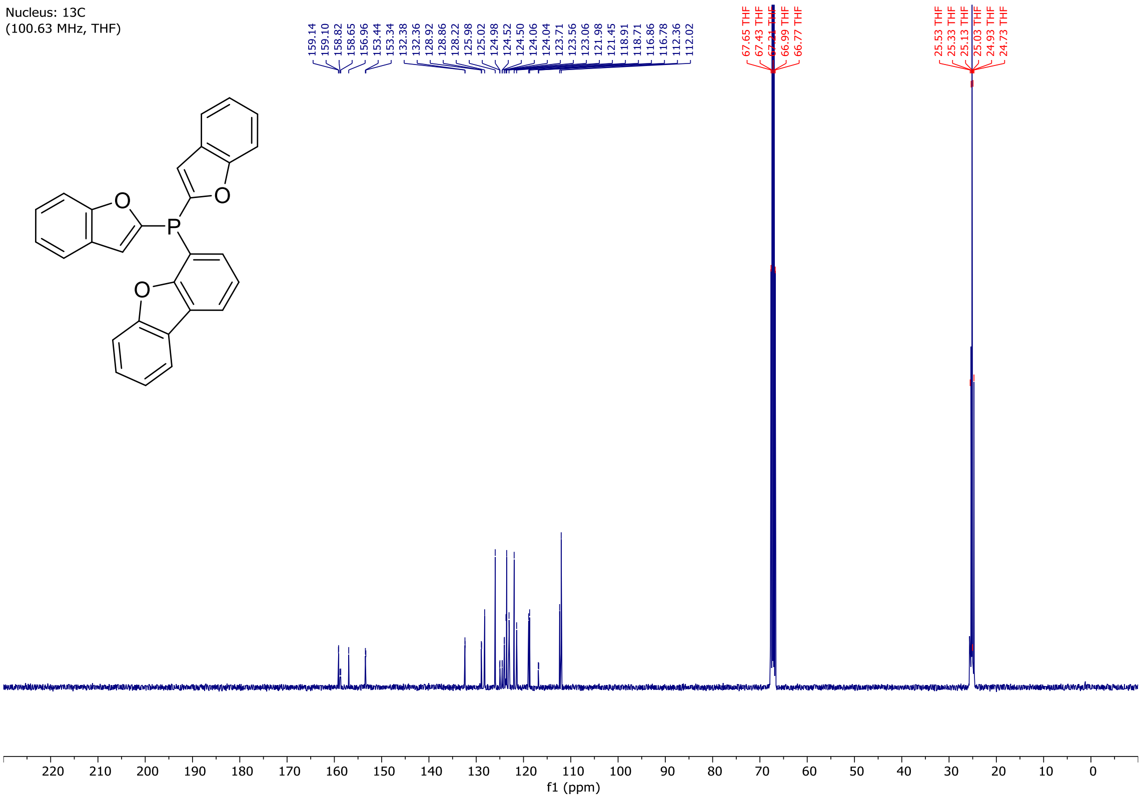

Nucleus: 31P
(161.98 MHz, THF)

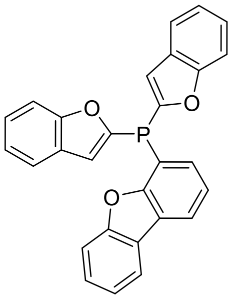

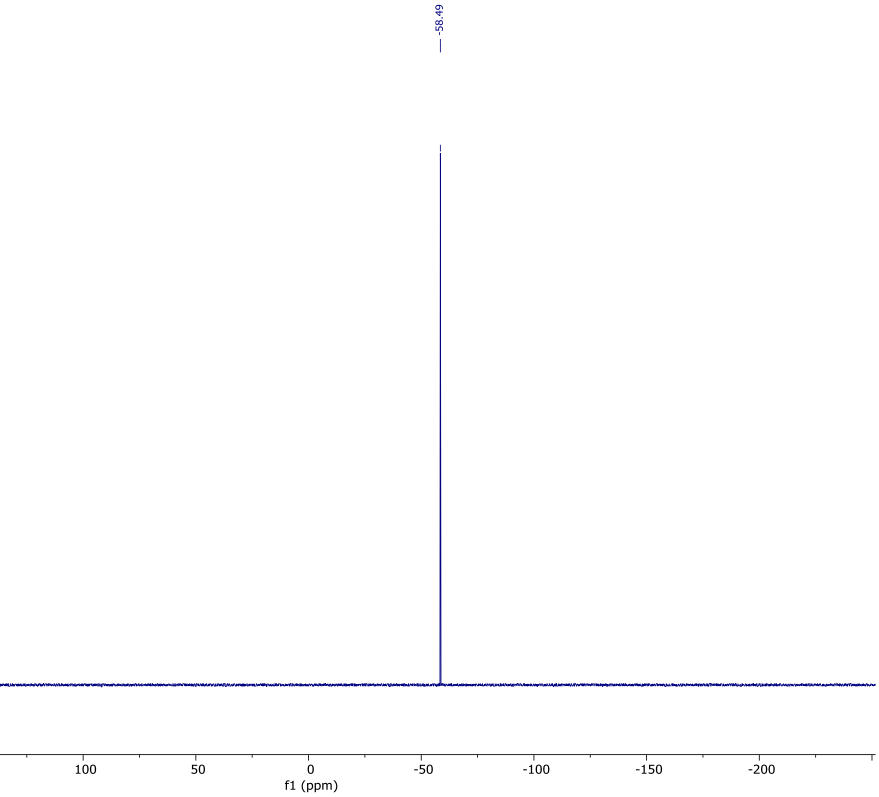

^{1}H NMR, ^{13}C NMR and ^{31}P NMR spectrum of **L25**

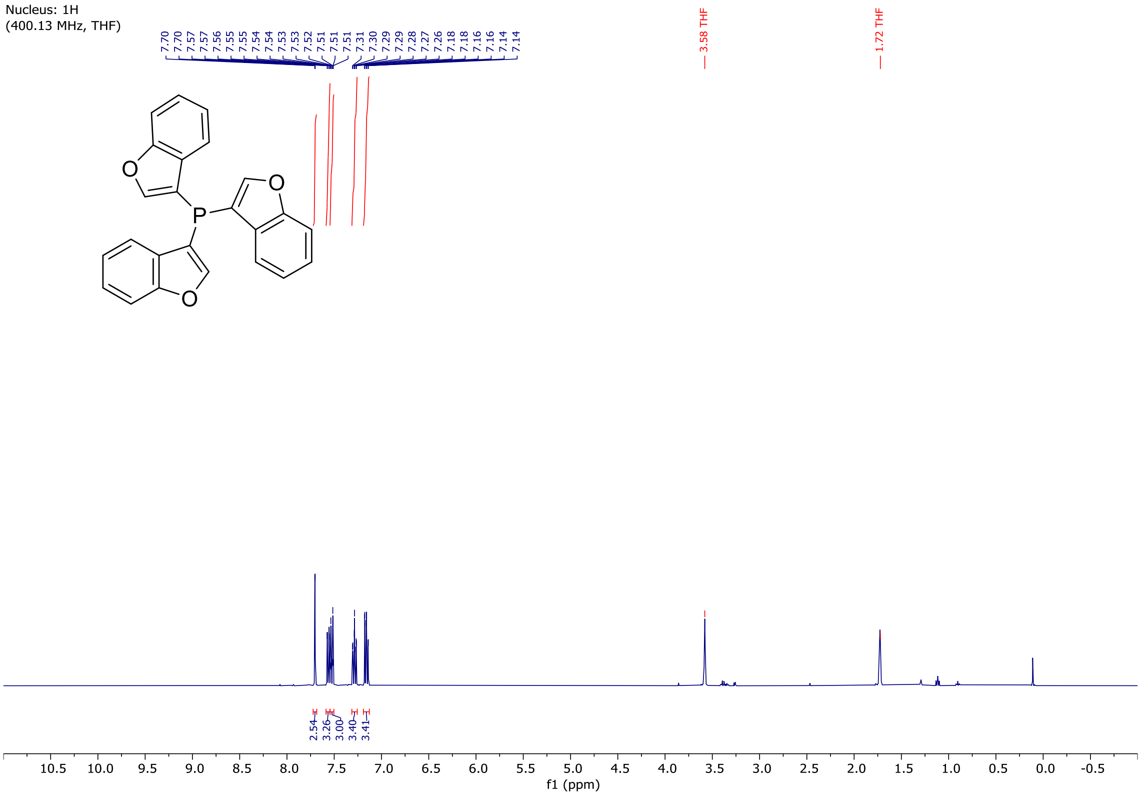

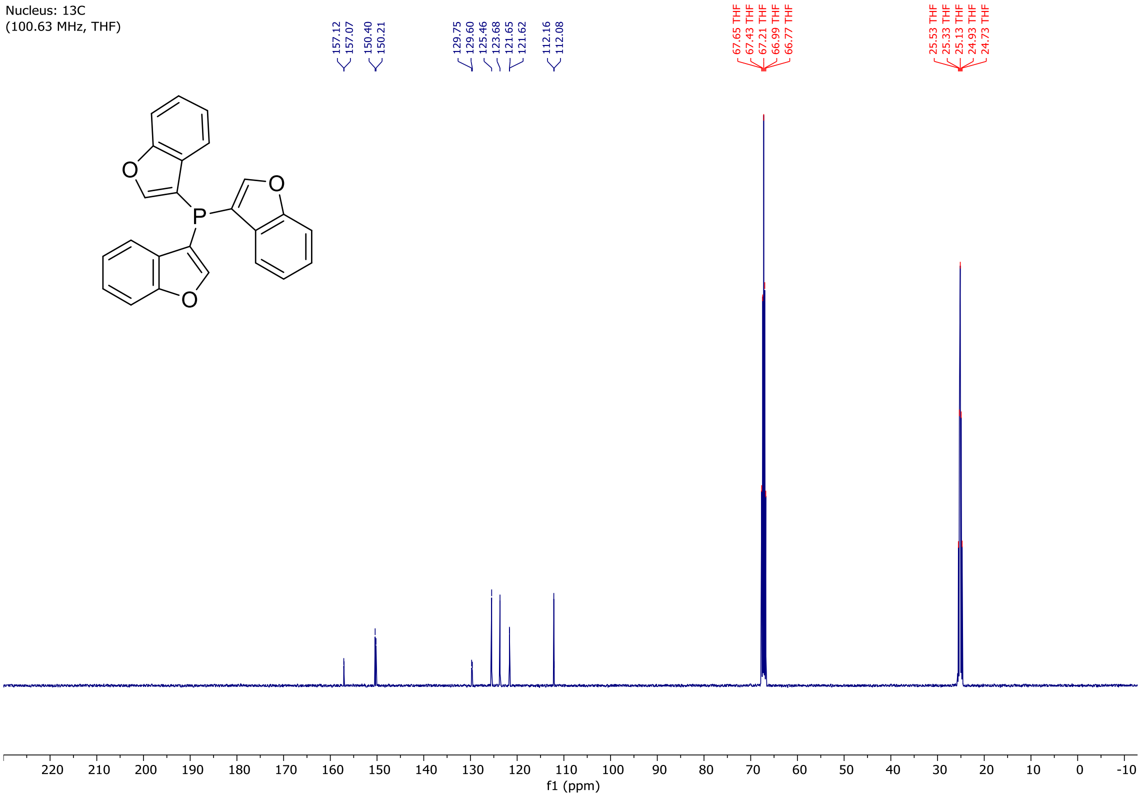

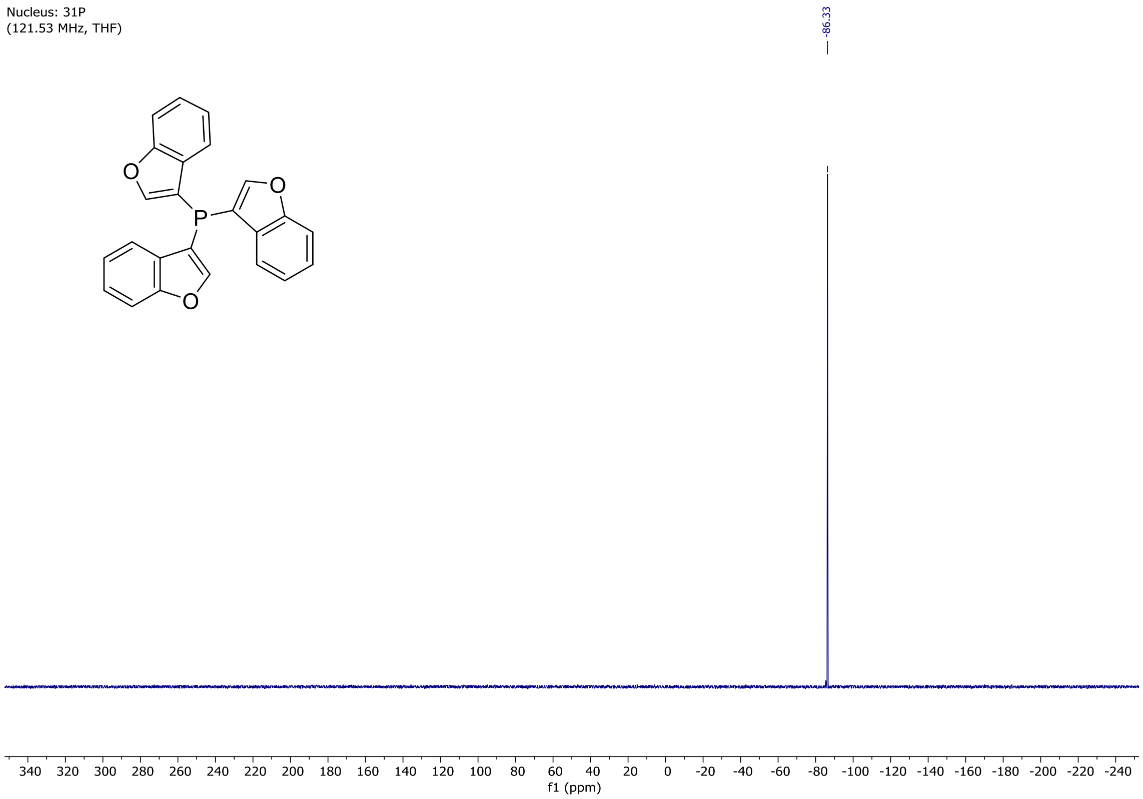
Nucleus: 31P
(121.53 MHz, THF)
-86.33
O
P
O
O
340 320 300 280 260 240 220 200 180 160 140 120 100 80 60 40 20 0 -20 -40 -60 -80 -100 -120 -140 -160 -180 -200 -220 -240
f1 (ppm)

^{1}H NMR, ^{13}C NMR and ^{31}P NMR spectrum of **L26**

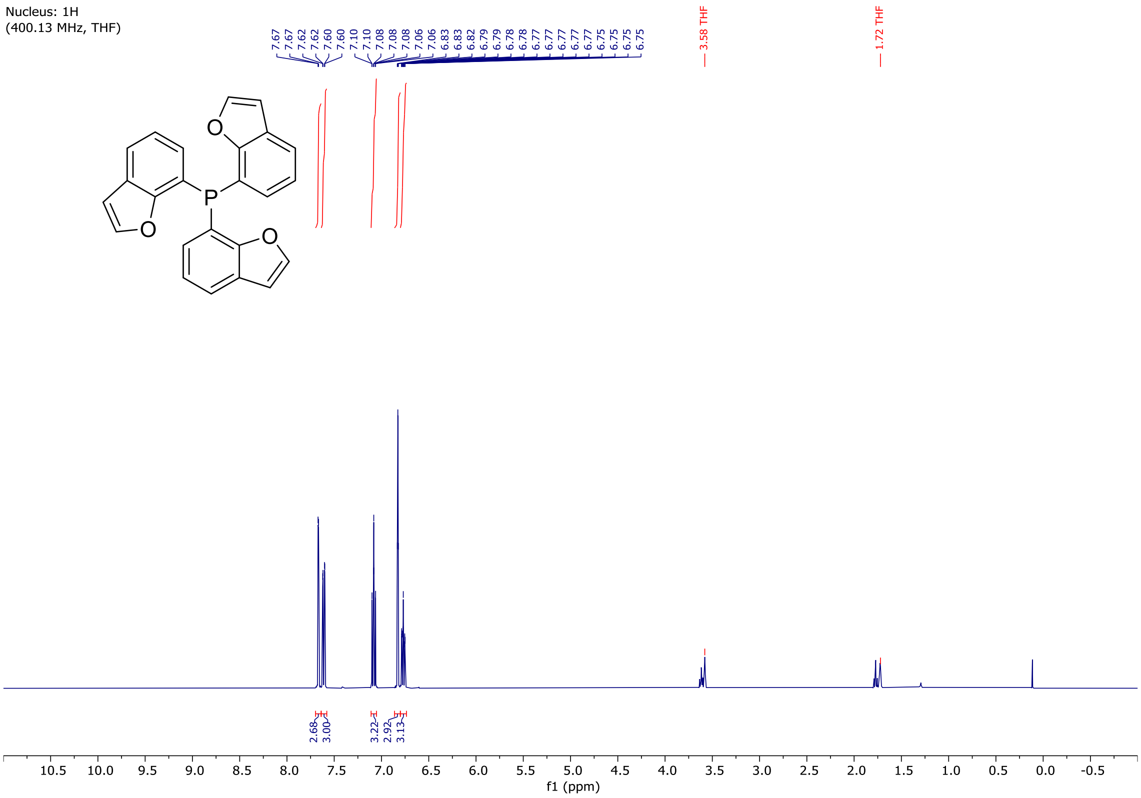

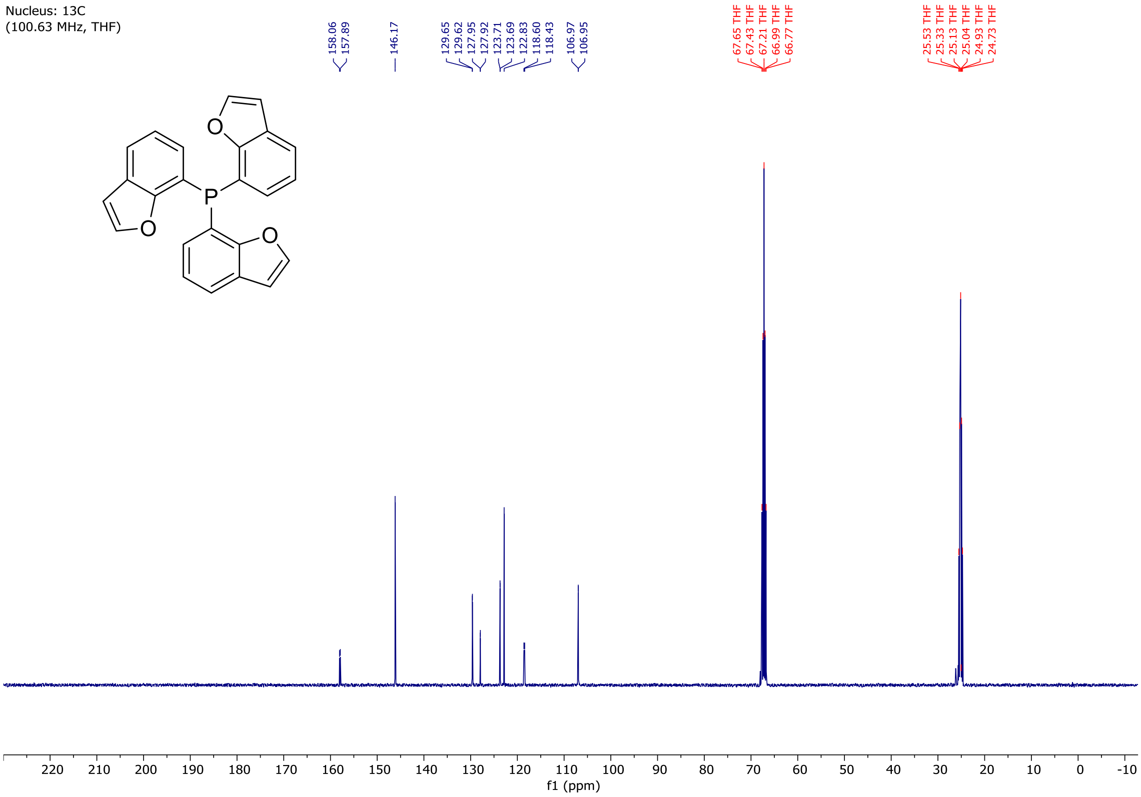

Nucleus: 31P
(161.98 MHz, THF)

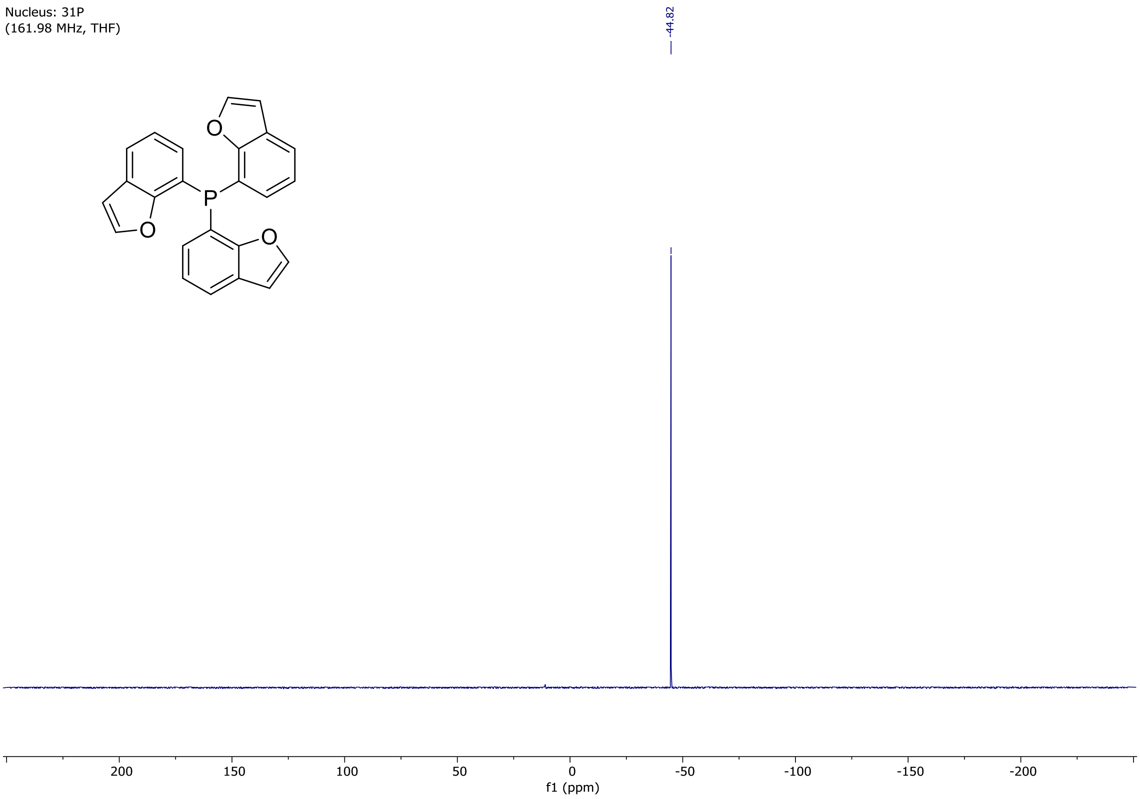

^{1}H NMR, ^{13}C NMR and ^{31}P NMR spectrum of **L27**

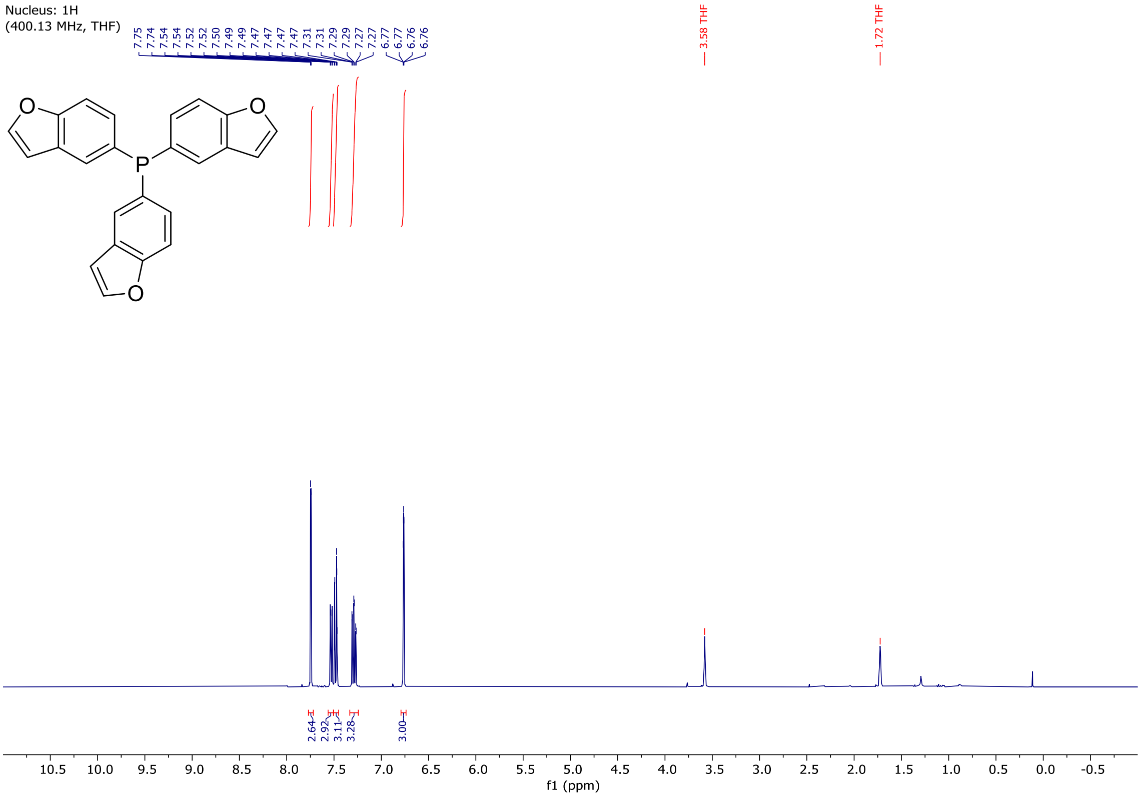

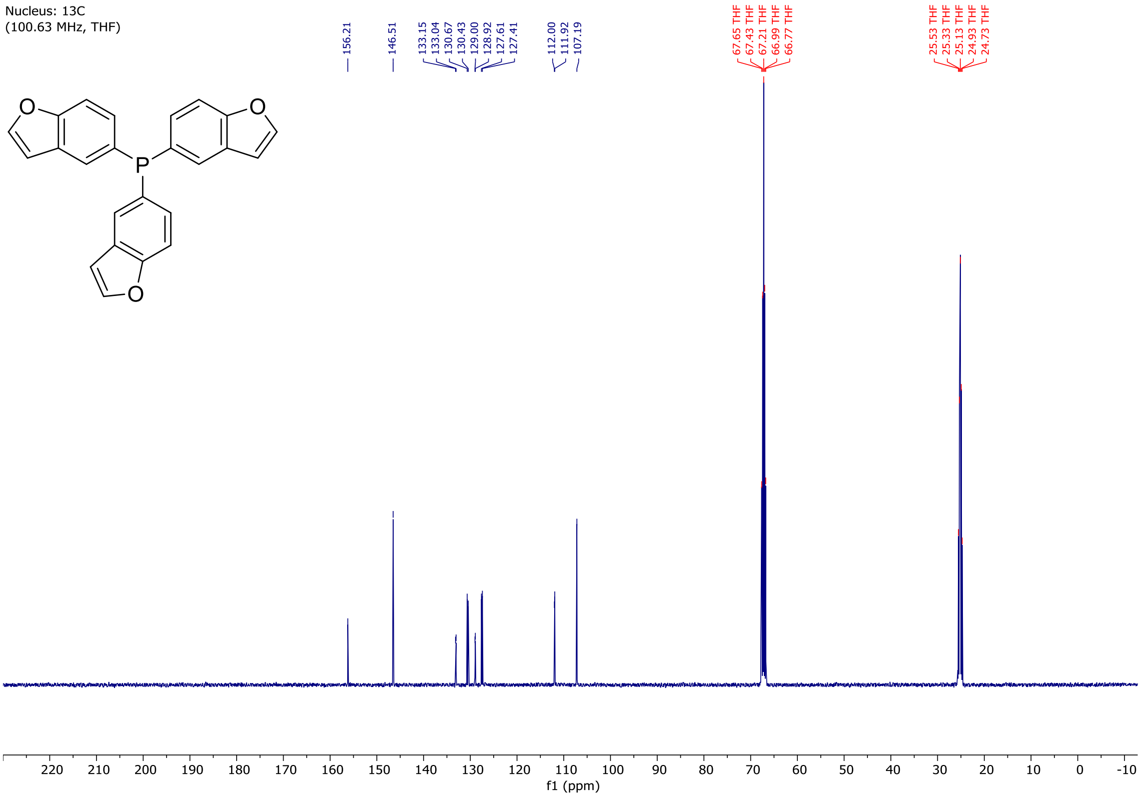

Nucleus: 31P
(121.53 MHz, THF)

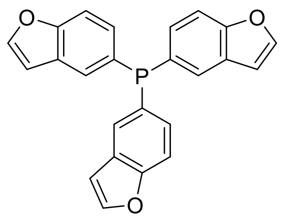

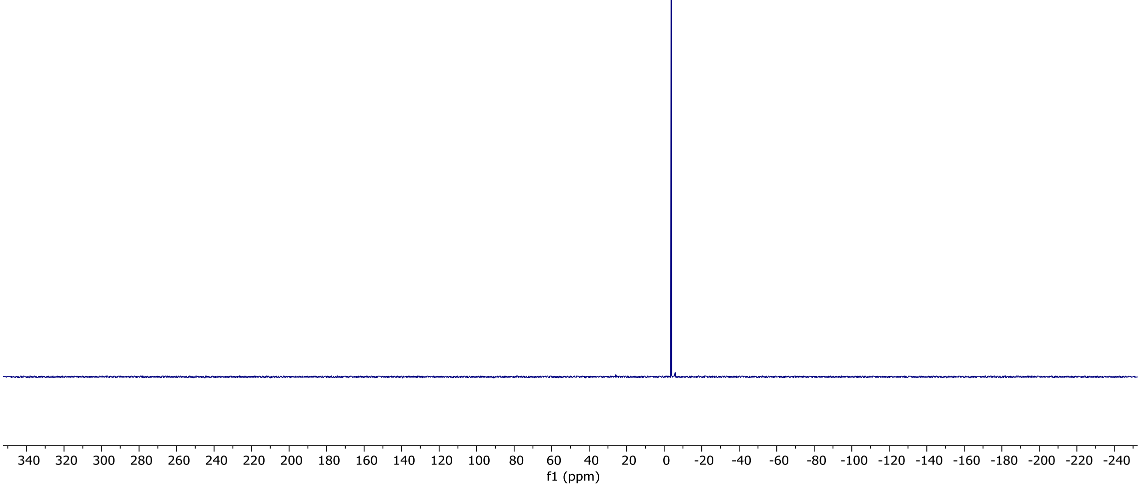

13.2 Allylamines Derivatives

^{1}H NMR and ^{13}C NMR spectrum of compound ***(E)*-1a**

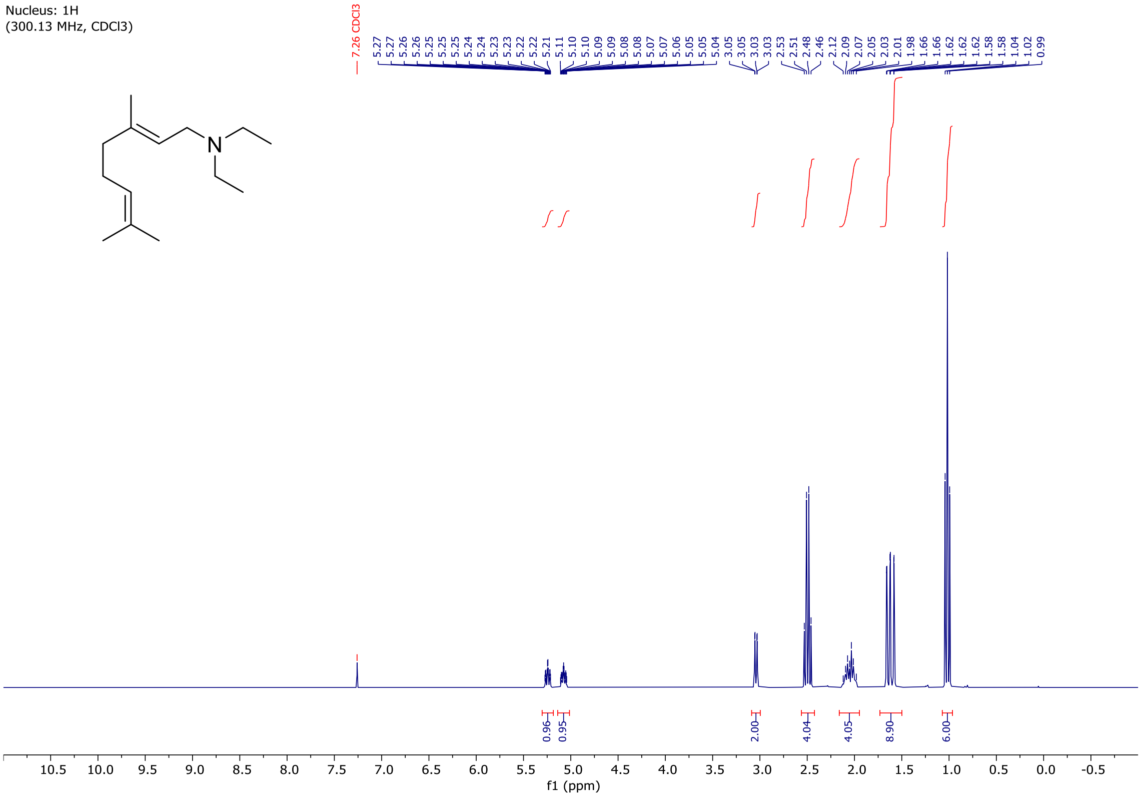

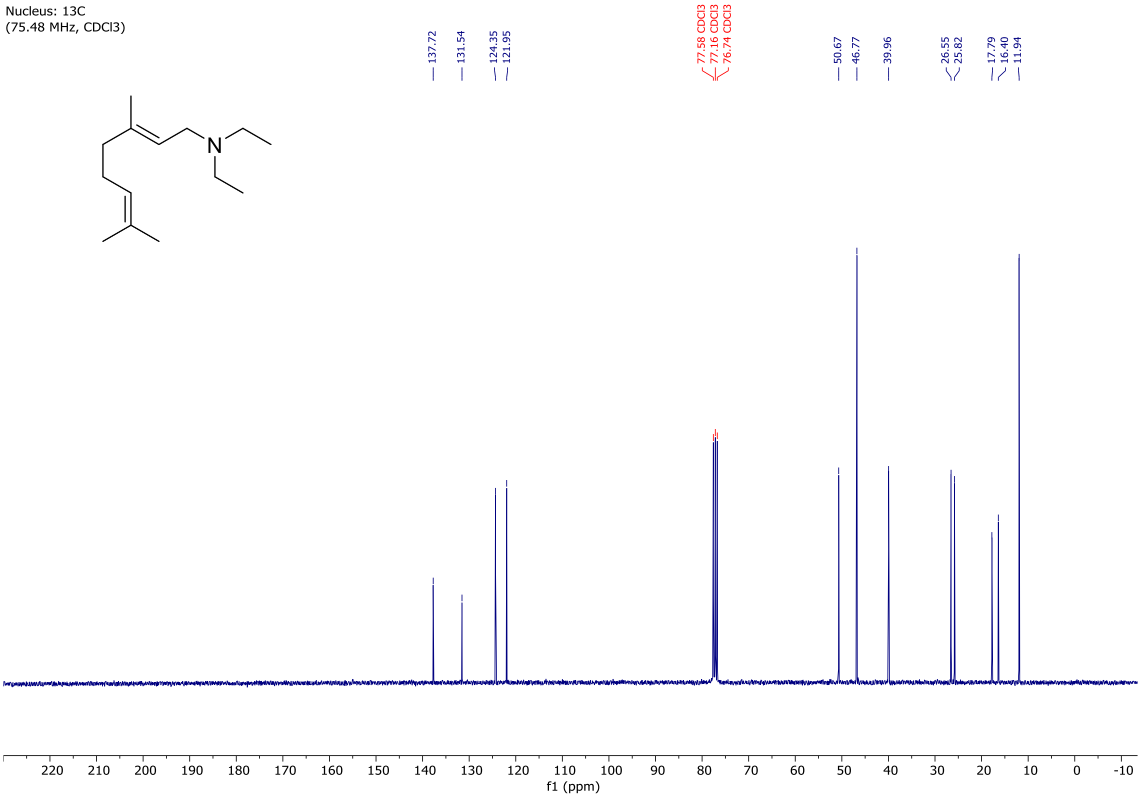

^{1}H NMR and ^{13}C NMR spectrum of compound **(*Z*)-1a**

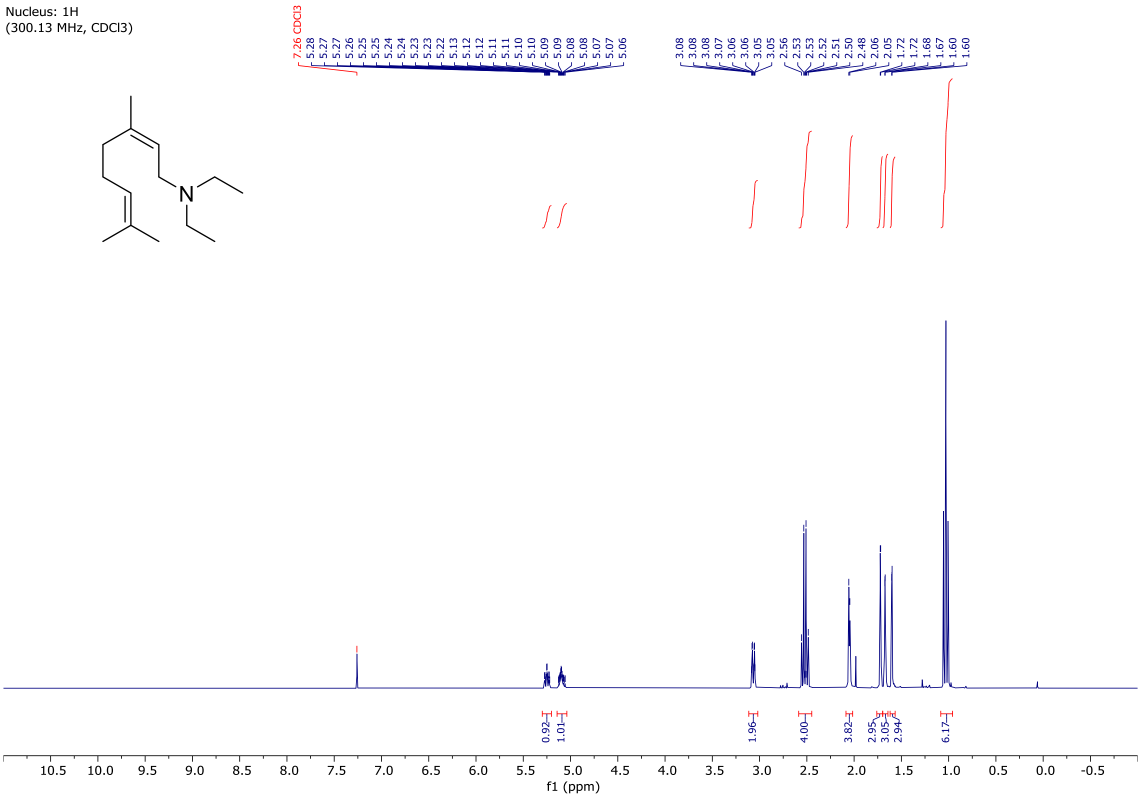

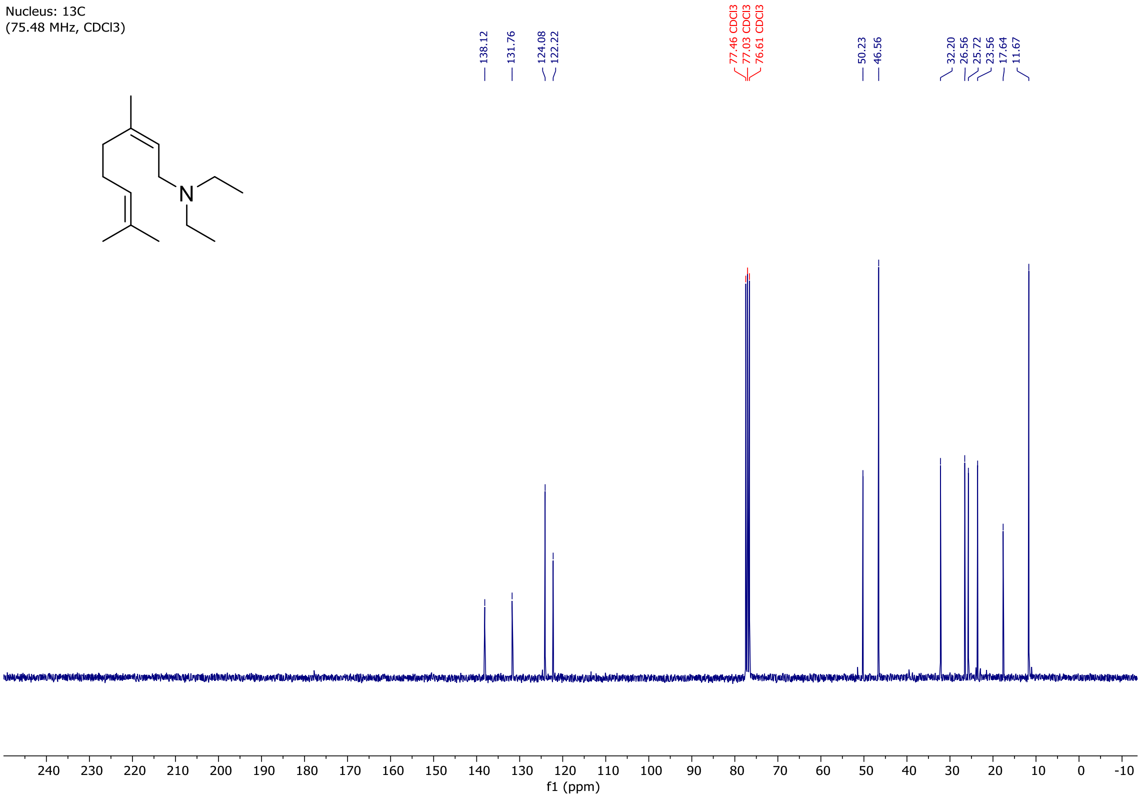

^{1}H NMR and ^{13}C NMR spectrum of compound **2a**

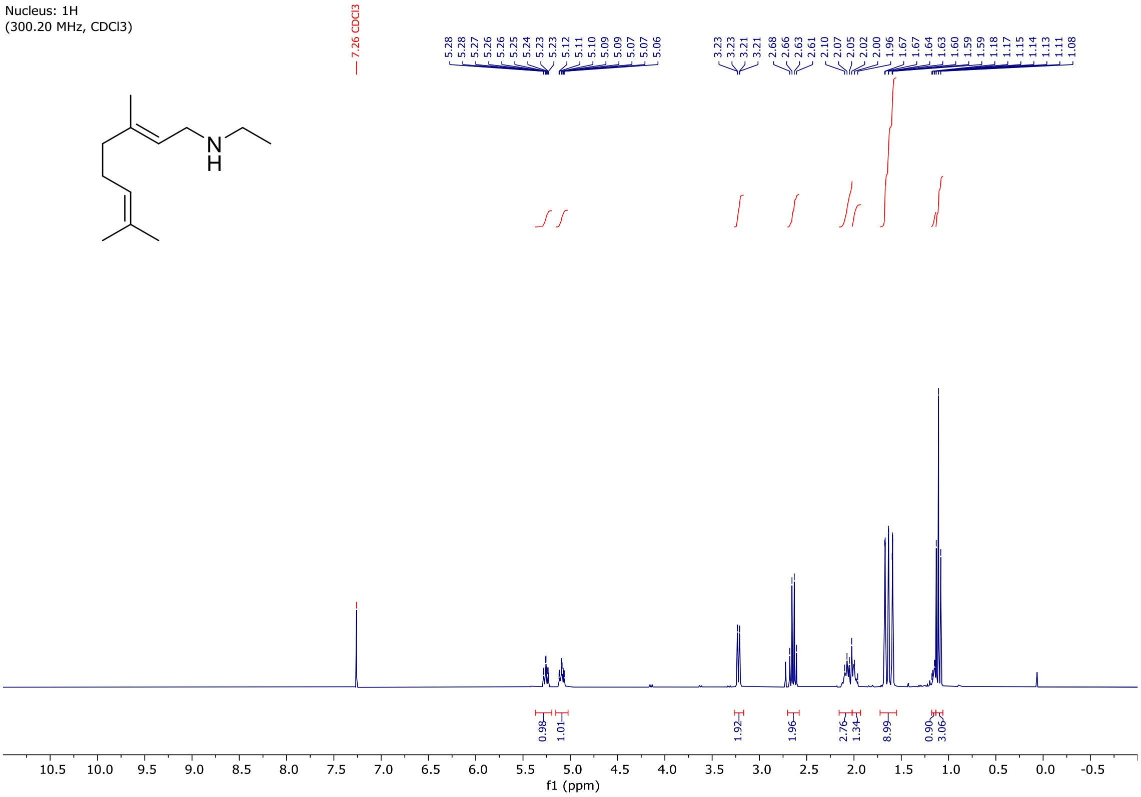

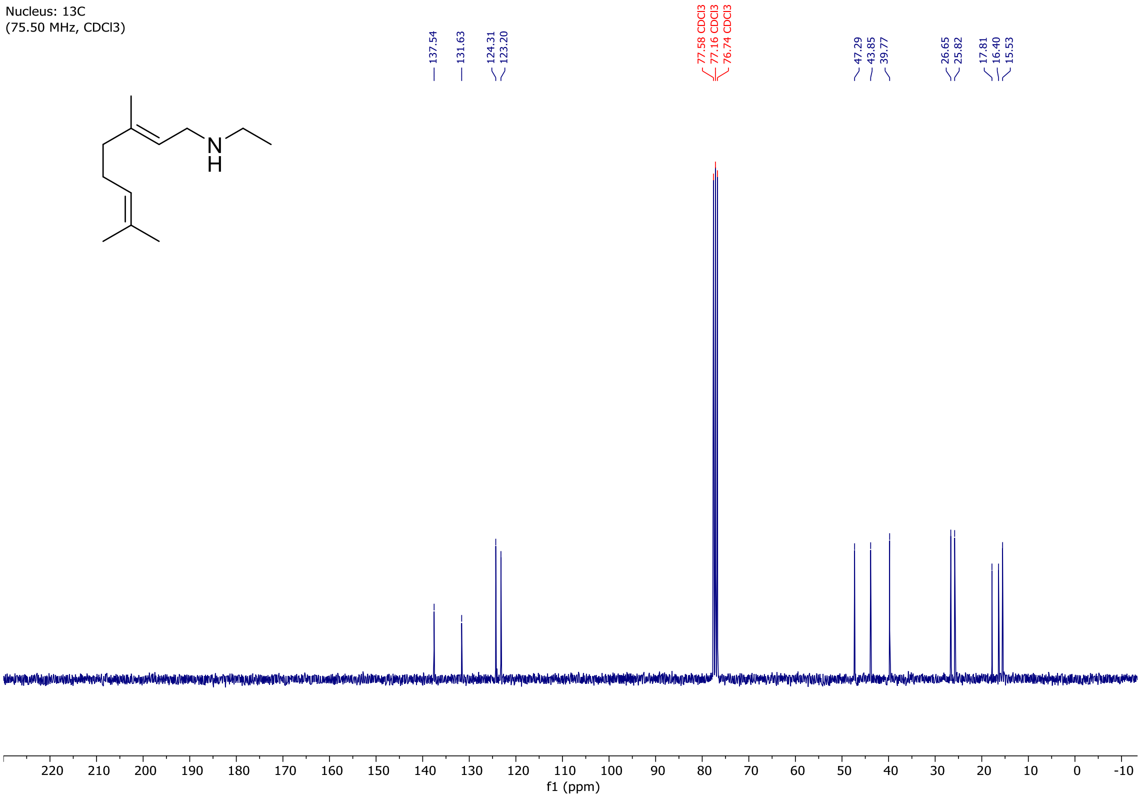

^{1}H NMR and ^{13}C NMR spectrum of compound **3a**

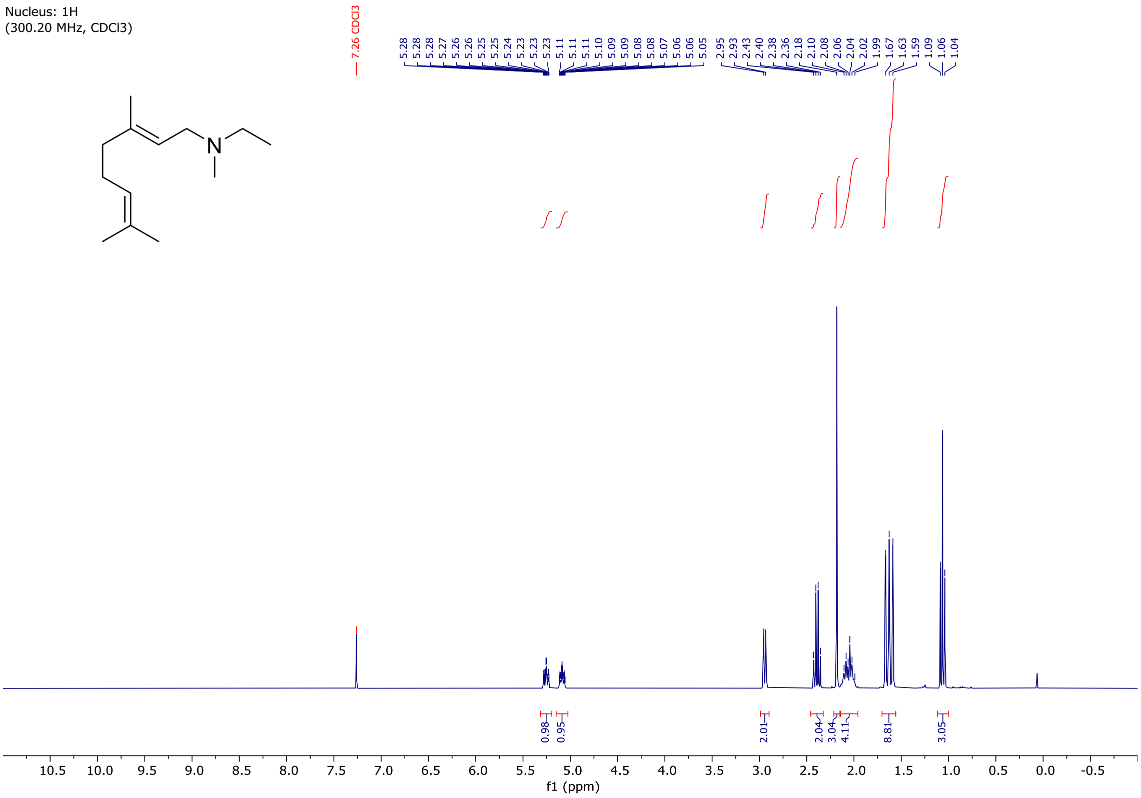

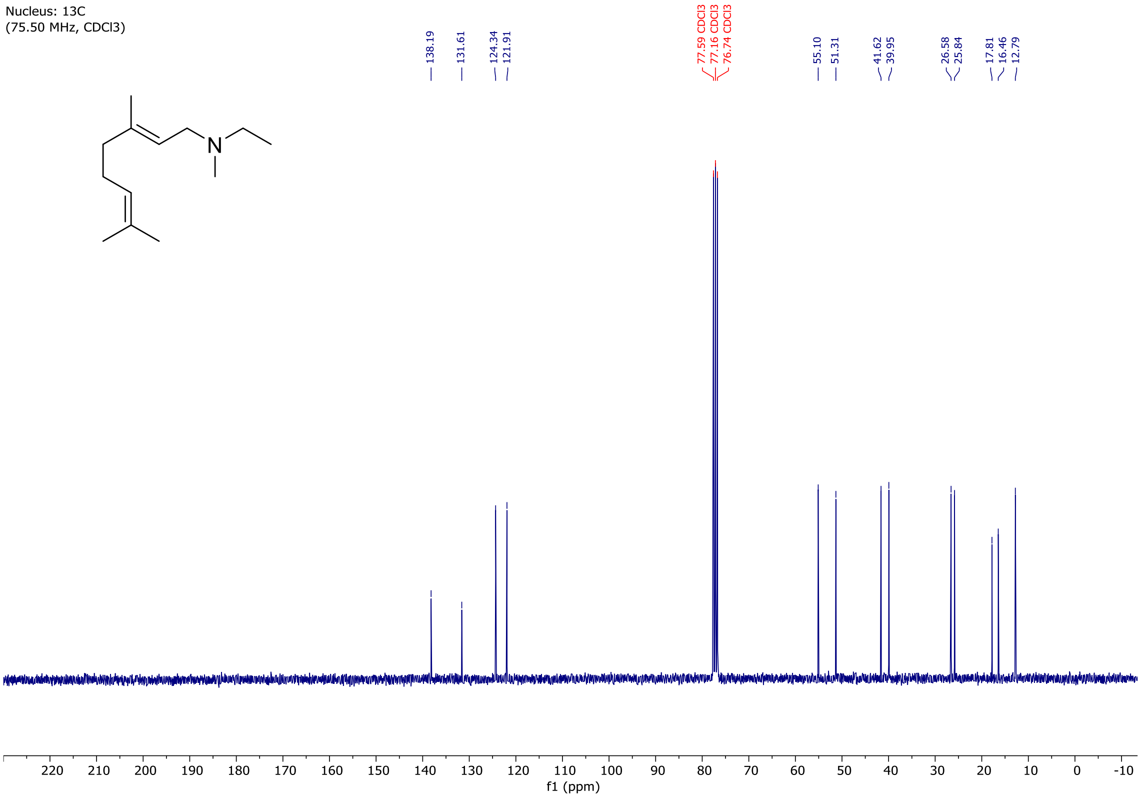

^{1}H NMR and ^{13}C NMR spectrum of compound **4a**

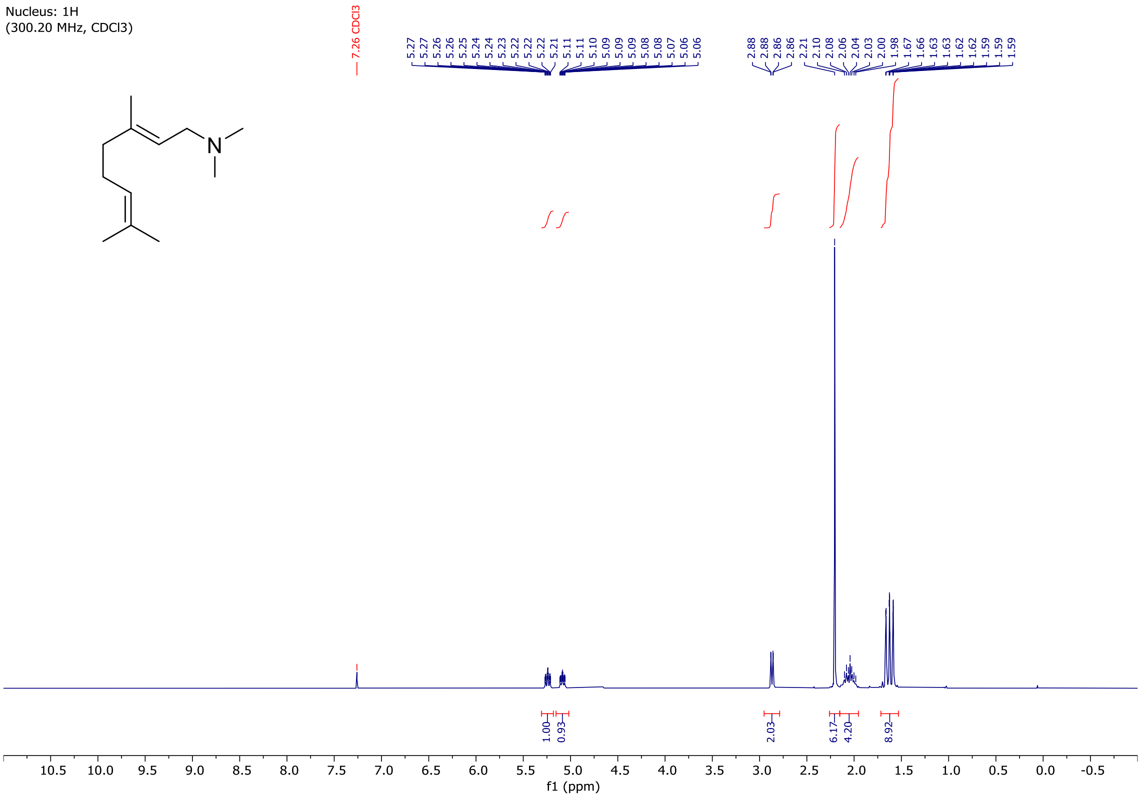

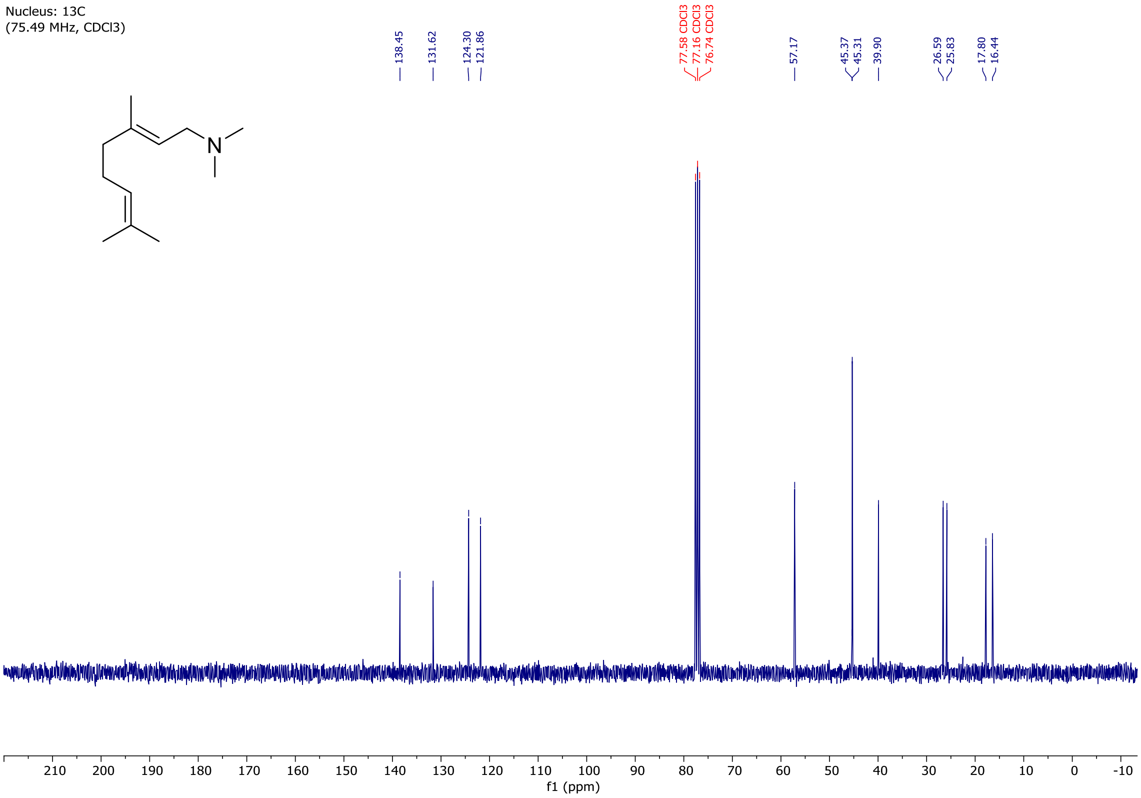

^{1}H NMR and ^{13}C NMR spectrum of compound **5a**

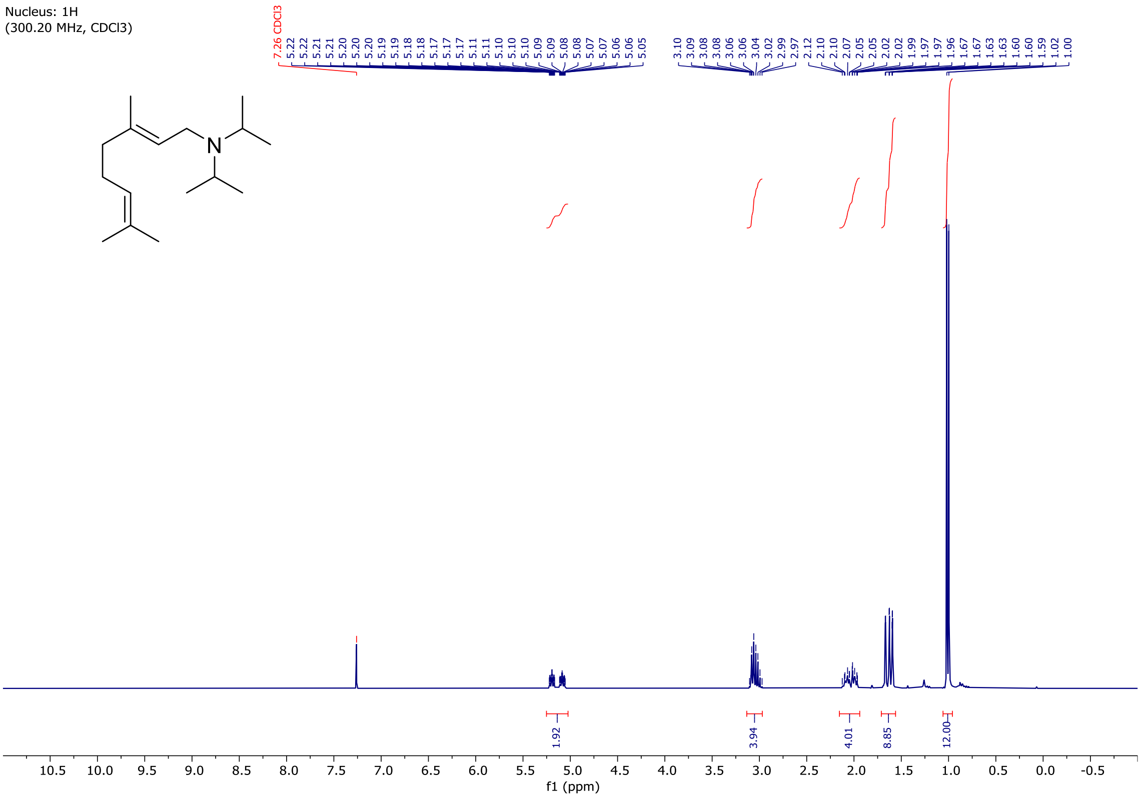

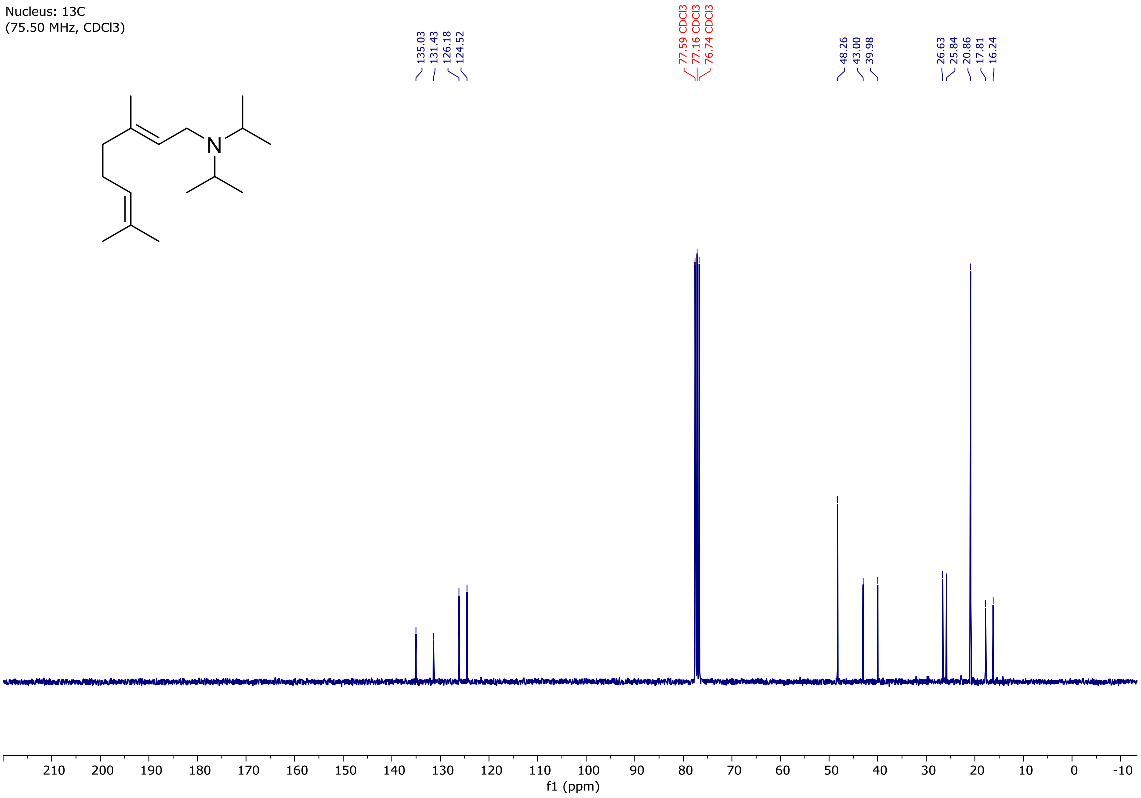

^{1}H NMR and ^{13}C NMR spectrum of compound **6a**

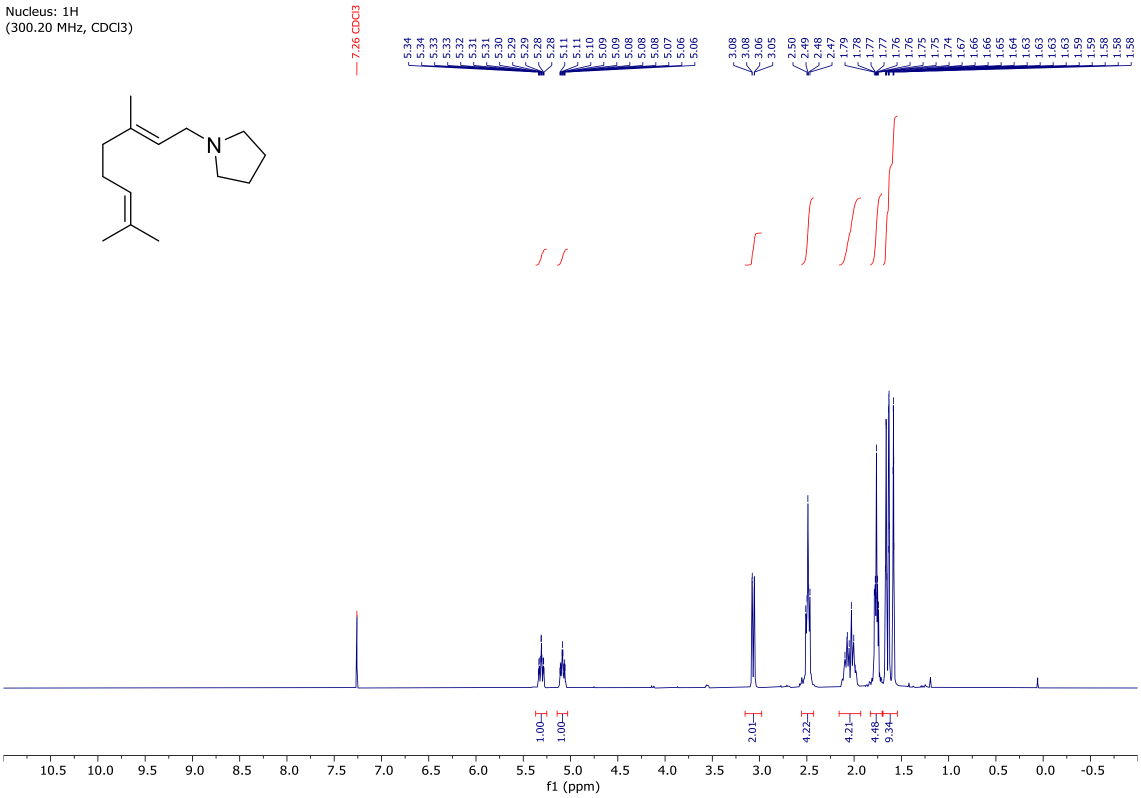

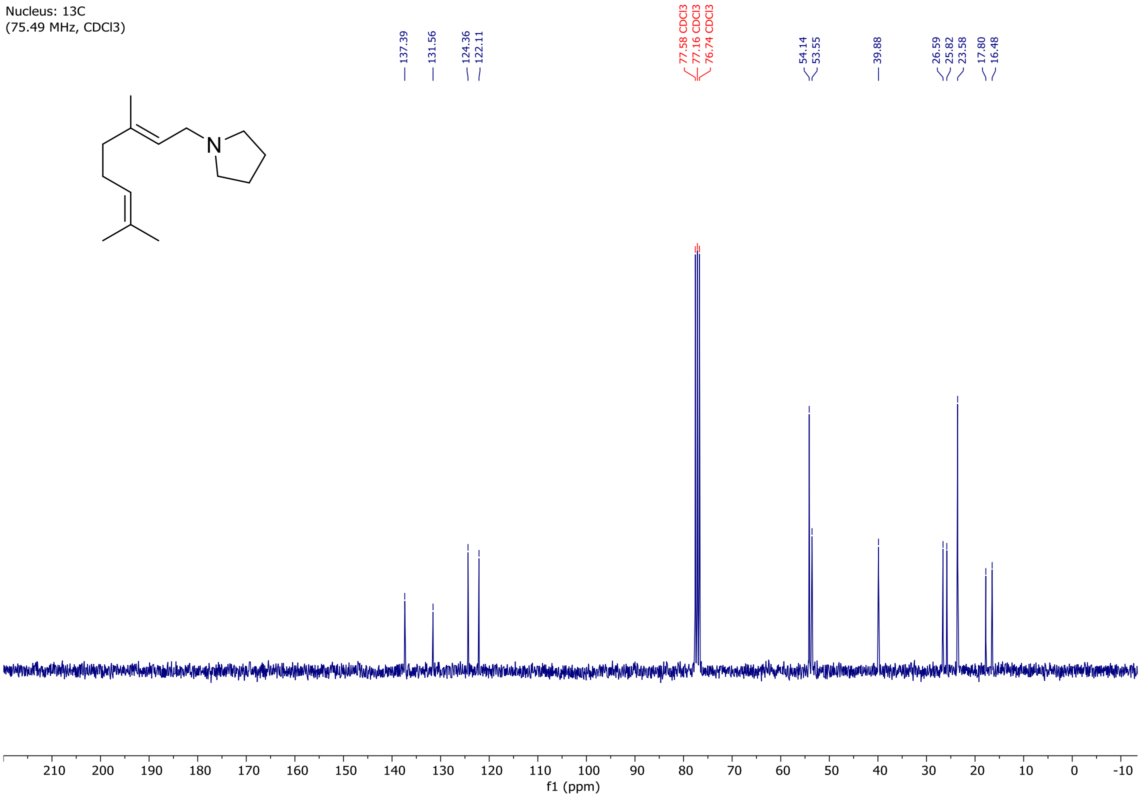

1H NMR and 13C NMR spectrum of compound **7a**

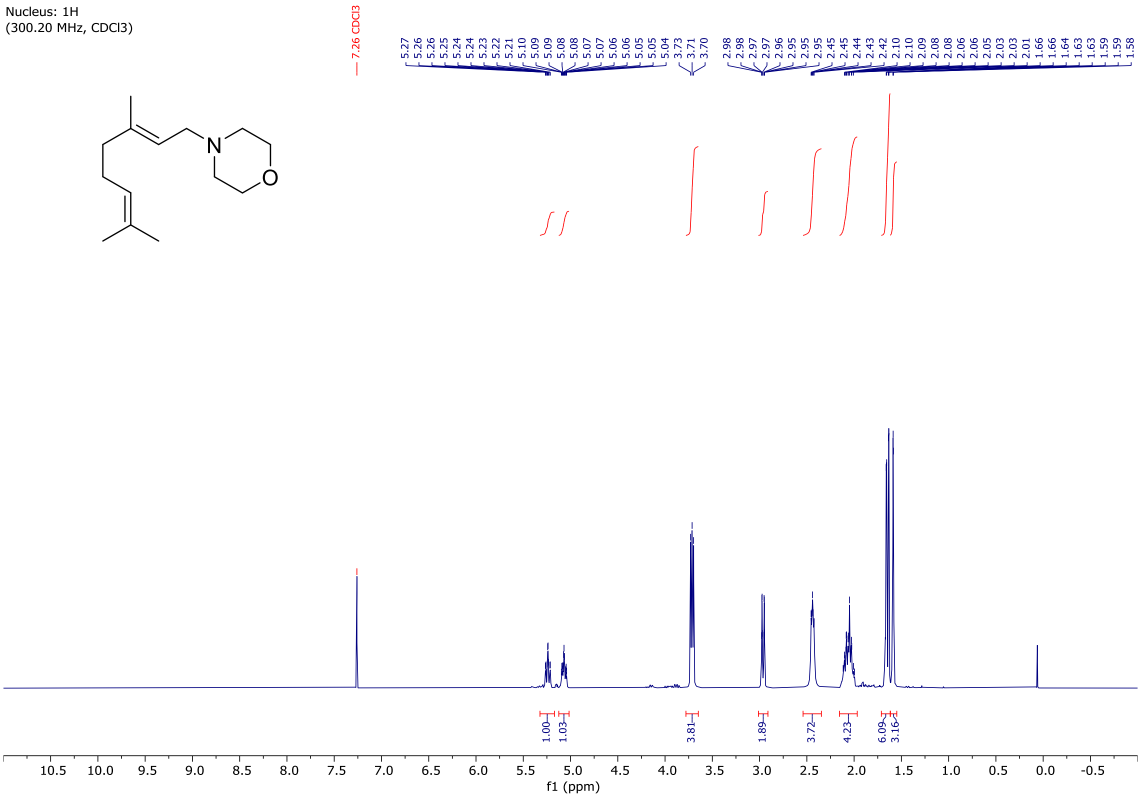

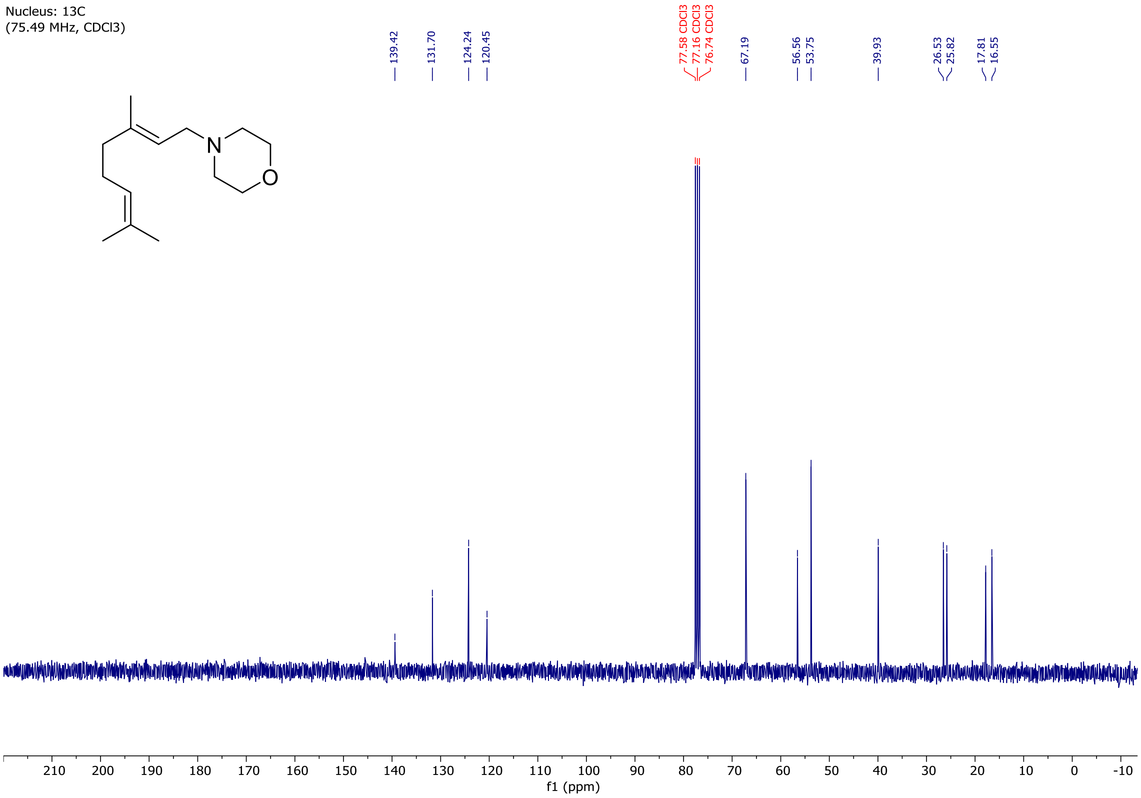

^{1}H NMR and ^{13}C NMR spectrum of compound **8a**

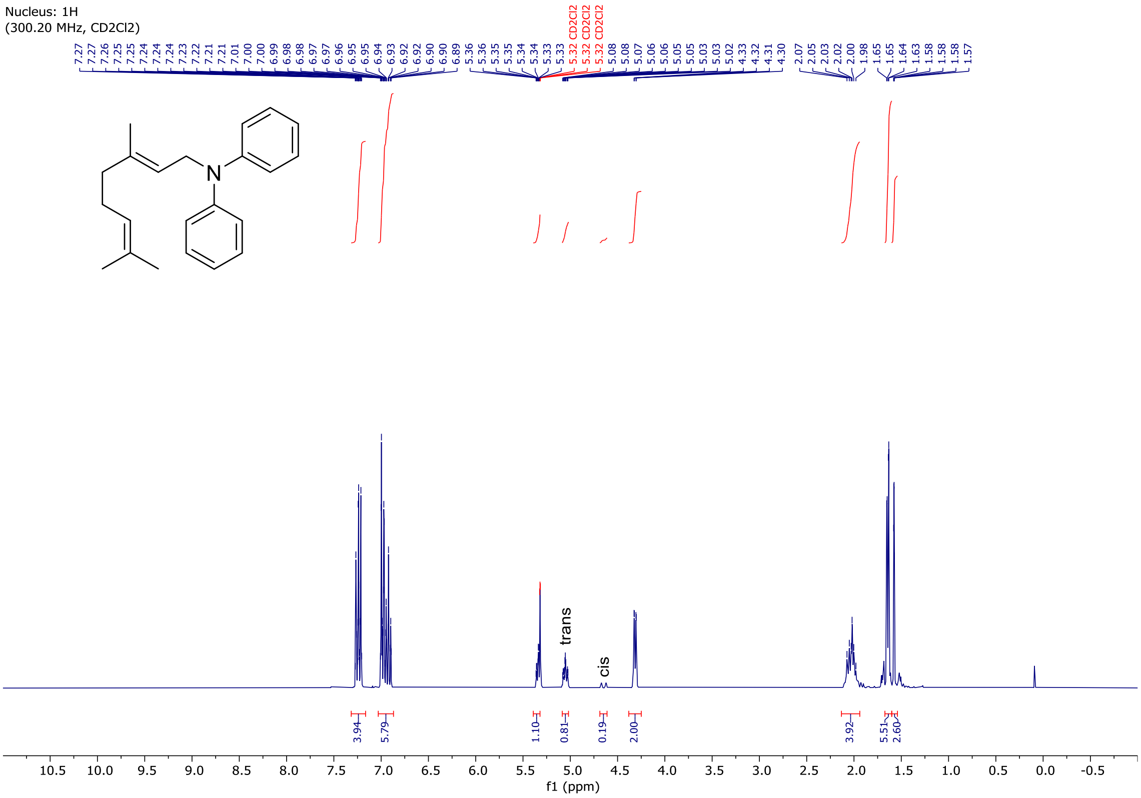

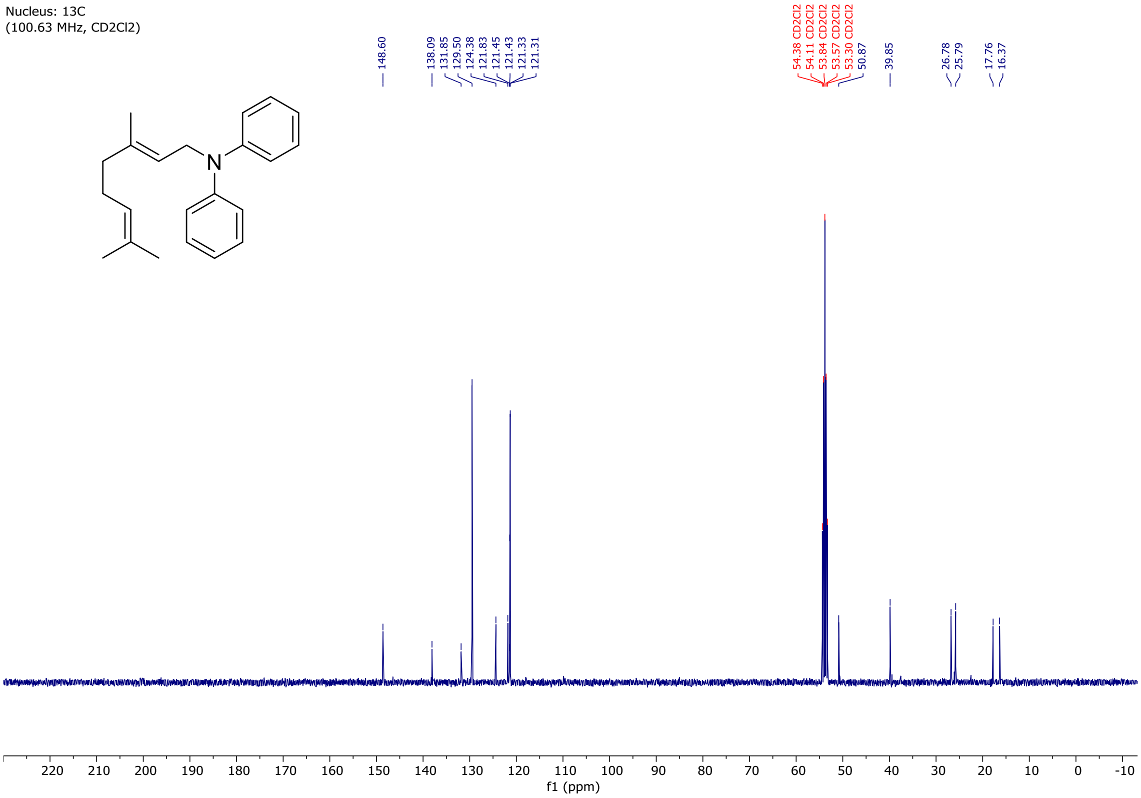

^{1}H NMR and ^{13}C NMR spectrum of compound **9a**

Nucleus: 1H
(300.20 MHz, CD2Cl2)

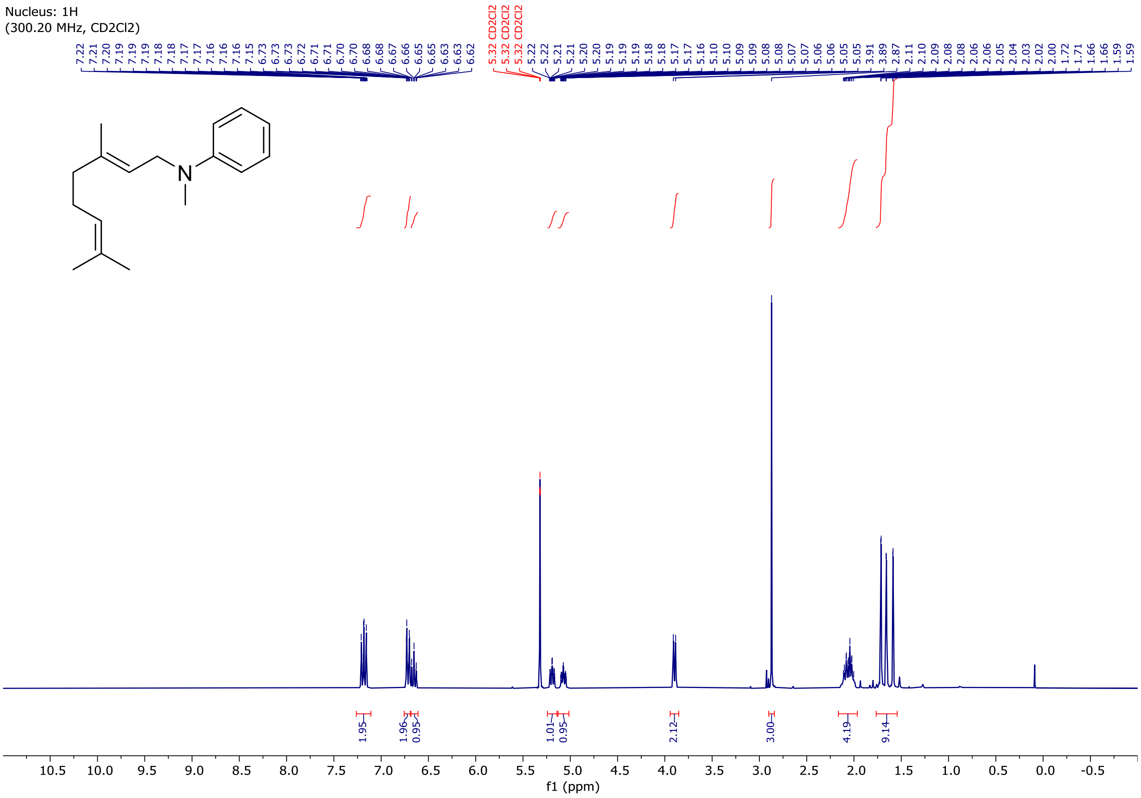

Nucleus: 13C
(75.50 MHz, CD2Cl2)

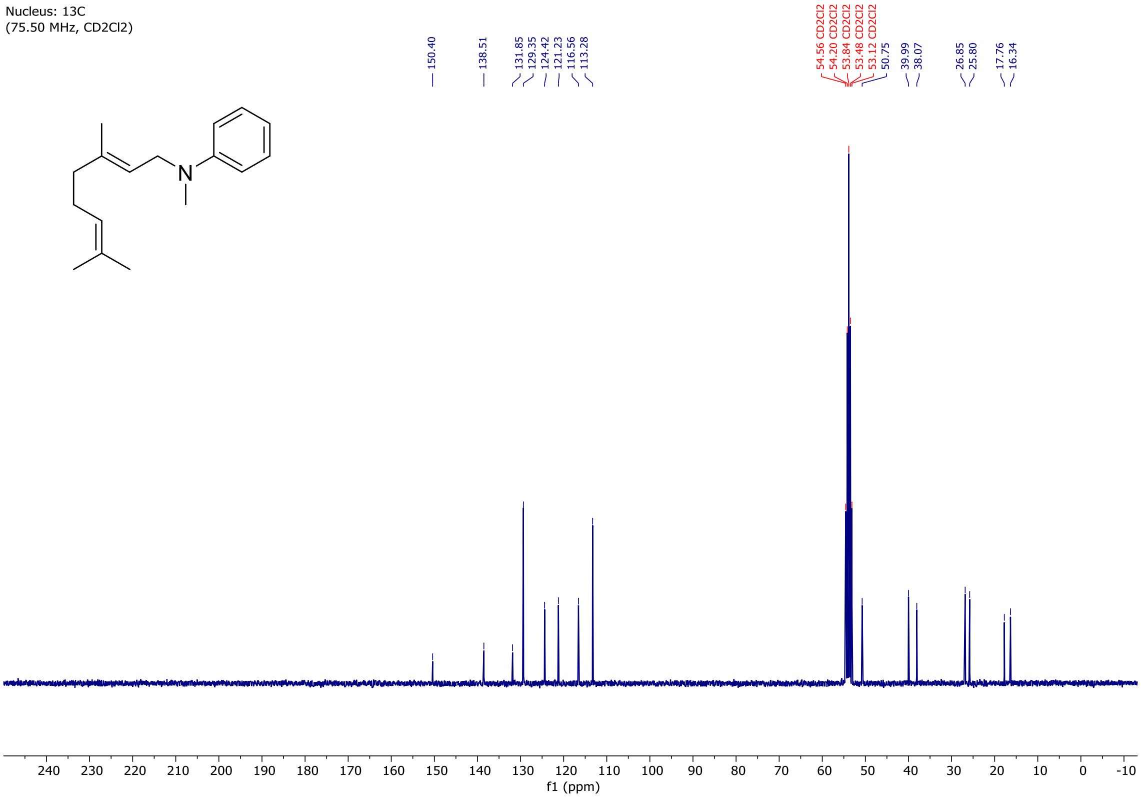

^{1}H NMR and ^{13}C NMR spectrum of compound **10a**

Nucleus: 1H
(300.20 MHz, CDCl3)

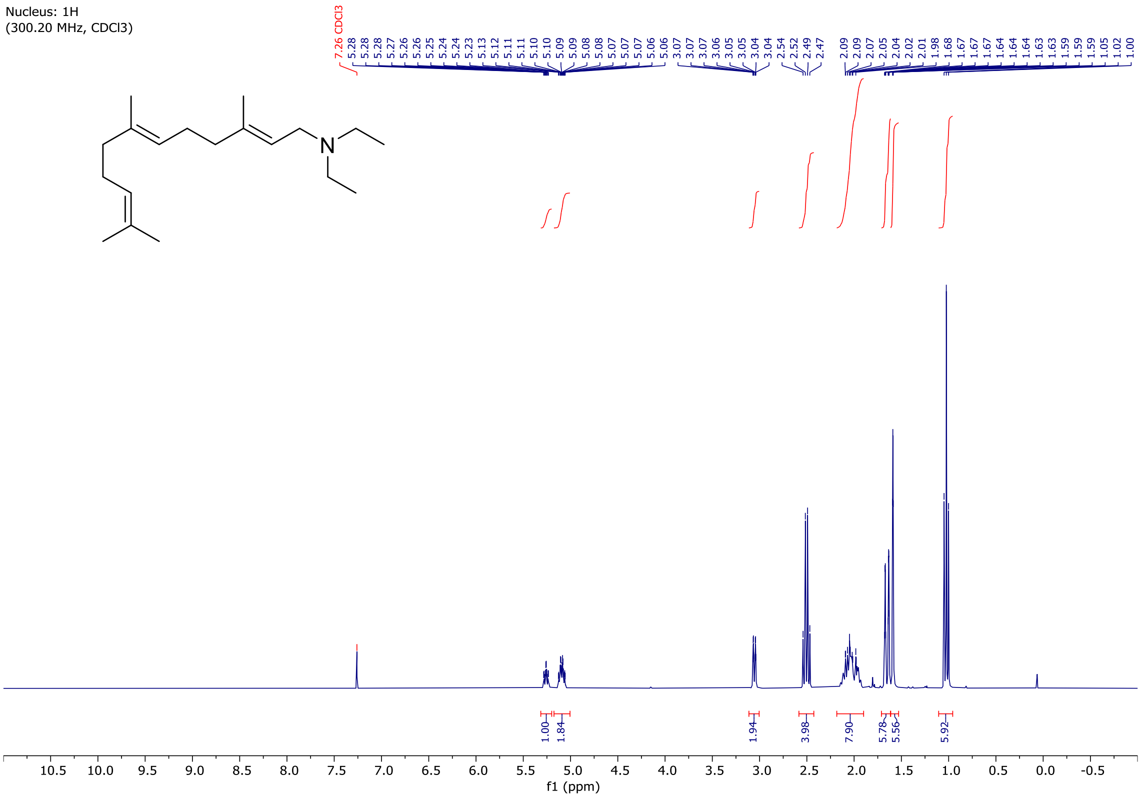

Nucleus: 13C
(75.49 MHz, CDCl3)

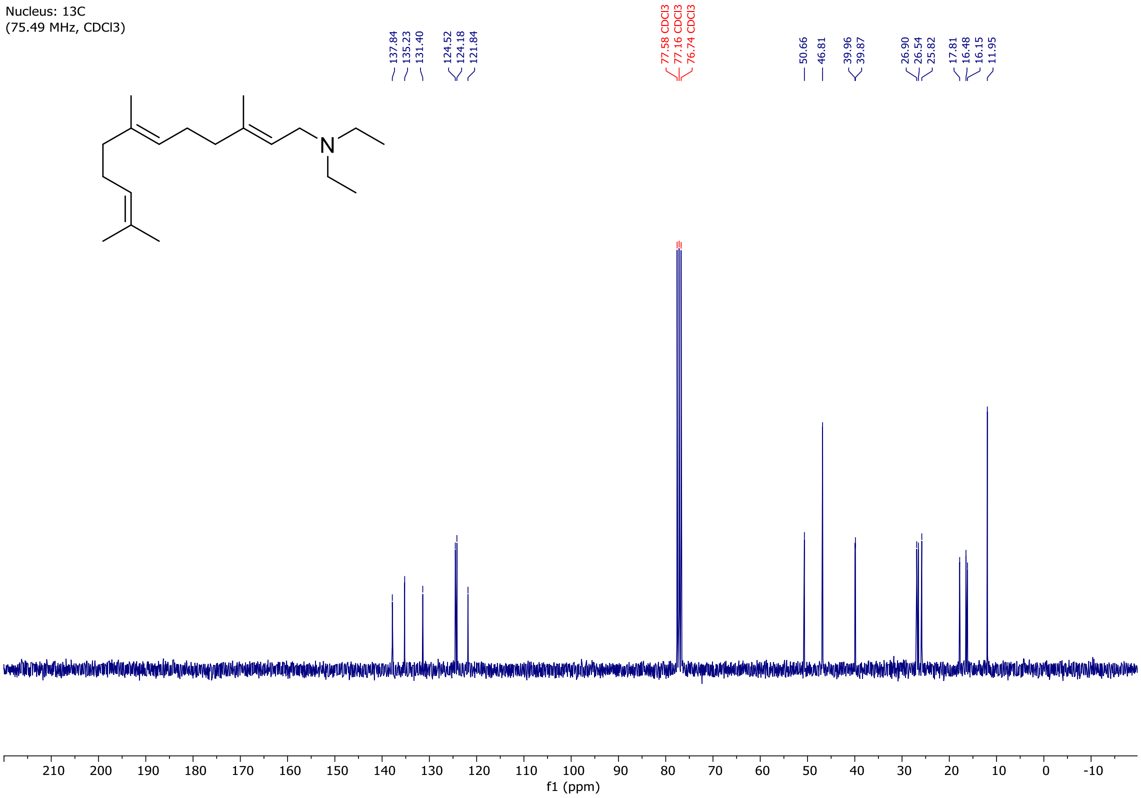

^{1}H NMR and ^{13}C NMR spectrum of compound **11a**

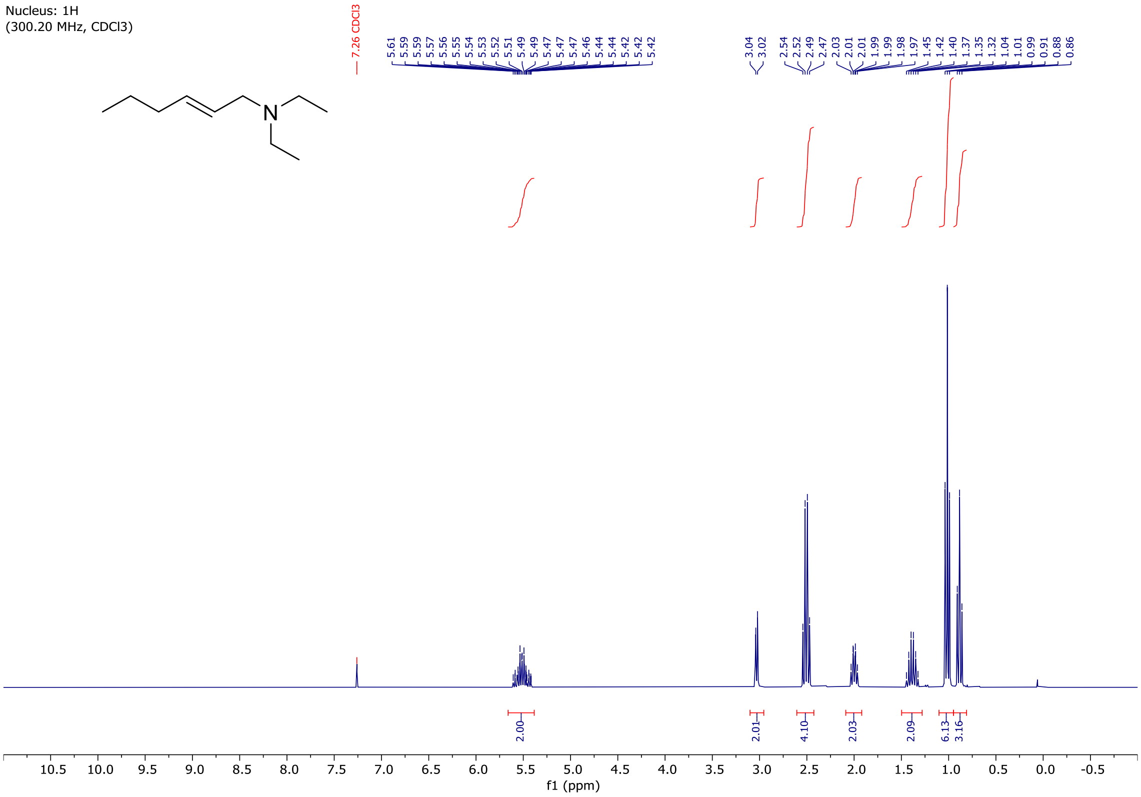

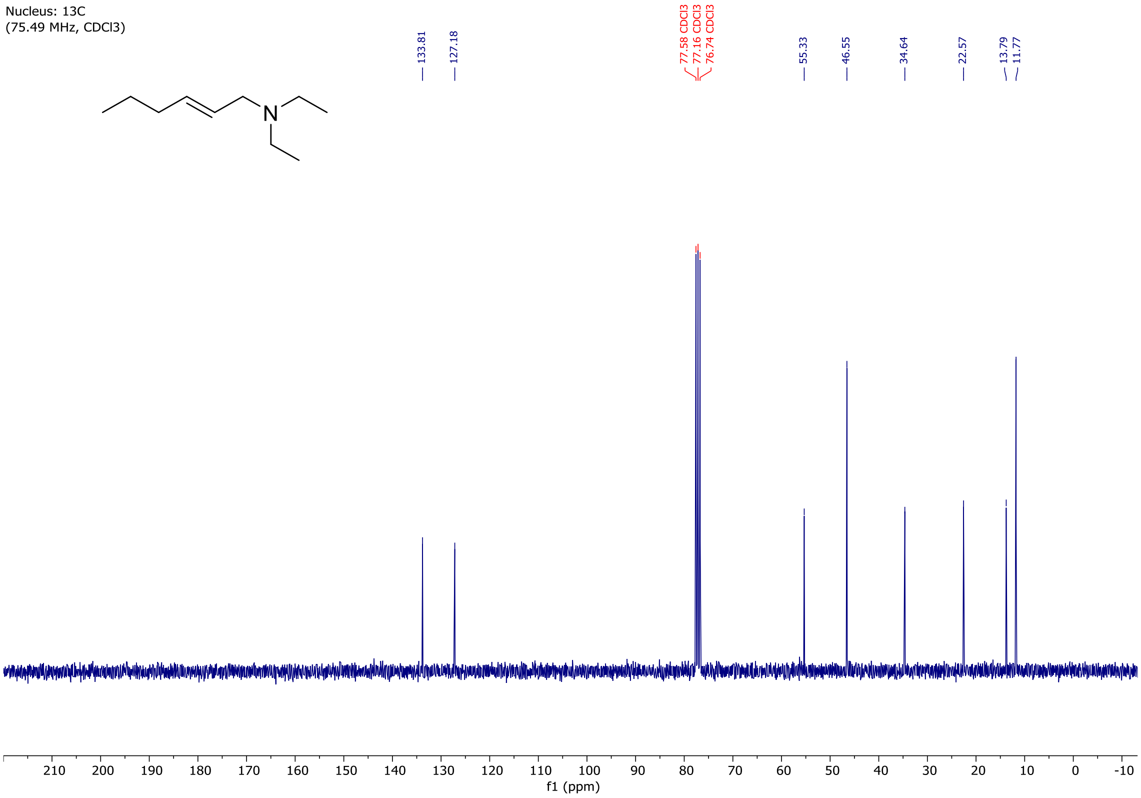

^{1}H NMR and ^{13}C NMR spectrum of compound **12a**

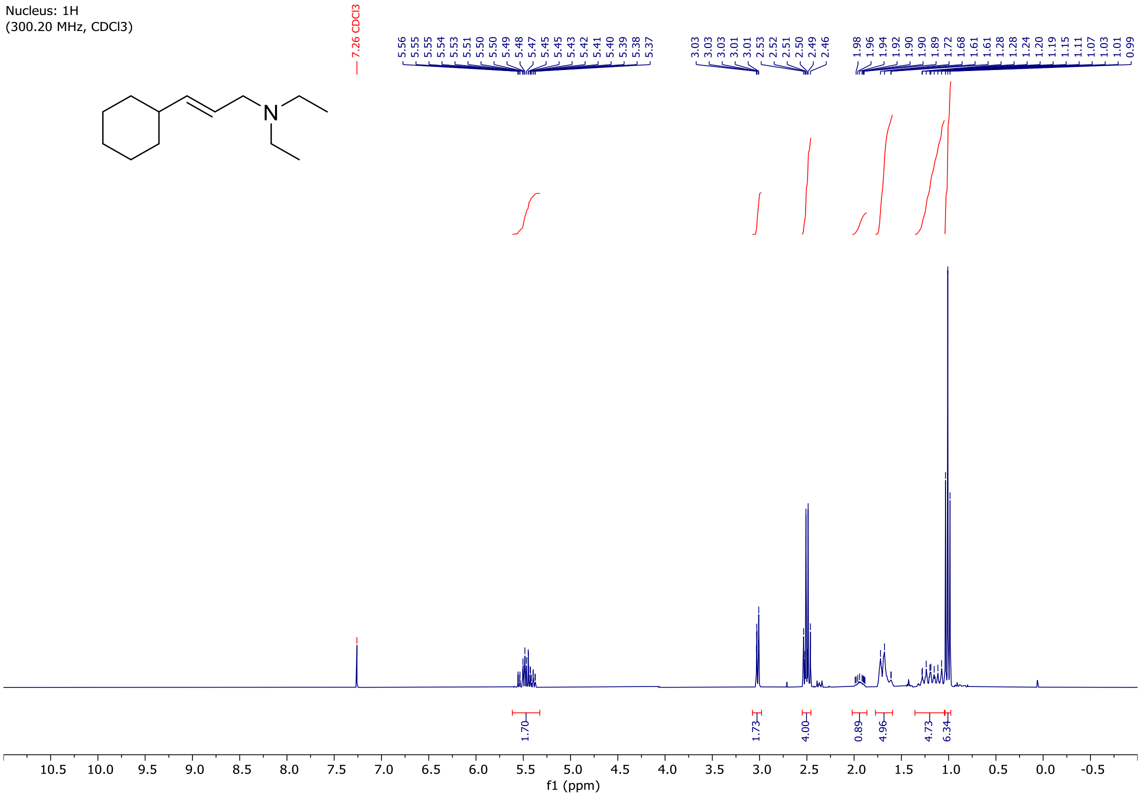

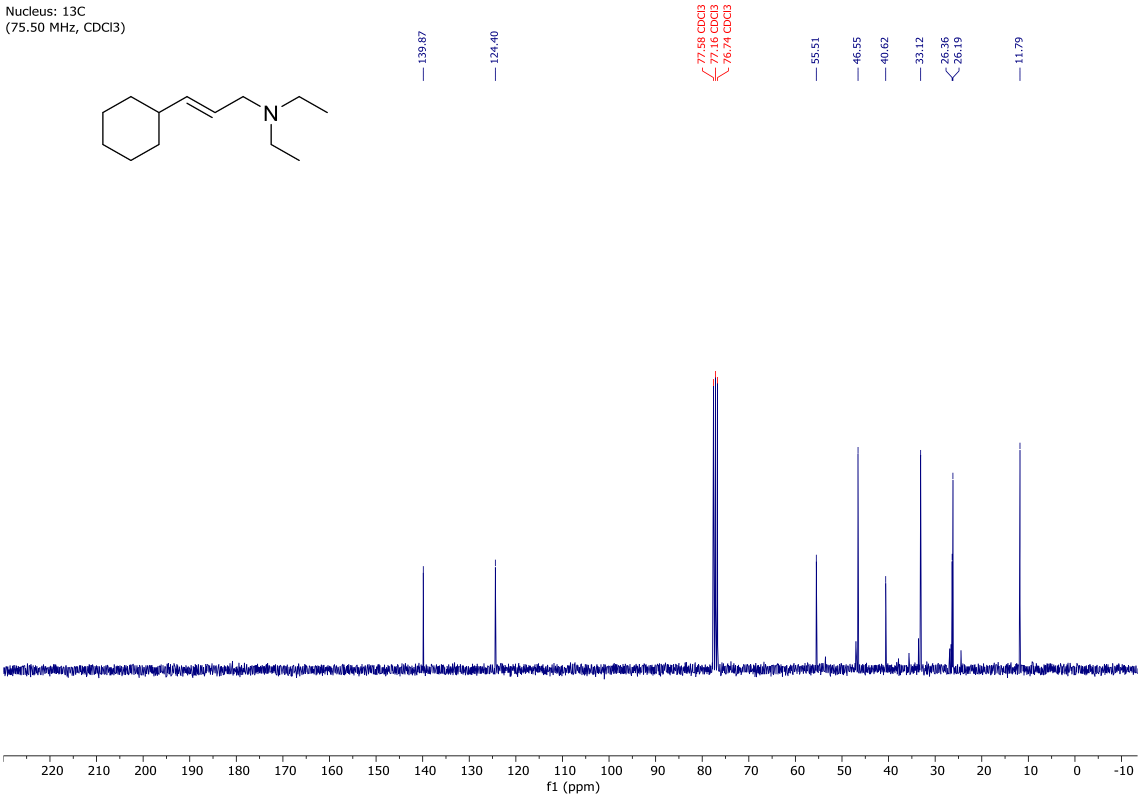

^{1}H NMR, ^{13}C NMR and ^{19}F NMR spectrum of compound **13a**

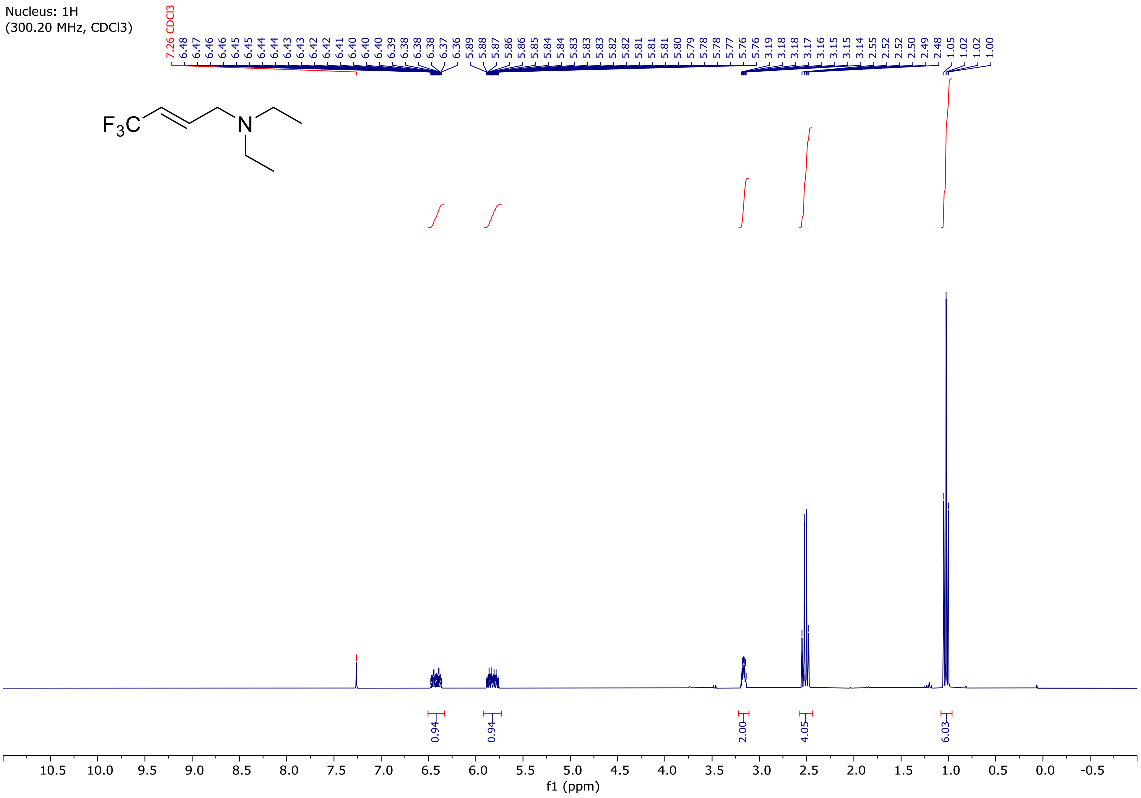

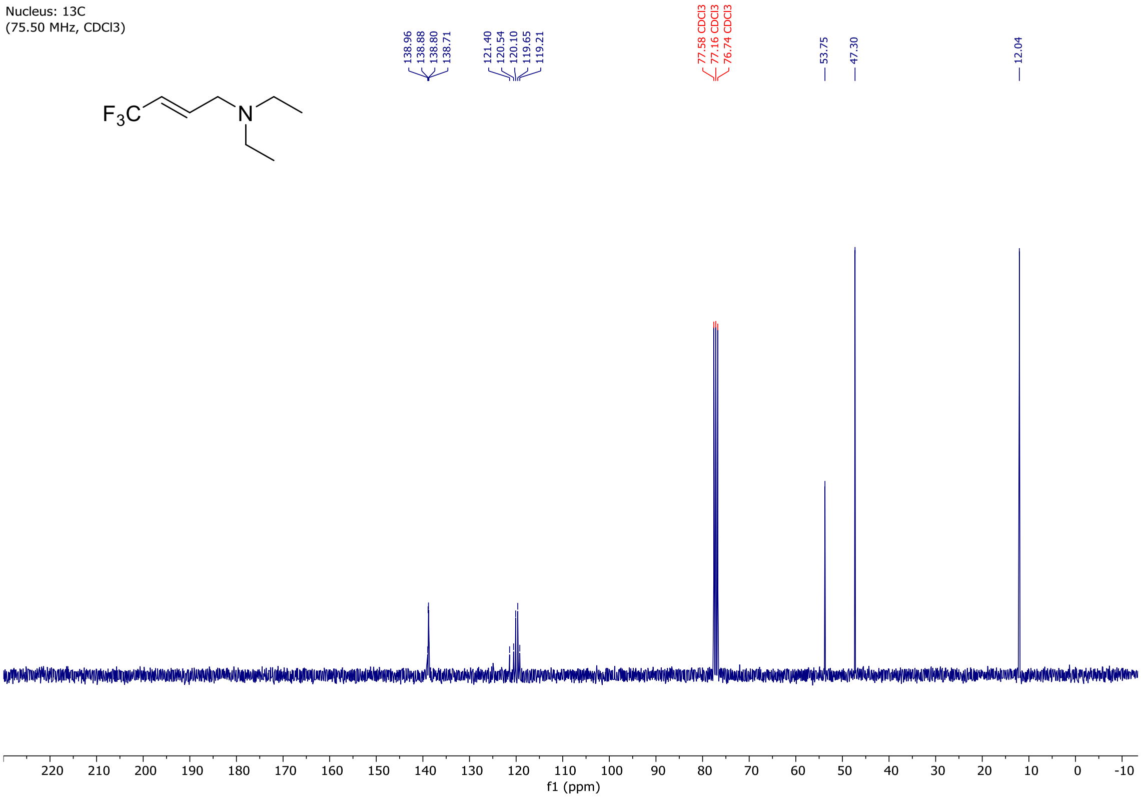

Nucleus: 19F
(282.46 MHz, CDCl3)

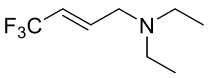

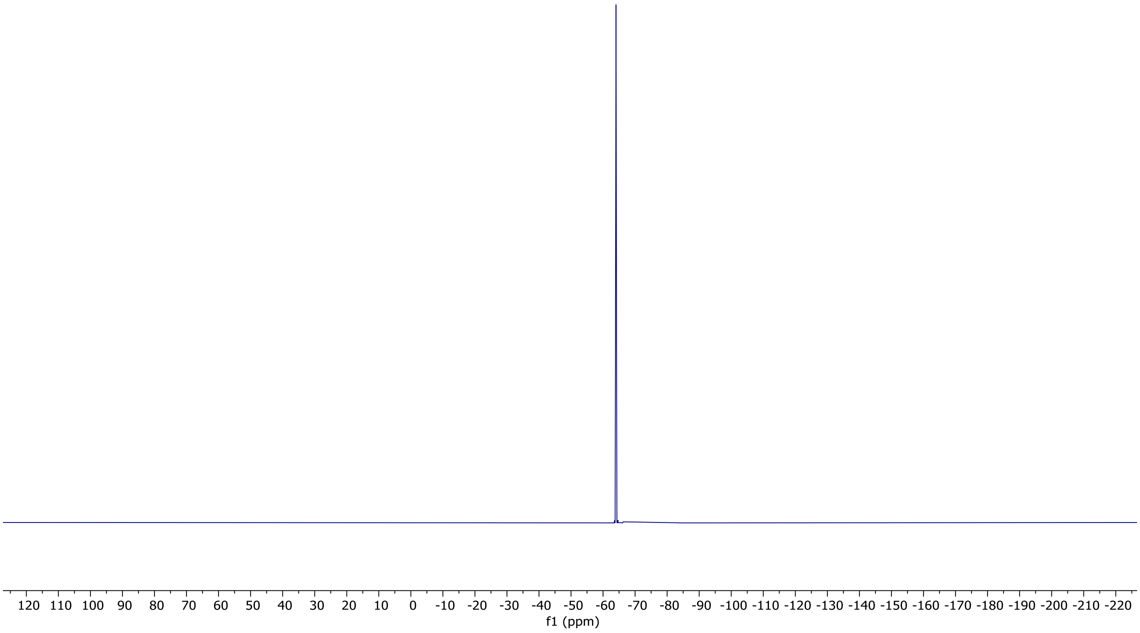

^{1}H NMR and ^{13}C NMR spectrum of compound **14a**

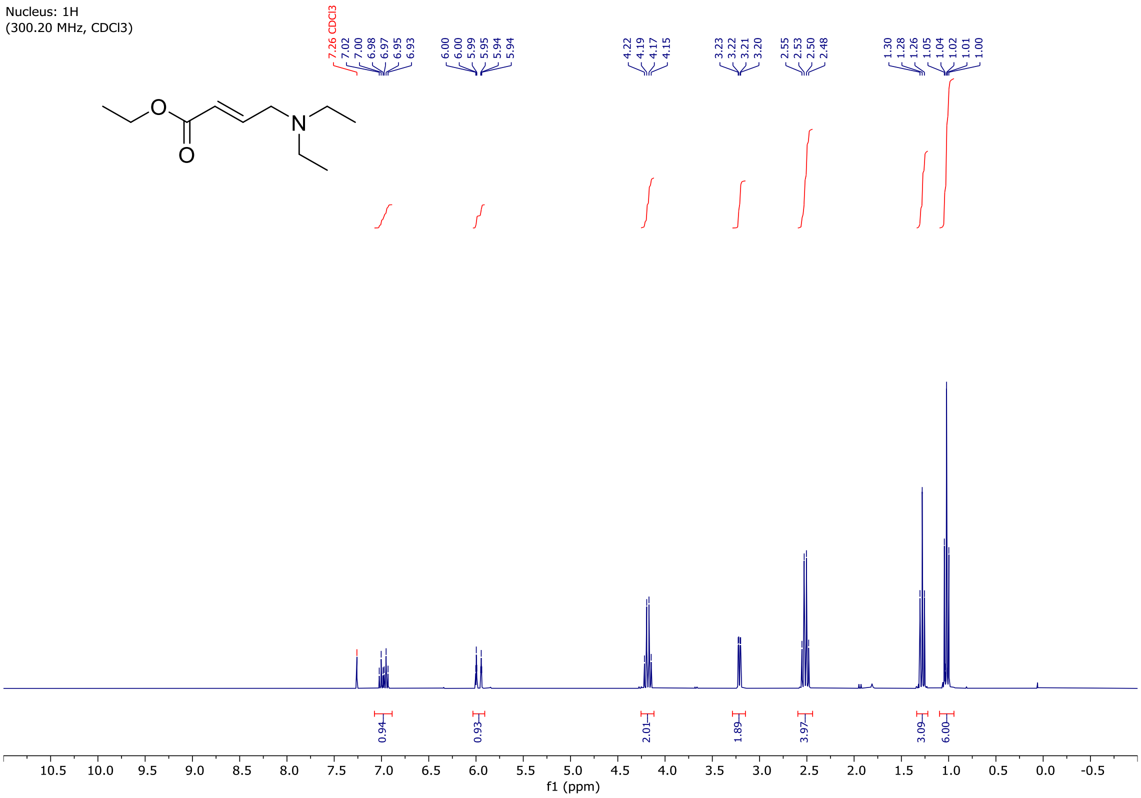

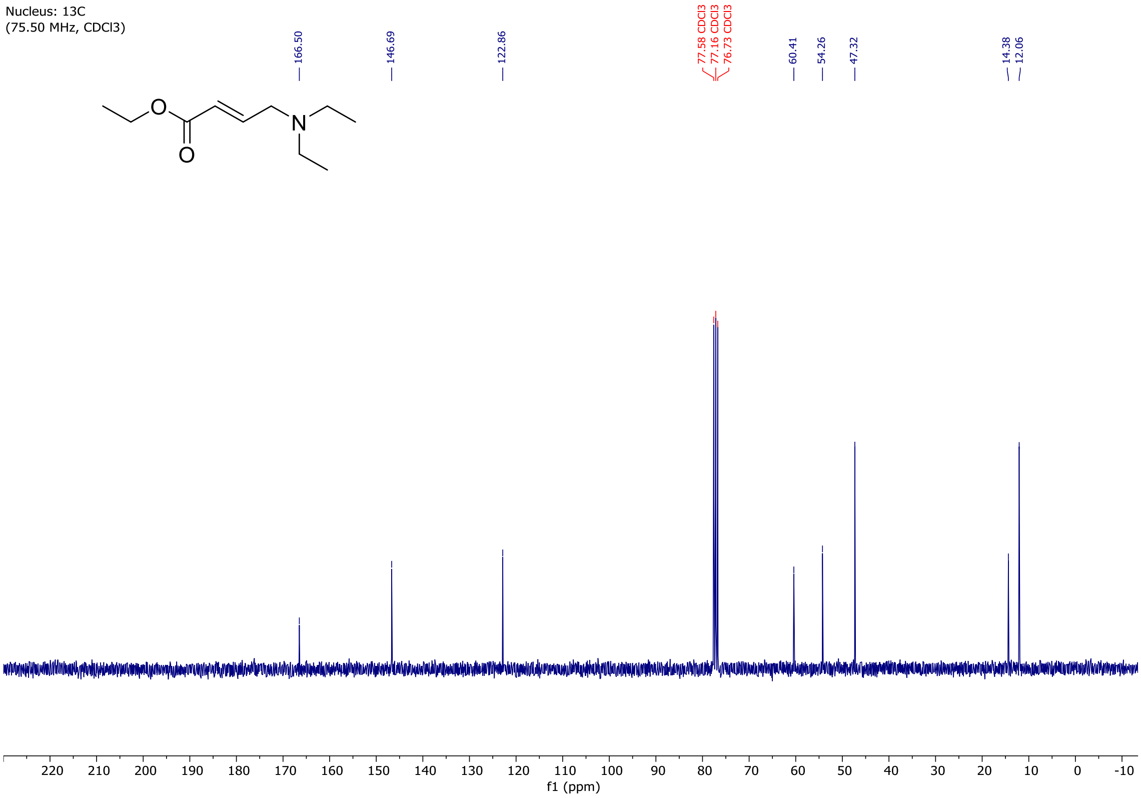

^{1}H NMR and ^{13}C NMR spectrum of compound **15a**

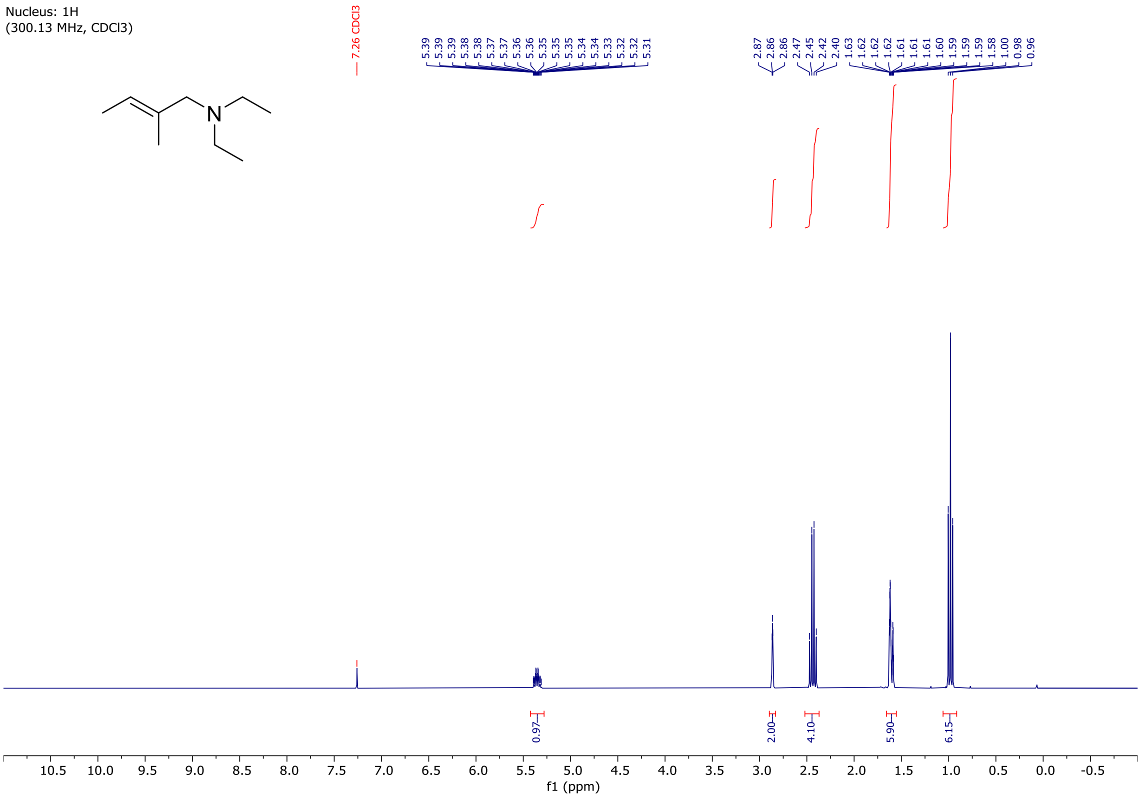

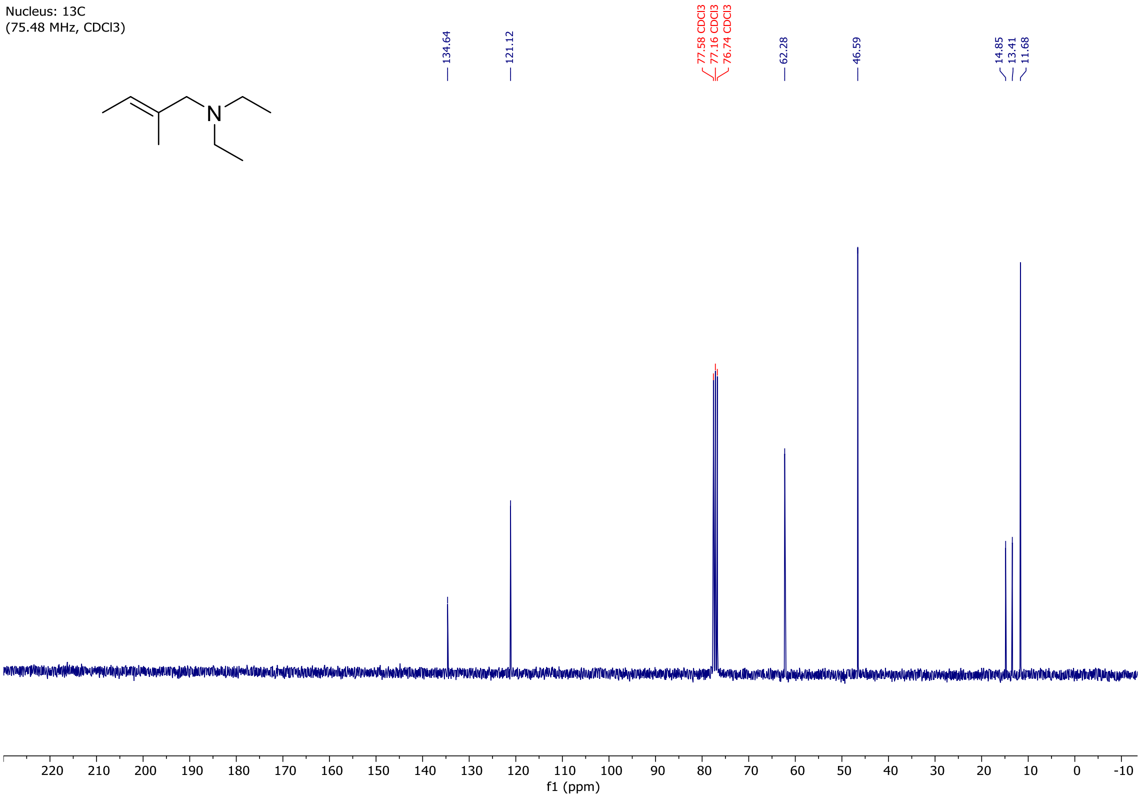

^{1}H NMR and ^{13}C NMR spectrum of compound **16a**

Nucleus: 1H
(300.20 MHz, CDCl3)

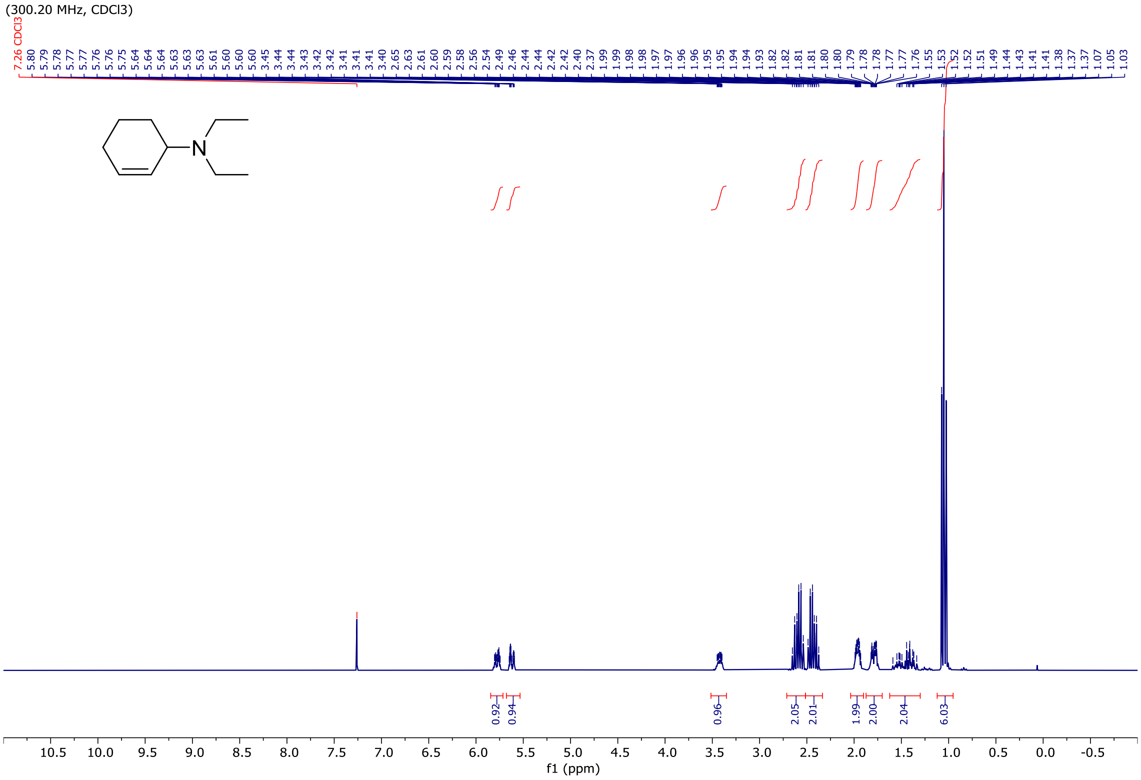

Nucleus: 13C
(75.50 MHz, CDCl3)

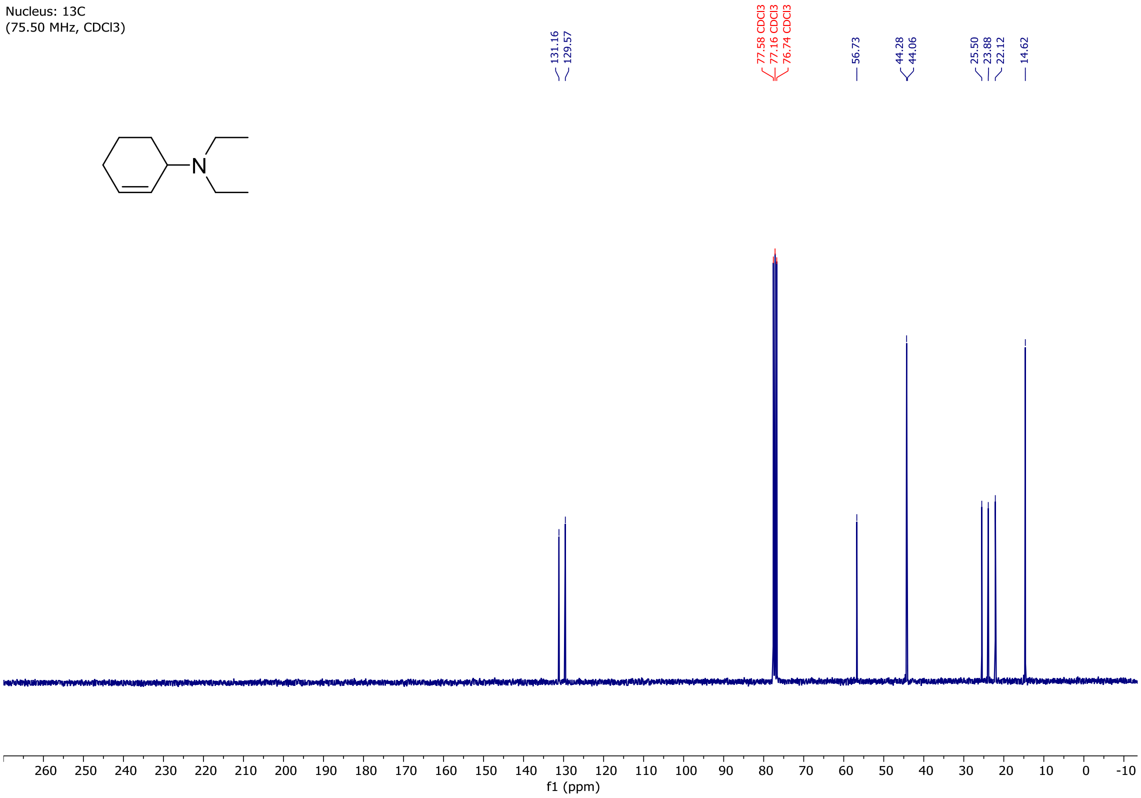

^{1}H NMR and ^{13}C NMR spectrum of compound **17a**

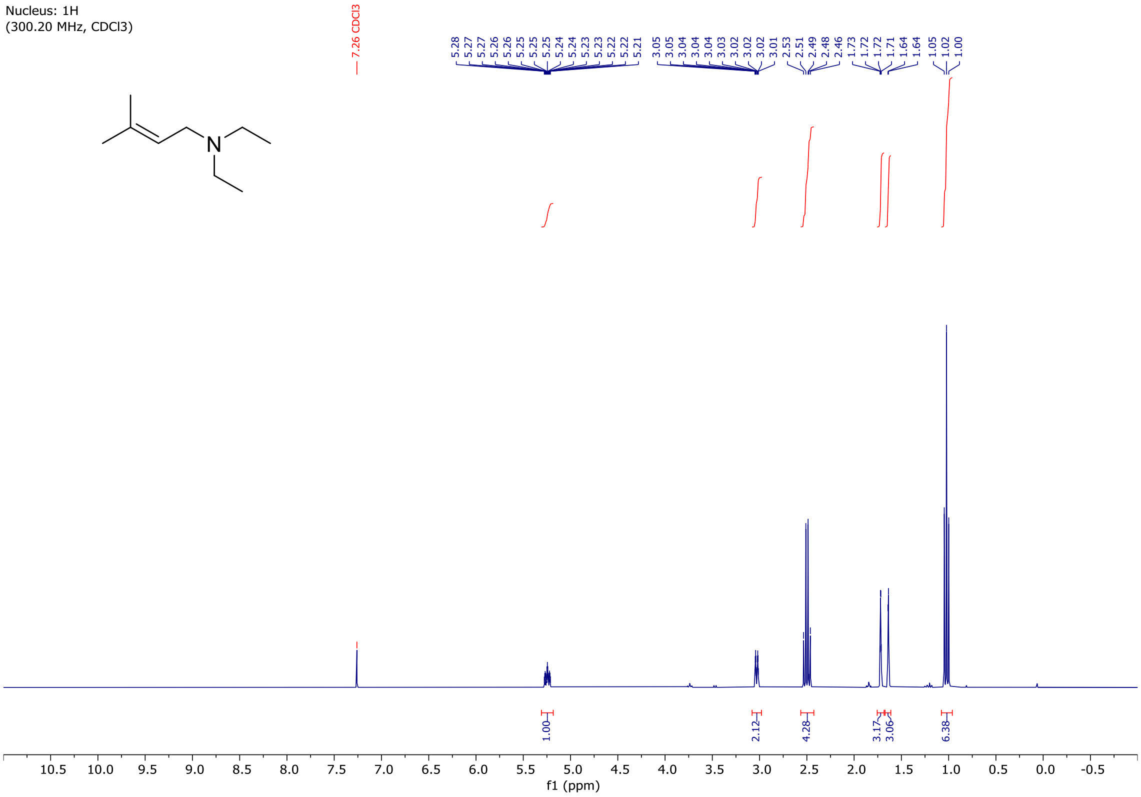

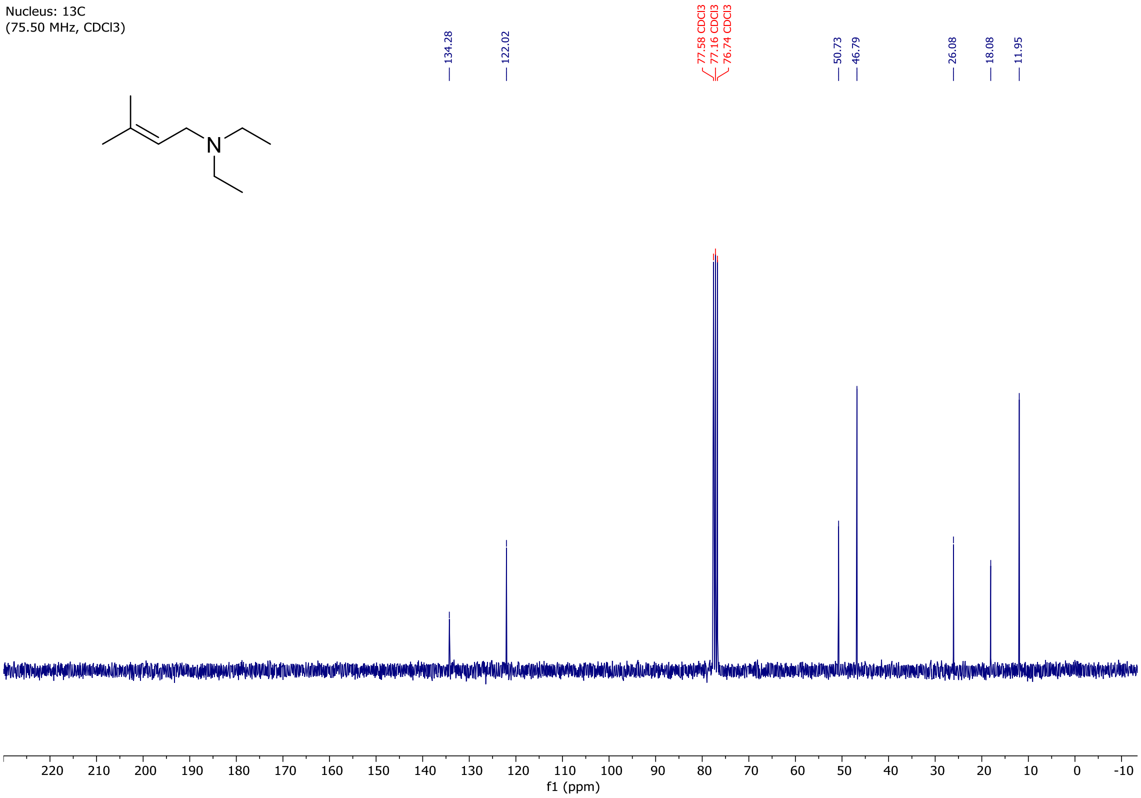

^{1}H NMR and ^{13}C NMR spectrum of compound **18a**

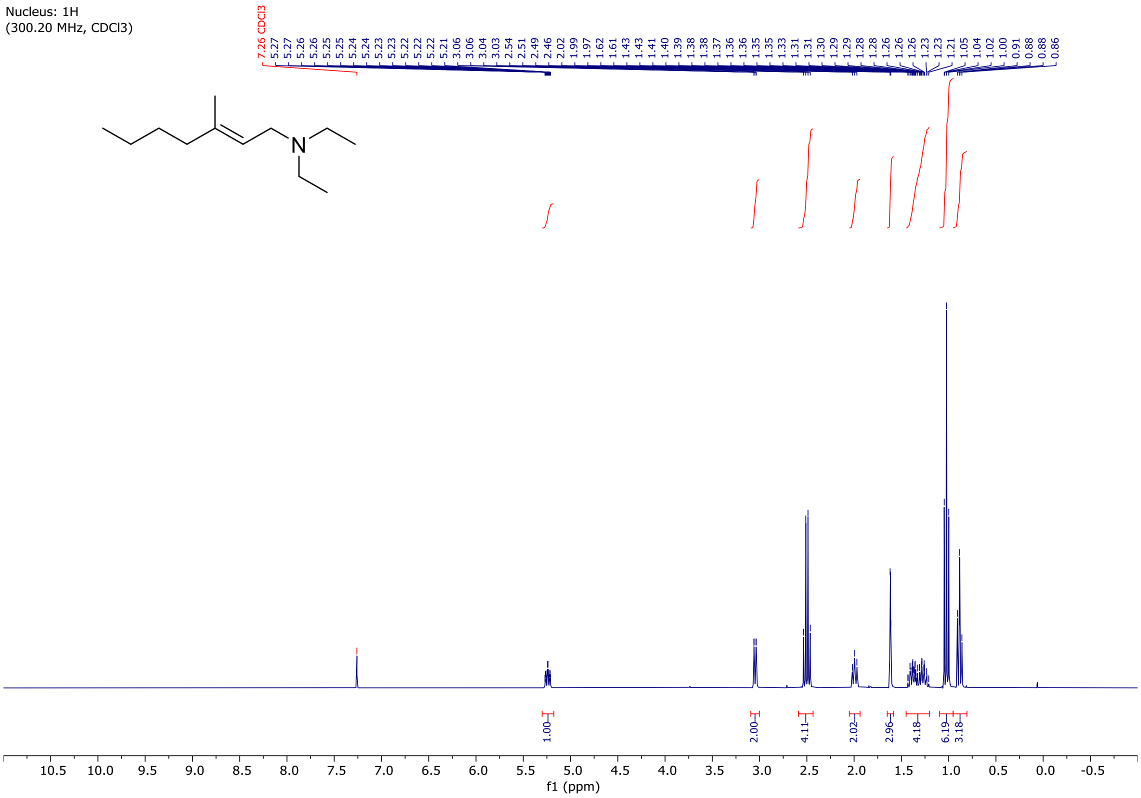

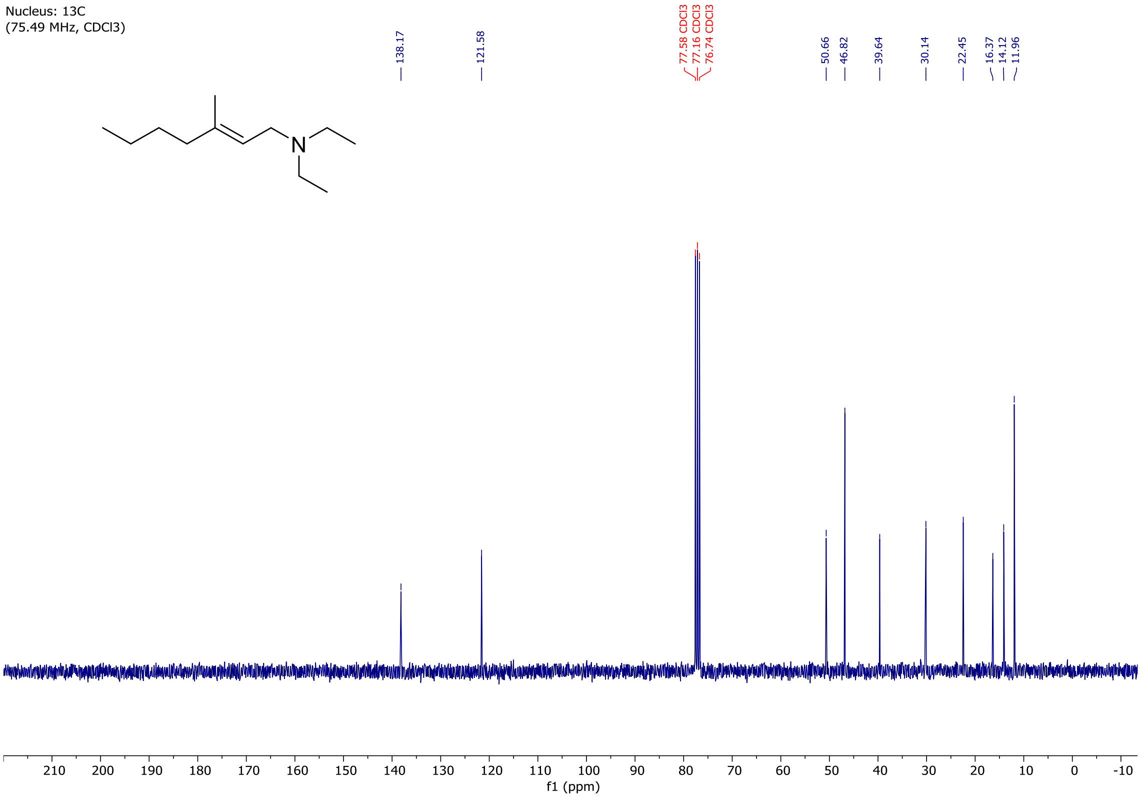

^{1}H NMR and ^{13}C NMR spectrum of compound **19a**

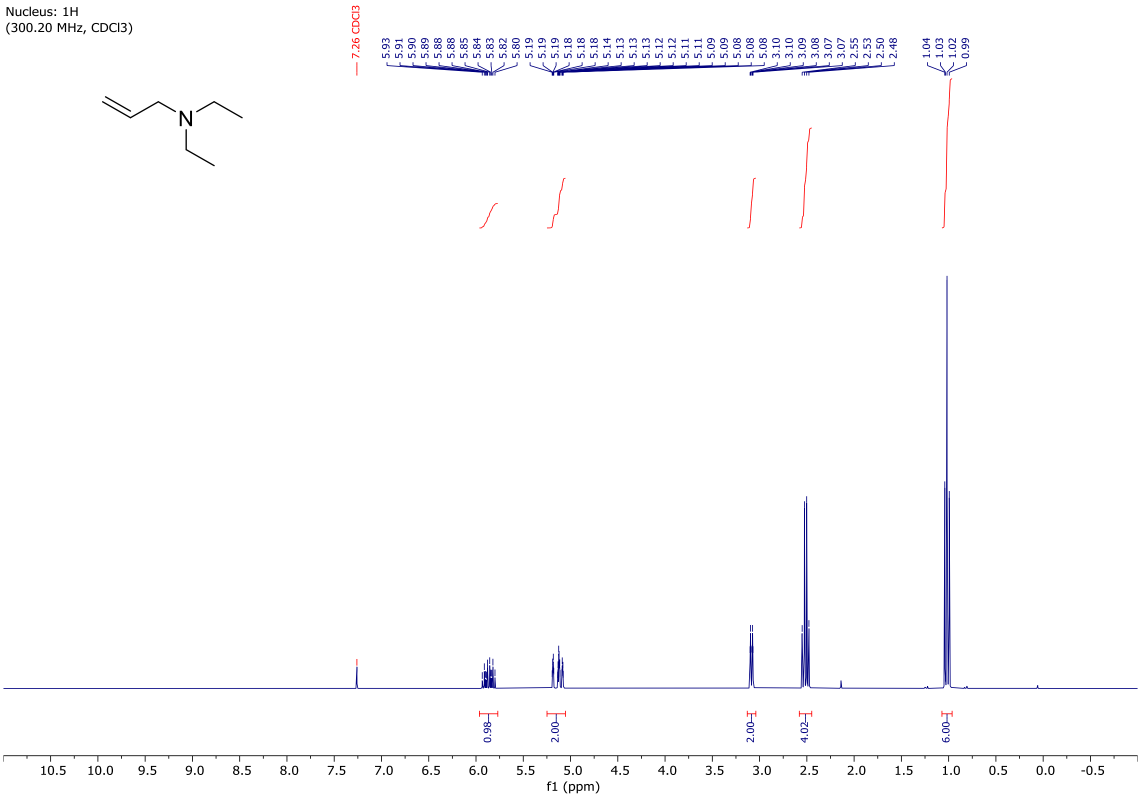

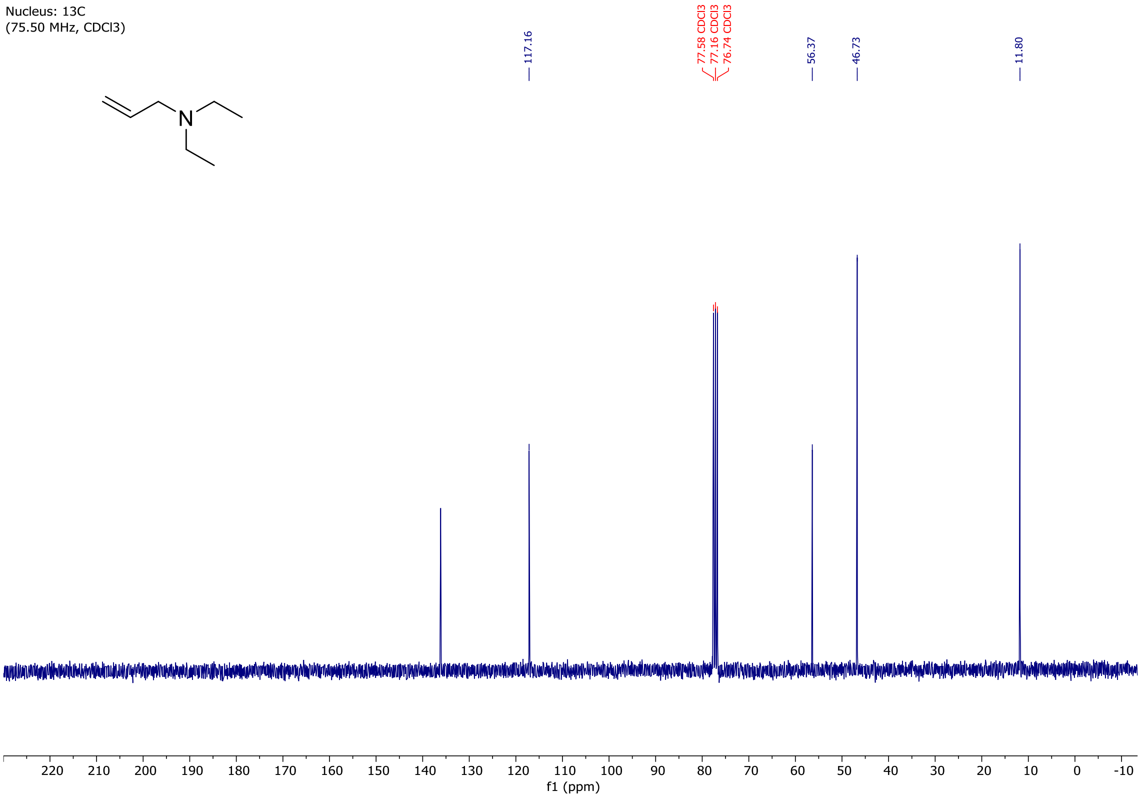

^{1}H NMR and ^{13}C NMR spectrum of compound **20a**

Nucleus: 1H
(300.20 MHz, CDCl3)

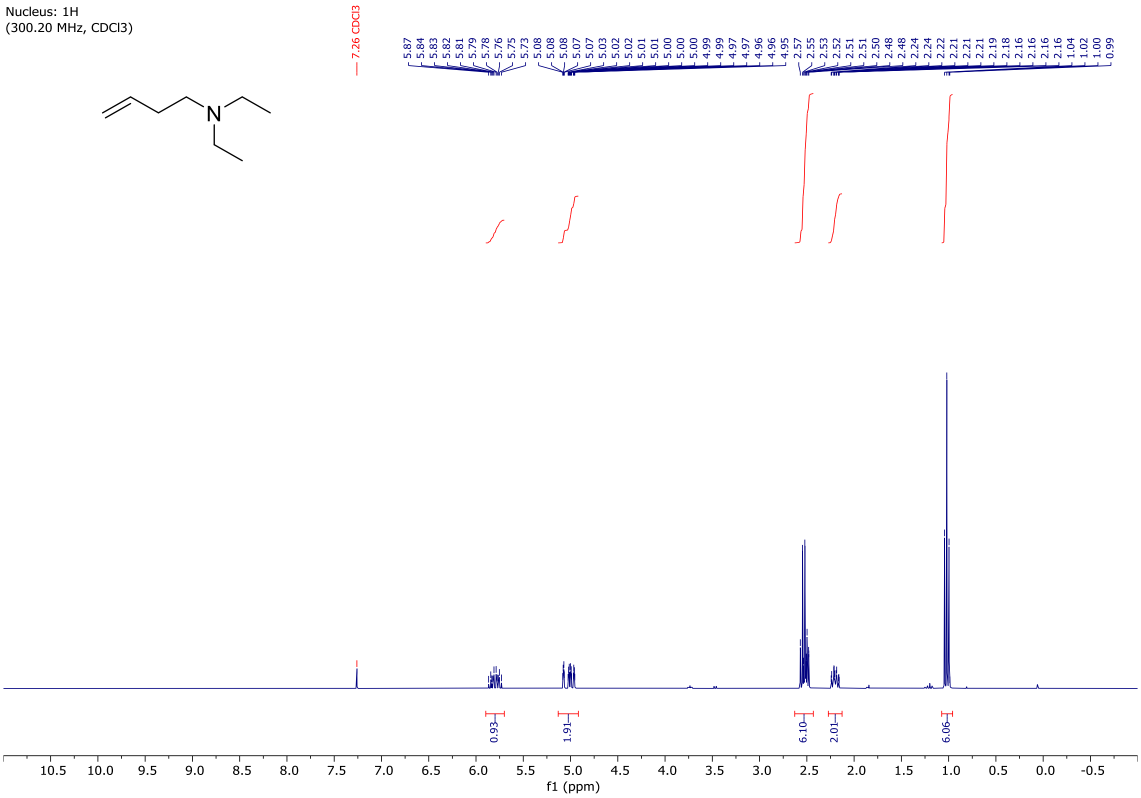

Nucleus: 13C
(75.50 MHz, CDCl3)

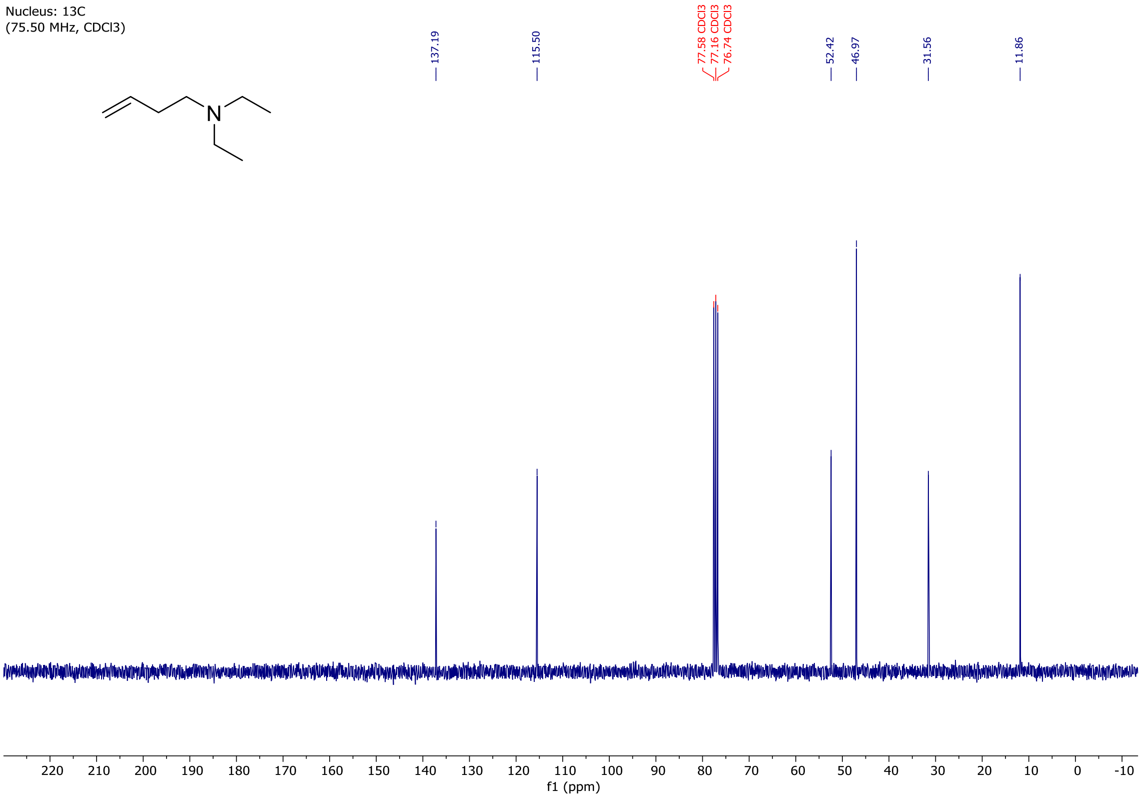

^{1}H NMR and ^{13}C NMR spectrum of compound **21a**

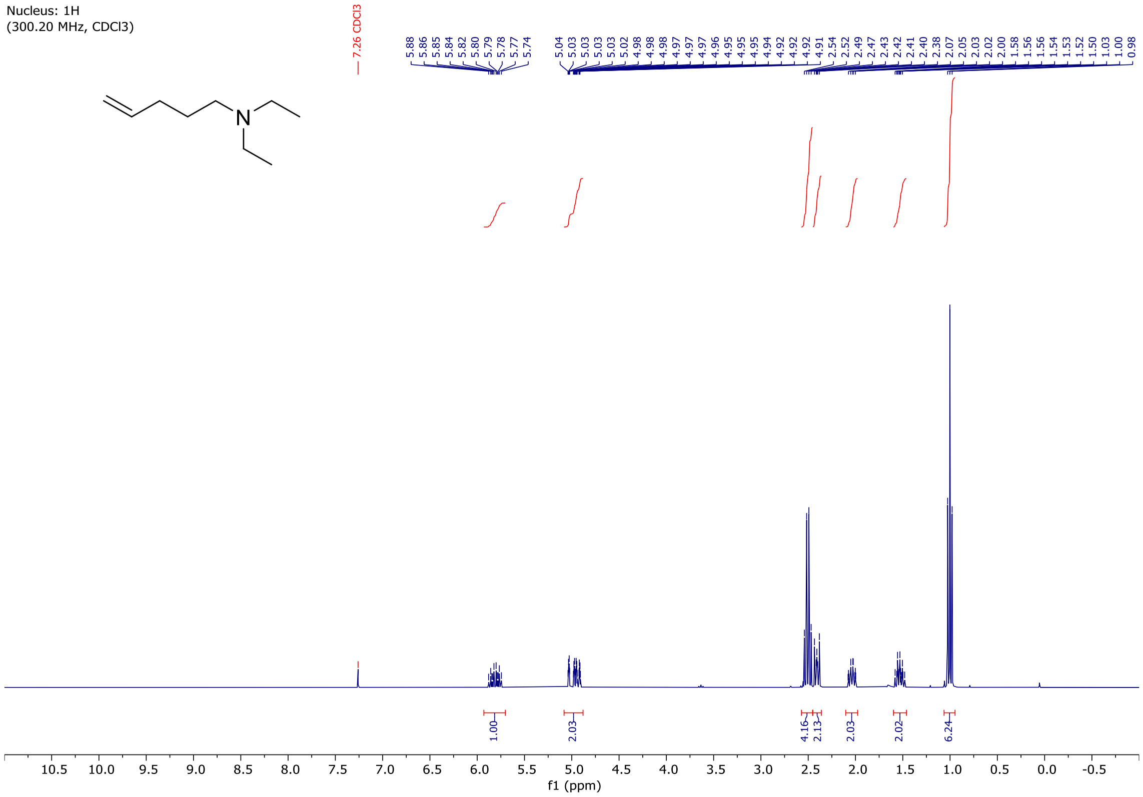

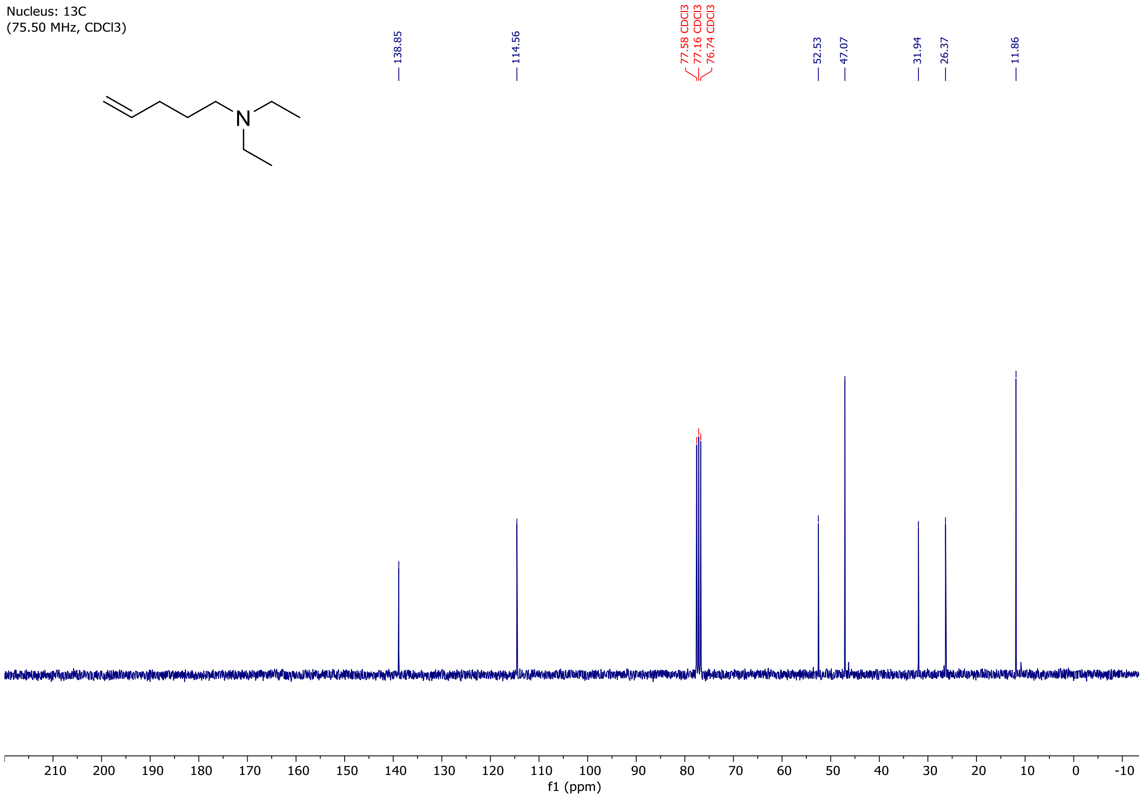

^{1}H NMR and ^{13}C NMR spectrum of compound **22a**

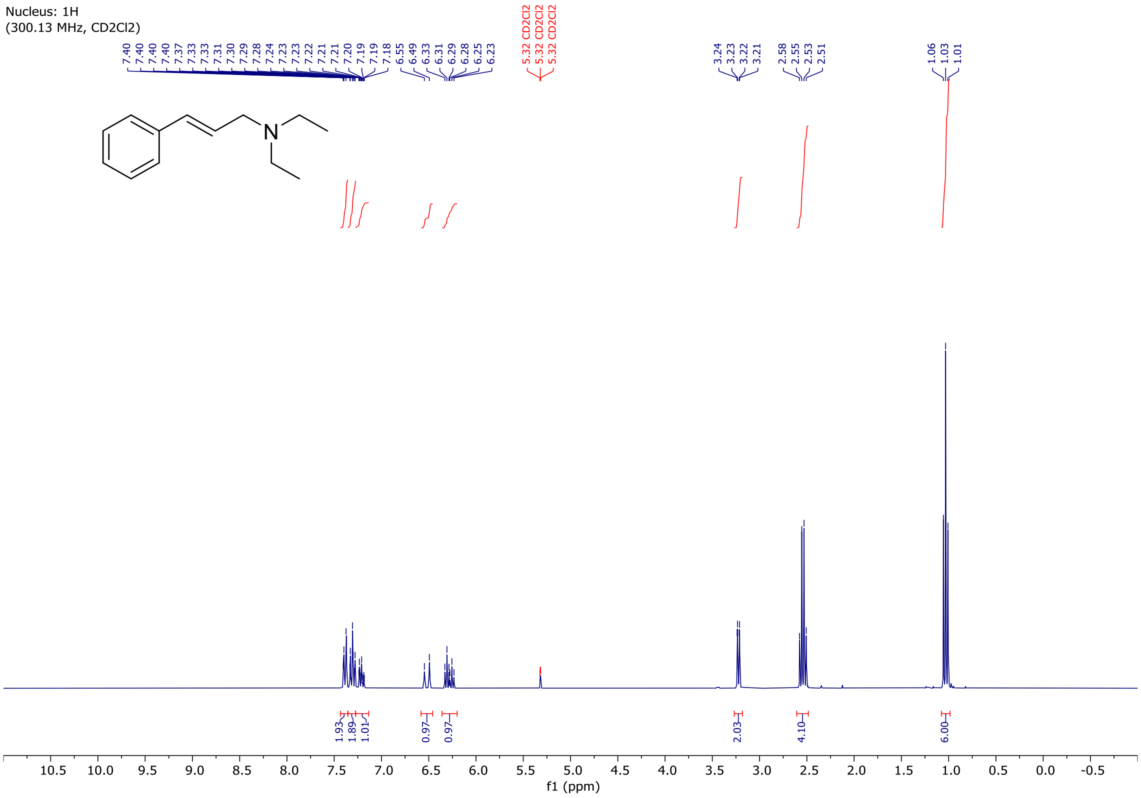

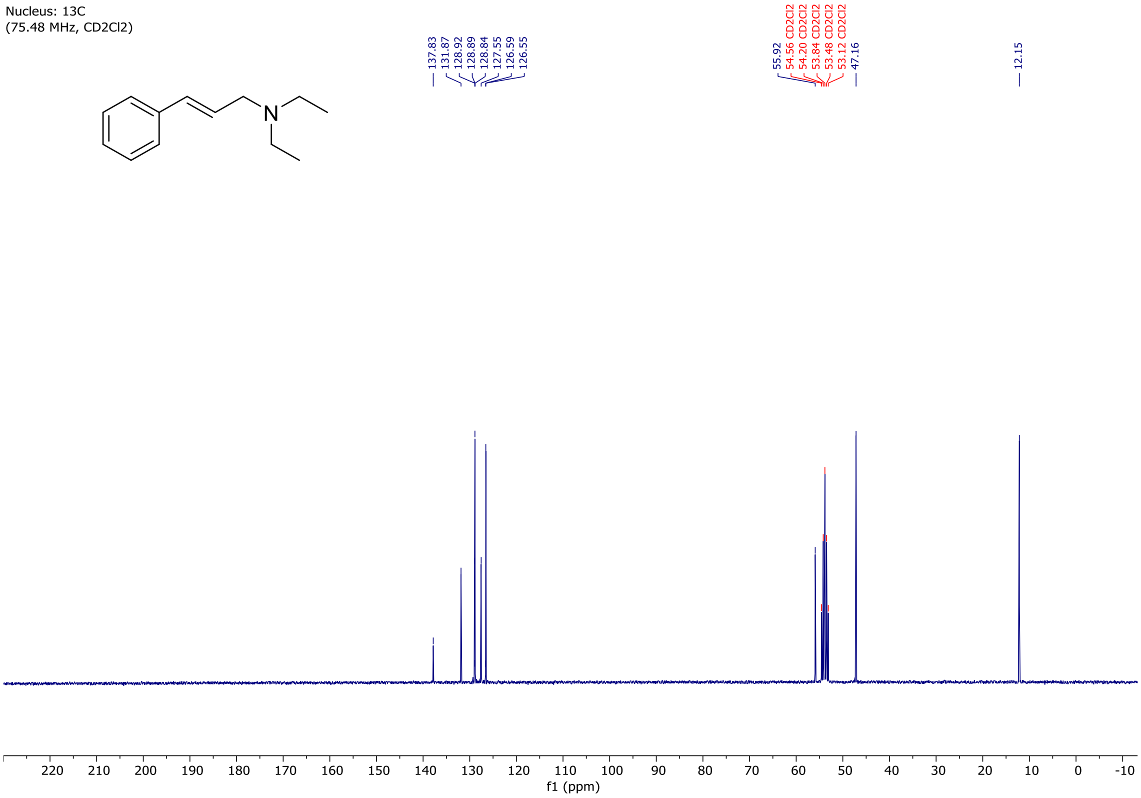

^{1}H NMR and ^{13}C NMR spectrum of compound **23a**

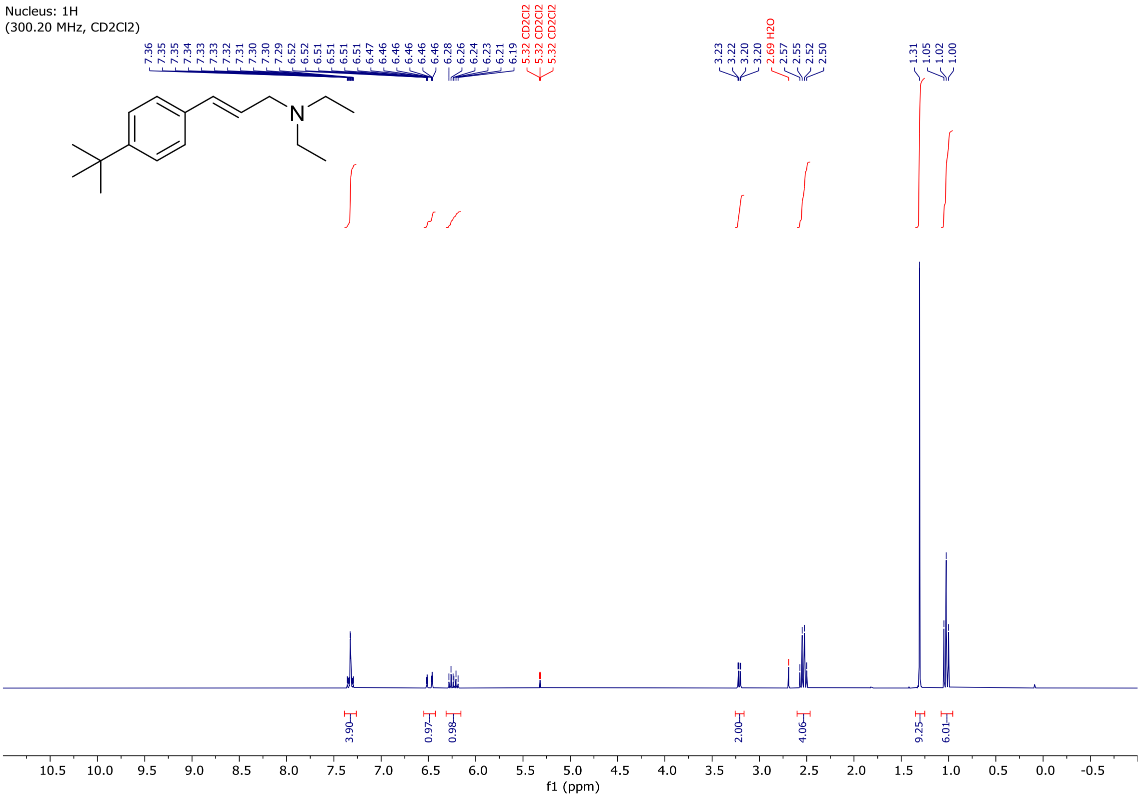

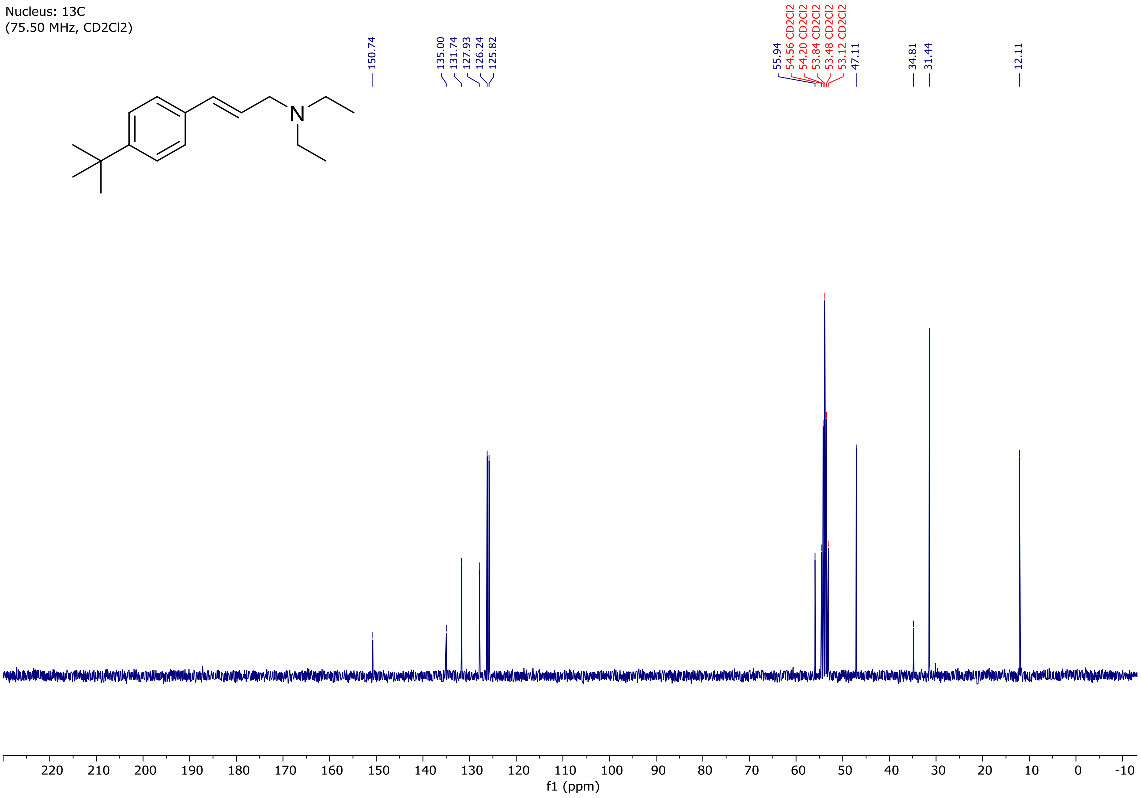

^{1}H NMR and ^{13}C NMR spectrum of compound **24a**

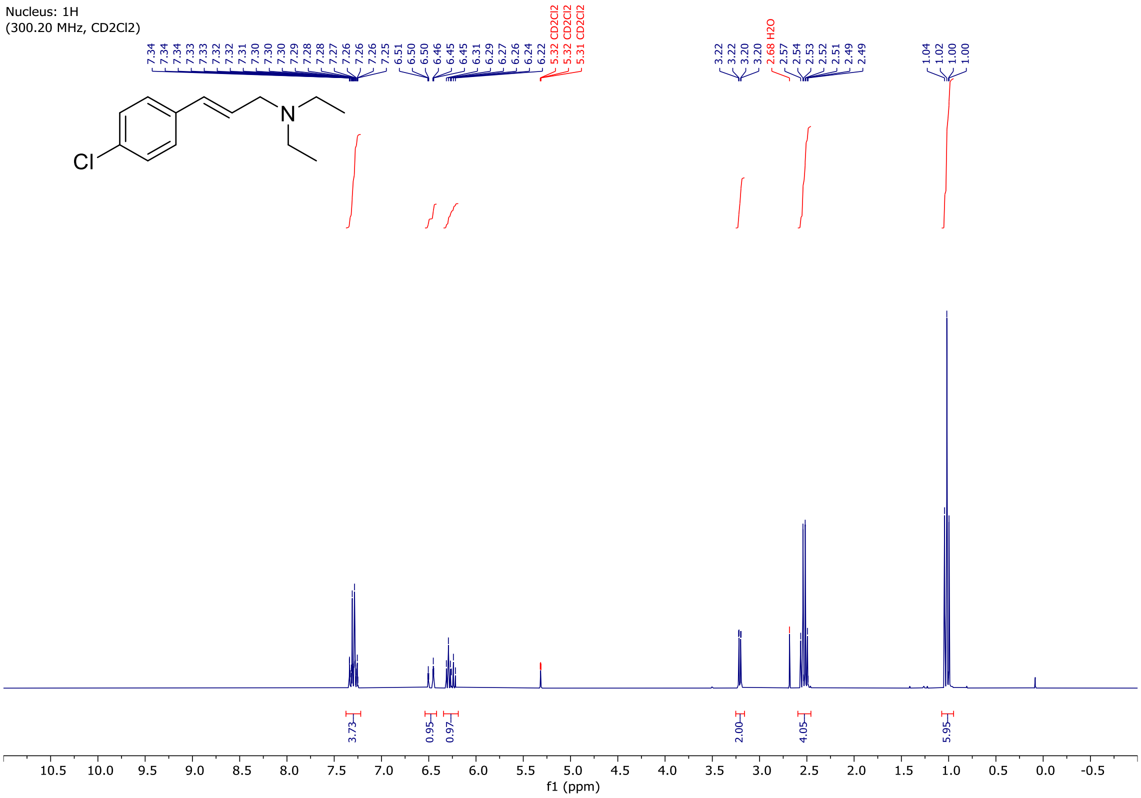

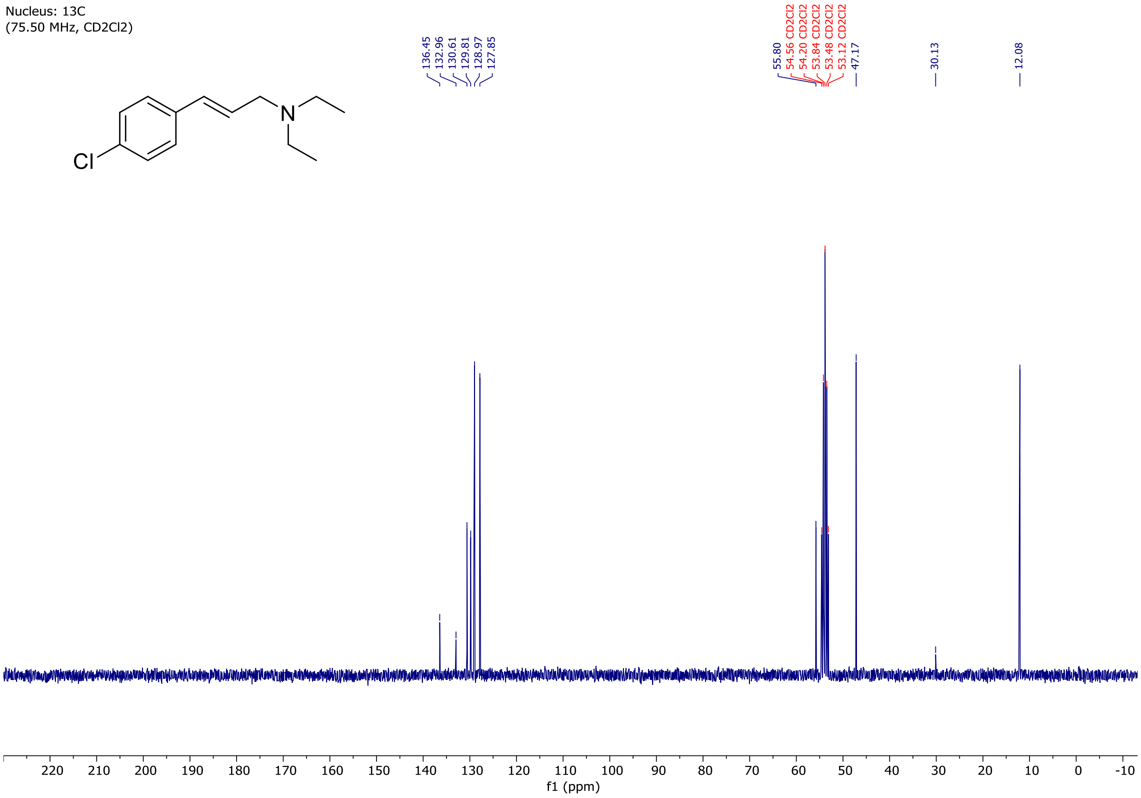

^{1}H NMR, ^{13}C NMR and ^{19}F NMR spectrum of compound **25a**

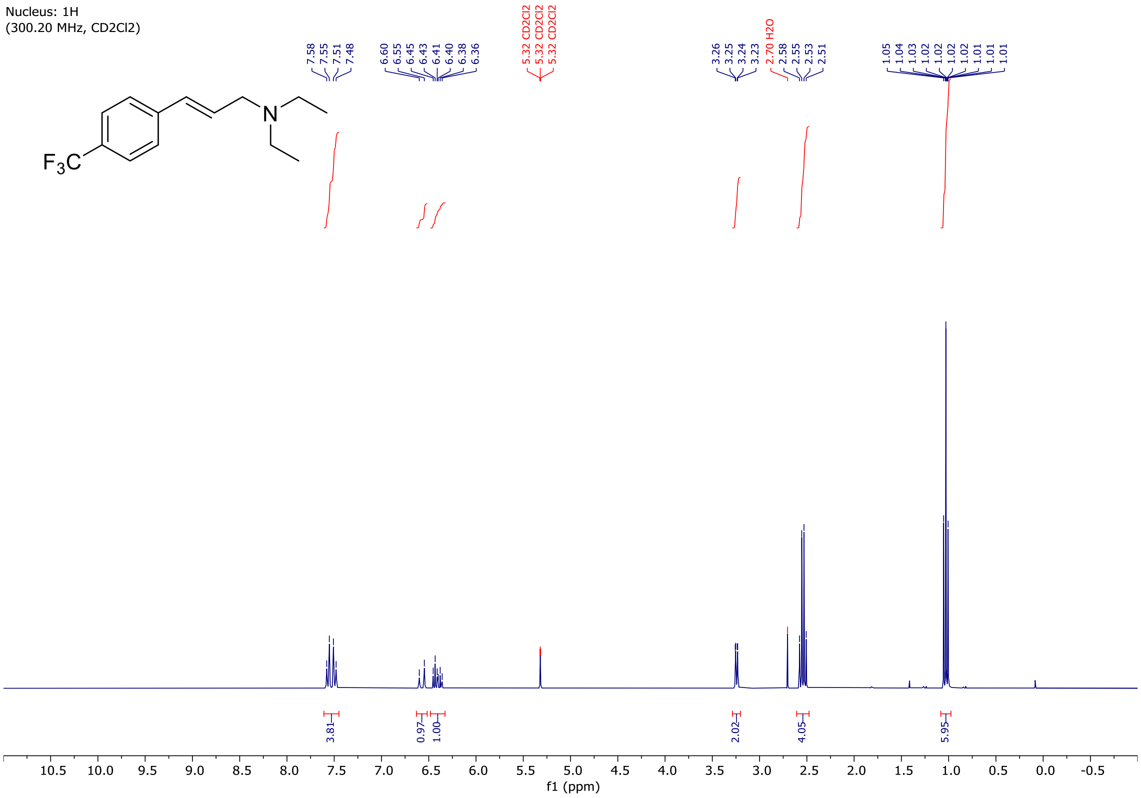

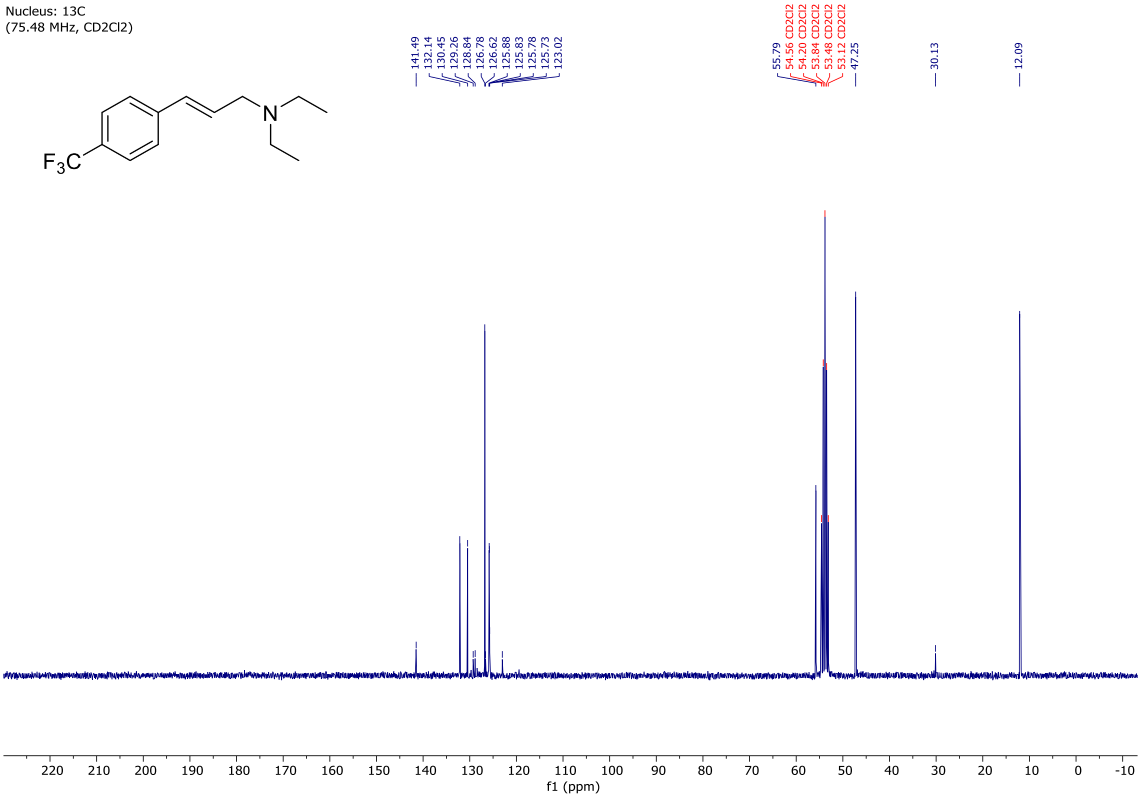

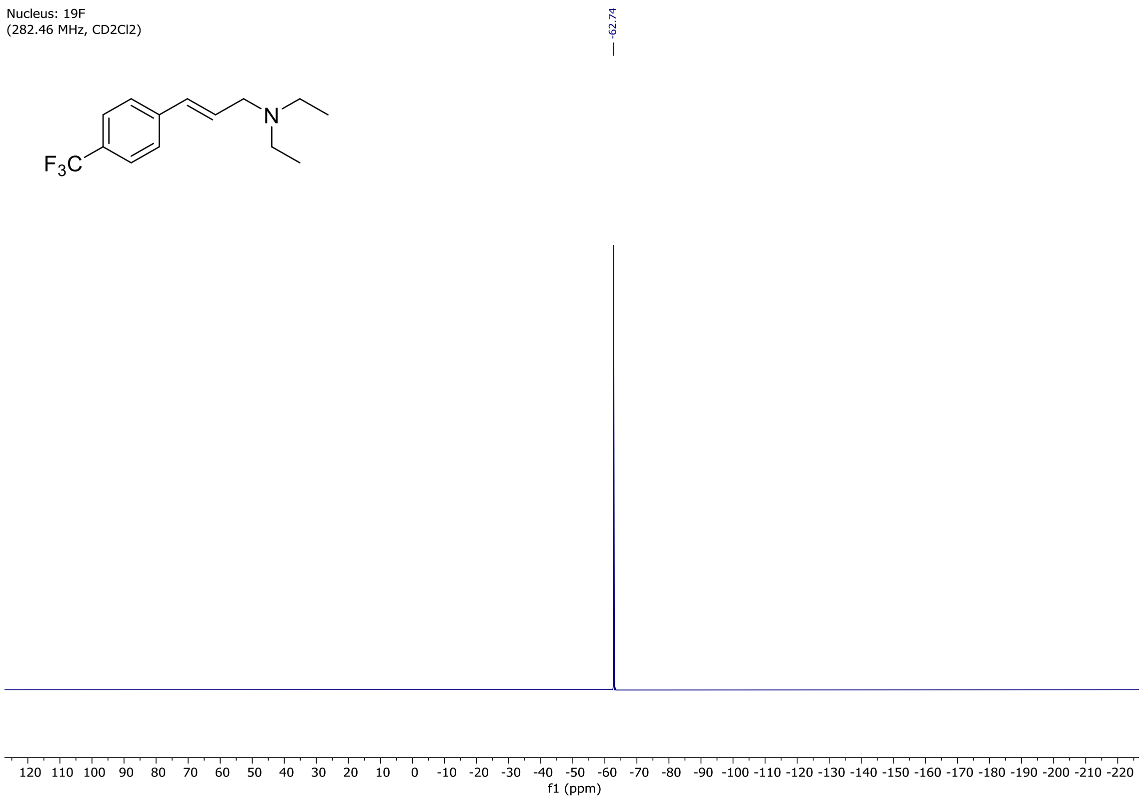
Nucleus: 19F
(282.46 MHz, CD2Cl2)
-62.74
F3C
N
120 110 100 90 80 70 60 50 40 30 20 10 0 -10 -20 -30 -40 -50 -60 -70 -80 -90 -100 -110 -120 -130 -140 -150 -160 -170 -180 -190 -200 -210 -220
f1 (ppm)

^{1}H NMR and ^{13}C NMR spectrum of compound **26a**

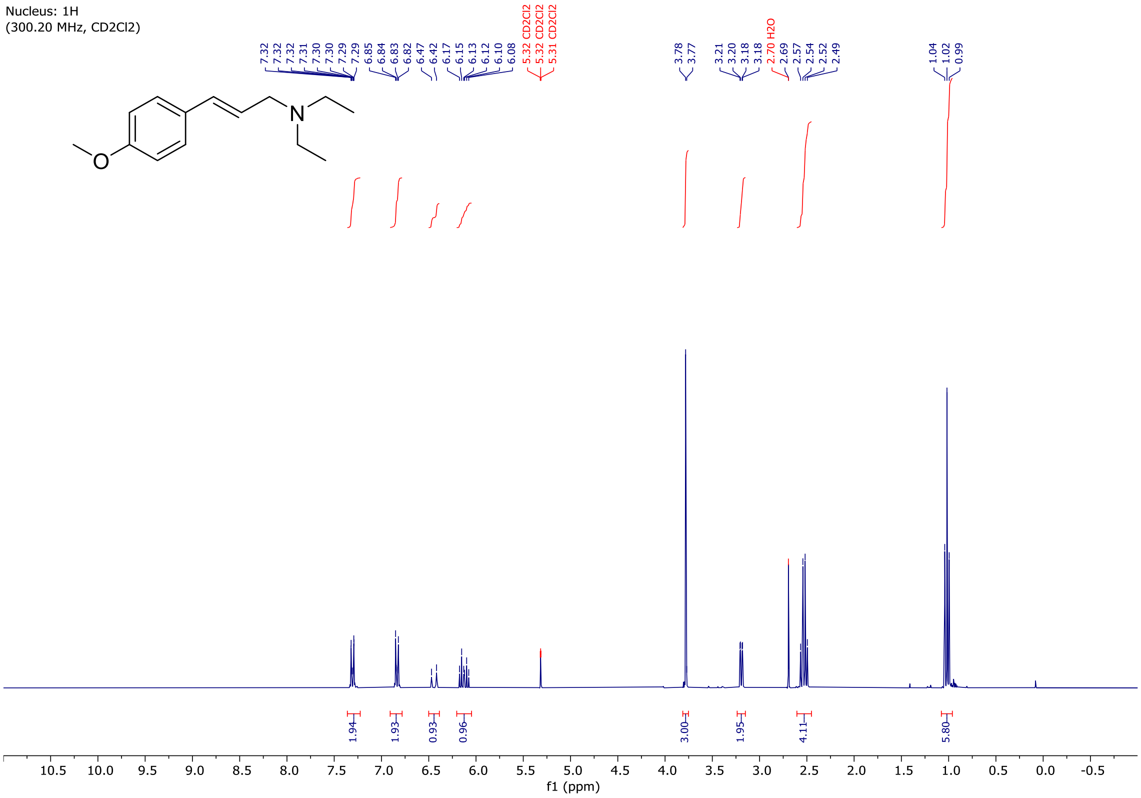

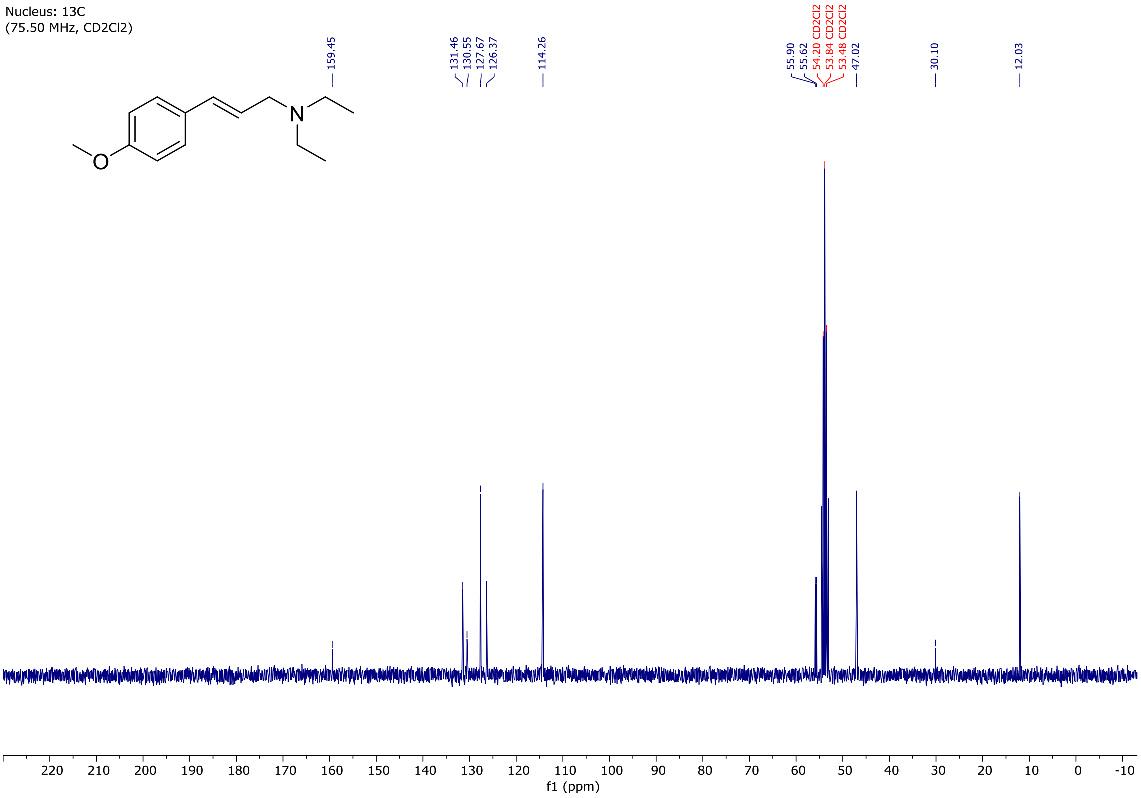

^{1}H NMR and ^{13}C NMR spectrum of compound **27a**

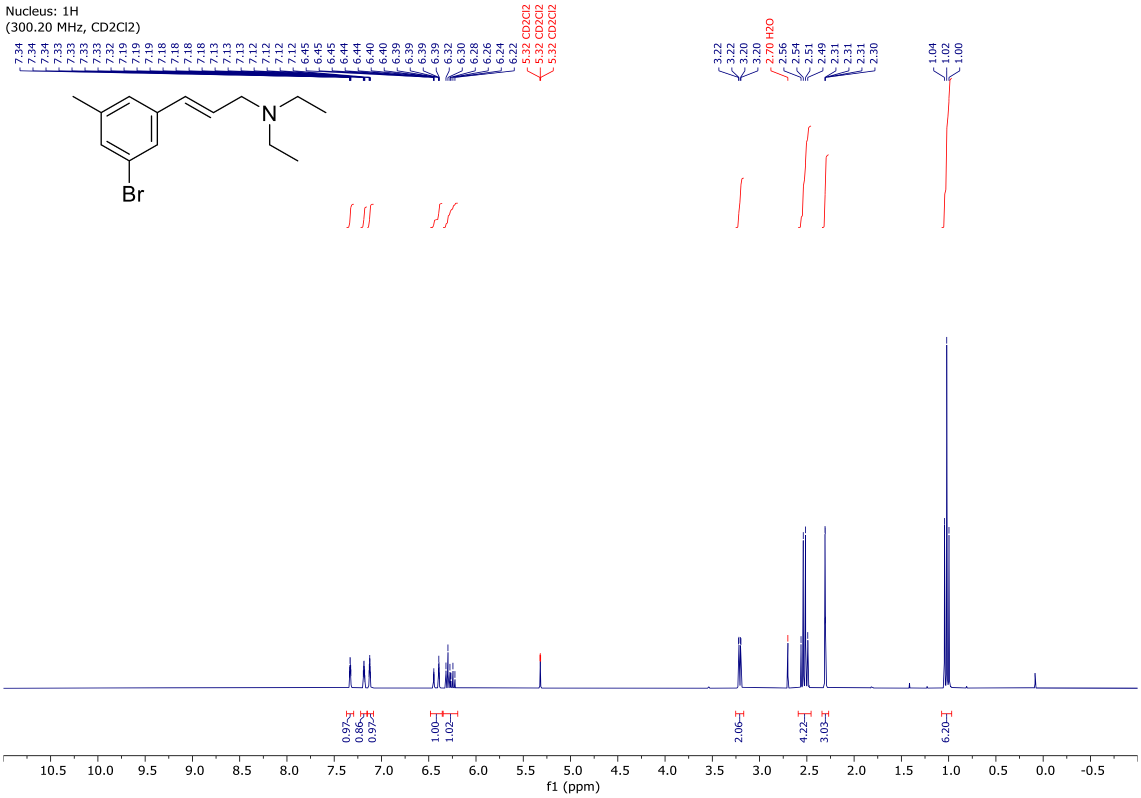

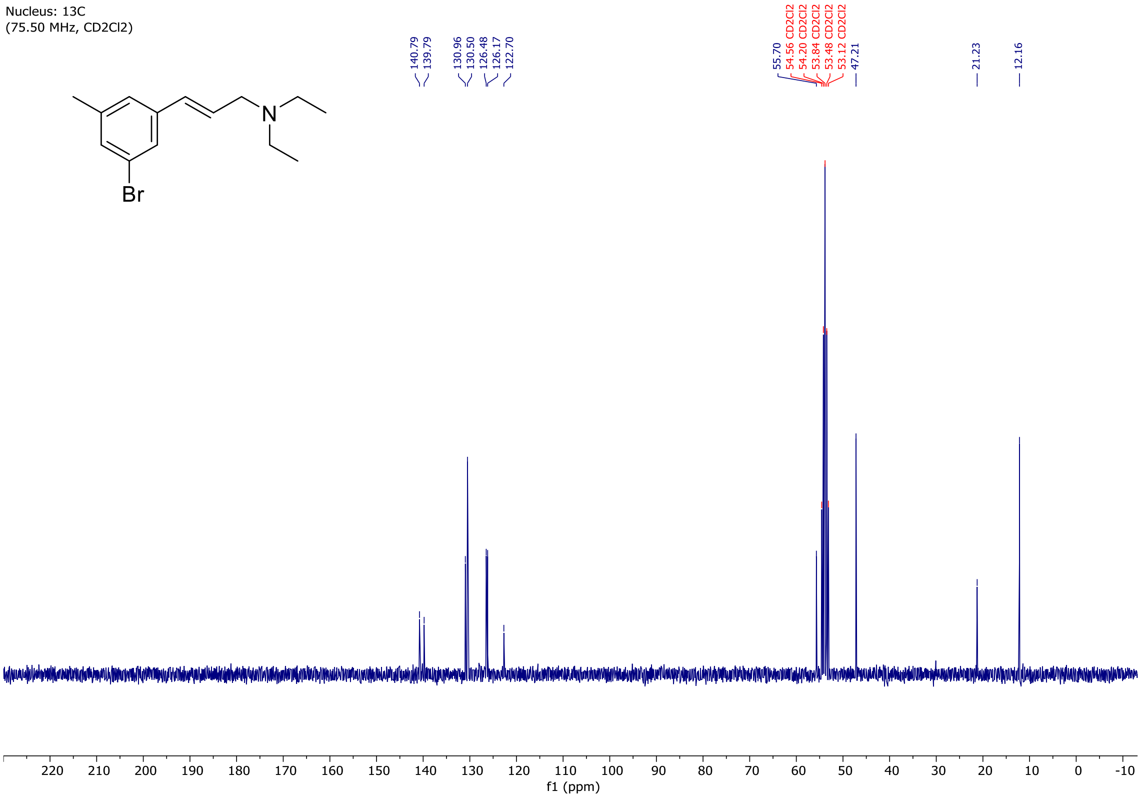

^{1}H NMR and ^{13}C NMR spectrum of compound **28a**

Nucleus: 1H
(300.20 MHz, CD2Cl2)

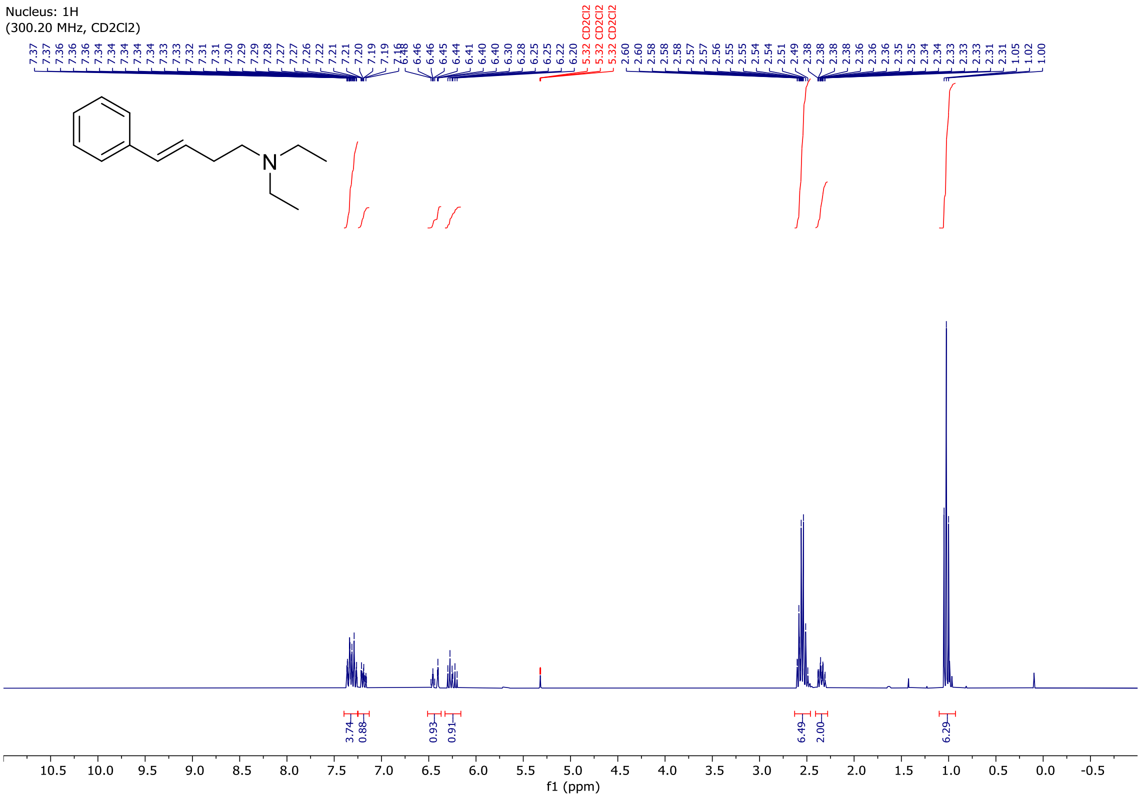

Nucleus: 13C
(75.50 MHz, CD2Cl2)

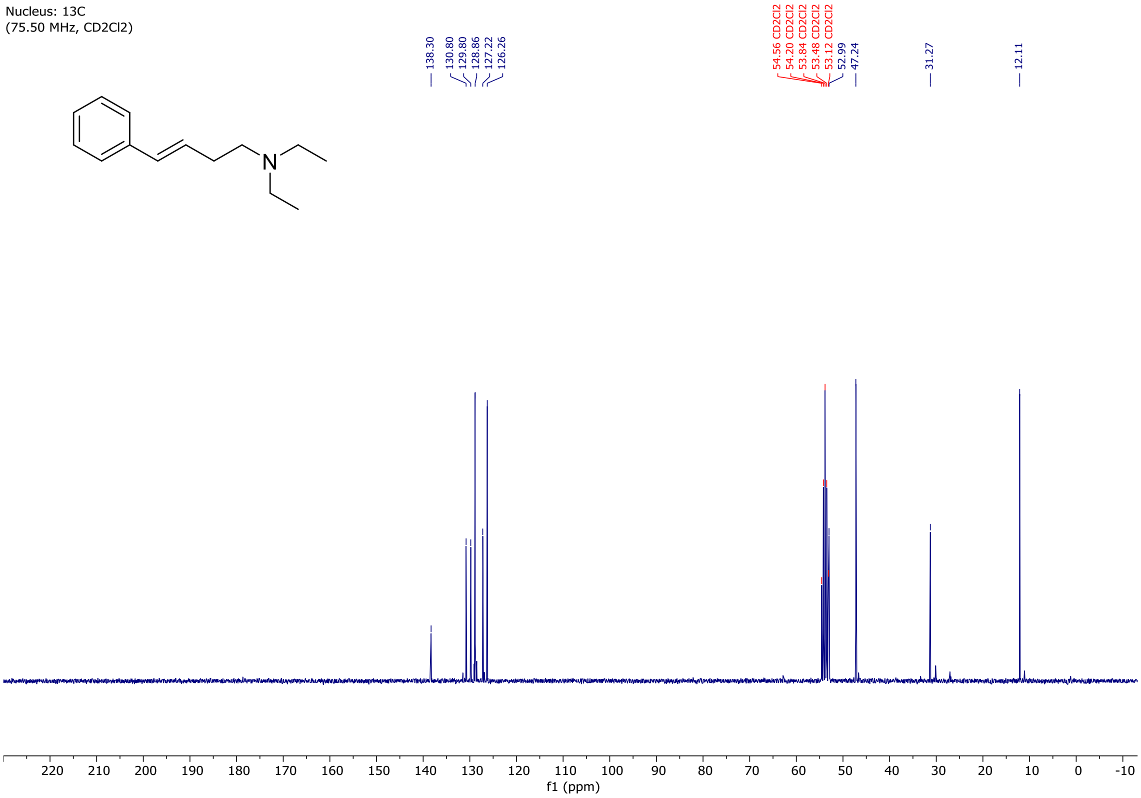

^{1}H NMR and ^{13}C NMR spectrum of compound **29a**

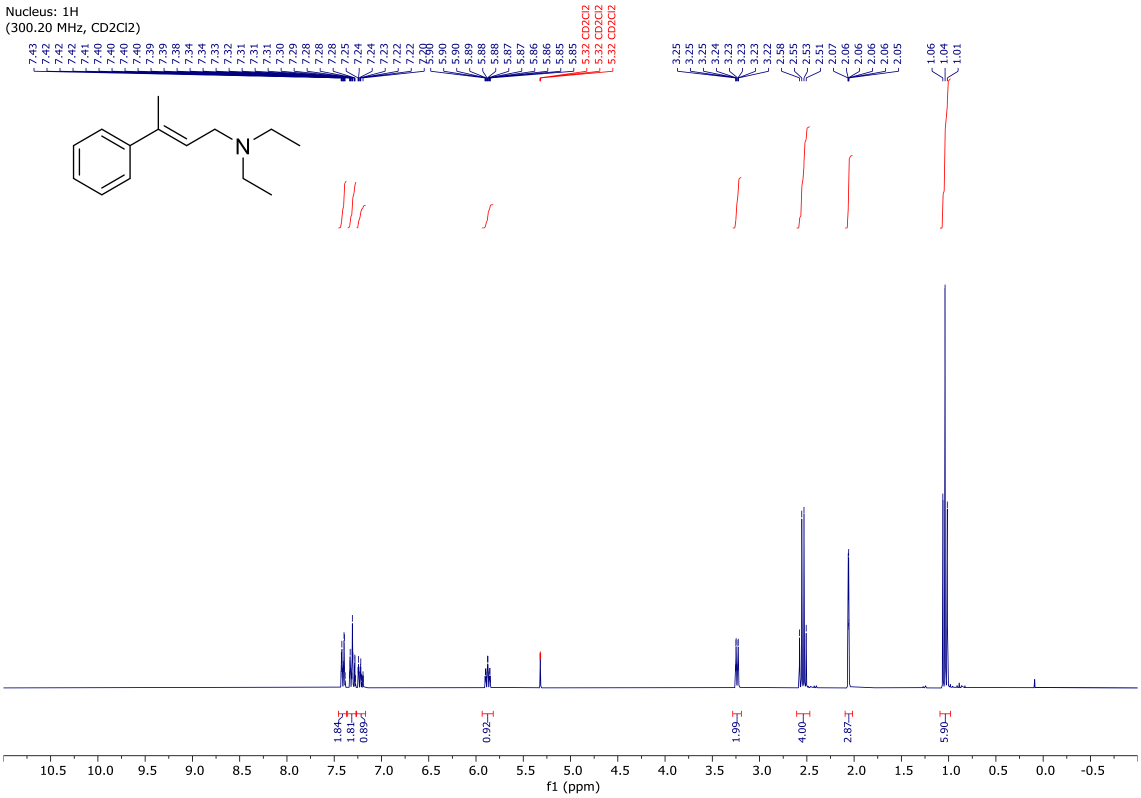

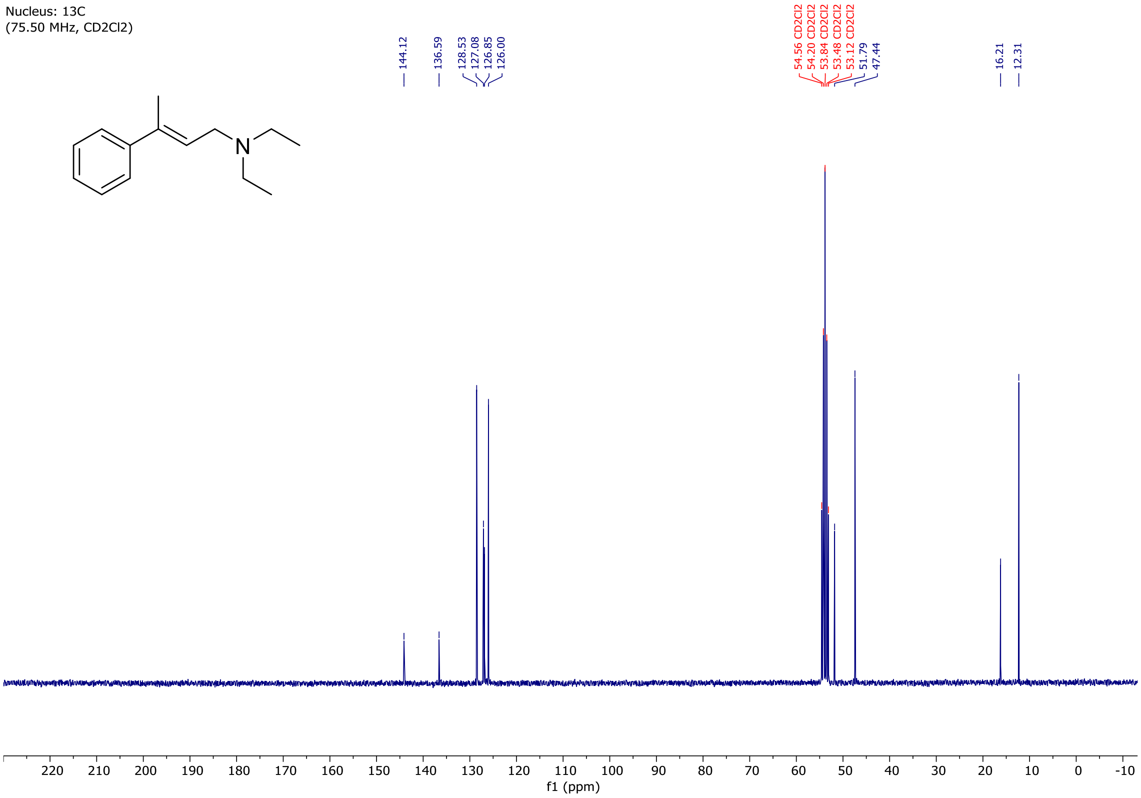

^{1}H NMR, ^{13}C NMR and ^{19}F spectrum of compound **30a**

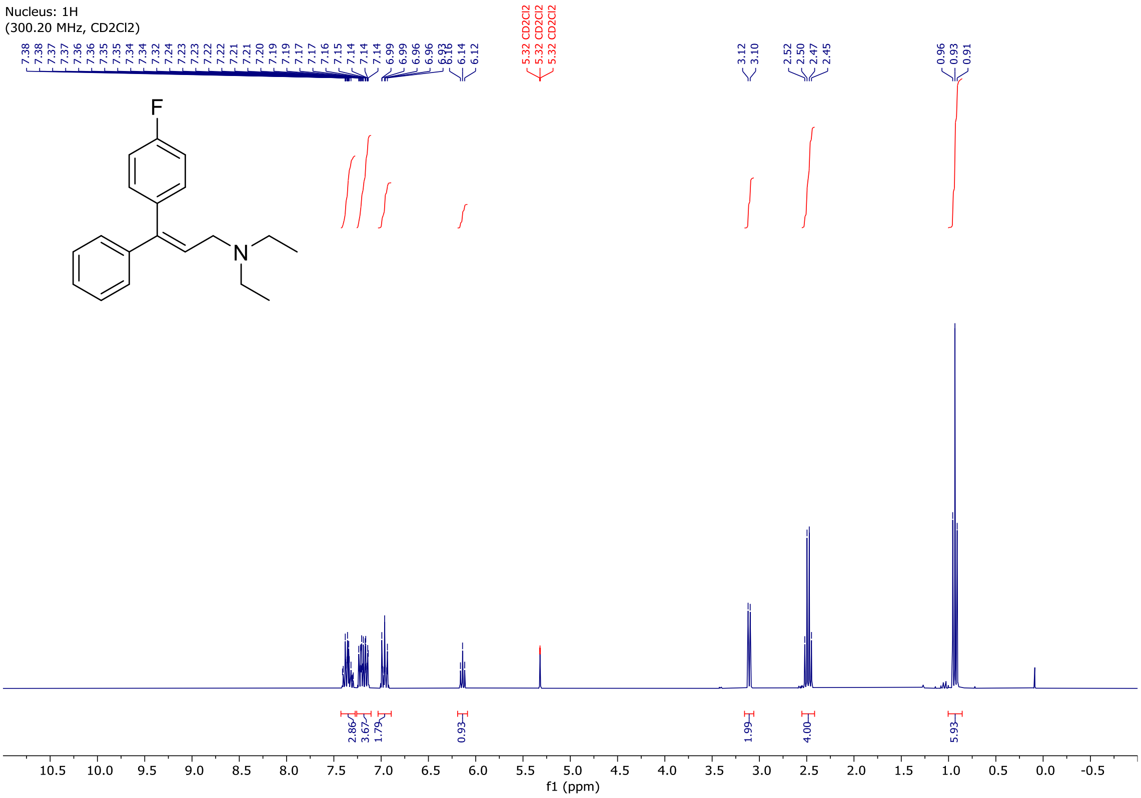

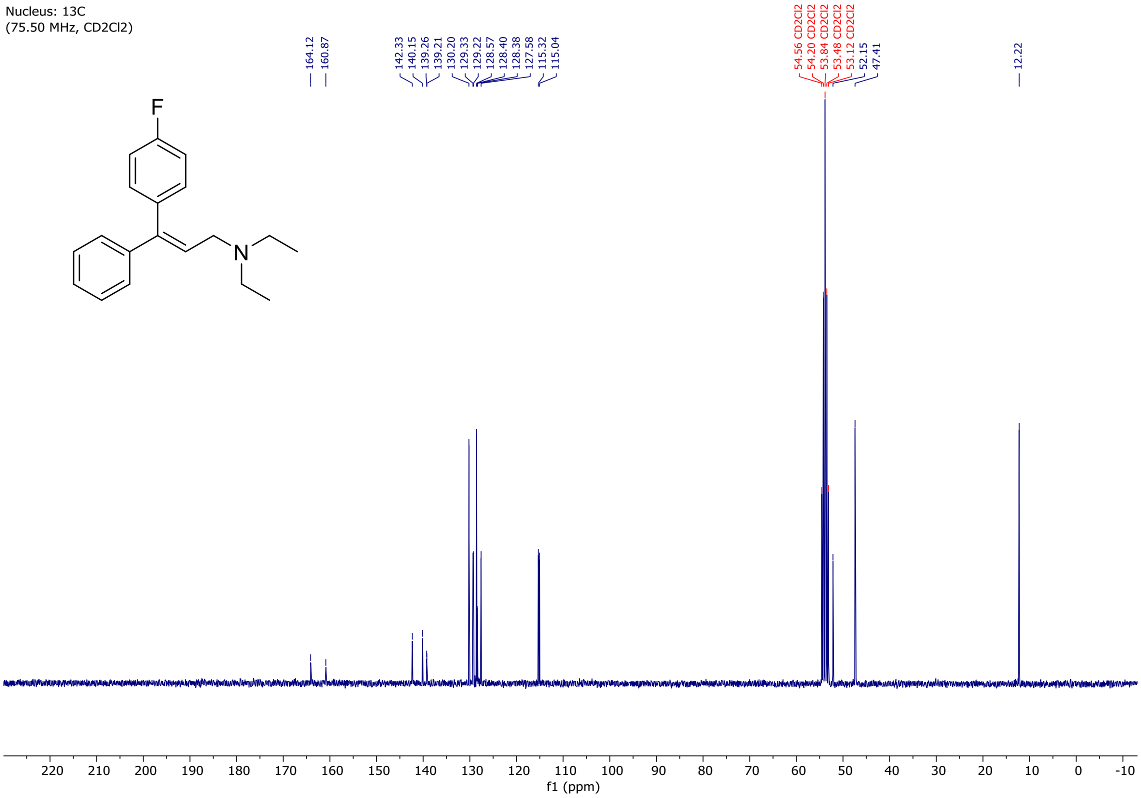

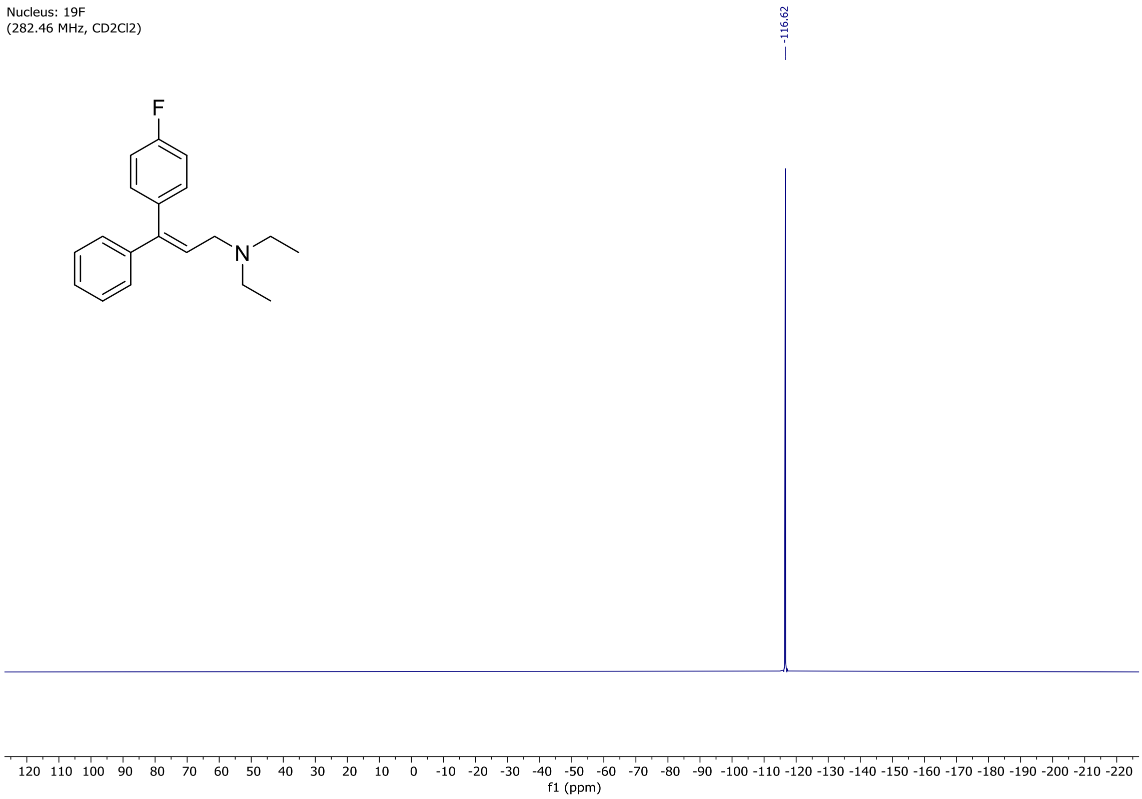
Nucleus: 19F
(282.46 MHz, CD2Cl2)
-116.62
F
N
120 110 100 90 80 70 60 50 40 30 20 10 0 -10 -20 -30 -40 -50 -60 -70 -80 -90 -100 -110 -120 -130 -140 -150 -160 -170 -180 -190 -200 -210 -220
f1 (ppm)

^{1}H NMR and ^{13}C NMR spectrum of compound **31a**

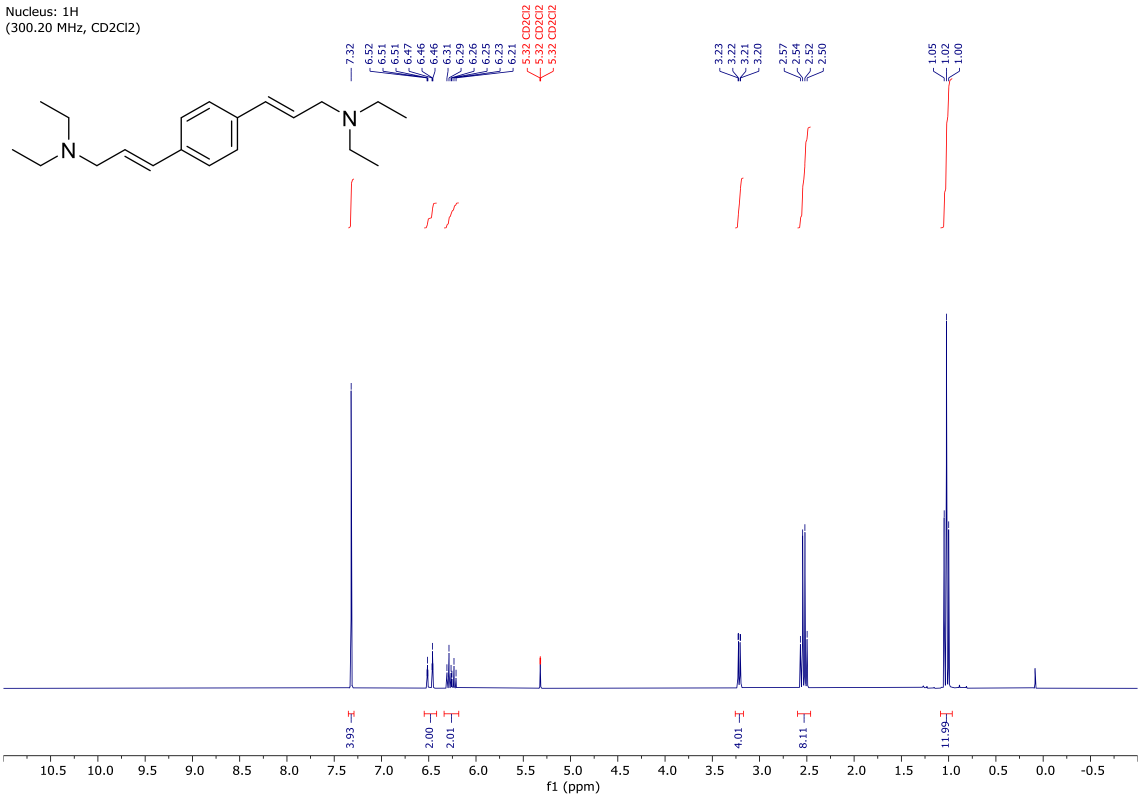

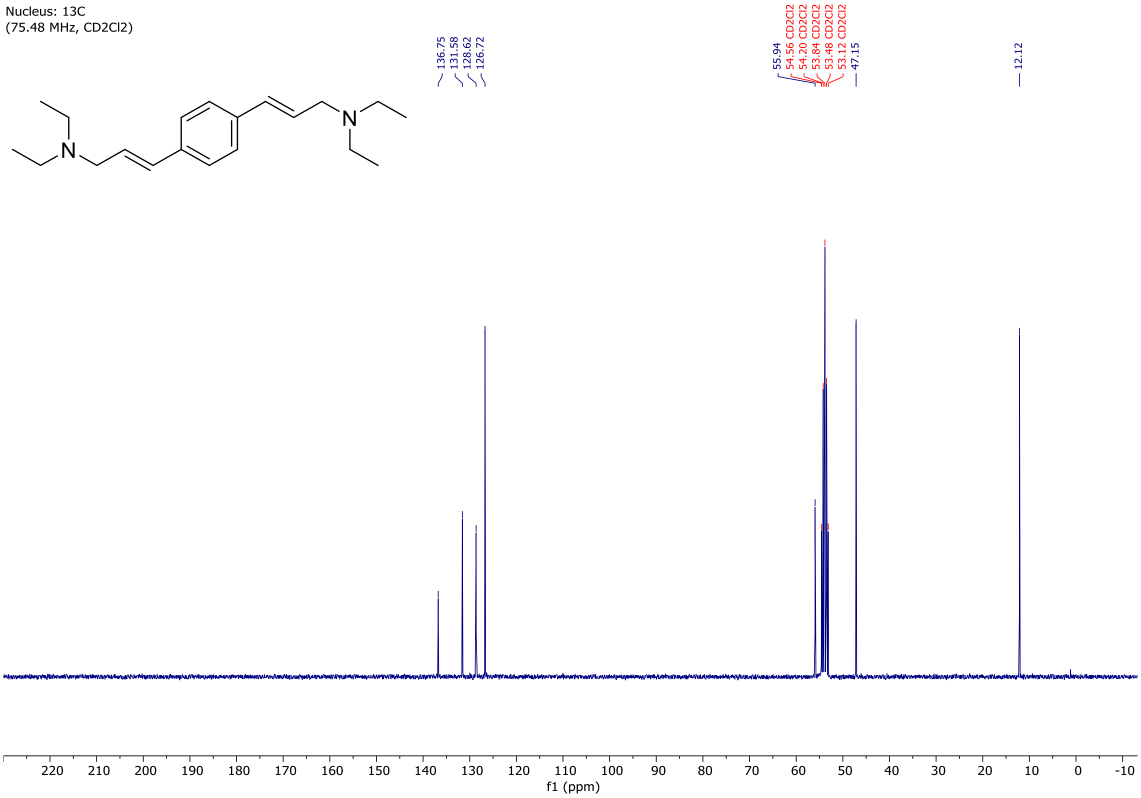

13.3 Isolated Enamines and Aldehydes

^{1}H NMR and ^{13}C NMR spectrum of compound **(*E*)-1b**

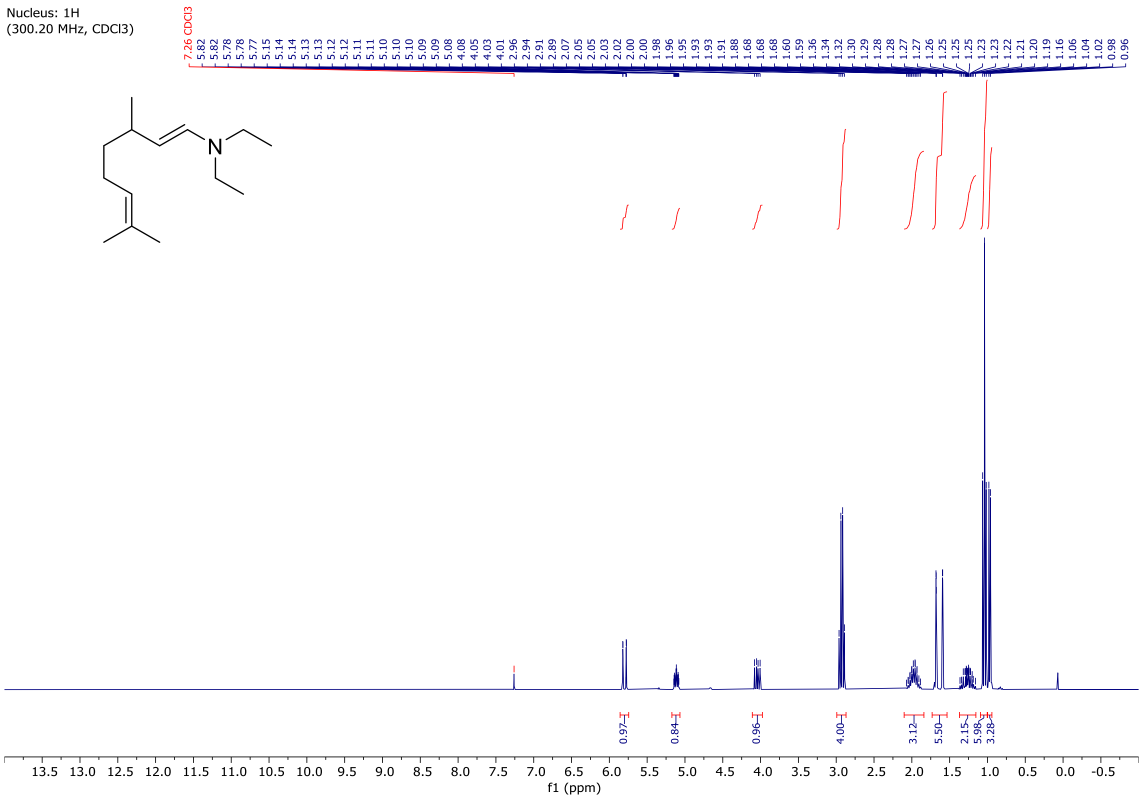

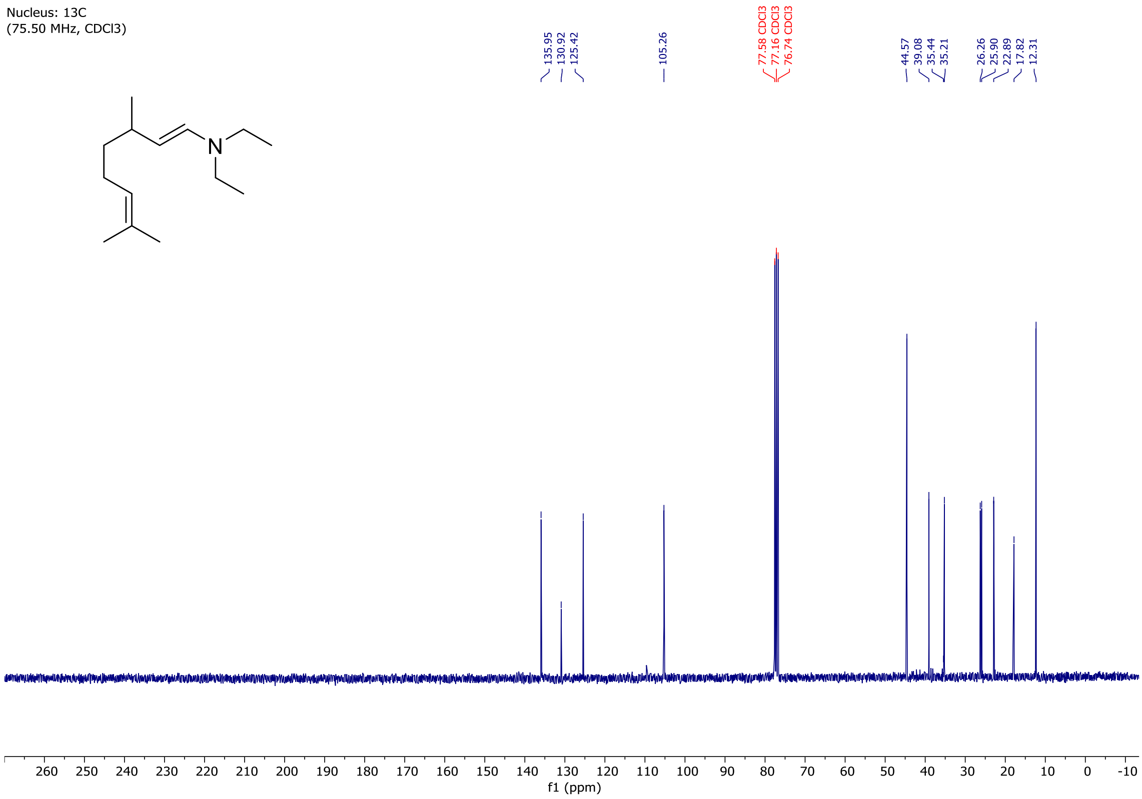

^{1}H NMR and ^{13}C NMR spectrum of compound **(*E*)-1c**

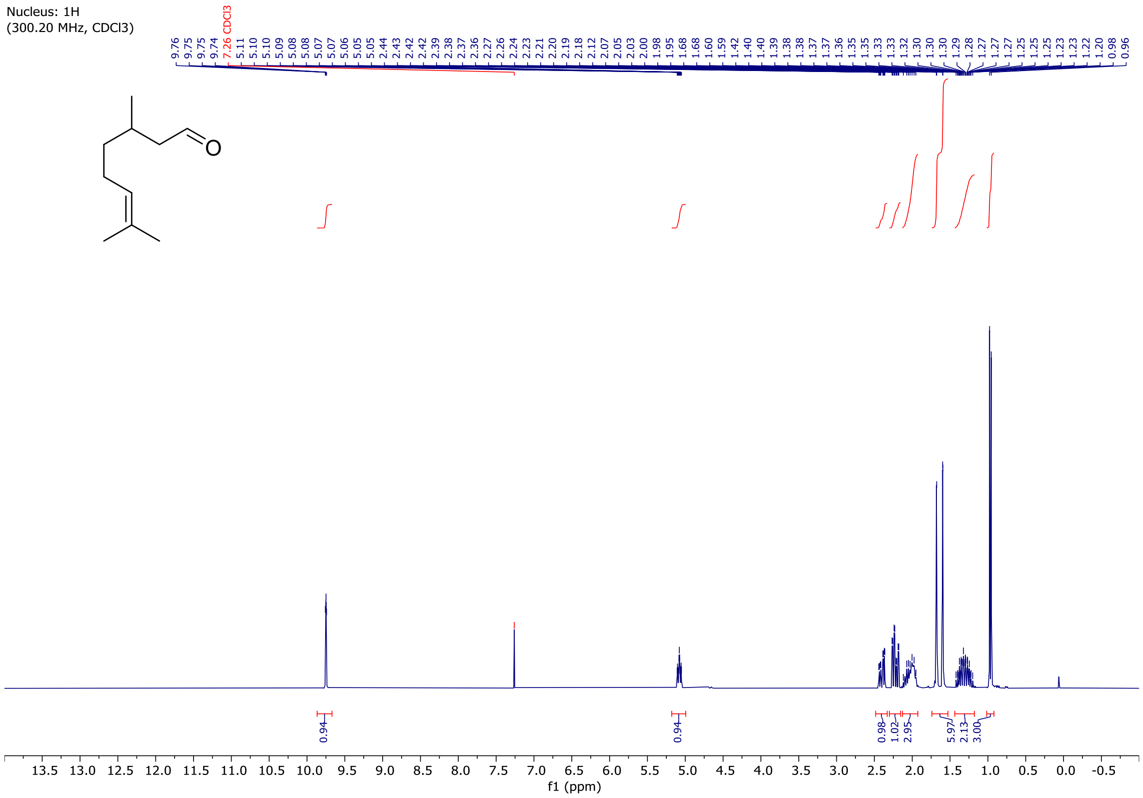

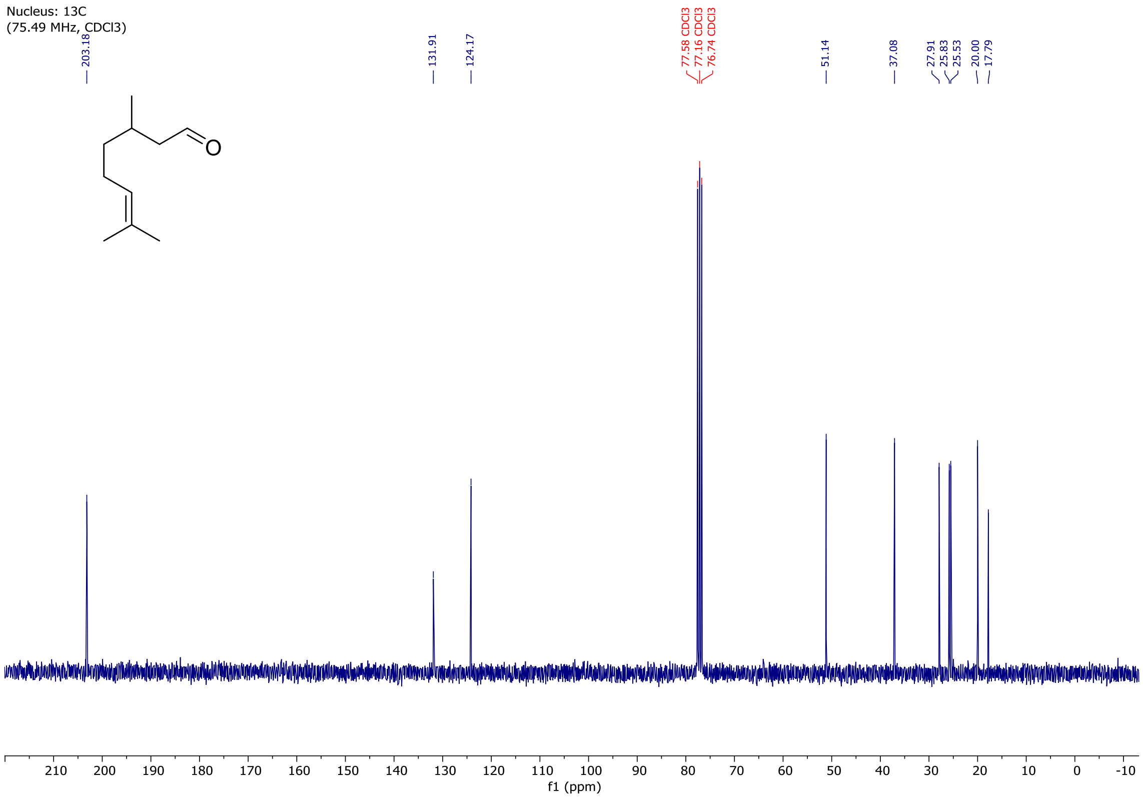

^{1}H NMR and ^{13}C NMR spectrum of compound **10c**

Nucleus: 1H
(300.20 MHz, CDCl3)

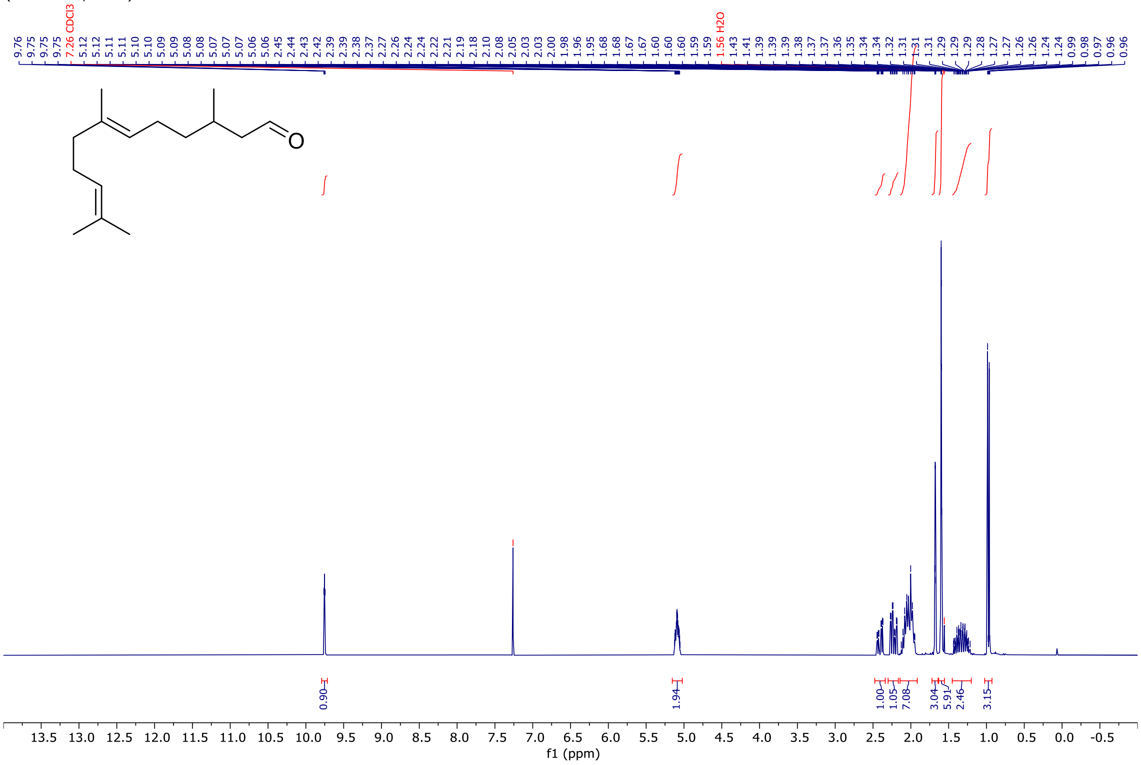

Nucleus: 13C
(75.50 MHz, CDCl3)

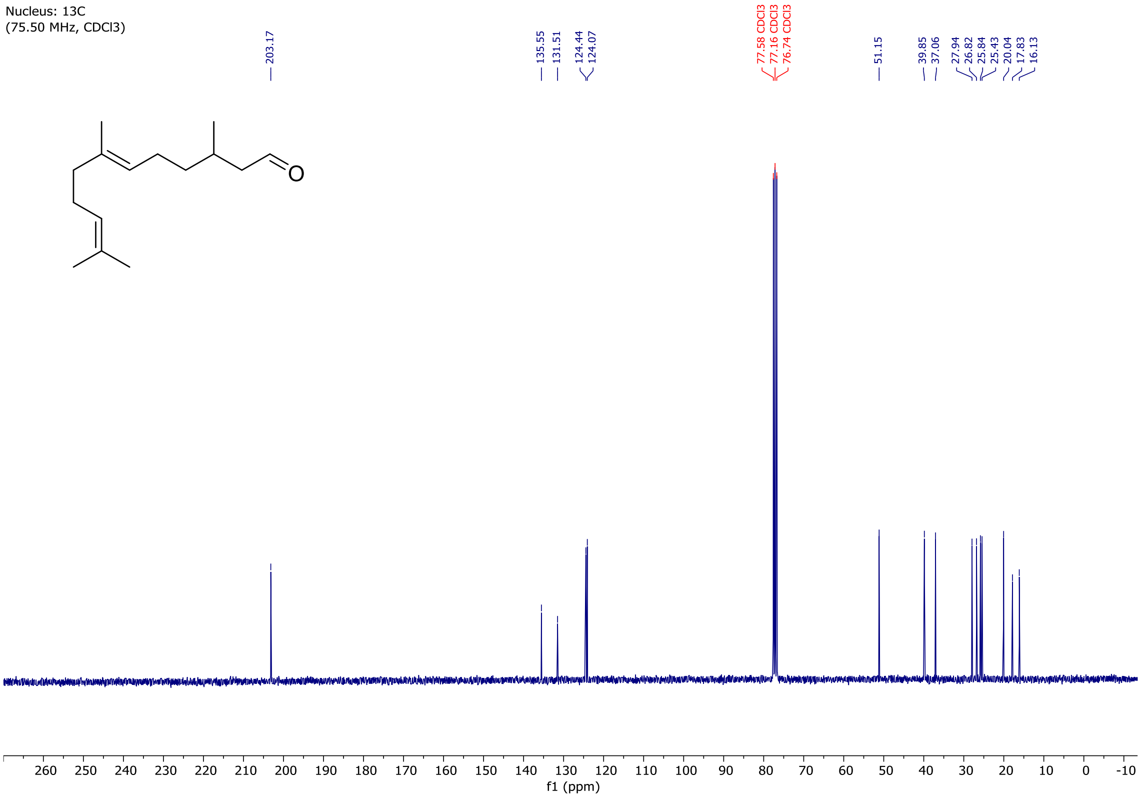

^{1}H NMR and ^{13}C NMR spectrum of compound **11c**

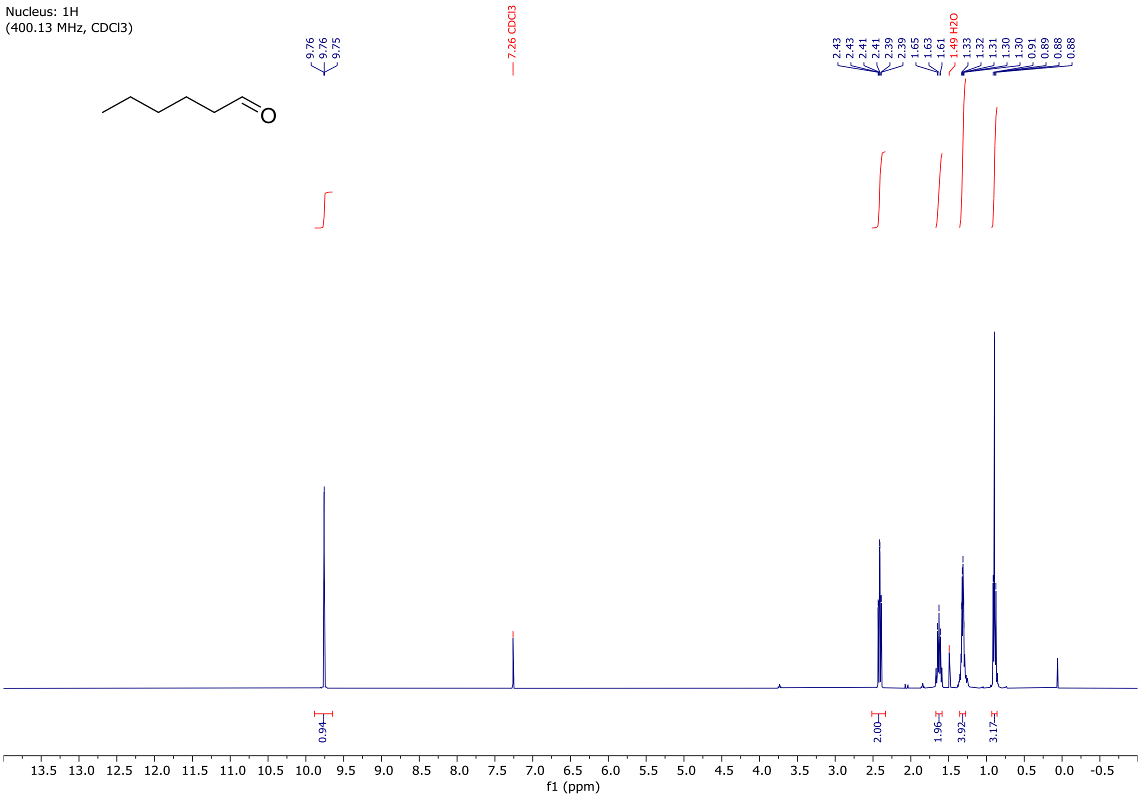

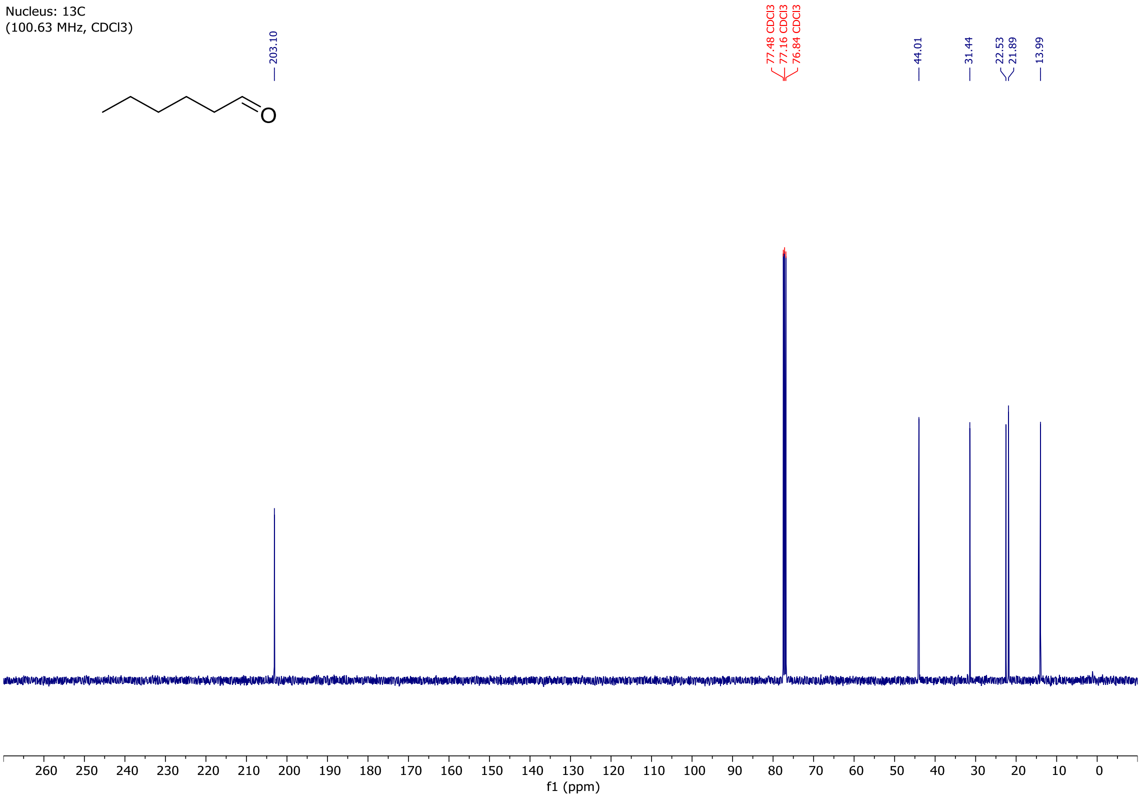

^{1}H NMR and ^{13}C NMR spectrum of compound **12c**

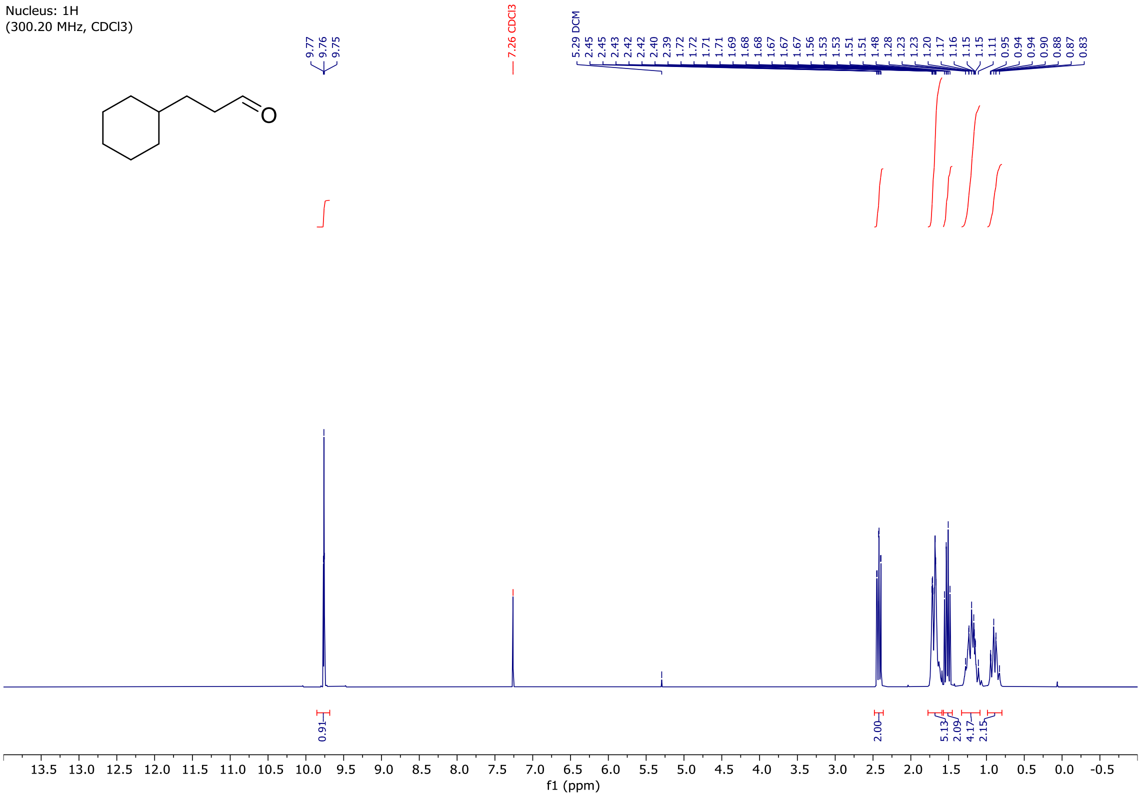

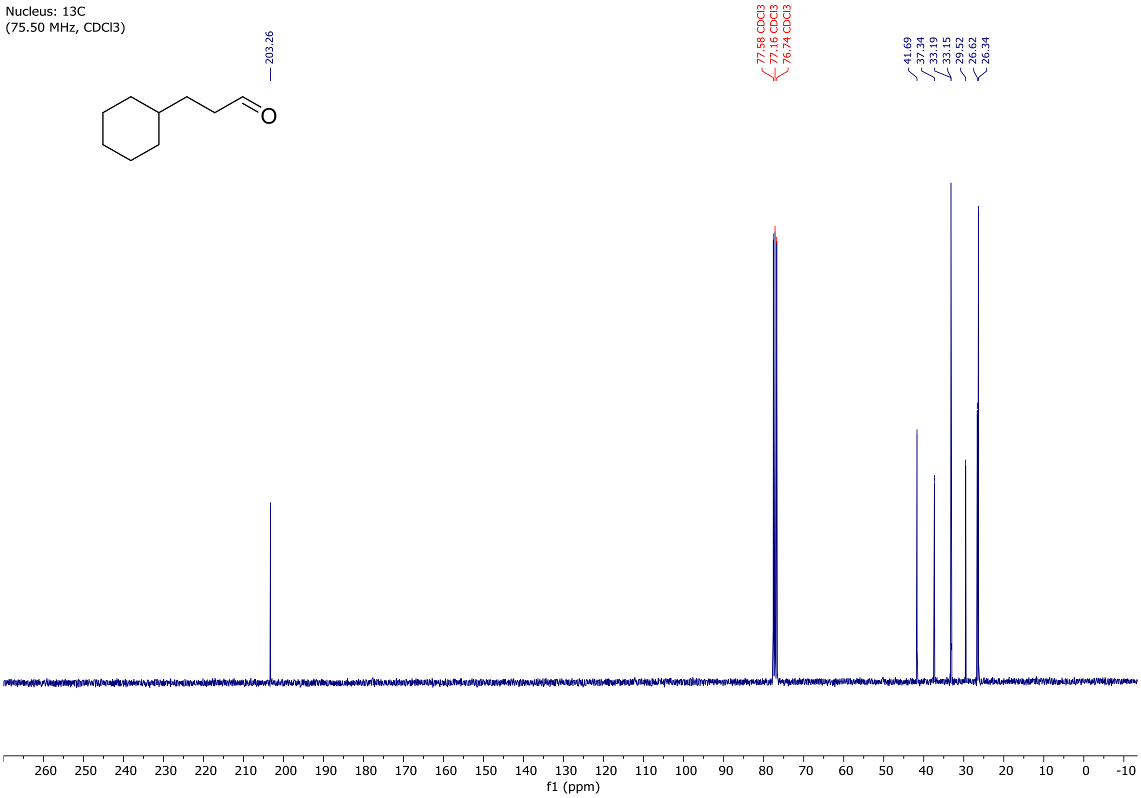

^{1}H NMR, ^{13}C NMR and ^{19}F NMR spectrum of compound **13b**

Nucleus: 1H
(300.20 MHz, CDCl3)

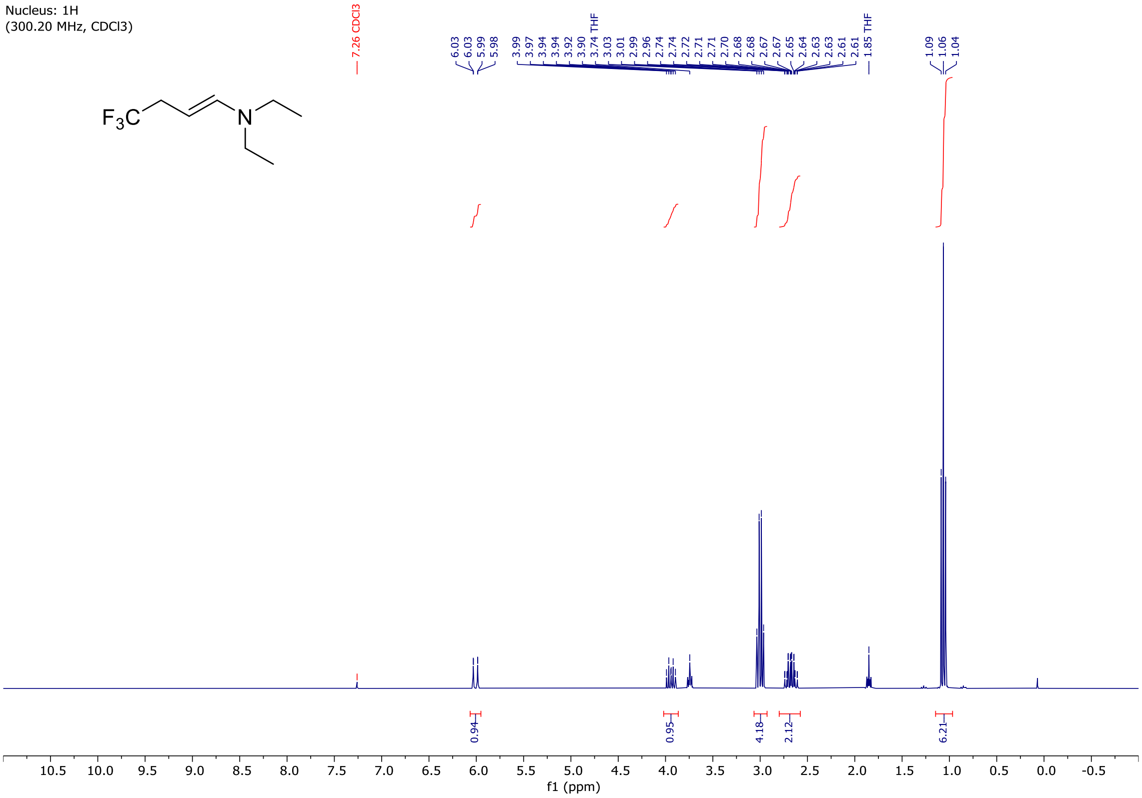

Nucleus: 13C
(75.50 MHz, CDCl3)

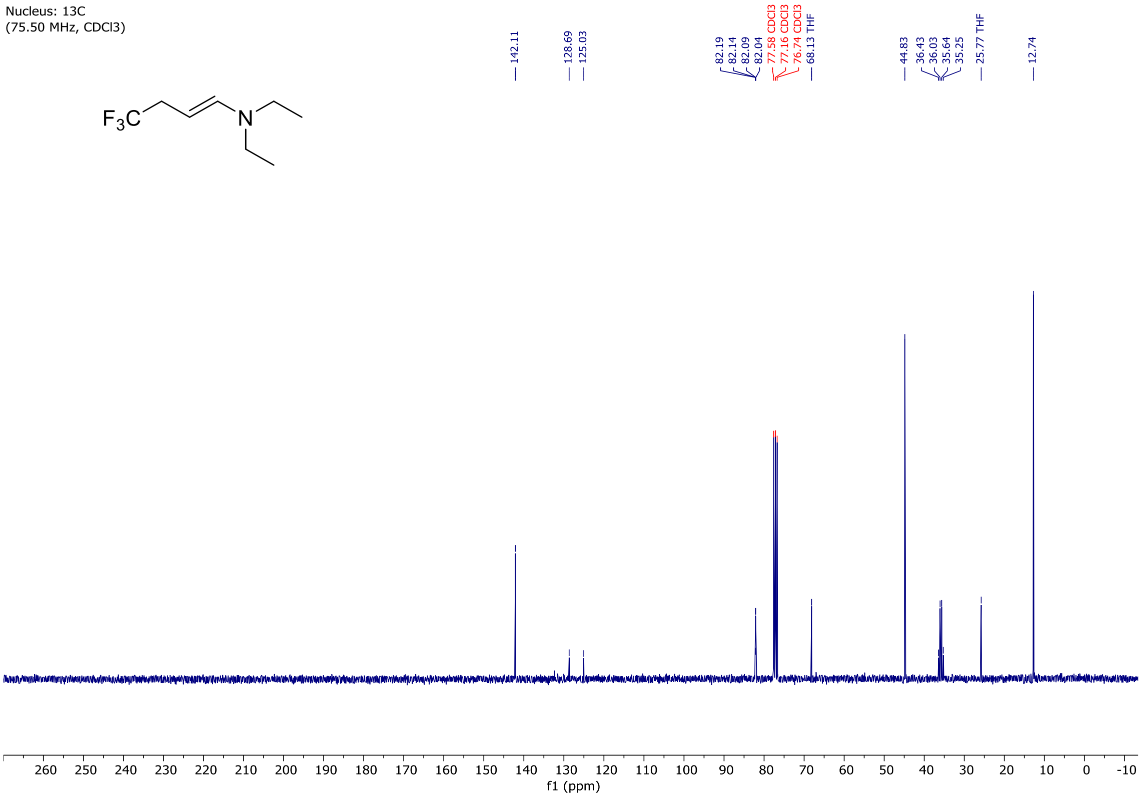

Nucleus: 19F
(282.46 MHz, CDCl3)

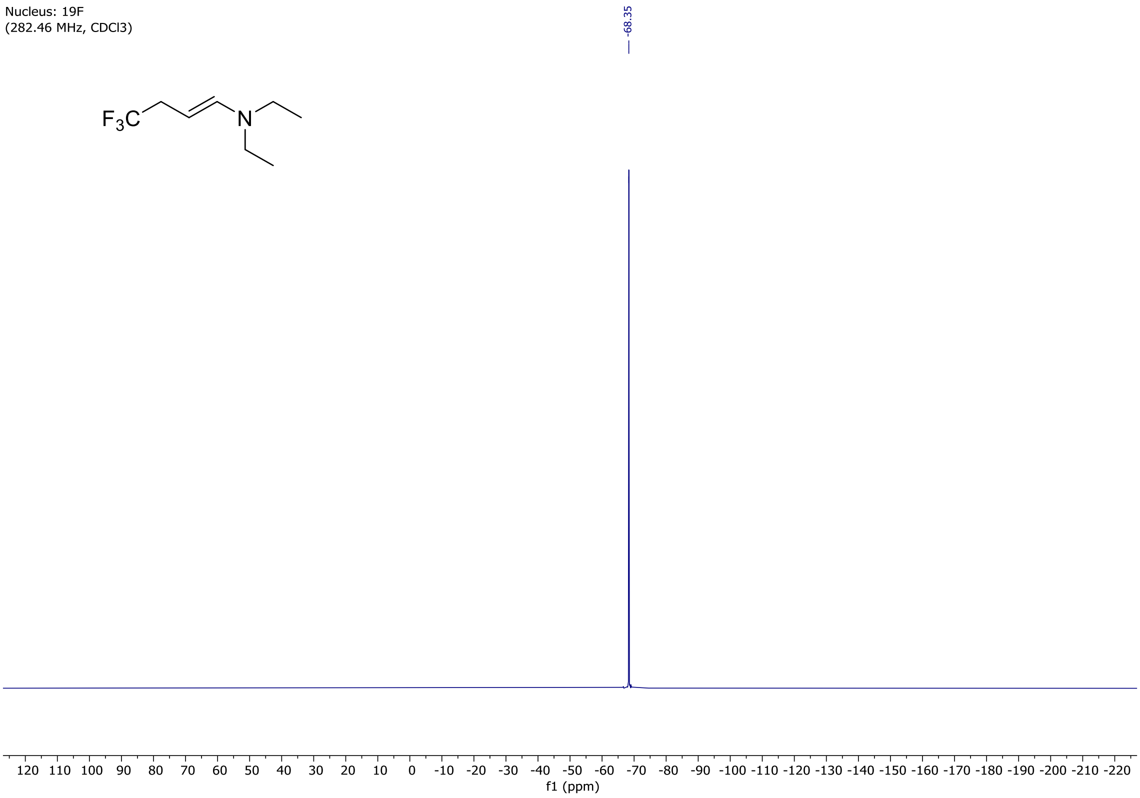

^{1}H NMR and ^{13}C NMR spectrum of compound **15c**

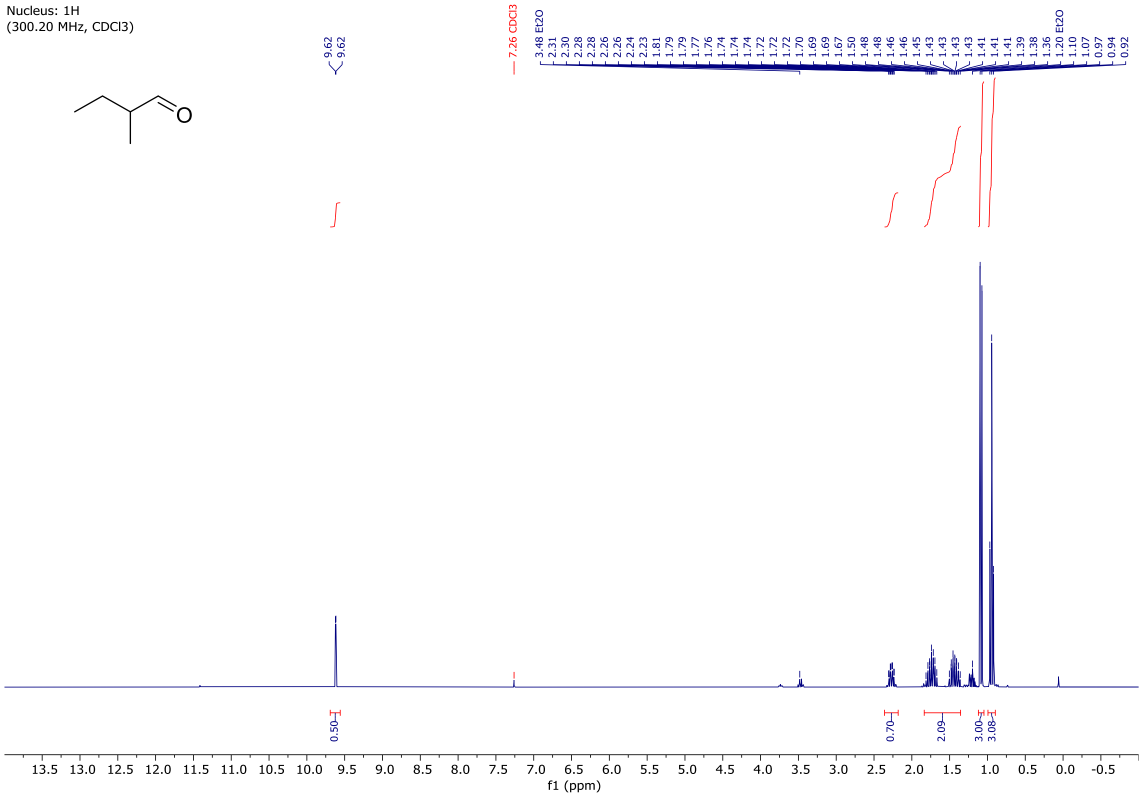

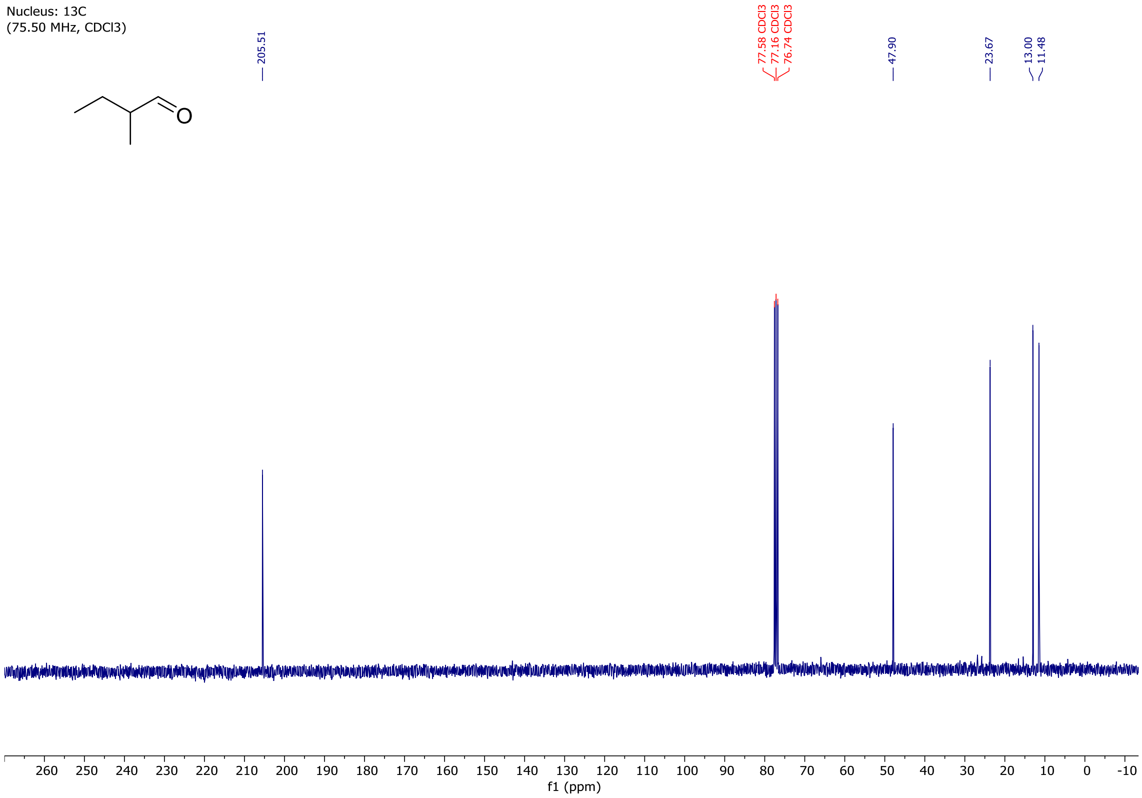

^{1}H NMR and ^{13}C NMR spectrum of compound **16b**

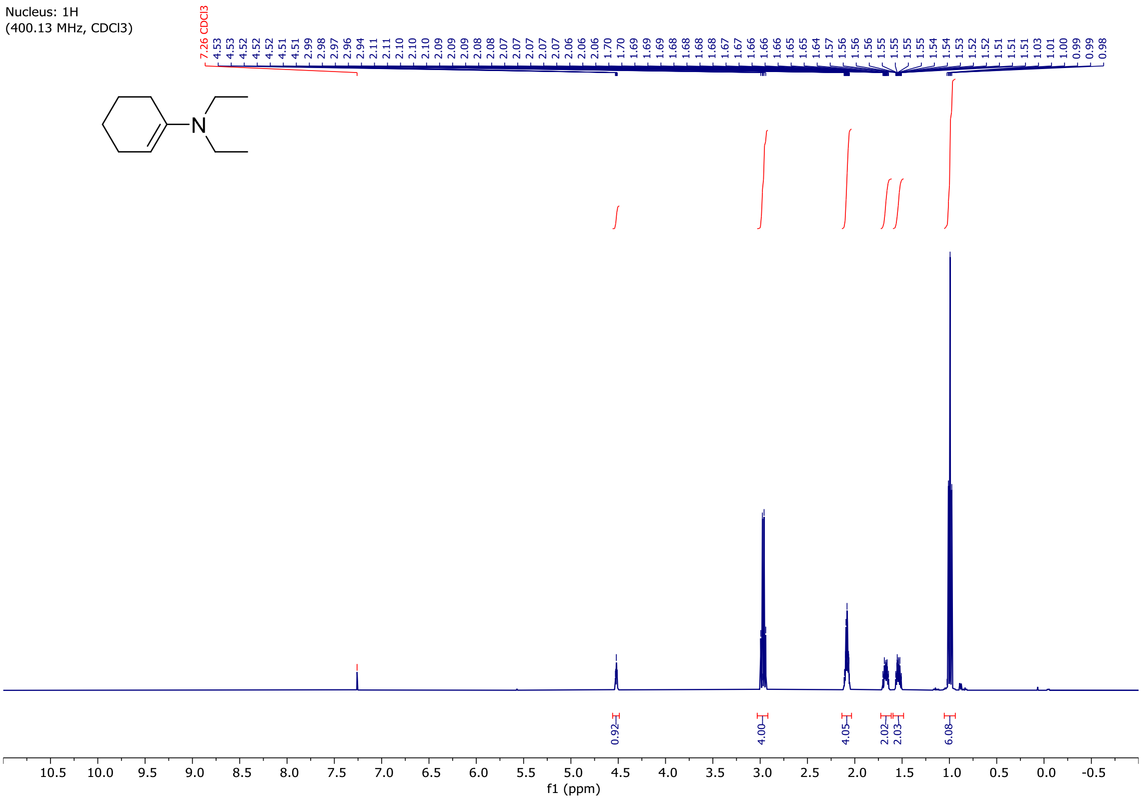

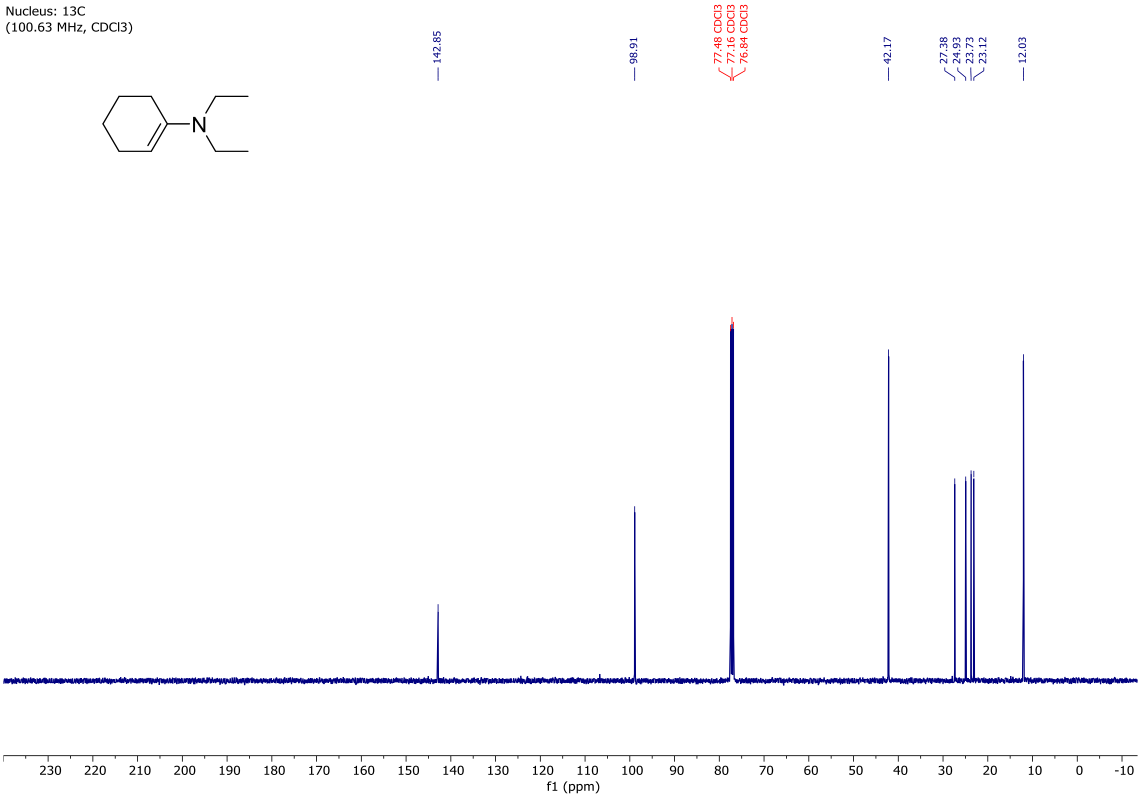

^{1}H NMR and ^{13}C NMR spectrum of compound **17c**

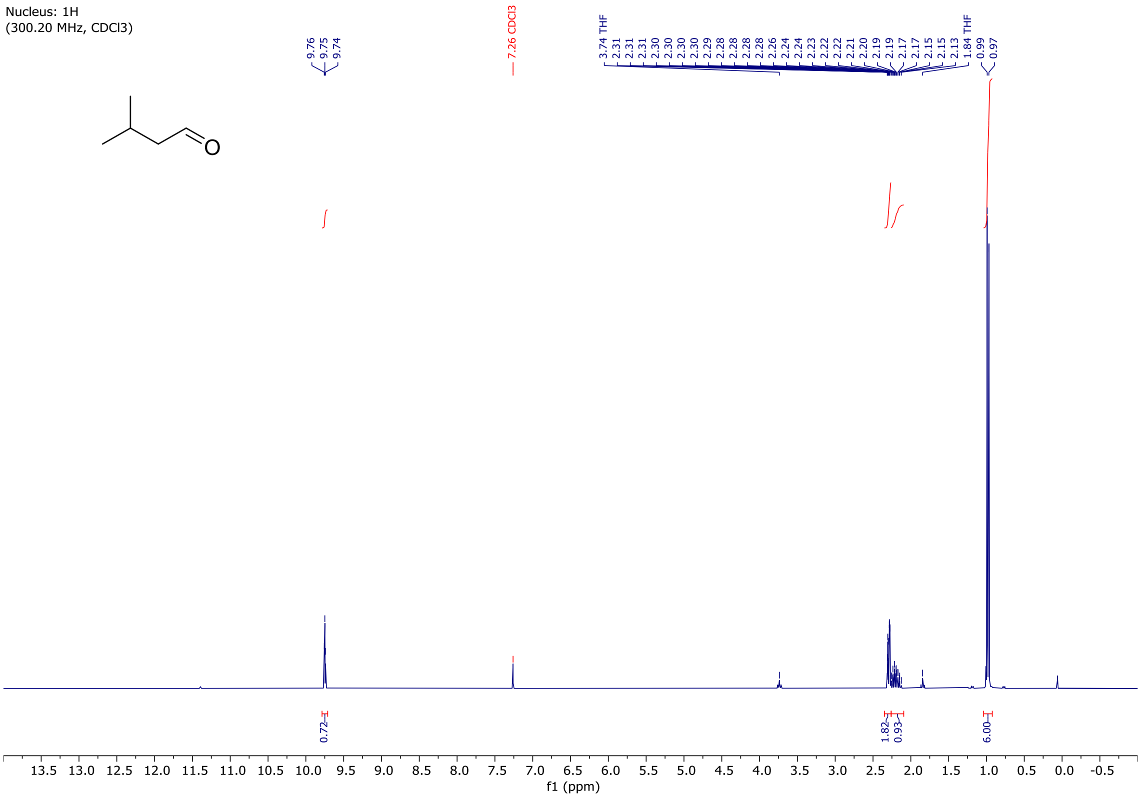

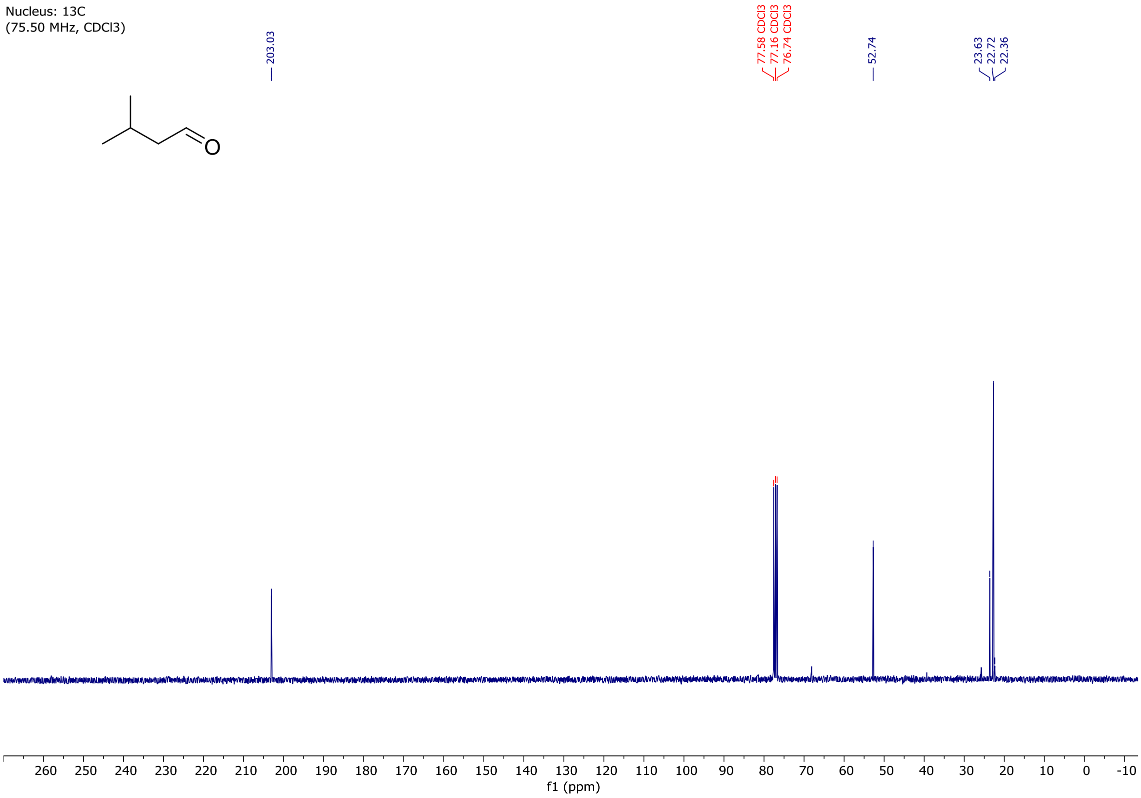

^{1}H NMR and ^{13}C NMR spectrum of compound **18c**

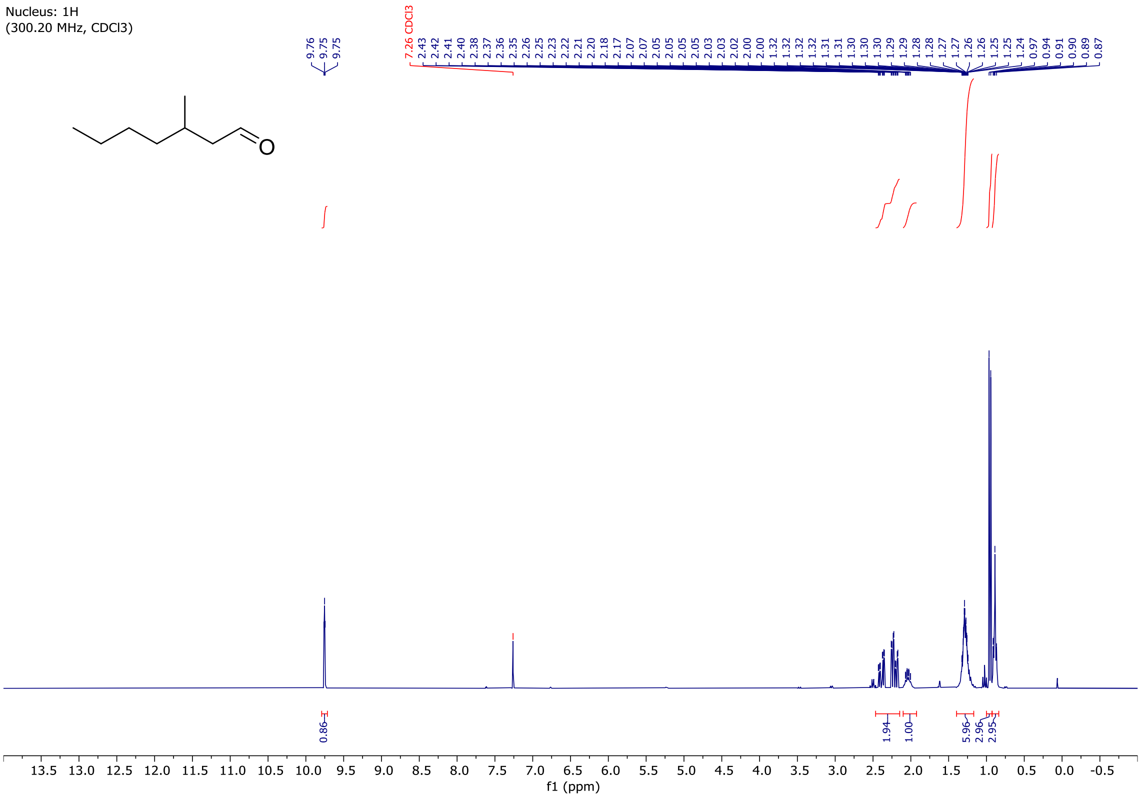

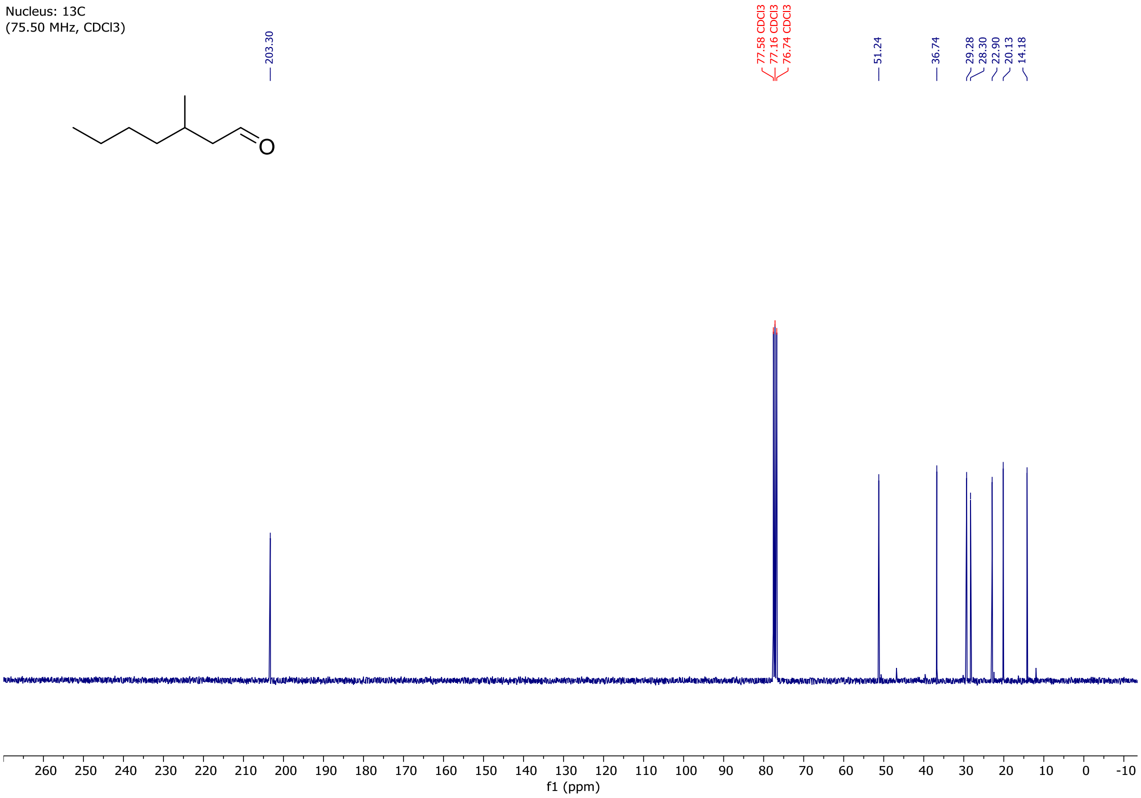

^{1}H NMR and ^{13}C NMR spectrum of compound **20c**

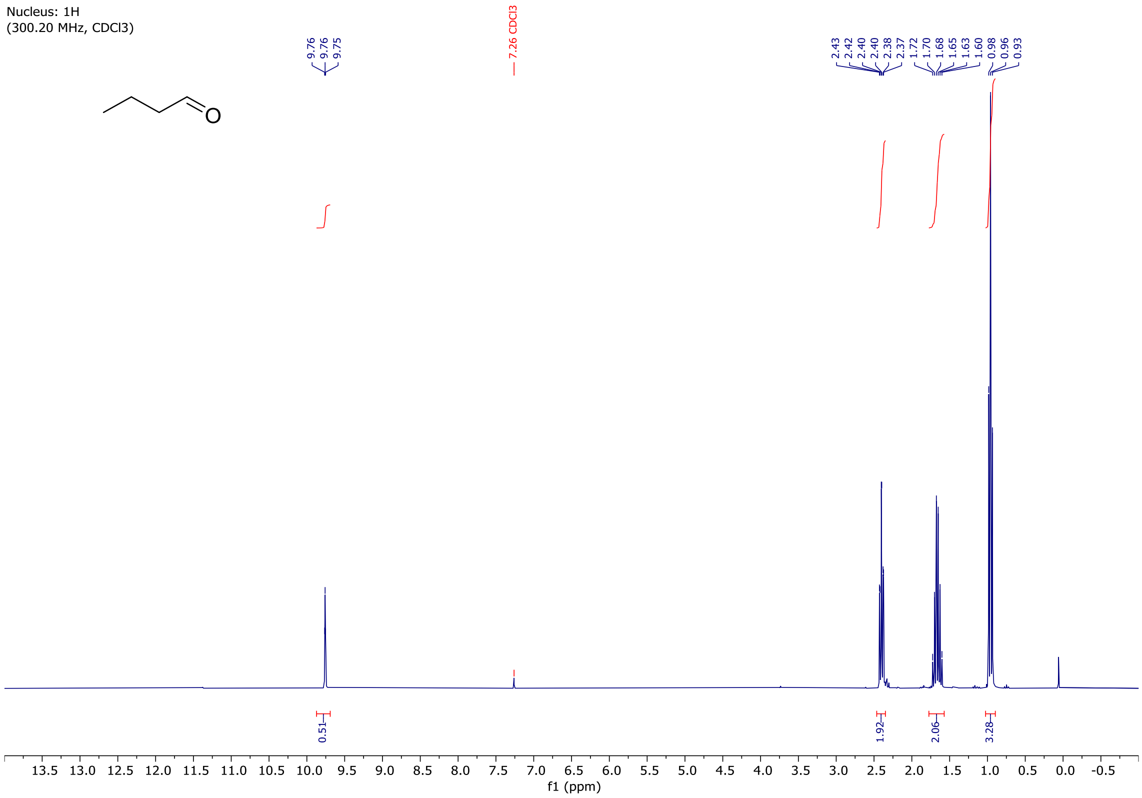

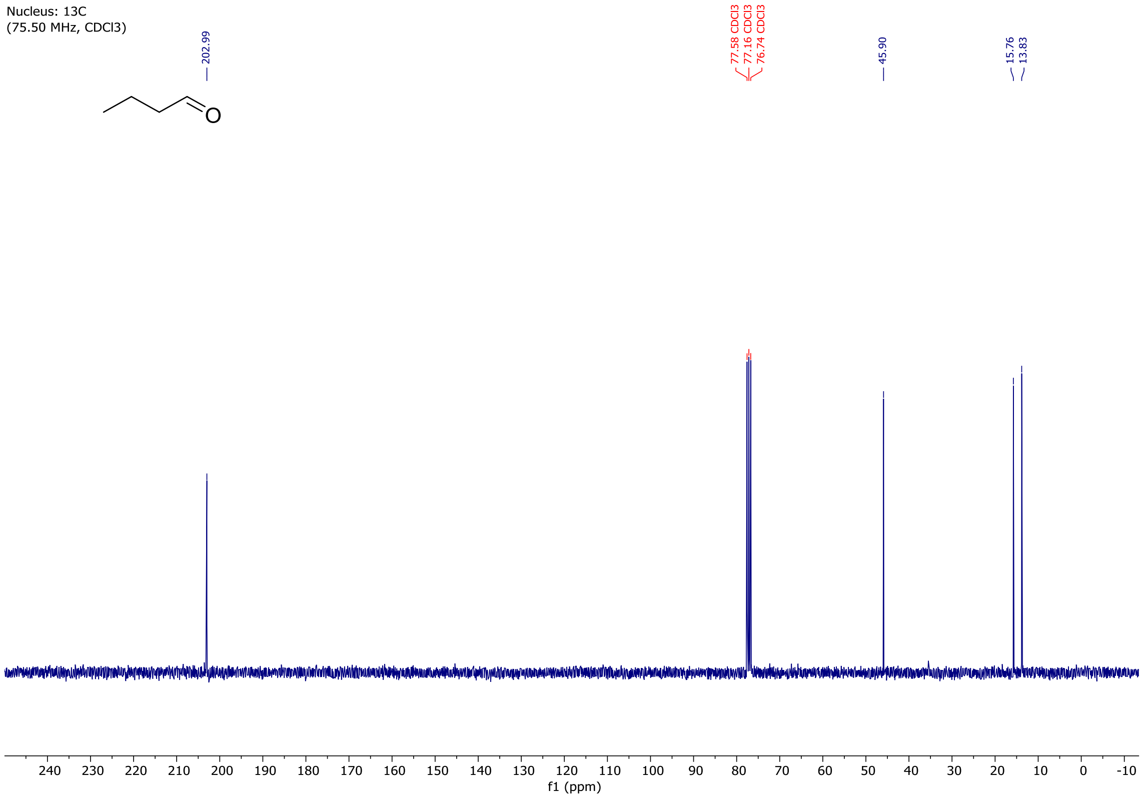

^{1}H NMR and ^{13}C NMR spectrum of compound **22b**

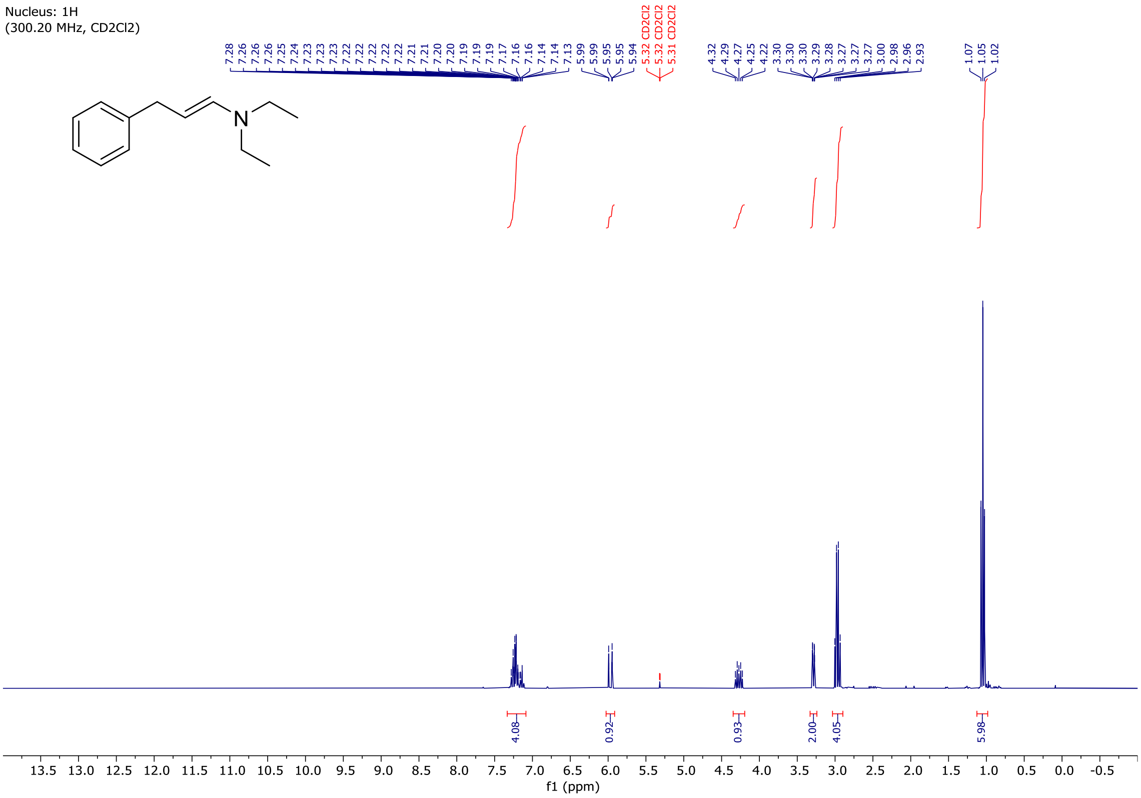

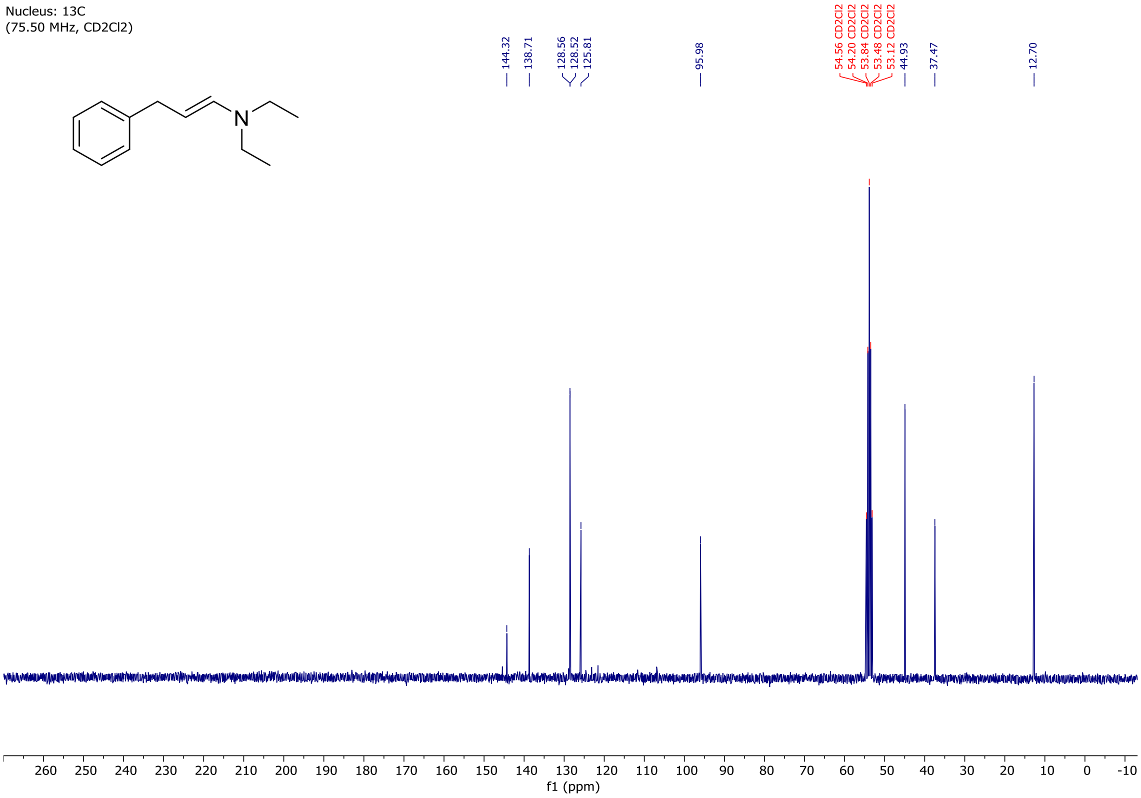

^{1}H NMR and ^{13}C NMR spectrum of compound **22c**

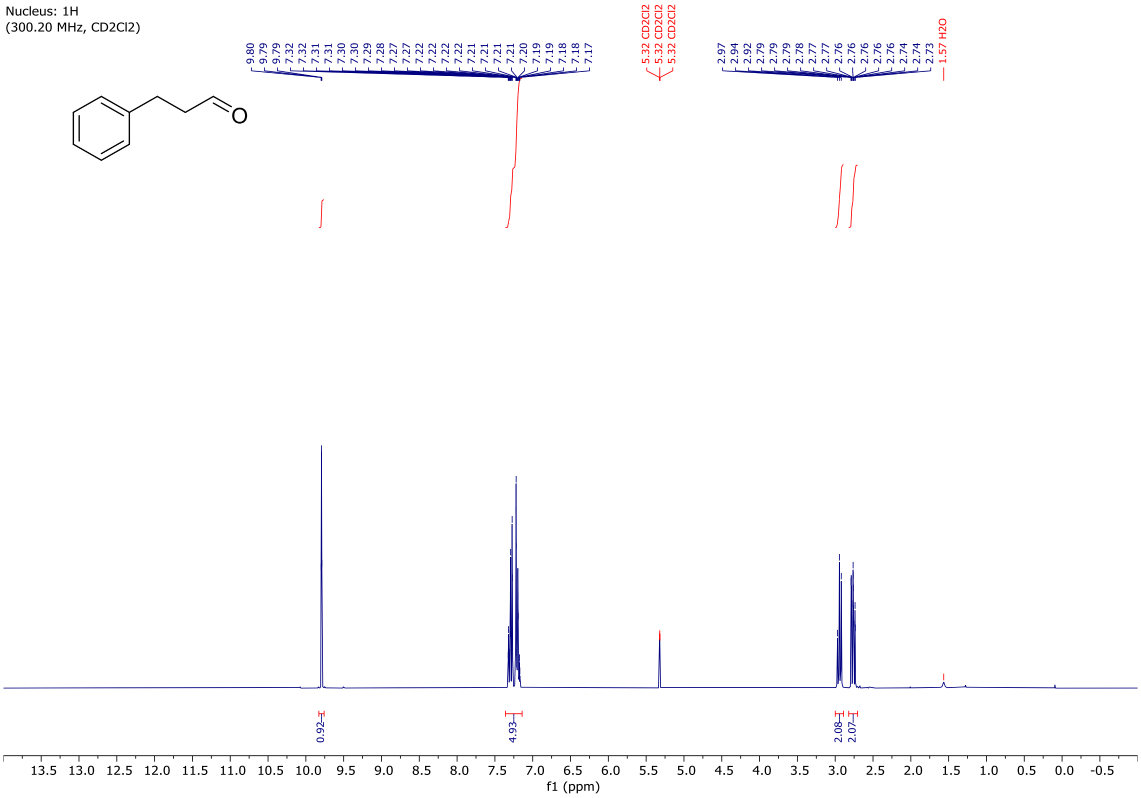

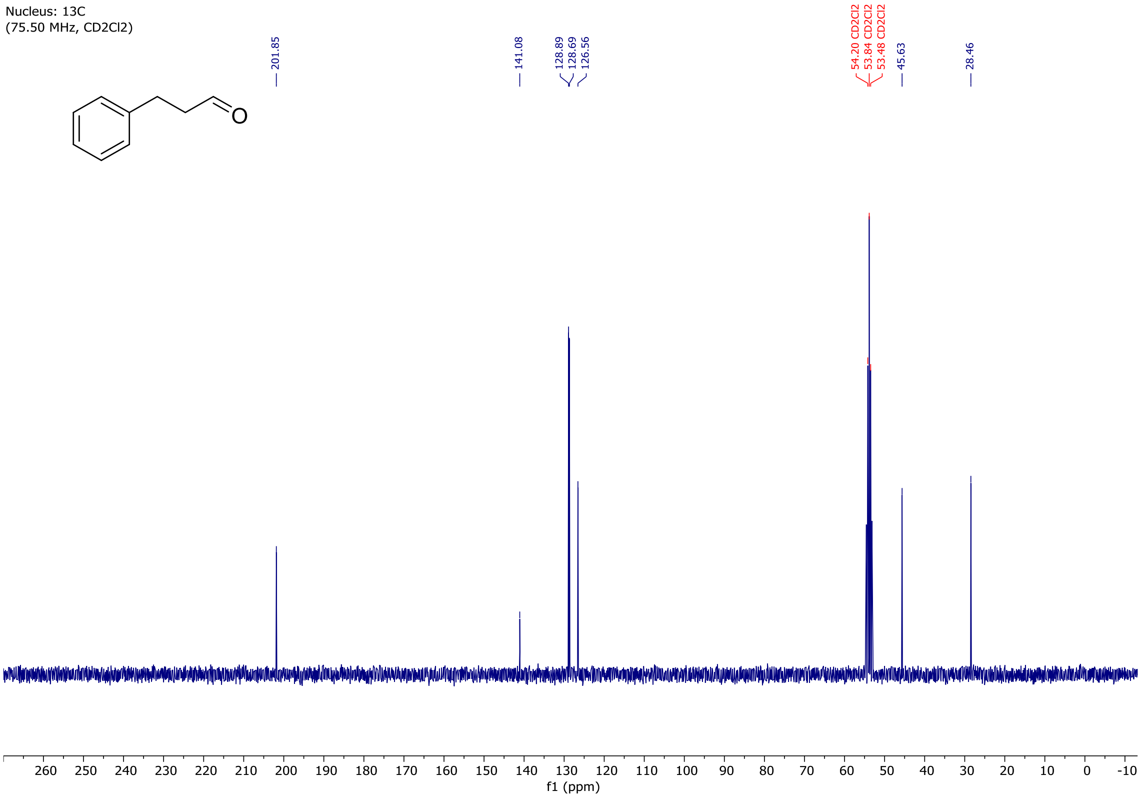

^{1}H NMR and ^{13}C NMR spectrum of compound **23c**

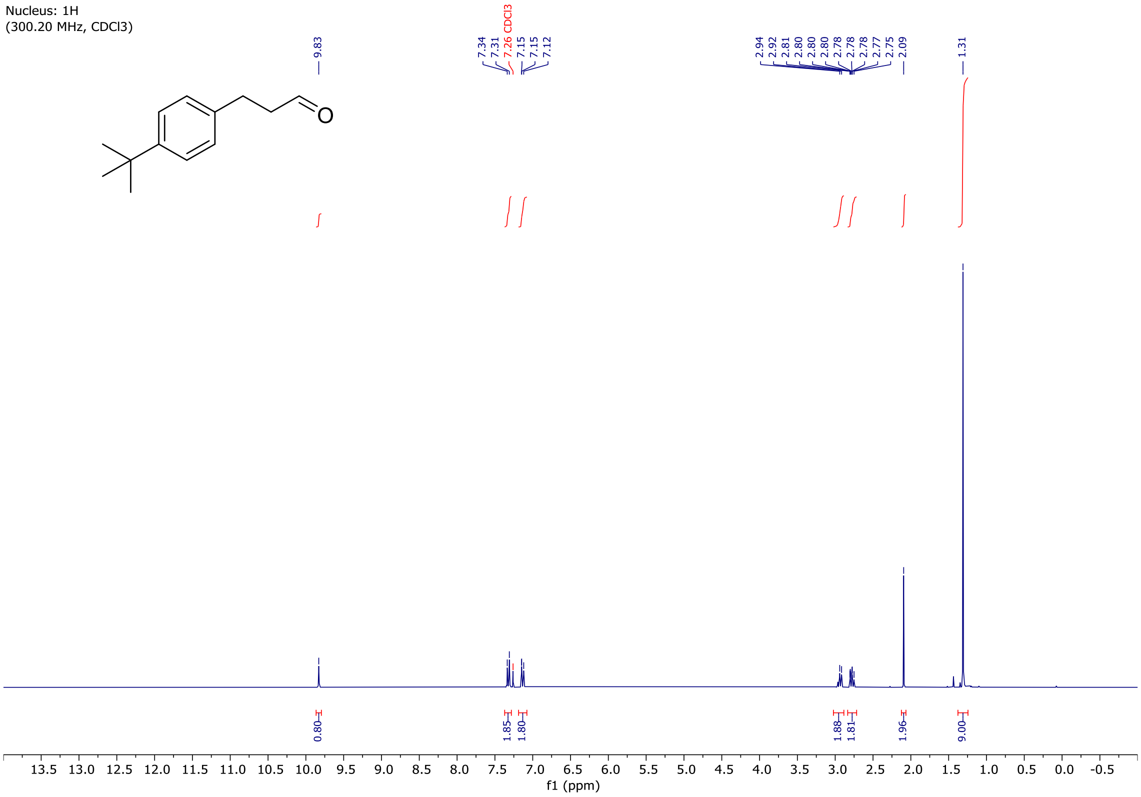

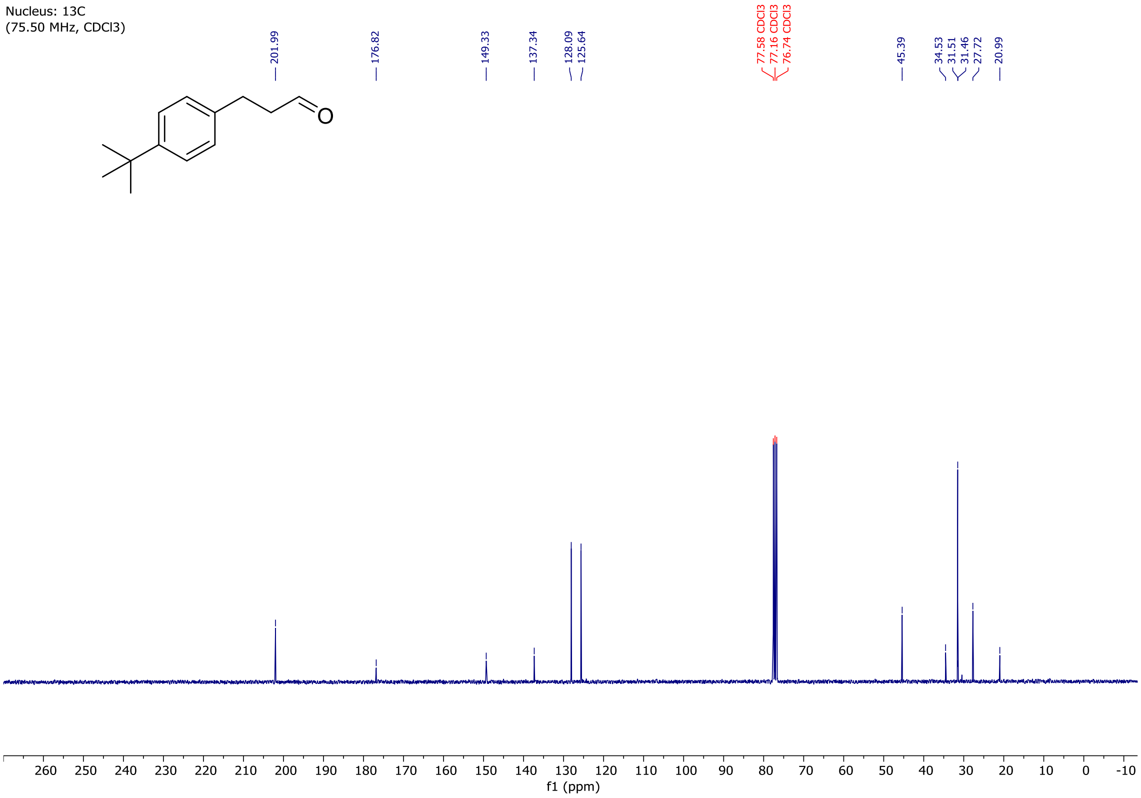

^{1}H NMR and ^{13}C NMR spectrum of compound **24c**

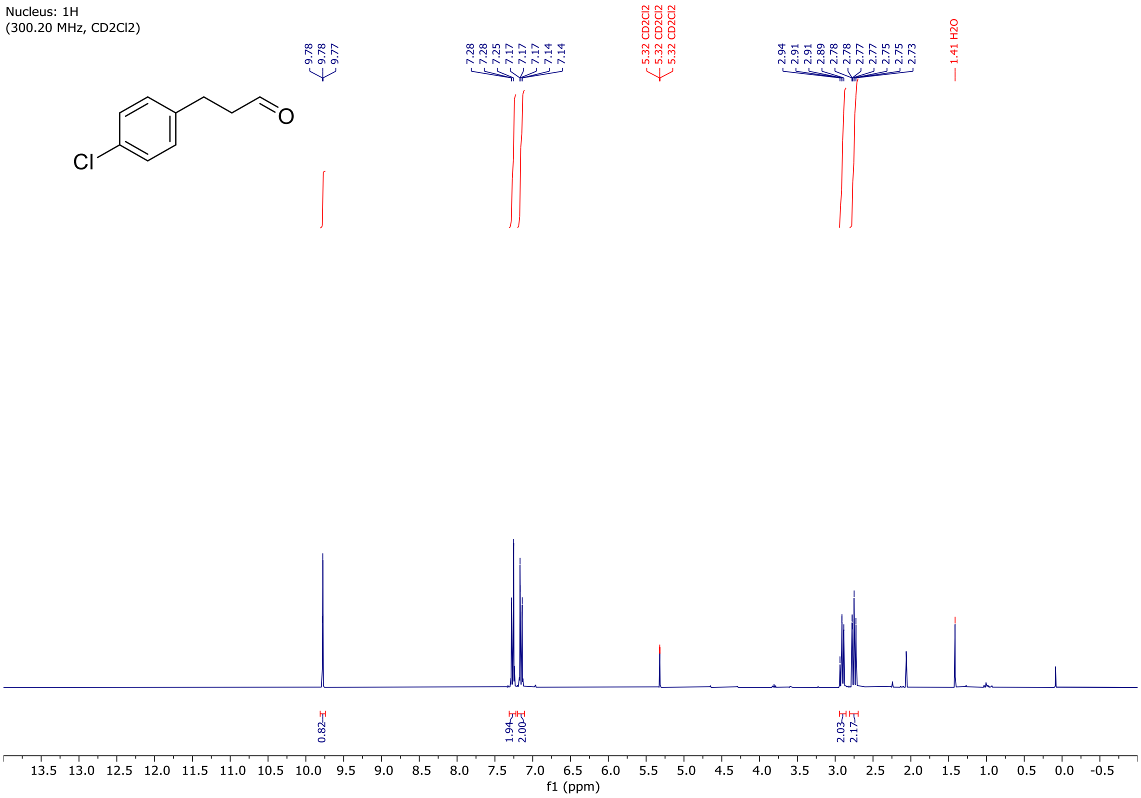

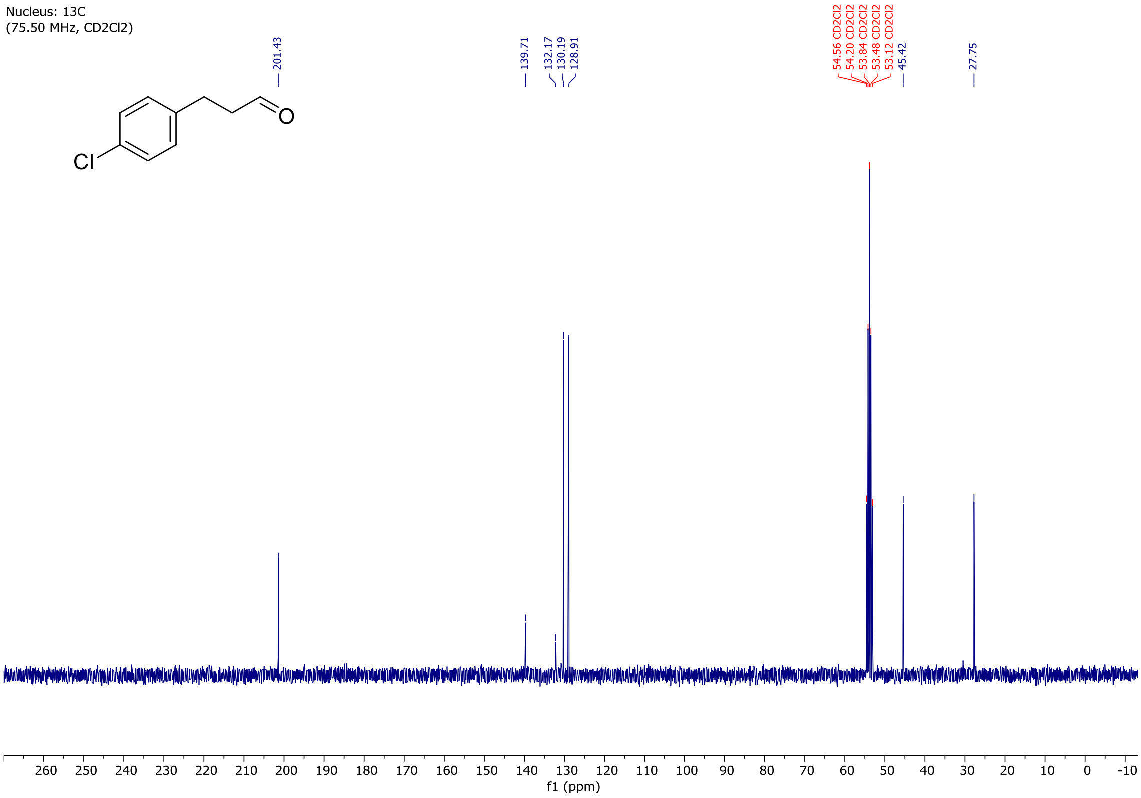

^{1}H NMR, ^{13}C NMR and ^{19}F NMR spectrum of compound **25c**

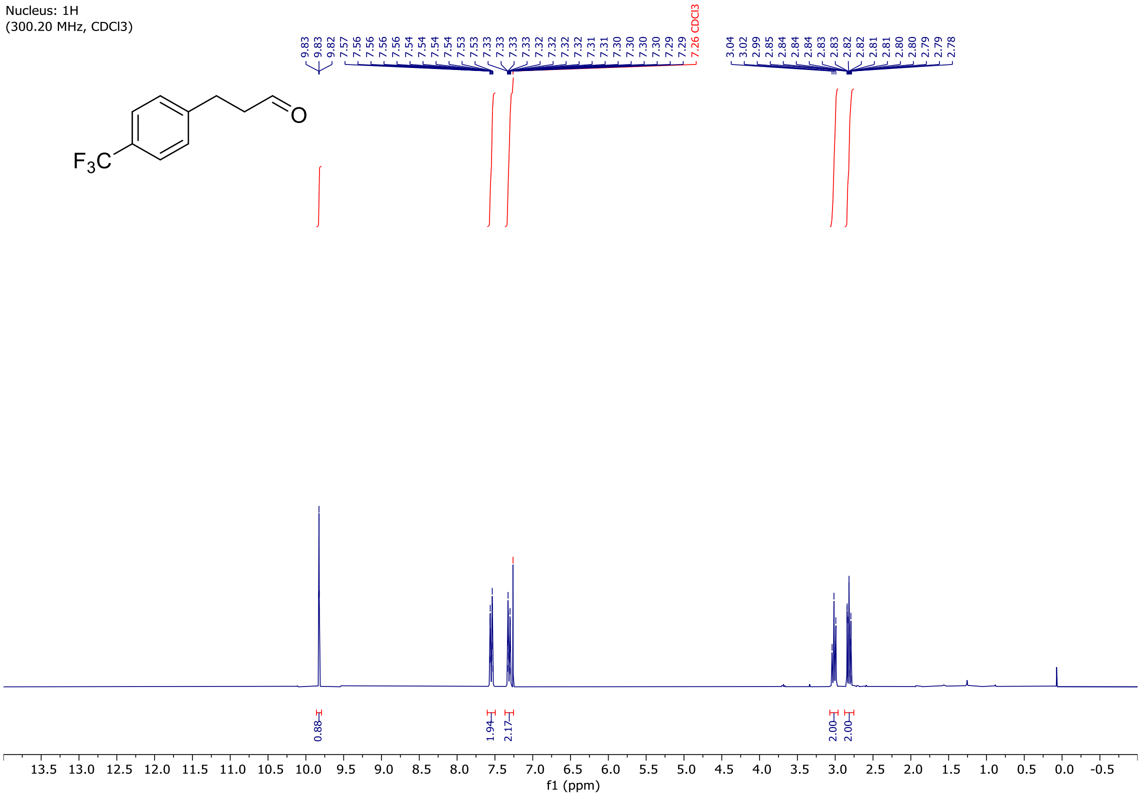

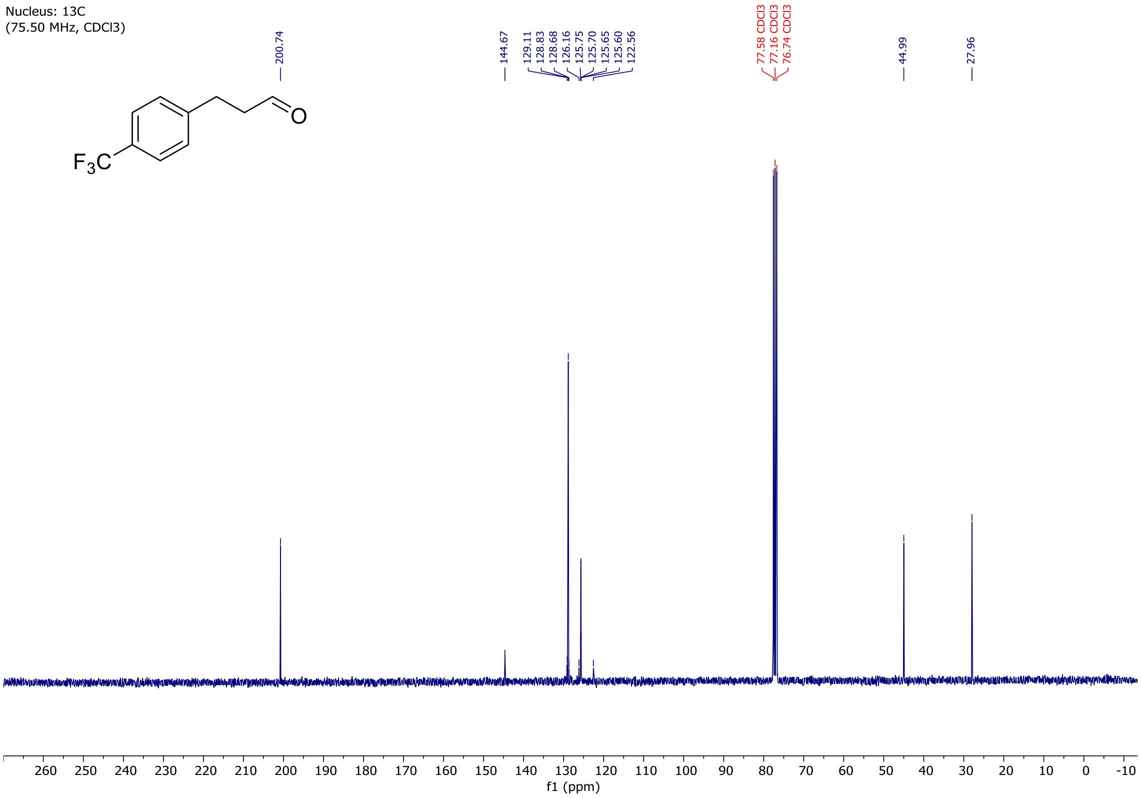

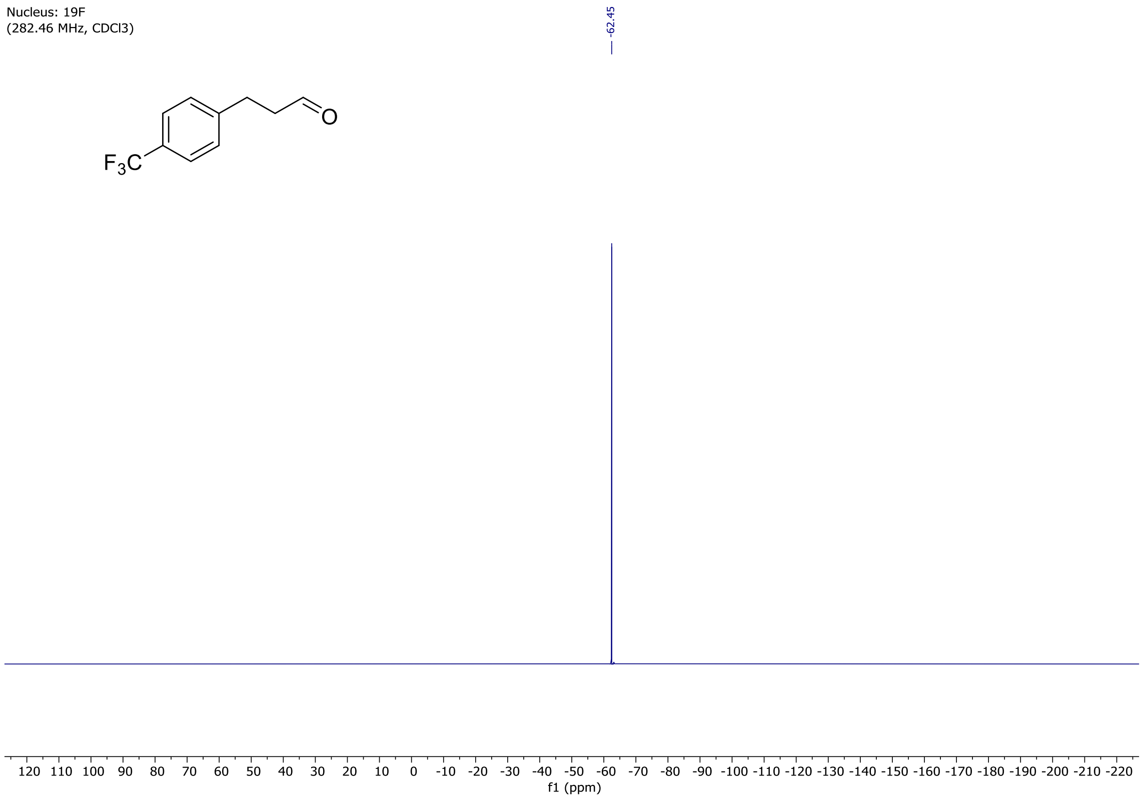
Nucleus: 19F
(282.46 MHz, CDCl3)
-62.45
F3C
O
120 110 100 90 80 70 60 50 40 30 20 10 0 -10 -20 -30 -40 -50 -60 -70 -80 -90 -100 -110 -120 -130 -140 -150 -160 -170 -180 -190 -200 -210 -220
f1 (ppm)

^{1}H NMR and ^{13}C NMR spectrum of compound **26c**

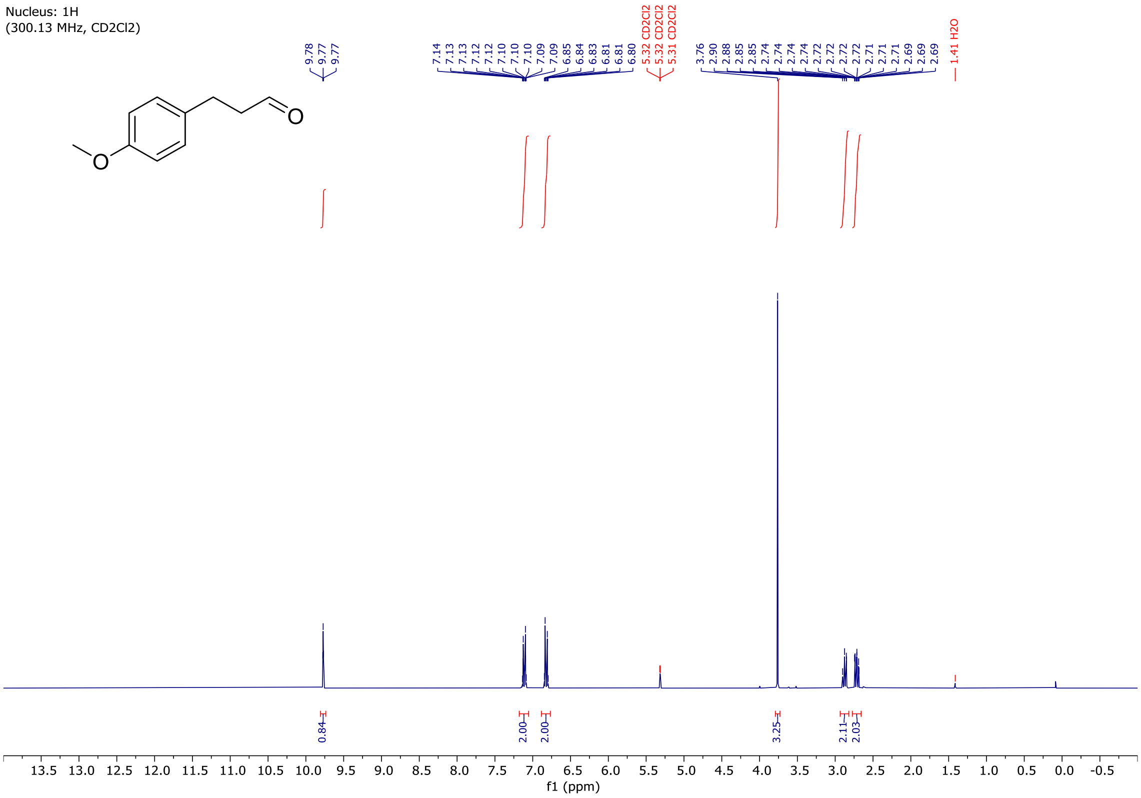

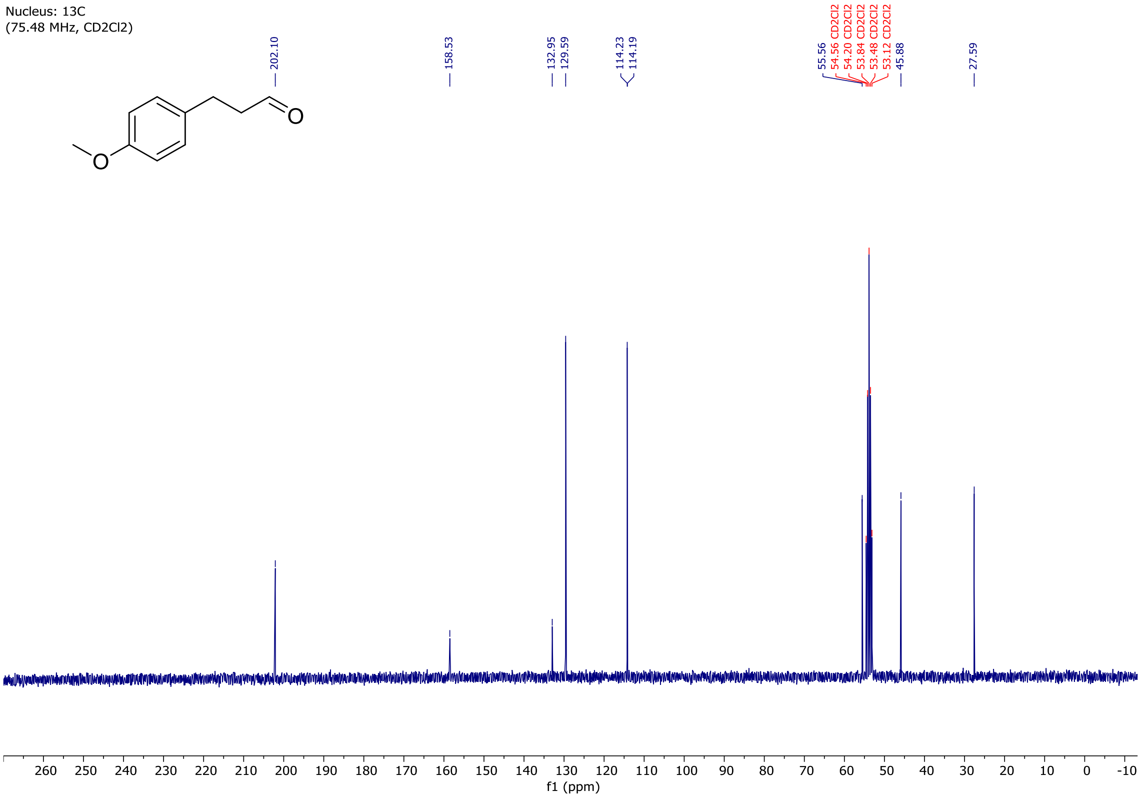

^{1}H NMR and ^{13}C NMR spectrum of compound **27c**

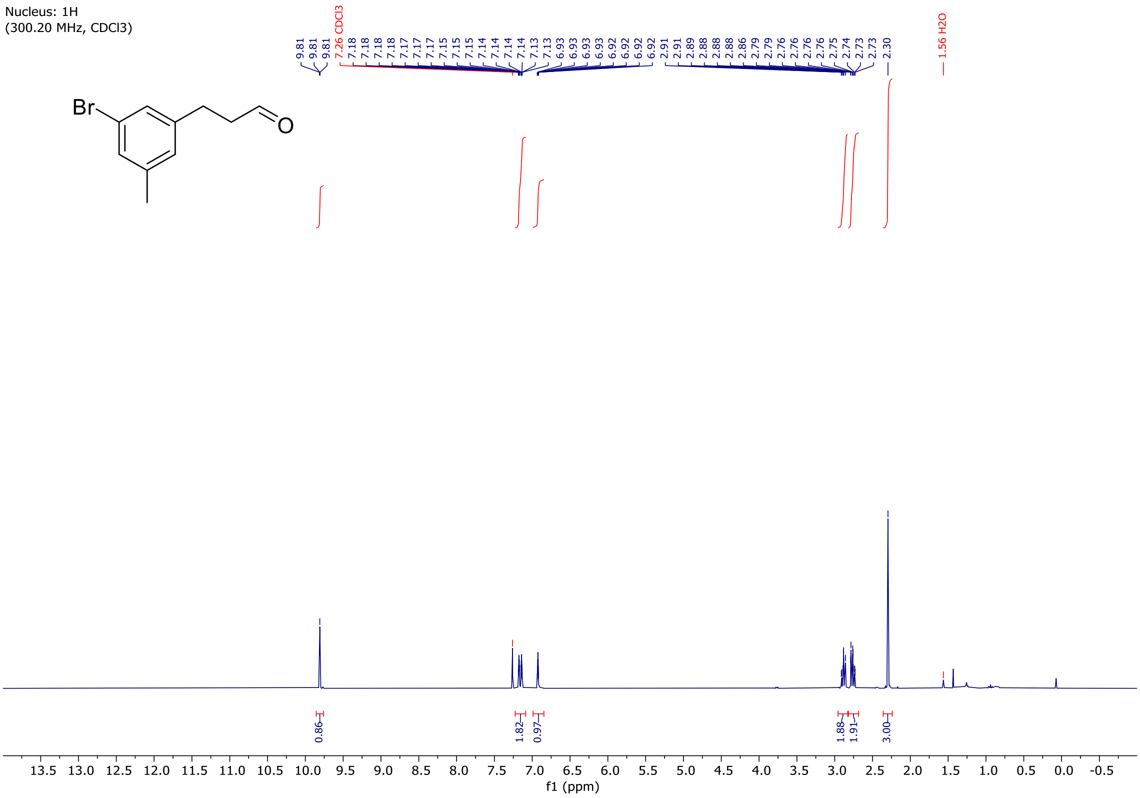

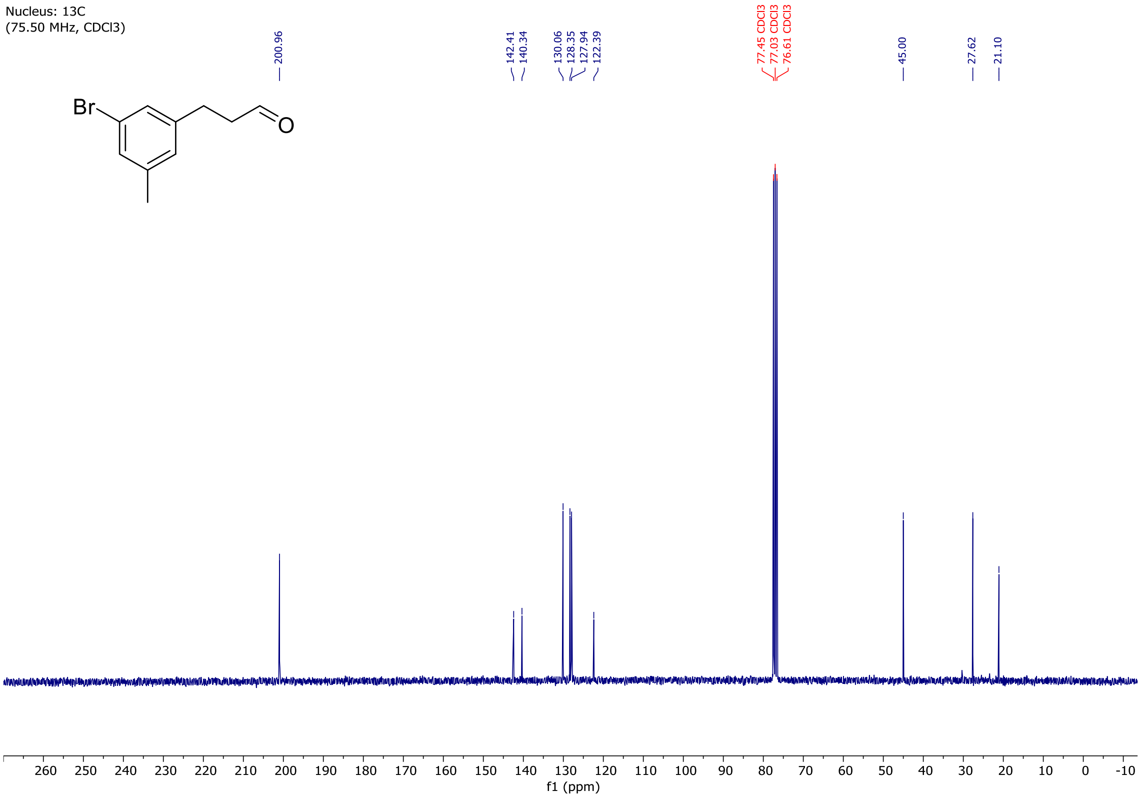

^{1}H NMR and ^{13}C NMR spectrum of compound **29c**

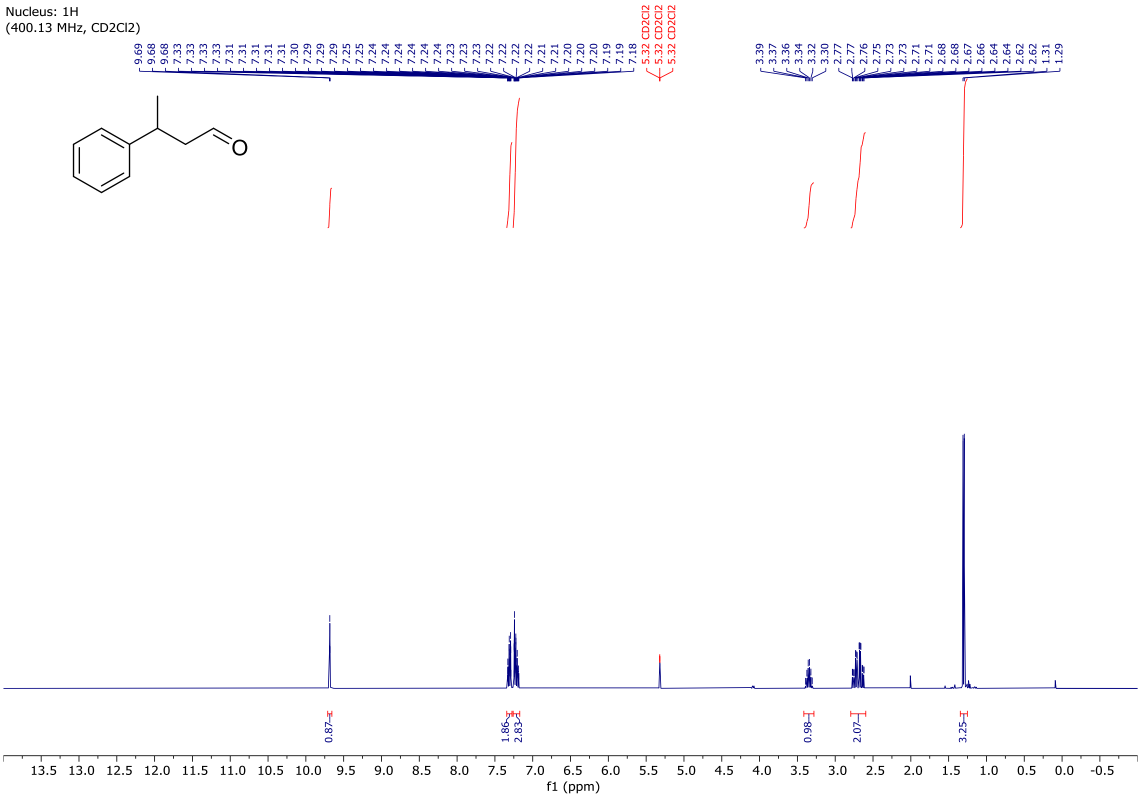

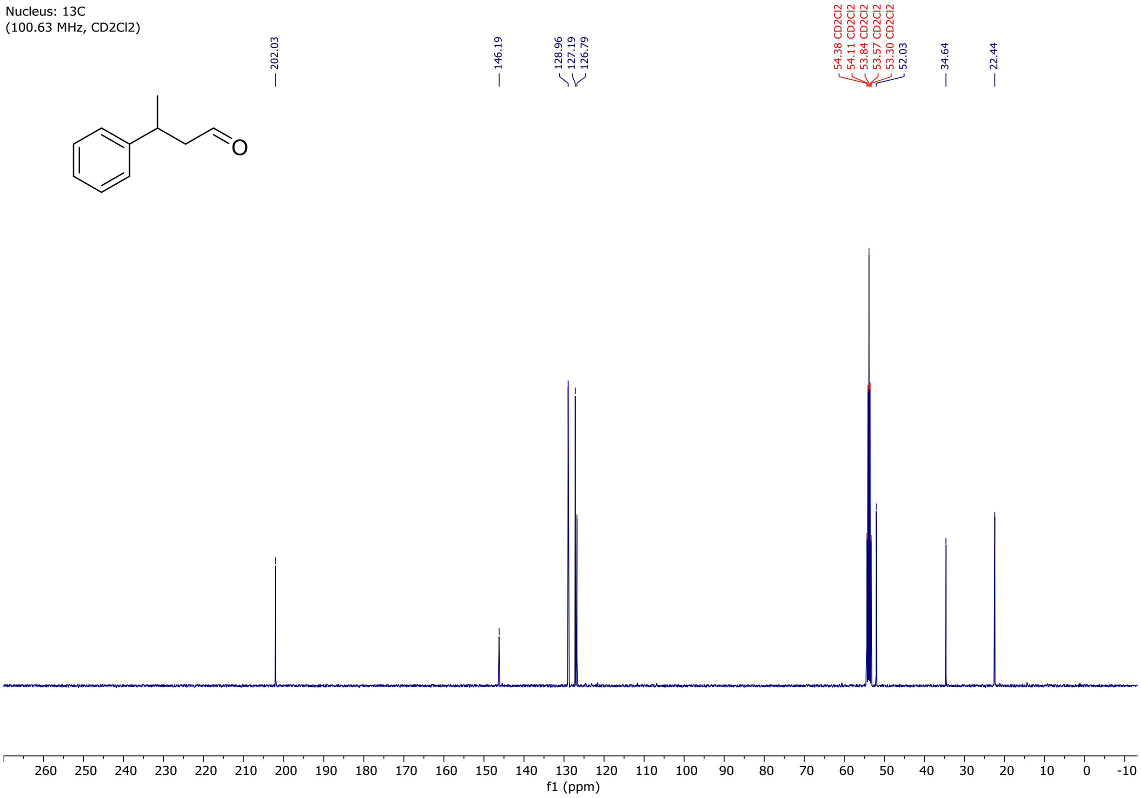

^{1}H NMR, ^{13}C NMR and 19F spectrum of compound **30c**

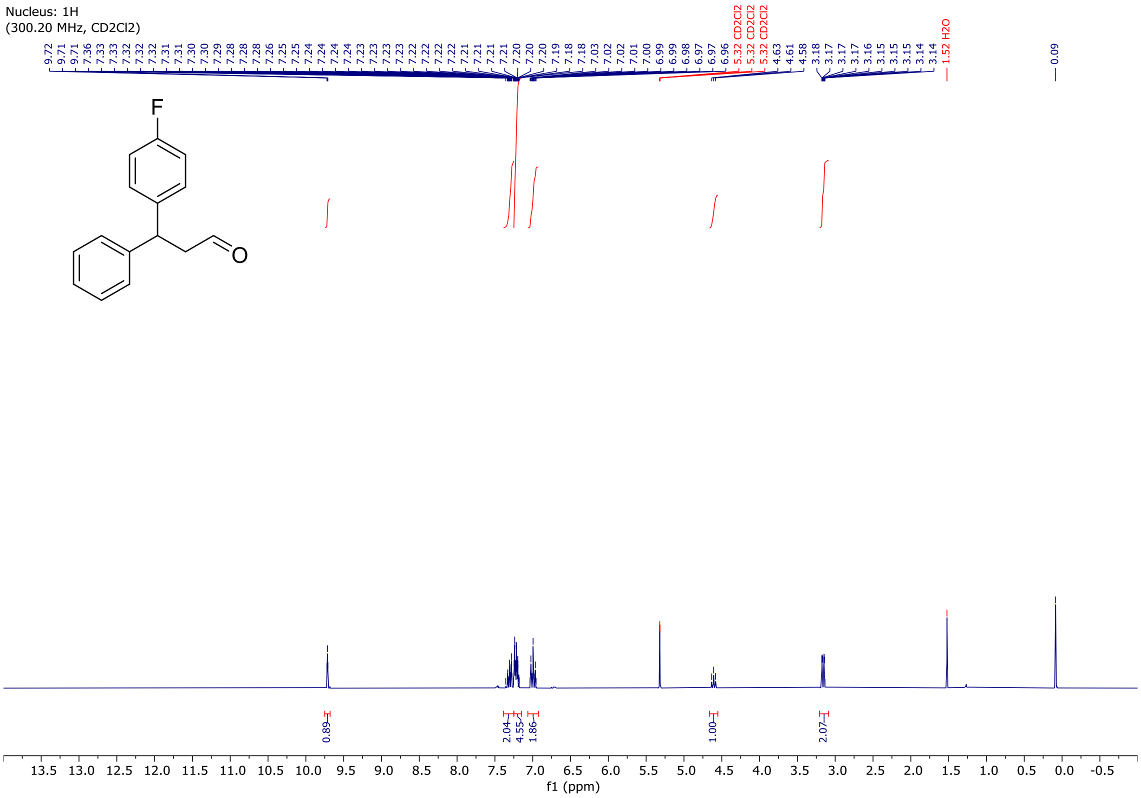

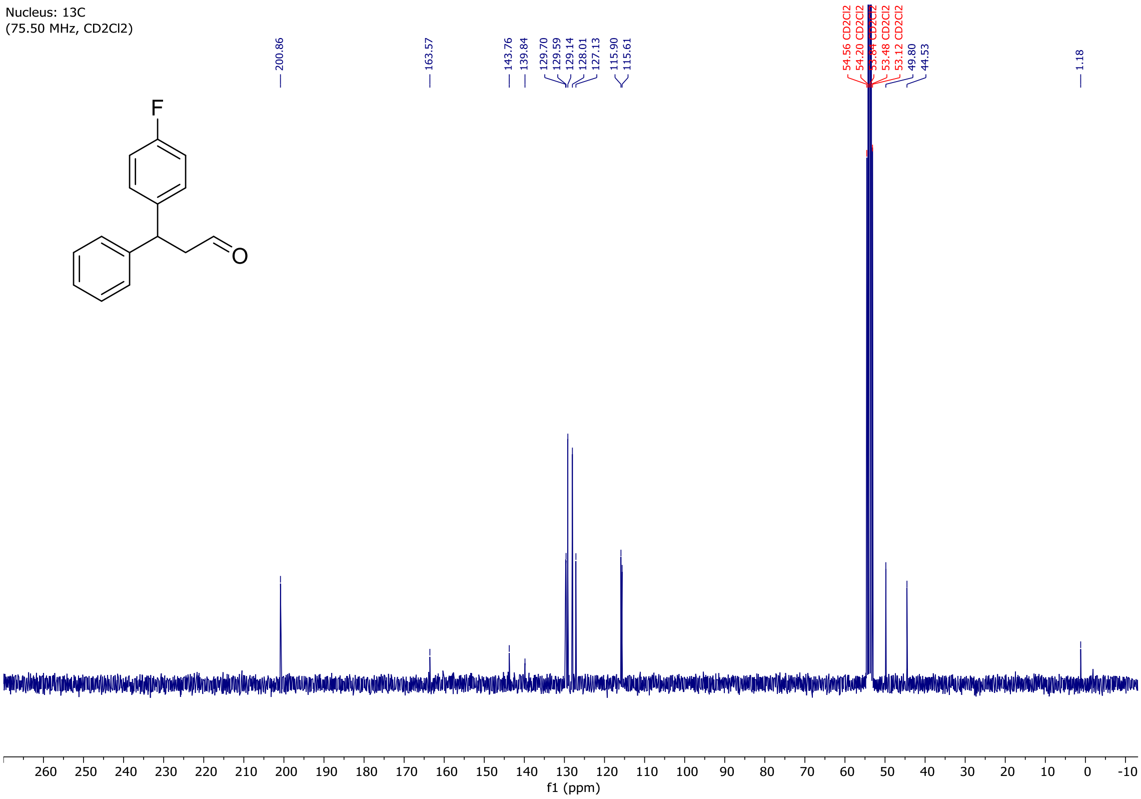

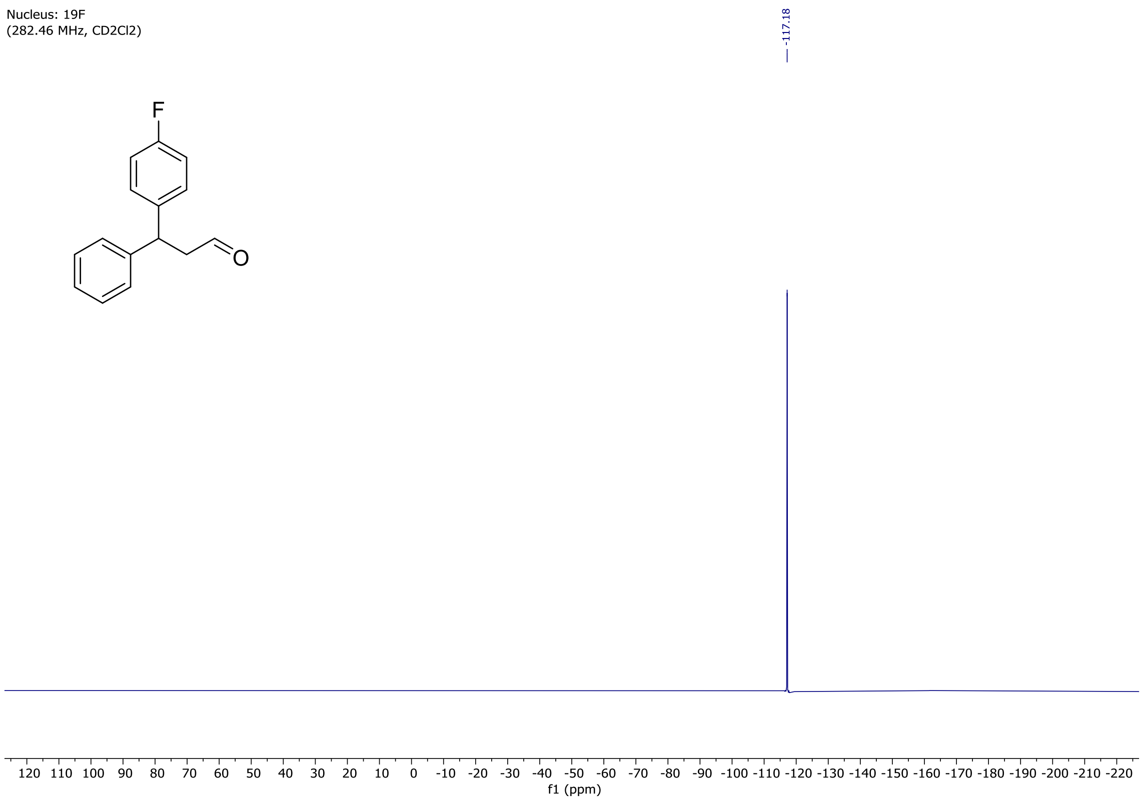
Nucleus: 19F
(282.46 MHz, CD2Cl2)
-117.18
F
O
120 110 100 90 80 70 60 50 40 30 20 10 0 -10 -20 -30 -40 -50 -60 -70 -80 -90 -100 -110 -120 -130 -140 -150 -160 -170 -180 -190 -200 -210 -220
f1 (ppm)

^{1}H NMR and ^{13}C NMR spectrum of compound **31c**

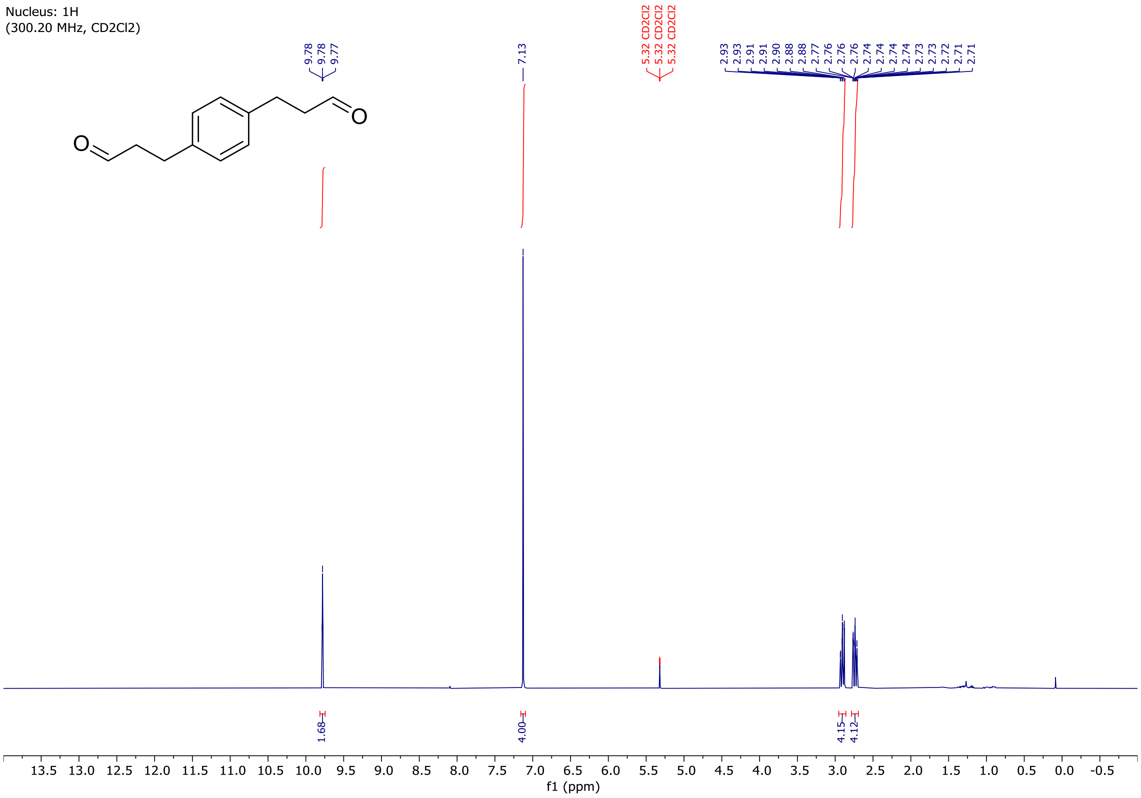

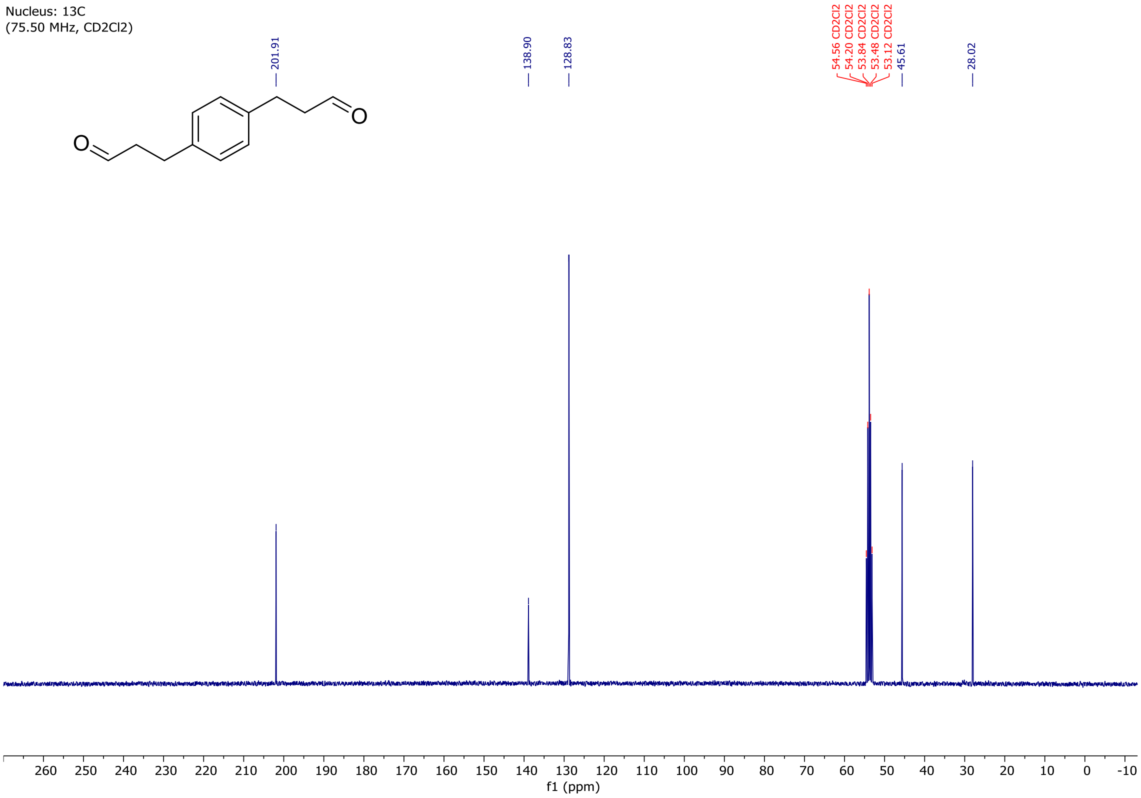

14 IR Spectra

IR spectrum (ATR) of compound **L8**

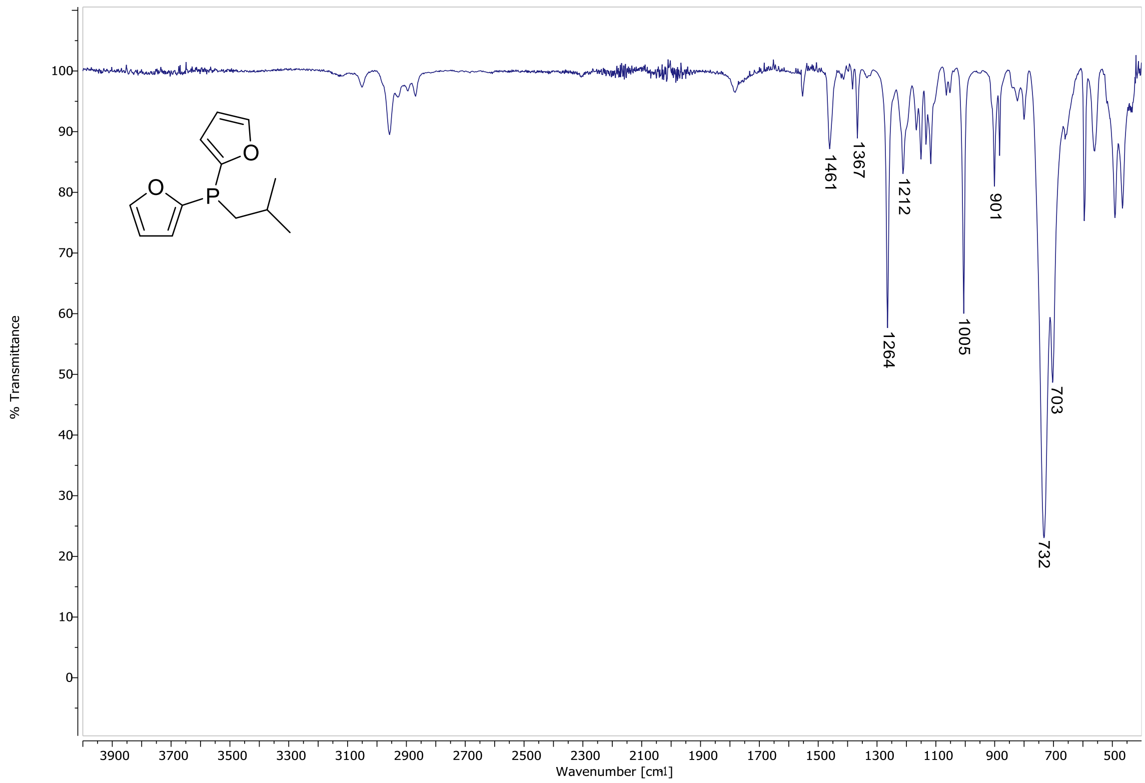

IR spectrum (ATR) of compound **L9**

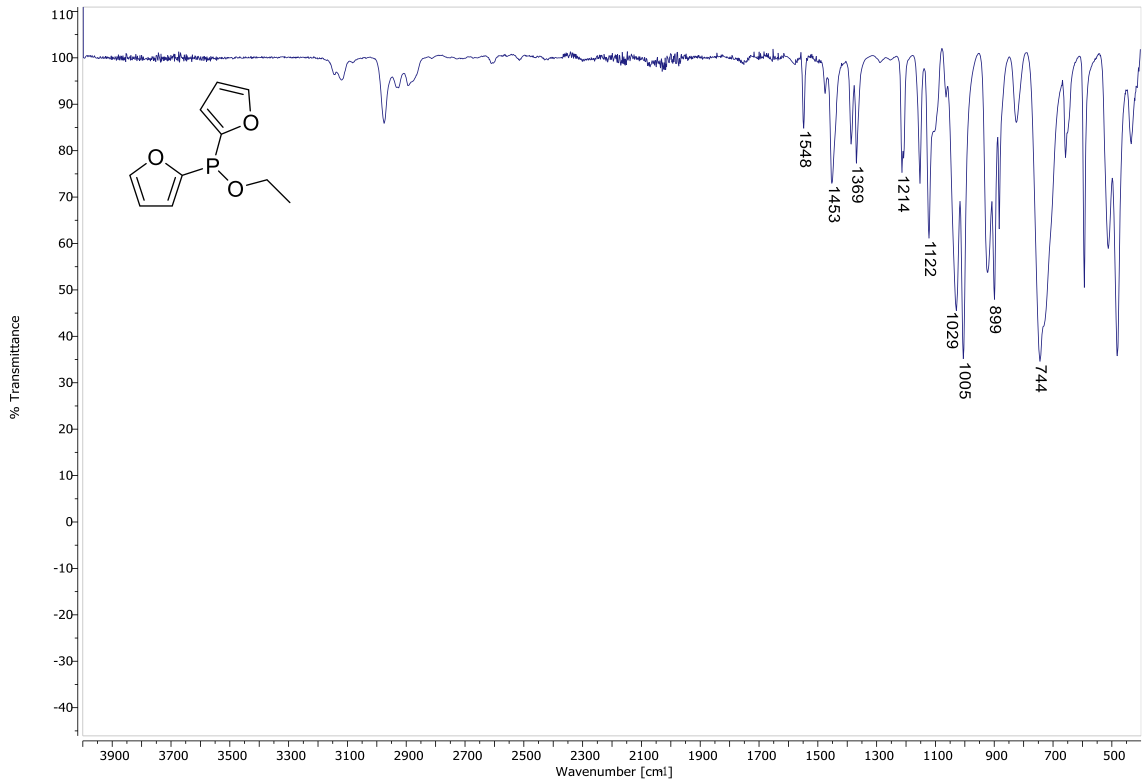

IR spectrum (ATR) of compound **L12**

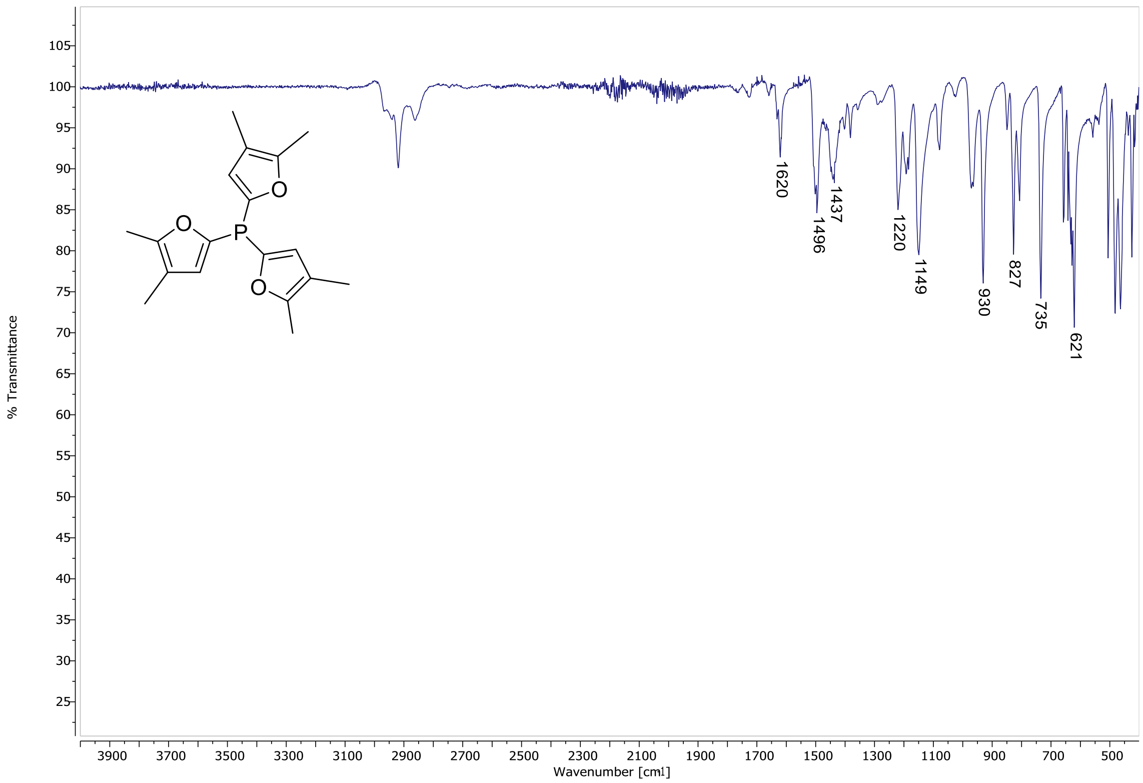

IR spectrum (ATR) of compound **L13**

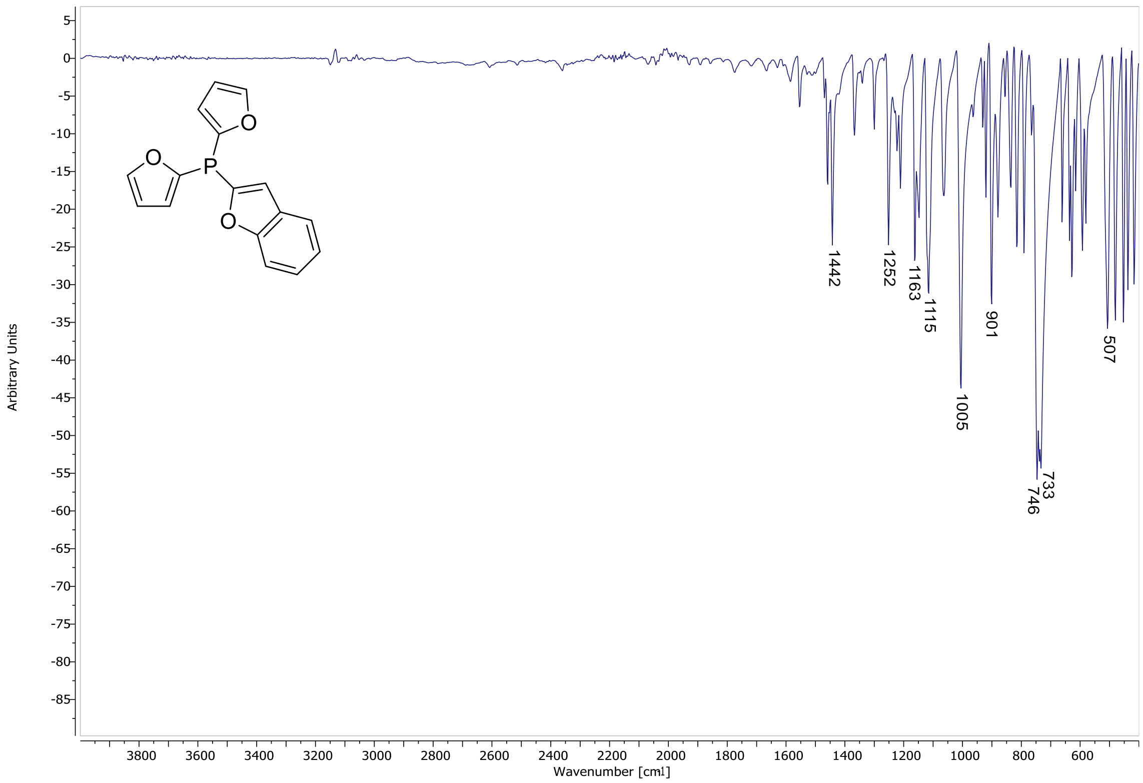

IR spectrum (ATR) of compound **L14**

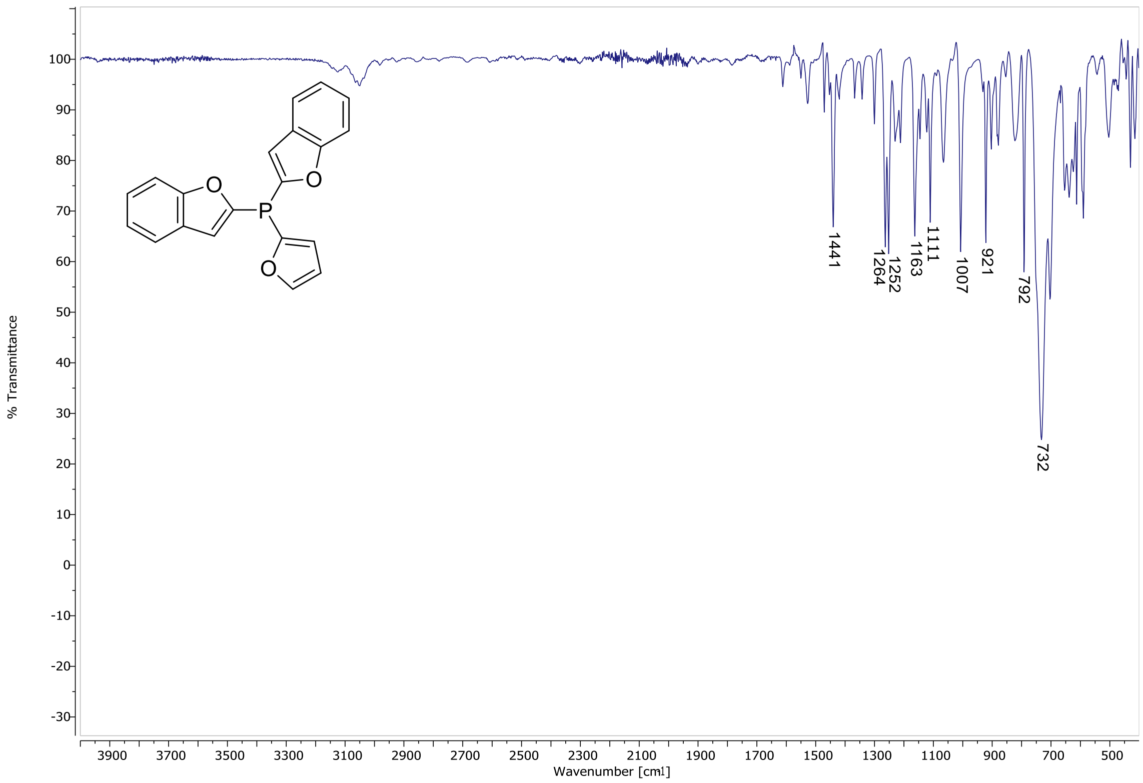

IR spectrum (ATR) of compound **L15**

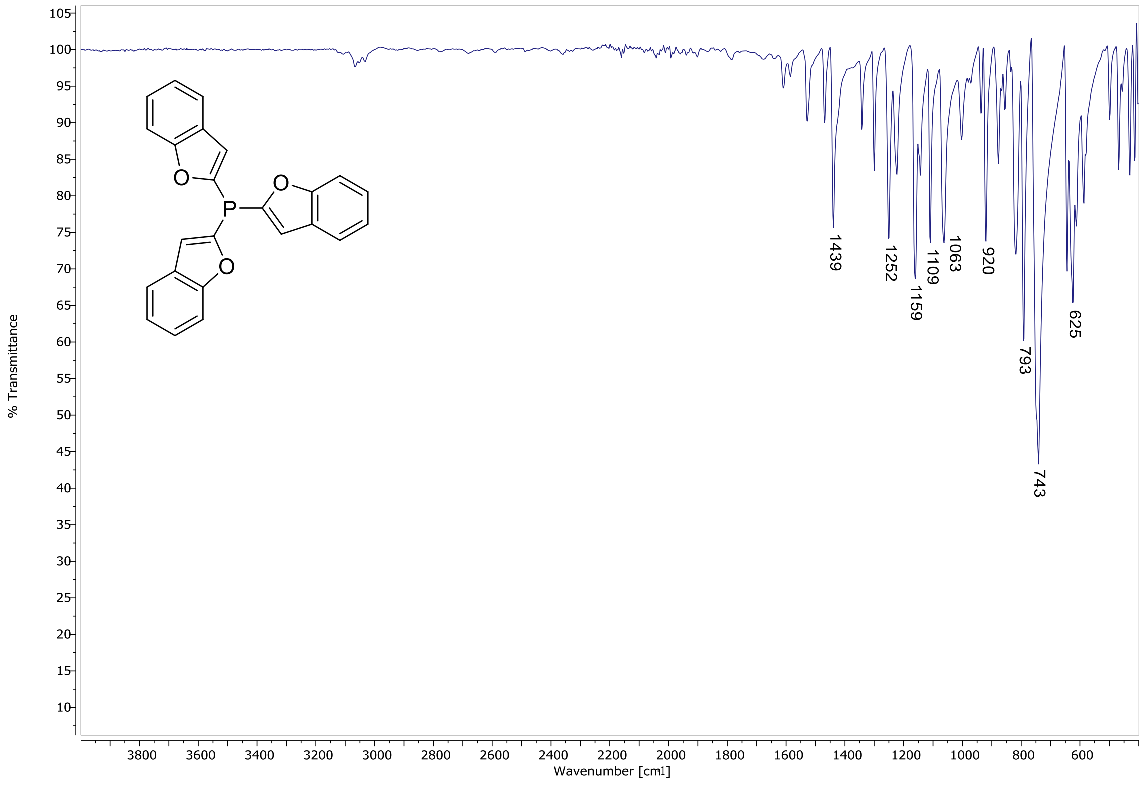

IR spectrum (ATR) of compound **L16**

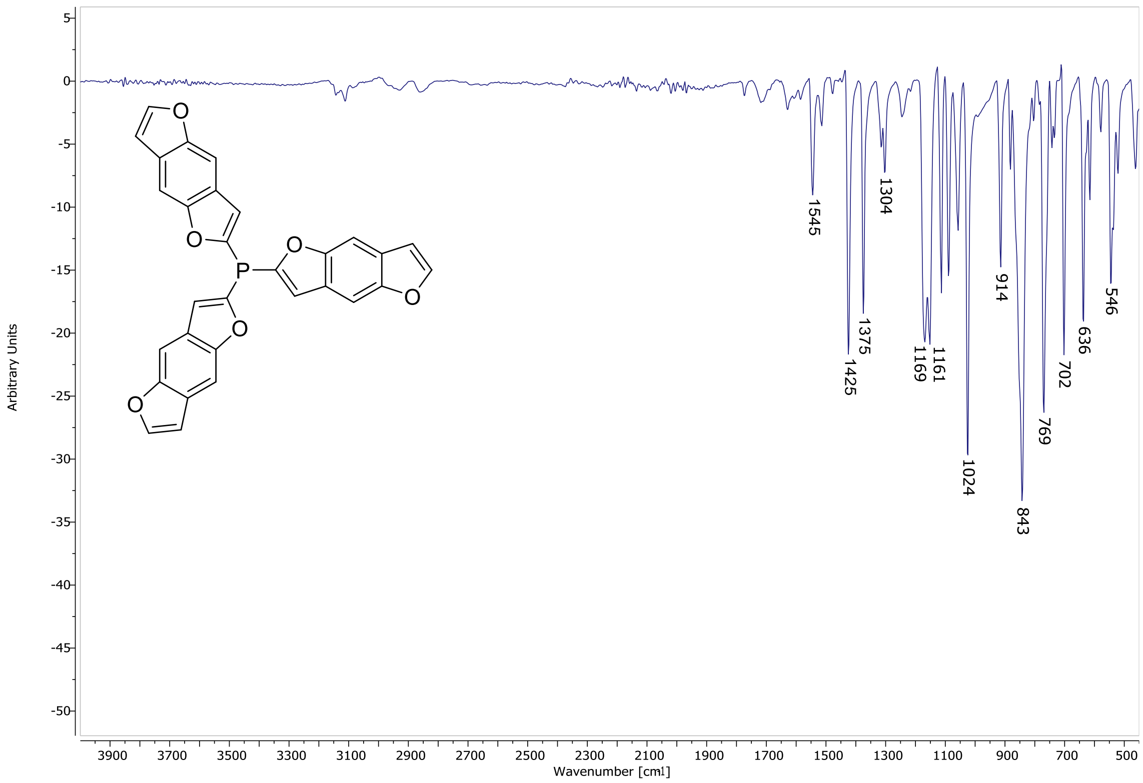

IR spectrum (ATR) of compound **L18**

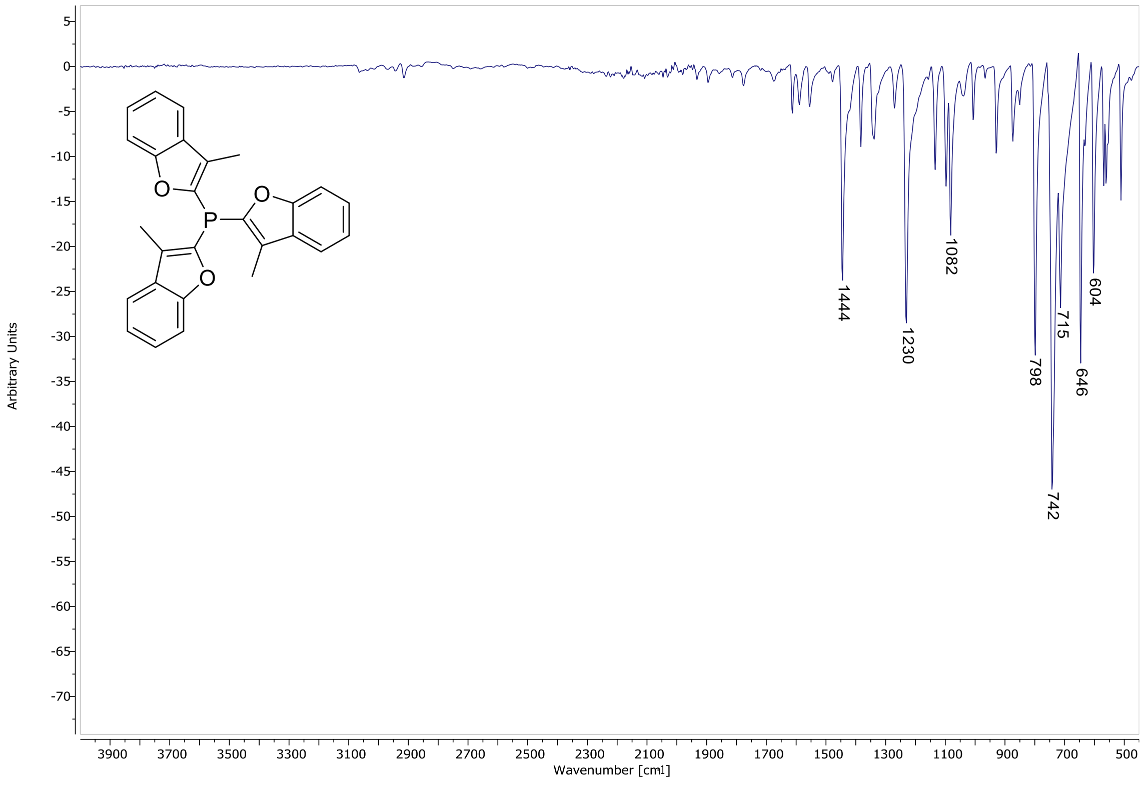

IR spectrum (ATR) of compound **L19**

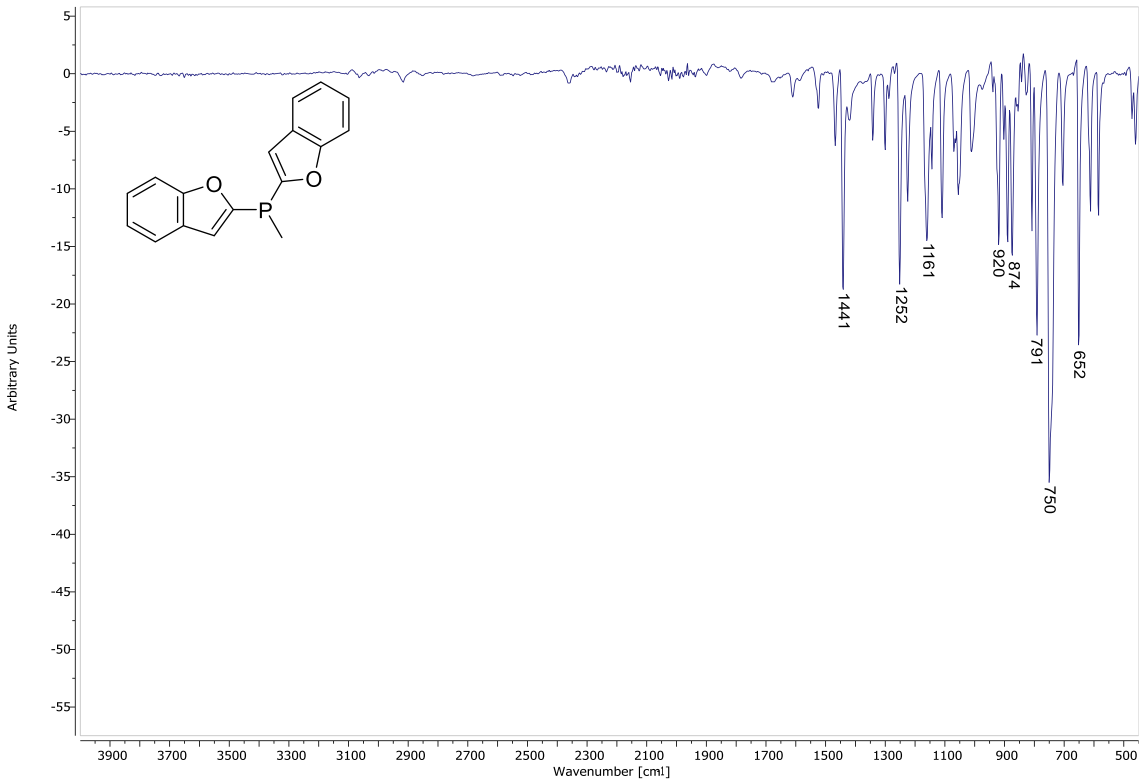

IR spectrum (ATR) of compound **L20**

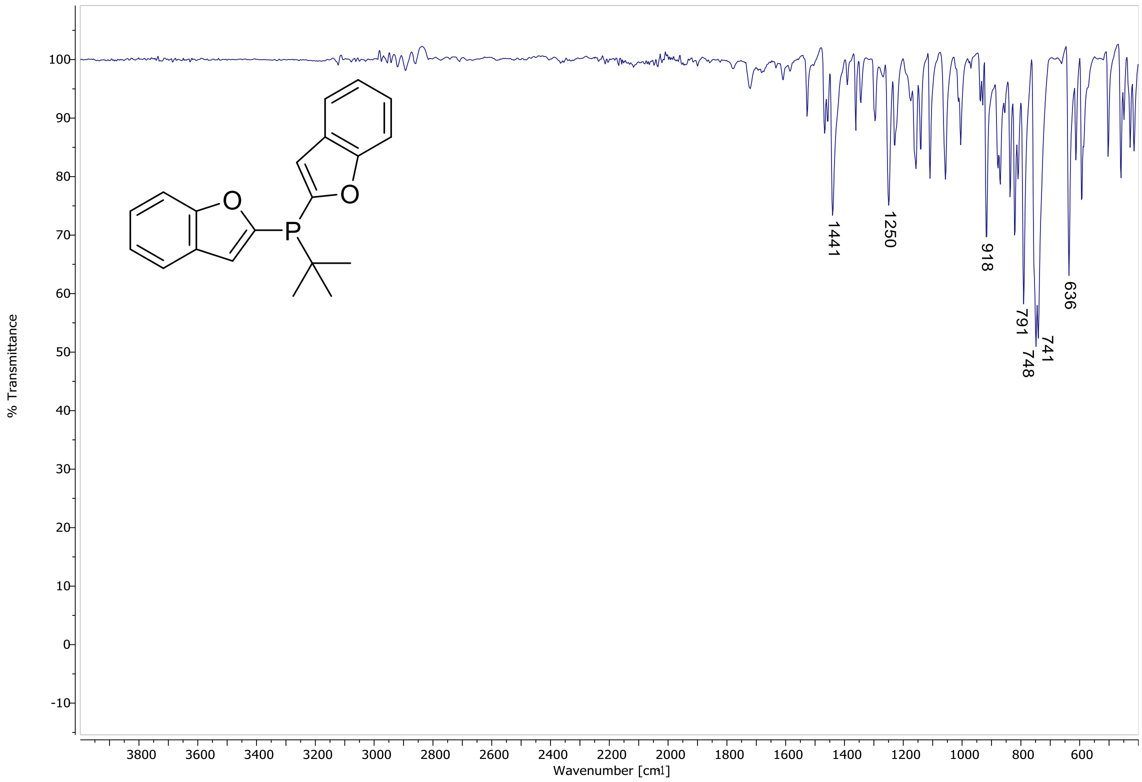

IR spectrum (ATR) of compound **L22**

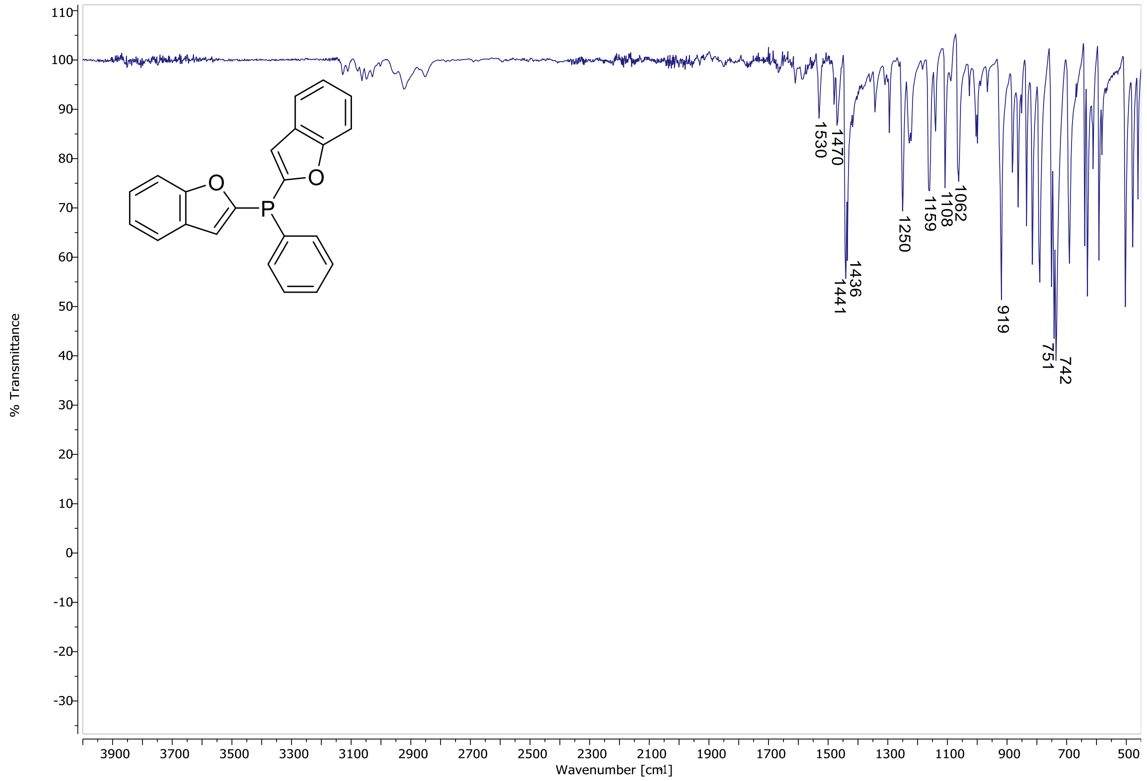

IR spectrum (ATR) of compound **L23**

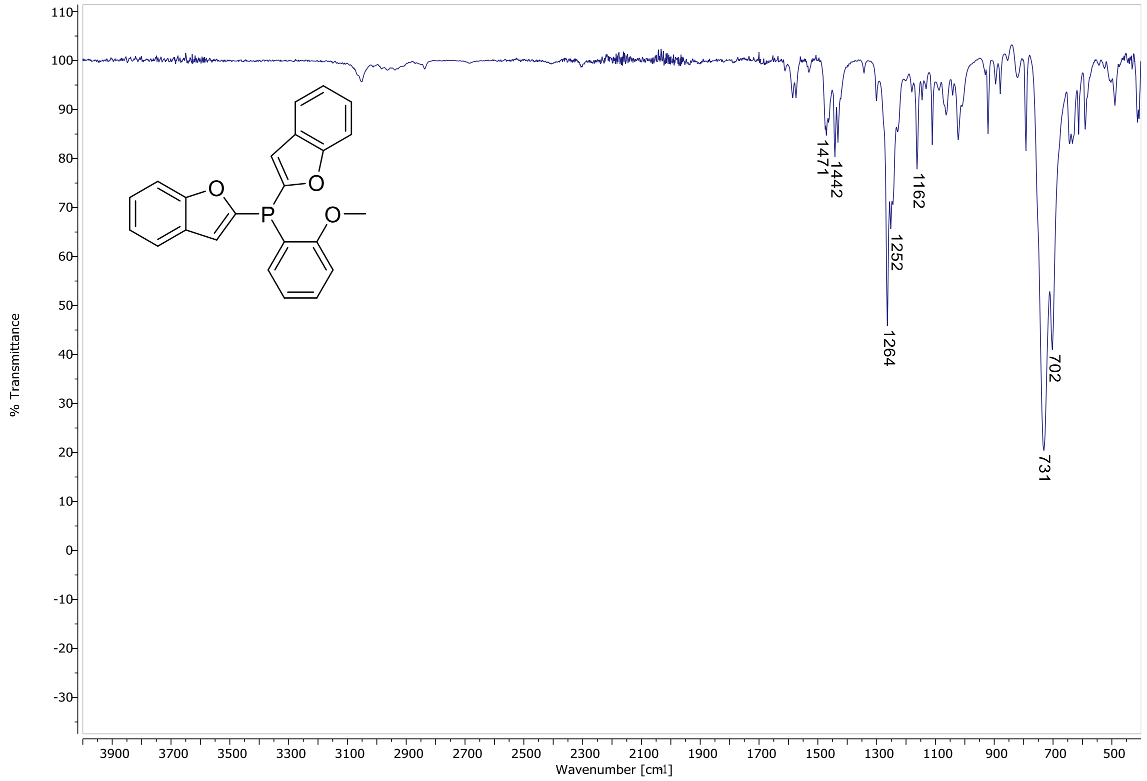

IR spectrum (ATR) of compound **L24**

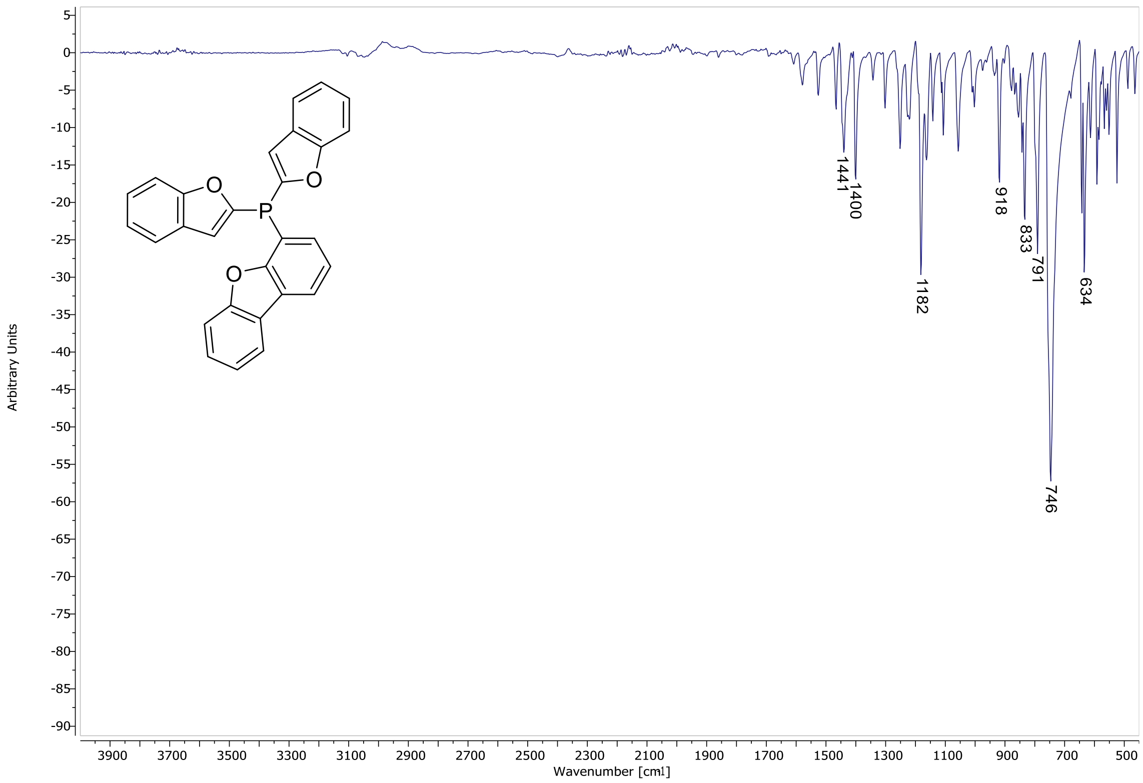

IR spectrum (ATR) of compound **L25**

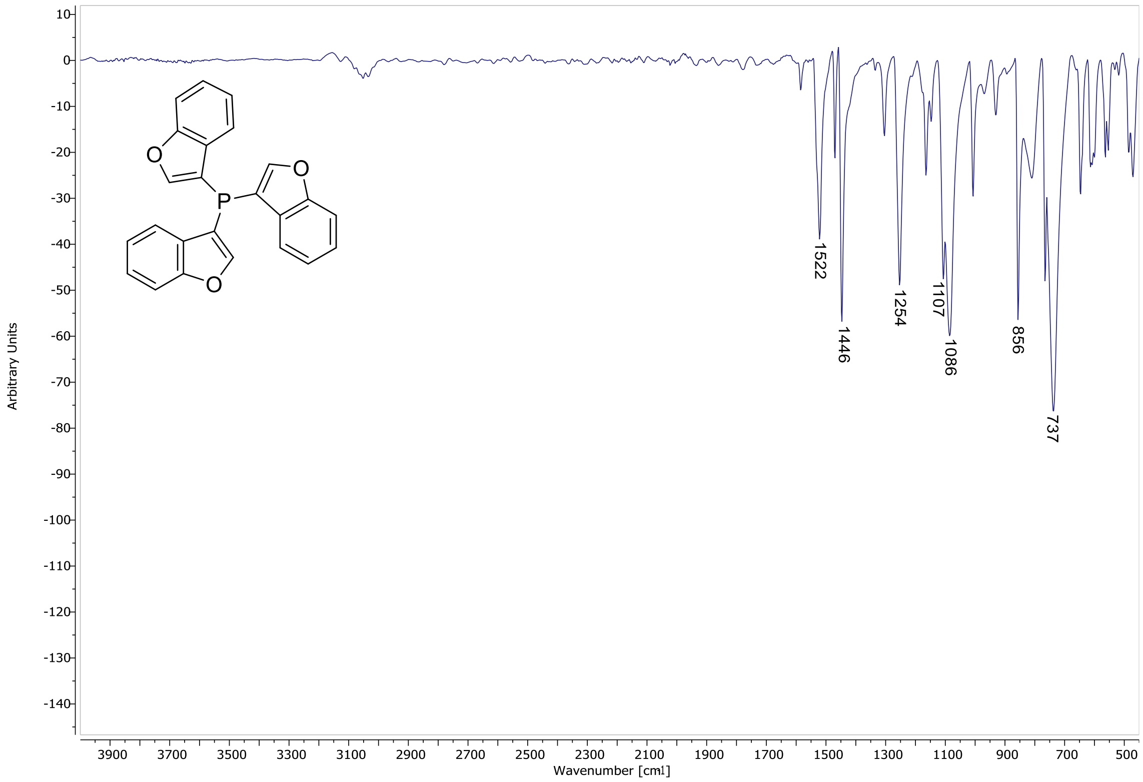

IR spectrum (ATR) of compound **L26**

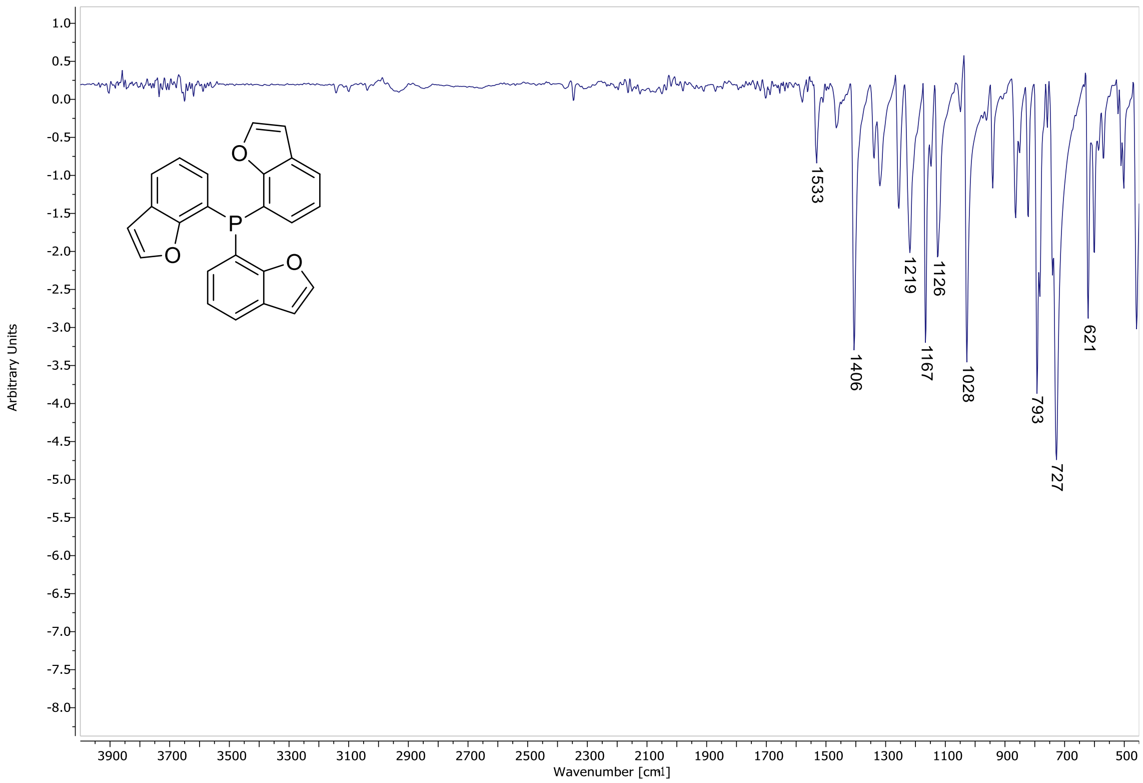

IR spectrum (ATR) of compound **L27**

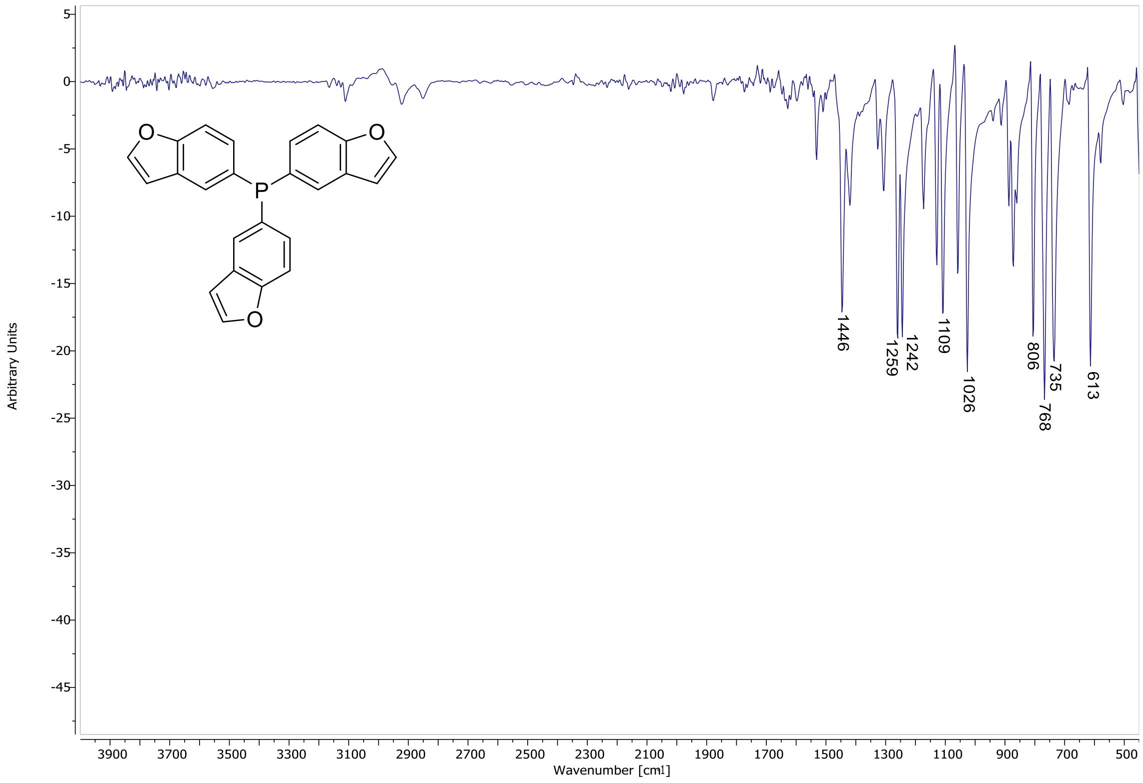

15 HRMS

Di(furan-2-yl)(isobutyl)phosphine (L8)

Formula	Expected Mass	Observed Mass	Error PPM	Error mDa
$C_{12}H_{15}O_2P$	223.0888	223.0884	-1.8	-0.4

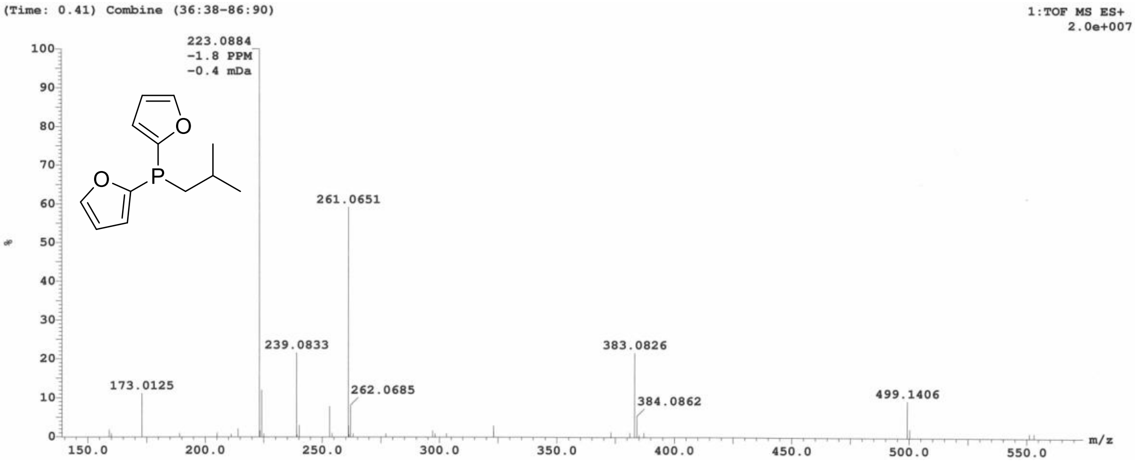

Figure S47: HRMS (ESI) of compound **L8**.

Ethoxydi(furan-2-yl)phosphine (L9)

Formula	Expected Mass	Observed Mass	Error PPM	Error mDa
$C_{10}H_{11}O_3P$	211.0519	211.0522	1.4	0.3

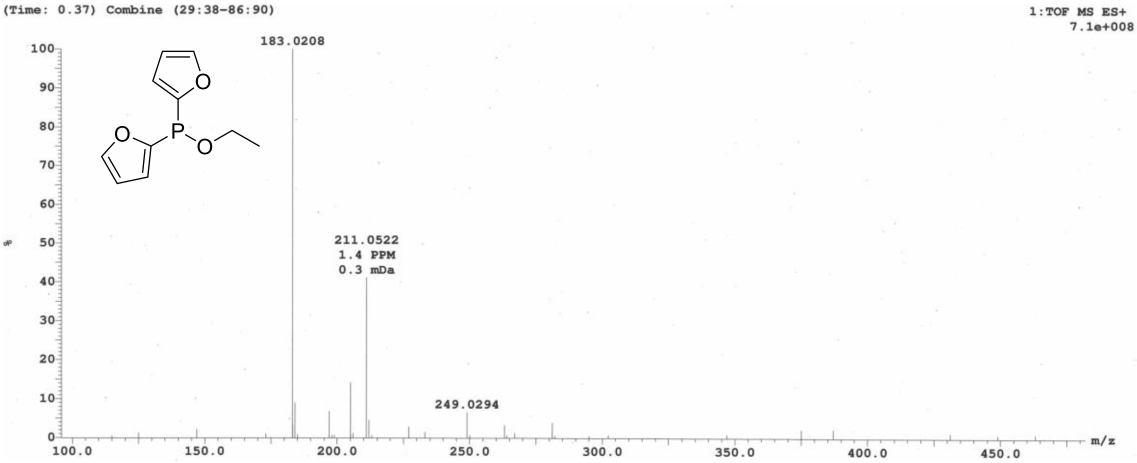

Figure S48: HRMS (ESI) of compound **L9**.

Tris(4,5-dimethylfuran-2-yl)phosphine (L12)

Formula	Expected Mass	Observed Mass	Error PPM	Error mDa
$C_{18}H_{21}O_3P$	317.1306	317.1306	0.0	0.0

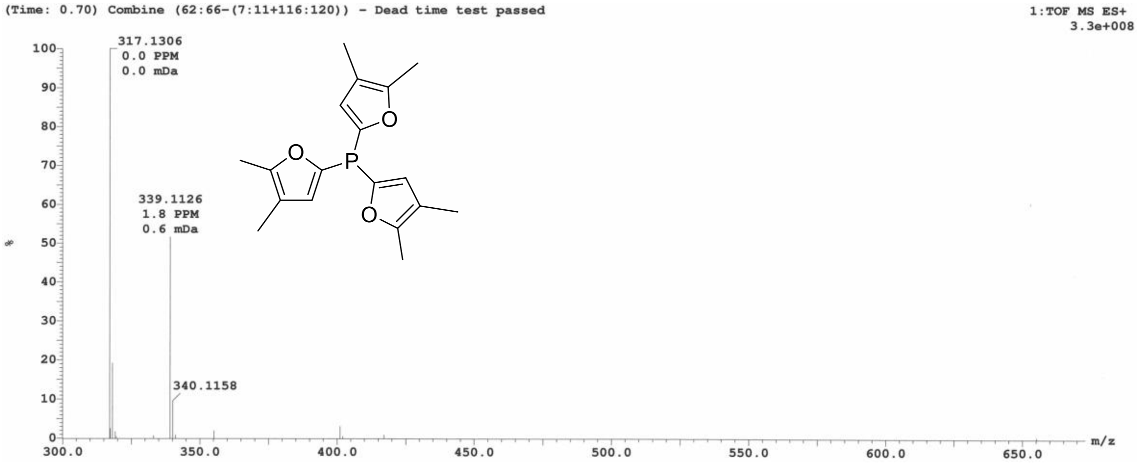

Figure S49: HRMS (ESI) of compound **L12**.

Benzofuran-2-yldi(furan-2-yl)phosphine (L13)

Formula	Expected Mass	Observed Mass	Error PPM	Error mDa
$C_{16}H_{11}O_3P$	283.0524	283.0516	-2.8	-0.8

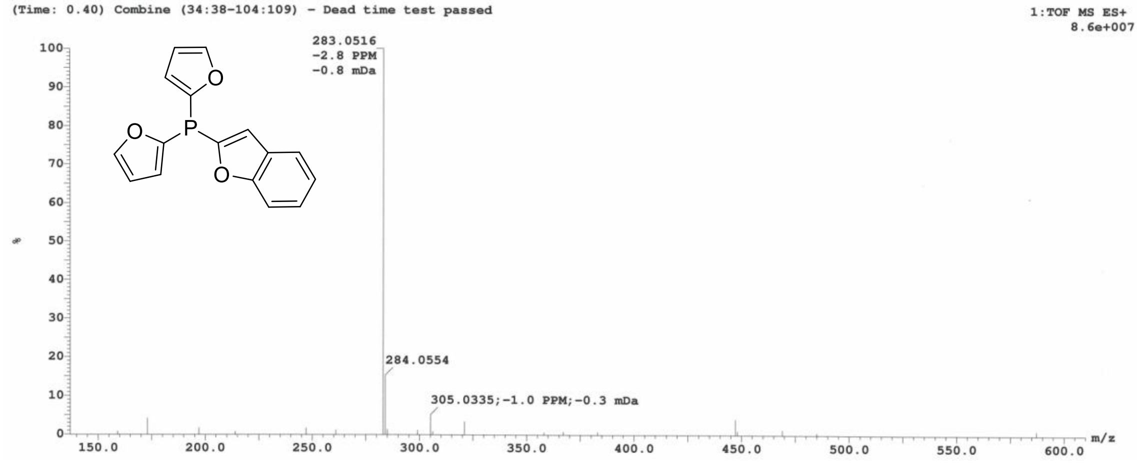

Figure S50: HRMS (ESI) of compound **L13**.

Di(benzofuran-2-yl)(furan-2-yl)phosphine (L14)

Formula	Expected Mass	Observed Mass	Error PPM	Error mDa
$C_{20}H_{13}O_3P$	355.0494	355.0500	1.7	0.6

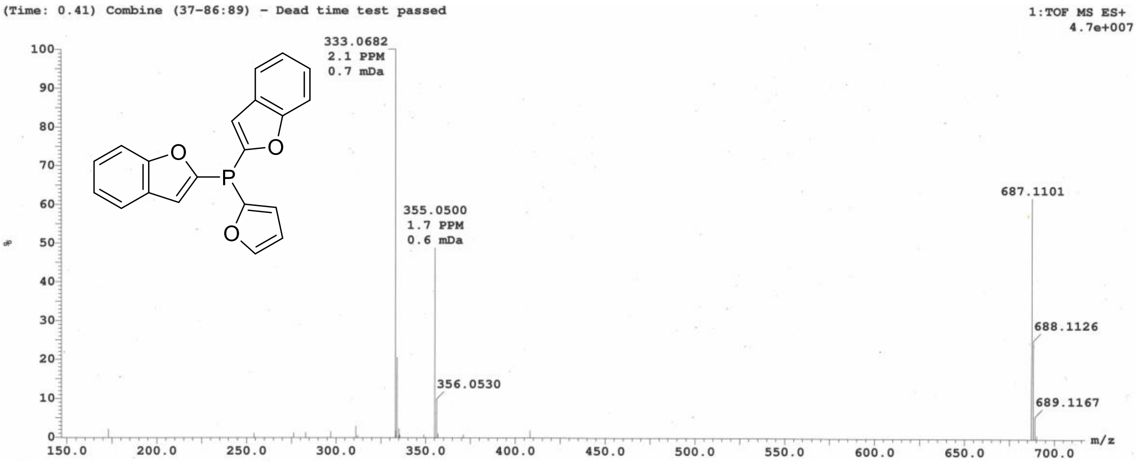

Figure S51: HRMS (ESI) of compound **L14**.

Tris(benzo[1,2-*b*:4,5-*b'*]difuran-2-yl)phosphine (L16)

Formula	Expected Mass	Observed Mass	Error PPM	Error mDa
$C_{30}H_{15}O_6P$	503.0685	503.0684	-0.2	-0.1

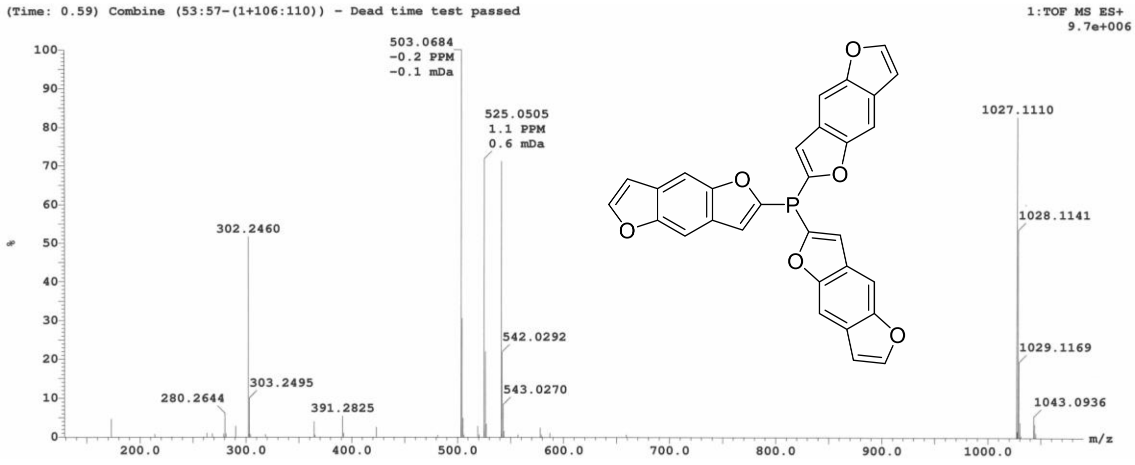

Figure S52: HRMS (ESI) of compound **L16**.

Tris(3-methylbenzofuran-2-yl)phosphine (L18)

Formula	Expected Mass	Observed Mass	Error PPM	Error mDa
$C_{27}H_{21}O_3P$	447.1120	447.1119	-0.2	-0.1

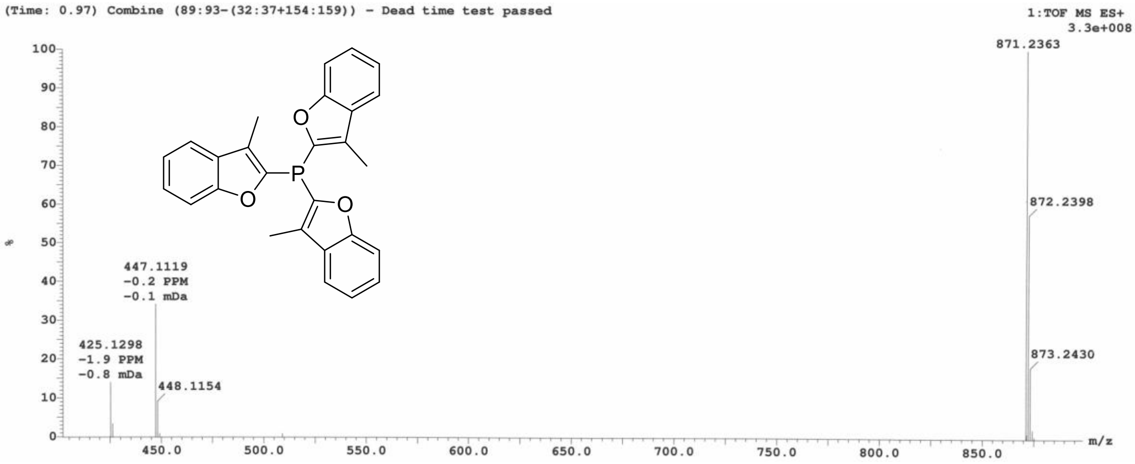

Figure S53: HRMS (ESI) of compound **L18**.

Di(benzofuran-2-yl)(methyl)phosphine (L19)

Formula	Expected Mass	Observed Mass	Error PPM	Error mDa
$C_{17}H_{13}O_2P$	281.0731	281.0726	-1.8	-0.5

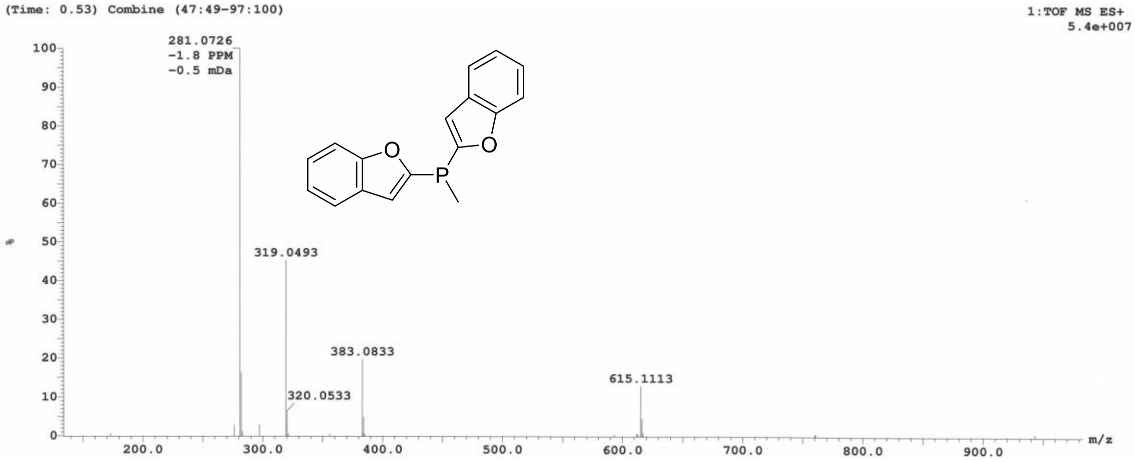

Figure S54: HRMS (ESI) of compound **L19**.

Di(benzofuran-2-yl)(1,3-dioxolan-2-yl)phosphine (L21)

Formula	Expected Mass	Observed Mass	Error PPM	Error mDa
$C_{19}H_{15}O_4P$	361.0600	361.0608	2.2	0.8

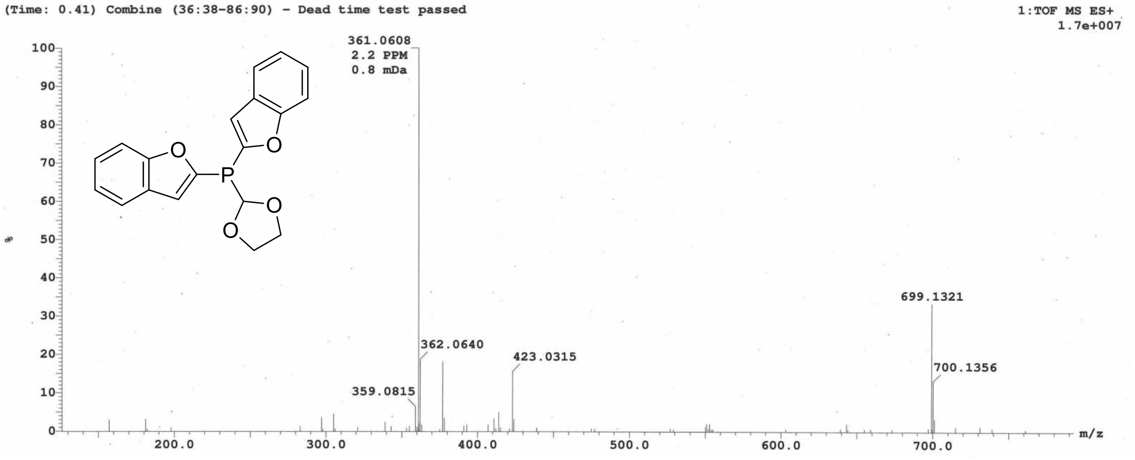

Figure S55: HRMS (ESI) of compound **L21**.

Di(benzofuran-2-yl)(phenyl)phosphine (L22)

Formula	Expected Mass	Observed Mass	Error PPM	Error mDa
$C_{22}H_{15}O_2P$	343.0888	343.0888	0.0	0.0

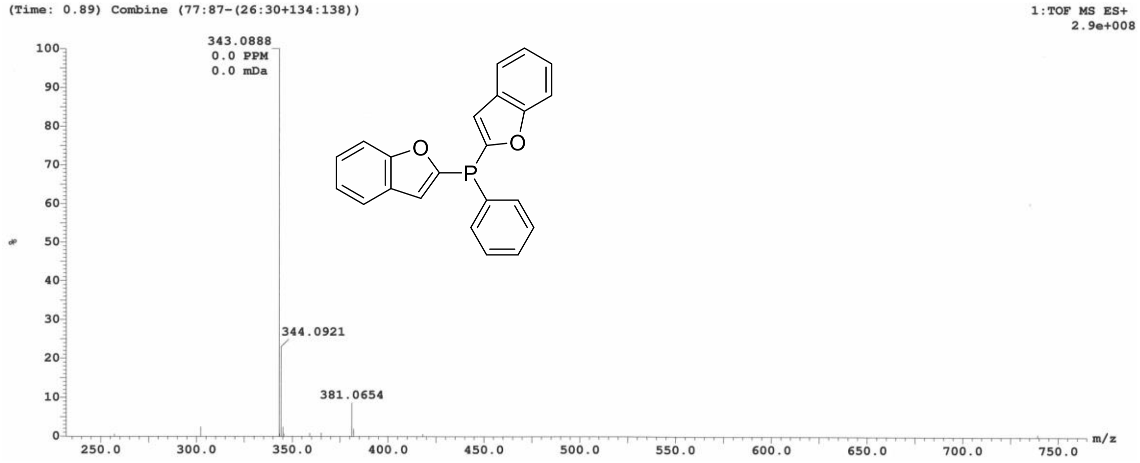

Figure S56: HRMS (ESI) of compound **L22**.

Di(benzofuran-2-yl)(2-methoxyphenyl)phosphine (L23)

Formula	Expected Mass	Observed Mass	Error PPM	Error mDa
$C_{23}H_{17}O_3P$	373.0993	373.0992	-0.3	-0.1

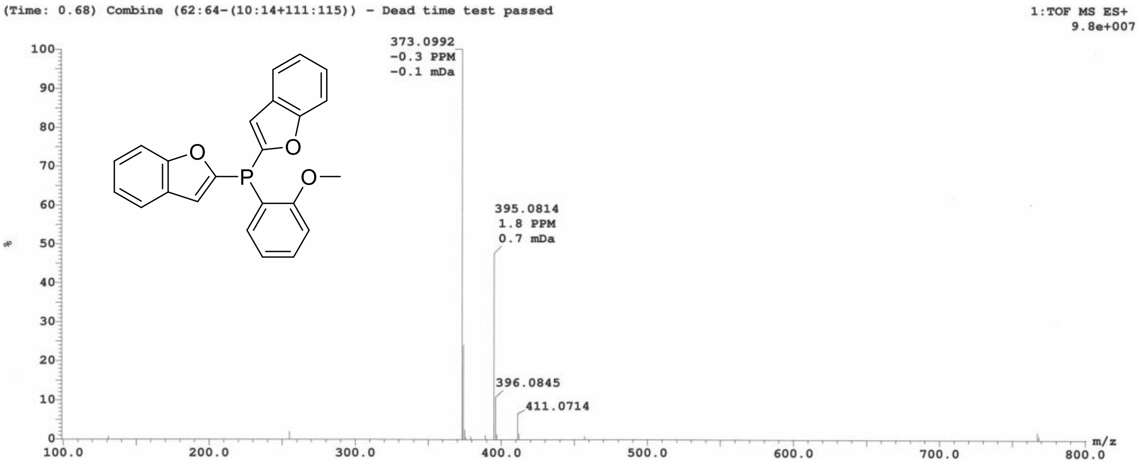

Figure S57: HRMS (ESI) of compound **L23**.

Di(benzofuran-2-yl)(dibenzo[*b,d*]furan-4-yl)phosphine (L24)

Formula	Expected Mass	Observed Mass	Error PPM	Error mDa
$C_{28}H_{17}O_3P$	455.0807	455.0808	0.2	0.1

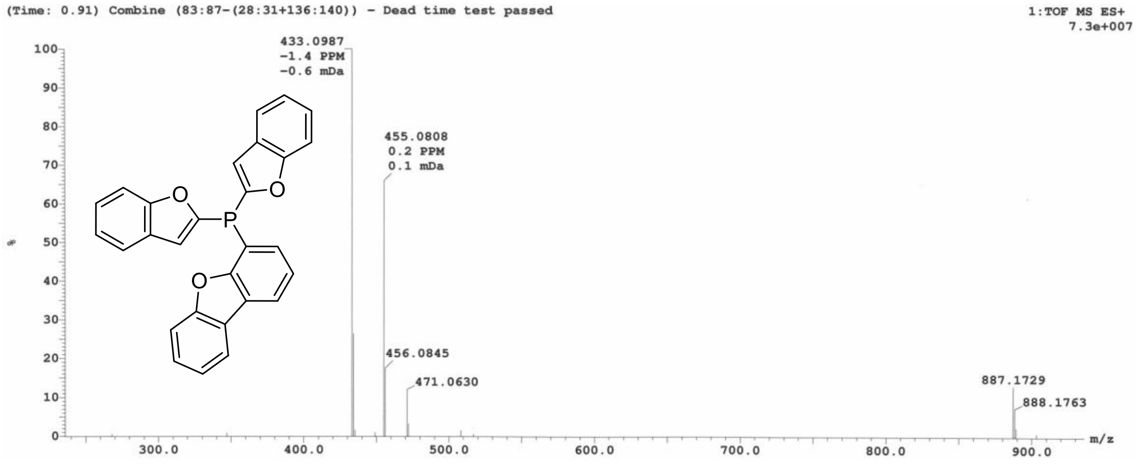

Figure S58: HRMS (ESI) of compound **L24**.

Tri(benzofuran-3-yl)phosphine (L25)

Formula	Expected Mass	Observed Mass	Error PPM	Error mDa
$C_{24}H_{15}O_3P$	383.0837	383.0828	-2.3	-0.9

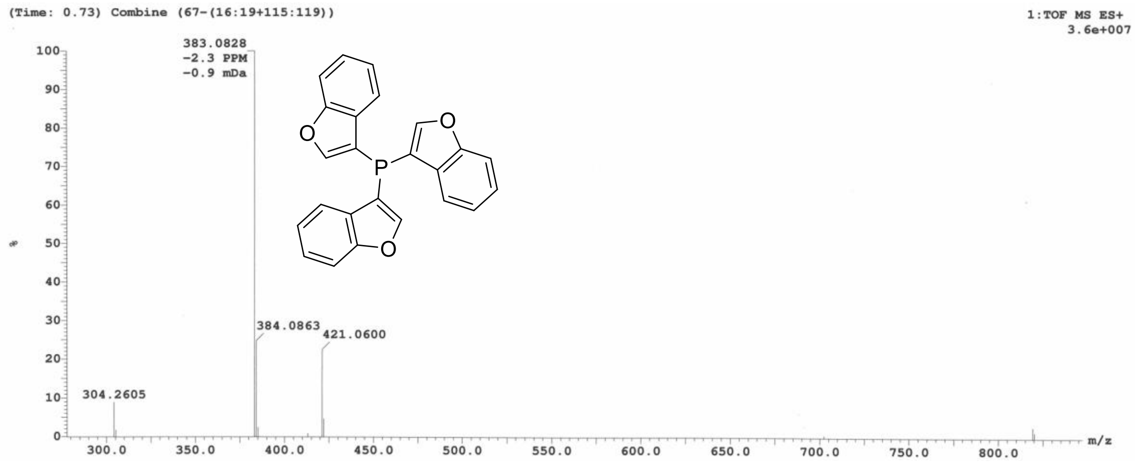

Figure S59: HRMS (ESI) of compound **L25**.

Tri(benzofuran-7-yl)phosphine (L26)

Formula	Expected Mass	Observed Mass	Error PPM	Error mDa
$C_{24}H_{15}O_3P$	405.0651	405.0649	-0.5	-0.2

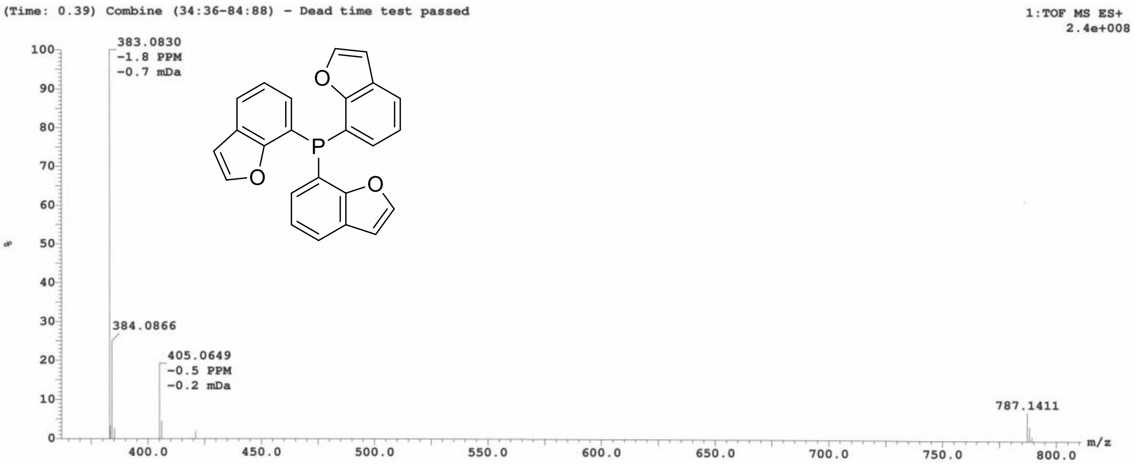

Figure S60: HRMS (ESI) of compound **L26**.

Tri(benzofuran-5-yl)phosphine (L27)

Formula	Expected Mass	Observed Mass	Error PPM	Error mDa
$C_{24}H_{15}O_3P$	383.0837	383.0836	-0.3	-0.1

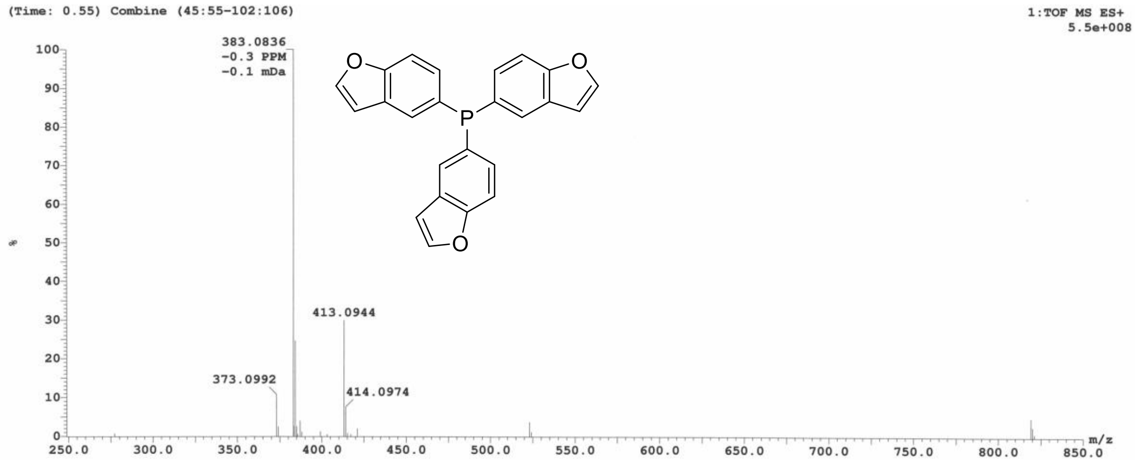

Figure S61: HRMS (ESI) of compound **L27**.

16 References

(1) Tani, K.; Yamagata, T.; Akutagawa, S.; Kumobayashi, H.; Taketomi, T.; Takaya, H.; Miyashita, A.; Noyori, R.; Otsuka, S. Metal-assisted terpenoid synthesis. 7. Highly enantioselective isomerization of prochiral allylamines catalyzed by chiral diphosphine rhodium(I) complexes. Preparation of optically active enamines. *J. Am. Chem. Soc.* **1984**, *106* (18), 5208-5217.

(2) Junge, K.; Wendt, B.; Cingolani, A.; Spannenberg, A.; Wei, Z.; Jiao, H.; Beller, M. Cobalt Pincer Complexes for Catalytic Reduction of Carboxylic Acid Esters. *Chem. Eur. J.* **2018**, *24* (5), 1046-1052. Liu, X.; Zhang, W.; Wang, Y.; Zhang, Z. X.; Jiao, L.; Liu, Q. Cobalt-Catalyzed Regioselective Olefin Isomerization Under Kinetic Control. *J. Am. Chem. Soc.* **2018**, *140* (22), 6873-6882.

(3) TOLMACHEV, A. A.; IVONIN, S. P.; KHARCHENKO, A. V.; KOZLOV, E. S. ChemInform Abstract: Phosphorylation of Furan, 2-Methylfuran, and Thiophene with Phosphorus Tribromide. *ChemInform* **1992**, *23* (51).

(4) Jian, Z.; Wucher, P.; Mecking, S. Heterocycle-Substituted Phosphinesulfonato Palladium(II) Complexes for Insertion Copolymerization of Methyl Acrylate. *Organometallics* **2014**, *33* (11), 2879-2888.

(5) Märkl, G.; Amrhein, J.; Stoiber, T.; Striebl, U.; Kreitmeier, P. 5,16-Dialkyl(diaryl)-5,16-dihydro-5,16-diphospha-tetraepoxy[22]annulene(2.1.2.1). *Tetrahedron* **2002**, *58* (13), 2551-2567.

(6) Mongin, F.; Bucher, A.; Bazureau, J. P.; Bayh, O.; Awad, H.; Trécourt, F. Deprotonation of furans using lithium magnesates. *Tetrahedron Lett.* **2005**, *46* (46), 7989-7992.

(7) Lindner, E.; Rauleder, H.; Scheytt, C.; Mayer, H. A.; Hiller, W.; Fawzi, R.; Wegner, P. Neuartige basische Liganden für die homogenkatalytische Homologisierung von Methanol zu Ethanol, II [1]. Synthese und komplexchemisches Verhalten potentiell zwei- und dreizähniger P–N- und P–O-haltiger Chelatliganden / Novel Basic Ligands for the Homogeneous Catalytic Homologation of Methanol to Ethanol, II [1]. Synthesis and Complex Chemical Behaviour of Potential Bi- and Tridentate P–N and P–O Containing Chelate Ligands. *Zeitschrift für Naturforschung B* **1984**, *39* (5), 632-642.

(8) Santelli-Rouvier, C.; Coin, C.; Toupet, L.; Santelli, M. Synthesis of the tertiaryphosphine derivatives of iron carbonyl phosphines: preparation of tetracarbonyl[tris(2-furyl) phosphine]iron(0), pentacarbonylbis[μ-bis(2-furyl)phosphido]-[tris(2-furyl)phosphine] diiron(0), tetracarbonyl[tris(2-benzofuryl) phosphine]iron(0) and pentacarbonylbis[μ-bis(2-benzofuryl) phosphido]-[tris(2-benzofuryl) phosphine]diiron-(0). *J. Organomet. Chem.* **1995**, *495* (1), 91-96.

(9) Kou, K. G. M.; Le, D. N.; Dong, V. M. Rh(I)-Catalyzed Intermolecular Hydroacylation: Enantioselective Cross-Coupling of Aldehydes and Ketoamides. *J. Am. Chem. Soc.* **2014**, *136* (26), 9471-9476.

(10) Shiner, C. S.; Tsunoda, T.; Goodman, B. A.; Ingham, S.; Lee, S. H.; Vorndam, P. E. (Dialkoxymethyl) lithiums: generation, stability, and synthetic transformations. *J. Am. Chem. Soc.* **1989**, *111* (4), 1381-1392.

(11) Laye, C.; Lusseau, J.; Robert, F.; Landais, Y. The Trityl-Cation Mediated Phosphine Oxides Reduction. *Adv. Synth. Catal.* **2021**, *363* (12), 3035-3043.

(12) Chmielewska, E.; Miodowska, N.; Dziuk, B.; Psurski, M.; Kafarski, P. One-Pot Phosphonylation of Heteroaromatic Lithium Reagents: The Scope and Limitations of Its Use for the Synthesis of Heteroaromatic Phosphonates. *Molecules* **2023**, *28* (7), 3135.

(13) Shintani, R.; Fujie, R.; Takeda, M.; Nozaki, K. Silylative Cyclopropanation of Allyl Phosphates with Silylboronates. *Angew. Chem. Int. Ed.* **2014**, *53* (25), 6546-6549.

(14) Craig, D.; King, N. P.; Mountford, D. M. Silyl-modified Belluš–Claisen rearrangement. *Chem. Commun.* **2007**, (10), 1077-1079.

(15) Wu, Z.; Laffoon, J. D.; Nguyen, T. T.; McAlpin, J. D.; Hull, K. L. Rhodium-Catalyzed Asymmetric Synthesis of β-Branched Amides. *Angew. Chem. Int. Ed.* **2017**, *56* (5), 1371-1375.

(16) GUOHUA, F. Preparation method of trans-citral. 2018.

(17) Margot, C.; Matsuda, H.; Schlosser, M. Mixed metal bases as promoters of 1,4-eliminations. *Tetrahedron* **1990**, *46* (7), 2425-2430.

(18) Yoon, T. P.; Dong, V. M.; MacMillan, D. W. C. Development of a New Lewis Acid-Catalyzed Claisen Rearrangement. *J. Am. Chem. Soc.* **1999**, *121* (41), 9726-9727.

(19) Imada, Y.; Shibata, O.; Murahashi, S.-I. Aza- and oxacarbonylations of allyl phosphates catalyzed by rhodium carbonyl cluster. Selective synthesis of β, γ-unsaturated amides, esters, and acids. *J. Organomet. Chem.* **1993**, *451* (1), 183-194.

(20) Torregrosa, J. L.; Baboulene, M.; Speziale, V.; Lattes, A. Hydroboration d'amines insaturees: VII. Stereochimie et mecanisme. *J. Organomet. Chem.* **1984**, *277* (2), 159-172.

(21) Loh, T.-P.; Li, X.-R. A Highly Stereoselective Synthesis of β-Trifluoromethylated Homoallylic Alchols in Water. *Angew. Chem. Int. Ed.* **1997**, *36* (9), 980-982.

(22) Meiß, R.; Kumar, K.; Waldmann, H. Divergent Gold(I)-Catalyzed Skeletal Rearrangements of 1,7-Enynes. *Chem. Eur. J.* **2015**, *21* (39), 13526-13530.

(23) Fischer, D. F.; Xin, Z.-q.; Peters, R. Asymmetric Formation of Allylic Amines with N-Substituted Quaternary Stereocenters by PdII-Catalyzed Aza-Claisen Rearrangements. *Angew. Chem. Int. Ed.* **2007**, *46* (40), 7704-7707.

(24) Angermund, K.; Bogdanović, B.; Koppetsch, G.; Krüger, C.; Mynott, R.; Schwickardi, M.; Tsay, Y.-H. Magnesiumorganische Innerkomplexe, Teil I [1] Bis(dialkylaminoalkyl)- und Bis(alkoxybutyl)magnesium-Verbindungen / Organomagnesium Inner Com plexes, Part I [1] Bis(dialkylaminoalkyl)- and Bis(alkoxybutyl)magnesium Compounds. *Zeitschrift für Naturforschung B* **1986**, *41* (4), 455-466.

(25) Limberger, J.; Claudino, T. S.; Monteiro, A. L. Stereoselective synthesis of (E)-3,3-diaryl and (E)-3-aryl-3-aryloxy allylamines and allylalcohols from trans-cinnamyl chloride and alcohol. *RSC Adv.* **2014**, *4* (85), 45558-45565.

(26) Boulton, K.; Shirley, I.; Smith, I. H.; Whiting, D. A. Mechanism of formation of natural cyclopropanes: synthesis of postulated intermediates in presqualene and chrysanthemyl alcohol biosynthesis. *J. Chem. Soc., Perkin Trans. 1* **1986**, (0), 1817-1824.

(27) Sedelmeier, J.; Ley, S. V.; Baxendale, I. R.; Baumann, M. KMnO4-Mediated Oxidation as a Continuous Flow Process. *Org. Lett.* **2010**, *12* (16), 3618-3621.

(28) Hamlin, T. A.; Kelly, C. B.; Leadbeater, N. E. Dehydrogenation of Perfluoroalkyl Ket-ones by Using a Recyclable Oxoammonium Salt. *Eur. J. Org. Chem.* **2013**, *2013* (18), 3658-3661.

(29) Song, T.; Ma, Z.; Yang, Y. Chemoselective Hydrogenation of α,β-Unsaturated Carbonyls Catalyzed by Biomass-Derived Cobalt Nanoparticles in Water. *ChemCatChem* **2019**, *11* (4), 1313-1319.

(30) Mantilli, L.; Mazet, C. Iridium-catalyzed isomerization of primary allylic alcohols under mild reaction conditions. *Tetrahedron Lett.* **2009**, *50* (28), 4141-4144.

(31) Langer, W.; Seebach, D. Enantioselektive 1,4-Additionen von metallorganischen Verbindungen an konjugierte Systeme im chiralen Medium DDB. *Helv. Chim. Acta.* **1979**, *62* (6), 1710-1722.

(32) Larionov, E.; Lin, L.; Guénée, L.; Mazet, C. Scope and Mechanism in Palladium-Catalyzed Isomerizations of Highly Substituted Allylic, Homoallylic, and Alkenyl Alcohols. *J. Am. Chem. Soc.* **2014**, *136* (48), 16882-16894.

(33) Cong, C.; Fujihara, T.; Terao, J.; Tsuji, Y. Iron oxide catalyzed reduction of acid chlorides to aldehydes with hydrosilanes. *Catal. Commun.* **2014**, *50*, 25-28.

(34) Ebner, C.; Pfaltz, A. Chiral dihydrobenzo[1,4]oxazines as catalysts for the asymmetric transfer-hydrogenation of α,β-unsaturated aldehydes. *Tetrahedron* **2011**, *67* (52), 10287-10290.

(35) Chen, X.; Zhang, Y.; Wan, H.; Wang, W.; Zhang, S. Stereoselective organocatalytic oxidation of alcohols to enals: a homologation method to prepare polyenes. *Chem. Commun.* **2016**, *52* (17), 3532-3535.

(36) Zhao, Y.; Truhlar, D. G. A new local density functional for main-group thermochemistry, transition metal bonding, thermochemical kinetics, and noncovalent interactions. *J. Chem. Phys.* **2006**, *125* (19).

(37) Schäfer, A.; Horn, H.; Ahlrichs, R. Fully optimized contracted Gaussian basis sets for atoms Li to Kr. *J. Chem. Phys.* **1992**, *97* (4), 2571-2577.

(38) Grimme, S.; Antony, J.; Ehrlich, S.; Krieg, H. A consistent and accurate ab initio parametrization of density functional dispersion correction (DFT-D) for the 94 elements H-Pu. *J. Chem. Phys.* **2010**, *132* (15).

(39) Perdew, J. P. Density-functional approximation for the correlation energy of the inhomogeneous electron gas. *Phys. Rev. B* **1986**, *33* (12), 8822-8824.

(40) Schäfer, A.; Huber, C.; Ahlrichs, R. Fully optimized contracted Gaussian basis sets of triple zeta valence quality for atoms Li to Kr. *J. Chem. Phys.* **1994**, *100* (8), 5829-5835.

(41) Marenich, A. V.; Cramer, C. J.; Truhlar, D. G. Universal Solvation Model Based on Solute Electron Density and on a Continuum Model of the Solvent Defined by the Bulk Dielectric Constant and Atomic Surface Tensions. *Phys. Rev. B* **2009**, *113* (18), 6378-6396.

(42) *Gaussian 16 Rev. C.01*; Wallingford, CT, 2016.
